# Abiraterone acetate plus prednisolone with or without enzalutamide for patients with metastatic prostate cancer starting androgen deprivation therapy: final results from two randomised phase 3 trials of the STAMPEDE platform protocol

**DOI:** 10.1016/S1470-2045(23)00148-1

**Published:** 2023-05-01

**Authors:** Gerhardt Attard, Laura Murphy, Noel W Clarke, Ashwin Sachdeva, Craig Jones, Alex Hoyle, William Cross, Robert J Jones, Christopher C Parker, Silke Gillessen, Adrian Cook, Chris Brawley, Clare Gilson, Hannah Rush, Hoda Abdel-Aty, Claire L Amos, Claire Murphy, Simon Chowdhury, Zafar Malik, J Martin Russell, Nazia Parkar, Cheryl Pugh, Carlos Diaz-Montana, Carmel Pezaro, Warren Grant, Helen Saxby, Ian Pedley, Joe M O’Sullivan, Alison Birtle, Joanna Gale, Narayanan Srihari, Carys Thomas, Jacob Tanguay, John Wagstaff, Prantik Das, Emma Gray, Mymoona Alzouebi, Omi Parikh, Angus Robinson, Amir H Montazeri, James Wylie, Anjali Zarkar, Richard Cathomas, Michael D Brown, Yatin Jain, David P Dearnaley, Malcolm D Mason, Duncan Gilbert, Ruth E Langley, Robin Millman, David Matheson, Matthew R Sydes, Louise C Brown, Mahesh K B Parmar, Nicholas D James, for the STAMPEDE investigators

**Affiliations:** Cancer Institute, University College London, London, UK; University College London Hospitals, London, UK; Medical Research Council Clinical Trials Unit, University College London, London, UK; Christie and Salford Royal NHS Foundation Trusts, Manchester, UK; St James’s University Hospital, Leeds, UK; Beatson West of Scotland Cancer Centre, University of Glasgow, Glasgow, UK; Royal Marsden NHS Foundation Trust and Institute of Cancer Research, London, UK; Oncology Institute of Southern Switzerland, Bellinzona, Switzerland; CH and Universita della Svizzera Italiana, Lugano, Switzerland; Medical Research Council Clinical Trials Unit, University College London, London, UK; Medical Research Council Clinical Trials Unit, University College London, London, UK; Guy’s and St Thomas’ NHS Foundation Trust, London, UK; Royal Marsden NHS Foundation Trust and Institute of Cancer Research, London, UK; Medical Research Council Clinical Trials Unit, University College London, London, UK; Guy’s and St Thomas’ NHS Foundation Trust, London, UK; Clatterbridge Cancer Centre NHS Foundation Trust, Wirral, UK; Institute of Cancer Sciences, University of Glasgow, Glasgow, UK; Medical Research Council Clinical Trials Unit, University College London, London, UK; Singleton Hospital, Swansea, UK; Gloucestershire Oncology Centre, Cheltenham, UK; Torbay and South Devon NHS Foundation Trust, Torbay, UK; Northern Centre for Cancer Care, Newcastle upon Tyne, UK; Queen’s University Belfast, Belfast, UK; Rosemere Cancer Centre, Royal Preston Hospital, Preston, UK; Queen Alexandra Hospital, Portsmouth, UK; Shrewsbury and Telford Hospital NHS Trust, Shrewsbury, UK; Kent Oncology Centre, Maidstone, UK; Velindre Cancer Centre, Cardiff, UK; Singleton Hospital, Swansea, UK; Royal Derby Hospital, Derby, UK; Yeovil District Hospital NHS Foundation Trust, Yeovil, UK; Weston Park Hospital, Sheffield, UK; East Lancashire Hospitals NHS Trust, Preston, UK; Royal Sussex County Hospital, Brighton, UK; Clatterbridge Cancer Centre NHS Foundation Trust, Wirral, UK; Christie and Salford Royal NHS Foundation Trusts, Manchester, UK; University Hospitals Birmingham NHS Foundation Trust, Birmingham, UK; Division of Oncology and Hematology, Cantonal Hospital Graubünden, Chur, Switzerland; Swiss Group for Clinical Cancer Research, Bern, Switzerland; Christie and Salford Royal NHS Foundation Trusts, Manchester, UK; Royal Marsden NHS Foundation Trust and Institute of Cancer Research, London, UK; Cardiff University, Cardiff, UK; Medical Research Council Clinical Trials Unit, University College London, London, UK; Faculty of Education Health and Wellbeing, University of Wolverhampton, Walsall, UK; Medical Research Council Clinical Trials Unit, University College London, London, UK; Royal Marsden NHS Foundation Trust and Institute of Cancer Research, London, UK

## Abstract

**Background:**

Abiraterone acetate plus prednisolone (herein referred to as abiraterone) or enzalutamide added at the start of androgen deprivation therapy improves outcomes for patients with metastatic prostate cancer. Here, we aimed to evaluate long-term outcomes and test whether combining enzalutamide with abiraterone and androgen deprivation therapy improves survival.

**Methods:**

We analysed two open-label, randomised, controlled, phase 3 trials of the STAMPEDE platform protocol, with no overlapping controls, conducted at 117 sites in the UK and Switzerland. Eligible patients (no age restriction) had metastatic, histologically-confirmed prostate adenocarcinoma; a WHO performance status of 0–2; and adequate haematological, renal, and liver function. Patients were randomly assigned (1:1) using a computerised algorithm and a minimisation technique to either standard of care (androgen deprivation therapy; docetaxel 75 mg/m^2^ intravenously for six cycles with prednisolone 10 mg orally once per day allowed from Dec 17, 2015) or standard of care plus abiraterone acetate 1000 mg and prednisolone 5 mg (in the abiraterone trial) orally or abiraterone acetate and prednisolone plus enzalutamide 160 mg orally once a day (in the abiraterone and enzalutamide trial). Patients were stratified by centre, age, WHO performance status, type of androgen deprivation therapy, use of aspirin or non-steroidal anti-inflammatory drugs, pelvic nodal status, planned radiotherapy, and planned docetaxel use. The primary outcome was overall survival assessed in the intention-to-treat population. Safety was assessed in all patients who started treatment. A fixed-effects meta-analysis of individual patient data was used to compare differences in survival between the two trials. STAMPEDE is registered with ClinicalTrials.gov (NCT00268476) and ISRCTN (ISRCTN78818544).

**Findings:**

Between Nov 15, 2011, and Jan 17, 2014, 1003 patients were randomly assigned to standard of care (n=502) or standard of care plus abiraterone (n=501) in the abiraterone trial. Between July 29, 2014, and March 31, 2016, 916 patients were randomly assigned to standard of care (n=454) or standard of care plus abiraterone and enzalutamide (n=462) in the abiraterone and enzalutamide trial. Median follow-up was 96 months (IQR 86–107) in the abiraterone trial and 72 months (61–74) in the abiraterone and enzalutamide trial. In the abiraterone trial, median overall survival was 76·6 months (95% CI 67·8–86·9) in the abiraterone group versus 45·7 months (41·6–52·0) in the standard of care group (hazard ratio [HR] 0·62 [95% CI 0·53–0·73]; p<0·0001). In the abiraterone and enzalutamide trial, median overall survival was 73·1 months (61·9–81·3) in the abiraterone and enzalutamide group versus 51·8 months (45·3–59·0) in the standard of care group (HR 0·65 [0·55–0·77]; p<0·0001). We found no difference in the treatment effect between these two trials (interaction HR 1·05 [0·83–1·32]; p_interaction_=0·71) or between-trial heterogeneity (*I*^2^ p=0·70). In the first 5 years of treatment, grade 3–5 toxic effects were higher when abiraterone was added to standard of care (271 [54%] of 498 *vs* 192 [38%] of 502 with standard of care) and the highest toxic effects were seen when abiraterone and enzalutamide were added to standard of care (302 [68%] of 445 *vs* 204 [45%] of 454 with standard of care). Cardiac causes were the most common cause of death due to adverse events (five [1%] with standard of care plus abiraterone and enzalutamide [two attributed to treatment] and one (<1%) with standard of care in the abiraterone trial).

**Interpretation:**

Enzalutamide and abiraterone should not be combined for patients with prostate cancer starting long-term androgen deprivation therapy. Clinically important improvements in survival from addition of abiraterone to androgen deprivation therapy are maintained for longer than 7 years.

**Funding:**

Cancer Research UK, UK Medical Research Council, Swiss Group for Clinical Cancer Research, Janssen, and Astellas.

## Introduction

Until around 10 years ago, patients with metastatic prostate cancer had a median overall survival of approximately 42 months from the start of treatment with long-term androgen-deprivation therapy.^1,2^ Since October 2005, STAMPEDE has used a multiarm multistage platform design^3^ to test in a series of concurrent or sequential phase 3 trials whether additional treatments at the initiation of androgen deprivation therapy improve overall survival. The docetaxel and abiraterone trials^4,5^ of the STAMPEDE platform protocol reported improvements in overall survival and contributed to a change in standard of care for patients with metastatic prostate cancer. The STAMPEDE abiraterone trial^6^ reported survival outcomes for metastatic patients after a median follow-up of 73 months and other trials of second-generation hormone therapies started for the same indication reported final survival analysis after shorter durations of median follow-up, namely 52 months for abiraterone,^7^ 44 months for apalutamide,^8^ and 45 months for enzalutamide.^9^ The STAMPEDE abiraterone trial has now closed, 10 years after initiation of accrual, providing the opportunity to assess new data on long-term efficacy of intensified hormone treatment for patients with metastatic prostate cancer.

The STAMPEDE abiraterone and enzalutamide trial^10^ was initiated after completion of accrual to the abiraterone trial and aimed to improve the efficacy of abiraterone acetate and prednisolone (herein referred to as abiraterone) or enzalutamide by addressing previous observations of an increase in androgen synthesis in patients who received androgen receptor antagonists and of residual hormones that could activate androgen receptor signalling in those who received abiraterone. We hypothesised that the combination of abiraterone and enzalutamide would prevent these consequences of either treatment, delay resistance, and improve efficacy when initiated for patients starting long-term androgen deprivation therapy. Given the expected increased toxic effects, we concluded that a large treatment effect size would be required to change clinical practice. We designed a pragmatic strategy comparing this combination with the standard of care at the time (ie, androgen deprivation therapy) in the STAMPEDE platform protocol and planned to use meta-analysis methods to indirectly compare with the abiraterone trial.^10^

Given that our group and others identified a larger than anticipated survival benefit for patients with metastatic cancer who received abiraterone^4,11^ or enzalutamide,^12^ in 2019, we amended the trial statistical plan to extend the abiraterone and enzalutamide trial and obtain sufficient power to indirectly identify an approximately 15% reduction in risk of death compared with abiraterone alone.^13^ There were no shared control patients and patients in both trials met the same eligibility criteria, underwent similar schedules of assessments, and were recruited at the same hospitals. Here, we report long-term outcomes of patients in the abiraterone trial and primary results from the abiraterone and enzalutamide trial.

## Methods

### Study design and participants

We analysed two open-label, randomised, controlled, phase 3 trials of the STAMPEDE platform protocol,^4,5^ conducted at 117 sites in the UK and Switzerland. The protocol recruited patients with advanced prostate cancer starting androgen deprivation therapy stratified by the presence or absence of distant metastases on conventional imaging (whole body bone scintigraphy or equivalent; CT or MRI of the pelvis, abdomen, and chest; and a chest x-ray if the chest was omitted) within 8 weeks of random assignment. Following a prespecified plan approved by the trial steering committee (independent from the trial management group) on Dec 2, 2019, patients with non-metastatic and metastatic disease were separated for the reporting of long-term outcomes.^13^ Outcomes for patients with metastatic disease in the abiraterone trial after 5 years of follow-up (data extraction on April 3, 2020)^6^ and for those with non-metastatic disease (combined with non-metastatic disease in the abiraterone and enzalutamide trial; data extraction on Aug 3, 2021) have been published.^14^ Trial data for patients with metastatic disease in the abiraterone and enzalutamide trial have not been reported previously. All patients classified as metastatic based on updated information at final database locking are included here.

Eligible patients had metastatic histologically-confirmed prostate adenocarcinoma; a WHO performance status of 0–2; and adequate haematological, renal, and liver function on laboratory tests. There were no age restrictions. Patients who relapsed after primary treatment were eligible when previous androgen deprivation therapy exposure lasted 12 months or less and was completed 12 months or more before random assignment. Patients with confirmed clinically significant cardiovascular disease (eg, severe angina, myocardial infarction, or a history of cardiac failure within 6 months of random assignment) were excluded. Full details of the patient population are provided in the protocol (appendix). All patients provided written informed consent. This trial was done in accordance with Good Clinical Practice guidelines and the Declaration of Helsinki, with relevant regulatory and ethical approvals.

### Randomisation and masking

Patients were randomly assigned (1:1) to either standard of care (control group) or standard of care plus abiraterone (in the abiraterone trial) or plus abiraterone and enzalutamide (in the abiraterone and enzalutamide trial). Each trial randomly assigned patients to its own control group; there were no overlapping controls. Randomisation was performed centrally by telephone using a computerised algorithm. The Medical Research Council Clinical Trials Unit at University College London (London, UK) developed and maintained the algorithm and three unmasked statisticians (LM, AC, and CB) in the same unit analysed the data. Minimisation with a random element of 80% was used with stratification according to randomisation centre, age (<70 *vs* ≥70 years), WHO performance status (0 *vs* 1 or 2), type of androgen deprivation therapy, regular long-term use of aspirin or non-steroidal anti-inflammatory drugs (NSAIDs; yes *vs* no), pelvic nodal status (positive *vs* negative), planned radiotherapy as standard of care (intention or administered; yes *vs* no), and planned docetaxel use before abiraterone and enzalutamide (yes *vs* no). Both trials were open-label because masking of the treatment assignment was deemed impracticable. Eligible patients could be allocated to any of the trials that were contemporaneously recruiting patients in the STAMPEDE platform protocol (appendix p 15).

### Procedures

The study protocol required patients to start life-long androgen deprivation therapy no longer than 12 weeks before random assignment. The standard of care was androgen deprivation therapy with luteinising hormone-releasing hormone agonists or antagonists or bilateral orchiectomy; after implementation of a protocol amendment (version 13; Dec 17, 2015), docetaxel (75 mg/m^2^) intravenously for six cycles with prednisolone (10 mg) orally once per day were permitted after starting androgen deprivation therapy but before starting study treatment. Radiotherapy was allowed for symptom palliation.

Study treatment was administered until radio-graphical progression or any reason that required discontinuation. Abiraterone acetate (1000 mg) and prednisolone (5 mg; prednisone at Swiss sites) were given orally once a day in the abiraterone trial and together with enzalutamide (160 mg) orally once a day in the abiraterone and enzalutamide trial. Dose modifications are described in the protocol (appendix).

Patients were assessed every 6 weeks during the first 6 months, once every 12 weeks until year 2, once every 6 months until year 5, and then once per year. Assessments included prostate-specific antigen (PSA) testing, safety laboratory tests, and ascertainment of adverse events; further tests were conducted at the discretion of the treating physician. The nadir PSA concentration was the lowest PSA concentration within 24 weeks after randomisation and was used to calculate PSA progression (biochemical failure) as defined in the protocol (appendix). After randomisation, imaging frequency occurred as per local guidelines. Investigator determined radiographical or local progression was reported in line with guidelines. Reporting of skeletal-related events was updated following a protocol amendment to distinguish whether this constituted progression (STAMPEDE protocol version 12; July 29, 2014). Adverse events were assessed by the National Cancer Institute Common Terminology Criteria for Adverse Events (version 3.0 updated to 4.0 in STAMPEDE protocol version 15; May 15, 2016). Serious adverse reactions were also reported. Race or ethnicity data were not collected. Pre-randomisation bone scans were retrieved to the Christie Hospital (Manchester, UK) central imaging repository after completion of accrual. Physicians (AS, CJ, AH, and YJ) classified patients by high-volume or low-volume disease using the CHAARTED trial criteria^2,15^ based on the number and site of bone metastases and whether local sites stated at randomisation that visceral disease was present on CT scan. Ascertainment of death from prostate cancer was determined using a prespecified algorithm or manual review by a panel of clinicians according to an agreed set of rules and without knowledge of randomised treatment allocation.

### Outcomes

The primary outcome was overall survival for both trials (defined as the time from randomisation to death from any cause).

Secondary outcomes were prostate cancer-specific survival (defined as the time from randomisation to death from prostate cancer); failure-free survival (defined as biochemical failure, local or lymph node progression, distant metastases, or death from prostate cancer); progression-free survival (defined as failure-free survival but excluding biochemical failure); metastatic progression-free survival (defined as progression of or new distant metastases or death from prostate cancer); symptomatic skeletal-related events (defined as pathological fracture or spinal cord compression or bone pain requiring radiotherapy or surgery); toxic effects and adverse events; and cost-effectiveness of research treatment. Cost-effectiveness was reported previously for the abiraterone trial^16^ and could be updated in the future based on longer-term follow-up included here.

### Statistical analysis

Efficacy analyses were specified in the STAMPEDE protocol. We stated our intention to include both trials in the meta-analysis before initiation of accrual to the abiraterone and enzalutamide trial^10^ and again, in the update to report outcomes for patients with high-risk localised and metastatic disease.^13^

Original sample size calculations were based on recruitment of at least 1800 patients with high-risk localised or metastatic disease to either trial and aimed to detect an event-driven overall hazard ratio (HR) of at least 0·75 with 90% power at two-sided 5% significance, assuming a 4-year median overall survival with standard of care. Both trials recruited the target sample size and there was no predefined sample size for patients with metastatic disease alone. There were three interim analyses in the abiraterone trial and two in the abiraterone and enzalutamide trial that all concluded with a recommendation to continue as planned. The decision to close these trials and timing of these analyses was based on funding availability.

Efficacy analyses were done in the intention-to-treat (ITT) population (defined as all randomly assigned patients). Toxic effects and adverse events were analysed in the safety population (defined as patients who started treatment within randomly assigned groups; reported as the maximum grade per patient for each adverse event) in the first 5 years of treatment to have enough representative patients in both control and study drug groups. To account for different durations of treatment exposure in each group, the time to any grade 3–5 event was analysed using a cumulative incidence curve.

Standard survival analysis methods including Cox proportional hazards regression and Kaplan-Meier curves were used to analyse and present time-to-event data. 95% CIs were calculated using the standard error of Kaplan-Meier survival estimates or survival HRs. Estimates were adjusted for stratification factors (except randomisation centre and androgen deprivation therapy type) and stratified according to accrual time periods defined by other recruiting trials within STAMPEDE. Patients without an event triggering an outcome definition were censored when last known to be event free according to a completed follow-up case report form. Adverse events of interest were liver derangement, hypertension, and fatigue reported previously as commonly occurring with abiraterone or enzalutamide.^4,7,12^ The proportional hazards assumption was tested using scaled Schoenfeld residuals regression over time. In case of non-proportional hazards in the treatment effect, restricted mean survival time-to-event difference was added from a flexible parametric model with time-varying treatment effect. Prostate cancer-specific survival used a competing risks approach with death from non-prostate cancer causes as the competing risk. Fixed-effects meta-analyses of individual patient data incorporating Cox proportional hazards were used to pool estimates from both trials using the ipdmetan module to compare inter-trial differences.^17^ Heterogeneity between trials was calculated using Cochran’s Q test (*I*^2^).

Prespecified subgroup analyses measured consistency of the treatment effect between both trials and across randomisation stratification factors (age, nodal stage, WHO status, NSAID or aspirin use, and planned docetaxel use as standard of care) using meta-analysis pooling of the interaction effect estimate between treatment allocation and each subgroup. Planned radiotherapy, randomising centre, and choice of androgen deprivation therapy were stratification factors but due to a small number of patients in one or more of the subgroups, were not included in the analysis. In case of unexpected significant interactions, a post-hoc Pearson correlation test was done to evaluate the association between subgroups.

Given that metastatic volume is prognostic, outcomes split by low-volume and high-volume disease are of interest and were included as an additional prespecified subgroup analysis, in line with other trials of combination therapies in this setting.^8,9,12^

The significance threshold for p values was set at 0·05 and data were analysed using Stata (version 17). STAMPEDE is registered with ClinicalTrials.gov (NCT00268476) and ISRCTN (ISRCTN78818544).

### Role of the funding source

Cancer Research UK approved the study design and subsequent amendments. Janssen and Astellas approved the study design, participated in discussions on progress of the trial, and were invited to comment on the manuscript. The funders of the study had no role in data collection, data analysis, or data interpretation.

## Results

**Figure 1:**
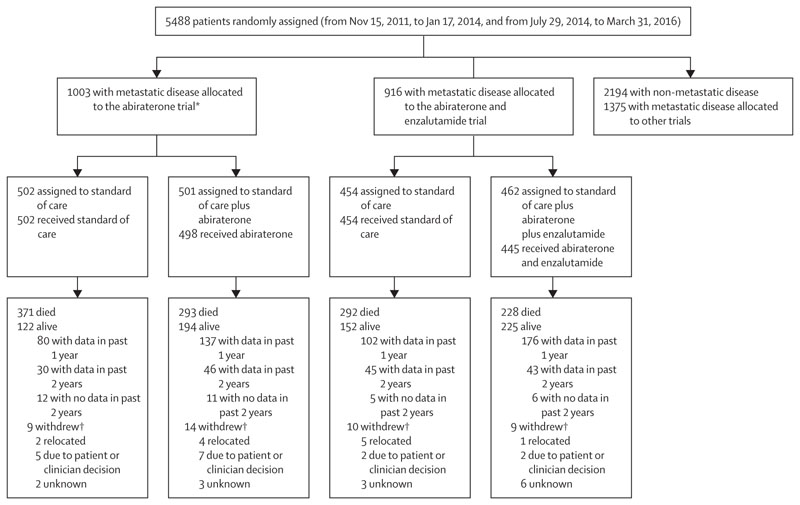
Trial profile Patients who did not start research treatment are not included in the safety analysis. All randomly assigned patients are included in the efficacy analyses. Abiraterone refers to abiraterone acetate and prednisolone. *Metastatic status was updated by recruiting sites for 11 patients after random assignment and after first report of the abiraterone trial;^4^ status was changed for six patients from non-metastatic to metastatic disease and for five patients from metastatic to non-metastatic disease. †Patients who withdrew and did not have data in the past 2 years; reasons were provided as free text by site and categorised centrally.

Between Nov 15, 2011, and Jan 17, 2014, 1003 patients with metastatic prostate cancer were randomly assigned to standard of care (n=502) or standard of care plus abiraterone (n=501) in the abiraterone trial. Between July 29, 2014, and March 31, 2016, 916 patients were randomly assigned to standard of care (n=454) or standard of care plus abiraterone plus enzalutamide (n=462) in the abiraterone and enzalutamide trial (figure 1). 5488 patients were randomly assigned in the STAMPEDE platform protocol during accrual to the two trials. 1974 patients with non-metastatic disease were randomly assigned in the abiraterone or abiraterone and enzalutamide trials, as reported previously,^10^ and 220 patients with non-metastatic disease and 1375 with metastatic disease were allocated to other trials (appendix p 15).

All randomly assigned patients in the abiraterone or abiraterone and enzalutamide trials were included in the efficacy analysis. 23 (2%) patients withdrew from further follow-up in the abiraterone trial, whereas 19 (2%) withdrew from further follow-up in the abiraterone and enzalutamide trial and were censored at the date of withdrawal.

Baseline characteristics were well balanced between the two groups (table 1; appendix p 2). The median age of patients was 67 years (IQR 62–71) in the abiraterone trial and 69 years (63–74) in the abiraterone and enzalutamide trial. 498 (99%) of 501 patients started the assigned abiraterone and 445 (96%) of 462 started abiraterone and enzalutamide treatment. No other patients in the abiraterone and enzalutamide trial reported starting abiraterone or enzalutamide. The median time from randomisation to starting abiraterone was 10 days (IQR 6–18) and the median time to starting abiraterone and enzalutamide was 13 days (7–24; appendix p 16). Across both trials, the median interval between starting androgen deprivation therapy and study treatment was 9·2 weeks (6·3–12·0). Data collection closed on Nov 30, 2021, for both trials; after data cleaning, the final database lock was on July 3, 2022. At trial closure, 117 (23%) of 498 patients continued abiraterone with no indication for treatment change in the abiraterone trial and 98 (22%) of 445 continued abiraterone and enzalutamide; three (1%) continued only enzalutamide and ten (2%) continued only abiraterone in the abiraterone and enzalutamide trial.

**Figure 2:**
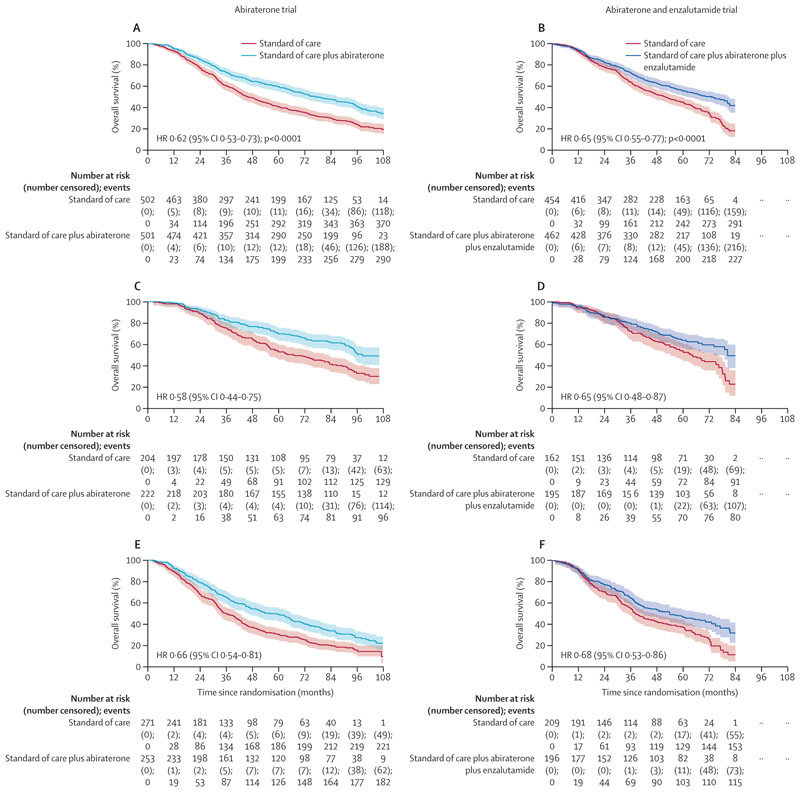
Overall survival analysis Shaded regions represent 95% CIs. Survival curves for the abiraterone and enzalutamide trial are capped at 84 months due to shorter follow-up than in the abiraterone trial. Abiraterone refers to abiraterone acetate and prednisolone. Overall survival in the abiraterone trial (A) and the abiraterone and enzalutamide trial (B), split by low-volume metastatic disease (C, D) and high-volume metastatic disease (E, F). Patients with unknown metastatic volume were not included in the subgroup analysis by metastatic volume.

After a median follow-up of 96 months (IQR 86–107), 664 (66%) of 1003 patients died in the abiraterone trial and after a median follow-up of 72 months (61–74), 520 (57%) of 916 died in the abiraterone and enzalutamide trial. Overall survival was significantly longer with the addition of study treatment to standard of care versus standard of care alone in both trials. In the abiraterone trial, median overall survival was 76·6 months (95% CI 67·8–86·9) in the abiraterone group versus 45·7 months (41·6–52·0) in the standard of care group (hazard ratio [HR] 0·62 [95% CI 0·53–0·73]; p<0·0001; figure 2). In the abiraterone and enzalutamide trial, median overall survival was 73·1 months (61·9–81·3) in the abiraterone and enzalutamide group versus 51·8 months (45·3–59·0) in the standard of care group (HR 0·65 [0·55–0·77]). We found no difference in the treatment effect between these two trials (interaction HR 1·05 [0·83–1·32]; p_interaction_**=**0·71) and no evidence of between-trial heterogeneity (*I*^2^ p=0·70).

For the primary outcome, we found non-proportional hazards in the abiraterone and enzalutamide trial but not in the abiraterone trial. Further investigation confirmed some non-proportional hazards for prostate cancer-specific and failure-free survival in both trials (appendix pp 3, 17). Deviation from proportional hazards can lead to a reduction in power but all outcome measures showed strong benefit. We conducted a prespecified analysis of restricted mean survival time for overall survival up to 84·0 months. The restricted mean survival times were 50·4 months (95% CI 48·1–52·8) with standard of care versus 60·6 months (58·2–63·0) with addition of abiraterone (difference 10·2 months [6·8–13·5]; p<0·0001) and 51·3 months (48·8–53·8) with standard of care versus 58·6 months (56·0–61·1) with addition of abiraterone and enzalutamide (difference 7·3 months [3·7–10·9]; p<0·0001). At 84 months in the abiraterone trial, overall survival was 30% (95% CI 26–34) with standard of care versus 48% (43–52) with standard of care plus abiraterone.

**Figure 3:**
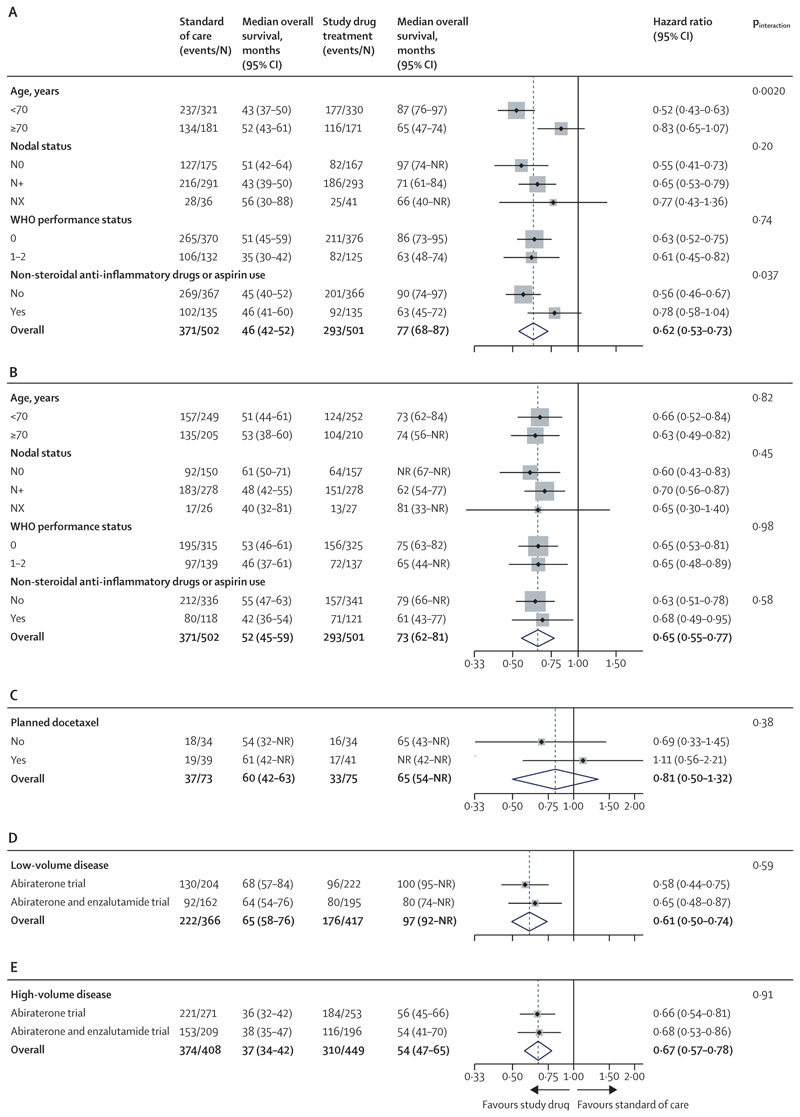
Overall survival subgroup analysis The dashed line indicates overall hazard ratio. Weighting is by sample size. Abiraterone refers to abiraterone acetate and prednisolone. Prespecified stratification factors at the start of accrual in the abiraterone trial with standard of care plus abiraterone treatment (A) and in the abiraterone and enzalutamide trial with standard of care plus abiraterone plus enzalutamide treatment (B) and by planned use of docetaxel, allowed after amendment of the abiraterone and enzalutamide trial (C). Additional prespecified analysis shows low-volume metastatic disease (D) and high-volume metastatic disease (E). NR=not reached.

In prespecified subgroup analyses by stratification factors, there was an interaction with NSAID or aspirin use at randomisation (p_interaction_**=**0·037) and age (p_interaction_**=**0·0020) in the abiraterone trial, which was not seen in the abiraterone and enzalutamide trial (figure 3A, B). There was a correlation between age and use of NSAID or aspirin in the abiraterone trial (Pearson χ^2^ p=0·0015), with more patients aged 70 years or older using NSAID or aspirin (116 [33%] of 352 *vs* 154 [24%] of 651 aged <70 years). In the abiraterone and enzalutamide trial, a similar proportion of patients used NSAID or aspirin in both age groups (109 [26%] of 415 aged ≥70 *vs* 130 [26%] of 501 aged <70).

There was no interaction with nodal status or WHO performance status. Docetaxel was allowed as standard of care for the last 148 (16%) patients randomly assigned in the abiraterone and enzalutamide trial. 80 (54%) of 148 patients were planned for docetaxel and were younger than those who were not offered docetaxel (median age 66·5 years [IQR 61·5–71·0] *vs* 69·0 [65.0–73·0]; appendix p 4). There was no interaction for planned docetaxel use (p_interaction_**=**0·38; figure 3C). Additional prespecified subgroup analysis identified no difference in the effect of study treatment in patients split by low-volume (p=0·59) or high-volume metastatic disease (p=0·91; figure 3D, E) and no evidence of a different treatment effect between these patients (p_interaction_**=**0·45).

**Figure 4:**
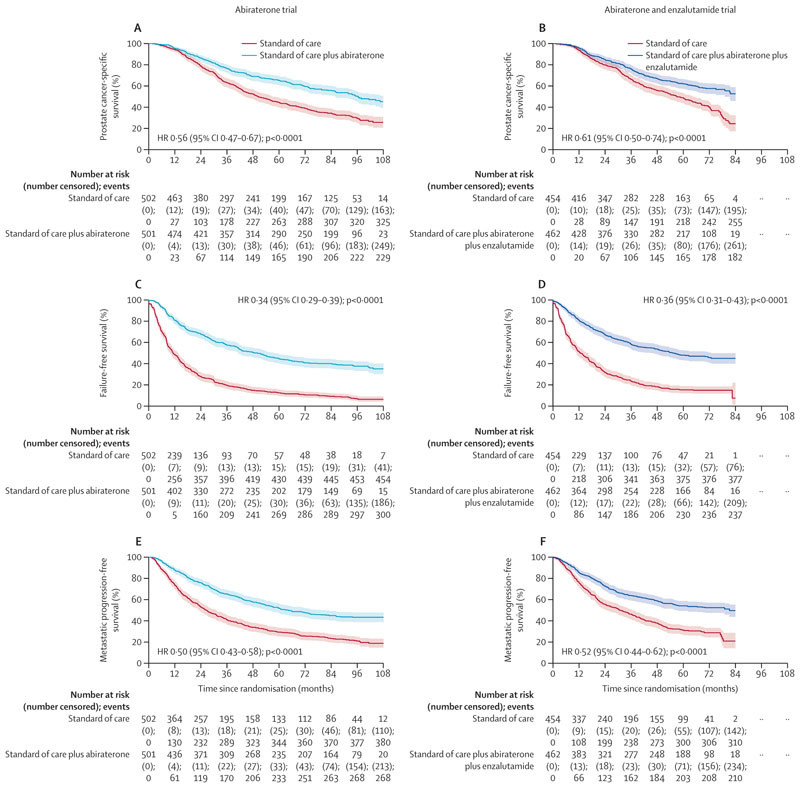
Secondary efficacy endpoint analysis Shaded regions represent 95% CIs. Survival curves for the abiraterone and enzalutamide trial are capped at 84 months due to shorter follow-up than in the abiraterone trial. Abiraterone refers to abiraterone acetate and prednisolone. Prostate cancer-specific survival (A, B), failure free survival (C, D) and metastatic progression free survival (E, F) in the abiraterone and abiraterone and enzalutamide trials.

325 (88%) of 371 deaths with standard of care and 231 (79%) of 293 with addition of abiraterone were attributed to prostate cancer. Similarly, 256 (88%) of 292 deaths with standard of care and 182 (80%) of 228 with addition of abiraterone and enzalutamide were attributed to prostate cancer. There was a statistically and clinically significant improvement in prostate cancer-specific survival in both trials with study drug treatment (HR 0·56 [95% CI 0·47–0·67]; p<0·0001 in the abiraterone trial and HR 0·61 [0·50–0·74]; p<0·0001 in the abiraterone and enzalutamide trial), with no difference in effect between the two trials (figure 4A, B). Improvements in all other secondary outcomes were significant and consistent across the abiraterone versus abiraterone and enzalutamide trials in terms of failure-free survival (HR 0·34 [0·29–0·39]; p<0·0001 *vs* HR 0·36 [0·31–0·43]; p<0·0001; figure 4C, D; appendix pp 5, 18), progression-free survival (HR 0·47 [0·40–0·55]; p<0·0001 *vs* HR 0·50 [0·42–0·59]; p<0·0001; appendix p 19), metastatic progression-free survival (HR 0·50 [0·43–0·58]; p<0·0001 *vs* HR 0·52 [0·44–0·62]; p<0·0001; figure 4E, F), and symptomatic skeletal-related events (HR 0·76 [0·57–0·99]; p=0·045 *vs* HR 0·61 [0·47–0·79]; p=0·00020; appendix p 20).

Of patients who reported progression before trial closure, 322 (71%) of 455 and 266 (71%) of 377 in the control groups of the abiraterone or abiraterone and enzalutamide trials, respectively, started a life prolonging treatment. Whereas 175 (58%) of 301 who received standard of care with abiraterone and 115 (49%) of 237 who received standard of care with abiraterone and enzalutamide started a life-prolonging treatment after progression. Of these patients, the proportion of those who received abiraterone or enzalutamide or docetaxel first at progression in the abiraterone trial was 82 (25%), 85 (26%), and 147 (46%), respectively, after standard of care compared with six (3%), 22 (13%), and 126 (72%) after standard of care and abiraterone (appendix p 21). In the abiraterone and enzalutamide trial, the proportion of patients who received abiraterone or enzalutamide or docetaxel first was 60 (23%), 117 (44%), and 81 (30%), respectively, after standard of care compared with six (3%), seven (3%), and 88 (77%) after standard of care plus abiraterone and enzalutamide.

Disease progression was the most common reason reported for permanently stopping treatment (202 [41%] of 498 in the abiraterone trial *vs* 151 [34%] of 445 stopping either treatment in the abiraterone and enzalutamide trial; appendix pp 6, 22). In the abiraterone and enzalutamide trial, 66 (15%) patients reported stopping both treatments because of adverse effects. 54 (12%) patients reported adverse effects as the reason for stopping abiraterone and 14 (3%) as the reason for stopping enzalutamide. Whereas 65 (13%) patients in the abiraterone trial reported stopping abiraterone due to toxic effects. In the first 5 years, grade 3–5 toxic effects were higher when abiraterone was added to standard of care (271 [54%] of 498 *vs* 192 [38%] of 502 with standard of care) and higher toxic effects were seen when abiraterone and enzalutamide were added to standard of care (302 [68%] of 445 *vs* 204 [45%] of 454 with standard of care; table 2; appendix pp 7–13).

In a post-hoc analysis, the higher rates of grade 3–5 adverse events with study treatment than with standard of care were observed from the start of treatment (appendix p 23). Grade 1–4 adverse events of interest that occurred most frequently were fatigue (342 [69%] of 498 with standard of care plus abiraterone *vs* 272 [54%] of 502 with standard of care in the abiraterone trial and 382 [86%] of 445 with standard of care plus abiraterone and enzalutamide *vs* 309 [68%] of 454 with standard of care in the abiraterone and enzalutamide trial), hypertension (187 [37%] *vs* 64 [13%] and 256 [58%] *vs* 72 [15%]), and alanine aminotransferase or aspartate aminotransferase increase (or both; 136 [27%] *vs* 72 [14%] and 166 [37%] *vs* 83 [18%]; table 2; appendix pp 7–13, 23). Cardiac causes were the most common cause of death due to adverse events (five [1%] with standard of care plus abiraterone and enzalutamide [two attributed to treatment] and one (<1%) with standard of care in the abiraterone trial; appendix pp 14, 24).

## Discussion

Combining abiraterone and enzalutamide with androgen deprivation therapy is more effective than androgen deprivation therapy alone, but from an indirect comparison we showed no improvement over abiraterone and androgen deprivation therapy. We have not formally compared the abiraterone and enzalutamide trial with trials testing enzalutamide. But, given the similar efficacy reported when combining enzalutamide with androgen deprivation therapy,^12^ we also conclude that combining abiraterone with enzalutamide would not be more effective. Overall, increased toxicity with the combination of abiraterone and enzalutamide does not justify further exploration in unselected patients.

Combining apalutamide or enzalutamide with abiraterone for patients with metastatic castration-resistant prostate cancer might improve progression-free survival but consistent with our findings, these trials showed no improvement in overall survival.^18–20^ Similarly, there was no improvement in overall survival of patients with high-risk localised prostate cancer when abiraterone was indirectly compared with abiraterone and enzalutamide (administered as adjuvant therapy for 2 years).^14^ Therefore, treatment efficacy with abiraterone is unlikely to be reduced by an increase in androgens or reactivation of ligand-binding domain-dependent reactivation of androgen receptor signalling (given that combining abiraterone with an effective androgen receptor antagonist, enzalutamide, has no discernible long-term benefit). This finding is consistent with the low response rate observed from sequential use of either treatment.^21^

The periods of accrual to both trials overlapped with another separate trial that randomly assigned patients with metastatic cancer to radiotherapy to the primary tumour and reported improved overall survival for those with low-volume metastatic disease.^22^ Although abiraterone-containing trials shared patients who received standard of care (control) with the radiotherapy trial, none were randomly assigned to the study drug groups so we are unable to comment on the interaction between radiotherapy and hormone intensification.

By leveraging resources across several trials, our follow-up of patients allocated to abiraterone was longer than feasible in other trials, which allowed us to make additional observations. Notably, the survival benefit of starting abiraterone with androgen deprivation therapy is maintained with longer follow-up and 23% of patients in the abiraterone trial and 22% in the abiraterone and enzalutamide trial continued abiraterone with no indication for a treatment change after more than 82 months of follow-up.

We also report fewer deaths attributed to prostate cancer in patients who received abiraterone with or without enzalutamide than in those who received standard of care (413 [79%] of 521 in treatment groups *vs* 581 [88%] of 663 in standard of care groups), suggesting that for some patients with metastatic disease hormone intensification at the start of androgen deprivation therapy controls the prostate cancer for long enough for death from other causes to occur.

Since 2022, the benefit of triplet therapy with androgen deprivation therapy supplemented by docetaxel and either abiraterone or enzalutamide, or darolutamide has been debated. As with previous trials,^8,12,23,24^ none of the patients who received androgen deprivation therapy and abiraterone or enzalutamide were randomly assigned to docetaxel but a small subgroup in the abiraterone and enzalutamide trial received docetaxel as standard of care. The point estimates for risk of death in patients with planned use of docetaxel versus those with no planned use of docetaxel were numerically different but we found no statistically significant interaction. Conclusions on the efficacy of docetaxel given before abiraterone and enzalutamide are confounded by physicians electing to use docetaxel for younger patients and putatively more aggressive cancers on clinical and pathological criteria and are limited by the small number of patients.

The interaction with NSAID or aspirin is statistically significant but not clinically significant. Moreover, the interaction is in the opposite direction to that observed in patients with non-metastatic disease recruited to STAMPEDE trials.^14^

Our trial design was limited to only detect large benefits from combining enzalutamide and abiraterone with androgen deprivation therapy. However, given the toxicity and cost of abiraterone and enzalutamide, missing small differences in treatment effect is an acceptable limitation. There was a 2-year difference in accrual periods that, given the changes in clinical practice in the past 10 years, resulted in patients allocated to the abiraterone and enzalutamide trial receiving different treatment sequences after progression than those in the abiraterone trial. We provided updated information on first therapy instituted at trial-defined progression in the abiraterone trial and new data from the abiraterone and enzalutamide trial. Given that funding for these agents before chemotherapy became more widely available in the UK National Health Service after 2015, more patients had this option at progression in the abiraterone and enzalutamide trial than in the abiraterone trial. Collection and reporting of treatment after progression can be difficult, particularly for later lines and when patients move hospitals or join subsequent trials. Although we anticipate the use of subsequent treatments might be under-reported, we have no reason to think there is bias according to the allocated treatment in these trials. We do not have a detailed assessment of the long-term metabolic toxic effects of intensified hormone treatment plus androgen deprivation therapy and abiraterone with or without enzalutamide. Any increase in mortality from treatment toxicity is offset by the improvement in prostate cancer control, but given the increased life expectancy, management of long-term treatment effects should be prioritised in clinical practice and future trials.

The excellent outcomes for 23% of patients who continued abiraterone or abiraterone and enzalutamide at trial closure introduces concerns regarding the risks of harm from continuous treatment to progression, especially for unselected patients with low-volume disease for whom treatment deintensification clinical trial protocols should be considered. Conversely, these and previous trials have reported that despite hormone intensification, a substantial proportion of patients with metastatic prostate cancer relapse and die within 36 months and identification of these patients to offer additional treatment options is a high priority for future trials. Both our trials have closed to further data collection, although we might explore long-term patient outcome monitoring using linkage with health-care systems data.^25^ In conclusion, enzalutamide and abiraterone should not be combined for patients with prostate cancer starting long-term androgen deprivation therapy; but clinically important improvements in overall survival from addition of abiraterone to androgen deprivation therapy are maintained for longer than 7 years.

## Supplementary Material

Appendix

## Figures and Tables

**Table 1:** Baseline characteristics

	Abiraterone trial			Abiraterone and enzalutamide trial	
	Standard of care (n=502)	Standard of care plus abiraterone (n=501)		Standard of care (n=454)	Standard of care plus enzalutamide and abiraterone (n=462)
**Age, years**					
Median	67 (62–72)	67 (62–71)		68 (64–73)	69 (63–74)
Min-max	39–84	42–85		37–83	48–84
**Prostate-specific antigen before androgen deprivation therapy, ng/mL**					
Median	97 (26–358)	96 (29–371)		97 (25–319)	85 (26–337)
Min-max	1–10530	0–21460		0–20590	1–6881
**Time from diagnosis to randomisation, days**					
Median	71 (51–95)	77 (54–97)		75 (56–99)	78 (59–100)
Min-max	0–4866	3–5384		4–5346	2–4676
**T stage**					
T0–T2	56 (11%)	51 (10%)		42 (9%)	47 (10%)
T3	270 (54%)	289 (58%)		252 (56%)	273 (59%)
T4	137 (27%)	118 (24%)		117 (26%)	95 (21%)
Tx	39 (8%)	43 (9%)		43 (9%)	47 (10%)
**N stage (pelvic nodes)**					
N0	175 (35%)	167 (33%)		150 (33%)	157 (34%)
N+	291 (58%)	293 (58%)		278 (61%)	278 (60%)
NX	36 (7%)	41 (8%)		26 (6%)	27 (6%)
**Metastatic volume**					
Low	204 (41%)	222 (44%)		162 (36%)	195 (42%)
High	271 (54%)	253 (51%)		209 (46%)	196 (42%)
Missing	27 (5%)	26 (5%)		83 (18%)	71 (15%)
**Gleason Score**					
≤7	118 (24%)	115 (23%)		92 (20%)	79 (17%)
8	106 (21%)	117 (23%)		88 (19%)	94 (20%)
9	245 (49%)	231 (46%)		239 (53%)	258 (56%)
10	23 (5%)	18 (4%)		18 (4%)	17 (4%)
Unknown	10 (2%)	20 (4%)		17 (4%)	14 (3%)
**WHO performance status**					
0	370 (74%)	376 (75%)		315 (69%)	325 (70%)
1	125 (25%)	118 (24%)		134 (30%)	129 (28%)
2	7 (1%)	7 (1%)		5 (1%)	8 (2%)
**Pain from prostate cancer**					
Absent	396 (79%)	389 (78%)		374 (82%)	368 (80%)
Present	102 (20%)	107 (21%)		78 (17%)	86 (19%)
Unknown	4 (1%)	5 (1%)		2 (<1%)	8 (2%)
**Aspirin use**					
No	412 (82%)	411 (82%)		374 (82%)	377 (82%)
Yes	90 (18%)	90 (18%)		80 (18%)	85 (18%)
**Non-steroidal anti-inflammatory drug use**					
No	448 (89%)	449 (90%)		411 (91%)	419 (91%)
Yes	54 (11%)	52 (10%)		43 (9%)	43 (9%)
**Planned or current hormone therapy**					
Orchiectomy	3 (1%)	3 (1%)		0	1 (<1%)
Luteinising hormone-releasing hormone agonists or antagonists	497 (99%)	498 (99%)		452 (100%)	460 (100%)
Bicalutamide	1 (<1%)	0		1 (<1%)	1 (<1%)
Maximum androgen blockade	1 (<1%)	0		1 (<1%)	0
**Palliative radiotherapy planned as standard of care**					
No	479 (95%)	480 (96%)		454 (100%)	460 (100%)
Yes	23 (5%)	21 (4%)		0	2 (<1%)
**Previous treatment to prostate**					
No	475 (95%)	466 (93%)		424 (93%)	432 (94%)
Yes	27 (5%)	35 (7%)		30 (7%)	30 (6%)
**Docetaxel planned as standard of care**					
No	0	0		34 (7%)	34 (7%)
Yes	0	0		39 (9%)	41 (9%)
Not applicable (before change in standard of care)	502 (100%)	501 (100%)		381 (84%)	387 (84%)

Data are median (IQR), min-max, or n (%). Abiraterone refers to abiraterone acetate and prednisolone. Additional characteristics are shown in the appendix (p 2).

**Table 2:** Summary of adverse events

	Standard of care in the abiraterone and enzalutamide trial (n=454)					Standard of care plus abiraterone and enzalutamide in the abiraterone and enzalutamide trial (n=445)			
	Grade 1-2	Grade 3	Grade 4	Grade 5		Grade 1-2	Grade 3	Grade 4	Grade 5
Blood and lymphatic	193 (43%)	21 (5%)	5 (1%)	0		217 (49%)	9 (2%)	4 (1%)	0
Cardiac	25 (6%)	10 (2%)	5 (1%)	0		45 (10%)	19 (4%)	7 (2%)	5 (1%)*
Eye	54 (12%)	5 (1%)	0	0		73 (16%)	6 (1%)	0	0
Gastrointestinal and hepatobiliary	242 (53%)	19 (4%)	4 (1%)	1 (<1%)		305 (69%)	30 (7%)	6 (1%)	0
General disorders and administration site conditions	329 (72%)	29 (6%)	1 (<1%)	0		348 (78%)	54 (12%)	2 (<1%)	2 (<1%)
Fatigue	296 (65%)	13 (3%)	0	0		348 (78%)	34 (8%)	0	0
Infections	89 (20%)	17 (4%)	1 (<1%)	1 (<1%)		101 (23%)	22 (5%)	3 (1%)	3 (1%)
Injury	8 (2%)	6 (1%)	0	0		23 (5%)	11 (2%)	0	0
Investigations	216 (48%)	25 (6%)	10 (2%)	0		246 (55%)	66 (15%)	10 (2%)	0
Alanine aminotransferase increased	75 (17%)	3 (1%)	0	0		112 (25%)	44 (10%)	1 (<1%)	0
Aspartate aminotransferase increased	17 (4%)	0	0	0		43 (10%)	9 (2%)	0	0
Metabolism and nutrition	143 (31%)	6 (1%)	0	0		190 (43%)	17 (4%)	3 (1%)	0
Glucose intolerance	31 (7%)	1 (<1%)	0	0		23 (5%)	7 (2%)	0	0
Hypokalaemia	21 (5%)	0	0	0		66 (15%)	7 (2%)	2 (<1%)	0
Musculoskeletal	288 (63%)	45 (10%)	0	0		292 (66%)	54 (12%)	0	0
Nervous system	144 (32%)	15 (3%)	1 (<1%)	0		232 (52%)	20 (4%)	6 (1%)	0
Seizure	1 (<1%)	0	0	0		0	4 (1%)	0	0
Cognitive disturbance	22 (5%)	2 (<1%)	0	0		83 (19%)	2 (<1%)	1 (<1%)	0
Psychiatric	187(41%)	6 (1%)	0	0		230 (52%)	3 (1%)	1 (<1%)	0
Renal and urinary	292 (64%)	17 (4%)	5 (1%)	0		306 (69%)	18 (4%)	5 (1%)	2 (<1%)
Reproductive	179 (39%)	58 (13%)	0	0		194 (44%)	78 (18%)	0	0
Respiratory	161 (35%)	9 (2%)	2 (<1%)	0		196 (44%)	12 (3%)	1 (<1%)	1 (<1%)
Skin	121 (27%)	4 (1%)	1 (<1%)	0		167 (38%)	3 (1%)	1 (<1%)	0
Vascular	365 (80%)	24 (5%)	1 (<1%)	0		324 (73%)	93 (21%)	1 (<1%)	0
Hypertension	60 (13%)	12 (3%)	0	0		187 (42%)	69 (16%)	0	0
Hot flushes	365 (80%)	12 (3%)	0	0		374 (84%)	24 (5%)	0	0

Grade 1–2 adverse events occurring in 10% or more of patients in any treatment group or any grade 3, 4, or 5 adverse events stratified by body system, including adverse events of special interest. Abiraterone refers to abiraterone acetate and prednisolone. All adverse events in the abiraterone trial are shown in the appendix (p 7). *Events on old form categorised as “Cardiovascular—other” have been included in the cardiac body system.

## Data Availability

Individual participant data are available upon request to the corresponding author and after deidentification, as per the moderated access approach of the Medical Research Council Clinical Trials Unit at University College London.

## Research in context

### Evidence before this study

We searched for randomised controlled trials published in English in MEDLINE from Jan 1, 1966, to June 30, 2021, Embase from Jan 1, 1982, to June 30, 2021, and major urology and oncology conference proceedings from Jan 1, 1990, to June 30, 2021, using search terms including (“metastatic” AND “prostate cancer” AND “sensitive”) AND (“abiraterone” OR “enzalutamide” OR “apalutamide” OR “darolutamide”). There were no randomised trials that tested the combination of abiraterone and one of the androgen receptor antagonists for patients with metastatic hormone-sensitive prostate cancer. However, six trials that randomly assigned patients to one of these agents reported improvements in survival and three of these reported final survival analysis after a maximum median follow-up of 51 months.

### Added value of this study

To our knowledge, this is the first phase 3 randomised trial evaluating abiraterone with an androgen receptor antagonist added at the start of androgen deprivation therapy for patients with metastatic prostate cancer. Our meta-analysis on two trials performed using a platform protocol compares abiraterone and enzalutamide added to androgen deprivation therapy versus abiraterone plus androgen deprivation therapy, including updated survival data after a median follow-up of 96 months (IQR 86–107). Extended follow-up of patients starting intensified hormone therapy for metastatic prostate cancer—now the standard of care—shows clinically significant survival benefits of adding abiraterone to androgen deprivation therapy, which were maintained after 84 months. Combining androgen deprivation therapy, abiraterone, and enzalutamide increases toxic effects but does not improve efficacy versus abiraterone plus androgen deprivation therapy.

### Implications of all the available evidence

Together with other trials in patients with metastatic castration-resistant and high-risk localised prostate cancer, we conclude that androgen receptor antagonists and abiraterone should not be combined for any disease setting. Clinically important improvements in survival and disease remission from addition of abiraterone to androgen deprivation therapy are maintained for longer than 7 years.
