# Supplementary material for: Abiraterone acetate plus prednisolone with or without enzalutamide for patients with metastatic prostate cancer starting androgen deprivation therapy: final results from two randomised phase 3 trials of the STAMPEDE platform protocol
Source: Lancet Oncol. Author manuscript; Available in PMC 2024 Nov 28. (PMC7616864; doi:10.1016/S1470-2045(23)00148-1)
Supplement: Appendix [file EMS200038-supplement-Appendix.pdf]

THE LANCET
Oncology

Supplementary appendix

This appendix formed part of the original submission and has been peer reviewed. We post it as supplied by the authors.

Supplement to: Attard G, Murphy L, Clarke NW, et al. Abiraterone acetate plus prednisolone with or without enzalutamide for patients with metastatic prostate cancer starting androgen deprivation therapy: final results from two randomised phase 3 trials of the STAMPEDE platform protocol. *Lancet Oncol* 2023; **24:** 443–56.

CONTENTS

SECTION 1

Supplementary Tables
S1. Extended characteristics at randomisation 2
S2. P values for tests of non-proportional hazards for each outcome 3
S3. Characteristics of patients planned (or not) for docetaxel after protocol amendment 4
S4. First type of event counted for calculating failure-free survival 5
S5. Reasons for permanent stopping of treatment 6
S6. Adverse events by body system 7
S7. Serious adverse reactions and suspected unexpected serious adverse reactions 14

Supplementary Figures
S1. Trials in the STAMPEDE platform protocol that recruited metastatic patients contemporaneously with or prior to the abiraterone or abiraterone and enzalutamide trials 15
S2. Weeks from randomisation to starting allocated research treatments. 16
S3. Treatment effect on survival over time 17
S4. Failure free survival split by low (A) and high (B) volume 18
S5. Progression free survival 19
S6. Skeletal-related events 20
S7. First life-prolonging treatment administered after progression. 21
S8. Time from starting to permanently stopping research treatments 22
S9. Adverse events. 23
S10. Grade 5 adverse events 24

SECTION 2

STAMPEDE protocol v21 25

Statistical analysis plan for abiraterone and enzalutamide trial including meta-analysis with abiraterone trial in M0 and M1 patients 176

List of collaborators 217

Table S1. Extended characteristics at randomisation (not included in Main Table 1).

Characteristic		Abiraterone trial		Abiraterone and enzalutamide trial	
		SOC only	SOC+Abi	SOC	SOC+Enz+Abi
		N (%)	N (%)	N (%)	N (%)
Short term bisphosphonate use	No	502 (100%)	499 (100%)	452 (100%)	462 (100%)
	Yes	0 (0%)	2 (<1%)	2 (<1%)	0 (0%)
Planned anti-androgen	No	45 (9%)	43 (9%)	33 (7%)	43 (9%)
	Short (disease flare)	456 (91%)	458 (91%)	420 (93%)	419 (91%)
	MAB	1 (<1%)	0 (0%)	1 (<1%)	0 (0%)
Hormone therapy started before randomisation	No	22 (4%)	18 (4%)	7 (2%)	3 (1%)
	Yes	480 (96%)	483 (96%)	447 (98%)	459 (99%)
Hypertension	No	315 (63%)	291 (58%)	282 (62%)	277 (60%)
	Yes, but still fit for trial	187 (37%)	209 (42%)	171 (38%)	183 (40%)
Total		502	501	454	462

20 patients across both trials did not meet all eligibility criteria.

SOC, standard-of-care, Abi, abiraterone acetate and prednisolone; Enz, enzalutamide

Table S2. P-values for tests of non-proportional hazards for each outcome

Outcome measure	Abiraterone trial	Enzalutamide and abiraterone trial
Overall Survival	0.366	0.004
Prostate cancer specific survival	0.770	0.004
Metastatic progression-free-survival	0.106	0.135
Progression-free survival	0.070	0.214
Failure-free survival	0.035	0.026
Symptomatic skeletal-related free survival	0.305	0.714

Table S3. Characteristics of patients planned (or not) for docetaxel after protocol amendment

Characteristic		No docetaxel	Docetaxel (planned)
**Age**	< 70	36 (53%)	54 (67%)
	70+	32 (47%)	26 (33%)
**N stage**	N0	28 (41%)	20 (25%)
	N+	38 (56%)	56 (70%)
	NX	2 (3%)	4 (5%)
**T stage**	T0	2 (3%)	0 (0%)
	T2	5 (7%)	5 (6%)
	T3	38 (56%)	46 (58%)
	T4	1 (21%)	23 (29%)
	Tx	9 (13%)	6 (8%)
**WHO PS**	0	49 (72%)	56 (70%)
	1-2	19 (28%)	24 (30%)
**NSAID/aspirin**	No	46 (68%)	61 (76%)
	Yes	32 (32%)	19 (24%)
**Gleason sum**	< 7	14 (21%)	7 (9%)
	8-10	53 (78%)	68 (85%)
	missing	1 (1%)	5 (6%)
**Pain from prostate cancer**	No	60 (88%)	63 (79%)
	Yes	8 (12%)	17 (21%)
**CHAARTED volume**	Low	23 (34%)	30 (38%)
	High	30 (44%)	39 (49%)
	Missing	15 (22%)	11 (14%)
**Total**		68 (100%)	80 (100%)

Table S4. First type of event counted for calculating failure-free survival, split by treatment allocation.

Characteristic	Abiraterone trial				Abiraterone and enzalutamide trial			
	SOC only		SOC+Abi		SOC		SOC+Enz+Abi	
	N (%)		N (%)		N (%)		N (%)	
Patients randomised	502		501		454		462	
**Failure-free survival event**	**455**		**301**		**377**		**237**	
**First event: (% of events)**								
Biochemical failure	384	85%	197	66%	310	83%	147	63%
Prostate cancer related death	4	1%	8	3%	6	1%	8	4%
Distant metastases (new/progression)	43	10%	71	24%	39	10%	51	22%
Lymph node involvement	3	1%	7	2%	6	2%	4	2%
Skeletal-related event	7	2%	5	2%	4	1%	11	5%
Local progression	10	2%	12	4%	10	3%	10	4%

SOC, standard-of-care, Abi, abiraterone acetate and prednisolone; Enz, enzalutamide

Table S5. Reasons for permanent stopping abiraterone in the abiraterone trial (**A**) or abiraterone and/or enzalutamide in the abiraterone and enzalutamide trial (**B**).

A

Reason for stopping abiraterone	N	%
Treatment complete	5	1%
Excessive toxicity	65	13%
Disease progression	202	41%
Death	10	2%
Other/Unknown	12	2%
Patient choice	28	6%
Clinician decision	24	5%
Intercurrent Illness	23	5%
Not stopped*	129	26%
**Total**	**498**	**100%**

B

Reason for stopping enzalutamide / Reason for stopping abiraterone	Treatment complete	Excessive toxicity	Disease progression	Death	Patient choice	Clinician decision	Intercurrent Illness	Unknown/Other*	*Not reported as stopped***	Total N	Total %
Treatment complete	2	0	0	0	0	1	0	0	0	3	1%
Excessive toxicity	0	66	20	0	4	4	1	1	24	120	27%
Disease progression	0	4	121	0	0	0	1	0	2	128	29%
Death	0	0	0	3	0	0	0	0	0	3	1%
Patient choice	0	3	1	0	25	0	0	0	0	29	7%
Clinician decision	0	1	0	0	0	12	0	0	0	13	3%
Intercurrent Illness	0	0	1	0	1	0	13	0	1	16	4%
Unknown/Other**	0	0	1	0	0	1	0	2	0	4	1%
Not stopped***	0	6	0	1	2	3	1	1	115	129	29%
Total	2	80	144	4	32	21	16	4	142	**445**	
%	<1%	18%	32%	1%	7%	5%	4%	1%	32%		

* 12 patients died before reporting stopping abiraterone

** Other reasons: One patient stopped both treatments due to returning to home country. Two patients had difficulty swallowing tablets.

*** 21 patients died before reporting stopping either treatment

.

Table S6. Adverse events by body system. Grade 4 and 5 events ≥1 in red font. Includes patients who received ≥1 dose of research treatment. Graded by National Cancer Institute Common Terminology Criteria for Adverse Events (initially, version 3.0; then STAMPEDE protocol v15 implemented 15 May 2016 updated to version 4.0).

	Standard of care in the abiraterone trial (n=502)				Standard of care in the abiraterone and enzalutamide trial (n=454)				Standard of care plus abiraterone acetate and prednisolone (n=498)				Standard of care plus abiraterone acetate and prednisolone and enzalutamide trial (n=445)			
	Grade 1–2	Grade 3	Grade 4	Grade 5	Grade 1–2	Grade 3	Grade 4	Grade 5	Grade 1–2	Grade 3	Grade 4	Grade 5	Grade 1–2	Grade 3	Grade 4	Grade 5
**Blood body system**																
Anaemia	179 (36%)	6 (1%)	1 (<1%)	0 (0%)	203 (45%)	9 (2%)	0 (0%)	0 (0%)	238 (48%)	5 (1%)	1 (<1%)	0 (0%)	221 (50%)	7 (2%)	0 (0%)	0 (0%)
Febrile neutropenia	n/a	2 (<1%)	0 (0%)	0 (0%)	n/a	12 (3%)	5 (1%)	0 (0%)	n/a	3 (1%)	0 (0%)	0 (0%)	n/a	1 (<1%)	4 (1%)	0 (0%)
Blood - other	0 (0%)	0 (0%)	0 (0%)	0 (0%)	1 (<1%)	0 (0%)	0 (0%)	0 (0%)	0 (0%)	0 (0%)	0 (0%)	0 (0%)	0 (0%)	1 (<1%)	0 (0%)	0 (0%)
**Cardiac body system**																
Acute coronary syndrome	0 (0%)	1 (<1%)	2 (<1%)	1 (<1%)	2 (<1%)	2 (<1%)	3 (1%)	0 (0%)	3 (1%)	2 (<1%)	3 (1%)	0 (0%)	3 (1%)	8 (2%)	3 (1%)	2 (<1%)
Atrial fibrilation	0 (0%)	0 (0%)	0 (0%)	0 (0%)	0 (0%)	0 (0%)	0 (0%)	0 (0%)	0 (0%)	0 (0%)	0 (0%)	0 (0%)	0 (0%)	0 (0%)	0 (0%)	0 (0%)
Chest pain	0 (0%)	0 (0%)	n/a	n/a	3 (1%)	1 (<1%)	n/a	n/a	0 (0%)	0 (0%)	n/a	n/a	0 (0%)	3 (1%)	n/a	n/a
Heart failure	1 (<1%)	0 (0%)	0 (0%)	0 (0%)	2 (<1%)	0 (0%)	0 (0%)	0 (0%)	0 (0%)	0 (0%)	1 (<1%)	0 (0%)	0 (0%)	1 (<1%)	0 (0%)	0 (0%)
Cardiac dysrhythmia	22 (4%)	0 (0%)	0 (0%)	0 (0%)	12 (3%)	2 (<1%)	0 (0%)	0 (0%)	29 (6%)	10 (2%)	3 (1%)	0 (0%)	29 (7%)	3 (1%)	0 (0%)	0 (0%)
Cardiac - other	1 (<1%)	0 (0%)	0 (0%)	0 (0%)	1 (<1%)	1 (<1%)	0 (0%)	0 (0%)	0 (0%)	0 (0%)	0 (0%)	0 (0%)	0 (0%)	2 (<1%)	1 (<1%)	3 (<1%)
**Eye, ear body system**																
Ears	0 (0%)	0 (0%)	0 (0%)	0 (0%)	2 (<1%)	1 (<1%)	0 (0%)	0 (0%)	0 (0%)	0 (0%)	0 (0%)	0 (0%)	0 (0%)	1 (<1%)	0 (0%)	0 (0%)
Blurred vision	16 (3%)	1 (<1%)	0 (0%)	0 (0%)	27 (6%)	0 (0%)	0 (0%)	0 (0%)	31 (6%)	1 (<1%)	0 (0%)	0 (0%)	26 (6%)	0 (0%)	0 (0%)	0 (0%)
Cataract	1 (<1%)	1 (<1%)	0 (0%)	0 (0%)	9 (2%)	4 (1%)	0 (0%)	0 (0%)	6 (1%)	0 (0%)	0 (0%)	0 (0%)	0 (0%)	2 (<1%)	0 (0%)	0 (0%)
(infective conjunctivitis)	2 (<1%)	0 (0%)	0 (0%)	0 (0%)	1 (<1%)	0 (0%)	0 (0%)	0 (0%)	4 (1%)	1 (<1%)	0 (0%)	0 (0%)	5 (1%)	0 (0%)	0 (0%)	0 (0%)
Eye disorders - other	20 (4%)	0 (0%)	0 (0%)	0 (0%)	28 (6%)	1 (<1%)	0 (0%)	0 (0%)	40 (8%)	6 (1%)	0 (0%)	0 (0%)	47 (11%)	4 (1%)	0 (0%)	0 (0%)

	Standard of care in the abiraterone trial (n=502)				Standard of care in the abiraterone and enzalutamide trial (n=454)				Standard of care plus abiraterone acetate and prednisolone (n=498)				Standard of care plus abiraterone acetate and prednisolone and enzalutamide trial (n=445)			
	Grade 1–2	Grade 3	Grade 4	Grade 5	Grade 1–2	Grade 3	Grade 4	Grade 5	Grade 1–2	Grade 3	Grade 4	Grade 5	Grade 1–2	Grade 3	Grade 4	Grade 5
**GI body system**																
Abdominal pain	50 (10%)	7 (1%)	0 (0%)	0 (0%)	66 (15%)	2 (<1%)	0 (0%)	0 (0%)	96 (19%)	8 (2%)	0 (0%)	0 (0%)	69 (16%)	4 (1%)	0 (0%)	0 (0%)
Constipation	106 (21%)	5 (1%)	0 (0%)	0 (0%)	136 (30%)	1 (<1%)	0 (0%)	0 (0%)	145 (29%)	4 (1%)	0 (0%)	0 (0%)	166 (37%)	6 (1%)	0 (0%)	0 (0%)
Diarrhoea	75 (15%)	3 (1%)	0 (0%)	0 (0%)	90 (20%)	3 (1%)	0 (0%)	0 (0%)	114 (23%)	9 (2%)	0 (0%)	0 (0%)	130 (29%)	10 (2%)	0 (0%)	0 (0%)
Lower GI haemorrhage	1 (<1%)	0 (0%)	0 (0%)	0 (0%)	3 (1%)	0 (0%)	0 (0%)	0 (0%)	6 (1%)	1 (<1%)	0 (0%)	0 (0%)	9 (2%)	1 (<1%)	0 (0%)	0 (0%)
Nausea	51 (10%)	0 (0%)	n/a	n/a	66 (15%)	3 (1%)	n/a	n/a	96 (19%)	1 (<1%)	n/a	n/a	119 (27%)	3 (1%)	n/a	n/a
Upper GI haemorrhage	0 (0%)	0 (0%)	0 (0%)	0 (0%)	0 (0%)	0 (0%)	0 (0%)	0 (0%)	0 (0%)	0 (0%)	0 (0%)	0 (0%)	0 (0%)	1 (<1%)	0 (0%)	0 (0%)
Vomiting	25 (5%)	0 (0%)	1 (<1%)	0 (0%)	32 (7%)	3 (1%)	0 (0%)	0 (0%)	49 (10%)	4 (1%)	0 (0%)	0 (0%)	51 (11%)	5 (1%)	0 (0%)	0 (0%)
Flatulence	51 (10%)	0 (0%)	0 (0%)	0 (0%)	57 (13%)	0 (0%)	0 (0%)	0 (0%)	73 (15%)	0 (0%)	0 (0%)	0 (0%)	0 (0%)	0 (0%)	0 (0%)	0 (0%)
Dyspepsia	52 (10%)	0 (0%)	0 (0%)	0 (0%)	53 (12%)	0 (0%)	0 (0%)	0 (0%)	98 (20%)	0 (0%)	0 (0%)	0 (0%)	0 (0%)	2 (<1%)	0 (0%)	0 (0%)
GI haemorrhage	14 (3%)	2 (<1%)	0 (0%)	0 (0%)	8 (2%)	1 (<1%)	0 (0%)	0 (0%)	16 (3%)	0 (0%)	0 (0%)	0 (0%)	0 (0%)	2 (<1%)	0 (0%)	0 (0%)
GI - other	50 (10%)	9 (2%)	2 (<1%)	0 (0%)	53 (12%)	8 (2%)	4 (1%)	1 (<1%)	98 (20%)	15 (3%)	3 (1%)	1 (<1%)	92 (21%)	4 (1%)	6 (1%)	0 (0%)
**General body system**																
Fatigue	261 (52%)	11 (2%)	n/a	n/a	296 (65%)	13 (3%)	n/a	n/a	329 (66%)	13 (3%)	n/a	n/a	348 (78%)	34 (8%)	n/a	n/a
Fever	15 (3%)	0 (0%)	0 (0%)	0 (0%)	29 (6%)	1 (<1%)	0 (0%)	0 (0%)	31 (6%)	1 (<1%)	0 (0%)	0 (0%)	26 (6%)	5 (1%)	0 (0%)	0 (0%)
Irritability	0 (0%)	0 (0%)	n/a	n/a	11 (2%)	0 (0%)	n/a	n/a	0 (0%)	0 (0%)	n/a	n/a	0 (0%)	0 (0%)	n/a	n/a
Localised (o)dema	76 (15%)	1 (<1%)	n/a	n/a	97 (21%)	2 (<1%)	n/a	n/a	118 (24%)	2 (<1%)	n/a	n/a	116 (26%)	4 (1%)	n/a	n/a
Multi-organ failure	0 (0%)	0 (0%)	0 (0%)	0 (0%)	0 (0%)	0 (0%)	0 (0%)	0 (0%)	0 (0%)	0 (0%)	0 (0%)	0 (0%)	0 (0%)	0 (0%)	0 (0%)	0 (0%)
Non-cardiac chest pain	0 (0%)	0 (0%)	0 (0%)	0 (0%)	0 (0%)	1 (<1%)	0 (0%)	0 (0%)	0 (0%)	0 (0%)	0 (0%)	0 (0%)	0 (0%)	1 (<1%)	0 (0%)	0 (0%)
Fall	6 (1%)	1 (<1%)	0 (0%)	0 (0%)	15 (3%)	4 (1%)	0 (0%)	0 (0%)	15 (3%)	4 (1%)	0 (0%)	0 (0%)	37 (8%)	6 (1%)	0 (0%)	0 (0%)
Flu-like symptoms	30 (6%)	0 (0%)	0 (0%)	0 (0%)	22 (5%)	0 (0%)	0 (0%)	0 (0%)	59 (12%)	1 (<1%)	0 (0%)	0 (0%)	34 (8%)	0 (0%)	0 (0%)	0 (0%)
Oral candidiasis	1 (<1%)	0 (0%)	0 (0%)	0 (0%)	4 (1%)	0 (0%)	0 (0%)	0 (0%)	8 (2%)	1 (<1%)	0 (0%)	0 (0%)	8 (2%)	0 (0%)	0 (0%)	0 (0%)
Generalised pain	151 (30%)	3 (1%)	0 (0%)	0 (0%)	126 (28%)	5 (1%)	0 (0%)	0 (0%)	168 (34%)	7 (1%)	0 (0%)	0 (0%)	106 (24%)	7 (2%)	0 (0%)	0 (0%)
General and admin - other	41 (8%)	3 (1%)	3 (1%)	0 (0%)	32 (7%)	7 (2%)	0 (0%)	0 (0%)	70 (14%)	8 (2%)	3 (1%)	2 (<1%)	70 (16%)	3 (1%)	2 (<1%)	2 (<1%)

	Standard of care in the abiraterone trial (n=502)				Standard of care in the abiraterone and enzalutamide trial (n=454)				Standard of care plus abiraterone acetate and prednisolone (n=498)				Standard of care plus abiraterone acetate and prednisolone and enzalutamide trial (n=445)			
	Grade 1–2	Grade 3	Grade 4	Grade 5	Grade 1–2	Grade 3	Grade 4	Grade 5	Grade 1–2	Grade 3	Grade 4	Grade 5	Grade 1–2	Grade 3	Grade 4	Grade 5
**Hepatobiliary disorders**																
Cholecystitis	0 (0%)	0 (0%)	0 (0%)	0 (0%)	0 (0%)	1 (<1%)	0 (0%)	0 (0%)	0 (0%)	0 (0%)	0 (0%)	0 (0%)	0 (0%)	0 (0%)	0 (0%)	0 (0%)
Abnormal hepatic function	28 (6%)	1 (<1%)	0 (0%)	0 (0%)	13 (3%)	1 (<1%)	0 (0%)	0 (0%)	54 (11%)	8 (2%)	1 (<1%)	0 (0%)	24 (5%)	3 (1%)	0 (0%)	0 (0%)
Hepatobiliary - other	15 (3%)	1 (<1%)	0 (0%)	0 (0%)	2 (<1%)	1 (<1%)	0 (0%)	0 (0%)	21 (4%)	0 (0%)	0 (0%)	0 (0%)	0 (0%)	0 (0%)	0 (0%)	0 (0%)
**Immune body system**																
Allergic reaction	18 (4%)	1 (<1%)	0 (0%)	0 (0%)	11 (2%)	0 (0%)	0 (0%)	0 (0%)	19 (4%)	2 (<1%)	0 (0%)	0 (0%)	11 (2%)	2 (<1%)	0 (0%)	0 (0%)
Immune - other	0 (0%)	0 (0%)	0 (0%)	0 (0%)	0 (0%)	0 (0%)	0 (0%)	0 (0%)	0 (0%)	0 (0%)	0 (0%)	0 (0%)	0 (0%)	0 (0%)	0 (0%)	0 (0%)
**Infections body system**																
Abdominal infection	0 (0%)	0 (0%)	0 (0%)	0 (0%)	0 (0%)	0 (0%)	0 (0%)	0 (0%)	0 (0%)	0 (0%)	0 (0%)	0 (0%)	0 (0%)	1 (<1%)	0 (0%)	0 (0%)
Lung infection	0 (0%)	0 (0%)	0 (0%)	0 (0%)	1 (<1%)	1 (<1%)	0 (0%)	1 (<1%)	0 (0%)	0 (0%)	0 (0%)	0 (0%)	0 (0%)	4 (1%)	1 (<1%)	1 (<1%)
Sepsis	0 (0%)	0 (0%)	0 (0%)	0 (0%)	0 (0%)	0 (0%)	0 (0%)	0 (0%)	0 (0%)	0 (0%)	0 (0%)	0 (0%)	0 (0%)	0 (0%)	0 (0%)	0 (0%)
Skin infection	0 (0%)	0 (0%)	0 (0%)	0 (0%)	0 (0%)	0 (0%)	0 (0%)	0 (0%)	0 (0%)	0 (0%)	0 (0%)	0 (0%)	0 (0%)	1 (<1%)	0 (0%)	0 (0%)
Urinary tract infection	34 (7%)	4 (1%)	0 (0%)	0 (0%)	33 (7%)	7 (2%)	0 (0%)	0 (0%)	33 (7%)	6 (1%)	0 (0%)	0 (0%)	35 (8%)	10 (2%)	1 (<1%)	1 (<1%)
Sinusitis	9 (2%)	0 (0%)	0 (0%)	0 (0%)	5 (1%)	0 (0%)	0 (0%)	0 (0%)	16 (3%)	1 (<1%)	0 (0%)	0 (0%)	15 (3%)	0 (0%)	0 (0%)	0 (0%)
Upper respiratory infection	37 (7%)	1 (<1%)	0 (0%)	0 (0%)	47 (10%)	3 (1%)	0 (0%)	0 (0%)	85 (17%)	2 (<1%)	0 (0%)	0 (0%)	64 (14%)	4 (1%)	0 (0%)	0 (0%)
Lower respiratory infection	3 (1%)	0 (0%)	0 (0%)	0 (0%)	9 (2%)	6 (1%)	1 (<1%)	0 (0%)	10 (2%)	9 (2%)	1 (<1%)	0 (0%)	8 (2%)	5 (1%)	0 (0%)	0 (0%)
Infections - other	0 (0%)	0 (0%)	0 (0%)	0 (0%)	8 (2%)	3 (1%)	0 (0%)	0 (0%)	2 (<1%)	0 (0%)	0 (0%)	1 (<1%)	0 (0%)	6 (1%)	1 (<1%)	1 (<1%)
**Injury, poisoning and procedural complications**																
Fracture	2 (<1%)	0 (0%)	0 (0%)	0 (0%)	5 (1%)	6 (1%)	0 (0%)	0 (0%)	6 (1%)	3 (1%)	0 (0%)	0 (0%)	17 (4%)	9 (2%)	0 (0%)	0 (0%)
Injury other	2 (<1%)	1 (<1%)	0 (0%)	0 (0%)	3 (1%)	0 (0%)	0 (0%)	0 (0%)	0 (0%)	0 (0%)	0 (0%)	0 (0%)	0 (0%)	2 (<1%)	0 (0%)	0 (0%)

	Standard of care in the abiraterone trial (n=502)				Standard of care in the abiraterone and enzalutamide trial (n=454)				Standard of care plus abiraterone acetate and prednisolone (n=498)				Standard of care plus abiraterone acetate and prednisolone and enzalutamide trial (n=445)			
	Grade 1–2	Grade 3	Grade 4	Grade 5	Grade 1–2	Grade 3	Grade 4	Grade 5	Grade 1–2	Grade 3	Grade 4	Grade 5	Grade 1–2	Grade 3	Grade 4	Grade 5
**Investigations**																
ALT increased	65 (13%)	4 (1%)	0 (0%)	n/a	75 (17%)	3 (1%)	0 (0%)	n/a	107 (21%)	26 (5%)	1 (<1%)	n/a	112 (25%)	44 (10%)	1 (<1%)	n/a
AST increased	13 (3%)	2 (<1%)	0 (0%)	n/a	17 (4%)	0 (0%)	0 (0%)	n/a	28 (6%)	8 (2%)	1 (<1%)	n/a	43 (10%)	9 (2%)	0 (0%)	n/a
Cholesterol high	6 (1%)	0 (0%)	0 (0%)	n/a	11 (2%)	0 (0%)	0 (0%)	n/a	7 (1%)	0 (0%)	0 (0%)	n/a	0 (0%)	0 (0%)	0 (0%)	n/a
Creatine increase	0 (0%)	0 (0%)	0 (0%)	n/a	6 (1%)	0 (0%)	0 (0%)	n/a	4 (1%)	0 (0%)	0 (0%)	n/a	0 (0%)	0 (0%)	0 (0%)	n/a
Blood bilirubin increased	2 (<1%)	1 (<1%)	0 (0%)	0 (0%)	7 (2%)	0 (0%)	0 (0%)	0 (0%)	10 (2%)	4 (1%)	0 (0%)	0 (0%)	22 (5%)	2 (<1%)	0 (0%)	0 (0%)
Platelet count decreased	18 (4%)	1 (<1%)	0 (0%)	0 (0%)	31 (7%)	1 (<1%)	1 (<1%)	0 (0%)	24 (5%)	1 (<1%)	0 (0%)	0 (0%)	24 (5%)	0 (0%)	1 (<1%)	0 (0%)
Neutrophil count decreased	23 (5%)	1 (<1%)	1 (<1%)	0 (0%)	26 (6%)	1 (<1%)	8 (2%)	0 (0%)	41 (8%)	6 (1%)	2 (<1%)	0 (0%)	35 (8%)	4 (1%)	4 (1%)	0 (0%)
Alkaline phosphatase increased	15 (3%)	1 (<1%)	0 (0%)	0 (0%)	52 (11%)	6 (1%)	0 (0%)	0 (0%)	26 (5%)	1 (<1%)	0 (0%)	0 (0%)	48 (11%)	7 (2%)	0 (0%)	0 (0%)
Weight gain	11 (2%)	0 (0%)	0 (0%)	0 (0%)	47 (10%)	1 (<1%)	0 (0%)	0 (0%)	24 (5%)	3 (1%)	0 (0%)	0 (0%)	46 (10%)	5 (1%)	0 (0%)	0 (0%)
Investigations - other	171 (34%)	13 (3%)	1 (<1%)	0 (0%)	141 (31%)	16 (4%)	2 (<1%)	0 (0%)	235 (47%)	11 (2%)	2 (<1%)	0 (0%)	166 (37%)	9 (2%)	4 (1%)	0 (0%)
**Metabolism and nutrition body system**																
Dehydration	0 (0%)	0 (0%)	0 (0%)	0 (0%)	0 (0%)	0 (0%)	0 (0%)	0 (0%)	0 (0%)	0 (0%)	0 (0%)	0 (0%)	0 (0%)	0 (0%)	0 (0%)	0 (0%)
Glucose intolerance	19 (4%)	3 (1%)	0 (0%)	0 (0%)	31 (7%)	1 (<1%)	0 (0%)	0 (0%)	54 (11%)	4 (1%)	0 (0%)	0 (0%)	23 (5%)	7 (2%)	0 (0%)	0 (0%)
Hypokalemia	16 (3%)	0 (0%)	1 (<1%)	0 (0%)	21 (5%)	0 (0%)	0 (0%)	0 (0%)	57 (11%)	7 (1%)	0 (0%)	0 (0%)	66 (15%)	7 (2%)	2 (<1%)	0 (0%)
Anorexia	39 (8%)	2 (<1%)	0 (0%)	0 (0%)	57 (13%)	2 (<1%)	0 (0%)	0 (0%)	57 (11%)	1 (<1%)	0 (0%)	0 (0%)	79 (18%)	3 (1%)	0 (0%)	0 (0%)
Hypoalbuminaemia	6 (1%)	0 (0%)	0 (0%)	0 (0%)	22 (5%)	0 (0%)	0 (0%)	0 (0%)	13 (3%)	0 (0%)	0 (0%)	0 (0%)	0 (0%)	0 (0%)	0 (0%)	0 (0%)
Hypercalcaemia	6 (1%)	0 (0%)	0 (0%)	0 (0%)	22 (5%)	0 (0%)	0 (0%)	0 (0%)	13 (3%)	0 (0%)	0 (0%)	0 (0%)	0 (0%)	0 (0%)	0 (0%)	0 (0%)
Hypertriglyceridaemia	2 (<1%)	0 (0%)	0 (0%)	0 (0%)	6 (1%)	0 (0%)	0 (0%)	0 (0%)	4 (1%)	0 (0%)	0 (0%)	0 (0%)	0 (0%)	2 (<1%)	0 (0%)	0 (0%)
Hyperphosphataemia	2 (<1%)	0 (0%)	0 (0%)	0 (0%)	6 (1%)	0 (0%)	0 (0%)	0 (0%)	4 (1%)	0 (0%)	0 (0%)	0 (0%)	0 (0%)	2 (<1%)	0 (0%)	0 (0%)
Metabolism and nutrition - other	49 (10%)	0 (0%)	0 (0%)	0 (0%)	36 (8%)	2 (<1%)	0 (0%)	0 (0%)	55 (11%)	2 (<1%)	0 (0%)	0 (0%)	63 (14%)	0 (0%)	2 (<1%)	0 (0%)

	Standard of care in the abiraterone trial (n=502)				Standard of care in the abiraterone and enzalutamide trial (n=454)				Standard of care plus abiraterone acetate and prednisolone (n=498)				Standard of care plus abiraterone acetate and prednisolone and enzalutamide trial (n=445)			
	Grade 1–2	Grade 3	Grade 4	Grade 5	Grade 1–2	Grade 3	Grade 4	Grade 5	Grade 1–2	Grade 3	Grade 4	Grade 5	Grade 1–2	Grade 3	Grade 4	Grade 5
**Musculoskeletal body system**																
Bone pain	194 (39%)	23 (5%)	0 (0%)	0 (0%)	189 (42%)	21 (5%)	0 (0%)	0 (0%)	197 (40%)	22 (4%)	0 (0%)	0 (0%)	175 (39%)	23 (5%)	0 (0%)	0 (0%)
Generalised muscle weakness	0 (0%)	0 (0%)	0 (0%)	0 (0%)	0 (0%)	0 (0%)	0 (0%)	0 (0%)	0 (0%)	0 (0%)	0 (0%)	0 (0%)	0 (0%)	0 (0%)	0 (0%)	0 (0%)
Osteoporosis	0 (0%)	0 (0%)	n/a	n/a	12 (3%)	0 (0%)	n/a	n/a	8 (2%)	0 (0%)	n/a	n/a	0 (0%)	1 (<1%)	n/a	n/a
Back pain	37 (7%)	0 (0%)	0 (0%)	0 (0%)	96 (21%)	14 (3%)	0 (0%)	0 (0%)	59 (12%)	7 (1%)	0 (0%)	0 (0%)	120 (27%)	15 (3%)	0 (0%)	0 (0%)
Arthralgia	110 (22%)	6 (1%)	n/a	n/a	141 (31%)	9 (2%)	n/a	n/a	144 (29%)	9 (2%)	n/a	n/a	172 (39%)	5 (1%)	n/a	n/a
Myalgia	73 (15%)	0 (0%)	n/a	n/a	80 (18%)	2 (<1%)	n/a	n/a	108 (22%)	0 (0%)	n/a	n/a	0 (0%)	1 (<1%)	n/a	n/a
Musculoskeletal and connective tissue - other	186 (37%)	10 (2%)	2 (<1%)	0 (0%)	140 (31%)	12 (3%)	0 (0%)	0 (0%)	221 (44%)	30 (6%)	0 (0%)	0 (0%)	164 (37%)	22 (5%)	0 (0%)	0 (0%)
**Neoplasms**																
Neoplasms - other	2 (<1%)	0 (0%)	0 (0%)	0 (0%)	1 (<1%)	2 (<1%)	0 (0%)	1 (<1%)	0 (0%)	0 (0%)	0 (0%)	0 (0%)	0 (0%)	1 (<1%)	1 (<1%)	1 (<1%)
**Nervous body system**																
Concentration impairment	1 (<1%)	0 (0%)	n/a	n/a	10 (2%)	1 (<1%)	n/a	n/a	0 (0%)	0 (0%)	n/a	n/a	0 (0%)	0 (0%)	n/a	n/a
Dizziness	50 (10%)	0 (0%)	n/a	n/a	57 (13%)	0 (0%)	n/a	n/a	91 (18%)	1 (<1%)	n/a	n/a	97 (22%)	3 (1%)	n/a	n/a
Seizure	1 (<1%)	0 (0%)	0 (0%)	0 (0%)	1 (<1%)	0 (0%)	0 (0%)	0 (0%)	2 (<1%)	0 (0%)	0 (0%)	0 (0%)	0 (0%)	4 (1%)	0 (0%)	0 (0%)
Stroke	0 (0%)	0 (0%)	0 (0%)	0 (0%)	1 (<1%)	1 (<1%)	0 (0%)	0 (0%)	0 (0%)	0 (0%)	0 (0%)	0 (0%)	0 (0%)	1 (<1%)	2 (<1%)	0 (0%)
TIA	0 (0%)	0 (0%)	0 (0%)	0 (0%)	0 (0%)	0 (0%)	0 (0%)	0 (0%)	0 (0%)	0 (0%)	0 (0%)	0 (0%)	0 (0%)	0 (0%)	0 (0%)	0 (0%)
Cognitive disturbance	18 (4%)	1 (<1%)	0 (0%)	0 (0%)	22 (5%)	2 (<1%)	0 (0%)	0 (0%)	33 (7%)	3 (1%)	0 (0%)	0 (0%)	83 (19%)	2 (<1%)	1 (<1%)	0 (0%)
Headache	52 (10%)	1 (<1%)	0 (0%)	0 (0%)	53 (12%)	2 (<1%)	0 (0%)	0 (0%)	90 (18%)	7 (1%)	0 (0%)	0 (0%)	110 (25%)	5 (1%)	0 (0%)	0 (0%)
Nervous system - other	42 (8%)	7 (1%)	6 (1%)	1 (<1%)	69 (15%)	11 (2%)	1 (<1%)	0 (0%)	75 (15%)	12 (2%)	3 (1%)	2 (<1%)	110 (25%)	9 (2%)	3 (1%)	0 (0%)
**Psychiatric body system**																
Anxiety	15 (3%)	0 (0%)	1 (<1%)	0 (0%)	50 (11%)	2 (<1%)	0 (0%)	0 (0%)	40 (8%)	0 (0%)	0 (0%)	0 (0%)	0 (0%)	0 (0%)	1 (<1%)	0 (0%)
Depression	14 (3%)	0 (0%)	1 (<1%)	0 (0%)	58 (13%)	0 (0%)	0 (0%)	0 (0%)	40 (8%)	1 (<1%)	0 (0%)	0 (0%)	82 (18%)	0 (0%)	1 (<1%)	0 (0%)
Insomnia	101 (20%)	5 (1%)	n/a	n/a	144 (32%)	3 (1%)	n/a	n/a	149 (30%)	6 (1%)	n/a	n/a	149 (33%)	0 (0%)	n/a	n/a
Libido decreased	1 (<1%)	n/a	n/a	n/a	24 (5%)	n/a	n/a	n/a	2 (<1%)	n/a	n/a	n/a	0 (0%)	n/a	n/a	n/a

	Standard of care in the abiraterone trial (n=502)				Standard of care in the abiraterone and enzalutamide trial (n=454)				Standard of care plus abiraterone acetate and prednisolone (n=498)				Standard of care plus abiraterone acetate and prednisolone and enzalutamide trial (n=445)			
	Grade 1–2	Grade 3	Grade 4	Grade 5	Grade 1–2	Grade 3	Grade 4	Grade 5	Grade 1–2	Grade 3	Grade 4	Grade 5	Grade 1–2	Grade 3	Grade 4	Grade 5
Psychiatric - other	70 (14%)	3 (1%)	1 (<1%)	0 (0%)	58 (13%)	1 (<1%)	0 (0%)	0 (0%)	112 (22%)	4 (1%)	0 (0%)	0 (0%)	83 (19%)	3 (1%)	0 (0%)	0 (0%)
**Renal and urinary disorders**																
Acute kidney injury	7 (1%)	3 (1%)	1 (<1%)	0 (0%)	22 (5%)	1 (<1%)	1 (<1%)	0 (0%)	10 (2%)	1 (<1%)	1 (<1%)	0 (0%)	19 (4%)	2 (<1%)	1 (<1%)	2 (<1%)
Haematuria	26 (5%)	2 (<1%)	0 (0%)	0 (0%)	17 (4%)	3 (1%)	0 (0%)	0 (0%)	34 (7%)	2 (<1%)	0 (0%)	0 (0%)	19 (4%)	3 (1%)	0 (0%)	0 (0%)
Renal calculi	0 (0%)	0 (0%)	0 (0%)	0 (0%)	0 (0%)	0 (0%)	0 (0%)	0 (0%)	0 (0%)	0 (0%)	0 (0%)	0 (0%)	0 (0%)	1 (<1%)	0 (0%)	0 (0%)
Urinary frequency	272 (54%)	n/a	n/a	n/a	271 (60%)	n/a	n/a	n/a	301 (60%)	n/a	n/a	n/a	0 (0%)	n/a	n/a	n/a
Urinary tract obstruction	3 (1%)	2 (<1%)	0 (0%)	0 (0%)	10 (2%)	3 (1%)	1 (<1%)	0 (0%)	8 (2%)	2 (<1%)	0 (0%)	0 (0%)	9 (2%)	2 (<1%)	2 (<1%)	0 (0%)
Chronic kidney disease	47 (9%)	3 (1%)	0 (0%)	0 (0%)	42 (9%)	4 (1%)	1 (<1%)	0 (0%)	41 (8%)	2 (<1%)	0 (0%)	0 (0%)	37 (8%)	3 (1%)	0 (0%)	0 (0%)
Urinary urgency	30 (6%)	0 (0%)	0 (0%)	0 (0%)	86 (19%)	0 (0%)	0 (0%)	0 (0%)	39 (8%)	0 (0%)	0 (0%)	0 (0%)	0 (0%)	0 (0%)	0 (0%)	0 (0%)
Renal and urinary - other	79 (16%)	12 (2%)	0 (0%)	0 (0%)	66 (15%)	14 (3%)	2 (<1%)	0 (0%)	73 (15%)	16 (3%)	0 (0%)	0 (0%)	77 (17%)	17 (4%)	2 (<1%)	0 (0%)
**Reproductive system**																
Erectile dysfunction	165 (33%)	59 (12%)	0 (0%)	0 (0%)	161 (35%)	56 (12%)	0 (0%)	0 (0%)	201 (40%)	63 (13%)	0 (0%)	0 (0%)	177 (40%)	76 (17%)	0 (0%)	0 (0%)
Gynecomastia	51 (10%)	1 (<1%)	n/a	n/a	87 (19%)	2 (<1%)	n/a	n/a	69 (14%)	0 (0%)	n/a	n/a	0 (0%)	3 (1%)	n/a	n/a
Reproductive - other	0 (0%)	0 (0%)	0 (0%)	0 (0%)	3 (1%)	0 (0%)	0 (0%)	0 (0%)	0 (0%)	0 (0%)	0 (0%)	0 (0%)	0 (0%)	0 (0%)	0 (0%)	0 (0%)
**Respiratory thoracic and mediastinal**																
Cough	63 (13%)	0 (0%)	0 (0%)	0 (0%)	101 (22%)	2 (<1%)	0 (0%)	0 (0%)	157 (32%)	1 (<1%)	0 (0%)	0 (0%)	95 (21%)	0 (0%)	0 (0%)	0 (0%)
Breathlessness	84 (17%)	5 (1%)	0 (0%)	0 (0%)	113 (25%)	6 (1%)	2 (<1%)	0 (0%)	147 (30%)	7 (1%)	2 (<1%)	0 (0%)	136 (31%)	4 (1%)	0 (0%)	0 (0%)
Wheezing	0 (0%)	0 (0%)	0 (0%)	0 (0%)	0 (0%)	0 (0%)	0 (0%)	0 (0%)	0 (0%)	0 (0%)	0 (0%)	0 (0%)	0 (0%)	0 (0%)	0 (0%)	0 (0%)
Allergic rhinitis	17 (3%)	0 (0%)	0 (0%)	0 (0%)	20 (4%)	0 (0%)	0 (0%)	0 (0%)	40 (8%)	0 (0%)	0 (0%)	0 (0%)	0 (0%)	0 (0%)	0 (0%)	0 (0%)
Respiratory - other	20 (4%)	7 (1%)	0 (0%)	1 (<1%)	22 (5%)	5 (1%)	1 (<1%)	0 (0%)	73 (15%)	7 (1%)	1 (<1%)	4 (1%)	54 (12%)	9 (2%)	1 (<1%)	1 (<1%)

	Standard of care in the abiraterone trial (n=502)				Standard of care in the abiraterone and enzalutamide trial (n=454)				Standard of care plus abiraterone acetate and prednisolone (n=498)				Standard of care plus abiraterone acetate and prednisolone and enzalutamide trial (n=445)			
	Grade 1–2	Grade 3	Grade 4	Grade 5	Grade 1–2	Grade 3	Grade 4	Grade 5	Grade 1–2	Grade 3	Grade 4	Grade 5	Grade 1–2	Grade 3	Grade 4	Grade 5
**Skin and subcutaneous tissue**																
Alopecia	3 (1%)	n/a	n/a	n/a	9 (2%)	n/a	n/a	n/a	3 (1%)	n/a	n/a	n/a	0 (0%)	n/a	n/a	n/a
Pruritus	0 (0%)	0 (0%)	n/a	n/a	4 (1%)	0 (0%)	n/a	n/a	0 (0%)	0 (0%)	n/a	n/a	0 (0%)	0 (0%)	n/a	n/a
Nail loss/dicolouration	25 (5%)	0 (0%)	0 (0%)	0 (0%)	36 (8%)	0 (0%)	0 (0%)	0 (0%)	33 (7%)	0 (0%)	0 (0%)	0 (0%)	0 (0%)	0 (0%)	0 (0%)	0 (0%)
Rash maculo-papular	39 (8%)	0 (0%)	0 (0%)	0 (0%)	47 (10%)	0 (0%)	0 (0%)	0 (0%)	74 (15%)	2 (<1%)	0 (0%)	0 (0%)	49 (11%)	0 (0%)	0 (0%)	0 (0%)
Skin hypopigmentation	0 (0%)	0 (0%)	0 (0%)	0 (0%)	2 (<1%)	0 (0%)	0 (0%)	0 (0%)	0 (0%)	0 (0%)	0 (0%)	0 (0%)	0 (0%)	0 (0%)	0 (0%)	0 (0%)
Skin - other	72 (14%)	5 (1%)	0 (0%)	0 (0%)	67 (15%)	4 (1%)	1 (<1%)	0 (0%)	127 (26%)	11 (2%)	1 (<1%)	0 (0%)	114 (26%)	3 (1%)	1 (<1%)	0 (0%)
**Social body system**																
Social - other	1 (<1%)	0 (0%)	0 (0%)	0 (0%)	1 (<1%)	0 (0%)	0 (0%)	0 (0%)	0 (0%)	0 (0%)	0 (0%)	0 (0%)	0 (0%)	0 (0%)	0 (0%)	0 (0%)
**Surgical body system**																
Surgical - other	0 (0%)	0 (0%)	0 (0%)	0 (0%)	0 (0%)	1 (<1%)	1 (<1%)	0 (0%)	0 (0%)	0 (0%)	0 (0%)	0 (0%)	0 (0%)	0 (0%)	0 (0%)	0 (0%)
**Vascular body system**																
Hypertension	58 (12%)	6 (1%)	0 (0%)	0 (0%)	60 (13%)	12 (3%)	0 (0%)	0 (0%)	161 (32%)	26 (5%)	0 (0%)	0 (0%)	187 (42%)	69 (16%)	0 (0%)	0 (0%)
Thromboembolic event	2 (<1%)	1 (<1%)	0 (0%)	0 (0%)	3 (1%)	0 (0%)	1 (<1%)	0 (0%)	0 (0%)	0 (0%)	0 (0%)	0 (0%)	0 (0%)	0 (0%)	1 (<1%)	0 (0%)
Hot flashes	384 (76%)	20 (4%)	n/a	n/a	365 (80%)	12 (3%)	n/a	n/a	406 (82%)	25 (5%)	n/a	n/a	374 (84%)	24 (5%)	n/a	n/a
Hypotension	7 (1%)	1 (<1%)	0 (0%)	0 (0%)	8 (2%)	1 (<1%)	0 (0%)	0 (0%)	23 (5%)	2 (<1%)	0 (0%)	0 (0%)	16 (4%)	3 (1%)	0 (0%)	0 (0%)
**Cardiovascular***																
Cardiovascular - other	23 (5%)	7 (1%)	1 (<1%)	0 (0%)	12 (3%)	6 (1%)	2 (<1%)	0 (0%)	37 (7%)	12 (2%)	3 (1%)	0 (0%)	19 (4%)	8 (2%)	4 (1%)	2 (<1%)

* events reported on Toxicity and SAE CRFs are now categorised by body system according to CTCAE v4.03, which includes separate 'cardiac disorder' and 'vascular disorder' categories. Prior to Nov-2018, toxicities could only be categorised into a single 'cardiovascular disorders' body system. Events that were previously categorised as 'cardiovascular' and attributed to specific toxicities (e.g. heart failure) have been reassigned to the relevant body system under CTCAE v4.03. However, it has not been possible to reassign toxicities that were previously classified as 'cardiovascular – other'. These are presented within the old 'cardiovascular' body system.

Table S7. Serious adverse reactions (SAR) and suspected unexpected serious adverse reactions (SUSAR). Cardiac were the most common SAR/SUSAR type: included in further break-down of attribution.

	SOC, Abi trial	SOC, Enz + Abi trial	SOC + Abi	SOC + Abi +Enz
**Total SAR/SUSAR**	5	5	27	16
**Cardiac SAR/SUSAR**	2	1	12	6
**Fatal SUSAR**	0	0	0	2*

*Both fatal SUSARS were attributed to a cardiac cause.

SOC, standard-of-care, Abi, abiraterone acetate and prednisolone; Enz, enzalutamide

Figure S1. Trials in the STAMPEDE platform protocol that recruited metastatic patients contemporaneously with or prior to the abiraterone or abiraterone and enzalutamide trials.

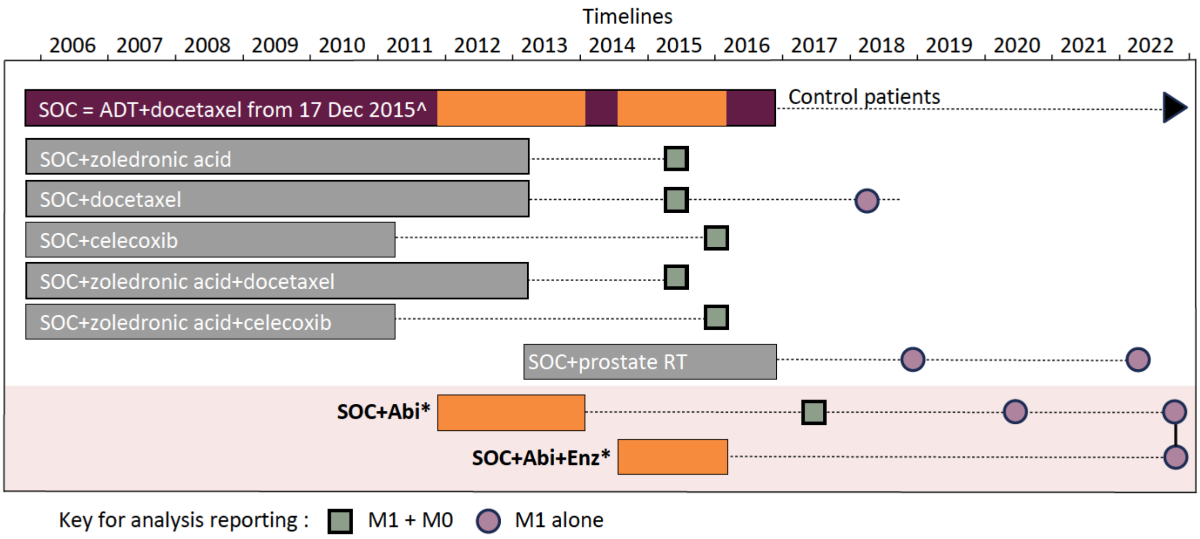

Period of accrual denoted by solid bars. Analysis timelines denoted by dotted lines and analysis reporting by symbols in key. SOC, standard of care; ADT, androgen deprivation therapy; Abi, abiraterone acetate and prednisolone; Enz, enzalutamide; M0, non-metastatic; M1 metastatic; ^when indicated; *meta-analysed trials

Figure S2. Weeks from randomisation to starting allocated research treatments.

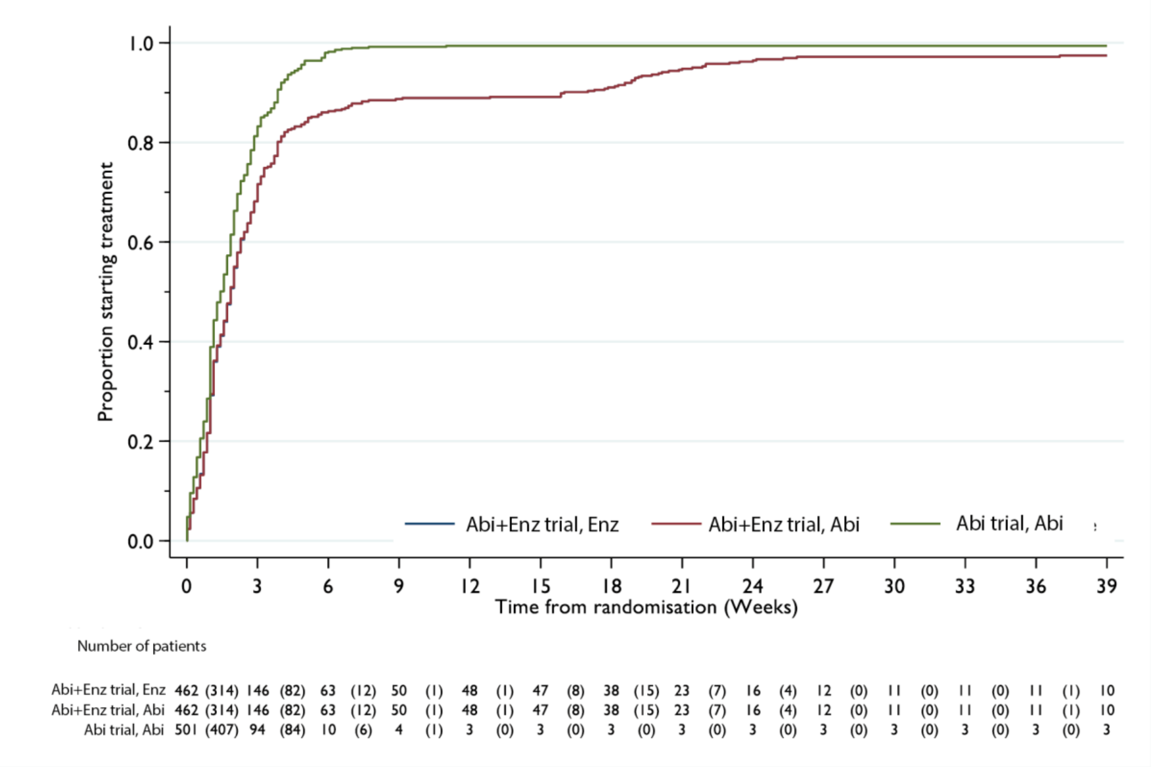

Note: Enzalutamide (blue) line is under abiraterone (red line) given in abi+enz trial. Abi, abiraterone acetate and prednisolone; enz, enzalutamide

Figure S3. Treatment effect on survival over time. Flexible parametric models used to calculate changes in treatment effect over time in the abiraterone trial (A, B) and abiraterone and enzalutamide trial (C, D).

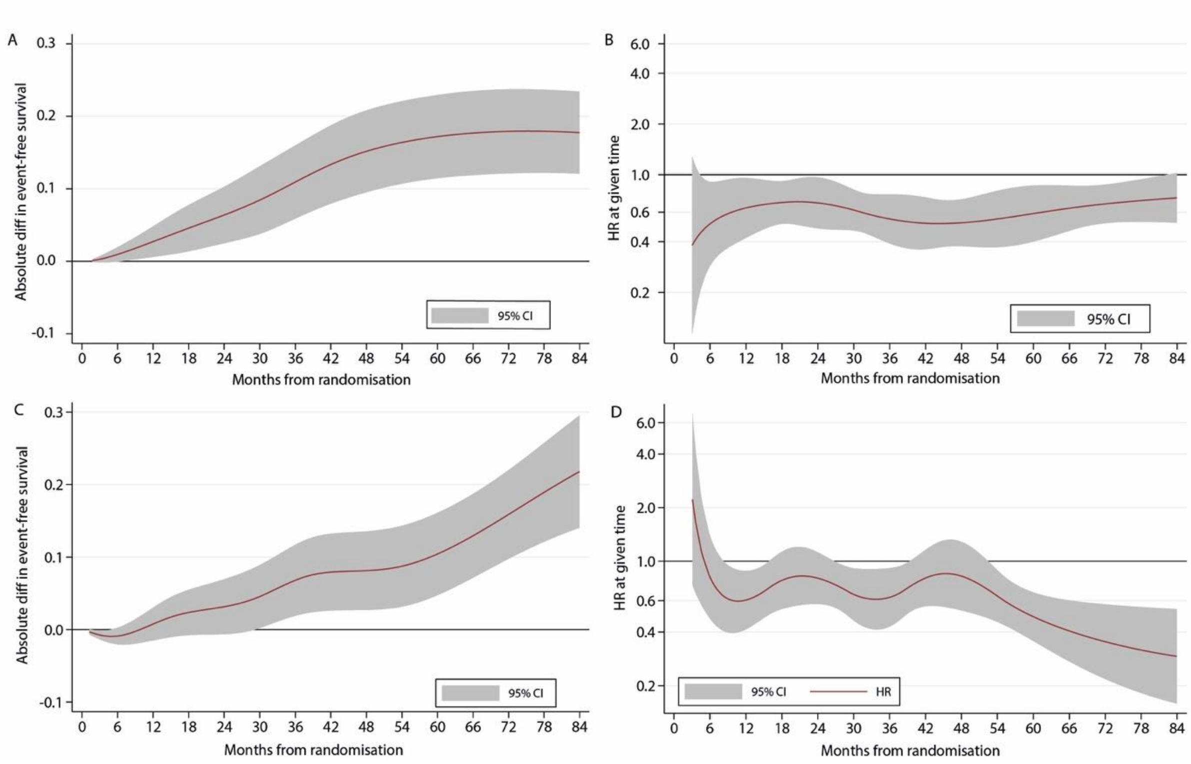

Figure S4. Failure-free survival split by low (A) and high (B) volume in the abiraterone trial and low (C) and high (D) volume in the abiraterone and enzalutamide trial.

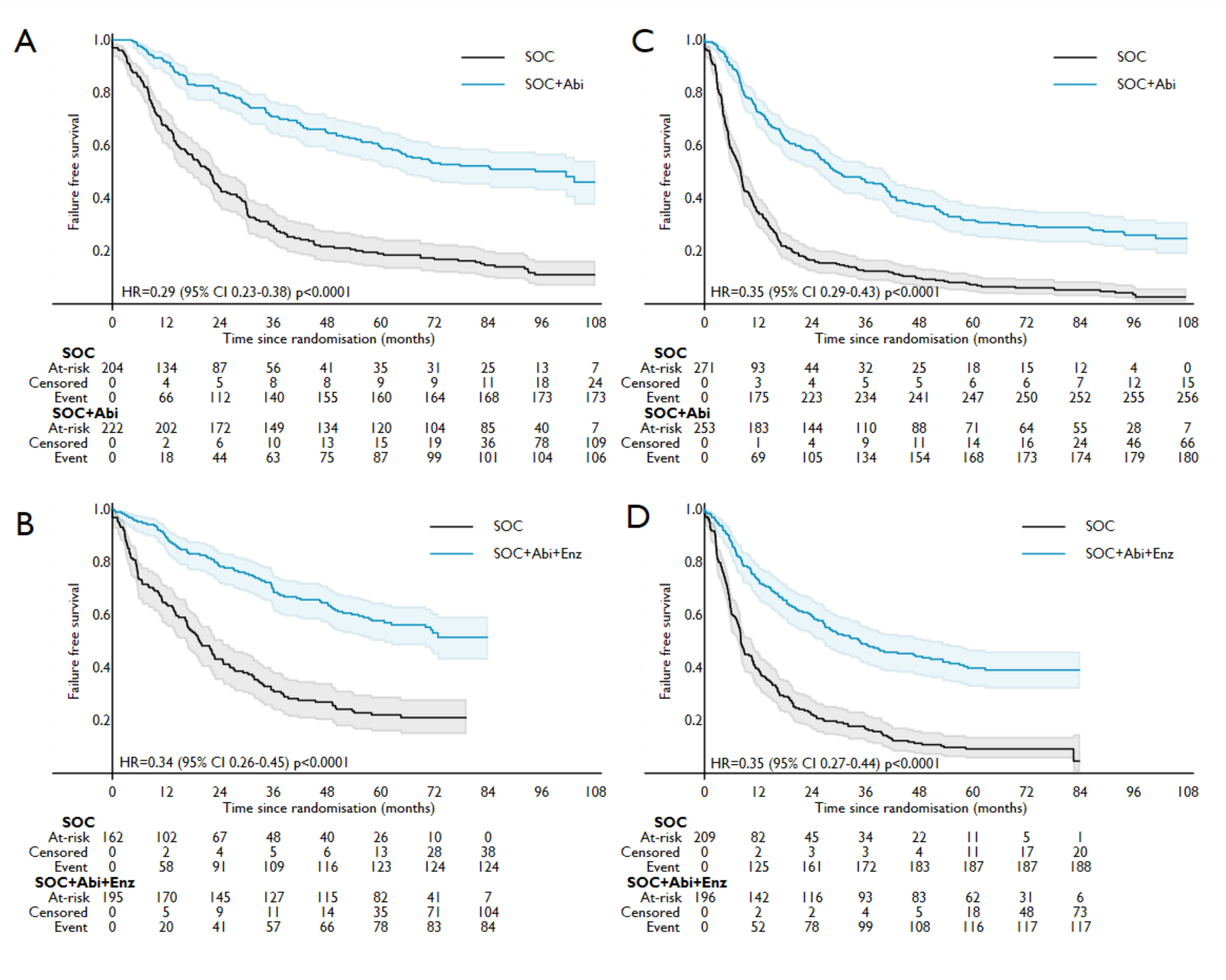

SOC, standard-of-care, Abi, abiraterone acetate and prednisolone; Enz, enzalutamide

Figure S5. Progression-free survival in the abiraterone (**A**) and abiraterone and enzalutamide (**B**) trials.

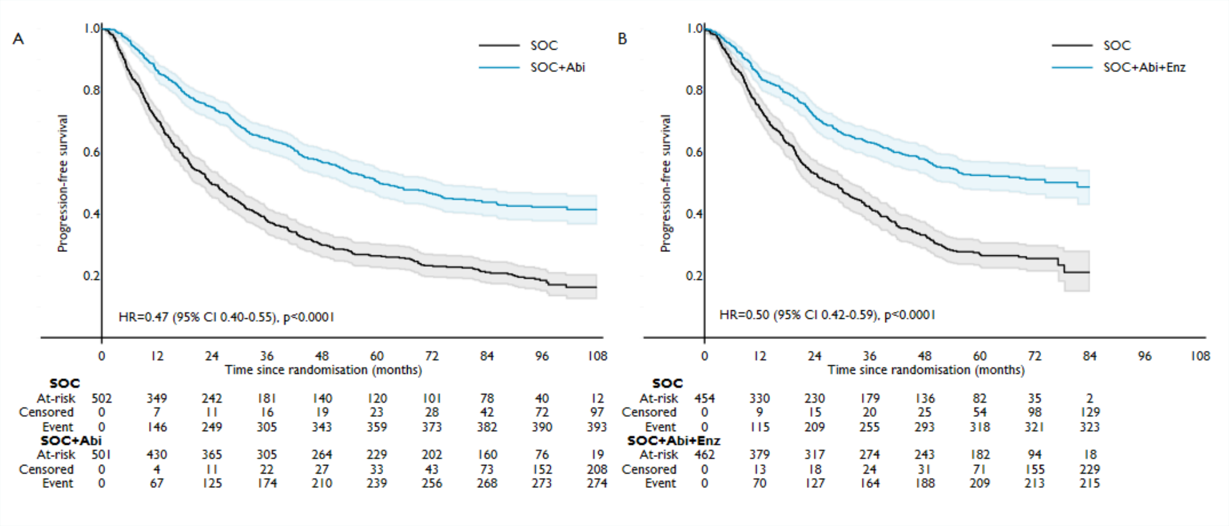

SOC, standard-of-care, Abi, abiraterone acetate and prednisolone; Enz, enzalutamide

Figure S6. Symptomatic skeletal-related events in the abiraterone (**A**) and abiraterone and enzalutamide (**B**) trials.

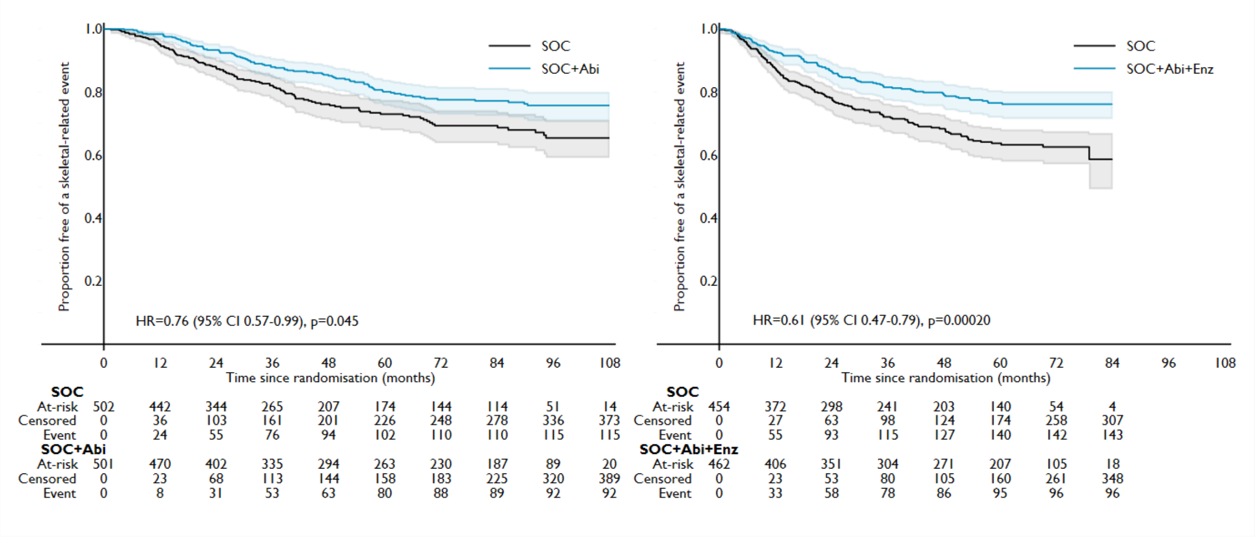

Reporting of skeletal-related event was updated to distinguish by whether this constituted progression or not in STAMPEDE protocol version 12 implemented July 29, 2014, leading to reporting of relatively more events in the abiraterone and enzalutamide trial.

SOC, standard-of-care, Abi, abiraterone acetate and prednisolone; Enz, enzalutamide

Figure S7. First life-prolonging treatment administered after progression. Proportion of patients for whom administration of a life-prolonging treatment was reported are included. Life-prolonging treatments used in clinical practice at time of report: abiraterone, enzalutamide, docetaxel, radium(223), cabazitaxel.

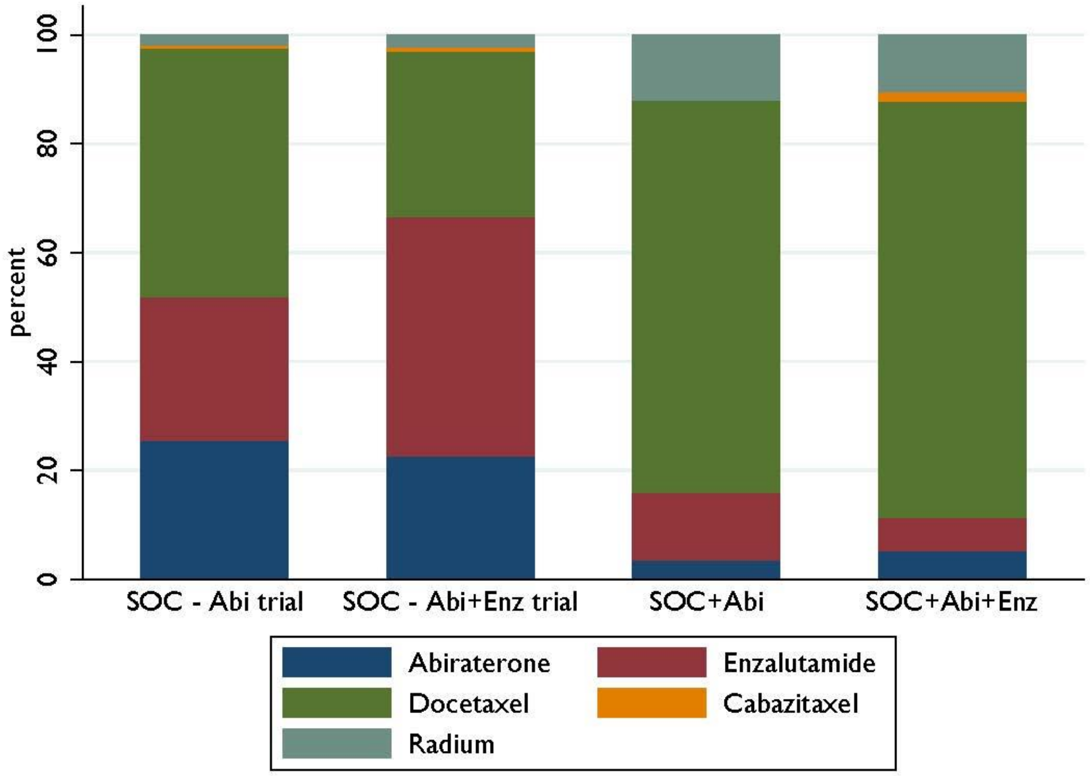

SOC, standard-of-care, Abi, abiraterone acetate and prednisolone; Enz, enzalutamide

Figure S8. Time from starting to permanently stopping research treatments. All patients are included, those reported stopping are censored at time of last contact

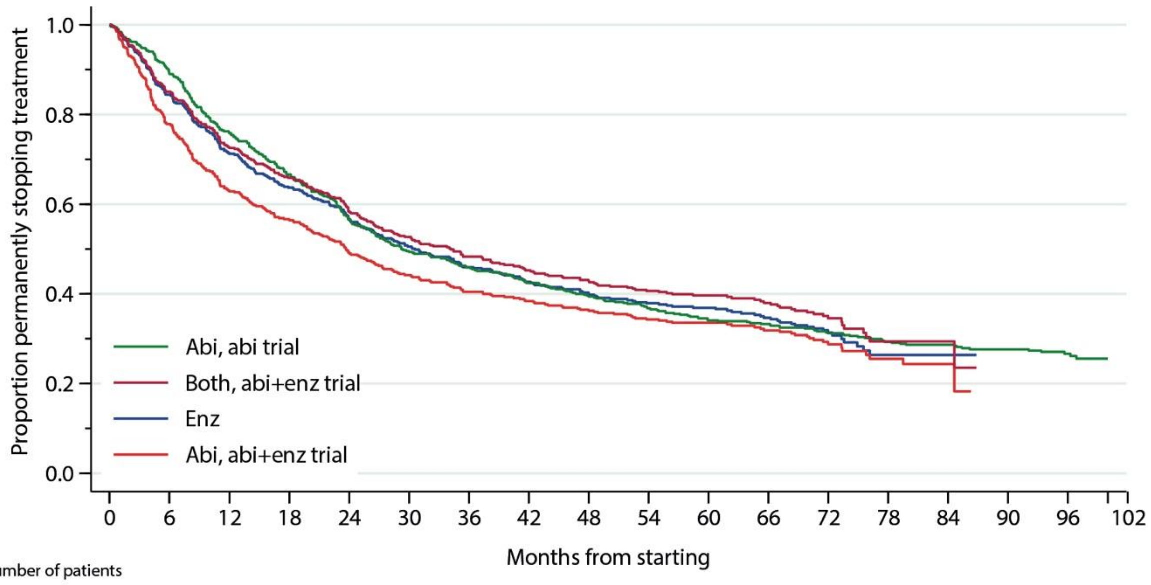

Number of patients

Abi, abi trial	501	(121)	378	(96)	282	(53)	226	(31)	195	(27)	167	(14)	150	(12)	119	(7)	56
Both, abi+enz trial	462	(125)	328	(63)	258	(44)	208	(22)	179	(14)	137	(14)	65	(6)	9	(1)	0
Enz	462	(131)	323	(65)	251	(47)	199	(24)	169	(14)	130	(16)	61	(6)	8	(0)	0
Abi, abi+enz trial	462	(169)	284	(61)	218	(38)	174	(17)	153	(12)	116	(13)	54	(5)	8	(1)	0

Abi, abiraterone acetate and prednisolone; Enz, enzalutamide

Figure S9. Adverse events. **A** Time from treatment start to reporting of grade 3-5 toxicity split by treatment allocation. **B** Adverse events of special interest split by treatment allocation. Also, one grade 4 ALT increase and one grade 4 AST increase with abiraterone acetate and prednisolone and one grade 4 ALT increase with abiraterone acetate and prednisolone and enzalutamide. STAMPEDE protocol v15 implemented 15 May 2016 updated reporting from CTCAE version 3 to CTCAE version 4. This could result in capture of more events in patients randomised nearer this date. Rates can be compared for directly randomised patients (intra-trial) and not inter-trial.

SOC, standard-of-care; abi, abiraterone acetate and prednisolone; enz, enzalutamide.

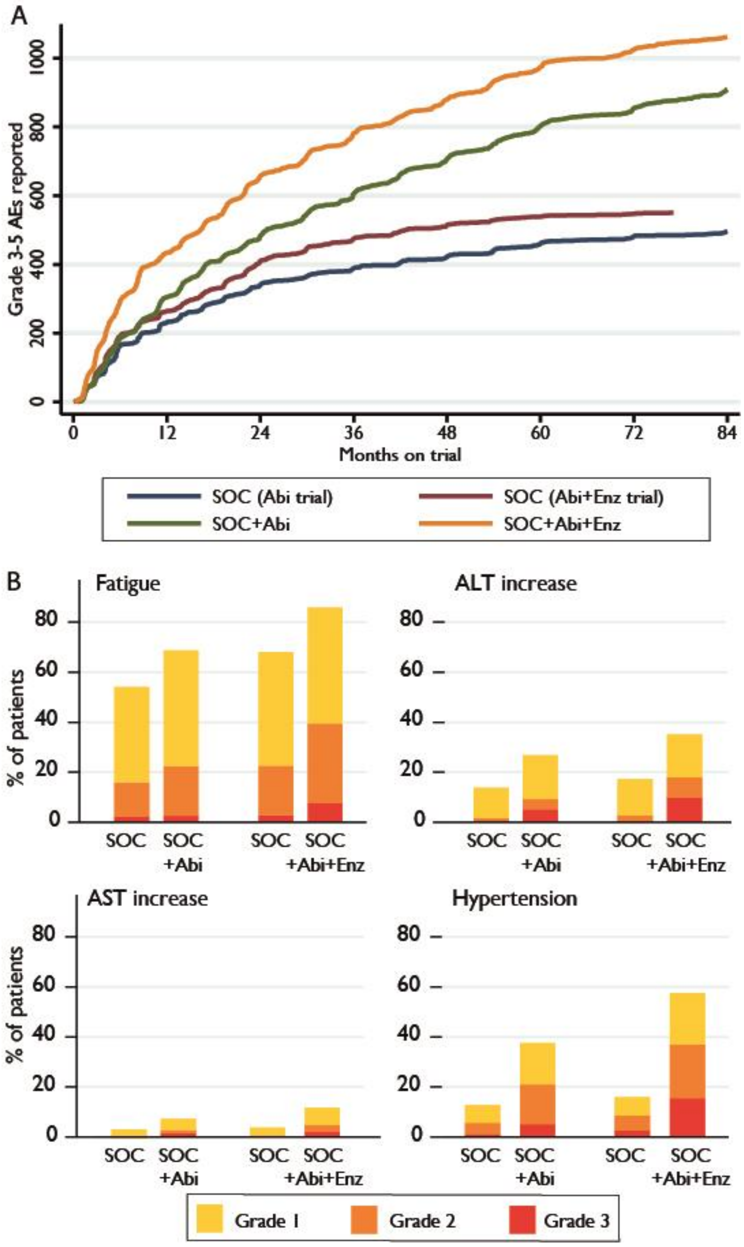

Figure S10. Grade 5 adverse events

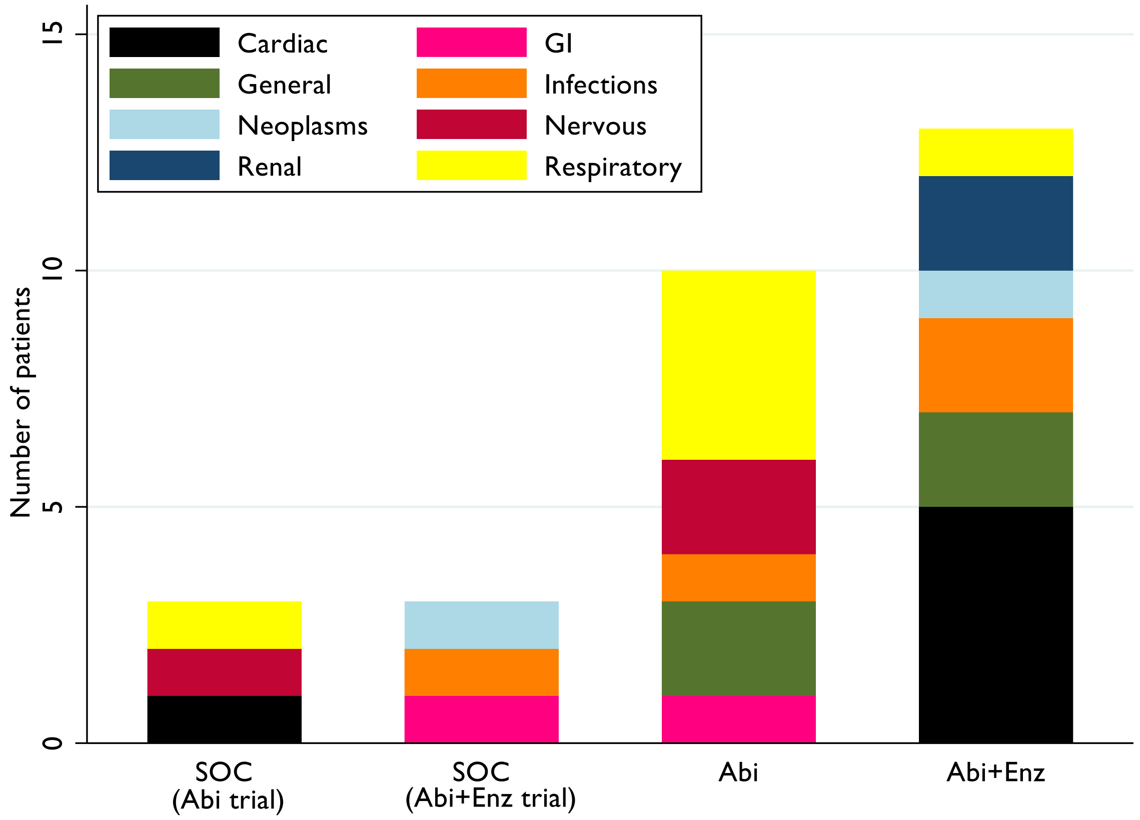

SOC, standard-of-care; Abi, abiraterone acetate and prednisolone; Enz, enzalutamide

STAMPEDE

Systemic Therapy in Advancing or Metastatic Prostate Cancer: Evaluation of Drug Efficacy

A multi-arm multi-stage randomised controlled trial

Version: 21.0
Date: 20 October 2020

MRC CTU AT UCL ID:	PR08
ISRCTN #:	ISRCTN78818544
NCT #:	NCT00268476
EUDRACT #:	2004-000193-31
CTA #:	20363/0404/001
MREC #:	04/MRE07/35

Authorised by:

Name: Professor Nicholas D James
Role: Chief Investigator & Comparison CI for "Abiraterone comparison"
Signature:

Name: Professor Louise Brown
Role: Trial Statistician
Signature:

Authorised by:

**Name:**	**PROFESSOR CHRIS PARKER**
Role:	**CCI “M1|RT COMPARISON”**
Signature:	
**Name:**	**PROFESSOR GERTHARDT ATTARD**
Role:	**CCI “ENZALUTAMIDE & ABIRATERONE COMPARISON”**
Signature:	
**Name:**	**PROFESSOR NOEL CLARKE**
Role:	**CO-CCI "METFORMIN COMPARISON"**
Signature:	
**Name:**	**PROFESSOR SILKE GILLESSEN**
Role:	**CO-CCI "METFORMIN COMPARISON"**
Signature:	
**Name:**	**PROFESSOR RUTH LANGLEY**
Role:	**CCI "TRANSDERMAL OESTRADIOL COMPARISON"**
Signature:	

GENERAL INFORMATION

This document was constructed using the MRC CTU at UCL Protocol Template Version 4.0. It describes the STAMPEDE trial, coordinated by the Medical Research Council (MRC) Clinical Trials Unit (CTU) at University College London (UCL), and provides information about procedures for entering patients into it. The protocol should not be used as an aide-memoire or guide for the treatment of other patients. Every care has been taken in drafting this protocol, but corrections or amendments may be necessary. These will be circulated to the registered investigators in the trial, but sites entering patients for the first time are advised to contact the Cancer And Other Non-Infectious Diseases Group, MRC CTU at UCL, London, UK, to confirm they have the most up-to-date version.

COMPLIANCE

The trial will be conducted in compliance with the approved protocol, the Declaration of Helsinki 1996, the principles of Good Clinical Practice (GCP), Commission Directive 2005/28/EC with the implementation in national legislation in the UK by Statutory Instrument 2004/1031 and subsequent amendments, the UK Data Protection Act (DPA number: Z6364106), and the National Health Service (NHS) Research Governance Framework for Health and Social Care (RGF).

*Until the Clinical Trials Regulation EU No 536/2014 becomes applicable, the trial will be conducted in accordance with the Clinical Trials Directive as implemented in the UK statutory instrument. When the directive is repealed on the day of entry into application of the Clinical Trial Regulation the trial will work towards implementation of the Regulation (536/2014) following any transition period.

International sites will comply with the principles of GCP as laid down by the ICH topic E6 (Note for Guidance on GCP), Commission Directive 2005/28/EC (the European Directive 2001/20/EC [where applicable]) and applicable national regulations.

SPONSOR

UCL is the sponsor of STAMPEDE and MRC CTU has been delegated responsibility for the overall management of STAMPEDE. Queries relating to UCL sponsorship should be addressed to the Director, Professor Max Parmar, Institute of Clinical Trials & Methodology, MRC CTU at UCL, 2nd Floor, 90 High Holborn, London, WC1V 6LJ UK, or via the STAMPEDE Trial Team.

FUNDING

Cancer Research UK's Clinical Research Committee (formerly the Clinical Trials Advisory Awards Committee), Medical Research Council, and educational grants from Novartis, Sanofi-Aventis, Pfizer, Janssen Pharma NV, Astellas, Clovis Oncology.

AUTHORISATIONS AND APPROVALS

The following persons are authorised to sign the final protocol and protocol amendments for the sponsor: Chief Investigator and Trial Statistician and the Co-Chief-investigators for each comparison subsequently added.

TRIAL REGISTRATION

This trial has been registered with the ClinicalTrials.gov Clinical Trials Register, where it is identified as NCT00268476.

RANDOMISATION
Call MRC CTU at UCL, Monday to Friday 0900-1700
Excluding public holidays or dates when notice has been given by the Unit.
Tel: +44 (0) 20 7670 4777

SAE REPORTING
Fax to 020 7670 4818 within 24 hours of becoming aware of the event
Or send via **encrypted** email to mrcctu.stampede@ucl.ac.uk

COORDINATING SITE

MRC Clinical Trials Unit at UCL	Switchboard:	020 7670 4700
90 High Holborn	Fax:	020 7670 4818
2nd Floor	Email:	mrcctu.stampede@ucl.ac.uk
London WC1V 6LJ	Website:	www.stampedetrial.org
UK		

MRC CTU AT UCL STAFF

Trial Manager:	Michelle Buckner	Tel:	0207 670 4840
Trial Manager:	Sarah Jackson	Tel:	0207 670 4674
Trial Manager	Dymphna Lee	Tel:	0207 670 4916
Trial Manager	Malissa Richmond	Tel:	0207 670 4910
Trial Manager:	Christopher Wanstall	Tel:	0207 670 4882
Trial Manager	Melanie Weiss	Tel:	0207 670 4843
Clinical Project Manager	Claire Amos	Tel:	0207 670 4771
Clinical Project Manager	Nafisah B Atako	Tel:	0207 670 4896
Clinical Project Manager	Cheryl Pugh	Tel:	0207 670 4935

FOR ALL OTHER CONTACTS: http://www.stampedetrial.org/contact-us/trial-team/

SAAK COORDINATING CENTRE

Zuzanna Maniecka   Bern, Switzerland

Chief Investigator

Prof Nicholas James

Prostate and Bladder Cancer Research Team Leader
The Institute of Cancer Research,
237 Fulham Road London, SW3 6JB
UK

Tel: 020 7153 5130 (PA)

Email: nick.james@icr.ac.uk

Co-investigators and TMG members

Prof Gerhardt Attard<br>Oncologist	London, UK	CCI "Enzalutamide+Abiraterone comparison"
Dr Simon Chowdhury<br>Oncologist	London, UK	
Prof Noel Clarke<br>Urologist	Manchester, UK	TMG Vice-Chair<br>Co-CCI "Metformin comparison"
Mr William Cross<br>Urologist	Leeds, UK	
Prof David Dearnaley<br>Oncologist	London, UK	
Dr Duncan Gilbert<br>Oncologist	London & Brighton, UK	
Prof Silke Gillessen<br>Oncologist	St Gallen, Switzerland	Co-CCI "Metformin comparison"
Prof Rob Jones<br>Oncologist	Glasgow, UK	
Prof Ruth Langley<br>Oncologist	London & Brighton, UK	CCI "Transdermal oestradiol comparison"
Dr Zafar Malik<br>Oncologist	Liverpool, UK	
Prof Chris Parker<br>Oncologist	London, UK	CCI "M1|RT comparison"
Dr Martin Russell<br>Oncologist	Glasgow, UK	
Robin Millman<br>Patient representative	UK	
David Matheson,<br>Patient representative	UK	
Prof Mark Sculpher<br>Health Economics Advisor	York, UK	
Rachel Hunter<br>PRIMENT UCL	London, UK	
Dr Amanda Adler<br>Metformin adviser	Cambridge UK	
Dr Hannah Rush<br>Clinical Research Fellow	London, UK	

Dr Archie Macnair<br>Clinical Research Fellow	London, UK
**TRIAL ADVISORY GROUPS**	**CHAIRS**
Biological Research Group<br>STRATOSPHere consortium	Prof Gerhardt Attard
Bone & Imaging Group	Prof Noel Clarke
Clinical Safety Committee	Prof Noel Clarke
Comparison Management Groups	Each led by Comparison Chief Investigator(s)
Metabolic Translational Group	Prof Silke Gillessen
Outcome Review Group	Prof Robert Jones
Site Advisory Team	Mr William Cross
Quality of Life Group	Prof Nicholas James

CONTENTS

GENERAL INFORMATION 3

CONTENTS 7

SUMMARY OF TRIAL 11

ABBREVIATIONS & GLOSSARY 19

2.1 INTRODUCTION AND SETTING 28
2.1.1 Long-term Androgen Deprivation Therapy 28
2.1.2 Role Of SOC Radiotherapy 28
2.1.3 Role Of SOC systemic therapy: docetaxel, abiraterone, enzalutamide or apalutamide in addition to ADT 29
2.2 DESIGN 30
2.3 PREVIOUSLY-REPORTED RESEARCH TREATMENTS 30
2.4 COMPARISONS IN FOLLOW-UP 31
2.5 RATIONALE FOR RECRUITING COMPARISONS 31
2.5.1 Metformin 31
2.5.2 Transdermal Oestradiol 32
3.1 SITE/INVESTIGATOR CRITERIA 34
3.1.1 Principle Investigator's Qualifications & Agreements 34
3.1.2 Adequate Resources 35
3.2 COMPARISON-SPECIFIC SITE ACCREDITATION 35
3.2.1 Transdermal Oestradiol Comparison: arms A & L 35
3.3 REQUIRED TRIAL DOCUMENTATION 36
4.1 IDENTIFYING POTENTIAL TRIAL PARTICIPANTS 37
4.2 APPROACH TO INFORMED CONSENT 37
4.2.1 Screening Investigations Prior To Randomisation 37
4.2.2 Baseline Investigations required for participants allocated to arms A, K, L 38
4.3 PRIOR PERMITTED SOC TREATMENTS 38
4.3.1 Hormone Treatment Prior To Randomisation 38
4.3.2 Standard-Of-Care (SOC) Radiotherapy 39
4.3.3 Standard-Of-Care (SOC) Systemic Therapy; docetaxel, abiraterone, enzalutamide or apalutamide 39
4.4 GENERAL INCLUSION CRITERIA – DISEASE CATEGORIES 40
4.4.1 High-Risk Newly-Diagnosed Non-Metastatic Node-Negative (N0/Nx) Disease 40
4.4.2 Newly-Diagnosed Metastatic Or Node-Positive Disease 40
4.4.3 Previously Radically Treated, Now Relapsing (Prior Radical Surgery And/or Radiotherapy) 40
4.5 GENERAL INCLUSION CRITERIA REQUIRED FOR ALL PARTICIPANTS 40
4.6 GENERAL EXCLUSION CRITERIA 41
4.7 COMPARISON-SPECIFIC ELIGIBILITY CRITERIA 42
4.7.1 Metformin Comparison (randomisation between arm A and arm K) 42
4.7.2 Transdermal Oestradiol Comparison (randomisation between arm A and arm L) 42
4.8 SUB-STUDY ELIGIBILITY CRITERIA 43
4.8.1 Eligibility for germline DNA sub-study (Saliva samples) 43
4.8.2 Eligibility for the circulating tumour DNA sub-study (sequential blood samples) 43
4.8.3 Eligibility for tumour sample analysis (FFPE blocks) 43
4.8.4 Eligibility for metformin metabolic sub-study 43
5.1 RANDOMISATION 44
5.2 CO-ENROLMENT GUIDELINES 44

6.1 STANDARD-OF-CARE (SOC) 46
6.1.1 Androgen Deprivation Therapy 46
6.1.2 SOC Radiotherapy to prostate 47
6.1.3 Administration of SOC RT 47
6.1.4 SOC upfront systemic therapy: docetaxel, abiraterone, enzalutamide or apalutamide 48
6.2 RESEARCH TREATMENTS 49
6.2.1 Treatment Duration 49
6.2.2 Research Abiraterone + Prednisolone (relevant to Arms G & J) 51
6.2.3 Research Enzalutamide (Arm J) 60
6.2.4 Research Metformin (Arm K) 63
6.2.5 Research Transdermal Oestradiol (Arm L) 68
6.3 CONCOMITANT TREATMENTS 71
6.4 TRIAL PRODUCTS 71
6.5 TREATMENT DATA COLLECTION 71
6.6 MEASURES OF COMPLIANCE/ADHERENCE 71
7.1 SCHEDULE FOR ASSESSMENTS 73
7.1.1 Follow-up Schedules 73
7.1.2 PSA, Testosterone And Oestradiol Measurements 73
7.1.3 Assessment Of Treatment Failure (Definition Of Progression) 73
7.1.4 Additional Metabolic And Cardiovascular Outcomes 75
7.1.5 Additional Safety Assessments 76
7.2 DATA COLLECTION PROCEDURES 77
7.2.1 Data Collection For SOC Hormone Therapy 77
7.2.2 Data Collection For SOC Systemic therapy: docetaxel, abiraterone, enzalutamide, or apalutamide 77
7.2.3 Data Collection And Non-Administration Of Standard Radiotherapy 78
7.2.4 Data Collection For Palliative Radiotherapy 78
7.2.5 Data Collection for Research (M1) Radiotherapy 78
7.2.6 Data Collection for Additional Treatments Given for Disease Progression 78
7.3 FOLLOW-UP PROCEDURE 79
7.3.1 Follow-up Telephone Consultations 79
7.3.2 Follow-up Using Electronic Healthcare Records 79
7.4 TRIAL & COMPARISON CLOSURE 80
7.4.1 Comparisons for which Follow-up has Ceased 80
8.1 STOPPING RESEARCH INTERVENTIONS 84
8.1.1 Stopping Research Treatment: Abiraterone, Enzalutamide + Abiraterone 84
8.1.2 Stopping Research Treatment: Metformin 84
8.1.3 Stopping Research Treatment: Transdermal Oestradiol 85
8.2 BREAKS IN SOC ADT 85
8.3 PARTICIPANT TRANSFERS 86
8.4 EARLY CESSATION OF TRIAL PARTICIPATION 86
9.1 METHOD OF RANDOMISATION 88
9.2 OUTCOME MEASURES 88
9.3 SAMPLE SIZE: PRINCIPLES 89
9.4 SAMPLE SIZE ISSUES & TRIAL STAGES: ADDITIONAL RESEARCH ARM H 92
9.4.1 Pilot Phase: Additional Research Arm H 92
9.4.2 Activity Stages I-III: Additional Research Arm H 92
9.4.3 Efficacy Stage IV: Additional Research Arm H 92
9.4.4 Sample Size For Additional Research Arm H 92
9.5 SAMPLE SIZE ISSUES & TRIAL STAGES: ADDITIONAL RESEARCH ARM J 94
9.5.1 Pilot Phase: Additional Research Arm J 94
9.5.2 Activity Stages I-II: Additional Research Arm J 94

9.5.3 Efficacy Stage III: Additional Research Arm J 94
9.5.4 Sample Size For Additional Research Arm J 95
9.5.5 Update to Efficacy Stage Analysis For Additional Research Arm J 95
9.6 SAMPLE SIZE ISSUES & TRIAL STAGES: ADDITIONAL RESEARCH ARM K 96
9.6.1 Implementation: Additional Research Arm K 96
9.6.2 Outcome Measures: Additional Research Arm K 97
9.6.3 Pilot Phase: Additional Research Arm K 98
9.6.4 Activity Stage I: Additional Research Arm K 98
9.6.5 Efficacy Stage II: Additional Research Arm K 98
9.6.6 Sample Size For Additional Research Arm K 99
9.6.7 Further Sample Size Issues For Additional Research Arm K 99
9.7 SAMPLE SIZE ISSUES & TRIAL STAGES: ADDITIONAL RESEARCH ARM L 100
9.7.1 Implementation And Outcome Measures: Additional Research Arm L 100
9.7.2 Additional Use of Outcome Data from the “transdermal oestradiol comparison” 101
9.7.3 Definition of PFS and Use As Co-primary Outcome Measure: Additional Research Arm L 101
9.7.4 Abiraterone/Enzalutamide/Apalutamide enhanced safety monitoring 102
9.8 FURTHER NOTES ON TRIAL DESIGN 102
9.8.1 Overall Sample Size 102
9.8.2 Factorial Design 102
9.9 INTERIM MONITORING AND ANALYSES 103
9.10 OUTLINE ANALYSIS PLAN 103
9.10.1 Pilot / Safety Phases 104
9.10.2 Activity And Efficacy Stages 104
10.1 DATA MONITORING 105
10.1.1 Central Monitoring Of Consent 105
10.1.2 Central Monitoring Of Data 105
10.1.3 Direct Access to Patient Data 105
10.1.4 Monitoring Visits to Investigator Sites 105
10.2 CONFIDENTIALITY 106
11.1 SAFETY REPORTING DEFINITIONS 107
11.1.1 Adverse event definitions 108
11.1.2 Defining “treatment” for the purposes of safety reporting 108
11.2 SITE INVESTIGATOR RESPONSIBILITIES 109
11.2.1 Notification period 109
11.2.2 Trial-Specific “Expedited Reporting” Exemptions 110
11.2.3 Investigator Assessment 111
11.2.4 Event Follow-up 113
11.3 CTU RESPONSIBILITIES 114
11.3.1 Sponsor (CTU) Assessment 114
12.1 ETHICAL CONSIDERATIONS 116
12.2 ETHICAL APPROVAL 117
13.1 DATA COLLECTION & RETENTION 119
16.1 TRIAL MANAGEMENT GROUP (TMG) 122
16.2 TRIAL STEERING COMMITTEE (TSC) 122
16.3 INDEPENDENT DATA MONITORING COMMITTEE (IDMC) 122
16.4 TMG SUB-GROUPS AND EXPERT PANELS 123
16.5 MRC CTU AT UCL INTERNAL GROUPS 123
17.1 PATIENT REPORTED OUTCOMES 125
17.1.1 Quality of life (QL) 125
17.1.2 Health Economics 125
17.2 TRANSLATIONAL SUB-STUDIES 127
17.2.1 Germline DNA Analysis 127

17.2.2 Circulating Tumour-DNA Analysis (Sequential Blood Samples) 127
17.2.3 Tissue Sample Analysis (FFPE Blocks) 127
17.2.4 Biomarker-Screening Pilot 128
17.2.5 Informed consent to receive results arising from genetic sub-studies 128
17.3 DISEASE VOLUMETRIC ANALYSIS SUB-STUDY 128
17.4 USING ROUTINE DATA TO IDENTIFY CLINICAL TRIAL OUTCOMES 128
17.5 METFORMIN METABOLIC SUBSTUDY 129
18.1 POTENTIAL IMPACT OF PPI 130
18.2 PATIENT REPRESENTATIVES 130
21.1 PROTOCOL 133
21.1.1 Amendments Made To Protocol Version 1.0 (May-2004) 133
21.1.2 Amendments Made To Protocol Version 1.1 (May-2005) 133
21.1.3 Amendments Made To Protocol Version 2.0 (Jun-2005) 133
21.1.4 Amendments Made To Protocol Version 3.0 (Jul-2006) 134
21.1.5 Amendments Made To Protocol Version 4.0 (Dec-2007) 135
21.1.6 Amendments Made To Protocol Version 5.0 (Aug-2008) 135
21.1.7 Amendments Made To Protocol Version 6.0 (Jul-2009) 136
21.1.8 Amendments Made To Protocol Version 7.0 (Jul--2011) 136
21.1.9 Amendments Made To Protocol Version 7.1 (Jul-2011) 137
21.1.10 Amendments Made To Protocol Version 8.0 (Sep-2011) 138
21.1.11 Amendments Made To Protocol Version 9.0 (Oct-2012) 139
21.1.12 Amendments Made To Protocol Version 10.0 (Apr-2013) 139
21.1.13 Amendments Made To Protocol Version 11.0 (Sep-2013) 140
21.1.14 Amendments Made To Protocol Version 12.0 (Jan-2014) 140
21.1.15 Amendments Made To Protocol Version 13.0 (Feb-2015) 140
21.1.16 Amendments Made To Protocol Version 14.0 (Oct-2015) 141
21.1.17 Amendments Made To Protocol Version 15.0 (Mar-2017) 142
21.1.18 Amendments Made To Protocol Version 16.0 (Oct-2017) 142
21.1.19 Amendments Made To Protocol Version 17.0 (Feb-2017) 143
21.1.20 Amendments made to Protocol Version 18.0 (Jun-2018) 144
21.1.21 Amendments made to Protocol Version 19.0 (Aug-2020) 145
21.1.22 Amendments made to Protocol Version 20.0 (Oct-2020) 147

SUMMARY OF TRIAL

SUMMARY INFORMATION TYPE	SUMMARY DETAILS
Acronym	STAMPEDE
Long Title of Trial	Systemic Therapy in Advancing or Metastatic Prostate Cancer: Evaluation of Drug Efficacy: A multi-arm multi-stage randomised controlled trial
Version	21.0
Date	20-October-2020
MRC CTU at UCL ID	PR08
NCT #	NCT00268476
EudraCT #	2004-000193-31
Study Design	Multi-arm multi-stage platform randomised controlled trial
Type of Participants to be Studied	People starting long-term hormone therapy for metastatic or high-risk non-metastatic prostate cancer
Setting	Tertiary care
Interventions to be Compared	Various - see comparison-specific tables
Study Hypothesis	Various - see comparison-specific tables
Definitive Primary Outcome Measure	Overall survival (unless stated)
Intermediate Primary Outcome Measure	Failure-free survival (unless stated)
Secondary Outcome Measure(s)	Toxicity<br>Symptomatic skeletal events<br>Quality-of-life<br>Cost-effectiveness
Randomisation	Minimisation using a random element across a number of stratification factors
Number of Participants	See comparison-specific tables
Duration	See comparison-specific tables
Sponsor	University College London
Funders	Cancer Research UK<br>Medical Research Council<br>Astellas<br>Clovis Oncology<br>Janssen<br>Novartis<br>Pfizer<br>Sanofi-Aventis

**SUMMARY INFORMATION TYPE**	**SUMMARY DETAILS**
"Original comparisons"	
Type of Participants to be Studied	People starting long-term hormone therapy for metastatic or high-risk non-metastatic prostate cancer
Control Arm	• Arm A: Standard-of-care (SOC)
Interventions to be Compared	• Arm B: SOC + zoledronic acid<br>• Arm C: SOC + docetaxel<br>• Arm D: SOC + celecoxib<br>• Arm E: SOC + zoledronic acid + docetaxel<br>• Arm F: SOC + zoledronic acid + celecoxib
Allocation ratio	• 2 control arm : 1 research arm [2A:1B:1C:1D:1E:1F]
Study Hypothesis	Research interventions will improve survival over SOC
Definitive Primary Outcome Measure	Overall survival
Intermediate Primary Outcome Measure	Failure-free survival
Status	Primary results published and **active follow-up discontinued** Summer 2018 (1, 2)
"Abiraterone comparison"	
Type of Participants to be Studied	People starting long-term hormone therapy for metastatic or high-risk non-metastatic prostate cancer
Control arm	• Arm A: Standard-of-care (SOC)
Intervention to be Compared	• Arm G: SOC + abiraterone
Allocation ratio	• 1 control arm : 1 research arm [1A:1G]
Study Hypothesis	Addition of abiraterone to SOC will improve survival over SOC alone
Definitive Primary Outcome Measure	Overall survival
Intermediate Primary Outcome Measure	Failure-free survival
Number of Participants	Around 1,800 patients for 267 control arm definitive primary outcome measure events
Duration	6 to 8 years
Status	Primary results published (3), remains on active follow-up to permit a further longer-term analysis
"M1|RT comparison"	
Type of Participants to be Studied	People starting long-term hormone therapy for newly-diagnosed metastatic prostate cancer with no contraindication to prostate radiotherapy
Control arm	• Arm A: Standard-of-care (SOC)
Intervention to be Compared	• Arm H: SOC + radiotherapy to the prostate (RT)
Allocation ratio	• 1 control arm : 1 research arm [1A:1H]
Study Hypothesis	Addition of RT to SOC will improve survival over SOC alone

SUMMARY INFORMATION TYPE	SUMMARY DETAILS
Definitive Primary Outcome Measure	Overall survival
Intermediate Primary Outcome Measure	Failure-free survival
Number of Participants	Around 1,800 patients for 267 control arm definitive primary outcome measure events
Duration	6 to 8 years
Status	Primary results published (4) remains on active follow-up to permit a further longer-term analysis
"Enzalutamide + abiraterone comparison"	
Type of Participants	People starting long-term hormone therapy for metastatic or high-risk non-metastatic prostate cancer
Control Arm	• Arm A: Standard-of-care (SOC)
Interventions to be Compared	• Arm J: SOC + enzalutamide + abiraterone
Allocation ratio	• 1 control arm : 1 research arm [1A:1J]
Study Hypothesis	Addition of enzalutamide, in combination with abiraterone, to SOC will improve survival over SOC alone
Definitive Primary Outcome Measure	Overall survival
Intermediate Primary Outcome Measure	Failure-free survival
Number of Participants	Around 1,800 patients for 267 control arm definitive primary outcome measure events
Duration	6 to 8 years
Status	In follow-up
"Metformin comparison"	
Type of Participants to be Studied	Non-diabetic people, with no contraindication to metformin, starting long-term hormone therapy for metastatic or high-risk non-metastatic prostate cancer
Control arm	• Arm A: Standard-of-care (SOC)
Intervention to be Compared	• Arm K: SOC + metformin
Allocation ratio	• 1 control arm : 1 research arm [1A:1K]
Study Hypothesis	Addition of metformin to SOC will improve survival over SOC alone
Definitive Primary Outcome Measure	Overall survival
Intermediate Primary Outcome Measure	Overall survival
Number of Participants	Around 2800 patients, including around 1,700 M1 (metastatic) patients, for 473 control arm definitive primary outcome measure events among M1 patients
Duration	7 years

SUMMARY INFORMATION TYPE	SUMMARY DETAILS
Status	Recruiting
"Transdermal oestradiol comparison"	
Type of Participants to be Studied	People starting long-term hormone therapy for metastatic or high-risk non-metastatic prostate cancer, having had no more than one 4-week (or one-month) LHRH (Luteinizing hormone releasing hormone) injection & 8 weeks of anti-androgens
Control arm	• Arm A: Standard-of-care (SOC)
Intervention to be Compared	• Arm L: Transdermal oestradiol ± RT ± docetaxel/abiraterone/enzalutamide/apalutamide
Allocation ratio	• 1 control arm : 1 research arm [1A:1L]
Study Hypothesis	Transdermal oestradiol will be non-inferior to standard hormone therapy, while having fewer side-effects and improved quality-of-life
Definitive Primary Outcome Measures	Co-primary endpoints of progression-free survival and overall survival
Intermediate Primary Outcome Measure	Progression-free survival
Number of Participants	Around 700 to include within a meta-analysis with the PATCH trial (EudraCT 2005-001030-33), which will include around 2,500 patients overall
Duration	4 to 6 years
Status	Recruiting

Figure 1: Randomisation schema from protocol v21.0 onwards

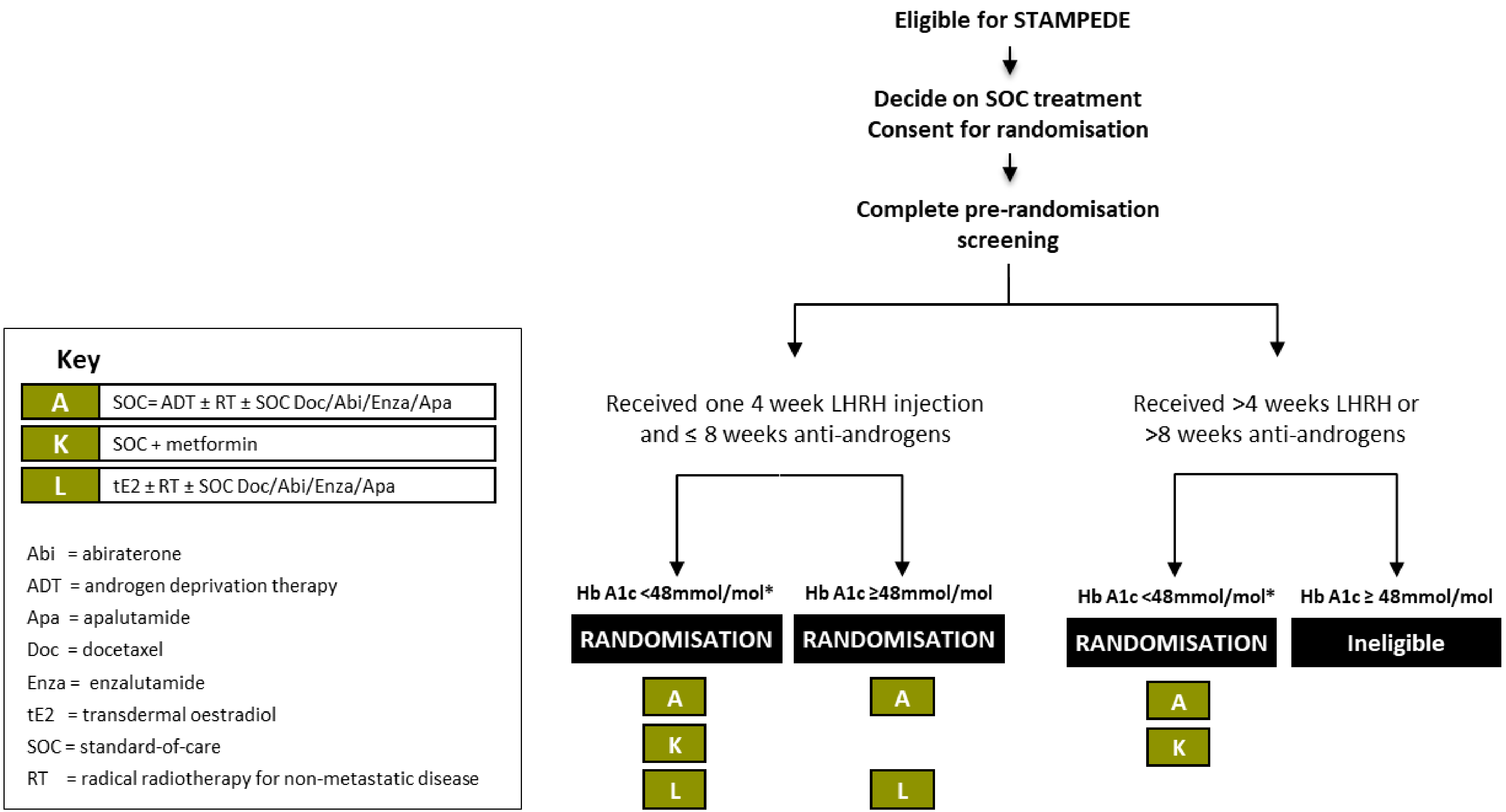

*Participants must not have received any treatment with any anti-diabetes medication but diet controlled diabetes is allowed if HbA1c now in limits.

Figure 2: Arms of the STAMPEDE trial open to recruitment over time

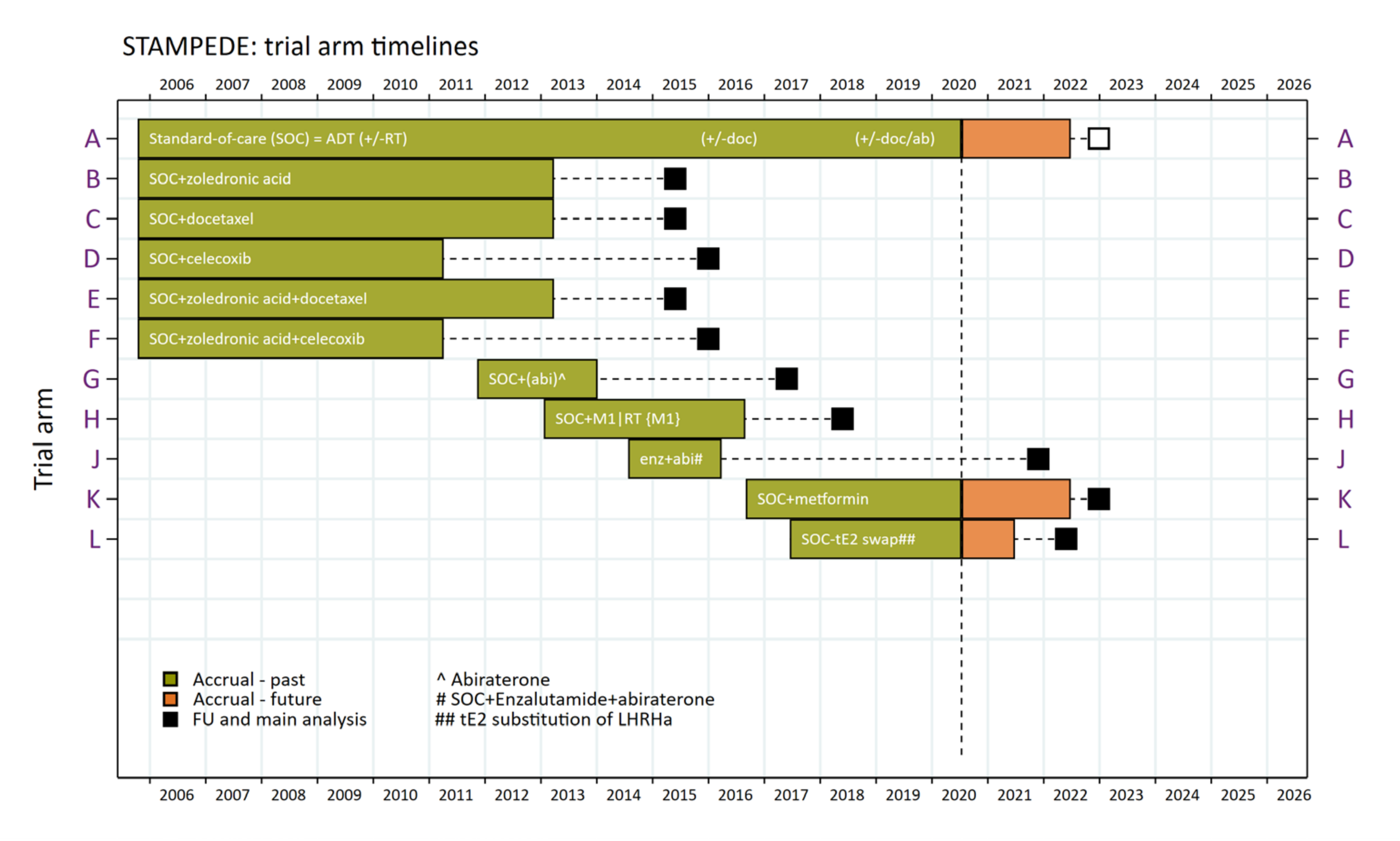

Table 1: Schedule of Assessments for Participants Randomised before 05-Sep-2016

	ASSESSMENT WEEK											ALL FURTHER VISITS [1]	AT EACH DISEASE EVENT [2]	END OF TRT	PRIOR TO 2ND LINE TRT
	4-6	12	18	24	36	48	60	72	84	96	104				
**Arm A/G/J**															
Blood collection cell-free DNA Streck™ tubes [3]						X [4]		X [4]	X [4]				X	X	X
Saliva sample [3]	Any time point														
FFPE block [3]	Once, at the point of request														
PSA	X	X	X	X	X	X	X	X	X	X	X	X			
Waist circumference + Weight	X	X	X	X	X	X	X	X	X	X	X	X			
Concomitant medication	X	X	X	X	X	X	X	X	X	X	X	X			
QL + HE [5,3]	X	X	X	X	X	X	X	X	X	X	X	X			
**Arm G&J only**															
Blood pressure[6]	X	X	X	X	X	X	X	X	X	X	X	X			
Safety bloods (LFTs and potassium) [6]	X	X	X	X	X	X	X	X	X	X	X	X			

[1] Follow-up visits after year 2 need to be carried out every 6 months for the first 5 years. At year 6 and onwards visits should be every 12 months whilst active follow-up continues.

[2] Disease events are defined as each type of disease progression: PSA (biochemical), clinical (symptomatic) and radiological (objective).

[3] Only if participating in relevant sub-study, for information regarding samples see the **Sample Collection & Handling Manual** available via the STAMPEDE website for details

[4] Sample only required for participants with metastatic disease at trial entry (M1)

[5] Review **Table 38** for a breakdown of participants that are still required to complete Quality-of-life (QL) + health economic (HE) questionnaires

[6] For participants receiving research abiraterone, BP, liver function tests (LFTs) and serum potassium monitoring is required 2-weekly in the first 12 weeks, then monthly until 12 months on treatment. For participants who have not experienced Toxicity following 12 months of treatment, this may be reduced to every 2 months whilst research abiraterone continues. Arm J participants continuing on enzalutamide alone may reduce to 3 monthly BP monitoring, no requirement for ongoing safety blood tests. Increased monitoring is required in participants experiencing toxicity; see **Table 13**, **Table 14** and **Table 15** for details.

Table 2: Schedule of Assessments for Participants Randomised on or after 05-Sep-2016

	Pre-Rand^{n}	ASSESSMENT WEEK											ALL FURTHER VISITS [1]	AT EACH DISEASE EVENT [2]	END OF TRT	PRIOR TO 2ND LINE TRT
		4-6	12	18	24	36	48	60	72	84	96	104				
**Arms A/K/L**																
Cardiac (BP)	X															
Screening bloods[3]	X															
Full radiological screening[4]	X															
WHO PS	X															
Blood collection cell-free DNA Streck™ tubes[5]	X						X[3]		X[3]	X[3]				X	X	X
Saliva sample[5]	Any time point															
FFPE block[5]	At the point of request															
Waist circumference + Weight	X	X	X	X	X	X	X	X	X	X	X	X	X			
Height	X															
QL & HE[5, 6]	X	X	X	X	X	X	X	X	X	X	X	X	X			
HbA1c & Lipid profile[7]	X				X [8]		X [8]					X [8]	X [8]			
Glucose & Triglycerides	X				X [8]		X[8]					X [8]	X [8]			
PSA	X[9]	X	X	X	X	X	X	X	X	X	X	X	X			
Concomitant medication	X	X	X	X	X	X	X	X	X	X	X	X	X			
**Arm K only**																
Safety bloods (eGFR) [10]					X		X		X		X		X			
**Arm L only**																
Testosterone & Oestradiol [11]		X[12]	X		X		X		X		X		X			

[1] Follow-up visits after year 2 need to be carried out every 6 months for the first 5 years. At year 6 and onwards visits should be every 12 months whilst active follow-up continues.

[2] Disease events are defined as each type of disease progression: PSA (biochemical), clinical (symptomatic) and radiological (objective).

[3] U&Es, LFTs, Serum creatinine and FBCs to be completed before randomisation. Cholesterol, albumin, serum corrected calcium, phosphate, magnesium within 4 weeks before or after randomisation.

[4] Pre-randomisation imaging must be representative of current disease status, see **section 4.2.1**

[5] Only if participating in relevant sub-study, for information regarding samples see the **Sample Collection & Handling Manual** available via the STAMPEDE website for details

[6] Review **Table 38** for a breakdown of participants that are still required to complete Quality-of-life (QL) + health economic (HE) questionnaires

[7] HBA1c required prior to randomisation for participants being considered for "metformin comparison".

[8] If missed, samples can be obtained +/-12 weeks of the scheduled FU visit, maintaining 10-12 weeks in between the tests due at week 24 and 48 weeks.

[9] Pre-ADT PSA must have been obtained within 6 months prior to randomisation and another PSA analysis should be completed within 2 weeks of randomisation.

[10] Increased monitoring of renal function required if renal function declines see **Table 23**. To continue until metformin permanently stopped.

[11] Hormone tests are required whilst the participant is receiving research transdermal oestradiol. Note that additional tests may be necessary as detailed in **Section 6.2.5.B**.

[12] First hormone tests for patients receiving research transdermal oestradiol should be at 4 weeks.

ABBREVIATIONS & GLOSSARY

ABBREVIATION	EXPANSION
AA	Anti-androgen
ACE	Angiotensin-Converting Enzyme
ACTH	Adrenocorticotropic hormone
ADT	Androgen deprivation therapy
AE	Adverse Event
AR	Androgen receptor
AS	Activity Stage
AUC	Area under the plasma concentration–time curve
BID	Twice a day (bis in die)
BP	Blood pressure
BRCA2	BReast CAncer gene 2
BRG	Biological Research Group
BSA	Body surface area
CCI	Comparison Chief Investigator
CF	Consent Form
CI	Confidence interval
Co-CCI	Comparison Co-Chief Investigator
Cox-2	Cyclooxygenase 2
CRF	Case Report Form
CRN	Clinical Research Network
CRUK	Cancer Research UK
CRPC	Castrate-Resistant Prostate Cancer
CT	Computerised tomography
CTA	Clinical Trials Authorisation
CTAAC	Clinical Trials Advisory and Awards Committee
ctDNA	Circulating tumour DNA
CTC	Common Toxicity Criteria
CTU	Clinical Trials Unit
CTV	Clinical Tumour Volume
CVS	Cardiovascular
CXR	Chest X-ray
DAB	Dual Androgen Blockade

ABBREVIATION	EXPANSION
DHT	Dihydrotestosterone
DNA	Deoxyribonucleic Acid
DPA	Data Protection Act
ES	Efficacy Stage
IB	Investigator Brochure
ICH	International Conference on Harmonization
ECG	Electrocardiogram
FBC	Full Blood Count
FFS	Failure-Free Survival
FFPE	Formalin Fixed Paraffin Embedded
GCP	Good Clinical Practice
GFR	Glomerular Filtration Rate
GP	General Practitioner
HbA1c	Glycosylated haemoglobin
Hb	Haemoglobin
HE	Health Economics
HES	Hospital Episode Statistics
Hr	Hour
HR	Hazard Ratio
HRA	Health Research Authority
HSCIC	Health & Social Care Information Centre
HSPC	Hormone Sensitive Prostate Cancer
HT	Hormone Therapy
IDMC	Independent Data Monitoring Committee
IM	Intramuscular
IMRT	Intensity Modulated Radiation Therapy
INR	International Normalized Ratio
IR	Immediate-Release
ISRCTN	International Standard Randomised Controlled Trial Number
IU	International Units
IV	Intravenous
LFTs	Liver Function Tests
LHRH	Luteinising Hormone Releasing Hormone
LHRHa	Luteinising Hormone Releasing Hormone antagonist/agonist

ABBREVIATION	EXPANSION
LREC	Local Research Ethics Committee
m	Month
mcg	Microgram
MHRA	Medicine and Healthcare Products Regulatory Agency
min	Minutes
MRC	Medical Research Council
MREC	Main Research Ethics Committee
MRI	Magnetic resonance imaging
mTOR	Mammalian Target of Rapamycin
M0	Non-metastatic
M1	Metastatic
NCI	National Cancer Institute (USA)
NCRAS	National Cancer Registration and Analysis Service
NHS	National Health Service
N0	Node-negative
N+	Node-positive
NSAID	Non-Steroidal Anti-inflammatory Drugs
NYHA	New York Heart Association
OD	Once per day (omne in die)
ONS	Office for National Statistics
OS	Overall Survival
PATCH	Prostate Adenocarcinoma: TransCutaneous Hormones
PFS	Progression-free survival
PHE	Public Health England
PI	Principal Investigator
PIS	Patient Information Sheet
po	Orally (per orum)
PSA	Prostate Specific Antigen
pts	Patients
PTV	Planned Tumour Volume
QALY	Quality-adjusted Life Years
qds	Four times each day (quater die sumendus)
QL	Quality-of-life
RSI	Reference Safety Information
RTDS	National Radiotherapy Dataset

ABBREVIATION	EXPANSION
R&D	Research and Development
SACT	Systemic Anti-Cancer Therapy Dataset
SAE	Serious Adverse Event
SAR	Serious Adverse Reaction
sc	Under skin (sub-cutaneous)
SmPC	Summary of Product Characteristics
SOC	Standard-of-Care
SR	Sustained-Release
SSA	Site Specific Assessment
SUSAR	Suspected Unexpected Serious Adverse Reactions
SWOG	South West Oncology Group
tE2	Transdermal Oestradiol
TMG	Trial Management Group
TMT	Trial Management Team
TEAE	Treatment-emergent adverse event
TURP	Trans-Urethral Resection of Prostate
TSC	Trial Steering Committee
T2DM	Type 2 Diabetes Mellitus
UCL	University College London
ULN	Upper Limit of Normal
U+E	Urea and Electrolytes
WHO	World Health Organisation

TERM	DEFINITION
ADT	Androgen deprivation therapy given in the form of LHRH agonists/antagonists (abbreviated to LHRH) or alternatively, transdermal oestradiol.
Anti-androgens	Refers to 1st generation oral androgen receptor blockers including bicalutamide, flutamide and cyproterone. Please note that the use of cyproterone will impact on comparison-specific eligibility.
Comparison	In STAMPEDE many research treatments are evaluated and compared with participants receiving the current protocol standard-of-care. The term comparison describes the participants who have been randomised to receive research treatment and their comparable controls, each comparison is named by the research treatment e.g. the "metformin comparison" refers to all participants in arm K and the comparable non-diabetic patients contemporaneously randomised to arm A.
Hormone Therapy	Refers to all forms of hormone therapy given in the first line setting and includes LHRH, anti-androgens, transdermal oestradiol, GnRH agonists and antagonists. This term does not include novel AR-targeted agents such as abiraterone or enzalutamide.
PSA nadir	For trial purposes, this refers to the lowest PSA value detected between randomisation and week 24 on trial. This is used to derive the PSA progression value.
Protocol research treatment	Investigational Medicinal Products (IMPs) that are additional treatments participants allocated to research arms receive as part of the STAMPEDE protocol e.g. metformin for participants allocated to arm K, or alternative in the case of transdermal oestradiol for participants allocated to arm L.
Protocol standard-of-care (SOC) treatment	Standard forms of background treatment which are IMPs, permitted as part of the STAMPEDE protocol which include licenced ADT (e.g. LHRH analogues) given in the setting of hormone-naïve prostate cancer and first-line use of docetaxel, abiraterone, enzalutamide or apalutamide.
Non-protocol treatments	All prostate cancer treatments given following disease progression in the management of CRPC.
Prednisolone	In Swiss sites this may be referred to as prednisone.

1 LAY SUMMARY

STAMPEDE is a large clinical trial that aims to assess new treatment approaches for people affected by high-risk prostate cancer. The trial has been open since 2005 and has tested many different ways of treating prostate cancer and some results are now already known. Each new or alternative treatment is compared with the current standard approach, referred to as a “comparison”. More than 11,000 people have joined STAMPEDE so far with answers becoming available throughout the trial as information on life expectancy and disease control rates are gathered and compared.

New participants joining the trial from protocol v21.0 onwards may be eligible to join one of two treatment comparisons:

- The “metformin comparison” made between the control arm (arm A) who receive standard treatment only and the metformin treatment group (arm K) who receive standard treatment and metformin. **Note: randomisation is open to only a select number of sites participating in the metabolic substudy.**
- The “transdermal oestradiol comparison” made between the control arm (arm A) and the transdermal oestradiol treatment group (arm L) who receive transdermal oestradiol as an alternative form of standard hormone treatment.

Eligibility for each treatment group is dependent on several factors including the stage of prostate cancer, whether it has spread to involve other parts of the body (metastatic), and how long a patient has received hormone therapy prior to joining STAMPEDE. A computer program will be used to randomly allocate participants between all treatment groups for which they are eligible. **Table 3** summarises which treatment arms are currently open to recruitment.

Trial participants are asked whether they would like to join certain sub-studies being run alongside the trial. These aim to address several additional research questions such as what effect each treatment has on quality-of-life (QL), and which provides the greater value for money for the health service. Some sub-studies are focused on improving our understanding of the biology of prostate cancer. For example, can genetic changes be identified in prostate cancer cells that could predict which treatments might work best and may explain why some treatments stop working?

Table 3: Summary of treatment groups currently open to recruitment (Protocol version 21.0)

Treatment being tested	Treatment group	Summary	From protocol version
Metformin	Arm K	This anti-diabetic medication is proposed to have anti-cancer effects and may help prevent the side-effects of long-term ADT. STAMPEDE will investigate whether adding metformin to the current standard-of-care for non-diabetic people can improve life expectancy.<br>Please note from protocol v21 onwards only sites participating in the metabolic sub-study can recruit to Arm K.	15.0
Transdermal oestradiol	Arm L	This is a form of hormone treatment which can suppress testosterone as effectively as standard forms of androgen-deprivation therapy (ADT) and has been shown to avoid some of the side-effects. For example, treatment with transdermal oestradiol does not appear to cause the bone to thin, a common problem with standard forms of ADT which might lead to the bones becoming fragile (osteoporosis) and more likely to break. It may also help to avoid some of the side-effects and therefore improve overall quality of life compared with standard forms of ADT. STAMPEDE will investigate whether transdermal oestradiol can treat prostate cancer as well as current standard forms of ADT. Transdermal oestradiol is currently being tested in another large clinical trial called PATCH which already has over 1,400 men participating.	16.0

Further results are expected in the next few years from other treatments tested in STAMPEDE, which have completed recruitment, summarised in **Table 4.**

Table 4: Summary of treatment groups closed to recruitment; results awaited but follow-up ongoing

Treatment being tested	Treatment group	Summary	From protocol version
Abiraterone and Enzalutamide combination	Arm J	Enzalutamide is another novel hormone treatment, similar to abiraterone, which is also used in advanced prostate cancer, when standard hormone therapy has stopped working. Enzalutamide works by blocking androgen receptors and this may complement abiraterone. STAMPEDE is testing whether this treatment combination is a more effective way of controlling prostate cancer growth for longer and improving life expectancy.	12.0

Abiraterone was tested alone in arm G and the primary results of this comparison have been presented. Follow-up is ongoing as a further longer term analysis is planned.

Table 5: Summary of treatment group for which primary results reported but follow-up ongoing

TREATMENT TESTED	TREATMENT GROUP	SUMMARY OF RATIONALE AND RESULTS	PROTOCOL VERSION ADDED
Abiraterone	Arm G	This is a novel hormone treatment which works by inhibiting steroid hormone synthesis so blocks prostate cancer cells from generating their own male hormones. This is thought to be a major way in which prostate cancer cells resume growth following anti-hormonal therapies. The results of STAMPEDE have shown that the addition of abiraterone with prednisone improves life expectancy and disease control or relapse rates when used earlier, for people with locally-advanced or metastatic disease.	8.0
Prostate radiotherapy	Arm H	This is treatment with high-energy x-rays targeted to the prostate gland. This treatment is now mandatory within STAMPEDE for participants with cancer that is confined to the prostate gland as large trials have shown it improves life expectancy. The results from the primary analysis of the Arm H comparison demonstrate RT to the prostate prolonged overall survival in patients with oligometastatic (low burden) prostate cancer. Prostate RT did not provide any survival benefit to patients with high burden metastatic disease.	9.0

In the past STAMPEDE also tested whether adding docetaxel chemotherapy, zoledronic acid, or celecoxib, alone or in combination, and radiotherapy to prostate in M1 patients was beneficial in controlling prostate cancer growth and improving life expectancy. Recruitment has been completed to all of these original treatment groups, the results have been presented and it is no longer necessary to provide follow-up information relating to participants allocated to these comparisons, see **Table 6**.

Table 6: Summary of treatment groups reported and no longer on active follow-up

TREATMENT TESTED	TREATMENT GROUP	SUMMARY OF RATIONALE AND RESULTS	PROTOCOL VERSION ADDED
Zoledronic acid	Arm B	Prostate cancer cells can spread to bones and weaken them. Zoledronic acid is a drug that reduces bone destruction and hardens bones.<br>The results of STAMPEDE show that the addition of zoledronic acid alone does not prolong life expectancy. These results were comparable with data from other similar trials.	1.0
Docetaxel	Arm C	Docetaxel is a type of chemotherapy which can stop cells replicating. It has been used to treat advanced prostate cancer for some time, and is also used in e.g. the treatment of lung, breast and ovarian cancer.<br>The results of STAMPEDE show that the addition of docetaxel to hormone treatment does improve life expectancy, most markedly in people with metastatic disease, and delays time to progression or relapse for people with locally-advanced and metastatic disease.<br>The results of STAMPEDE were combined with other similar trials testing docetaxel and together, the results support this effect.<br>Docetaxel may now be given as part of standard treatment to all suitable people entering STAMPEDE (from protocol v14.0).	1.0
Celecoxib	Arm D	Celecoxib is an aspirin-like drug that is used to treat arthritis. It slows down the growth of cancer cells in the laboratory. STAMPEDE tested whether the addition of celecoxib could delay the growth of prostate cancer cells. Recruitment stopped early as an earlier analysis failed to demonstrate sufficient benefit. The final results were presented at GU ASCO 2016, a major international congress, and show that alone, celecoxib does not improve life expectancy.	1.0
Docetaxel and zoledronic acid combination	Arm E	The combination of these two medications did not offer any benefit to overall survival compared to the docetaxel alone.<br>Currently we do not recommend this combination as treatment for HSPC in STAMPEDE	1.0
Zoledronic and celecoxib	Arm F	The combination of these two medications did not improve overall survival in all patients randomised to this comparison. However, there was a small effect seen in patients with metastatic disease.<br>Currently we do not recommend this combination as treatment for HSPC in STAMPEDE	1.0

For further information relevant to these treatment groups, refer to the STAMPEDE website where you can see earlier versions of the protocol and find summaries of the results and links to the scientific publications, www.stampedetrial.org.

2 BACKGROUND

2.1 INTRODUCTION AND SETTING

Prostate cancer is a major health problem world-wide and accounts for nearly one fifth of all newly-diagnosed male cancers. In the UK, approximately 47,150 people were diagnosed with prostate cancer in 2015 and over 11,000 people died from the disease (5).

2.1.1 Long-term Androgen Deprivation Therapy

The initial (first-line) treatment for locally-advanced or metastatic prostate cancer is based on androgen deprivation therapy (ADT) achieved either surgically with bilateral orchidectomy, or medically with LHRH agonists or antagonists (6). Long-term use of oral anti-androgens is permitted only when given with LHRH agonists, to achieve dual androgen blockade (previously termed maximum androgen blockade - MAB).

When used alone ADT produces initial responses in up to 95% of patients but is rarely curative. STAMPEDE aims to improve outcomes for people affected by high-risk prostate cancer by testing if additional treatments added to ADT can improve disease control and life-expectancy. Data from the control arm in STAMPEDE has shown that for people with newly-diagnosed metastatic disease treated with ADT alone, the time to progression is just 11 months (6). Such progressive disease is referred to as castrate-resistant prostate cancer (CRPC).

Another important issue with ADT is the numerous associated side-effects, particularly with prolonged use. Since patients continue on LHRH after disease progression (with additional agents added), many people remain on treatment for a decade or longer. STAMPEDE is evaluating alternative forms of ADT and additional treatment with metformin aiming to mitigate some of the adverse effects of ADT which include osteoporosis (leading to an increased risk of fracture), adverse metabolic disturbance, cognitive decline, sexual dysfunction, hot flushes, physical deterioration and fatigue.

2.1.2 Role Of SOC Radiotherapy

Two randomised trials, SPCG7 (7) and NCIC PR.3 / MRC PR07 (8-10) have tested the question of whether ADT alone combined with radiotherapy is the best treatment for patients with high-risk localised prostate cancer (N0M0). Both trials demonstrated an improvement in overall and disease specific survival from the addition of radiotherapy to ADT. The size of this overall survival benefit is substantial (hazard ratio 0.68 in SPCG7 and 0.77 in PR07). As these two mature, large, well-conducted randomised trials have demonstrated benefit, we now mandate that radiotherapy be standard for patients with N0M0 disease (i.e. no nodal or metastatic spread). Patients with node-negative M0 prostate cancer will only be allowed to enter the trial if standard radiotherapy is planned. For patients with node-positive, M0 disease there are no randomised data on whether radiotherapy is indicated or not. However the NCIC PR.3 / MRC PR07 trial included patients with unknown nodal status who received whole pelvic radiotherapy (11) and demonstrated a large overall benefit. Additionally, non-randomised data from the STAMPEDE control arm suggests that the benefit observed in patients with N0M0 disease can be extended to those with pelvic nodal involvement. Therefore the STAMPEDE TMG recommends that pelvic nodal radiotherapy be considered for patients with node-positive, M0 disease at the discretion of the treating clinician (12).

More recently, data from the “M1:RT” arm showed that in patients with oligo-metastatic disease, RT to the prostate improved overall survival (4). Therefore, the STAMPEDE TMG recommends that

prostate RT is considered for patients with oligometastatic (low burden) disease at the discretion of the treating physician.

2.1.3 Role Of SOC systemic therapy: docetaxel, abiraterone, enzalutamide or apalutamide in addition to ADT

A variety of trials have demonstrated that addition of systemic therapy at the start of long term ADT prolongs survival, particularly in participants with metastatic disease. On the basis of the studies described below, the STAMPEDE TMG stongly recommends the clinician to consider either docetaxel, abiraterone, enzalutamide or apalutamide in all participants with metastatic disease at presentation who are commencing ADT for the first time and are fit enough to additional treatment. Choice of which systemic therapy to use is at the discretion of the clinician, but will need to be guided by availability of each treatment at site. We also suggest site investigators keep abreast of the latest published literature to inform choices between these treatments when options are available.

The primary analysis of the "original comparisons" has shown docetaxel significantly prolongs survival (HR 0.78; 95% CI 0.66-0.93)(1). This is in support of the results of the CHAARTED trial which showed docetaxel improved survival in people with metastatic disease (13, 14). There was no evidence of heterogeneity in STAMPEDE in the treatment effect across any patient subgroups and median survival was improved by 10 months, from 71 to 81 months. In a well powered and pre-planned sub-group analysis of people with metastatic disease at randomisation the treatment effect was most apparent with a median survival benefit of 15 months.

Data from the long term follow up of the docetaxel arm, specifically in men with non-metastatic disease, demonstrate that men who had RT to the prostate without chemotherapy had a superior FFS, PFS and a trend towards better OS, compared to men treated with both (15) . Therefore, although docetaxel is now permitted as part of the standard-of-care for all people entering STAMPEDE, we recommend patients with non-metastatic disease should be considered primarily for prostate RT, and chemotherapy considered only for those in whom RT is contra-indicated. Ultimately the decision is at the discretion of the treating clinician and patient.

The primary analysis of the “abiraterone comparison” has shown abiraterone improves survival in the HSPC setting (HR 0.63; 0.52 to 0.76; p<0.001)(3). The results are consistent with the co-published LATITUDE trial which recruited an overlapping subset (newly-diagnosed high-risk metastatic patients (16)) of the population eligible for STAMPEDE. A post-hoc subgroup analysis of the metastatic HSPC participants recruited to the STAMPEDE abiraterone comparison, suggest that benefit from abiraterone was irrespective of risk stratification via “risk” or “volume” measures (17).

The ENZAMET trial (18) demonstrated that enzalutamide used alongside ADT in the metastatic HSPC setting improved overall survival (HR 0.67; 0.52 to 0.86; P=0.002). Where available it is acceptable to use the addition of enzalutamide to ADT on the basis of evidence of benefit. Of note, there was no additional survival benefit seen in those patients treated with both docetaxel and enzalutamide in the upfront setting, whilst this combination resulted in higher rates of peripheral sensory neuropathy.

The TITAN trial (19) demonstrated that apalutamide used alongside ADT in the metastatic HSPC improved overall survival (HR 0.67; 0.51 to 0.89; p=0.005).

In the absence of data supporting a combination of treatment in the upfront setting, investigators are required to specify which upfront treatment will be used. It is **not** appropriate to use a **combination** of these treatments. Therefore, from protocol v21.0 onwards, SOC use of docetaxel**, or**

abiraterone, **or** enzalutamide, **or** apalutamide is permitted at the discretion of the treating clinician and patient. The choice of SOC treatment must be selected **prior** to randomisation.

Transdermal oestradiol has not previously been used alongside abiraterone, enzalutamide or apalutamide. Therefore the first cohort of participants recruited to STAMPEDE and started on this combination will have enhanced safety monitoring, with close monitoring of hormone levels. Any concerns that an interaction between these treatments is impacting efficacy will result in a pause to recruitment whilst this is investigated. A pre-planned review of early efficacy to achieve castration will be carried out once sufficient participants have been treated with these combinations. See Section 9.7.4 for further details.

2.2 DESIGN

STAMPEDE (also known as MRC PR08) is an innovative, multi-arm multi-stage (MAMS) randomised controlled trial open in multiple sites in the UK and Switzerland. The multi-arm design allows many treatment approaches to be tested simultaneously, and multi-stage refers to the pre-specified interim analyses that can be used to stop recruitment early to arms showing insufficient evidence of activity. The trial recruits people with high-risk locally advanced or metastatic prostate cancer, commencing long-term ADT for the first time.

The trial opened to recruitment in 2005 and initially assessed the effects of a bisphosphonate (zoledronic acid), a cytotoxic chemotherapeutic agent (docetaxel) and a cyclooxygenase (Cox-2) inhibitor (celecoxib), as single agents or combinations (arms B-F), referred to as the "original comparisons".

Since the start of the trial, a number of new research arms have been added to STAMPEDE to evaluate:

- Abiraterone, a steroid synthesis inhibitor (arm G)
- Prostate radiotherapy for patients with newly-diagnosed metastatic disease (arm H)
- Enzalutamide, an inhibitor of androgen receptor signalling, given with abiraterone (arm J)
- Metformin, a repurposed anti-diabetic medication (arm K)
- Transdermal oestradiol, a repurposed alternative form of ADT (arm L)

2.3 PREVIOUSLY-REPORTED RESEARCH TREATMENTS

Data have been reported on the "original comparisons" evaluating zoledronic acid, docetaxel, celecoxib and the combination of zoledronic acid with docetaxel or with celecoxib (1, 2, 20). As such, the rationale for these treatments, along with their design and details of treatment administration, are no longer covered within this version of the protocol.

The primary survival analysis of the "abiraterone comparison" has also now been reported although these participants remain on active follow-up as subsequent analyses of long-term follow-up are planned (3). The rationale can be found in previous protocol versions, however treatment information remains as it is relevant to participants who remain on abiraterone given alone (arm G) and in combination with enzalutamide (arm J).

The primary survival analysis of the "M1:RT" comparison testing RT to the primary tumour for men with newly diagnosed metastatic prostate cancer (Arm H) has now also been reported. These patients remain on active follow-up as subsequent analyses of long term follow-up planned. The treatment information is no longer covered within this version of the protocol as all treatment has now completed, however this can be accessed via previous versions of the protocol as below.

All previous versions of the protocol are available via www.stampedetrial.org, please refer to:

- Protocol version 11.0 and before for information relevant to “original comparisons” (Zoledronic acid, docetaxel, celecoxib)
- Protocol version 8.0 to 13.0 for information relevant to the “abiraterone comparison”
- Protocol version version 9.0 to 15.0 for information relevant to the “M1:RT comparison”

2.4 COMPARISONS IN FOLLOW-UP

The rationale for comparisons that have completed recruitment and remain in follow-up can be found in previous versions of the protocol. Recruitment was completed to the “enzalutamide and abiraterone comparison” in March 2016, as the recruitment target was reached. Participants remain on treatment therefore this information remains in this protocol version.

All previous versions of the protocol are available via www.stampedetrial.org, please refer to:

- Protocol version 16.0 or older for details relevant to “enzalutamide & abiraterone comparison”

2.5 RATIONALE FOR RECRUITING COMPARISONS

2.5.1 Metformin

All people joining STAMPEDE are planned for long-term ADT, a treatment associated with an increased risk of insulin resistance, hyperglycaemia, dyslipidaemia and obesity. Over 50% of people receiving long-term ADT will develop metabolic syndrome resulting in increased cardiovascular morbidity and mortality (21). Obesity and high bind insulin C-peptide levels, indicating insulin resistance are independent predictors of increased prostate cancer-specific mortality and the presence of metabolic syndrome and diabetes in people treated with ADT is associated with shorter survival.

Metformin, which in non-diabetic individuals has been shown to lower the incidence of diabetes, counteracts some of these side-effects of ADT, including insulin insensitivity, hyperinsulinaemia and diabetes. It also reduces the levels of cholesterol, LDLs and triglycerides by inhibiting the fatty acid synthesis via activation of Adenosine Monophosphate Activated Kinase (AMPK) and decreases the platelet aggregation factor 1, platelet aggregation, vascular adhesion molecules, CRP and leptin (22-25). Through mitigation of the cardiovascular and metabolic consequences of ADT, metformin is proposed to reduce treatment-associated morbidity and improve all-cause mortality.

In addition, recent data has emerged consolidating the knowledge that cancer progression is linked integrally with metabolic modulators and that modification of this process by metformin has an important effect on cancer progression and survival. Pre-clinical data has shown that metformin is an important stimulator of AMPK which acts as the cellular “master switch” for energy regulation. AMPK acts to inhibit the effects of elevated insulin levels which promote metastasis, tumour growth and treatment resistance. Insulin increases mRNA and protein expression of steroidogenic enzymes leading to the up-regulation of intracellular testosterone levels, secreted androgens, thereby activating the AR (26). Metformin also influences the PI3K-AKT pathway and has an anti-proliferative effect via inhibitor of mTOR as well as targeting cancer stem cells. In vitro, metformin has been shown to inhibit androgen-induced IGF-IR up-regulation through disruption of androgen signalling (27).

Evidence in support of this includes a systematic review and meta-analysis of 13,008 people with type 2 diabetes mellitus (T2DM) and concurrent cancer which has shown improved survival in people treated with metformin compared with other anti-diabetic agents. In a systematic review of

observational data from over 1 million people, there was a significant association seen between metformin and decreased risk of death from any cancer. Another systematic review found that the use of metformin in diabetic patients was associated with a significantly lower risk of cancer incidence and cancer mortality (28). In a large retrospective cohort study of 3837 diabetic people with prostate cancer, metformin was associated with a decreased risk of prostate cancer specific mortality (HR=0.76 [0.64-0.89]) and death (HR=0.76 [0.70-0.82]). In a prospective non-randomised phase II study in non-diabetic CRPC patients, 36% of patients receiving metformin were progression-free at 3 months and >50% had a prolongation of their PSA doubling time (29).

In summary, meformin is proposed to mitigate many of the adverse side-effects of long-term ADT as well as having multiple potential anti-cancer effects and therefore STAMPEDE will evaluate re-purposing this treatment as a novel therapeutic approach in the management of high risk locally-advanced or metastatic prostate cancer.

2.5.2 Transdermal Oestradiol

2.5.2.A Background & Rationale

ADT with LHRH analogue injections suppresses testosterone to castrate levels, but also depletes oestradiol, since around 80% of oestradiol in men is derived by aromatisation from testosterone. Thus men who are treated with LHRHa will have toxicities caused by low levels of both testosterone and oestrogen. The LHRH-associated toxicities which are due to low testosterone include loss of libido, erectile dysfunction and decrease in muscle mass. Other toxicities associated with LHRHa such as osteoporosis, increased fracture risk, hot flushes, memory loss, dyslipidemia and increased body fat deposition are thought to be due to oestradiol deficiency. In particular, the adverse effect of LHRHa on bone health has been well documented. Oestradiol deficiency prolongs the life-span of bone-resorptive osteoclasts, with the resulting imbalance between osteoclasts and bone-forming osteoblasts increasing the rate of bone thinning. This may lead to osteoporosis and increased risk of fracture, with the rate of fracture increasing with duration of LHRHa (30).

Transdermal oestradiol is a potential alternative to LHRHa that may avoid some treatment-related side-effects, therefore improving quality-of-life, which would be advantageous if shown to be equally effective at prolonging survival. Exogenous administration of oestradiol suppresses androgen production through a negative feedback loop involving the hypothalamic-pituitary axis, whilst avoiding the fall in oestradiol associated with castrate levels of testosterone (31). This, in turn, mitigates the toxicities of LHRH associated with oestradiol deficiency. Oral oestrogen was previously used for ADT before the development of LHRHa, but discontinued as first-line treatment due to increased thromboembolic toxicity, attributable to first-pass hepatic metabolism (32).

Parenteral administration (e.g. intravenous, intramuscular or transdermal oestradiol) avoids first-pass hepatic metabolism, mitigating the cardiovascular risk, as supported by results so far from the ongoing PATCH (Prostate Adenocarcinoma TransCutaneous Hormones [MRC PR09; ISRCTN70406718]) trial and previous studies evaluating parenteral oestradiol in the form of intramuscular polyestradiol phosphate (31, 33).

To date, there are a number of encouraging results from the PATCH trial demonstrating the safety and early activity of transdermal oestradiol compared to LHRH agonists in people with advanced hormone-naïve prostate cancer (see **Appendix I** for further details). In particular, similar rates of cardiovascular events have been observed in the transdermal oestradiol and LHRHa arms, as well as equivalent rates of testosterone suppression (based on around 900 patients enrolled up to Oct-2015) (31). Transdermal oestradiol has been shown to avoid the loss in bone mineral density associated with LHRHa, and results in improved metabolic profiles and quality-of-life compared to LHRHa (34). Furthermore, a pre-planned, confidential, interim analysis of the PATCH trial in Jun-2013 based on progression-free survival (PFS) led to the trial being extended to phase III; that analysis

included n=638 participants with 206 PFS events, reviewed against a pre-specified non-inferiority margin hazard ratio of 1.25 and 1-sided alpha 0.25. The phase III evaluation of clinical efficacy for transdermal oestradiol will be based on progression-free and overall survival as co-primary outcome measures.

Demonstrating that transdermal oestradiol is an equally effective approach to ADT would provide a globally important alternative (to LHRHa), with the potential to reduce treatment-associated morbidity and improve quality-of-life. In addition, there is a possibility that transdermal oestradiol may improve overall survival compared to standard hormone therapy. First, transdermal oestradiol may reduce treatment-associated morbidity and could potentially benefit overall survival. Second, up to 30% of people with castrate-resistant prostate cancer respond to oral oestrogen as post-relapse therapy, suggesting oestradiol may potentially have additional direct anti-tumour effects (35).

2.5.2.B Meta-Analysis With PATCH Trial

To further assess the clinical efficacy of transdermal oestradiol, the relevant data from the "transdermal oestradiol comparison" within STAMPEDE will be combined with data from patients recruited into PATCH, i.e. the "transdermal oestradiol comparison" within STAMPEDE is not sufficiently powered to form a stand-alone analysis. The evaluation of transdermal oestradiol will be based on a non-inferiority approach (in contrast to the other comparisons within STAMPEDE which are superiority questions), to test the hypothesis that transdermal oestradiol is at least as effective as standard hormone therapy, but with fewer side-effects.

Recruitment of patients to the "transdermal oestradiol comparison" through STAMPEDE enables the transdermal oestradiol research question to be answered more quickly than via PATCH alone. It also reduces the number of participants allocated standard treatment alone in both trials, thereby increasing the proportion of participants receiving a novel treatment approach and improving trial efficiency.

As of Feb-2017, nearly 1,200 participants had been recruited directly to the PATCH trial (also coordinated by MRC CTU at UCL) for the phase III evaluation of clinical efficacy of transdermal oestradiol. The overall recruitment target for the transdermal oestradiol evaluation is approximately 2,500 participants (including around 700 to be recruited through STAMPEDE).

3 SELECTION OF INSTITUTIONS AND INVESTIGATORS

Sites who wish to participate in STAMPEDE should be registered with the MRC CTU at UCL for this purpose. Before any participants are randomised, the CTU must receive a completed and signed Investigator Statement. The STAMPEDE Investigator Statement is signed by the Principal Investigator for that institution (download from http://www.stampedetrial.org/). The return of the Investigator Statement will be taken as confirmation of agreement to adhere to the trial protocol. In addition, a fully-signed model agreement is also required before recruitment can begin.

In compliance with the principles of GCP, all institutions participating in the trial will complete a delegation log and forward this to the CTU. Each person working on the STAMPEDE trial must sign off a section of this log indicating their responsibilities. The CTU must be notified of any changes to trial personnel and/or their responsibilities and an updated delegation log needs to be sent in to the CTU. An up-to-date copy of this log must be stored in the Investigator Site file at the institution and also at the CTU.

The Clinical Trial Authorisation (CTA) for the STAMPEDE trial requires that the Medicines and Healthcare Products Regulatory Agency (MHRA) be supplied with the names and addresses of all participating investigators/institutions. Trial staff at the CTU will perform this task; hence, it is vital to receive full contact details for all investigators prior to their entering participants.

Following substantial amendments and new comparisons opening, sites will be notified of relevant documents and training required and if and when they are able to participate. Further accreditation packs may be circulated as a result to update trial documentation.

3.1 SITE/INVESTIGATOR CRITERIA

3.1.1 Principle Investigator's Qualifications & Agreements

1. The investigator(s) should be qualified by education, training, and experience to assume responsibility for the proper conduct of the trial at their site and should provide evidence of such qualifications through an up-to-date curriculum vitae and/or other relevant documentation requested by the Sponsor, the REC, the IRB, and/or the regulatory authorities.

2. The investigator must hold a long term contract with their site. Locum members of staff cannot fill the role of Principle Investigator (PI).

3. The investigator should be thoroughly familiar with the appropriate use of the investigational products, as described in the protocol, current Investigator Brochure or Summary of Product Characteristics and in other information sources provided by the Sponsor.

4. The investigator should be aware of, and should comply with, the principles of GCP and the applicable regulatory requirements. A record of GCP training should be accessible for all investigators.

5. The investigator/site should permit monitoring and auditing by the Sponsor, and inspection by the appropriate regulatory authorities.

6. The investigator should maintain a delegation log of appropriately-qualified persons to whom the investigator has delegated significant trial-related duties.

7. The investigator should sign an investigator statement, which verifies that the site is willing and able to comply with the requirements of the trial.

3.1.2 Adequate Resources

1. The investigator should be able to demonstrate a potential for recruiting the required number of suitable subjects within the agreed recruitment period (that is, the investigator regularly treats the target population).

2. The investigator should have sufficient time to properly conduct and complete the trial within the agreed trial period.

3. The investigator should have available an adequate number of qualified staff and adequate facilities for the foreseen duration of the trial to conduct the trial properly and safely. In addition, the investigator should arrange for suitably qualified investigator cover for safety reporting in the event of their absence.

4. The investigator should ensure that all persons assisting with the trial are adequately informed about the protocol, the investigational products, and their trial-related duties and functions.

5. The site should have sufficient data management resources to allow prompt data return to the CTU.

3.2 COMPARISON-SPECIFIC SITE ACCREDITATION

3.2.1 Transdermal Oestradiol Comparison: arms A & L

Only UK sites participating in STAMPEDE will be accredited for the “transdermal oestradiol comparison”.

3.3 REQUIRED TRIAL DOCUMENTATION

Table 7 presents a summary of the required trial documentation for participating sites. Templates are provided on the STAMPEDE website, www.stampedetrial.org.

Table 7: Trial documentation required for participating sites

TRIAL DOCUMENTATION	TIMING
Confirmation of capacity and capability (including IRMER approval)	Before site participation
Signed Investigator Statement	Before site participation
Signature list & delegation of responsibilities	Before site participation
Trial personnel contact details	Before site participation
Participant information sheets (PIS), GP Letter & Informed consent form (ICF) on local paper	Before site participation
Signed Clinical Trial Agreement between Trust and Sponsor (or Variation if applicable)	Before site participation
Site initiation training	Before site participation
Signed Pharmacy Pack acknowledgment	Before site participation

4 SELECTION OF PARTICIPANTS

4.1 IDENTIFYING POTENTIAL TRIAL PARTICIPANTS

STAMPEDE recruits participants with high-risk prostate cancer who are commencing long-term androgen-deprivation therapy (defined as at least 2 years) for the first time. All participants must fulfil one of the following **broad disease categories:**

- High-risk newly-diagnosed non-metastatic, node-negative disease
- Newly-diagnosed metastatic or node-positive disease
- Previously radically treated, now relapsing with high risk features

See **Section 4.4** for detailed category inclusion criteria

4.2 APPROACH TO INFORMED CONSENT

Potential participants should be provided with information about STAMPEDE at the earliest opportunity to allow sufficient time to consider their participation and complete the required screening procedures to determine comparison-specific eligibility.

Informed consent is an ongoing process and participants must be made aware that refusal to take part in all or any aspect of the trial at any time for any reason is permitted, without incurring any consequence or impact on their standard treatment. All aspects of the trial e.g. sub-studies should be presented and optional participation discussed, and investigators are encouraged to adopt a staged approach where possible to avoid information overload.

Original signed consent forms must be stored in the site investigator file, a copy stored in the patient's medical notes (electronic/paper), and a copy provided to the participant. For central monitoring purposes an anonymised copy must also be sent to the CTU, refer to **Section 10.1.1**.

4.2.1 Screening Investigations Prior To Randomisation

All participants must have the following examinations performed to confirm eligibility prior to randomisation. Please note, all screening investigations should be recent such that they reflect the participant's current disease status.

The following imaging is always required within 6 months (184 days) prior to randomisation:

- Cross-sectional imaging (CT, MRI, PSMA-PET-CT or Choline-PET-CT) of pelvis and abdomen, SPECT-CT is not sufficient
- AND Bone Scan (or equivalent e.g. whole body MRI, or SPECT-CT)
- AND Chest X-ray (only if chest was not included in cross sectional imaging i.e. CT, Choline-PET-CT or PSMA-CT-PET which would be preferable; MRI imaging of chest is not sufficient on its own)

Please note, for trial purposes M1 disease will be defined using internationally agreed criteria, therefore M1 staging cannot be based solely on PET avid lesions. To be considered M1, the metastatic lesion must also be visible on standard imaging i.e. CT (can be CT component of PET-CT) or bone scan.

The following blood tests are required within **6 months (184 days)** prior to randomisation:

- Pre-hormone treatment PSA

- Pre-hormone treatment Testosterone (if available)

The following bloods and additional measurements are required within **4 weeks (28 days)** prior to randomisation:

- Haematology: Full blood count*
- Biochemistry: Liver function tests, serum creatinine
- Systolic and diastolic blood pressure
- Waist circumference measurement
- Weight and height

The following bloods and additional measurements are required within **2 weeks (14 days)** prior to randomisation:

- Baseline PSA

* If a participant has started SOC docetaxel please use a full blood count measured shortly prior to chemotherapy. This means in the setting of SOC docetaxel these results provided should be from within the last 16 weeks (112 days) prior to randomisation. This will ensure an appropriate baseline is reviewed to confirm fitness for treatment and eligibility for participants. For all other participants the blood count used should be taken within 4 weeks prior to randomisation.

Participants who initially fail to meet the trial eligibility criteria can be re-screened at a later date if timelines permit.

4.2.2 Baseline Investigations required for participants allocated to arms A, K, L

The following blood tests and additional measurements are required at baseline **within 4 weeks (28 days)** prior to randomisation:

- HbA1c (for participants being considered for metformin comparison)
- Glucose and triglycerides (preferably fasting for metabolic analysis, but if only able to obtain a non-fasting result please record on randomisation CRF)
- Lipid profile (fasting or non-fasting; total cholesterol, LDL and HDL)

See **Table 2** for a detailed schedule of assessments for all participants randomised to arms A, K or L.

We encourage site investigators to carry out any additional investigations they feel are necessary in particular cases to ensure that participants are appropriately fit to be randomised in the STAMPEDE trial.

4.3 PRIOR PERMITTED SOC TREATMENTS

4.3.1 Hormone Treatment Prior To Randomisation

From protocol v16.0, participants can potentially be randomised to the “transdermal oestradiol comparison” and it would be preferable for these participants to have had as little exposure to ADT as possible.

Within the separate PATCH trial, participants are randomised within 8 weeks after starting anti-androgens and cannot have received an LHRHa injection. This approach is also favoured in STAMPEDE, but participants who have received a single 4-week (or 1-month) LHRHa injection remain eligible, as shown in **Table 8.**

Anti-androgen monotherapy is not permitted as a form of long-term hormone therapy but initial use is encouraged to meet the eligibility criteria for the “transdermal oestradiol comparison”. Anti-

androgens may include flutamide or bicalutamide, however use of cyproterone will mean the participant is ineligible for arm L (36).

Table 8: Maximum prior hormone therapy

TIME CONSIDERATIONS	PRIOR ANTI-ANDROGENS	PRIOR LHRH	PRIOR SOC ABIRATERONE, ENZALUTAMIDE OR APALUTAMIDE	ELIGIBLE FOR INCLUSION TO
Maximum duration – all arms except Arm L	14 weeks	12 weeks	12 weeks	A:K
Arm L	≤8 weeks	≤4 weeks	Nil	A:L

Permitted prior hormone therapy for now-relapsing disease: Any patients now presenting with relapsed disease, previously treated with adjuvant or neo-adjuvant hormone therapy alongside their radical surgery or radiotherapy, must have completed that period of hormone therapy **at least 12 months** before joining STAMPEDE and it must have been **no longer than 12 months in duration**.

4.3.2 Standard-Of-Care (SOC) Radiotherapy

In participants with N0M0, N+M0 and oligometastatic disease (as per M1RT definition (4), see Section 6.1.3), the treating clinician and participant must have decided, **prior** to randomisation, whether prostate radiotherapy will be given as part of SOC.

4.3.3 Standard-Of-Care (SOC) Systemic Therapy; docetaxel, abiraterone, enzalutamide or apalutamide

The treating clinician and participant must have decided, **prior** to randomisation, whether docetaxel, abiraterone, enzalutamide or apalutamide is to be given as part of SOC.

Please note that only one SOC treatment can be selected at randomisation. See Section 6.1.4 for treatment details.

Investigators should aim to start SOC docetaxel treatment within 12 weeks after starting ADT, consistent with the timelines achieved for research arm C. Participants may start docetaxel treatment prior to randomisation. See Section 6.1.3 for treatment details.

If SOC docetaxel treatment was not commenced prior to randomisation and participants are subsequently allocated to receive transdermal oestradiol (Arm L), it is recommended that docetaxel treatment commences **after** participants have been established on transdermal oestradiol for around 4 weeks, when most participants are likely to have completed the induction period (see Section 6.2.5.A).

Participants may start abiraterone, enzalutamide or apalutamide prior to randomisation. However the use of these treatments prior to randomisation will impact comparison-specific eligibility. At present participants who have **already started** these treatments will only be eligible for the "metformin comparison". Patients **planned** for these treatments can be considered for both metformin and transdermal oestradiol comparisons.

At present, there are no safety data available on the use of abiraterone, enzalutamide or apalutamide in combination with transdermal oestradiol. The initial cohort of participants randomised to receive transdermal oestradiol and planned for SOC abiraterone, enzalutamide or apalutamide will be subject to additional CTU review to monitor these combinations, and an additional early pre-planned analysis of safety.

When complete, the findings will be reviewed by the relevant committees and sites will be advised as to whether these combinations can continue. If found to be safe in the upfront setting then we will also there-after permit the use of the medications alongside transdermal oestradiol patches in the CRPC setting.

4.4 GENERAL INCLUSION CRITERIA – DISEASE CATEGORIES

Participants must fulfil all the criteria in one of the following three categories:

4.4.1 High-Risk Newly-Diagnosed Non-Metastatic Node-Negative (N0/Nx) Disease

Both:

- At least two of: T category T3/4, PSA≥40ng/ml or Gleason sum score 8-10
- Intention to treat with radical radiotherapy (unless there is a contra-indication)

OR

4.4.2 Newly-Diagnosed Metastatic Or Node-Positive Disease

At least one of:

- Stage T_{any} N+ M0
- Stage T_{any} N_{any} M+

OR

4.4.3 Previously Radically Treated, Now Relapsing (Prior Radical Surgery And/or Radiotherapy)

At least one of:

- PSA ≥4ng/ml and rising with doubling time less than 6 months
- PSA ≥20ng/ml
- N+
- M+

AND

4.5 GENERAL INCLUSION CRITERIA REQUIRED FOR ALL PARTICIPANTS

I. Histologically confirmed prostate adenocarcinoma
II. Intention to treat with long-term androgen deprivation therapy
III. Fit for all protocol treatment[1] and follow-up, WHO performance status 0-2[2]
IV. Have completed the appropriate investigations prior to randomisation
V. Adequate haematological function: neutrophil count $\geq 1.5 \times 10^9$/l and platelets $\geq 100 \times 10^9$/l
VI. Adequate renal function, defined as GFR ≥30ml/min/$1.73m^2$
VII. Written informed consent
VIII. Willing and expected to comply with follow-up schedule
IX. Using effective contraceptive method if applicable

[1] Medical contraindications to the trial medications are given in **Section 6**
[2] For WHO performance status definitions see **Appendix A**

4.6 GENERAL EXCLUSION CRITERIA

I. Prior systemic therapy for locally-advanced or metastatic prostate cancer (except as listed in **Section 4.3**[1])

II. Prior exposure to hormone therapy for a duration of > 12 months, or prior exposure completing < 12 months before randomisation (see **Section 4.3.1** for permitted prior exposure details)

III. Metastatic brain disease or leptomeningeal disease

IV. Abnormal liver functions consisting of any of the following:
   - Serum bilirubin ≥1.5 x ULN (except for participants with Gilbert's disease, for whom the upper limit of serum bilirubin is 51.3µmol/l or 3mg/dl)
   - Aspartate aminotransferase (AST) or alanine aminotransferase (ALT) ≥2.5 x ULN - site must indicate at randomisation whether one or both tests are performed at site. Where both results are available, both must confirm eligibility.

V. Any other previous or current malignant disease which, in the judgement of the responsible clinician, is likely to interfere with STAMPEDE treatment or assessment

VI. Any surgical wound (e.g. TURP) which in the judgement of the responsible clinician may interfere with or be exacerbated by protocol treatment

VII. Participants with significant cardiovascular disease, including:

- Severe/unstable angina
- Myocardial infarction less than 6 months prior to randomisation
- Arterial thrombotic events less than 6 months prior to randomisation
- Clinically significant cardiac failure requiring treatment, defined as New York Heart Association (NYHA) class II or above[2]
- Cerebrovascular disease (e.g. stroke or transient ischaemic episode) less than 6 months prior to randomisation
- Any other significant cardiovascular disease that in the investigator's opinion means the participant is unfit for any of the study treatments.

[1] Details timelines for recently initiated SOC docetaxel, abiraterone, enzalutamide, apalutamide

[2] NYHA classifications can be found in Appendix A

4.7 COMPARISON-SPECIFIC ELIGIBILITY CRITERIA

In addition to the general inclusion and exclusion criteria, the following comparison-specific eligibility criteria apply.

4.7.1 Metformin Comparison (randomisation between arm A and arm K)

Please note from protocol v21 only patients willing to participate in the metabolic sub study should be randomised to the metformin comparison. The sub study will be conducted in a limited number of sites, see section 4.7.4 for further information.

In addition to the general inclusion and general exclusion criteria the following comparison-specific inclusion criteria must be met to be eligible for randomisation to the "metformin comparison":

- Hb A1c <48mmol/mol (equivalent to <6.5%)
- Adequate renal function, defined as GFR ≥45ml/min/1.73m^2 (except for Switzerland[1])
- No history of lactic acidosis or predisposing conditions
- No current or previous treatment with metformin
- No current or previous medication for treatment of diabetes
- No contraindications to metformin
- Willingness to join the metabolic sub study

The method used to determine glomerular filtration rate may vary according to local practice. Equations that either estimate glomerular filtration rate (eGFR) or creatinine clearance (CrCl) may be used and the same threshold value applies. Where possible, HbA1c should be performed prior to commencing SOC docetaxel to reduce the likelihood of corticosteroid-related hyperglycaemia impacting on eligibility. All participants with abnormal baseline HbA1c (i.e. 6.5% or higher) should be informed and referred to their GP for further management.

4.7.2 Transdermal Oestradiol Comparison (randomisation between arm A and arm L)

In addition to the general inclusion and exclusion criteria, participants fulfilling all of the following are eligible for the “transdermal oestradiol comparison”:

- ≤8 weeks of anti-androgen (AR-antagonists) use
- Maximum of 1 dose of monthly or 4-weekly LHRH agonist/antagonist
- No prior LHRH agonist injection with a stated duration of effect greater than 1 month
- ≤12 weeks since first dose of any hormone therapy
- Not had a bilateral orchidectomy
- No use of cyproterone acetate prior to randomisation
- No known porphyria
- No history of radiologically confirmed deep vein thrombosis or pulmonary embolism
- No known thrombophilic disorder (e.g. Protein C, Protein S, antithrombin deficiency)
- Not yet started SOC abiraterone, enzalutamide or apalutamide (see Section 4.3.4 for information)

[1] Switzerland sites - please refer to SAKK appendix for local guidance

4.8 SUB-STUDY ELIGIBILITY CRITERIA

There are currently four sub-studies that aim to further the understanding of the biology of prostate cancer through additional genetic analyses and correlation with clinical data. For details on each sub-study, see **Section 17.2.**

4.8.1 Eligibility for germline DNA sub-study (Saliva samples)

All newly randomised trial participants who join arms A, K or L are asked to provide a saliva sample from which germline (inherited) DNA can be extracted.

Participants randomised from **15-Nov-2011** onwards who consented to provide a blood spot (Consent Form version 4.0 part K) can also be retrospectively approached to provide a saliva sample providing they have received the REC-approved letter explaining the need for additional saliva sample collection as the DNA extraction using the blood spot method did not work as well as anticipated.

For further information please refer to the **Sample collection and handling manual**.

4.8.2 Eligibility for the circulating tumour DNA sub-study (sequential blood samples)

This substudy is not recruiting currently. For details relating to blood sample collection for patients already participating in the substudy, including eligibility criteria and shipping refer to the **Sample collection and handling manual.**

4.8.3 Eligibility for tumour sample analysis (FFPE blocks)

All newly randomised trial participants should be provided with the STAMPEDE Additional Research Participant Information Sheet in order to consider optional donation of remaining diagnostic prostate cancer tissue stored as formalin fixed paraffin embedded (FFPE) blocks.

The criteria for enrolment into the FFPE block collection:

- Newly randomised STAMPEDE participants
- Informed consent to gift remaining tissue to be used for additional research analyses

Tumour blocks will only need to be sent for a select subset of STAMPEDE patients. For more details, please refer to the **Sample collection and handling manual.**

4.8.4 Eligibility for metformin metabolic sub-study

All newly randomised trial participants who meet the eligibility criteria to join the A/K comparison are eligible to join the metformin metabolic sub-study, if the site where they are being treated is participating in this sub-study. A limited number of sites will be recruiting for this sub-study. Selected sites involved have volunteered and demonstrated they have sufficient resources to undertake the metabolic sub-study.

Participants who are eligible for randomisation to the metformin comparison must be willing to take part in the metabolic sub-study and be able to adhere to the blood sample schedule. Appropriate consent to the additional blood samples must be provided.

5 RANDOMISATION AND ENROLMENT

5.1 RANDOMISATION

Participant eligibility will be confirmed during the randomisation process and participants will be allocated to any of the open research comparisons for which they are eligible (see **Section 4.6**). To randomise a participant please carefully complete the Randomisation CRF and then contact the CTU.

RANDOMISATION
Call MRC CTU at UCL, Monday to Friday 0900-1700
Excluding public holidays or dates when notice has been given by the CTU.
Tel: +44 (0) 20 7670 4777

A trial ID and treatment will be allocated and given over the phone and by email confirmation. In addition, a letter confirming these details will be sent. The trial ID will be the primary way in which the participant will be identified and should be used in all correspondence. Sites should send a letter to the participant's GP to inform them of their trial participation and treatment allocation. The GP letter is supplied as a template and can be downloaded from the trial website www.stampedetrial.org.

The randomisation CRF, eligibility checklist and anonymised consent form* must be submitted to the CTU following randomisation.

**Anonymised consent forms are not required to be submitted for Swiss participants.*

5.2 CO-ENROLMENT GUIDELINES

Interventional clinical trials
STAMPEDE participants should not join any other interventional clinical trials of prostate cancer treatment until the following criteria have been met:

- The participant has experienced at least one failure-free survival (FFS) event
- The participant is no longer on any STAMPEDE research treatment that is permitted to continue post first progression e.g. metformin, abiraterone or enzalutamide

Once both criteria are satisfied the participant may be entered into further treatment studies evaluating treatments for CRPC.

Site investigators should check with the CTU prior to participants commencing any IMP within an **interventional clinical trial** for any other medical condition, such as a new malignancy, to ensure there are no concerns about interactions with STAMPEDE treatments. Note that STAMPEDE treatment can be continued alongside **non-trial treatments** for a new malignancy providing local pharmacy review to ensure there are no interactions.

The primary outcome measure of STAMPEDE is overall survival, therefore follow-up must continue after co-enrolment (unless the participant withdraws consent). Participation in interventional studies must be reported to CTU on the Co-enrolment CRF. Details of any interventional treatments received post-progression in such studies must be reported on the Additional Treatment Log.

Non-interventional clinical trials

Co-enrolment in non-interventional studies for any indication is permitted at any time providing that it does not interfere with treatment or assessment in STAMPEDE. This does **not** require reporting using the Co-enrolment CRF which captures details of interventional prostate cancer clinical trials only.

Data sharing agreements with “downstream” trials are encouraged to improve data quality in both trials and to reduce costs to both organisations.

6 TREATMENT OF PARTICIPANTS

6.1 STANDARD-OF-CARE (SOC)

The SOC for this patient group is **androgen deprivation therapy** (ADT) as per local practice (see Section 6.1.1). For some participant groups, this should now be supplemented with SOC radiotherapy (see Section 6.1.2). From protocol v14.0 onwards, SOC docetaxel is permitted for all suitable participants. From protocol v19.0 onwards, SOC abiraterone is also be permited as an alternative to docetaxel, where this is available. From protocol v21.0 onwards SOC enzalutamide and apalutamide are also permitted as an alternative to docetaxel or abiraterone, where these are available, (see Section 6.1.4).

SOC combinations	Metformin comparison	Transdermal oestradiol comparison
ADT alone	Yes	Yes
ADT + prostate RT +/- nodal RT	Yes	Yes
ADT + docetaxel	Yes	Yes
ADT + docetaxel + RT	Yes	Yes
ADT + abiraterone/enzalutamide/apalutamide	Yes	Yes*
ADT + abiraterone/enzalutamide/apalutamide + RT	Yes	Yes*

*See section 7.1.5.C for guidelines for submitting hormone treatment logs – please send as quickly as possible to facilitate safety monitoring of combination.

6.1.1 Androgen Deprivation Therapy

The planned duration of ADT should be **at least 2 years** and lifelong in those with metastatic disease. With the exception of those allocated to transdermal oestradiol (Arm L), all participants will receive ADT as per local practice to achieve castrate levels of testosterone. The method of planned or current long-term standard-of-care ADT must be specified prior to randomisation. See below for the permitted methods of ADT and see Section 4.3.1 for more information on ADT timing before randomisation. Participants allocated to Arm L will go on to receive transdermal oestradiol in place of standard ADT methods.

6.1.1.A Bilateral Orchidectomy

Operations should be performed by appropriately trained surgeons. A total or sub-capsular orchidectomy may be performed. Participants having a bilateral orchidectomy are required to adhere to the same timelines for prior LHRH and/or anti-adrogen exposure as specified in Section 4.3.1. Note, bilateral orchidectomy is an exclusion criteria for the “transdermal oestradiol” comparison, see section 4.6.2.

6.1.1.B LHRH Agonists e.g. goserelin, leuprorelin

LHRH agonists used according to local practice. The prophylactic use of anti-androgens to prevent tumour “flare” is recommended.

6.1.1.C LHRH Antagonists e.g. degarelix

LHRH antagonists used according to local practice. The use of prophylactic use of anti-androgens to prevent tumour “flare” is not necessary.

6.1.1.D Dual Androgen Blockade

Long-term use of anti-androgens alongside LHRH agonists, according to local practice. Note this was previously referred to as maximum androgen blockade. Anti-androgen monotherapy is not deemed an androgen deprivation regimen.

6.1.2 SOC Radiotherapy to prostate

6.1.2.A N0M0 Participants

Investigators should give standard RT to participants with node-negative, non-metastatic disease (N0M0), in accordance with data from the PR07 and SPCG trials (7, 10). If RT is contra-indicated this must be recorded on the Randomisation CRF. See **Section 6.1.3** for further details of RT administration.

6.1.2.B N+M0 Participants

For participants with node-positive, M0 disease there are no randomised data on whether radiotherapy is indicated or not. However the NCIC PR.3 / MRC PR07 trial included participants with unknown nodal status who received whole pelvic RT (11) and demonstrated a large overall benefit. Additionally, non-randomised data from the STAMPEDE control arm (Arm A) suggests that the benefit observed in participants with N0M0 disease can be extended to those with pelvic nodal involvement. Therefore, the STAMPEDE TMG recommends that pelvic nodal RT be considered for participants with node-positive, M0 disease at the discretion of the treating clinician (12).

6.1.2.C Oligometastatic Participants

For participants with oligometastatic disease, data from the M1:RT arm supports the use of RT to the prostate (4). Currently, data strongly supports the use of prostate RT in men with up to 3 bone metastases and/or lymph node only disease, however we are aware that ongoing analyses may redefine which patients benefit from this treatment. Therefore, the STAMPEDE TMG recommends that prostate +/- pelvic nodal RT be considered for participants with oligometastatic disease, with the treating clinician to determine whether the participant has oligometastatic disease that they deem likely to benefit from this treatment.

6.1.2.D Planned Use Of SOC RT

Suitability for RT is assessed by the treating clinicians. Investigators will be asked to state their intention with regards to planned RT in this group at randomisation. Intention to give RT (or not) for **all** participants must be stated at randomisation to ensure that there is no bias towards particular combinations of systemic therapy with RT.

SOC RT administration is not being investigated as part of the trial, therefore only minimal data about SOC RT will be collected. It is accepted that some participants will develop progressive disease before RT can be administered and if this occurs the reasons for non-delivery of treatment must be recorded on the Radiotherapy Detail CRF.

6.1.3 Administration of SOC RT

Standard radiotherapy will be given to appropriate participants in each of the trial arms, following a period of neo-adjuvant ADT therapy, as is generally standard in UK practice. For participants with negative nodes on axial imaging, clinicians may choose between irradiating prostate and seminal vesicles alone or including the pelvic nodes in addition. Additional staging tests such as pelvic node sampling may be considered in making this decision. Conformal or intensity modulated radiotherapy should be used in all participants. Where participants have good clinical evidence that nodes are free of tumour or participants for whom nodal radiotherapy is contra-indicated (e.g. significant bowel disease), treatment may be given to the prostate gland and seminal vesicles only. The recommended dose is 74Gy in 37 fractions to the prostate and seminal vesicles or the equivalent using hypo-

fractionated schedule, 60Gy in 20 fractions. Alternative dosing schedules are permitted but must be agreed with the STAMPEDE Trial Management Group (TMG).

6.1.3.A Standard-Of-Care RT Timing

If receiving docetaxel as part of the standard-of-care (permitted from protocol v14.0), the participant must have sufficiently recovered from any docetaxel toxicity before RT can begin. In all other participants not receiving SOC docetaxel, SOC RT may be started sooner (2-6 months post-randomisation) consistent with the data from the MRC PR07 trial (11).

6.1.4 SOC upfront systemic therapy: docetaxel, abiraterone, enzalutamide or apalutamide

Docetaxel, abiraterone, enzalutamide or apalutamide may be considered for use as SOC treatments, provided the treatment is available locally. Choice of which systemic therapy to use is at the discretion of the clinician. However, we suggest site investigators keep abreast of the emerging literature to inform choices between these treatments, when options are available.

From protocol v14.0 investigators may consider giving docetaxel as part of the SOC for participants with newly-diagnosed metastatic disease, based on the survival benefit demonstrated by both STAMPEDE in the primary analysis of the "original comparisons" and CHAARTED (13) (14, 20). Investigators may also consider giving docetaxel to participants with high-risk locally-advanced disease.

From protocol v19.0 onwards, the treating clinician and participant may consider the use of abiraterone in the newly diagnosed setting, where this is available.

From protocol v21.0 onwards the treating clinician and participant can also consider the alternate options to use enzalutamide or apalutamide in the newly diagnosed setting, if available.

The treating clinician and participant must have decided **prior** to randomisation if SOC docetaxel, abiraterone, enzalutamide or apalutamide is to be given to ensure use is balanced between control and treatment arms. Treatment with SOC systemic therapy may start prior to randomisation, except in Arm L when abiraterone, enzalutamide or apalutamide cannot have started prior to starting trial treatment. In addition, for participants allocated to Arm L who have not already started SOC docetaxel prior to randomisation, it is recommended that docetaxel commences around 4 weeks after starting research treatment (see Section 6.2.5).

In the absence of data supporting the routine use of concurrent or sequential use in the absence of disease progression, investigators are required to specify which SOC treatment will be used (i.e.: **one** of docetaxel, abiraterone, enzalutamide or apalutamide) and may **not** plan to use a combination of these. In the case that SOC treatment is changed from one agent to another in order to manage toxicity/intolerance (as per current national guidelines) please update the SOC Systemic Treatment CRF.

We recommend starting SOC systemic therapy within 12 weeks of initiation of ADT. This timing is consistent with the time-scale for starting treatment within the aforementioned clinical trials.

Docetaxel is given according to local protocols as a standard non-trial treatment. The regime used previously within STAMPEDE (Arm C and Arm E) was 75mg/m^2 Day 1 as 1hr IV infusion, plus prednisolone 5mg BID for 21 days repeated every 3 weeks for a maximum of 6 cycles. GCSF use is at the investigator’s discretion; prednisolone may be omitted.

Abiraterone, enzalutamide or apalutamide should be given according to local protocols as a standard non-trial treatment. Currently abiraterone, enzalutamide and apalutamide are funded differently by the NHS dependent on burden of disease and which country the participant is being treated in; follow national guidelines for duration and management of therapy once available.

The dosing, safety monitoring and toxicity management contained within the STAMPEDE protocol refers to research abiraterone and enzalutamide given to participants previously allocated to Arms G or J, but may be used as a guide if required. The protocol guidelines for abiraterone were based on the recommendations from the manufacturers, which are included in the summary of product characteristics and can be found online. Similarly, for dosing, safety monitoring and toxicity management of enzalutamide and apalutamide consider referring to the summary of product characteristics produced by the manufacturers, and any local or national guidelines available.

A SOC Systemic Treatment CRF should be completed for all participants randomised to STAMPEDE regardless of whether any SOC systemic therapy was planned. See **Section 7.2.3** for details of data collection for SOC Systemic Treatment.

6.2 RESEARCH TREATMENTS

Research treatment durations are outlined below with a separate section for each individual Investigational Medicinal Product (IMP) detailing the cautions and contraindications, interactions, safety monitoring and toxicity clinical management.

6.2.1 Treatment Duration

Table 9: Intended treatment duration – All arms

RANDOMISED ARM AND TREATMENT	TREATMENT DURATION IS DEPENDENT ON DISEASE STATE		
	M0 WITH PLANNED RADICAL TREATMENT[1]	M0 WITH NO PLANNED RADICAL TREATMENT[1]	M1
Arm A:SOC androgen deprivation therapy	Minimum 2 years	Continue lifelong	Continue lifelong
Arm G: Abiraterone	2 years - *unless progression occurs before (see* **Table 10***)*	Continue until all categories of disease progression have occurred (see **Table 10**)	Continue until all categories of disease progression have occurred (see **Table 10**)
Arm J: Abiraterone and enzalutamide	2 years - *unless progression occurs before (see* **Table 10***)*	Continue until all categories of disease progression have occurred (see **Table 10**)	Continue until all categories of disease progression have occurred (see **Table 10**)
Arm K: Metformin	Minimum 3 years (continue for 12 months after the last injection of LHRHa to allow for the delay in testosterone levels returning to normal)	Continue lifelong as long as the investigator feels it is in the best interests of the participant.	Continue lifelong as long as the investigator feels it is in the best interests of the participant.
Arm L: Transdermal oestradiol (TE2)	Minimum 2 years – *unless progression occurs before (see* **Table 10***)*	Continue until disease progression at which point it is the site investigators prerogative to choose whether to continue TE2 or change to LHRHa. (see **Table 10** for additional details on treatment post-progression)	Continue until disease progression at which point it is the site investigators prerogative to choose whether to continue TE2 or change to LHRHa. (see **Table 10** for additional details on treatment post-progression)

[1] For trial purposes: Report the planned duration of trial treatment based on the intention at the time of randomisation, i.e.: based on plans for radical RT. **However** if the treatment received is different from that planned, please ensure treatment the participant receives reflects the most appropriate duration:

E.g.: N0M0 patient does not receive planned RT > treat with lifelong ADT as per "M0 with no radical treatment"
N0M0 patient receives RT that was not planned > treat with minimum 2 years ADT as per "M0 with radical treatment"

Table 10: Management of trial treatment post progression

Continues on next page.

**Randomised treatment**	**Post-progression: How to manage Trial Treatment**			
	**M0 with radical treatment - progresses whilst still on ADT**	**M0 with radical treatment - progresses after completing 2 years ADT**	**M0 with <u>no</u> radical treatment**	**M1**
Arm A:<br>SOC androgen deprivation therapy	Participants who progress before completing 2 years of ADT should continue with ADT.<br>*ADT after progression is not considered a trial protocol treatment.*	M0 participants who progress after stopping ADT at 2 years, should restart ADT.<br>*ADT after progression is not considered a trial protocol treatment.*	*Continue ADT post-progression <u>but</u> ADT is no longer considered a trial protocol treatment.*	
Arm G:<br>Abiraterone	Continue until <u>all</u> types of progression reported:<br>• PSA progression (as defined in section **7.1.3.A**)<br>• Radiological progression<br>• Clinical progression<br>However, if the participant starts second-line treatment before meeting all progression types, abiraterone must be stopped[1].	If the site clinician wishes to retreat with abiraterone this must be done following national guidelines for use in the CRPC setting.<br>*Re-treating with abiraterone in the CRPC setting is not a trial protocol treatment.*	Continue until <u>all</u> types of progression reported:<br>• PSA progression (as defined in section **7.1.3.A**)<br>• Radiological progression<br>• Clinical progression<br>However, if the participant starts second-line treatment before meeting all progression types, abiraterone must be stopped[1].	
Arm: J<br>Abiraterone and enzalutamide	Continue until <u>all</u> types of progression reported:<br>• PSA progression (as defined in section **7.1.3.A**)<br>• Radiological progression<br>• Clinical progression<br>However, if the participant starts second-line treatment before meeting all progression types, abiraterone and enzalutamide must be stopped[1].	If the site clinician wishes to retreat with abiraterone or enzalutamide this must be done following national guidelines for use in the CRPC setting.<br>Re-treating with abiraterone or enzalutamide in the CRPC setting is not a trial protocol treatment.	Continue until <u>all</u> types of progression reported:<br>• PSA progression (as defined in section **7.1.3.A**)<br>• Radiological progression<br>• Clinical progression<br>However, if the participant starts second-line treatment before meeting all progression types, abiraterone and enzalutamide must be stopped[1].	
[1] Participants may continue on abiraterone or abiraterone and enzalutamide if they receive radiotherapy on a single occasion for a skeletal-related event				

	POST-PROGRESSION: HOW TO MANAGE TRIAL TREATMENT			
**RANDOMISED TREATMENT**	**M0 WITH RADICAL TREATMENT - PROGRESSES WHILST STILL ON ADT**	**M0 WITH RADICAL TREATMENT - PROGRESSES AFTER COMPLETING 2 YEARS ADT**	**M0 WITH NO RADICAL TREATMENT**	**M1**
Arm K: Metformin	Continue metformin post-progression. Post-progression metformin should be continued for as long as the investigator feels it is in the best interests of the participant. Metformin can be given alongside any second-line treatment for prostate cancer. However, if another trial with any IMP is started in the second-line setting, metformin must be stopped.	If progression occurs after stopping ADT but while metformin continues, (i.e. progression within the first 12 months of stopping ADT), continue metformin post-progression. If progression occurs after metformin has stopped (i.e. progression >12 months after the last administration of LHRHa), metformin should not be restarted.	Continue metformin post-progression. Post-progression metformin should be continued for as long as the investigator feels it is in the best interests of the participant. Metformin can be given alongside any second-line treatment for prostate cancer. However, if another trial with any IMP is started in the second-line setting, metformin must be stopped.	
Arm L: Transdermal oestradiol (TE2)	Continuing treatment with TE2 or change to LHRHa is at the discretion of the treating clinician.	Restart treatment with TE2 or LHRHa, the choice is at the discretion of the treating clinician.	Continuing treatment with TE2 or changing to LHRHa is at the discretion of the treating clinician.	
	TE2 can be used in combination with docetaxel, cabazitaxel and radium in the CRPC setting. We are currently evaluating the combination of abiraterone, enzalutamide and apalutamide alongside TE2 in the upfront setting. If this is proven to be safe and effective then sites will be informed and it will thereafter also be allowed in the CRPC setting. In the meantime if you wish to use abiraterone, enzalutamide or apalutamide in the CRPC setting the participant must change to LHRHa. For participants who are on the 3 patch maintenance dose and have castrate levels of testosterone, there is currently no evidence that increasing the number of patches further once the participant has progressed would be beneficial and is therefore not recommended.			

6.2.2 Research Abiraterone + Prednisolone (relevant to Arms G & J)

Note: recruitment has closed to all research comparisons containing abiraterone; that is Arm G (SOC + abiraterone) and Arm J (SOC + enzalutamide + abiraterone).

Participants allocated to Arm G or Arm J will receive abiraterone:

- Arm G: abiraterone alone (taken with prednisolone), in addition to SOC ADT
- Arm J: abiraterone (taken with prednisolone) in combination with enzalutamide, in addition to SOC ADT

6.2.2.A Abiraterone: Clinical particulars – posology and administration

Abiraterone is administered as a single 1000mg daily oral dose (4 tablets to be taken together once a day).

Abiraterone should be taken with prednisolone 5mg (or prednisone 5mg in Switzerland) daily to prevent secondary mineralocorticoid excess. See **Section 6.2.2.H** for further details on prednisolone (and prednisone).

Abiraterone absorption is increased by food therefore should be taken on an empty stomach without food. The tablets should be taken at least 2 hours after food, swallowed whole with water. No food should be eaten for 1 hour afterwards.

6.2.2.B Abiraterone: Clinical particulars - treatment duration

See **Table 9** for details. See **Section 7.1.3** for further information on the trial definition of progression.

6.2.2.C Abiraterone: Safety monitoring

: Hypokalaemia, hepatic impairment and hypertension

Abiraterone may cause:

- Hypokalaemia, due to secondary mineralocorticoid excess; this can be counteracted by co-prescription of prednisolone
- Increased liver enzymes and hepatotoxicity
- Hypertension

Regular monitoring of blood serum potassium, LFTs and blood pressure are therefore required whilst on treatment. Requirements for STAMPEDE are provided in **Table 11**. Safety monitoring requirements are consistent with the approach adopted in the LATITUDE trial in which abiraterone was evaluated in high-risk metastatic hormone-naïve prostate cancer (16) and the abiraterone Investigator Brochure (38).

In summary:

- **Two weekly** monitoring of potassium, LFTs and BP for the **first 12 weeks**
- **Monthly** monitoring of potassium, LFTs and BP from **12 weeks until 1 year**
- **After 1 year** safety monitoring of potassium, LFTs and BP can reduce to **two monthly** if the site investigator thinks it is safe and appropriate to do so.

Table 11: Safety monitoring for participants receiving research abiraterone

Note: In acute toxicity monitoring requirements may increase – see toxicity tables for additional advice.

Adverse event of interest	Monitoring Required	Frequency of monitoring whilst on abiraterone alone or in combination with enzalutamide treatment in the trial setting		
		Weeks 0 – 12	Week 12 – 12 months	12 months – end of treatment
Hypokalaemia	Blood serum potassium*	Every 2 weeks	Monthly	Participants may have the frequency of monitoring reduced to every 2 months*** if judged appropriate by the investigator.<br><br>Continued monthly monitoring is required for participants if there are concerns related to research abiraterone causing hypokalaemia.
Hepatic impairment	LFTs (ALT or AST, and bilirubin)*	Every 2 weeks	Monthly	Participants may have the frequency of monitoring reduced to every 2 months*** if judged appropriate by the investigator.<br><br>Continued monthly monitoring is required for participants if there are concerns related to research abiraterone causing hepatic impairment.
Hypertension	Blood pressure**	Every 2 weeks	Monthly	Participants may have the frequency of monitoring reduced to every 2 months*** if judged appropriate by the investigator.<br><br>Continued monthly monitoring is required for participants if there are concerns related to research abiraterone causing hypertension.

* Blood tests may be taken in the community or by a GP surgery, however the results must be reviewed contemporaneously by the trial team. It is not acceptable to wait until the participant's next oncology appointment before these are reviewed.
**Blood pressure may be monitored using documented self-monitoring or via the GP providing this is reviewed at each follow up.
***Based on advice from Janssen, product IB/SmPC and LATITUDE protocol (16)

:: Hypokalaemia - additional notes
Abiraterone may cause hypokalaemia due to secondary mineralocorticoid excess, this can be counteracted by co-prescription of prednisolone (see management of hypokalaemia Table 14).

After the first 12 months, provided the site investigator feels it is appropriate and safe to do so, it is permissible for patients to be prescribed 3 months of abiraterone. As above, safety monitoring needs to be completed at 2 monthly intervals and these results need to be monitored at the time they are available to ensure it is safe for the patient to continue taking their trial medications.

:: Hepatic impairment – additional notes
Abiraterone treatment can be associated with increased liver enzymes and hepatotoxicity, therefore regular monitoring of LFTs is required whilst on treatment. LFTs should include ALT or AST, and

bilirubin. If clinical symptoms or signs suggestive of hepatotoxicity develop, serum transaminases, in particular ALT, should be measured immediately (see management of abnormal LFTs Table 15).

After the first 12 months, provided the site investigator feels it is appropriate and safe to do so it is permissible for patients to be prescribed 3 months of abiraterone. As above, safety monitoring needs to be completed at 2 monthly intervals and these results need to be monitored at the time they are available to ensure it is safe for the patient to continue taking their trial medications.

:: Blood pressure management – additional notes
Abiraterone may cause hypertension. Investigators are required to ensure blood pressure is performed and reviewed. , it is acceptable for this to be documented self-monitoring or via the GP providing this is reviewed at each follow-up. For the management of abiraterone-induced hypertension see Table 13.

6.2.2.D Abiraterone: Cautions and contra-indications

- Unusual or allergic reaction to past abiraterone treatment
- Uncontrolled hypertension
- Uncontrolled heart failure
- Active or chronic liver disease

:: Cardiovascular history
Abiraterone should be used with caution in participants with a history of cardiovascular disease. The safety of abiraterone in participants with left ventricular ejection fraction <50% or New York Heart Association (NYHA) Class III or IV heart failure has not been established. Before treatment with abiraterone, hypertension must be controlled and hypokalaemia must be corrected.

Caution is required in treating participants whose underlying medical conditions might be compromised by increases in blood pressure, hypokalaemia, or fluid retention, e.g. those with heart failure, recent myocardial infarction, or ventricular arrhythmia.

:: Renal impairment
No dose adjustments are required in renal impairment; however, caution is advised if participants develop severe renal impairment as there is limited clinical data in this population. Systemic exposure to abiraterone after a single oral 1000mg dose did not increase in participants with end-stage renal disease on dialysis.

6.2.2.E Abiraterone: Special warnings

:: Overdose
Human experience of overdose with abiraterone is limited. There is no specific antidote to abiraterone. In the event of an overdose, administration of abiraterone should be with-held and general supportive measures undertaken, including monitoring for cardiac arrhythmias, liver function and electrolytes.

6.2.2.F Abiraterone: Interactions (medications)

Details on drug interactions are described in Table 12 provides a summary on the main interactions.

:: Anti-androgens
Abiraterone is steroid synthesis inhibitor and should not be given together with any other anti-androgens given the risk of toxicity. Cyproterone acetate should be discontinued 10 days and finasteride stopped 48 hours before commencing abiraterone. Concomitant use of any anti-androgen including dutasteride, bicalutamide, flutamide and tamoxifen is not recommended.

Participants receiving or planned for dual androgen blockade (DAB) at randomisation should not continue anti-androgens if randomised to receiving abiraterone.

:: Spironalactone
Spironolactone binds to the androgen receptor, may increase PSA levels and is associated with abiraterone resistance therefore concomitant use is **contraindicated.**

:: Statins and medicinal products associated with myopathy/rhabdomyolysis
Myopathy has occurred in patients treated with abiraterone, typically this occurs when first initiating treatment and resolves when abiraterone is stopped. Caution is recommended in participants receiving concomitant treatments with medicinal products known to be associated with myopathy/rhabdomyolysis e.g. statins.

Table 12: Drugs that may interact with abiraterone

DRUGS WHICH MAY REDUCE ABIRATERONE LEVELS			
**Substrate**	**Clinical Use**	**Drug**	**Recommendation**
CYP3A4 inducers	Anti-epileptics*	Phenytoin<br>Carbamazepine<br>Phenobarbital<br>Primadone (39)	Avoid unless no therapeutic alternative, due to risk of decreased exposure to abiraterone.
	Anti-depressants	St Johns Wart	
	Anti-TB	Rifampicin<br>Rifabutin<br>Rifapentine	
**DRUGS WHICH MAY INCREASE ABIRATERONE LEVELS**			
**Substrate**	**Clinical Use**	**Drug**	**Recommendation**
CYP3A4 inhibitors	Anti-retroviral	Atazanavir<br>Saquinavir<br>Ritonavir<br>Indinavir<br>Nelfanavir	Whilst PK studies of other CYP3A4 inhibitors did not indicate a clinically meaningful interaction, there are no specific studies of assessing an abiraterone - anti-retroviral interaction. If it is not possible to avoid the use of anti-retrovirals it would be prudent to have an awareness of a potential for an interaction and monitor for adverse effects from abiraterone.(40)
	Anti-fungal	Ketoconzole	PK studies suggest no clinically meaningful impact of interaction
**DRUGS WHICH MAY ACCUMULATE WHEN GIVEN WITH ABIRATERONE**			
**Substrate**	**Clinical Use**	**Drug**	**Recommendation**
CYP2D6	Cardiac	Metoprolol<br>Propranolol<br>Propafenone<br>Flecainide	Clinical vigilance required as drug levels may increase with abiraterone use, consider a dose reduction of medicinal products metabolised by CYP2D6.
	Anti-depressants	Desipramine<br>Venlafaxine<br>Citalopram	
	Anti-psychotics	Haloperidol<br>Risperidone	
	Analgesia	Tramadol<br>Codeine<br>Oxycodone	
	Alpha blockers	Tamsulosin (41)	
	Anti-diabetic	Repaglinide (42)<br>Pioglitazone	
	Cough suppressant	Dextromethorpam	

*narrow therapeutic index

6.2.2.G Abiraterone Undesirable Effects

The most common adverse drug reactions observed in the integrated safety data for those participants who received 1000mg abiraterone plus prednisone or prednisolone in clinical studies (n=1,070) were fatigue, arthralgia, peripheral oedema, back pain, bone pain, nausea, constipation, hypokalemia and anaemia.

The adverse events graded as Grade 3 or Grade 4 and which occurred in more than 5% of participants were fatigue, peripheral oedema, anaemia and back pain(38).

In the event of a toxicity not listed below clinicians should use their clinical judgement and take appropriate measures to treat the participant, including interruption of research treatment and/or implementing dose modifications if required. Please update treatment logs with any changes. Contact the MRC CTU for further advice if required.

Table 13: Management of abiraterone-associated hypertension (given alone or with enzalutamide)

TOXICITY EVENT	ACTION
BP repeatedly in range of 120-139/80-89 mmHg	**Continue abiraterone (and enzalutamide).**<br>Management as per investigator.
BP repeatedly in range of 140-159/90-99 mmHg	**Continue abiraterone (and enzalutamide).**<br>Management as per investigator with anti-hypertensive treatment. Follow local guidance for selection of anti-hypertensives but avoid thiazide diuretics to minimise risk of serum potassium derangement. Calcium channel antagonists or beta blockers are often preferred.<br>As with other symptoms of mineralocorticoid excess, consider increasing prednisolone dose to 5mg BID.
BP repeatedly ≥ 160/100 mmHg or life-threatening consequences of hypertension)	**Withhold abiraterone and enzalutamide**.<br>Adjust or add anti-hypertensive medications to mitigate the toxicity. When blood pressure resolves to being predominantly <140/90 or baseline, resume both abiraterone and enzalutamide at full dose with prednisolone 5mg bid.

Record hypertension grade as per CTCAE on the follow-up form if required. If a patient experiences ongoing hypertension that the treating clinician deems clinically concerning then consider referral to cardiologist or hypertension clinic.

Table 14: Management of abiraterone associated hypokalaemia (given alone or with enzalutamide)

TOXICITY EVENT	ACTION
Grade 1<br>(<LLN – 3.0mmol/L)	**Continue abiraterone (and enzalutamide).**<br>Supplement with oral potassium and monitor closely and increase prednisolone dose to 5mg BID.<br>Exclude and manage other causes of hypokalemia.
Grade 2<br>(<LLN – 3.0mmol/L and symptomatic)	**Withhold abiraterone (continue enzalutamide).**<br>Supplement with oral potassium and monitor closely and increase prednisolone dose to 5mg BID.<br>Exclude and manage other causes of hypokalemia.<br>Re-start abiraterone with close monitoring, discontinue if recurs.
Grade 3<br>(<3.0 – 2.5mmol/L)<br>or Grade 4<br>(<2.5mmol/L and life-threatening)	**Permanent discontinuation of abiraterone** and hospitalisation for intravenous potassium replacement and cardiac monitoring.<br>After the return of serum potassium to normal, prednisolone (prednisone in Switzerland) should also be discontinued.<br>The participant can continue on enzalutamide alone. If hypokalaemia persists, consider a dose reduction of enzalutamide to 120mg once a day.

Table 15: Management of abnormal LFTs associated with abiraterone (given alone or with enzalutamide)

TOXICITY EVENT	ACTION
**Grade 1** increases in AST, ALT or bilirubin (e.g. increase in AST or ALT from ULN to 3.0X ULN; increase in total bilirubin from ULN to 1.5X ULN)	**Continue abiraterone (and enzalutamide).**<br>Increase frequency of LFT monitoring to at least weekly, if the investigator judges that the laboratory abnormalities are potentially related to study medication.<br>Providing LFTs are stable for 4 weeks, resume normal LFT monitoring.
**Grade 2** increases in AST, ALT or bilirubin (e.g. increase in AST or ALT to >3.0-5.0X ULN; increase in total bilirubin from >1.5-3.0X ULN)	**Withhold abiraterone, enzalutamide** and all other concomitant medications that are potentially hepatotoxic.<br>Increase frequency of LFT monitoring to at least weekly until the LFTs return to baseline value or Grade 1, when both abiraterone and enzalutamide can be re-started.<br>Enzalutamide can be re-started with no dose reduction.<br>Abiraterone can be re-started with no dose reduction after one episode, providing this resolved within 4 weeks.<br>Dose reduction should be considered if Grade 2 derangements persist or recur; see below.
**Grade 3** increases in AST, ALT or bilirubin (e.g. increase in AST or ALT to >5.0X ULN; increase in total bilirubin to >3.0X ULN),	**Withhold abiraterone and enzalutamide** and all other concomitant medications that are potentially hepatotoxic.<br>Immediately increase the frequency of LFT monitoring to at least weekly until the LFTs return to baseline value or Grade 1.<br>Enzalutamide can be re-started with no dose reduction.<br>Abiraterone can be re-started with dose reduction to 250mg once toxicities resolved to Grade 1 or baseline. This dose can be titrated as per liver function blood tests.
**Grade 4** increases in AST, ALT or bilirubin (e.g. increase in AST or ALT to >20.0X ULN; increase in total bilirubin to >10.0X ULN)	**Immediate discontinuation of abiraterone and enzalutamide.**<br>Increase the frequency of LFT monitoring to at least weekly until the LFTs return to baseline value or Grade 1.<br>Prednisone can then be discontinued and the investigator can consider restarting enzalutamide.<br>Abiraterone should not be re-introduced.
**SCENARIO**	**ACTION**
Recurrent or persistent Grade 2 AST, ALT, or bilirubin derangement	**Withhold abiraterone and enzalutamide** and all other concomitant medications that are potentially hepatotoxic.<br>Once LFTs return to Grade 1 restart abiraterone at 250mg and titrate upwards, guided by weekly blood tests.
Second episode of Grade 3 AST, ALT or bilirubin derangement	**Withhold abiraterone and enzalutamide** and all other concomitant medications that are potentially hepatotoxic.<br>Immediately increase LFT monitoring at least weekly is required, continue until returned to baseline values or Grade 1.<br>Recommence enzalutamide initially. If abiraterone resumption is then considered, resume study treatment with abiraterone dose starting at 250mg and titrate upwards guided by LFTs
Third episode of Grade 3 AST, ALT or bilirubin derangement	Permanently discontinue abiraterone.<br>Prednisone can then be discontinued and the investigator can consider restarting enzalutamide.

An opinion from a hepatologist should be considered if there are any concerns or liver function derangement shows no improvement within 2 weeks of discontinuation of abiraterone.

Table 16: Management of fluid retention/oedema associated with abiraterone (given alone or with enzalutamide)

TOXICITY EVENT	ACTION
Grade 1-2	**Continue abiraterone (and enzalutamide).**<br>Increase prednisolone dose to 5mg bid.
Grade 3-4	**Withhold abiraterone. Enzalutamide can be continued.**<br>Consider addition of mineralocorticoid receptor antagonist eplerenone until resolution of symptoms. When fluid retention/oedema returns to baseline or resolves to ≤Grade 1, resume abiraterone at full dose with prednisone 5mg bid.<br>If symptoms do not resolve abiraterone should not be re-started and enzalutamide should be dose reduced to 120mg per day.

Table 17: Management of diarrhoea (associated with abiraterone or enzalutamide)

TOXICITY EVENT	ACTION
Grade 1-2	**Continue abiraterone and enzalutamide.**<br>Symptomatic management.
Grade 3-4	**Withhold abiraterone**. Enzalutamide can be continued in the first instance.<br>If no improvement from witholding abiraterone alone, reduce dose of enzalutamide to 120mg per day.<br>If still no improvement reduce dose of enzaluatmide to 80mg. If diarrhoea persists despite this (and it is believed symptoms are caused by abiraterone or enzalutamide) we recommend the patient stops trial treatment<br>Once resolved to Grade 1, recommence abiraterone at 750mg per day.

6.2.2.H Abiraterone: Prednisolone (prednisone in Switzerland)

The co-administration of prednisolone (prednisone in Switzerland) 5mg once daily is required whilst receiving abiraterone to prevent secondary mineralocorticoid excess.

Prednisolone should be taken as a single dose with food in the morning. If mineralocorticoid-related toxicities occur (e.g., hypokalaemia, hypertension, peripheral oedema) the prednisolone dose should be reviewed. See Table 13, Table 14 and Table 16 for advice on when an increase to 5mg BID is recommended.

If a participant experiences serious symptoms of Cushing's syndrome (e.g., weight gain, muscle loss) investigators may reduce the steroid dose but participants should be closely monitored for symptoms of secondary mineralocorticoid excess. It should be noted that weight gain and muscle loss are also associated with ADT.

If a participant allocated to receive abiraterone develops only biochemical failure, the responsible clinician may switch from abiraterone + prednisolone 5mg OD to abiraterone + dexamethasone 0.5mg OD.

6.2.3 Research Enzalutamide (Arm J)

Note: recruitment has closed to Arm J (SOC + enzalutamide + abiraterone).

Participants allocated to Arm J will receive enzalutamide in combination with abiraterone, in addition to SOC ADT. For information relating to treatment with abiraterone (and prednisolone), refer to Section 6.2.2.

6.2.3.A Enzalutamide: Clinical particulars – posology and administration

Enzalutamide will be administered as a 160mg oral dose (four capsules), taken together at the same time every day, with or without food.

6.2.3.B Enzalutamide: Clinical particulars - treatment duration

Enzalutamide will be taken for the same duration as the co-administered abiraterone, unless either abiraterone or enzalutamide is stopped for toxicity, in which case the other drug may continue.

Enzalutamide treatment duration is included in Table 9. See Section 7.1.3 for further information on the definition of progression.

6.2.3.C Enzalutamide: Safety monitoring

Safety monitoring for participants receiving research enzalutamide alone.

Please see Table 11 for safety monitoring if enzalutamide given alongside abiraterone. In acute toxicity monitoring requirements may increase – see toxicity tables for more advice in this setting.

Adverse Event of Interest	Monitoring Required	Frequency of monitoring whilst on research enzalutamide ALONE (if abiraterone previously stopped for toxicity) in the trial setting		
		Weeks 0 – 12	Week 12 – 12 months	12 months – end of treatment
Hypertension	Blood pressure*	Every 2 weeks	Monthly	Participants may have the frequency of monitoring reduced to every 3 months if judged appropriate by the investigator.<br><br>Continued monthly monitoring is required for participants if there are concerns related to research enzalutamide causing hypertension.

* Blood pressure may be monitored using documented self-monitoring or via the GP providing this is reviewed at each follow up.

6.2.3.D Enzalutamide: Cautions and contra-indications

:: History of seizures

Caution should be used in administering enzalutamide to participants with a history of seizures or other predisposing factors including, but not limited to, underlying brain injury, stroke, primary brain tumours or brain metastases or alcoholism. In addition, the risk of seizure may be increased in participants receiving concomitant medications that may lower the seizure threshold. Enzalutamide should be permanently discontinued in participants who have a seizure while on treatment.

:: Hepatic impairment

A hepatic impairment study showed that the composite AUC of enzalutamide plus N-desmethyl enzalutamide after administration of a single dose of enzalutamide was similar in patients with

baseline mild, moderate or severe hepatic impairment (Child-Pugh Class A, B or C respectively) relative to patients with normal hepatic function, and no starting dose adjustment is needed (43).

:: Renal impairment
No dose adjustments are required in renal impairment; however, caution is advised if participants develop severe renal impairment as there is limited clinical data in this population.

6.2.3.E Enzalutamide: Special warnings

:: Overdose
There is no antidote for enzalutamide. In the event of an overdose, stop treatment with enzalutamide and initiate general supportive measures taking into consideration the half life of 5.8 days. Participants may be at increased risk of seizures following an overdose.

6.2.3.F Enzalutamide: Interactions (medications)

The full induction potential of enzalutamide may not occur until approximately 1 month after the start of treatment, when steady-state plasma concentrations of enzalutamide are reached, although some induction effects may be apparent earlier. Monitoring for drug interactions should continue for at least the first month of treatment and dose adjustments considered. Given the long half-life of enzalutamide (5.8 days), effects on enzymes may persist for one month or longer after stopping enzalutamide. A gradual dose reduction of the concomitant medicinal product may be necessary when stopping enzalutamide treatment. See for further details on specific drug interactions with enzalutamide.

Details on drug interactions are described in **Table 18** provides a summary on the main interactions.

:: Anti-androgens
Enzalutamide is potent androgen receptor antagonist and should **not** be given together with any other anti-androgens given the risk of toxicity. Cyproterone acetate should be discontinued 10 days and finasteride stopped 48 hours before commencing enzalutamide (+ abiraterone). Concomitant use anti-androgens including dutasteride, bicalutamide, flutamide and tamoxifen are **not recommended**.

Participants receiving or planned for dual androgen blockade (DAB) at randomisation should not continue anti-androgens if randomised to receiving enzalutamide (+abiraterone).

Table 18: Drugs which may interact with enzalutamide

DRUGS WHICH MAY INCREASE ENZALUTAMIDE LEVELS			
**Substrate**	**Clinical Use**	**Drug**	**Recommendation**
CYP2C8 inhibitors	Lipid-lowering	Gemfibrozil	Avoid, if no alternatives, reduce enzalutamide dose to 80mg
**DRUGS WHICH MAY DECREASE ENZALUTAMIDE LEVELS**			
**Substrate**	**Clinical Use**	**Drug**	**Recommendation**
CYP2C8 inducers	Anti-tuberculosis	Rifampicin<br>Rifabutin	Avoid if possible. If the therapeutic effect of these medications is of large importance to the patient, and dose adjustments are not easily performed based on monitoring of efficacy or plasma concentrations of these medicinal products, use with caution, due to risk of decreased exposure to enzalutamide.
CYP3A4 inducers	Anti-epileptics	Phenytoin<br>Carbamazepine<br>Phenobarbital	
	Anti-depressant	St John's wort	
	Anti-retrovirals	Atazanavir<br>Saquinavir<br>Ritonavir<br>Indinavir<br>Nelfanavir	Avoid if possible. If the therapeutic effect of these medications is of large importance to the patient, and dose adjustments are not easily performed based on monitoring of efficacy or plasma concentrations of these medicinal products, use with caution. This is due to risk of decreased exposure to enzalutamide and increased risk of toxicity from the anti-retroviral medication.
**ENZALUTAMIDE MAY REDUCE DRUG LEVELS**			
**Substrate**	**Clinical Use**	**Drug**	**Recommendation**
CYP2C19	Gastric protection	Omeprazole	Omeprazole AUC reduced by 70%<br>Consider increasing dose of omeprazole for same therapeutic effect
CYP3A4	Analgesia	Fentanyl*<br>Alfentanil*<br>Tramadol	Consider alternatives as clinical effect may be reduced, if no therapeutic alternative monitor closely
	Immunosuppressants	Sirolimus*<br>Tacrolimus*<br>Cyclosporine*	
	Anti-migraine	Ergotamine	
	Cardiac	Nifedipine<br>Ivabradine	
CYP2C9	Anti-epileptics	Phenytoin*	Contra-indicated
	Anti-coagulants	Warfarin*	Warfarin AUC reduced by 56%<br>Consider switching to low molecular heparin, increase INR monitoring if this is not possible
**DRUGS WHICH MAY ACCUMULATE WHEN GIVEN WITH ENZALUTAMIDE**			
**Substrate**	**Clinical Use**	**Drug**	**Recommendation**
p-gp		Colchicine*<br>Dabigatran*<br>Digoxin*	Consider alternatives, if no therapeutic alternative monitor closely

*narrow therapeutic index

6.2.3.G Enzalutamide: Undesirable effects

Please refer to section 6.2.2.G for management of hypokalaemia, deranged LFTs, hypertension, fluid retention and diarrhoea that occurs whilst on enzalutamide in combination with abiraterone.

The safety monitoring and toxicity management plan described below takes into account AEs based on the reported clinical safety data of abiraterone and enzalutamide given separately. There are limited reported data on the safety and toxicity of the combination of enzalutamide and abiraterone; however, the recommendations summarised here have been updated in light of the experience gained in STAMPEDE as recommended by the STAMPEDE TMG.

Additional toxicity to be aware of relevant to enzalutamide alone include the following:

Table 19: Management of seizure whilst on enzalutamide treatment

TOXICITY EVENT	ACTION
Seizure (any Grade)	If any participant suffers a seizure whilst on treatment, enzalutamide should be permanently discontinued immediately. Abiraterone and prednisolone can be continued providing there are no abiraterone-specific toxicities.

Table 20: Management of arthralgia & muscle pain (associated with enzalutamide)

TOXICITY EVENT	ACTION
Grade 1-2	Symptomatic management
Grade 3-4	Reduce dose of enzalutamide to 120 mg /day

Table 21: Management of fatigue (associated with enzalutamide)

TOXICITY EVENT	ACTION
Grade 1-2	Consider a dose reduction to 120 mg/day
Grade 3	**Pause enzalutamide** for 1 week or until the toxicity grade improves to Grade 2 or lower severity.<br>Re-start at a reduced dose (120mg/day or 80mg/day), dose chosen to restart is at the treating clinicians discretion.

6.2.4 Research Metformin (Arm K)

Note: General recruitment has closed to Arm K. From protocol 21 only participants to be involved in the metabolic sub-study can be randomised to Arm K.

Participants allocated to Arm K will receive metformin, in addition to SOC treatments. All potential SOC systemic treatment options are suitable for combination with metformin.

6.2.4.A Metformin Clinical particulars – posology and administration

For all participants allocated to Arm K, metformin should start as soon as possible after randomisation and ideally within a maximum of 12 weeks.

Metformin will be given as a daily dose in addition to SOC treatment. The target dose is **850mg Std BID.**

The starting dose for metformin is 850mg Std OD. If tolerated, this should be increased to the target dose after 4-6 weeks i.e. at the first follow-up visit.

Metformin should be taken around the same time each day and treatment tolerance is best if taken with or after food. For twice daily dosing, the minimum time between doses should be 8 hours; doses should not be taken closer together if forgotten or missed.

Providing participants have a sufficient supply of STAMPEDE-labelled IMP metformin tablets, a telephone consultation may be sufficient to assess tolerance and give advice regarding dose modification in order to limit hospital visits. This interaction **must be documented** in the medical records.

:: Metformin dose modifications

If metformin target dose of 850mg Std BID is not well tolerated, the dose reductions listed in Table 22 could be implemented.

Note: Sustained release formulations (SR) can sometimes be better tolerated with less GI side-effects than the standard or immediate release formulations (Std). Both drugs provide similar exposure at a given daily dose.

Table 22: Management of metformin related GI-toxicity

<table>
<tr><th colspan="5">GRADE 1 OR 2 TOXICITY:
ASSUMES STARTING ON 850MG STD BD (1,700MG TOTAL DAILY DOSE) DOSE.
IF TOXICITY OCCURS ON LOWER DOSE – DROP TO THE NEXT DOSE LEVEL DOWN</th></tr>
<tr><th colspan="5">IF ONGOING TOXICITY</th></tr>
<tr><th>ACTION 1:</th><th>ACTION 1A:
DOSE REDUCTION LEVEL 1
(OPTIONS)</th><th>ACTION 1B:
DOSE ON RESTART AFTER PAUSE
(OPTIONS)</th><th>ACTION 2:
DOSE REDUCTION LEVEL 2
(OPTIONS)</th><th>ACTION 3:
STOP TREATMENT</th></tr>
<tr><td rowspan="2">Ensure metformin is taken with or after food and consider dose reduction
OR
Ensure metformin is taken with or after food and consider 1-2 week treatment pause.</td><td>a) 750mg SR BID (1500mg total daily dose)
OR
b) 500mg SR BID (1000mg total daily dose)
OR
c) 500mg Std BID (1000mg total daily dose)</td><td>a) 850mg Std OD (850mg total daily dose)
OR
b) 500mg SR OD (500mg total daily dose)</td><td rowspan="2">a) 850mg Std OD (850mg total daily dose)
OR
b) 750mg SR OD (750mg total daily dose)
OR
c) 500mg SR OD (500mg total daily dose)
OR
d) 500mg Std OD (500 mg total daily dose)</td><td rowspan="2">If toxicity recurrs after two dose reductions, we recommend stopping treatment</td></tr>
<tr><td colspan="2">Re-attempt a dose escalation after 1-2 month aiming to continue at the maximum tolerated dose</td></tr>
</table>

:: Treatment breaks
It is anticipated that metformin treatment will be paused for approximately 72 hours around the time of contrast-enhanced CT scans (see **Table 24**) and may need to be paused during episodes of inter-current illness.

- If metformin is paused for 6 days or less this information does not need to be recorded and no additional action is needed.
- Treatment pauses of ≥7 days must be recorded by updating the Metformin Treatment Log CRF.
- If metformin treatment is paused for more than 2 weeks, investigators may consider re-starting at 850mg Std once daily for the first 4 weeks before escalating to full dose providing tolerance is acceptable.
- If treatment is paused for >3 months or >50% of doses are missed for any reason, it is at the discretion of site investigators as to whether it is appropriate to restart trial treatment with metformin. Please ensure treatment pauses are recorded and feel free to contact the CTU for any advice if required.

6.2.4.B Metformin: Clinical particulars – treatment duration
Metformin treatment duration is included in **Table 9**.

6.2.4.C Metformin: Safety monitoring
Routine safety monitoring frequency is described in **Table 23**.

Table 23: Renal function monitoring required whilst on treatment with metformin

RENAL FUNCTION:	FREQUENCY OF MONITORING:
• Stable renal function **AND**<br>• eGFR ≥45ml/min/1.73m^2	6 monthly
• Risk of deteriorating renal function **AND/OR**<br>• eGFR falls to >30 and <45 ml/min/1.73m^2	At least 3 monthly (44)<br>*NB: Max dose is 1000mg per 24 hours in this setting*
• eGFR falls to ≤30ml /min/1.73m^2	Metformin should be paused*

*Should the Site Investigator decide the decline in renal function to ≤30ml /min/1.73m^2 is irreversible then metformin must be permanently stopped

6.2.4.D Metformin: Cautions and contraindications
:: Renal impairment
Metformin is not nephrotoxic, but is exclusively excreted by the kidneys. Therefore treatment should only be started in participants with stable renal function. Metformin should be only started when the GFR ≥45ml/min/1.73m^2, as per the metformin comparison-specific eligiblity criteria in section **4.6.1**.

Additional renal monitoring is required in any participant at risk of deteriorating renal function. In line with published prescribing recommendations, if the GFR falls to between 30-45 ml/min/1.73m^2 a **dose reduction** is required to ensure the maximum 24hr dose is 1000mg or less and monitoring of renal function is required at least 3 monthly (44).

Metformin should be **permanently stopped** if the GFR falls to ≤30ml/min/1.73m^2 and is irreversible.

See **Table 24** for situations when metformin treatment should be paused due to the risk of deteriorating renal function.

Table 24: Situations when metformin treatment should be paused due to risk of deteriorating renal function

SITUATIONS	RISK FACTOR
Iodinated contrast agents	Metformin should be paused for 24 hours prior to receiving contrast and re-started 48 hours post-administration(45).
Anaesthesia (peridural; spinal or general)	Pause metformin 48 hours prior to procedure and re-start no earlier than 48 hours following procedure, providing oral intake re-established and renal function is stable and at baseline.
Surgery	Pause metformin 48 hours prior to procedure and re-start no earlier than 48 hours following procedure, providing oral intake re-established and renal function is stable and at baseline.
Dehydration<br>e.g. nausea, vomiting or diarrhoea	Pause metformin and re-start only when oral intake is re-established and renal function is stable and at baseline.
Obstructive uropathy<br>e.g. urinary retention or ureteric obstruction	Pause metformin and re-start only when renal function confirmed to be stable and at baseline.

6.2.4.E Metformin: Special warnings

:: Metformin overdose

Hypoglycaemia has not been reported with even significant metformin overdoses although lactic acidosis has occurred in such circumstances. Participants should be urgently assessed in the event of an overdose and hospital admission considered. The management of metformin overdoses should be as per standard clinical care by the local team. The most effective way to remove lactate and metformin is haemodialysis.

6.2.4.F Metformin: Interactions (medications)

Caution is needed when initiating potential nephrotoxic drugs as metformin is renally excreted and therefore may accumulate if renal function deteriorates. Please refer to **Table 25** for more information on drugs which may require additional monitoring of renal function, at the discretion of the treating clinician.

Metformin does **not** interact with any of the other treatments for prostate cancer and **should be continued** during all further treatments given for disease progression, **provided clinicians feel it remains in the participants best interests**, as per **Table 9**.

As metformin is being given as an IMP in the context of a clinical trial, continued use will not be permitted if participants participate in other interventional clinical trials for prostate cancer (i.e. CRPC setting).

Table 25: Drugs which may require additional monitoring of renal function

Clinical use	Drug	Recommendation
Anti-hypertensives and other cardiac disease	ACE inhibitors/angiotensin II receptor blockers<br>e.g. ramipril, lisinopril, irbesartan	Increased frequency of renal function monitoring until confirmed to be stable
	Diuretics<br>e.g. furosemide, bumetanide	
Antibiotics	Aminoglycoside antibiotics<br>e.g. gentamicin or amikacin	Pause metformin during treatment<br>Re-start once treatment complete
Analgesia	NSAIDs<br>e.g. Ibuprofen, diclofenac, naproxen	Avoid if possible<br>If used increased frequency of renal function monitoring is required until confirmed to be stable

6.2.4.G Metformin: Undesirable effects

:: Gastrointestinal disturbance

Gastrointestinal disturbances are very common with metformin and include nausea, vomiting, diarrhoea, abdominal pain and loss of appetite. These are most common when first starting treatment (occur in >1/10 individuals).

If toxicities occur, a dose reduction and/or a switch to a sustained release (SR) preparation, if available, is recommended (see **Table 22**).

Other possible metformin-related toxicites included taste disturbance, skin reactions and B12 deficiency resulting in megaloblastic anaemia. If a participant becomes anaemic whilst taking metformin, investigators should consider measuring haematinics, including vitamin B12, and replace if deficient.

:: Lactic acidosis

Lactic acidosis is a very rare (3/100,000 patient years), but serious metabolic consequence. Reported cases have occurred primarily in diabetic patients with significant renal impairment who are also dehydrated. It is unclear whether this is due to the underlying diabetes or metformin. This is supported by a meta-analysis demonstrating similar rates of lactic acidosis in people with diabetes taking metformin compared with diabetic participants not taking metformin (46). This evidence suggests this side effect may be a complication of diabetes and may not be associated with metformin treatment.

The risk factors for lactic acidosis are: renal impairment, prolonged fasting or malnutrition, excessive alcohol intake, hepatic insufficiency or any condition associated with hypoxia e.g. cardiac or respiratory failure or circulatory shock due to any cause.

The risk of lactic acidosis should be considered in the event of non-specific symptoms such as muscle cramps, abdominal pain and/or severe weakness or lethargy. Any participant with a suspected metabolic acidosis requires **immediate discontinuation of metformin** and evaluation. Lactic acidosis is characterised by metabolic acidosis (decreased blood pH, high lactate above 5mmol/L and an increased anion gap and lactate/pyruvate ratio). The most effective way to remove lactate and metformin is haemodialysis.

6.2.5 Research Transdermal Oestradiol (Arm L)

:: Timeframe for commencing treatment

For all participants allocated to transdermal oestradiol, treatment should start as soon as possible after randomisation (and ideally within 1 week after randomisation). It is not necessary to wait for completion of the 4-week (or 1-month) duration of the LHRHa injection if this was previously given prior to randomisation. For those prescribed bicalutamide or flutamide prior to randomisation, this treatment should be discontinued before treatment with transdermal oestradiol can commence (no washout period is needed).

:: Transdermal oestradiol with SOC treatments

If SOC docetaxel has not been started before randomisation, it is recommended that it is started, for suitable participants, once established on transdermal oestradiol for around 4 weeks, when most participants are likely to have completed the induction period).

From protocol v21.0 onwards, participants randomised to the transdermal oestradiol comparison are also eligible to receive SOC abiraterone, enzalutamide or apalutamide in the upfront setting, as an alternative to docetaxel (see **Section 6.1.4**). However, the use of enzalutamide, abiraterone or apalutamide *prior to starting treatment* with transdermal oestradiol patches is not authorised (see **Section 4.3.3**).

Participants randomised to receive transdermal oestradiol may also receive SOC radiotherapy (see Section **6.1.2**) as clinically appropriate, as has been done in the PATCH trial.

6.2.5.A Transdermal oestradiol: Clinical particulars – posology and administration

Transdermal oestradiol is delivered either as Progynova TS 100 mcg/24 hours or since protocol v21.0 as Femseven 100 mcg/24hours transcutaneous oestradiol patches. Administration should proceed according to the following dose regimen which has been shown within the PATCH trial to be sufficient for achieving castrate levels of testosterone.

The changing of patch brand should be avoided unless absolutely necessary and should only be done following discussion with the CTU trial team.

Other type of patches should only be used in expectional circumstances and after discussion with the CTU team, as we currently do not have sufficient pharmacokinetic and clinical data to recommend the use of other types of patches.

6.2.5.B :: Induction regimen

Four transdermal transdermal oestradiol patches to be changed twice weekly (e.g. Monday and Thursday) for four weeks. A confirmatory testosterone and oestradiol sample should be taken at 4 weeks with the sample drawn the **day before** the patches are changed.

6.2.5.C :: Maintenance regimen

If the participant has achieved a testosterone value of ≤1.7nmol/L and has an oestradiol level >300pmol/L at 4 weeks, then treatment may be changed to a **maintenance regimen** of **three** patches changed twice weekly. However, current observations from the PATCH trial suggest castrate levels of testosterone are typically achieved with a plasma oestradiol level ≥500pmol/L, and sites can opt to wait until oestradiol reaches this level before switching if they prefer.

If a participant's testosterone is >1.7nmol/L or the oestradiol level is <300pmol/L at four weeks then they should remain on the induction regimen for another 4 week period, with monitoring of testosterone and oestradiol samples taken at around the week 8 time point, the day before patches

are changed. Once the participant achieves a castrate level of testosterone ≤1.7nmol/L and an oestradiol level of ≥300pmol/L, they can be switched to the maintenance regimen.

It is expected that participants remain on the prescribed dose, and any potential dose modifications other than those indicated in **Section 6.2.5** should be first discussed with the CTU team.

6.2.5.D :: Administration guidelines

Consecutive patches should be applied to different sites. It is recommended that patches are placed on dry, intact and hairless skin and on areas where little wrinkling occurs, at the following sites only:

- Shoulder girdle
- Upper body
- Hip
- Abdomen
- Back
- Upper arms
- Buttocks

Patches should not be placed on or near the breast area, or on areas of the body where there are large amounts of subcutaneous fat, particularly around the abdomen, as this could affect absorption. Please note that these recommendations are mainly based on studies in women using the patches.

To apply the patch, remove the protective liner and press on to the skin immediately, holding for at least 30 seconds to ensure proper adhesion. If necessary, tape can be used to fix the patch in place. If applied correctly, the participant can bath or shower as normal; however, the patches might come off in very hot water or in a sauna.

Prior treatment start, participants should be provided with the STAMPEDE (Arm L) Study Hormone Patch Application Information for Participants to promote good treatment compliance.

6.2.5.E Transdermal oestradiol: Monitoring Hormone Levels

During enrolment of the first cohort of participants starting treatment with a combination of abiraterone, enzalutamide or apalutamide alongside transdermal oestradiol, the CTU will monitor the changing testosterone, oestradiol and PSA levels in real time. This is to ensure that the combination of treatment does not impact the efficacy of the transdermal oestradiol patches. **Therefore please send the updated hormone results log to the CTU urgently following any blood test in the first 3 months to facilitate this safety analysis.**

Oestradiol and testosterone levels should continue to be monitored throughout follow-up, while the participant remains on transdermal oestradiol treatment, to assess for evidence of compliance and to also ensure the participant is on the appropriate dose. **Table 2** describes when these values are required, noting also that the samples can be taken at the same time as scheduled PSA measurements.

Scenarios when additional oestradiol and testosterone monitoring is required are given below.

:: Oestradiol <300pmol/L or >2000pmol/L or testosterone >1.7nmol/L while on the maintenance regime

A repeat blood test should be carried out within 4 weeks if, at any time, the participant's oestradiol level is found to be <300pmol/L or >2000pmol/L or the testosterone level is >1.7nmol/L while on the maintenance regime, with particular attention paid to the day that the patches are changed

compared to when the blood sample is drawn (should be the day before changing patches). If the participant continues to have out of range oestradiol levels, and/or persistent testosterone >1.7nmol/L, then the CTU team should be contacted for advice.

:: Change of maintenance patch dose

If the maintenance patch dose is changed at any time (for example, reducing from 3 to 2 patches changed twice weekly), then additional oestradiol and testosterone tests are required around 4 weeks after dose modification.

:: Change of patch brand

The changing of patch brand should be avoided unless absolutely necessary (see Section 6.2.5.A) but if advised by the CTU trial team, then additional oestradiol, testosterone and PSA tests are required following the change (see Table 26). It is important that participants are then monitored in real-time during this initial period, with the CTU team to be contacted if the hormone results are out of range as it may be necessary to modify the dose regimen.

Table 26: Additional assessments required following change of maintenance patch dose or brand

Assessments required	Weeks from dose modification or change in patch brand*			
	Week 0 (Prior to change)	Week 4 (Post change)	Week 8 (Post change)	Week 12 (Post change)
	Change of maintenance dose			
Oestradiol Testosterone PSA	X	X		
	Change of patch brand			
Oestradiol, Testosterone PSA	X	X	X	X**

* These additional tests are timed from the day of dose modification or day of starting new patch brand. However, if the post-change tests coincide within 1 week of scheduled tests (see Table 2), it is not necessary to repeat the tests.

** Dependent on prior results, 12 week test may be requested by CTU trial team

6.2.5.F Transdermal oestradiol: Cautions and contraindications

Tamoxifen should not be prescribed for participants receiving transdermal oestradiol.

6.2.5.G Transdermal oestradiol: Special warnings

If a participant has a cardiovascular event (see Section 7.1.4.A), discontinuation of treatment with transdermal oestradiol may be considered at the discretion of the treating clinician.

6.2.5.H Transdermal oestradiol: Interactions (medications)

The metabolism of oestrogens may be increased by concomitant use of substances known to induce drug metabolising enzymes, specifically CYP450 enzymes. However, with transdermal administration, the first-pass effect in the liver is avoided and, thus, transdermally applied oestrogens might be less affected than oral hormones by enzyme inducers. Oestradiol levels are already monitored as part of trial follow-up while participants are on transdermal oestradiol, therefore no additional monitoring is required when combining enzyme inducers with transdermal oestradiol.

6.2.5.I Transdermal oestradiol: Undesirable effects

Dermatitis can be a common side-effect of using the patches, especially in the induction period, which can usually be controlled by alternating the site of patch application. Participants should be advised that if patches become dislodged they should not put on extra patches, but apply their next set of patches when they are next due to be applied.

Prophylactic irradiation of the breast area, shown to reduce risk of gynaecomastia is permitted: a single fraction of 8Gy is recommended preferably before treatment with transdermal oestradiol (47).

6.3 CONCOMITANT TREATMENTS

All concomitant medications should be continued throughout the trial unless the responsible clinician decides otherwise, or there is a potential interaction with the trial treatment, in which case it is the responsibility of the responsible clinician to take the advised action. Please refer to each individual trial treatment section to see a list of drugs which may potentially interact.

6.3.1.A Data collection on concomitant treatments for participants in arms A, G, H, J, K, L

Long-term (>6 months) use of the following concomitant medications of classes of interest is collected:

- Statins
- Metformin (except as Arm K trial treatment)
- Aspirin
- Bisphosphonates or denosumab

This information is of interest both in terms of baseline use and ongoing use through the trial; as such it should be recorded on the Randomisation CRF and will be collected at each follow-up assessment (see Table 1 and Table 2).

6.4 TRIAL PRODUCTS

Details of the procedures for obtaining the drugs within the trial, dispensing and disposal of unused drug are given in **STAMPEDE Pharmacy Information Sheet**. Arrangements for free or discounted drugs are given in the Finance section (Section 15).

6.5 TREATMENT DATA COLLECTION

Data will be recorded on case report forms (CRFs); the original should be sent to CTU for data entry and a copy kept at the local site. Current versions of all CRFs can be found on the trial website (http://www.stampedetrial.org/) and sites will be notified of any changes throughout the course of the trial. The type of data to be recorded is detailed in the Assessments and Procedures section (Section 7).

6.6 MEASURES OF COMPLIANCE/ADHERENCE

Date of treatment, dose, delays and reasons for delays or dose modifications of all trial treatments will be recorded. The estimated number of abiraterone tablets, enzalutamide capsules or metformin tablets taken in a given time period will also be recorded as well as any dose reductions. See Table 28 for a description of the treatment logs.

Oestradiol levels will be collected for participants in the transdermal oestradiol arm and used to assess compliance to treatment (see Section 6.2.5.C).

Evidence of compliance with safety monitoring is required for participants on research abiraterone and research enzalutamide e.g. potassium and LFTs, or metformin treatment e.g. renal function, as described in sections **6.2.2.C**, **6.2.3.C**, and **6.2.4.C**. Site investigators should document in the participant's medical records the date of the blood test or review of blood pressure measurements and confirmation that the results were known to be within acceptable limits and if not, the toxicity should be graded according to CTCAE V4.0 and the action described. This should be available at on-site monitoring visits and used to verify the information provided on the follow-up CRF and treatment logs.

Note, safety monitoring for SOC abiraterone, enzalutamide and apalutamide is as per local practice and compliance data is not required by the trial.

7 ASSESSMENTS AND PROCEDURES

7.1 SCHEDULE FOR ASSESSMENTS

7.1.1 Follow-up Schedules

An individualised form with a follow-up schedule will be provided for each randomised participant. Which follow-up schedule applies depends on which comparison the participant was randomised to as summarised in Table 27.

Table 27: Summary of follow-up schedules by participant group

COMPARISON	PARTICIPANT DETAILS	FOLLOW-UP SCHEDULE
"Original"	Arms B, C, D, E, F and Arm A recruited between trial start (2005) and 15-Nov-2011	Active follow-up discontinued in Q3 2018
"Abiraterone"	Arms A and G randomised between 15-Nov-2011 and 17-Jan-2014	See **Table 1**
"Abiraterone and enzalutamide"	Arms A and J randomised between 29-Jul-2014 and 31-March 2016	See **Table 1**
"M1|RT"	Arms A and H randomised between 22-Jan-2013 and 02-Sep-2016	See **Table 1**
"Metformin"	Arms A and K randomised since 05-Sep-2016	See **Table 2**
"Transdermal oestradiol"	Arms A and L randomised since 20-Jun-2017	See **Table 2**

7.1.2 PSA, Testosterone And Oestradiol Measurements

All participants should have PSA measured prior to starting ADT and at every subsequent trial follow-up visit, regardless of allocated treatment arm. For participants who do not have a scheduled hospital visit, it is acceptable for arrangements to be made for blood samples to be drawn at their GP surgery.

For arm L participants, oestradiol and testosterone levels should continue to be monitored while the participant remains on transdermal oestradiol treatment. The first follow-up visit post-randomisation can be scheduled at 4 instead of 6 weeks to coincide with the 4-week hormone tests (see **Section 6.2.5**). These samples could be taken at the same time as the PSA tests, unless additional tests are required as detailed in **Section 6.2.5**. Blood samples should to be taken the day before the oestradiol patches are changed, to allow consistent measurements of testosterone and oestradiol with respect to the pharmacokinetic profile of the patches.

7.1.3 Assessment Of Treatment Failure (Definition Of Progression)

All participants should have baseline radiological examinations as detailed in **Section 4.2.1**. Participants are not routinely assessed for response. However, in order that objective progression can be assessed, it is recommended to have imaging taken at time of best response as judged by the treating clinician. The frequency of imaging is at the discretion of the treating clinician.

The following outcomes should be reported on the Progression log:

- Biochemical failure

- Local progression
- Lymph node progression
- Progression or development of new distant metastases, defined as lymph nodes outside the pelvis, bone or organ involvement
- Skeletal-related events confirmed as progression (see below)

7.1.3.A Biochemical Failure

For the purposes of the STAMPEDE trial, a unique threshold PSA value for biochemical failure is calculated, referred to as the **PSA progression value**.

This value is derived for each participant based on their **PSA nadir,** defined as the lowest PSA value reported between *randomisation* and 24 weeks on trial. Please refer to the PSA progression value calculator on the STAMPEDE website.

The exact method for deriving the progression value for a participant depends on the value of their PSA nadir, and how this compares to their pre-treatment PSA value (i.e. the extent of the fall in PSA from the starting point).

The PSA progression values are shown within **Figure 3**, they are calculated in one of three ways:

A. If the lowest recorded PSA value in the 24 weeks following randomisation is more than 4ng/ml and more than 50% of the pre-treatment PSA level then the participant fulfils the criteria for immediate treatment failure.

B. For participants whose PSA nadir in the 24 weeks following randomisation is less than or equal to 50% of the pre-treatment PSA level but remains above 4ng/ml, biochemical failure will be defined as a rise of 50% above the nadir level.

C. For participants whose PSA nadir is less than or equal to 4ng/ml, biochemical failure is defined as at least a 50% rise above the nadir value that is also above 4ng/ml.

Figure 3 PSA progression example scenarios

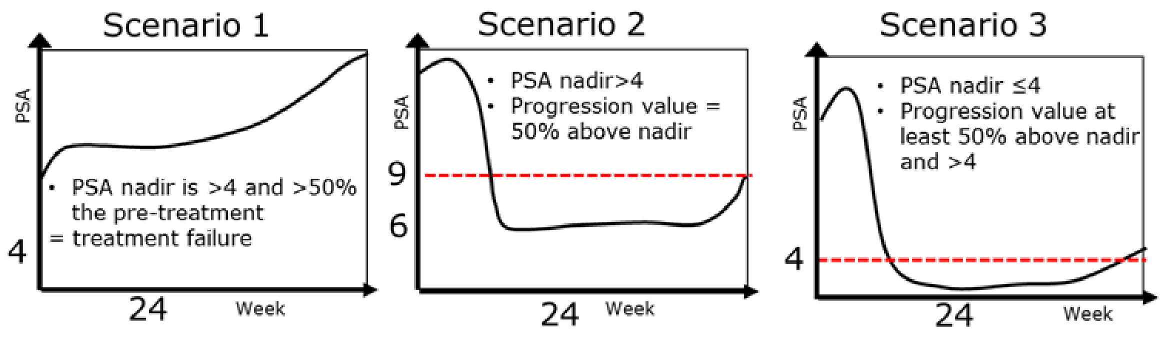

Confirming biochemical failure: the timing of assessments needs to be considered because spurious rises in PSA can occur e.g. following procedures involving the urinary tract. For this reason, any isolated rise in PSA should be confirmed before reporting biochemical failure.

In the case that the raised PSA value reaches the progression value, a confirmatory PSA test should be performed between one week and 3 months later. Biochemical failure is confirmed if the second value is around the same level or higher i.e. the trend is confirmed. The date of PSA progression

should be provided on the Progression Log as the date of the **first** raised PSA that fulfilled the trial definition of progression. Only the first instance of biochemical failure needs to be reported.

A confirmatory PSA is not required if there are other signs of progression e.g. progression of cancer related symptoms (clinical progression) or new radiological progression.

Second-line treatment commenced specifically for biochemical failure should not start until the trial definition for biochemical failure has been met. However, if second-line treatment does start before the trial definition is met then report the closest PSA value prior to the treatment start date as the progression value. This is not required if second-line treatment is being started for other signs of progression e.g. clinical or radiological.

Testosterone levels: are only required when reporting biochemical progression whilst receiving hormone treatment to confirm the diagnosis of castrate-resistant prostate cancer. Testosterone levels are not required when reporting biochemical progression in participants not receiving hormone therapy e.g. participants who presented with non-metastatic disease have relapsed following completion of treatment.

7.1.3.B Local, Lymph Node And Metastatic Failure

For each of local, lymph node and distant metastases progression, **both of** the following should be reported:

- Date of first clinical/symptomatic progression
- Date of first objective/radiological progression

7.1.3.C Skeletal-related Events

- Pathological Fracture
- Spinal cord compression
- Requirement for RT to bone (e.g. for pain or impending fracture)
- Requirement for surgery (e.g. for prevention or management of fracture)

SREs are a secondary outcome measure and a disease event of interest. SREs may represent disease progression but can also occur due to treatment-related effects e.g. osteoporotic fracture due to treatment-related bone-mineral density loss. All SREs should be recorded on the Follow-up form.

All SREs should be investigated further to establish whether or not the participant has progressed and, if confirmed as progression, a Progression Log should be completed to record this along with an Additional Treatment Log to give details of any treatment received (e.g. radiotherapy or surgical). The summary of timing of Case Report Forms can be viewed in Table 28 and Table 29.

7.1.3.D Objective/Radiological progression

Investigator determined radiological progression should be reported. For specific comparisons it may be necessary to centrally review baseline and progression scans e.g. CT scans and bone scans. Requests for scans will be made if and when these are required for a proportion of relevant participants and processes put in place for electronic transfer and site reimbursement.

7.1.4 Additional Metabolic And Cardiovascular Outcomes

A number of metabolic and cardiovascular (CVS) outcomes are being assessed in the “metformin comparison” and “transdermal oestradiol comparison” as outlined below. From protocol v17.0 onwards, a metabolic profile (lipids, glucose and HbA1c) will be measured for all participants randomised from 05-Sep-2016 onwards to capture data on metabolic and cardiovascular outcomes for both comparisons. This is collected to improve the understanding of the metabolic impacts of

ADT, and in those participants on metformin, whether any of these effects are mitigated. As this is independent of progression, testing continues post progression. See **Table 2** for a schedule of assessments, please note it is permitted to obtain these measurements within 12 weeks of the scheduled follow-up visit. The summary of timing of Case Report Forms can be viewed in **Table 28** and **Table 29**.

7.1.4.A Cardiovascular Outcomes: Transdermal Oestradiol Comparison

Cardiovascular morbidity and mortality was the primary outcome measure for the first stage in the PATCH trial (completed in 2010), which showed similar rates of CVS events in participants receiving transdermal oestradiol compared to those receiving LHRHa injections (31). These results have been confirmed by longer-term data within the trial (see **Appendix I**). Continued monitoring of CVS outcomes will be undertaken by the PATCH IDMC for both the PATCH trial, as well as for the participants in STAMPEDE allocated to transdermal oestradiol together with their contemporaneous controls.

While Arm L participants are undergoing treatment with transdermal oestradiol, the majority of these CVS events will fall under the definitions of Serious Adverse Events (see **Section 11**). Once a participant has a cardiovascular event, the discontinuation of treatment with transdermal oestradiol may be considered at the discretion of the treating clinician and the participant switched to standard of care hormone therapy.

An increased risk of venous thromboembolism has been observed when docetaxel is used in combination with certain agents for the treatment of prostate cancer. Therefore, the rate of VTE and CVS events will be closely monitored among participants within Arm L who are receiving docetaxel as part of their first-line treatment. For more details see **Appendix I**. However, within the PATCH trial (based on data up to 17-Sep-2017), no cardiovascular endpoint events had been reported among participants on transdermal oestradiol receiving upfront docetaxel.

7.1.5 Additional Safety Assessments

The comparison specific follow-up schedules are summarised in **Table 1** and **Table 2.**
These summarise all the required additional safety assessments that are required whilst participants are receiving research treatments: abiraterone, enzalutamide, metformin and transdermal oestradiol. All safety assessments are required until research treatments have been permanently stopped for more than 30 days.

The summary of the timing of Case Report Forms also can be viewed in **Table 28** and **Table 29**.

7.1.5.A Additional Safety Assessment: Research Abiraterone with or without Enzalutamide

Due to the risk of liver toxicity and secondary hyperaldosteronism with abiraterone, all participants require regular monitoring of **potassium, liver function tests and blood pressure** whilst receiving research abiraterone with or without enzalutamide. Refer to **Section 6.2.2.C** for required frequency of monitoring. Participants from Arm J who remain on enzalutamide alone require regular monitoring of blood pressure. Refer to **Section 6.2.3.C** for required frequency of monitoring.

Confirmation that potassium and liver functions test have been performed regularly and blood pressure control reviewed will be required at each follow-up visit. Any abnormalities should be graded according to CTCAE version 4.03 and recorded on the toxicity section of the follow-up CRF; any abnormalities fulfilling the criteria for a SAE (e.g. requiring hospital admission) should also be reported on a SAE CRF (see **Section 11**).

Please note, the protocol guidance relates to research abiraterone i.e. treatment received by participants allocated to arms G and J. This may be used as a guide when using SOC abiraterone, but investigators should adhere to local practice.

7.1.5.B Additional Safety Assessment: Enzalutamide

Participants in Arm J who stop abiraterone but continue with enzalutamide require ongoing monitoring of **blood pressure every 3 months** whilst receiving research enzalutamide. Safety blood tests are not routinely required for patients who remain on enzalutamide alone but can be completed at the discretion of the treating clinician.

7.1.5.C Additional Safety Assessment: Metformin

Participants with normal and stable renal function receiving metformin require monitoring of **renal function (U&Es) every 6 months** whilst on treatment. More frequent monitoring is required in participants with declining renal function, or when initiating new potentially nephrotoxic medications or at times of intercurrent illness (see Section 6.2.4.C). Changes in renal function (eGFR, graded according to CTCAEv.4) are recorded on the Follow-up CRF. It is acceptable for blood sampling to be arranged via the GP at the participant's home or local hospital.

7.1.5.D Additional Safety Assessment: Transdermal Oestradiol

Hormone levels are monitored while participants are on transdermal oestradiol, and if oestradiol levels are found to be >2000pmol/L with confirmed repeat test, please contact CTU for advice (see Section 6.2.5.B).

Real-time monitoring of testosterone, oestradiol and PSA levels is required in the first cohort of participants starting treatment with a combination of abiraterone, enzalutamide, or apalutamide alongside transdermal oestradiol (see Section 6.2.5.C).

7.2 DATA COLLECTION PROCEDURES

Treatment-related data are collected on Treatment Specific Logs. It is important that **all** treatments given for progressive disease are recorded on the Additional Treatment Log. The summary of timing of Case Report Forms can be viewed in Table 29**.**

7.2.1 Data Collection For SOC Hormone Therapy

Information relating to SOC hormone therapy is recorded on the SOC Hormone Therapy Log, unless it is a treatment change for disease progression. The SOC Hormone Therapy Log should be updated with any changes in long-term hormone therapy e.g. if anti-androgens are being added to LHRHa for dual androgen blockade in the **absence of progression**. If however, anti-androgens are being added as an additional treatment for progressive disease, then this should be recorded on the Additional Treatment Log. Please note SOC hormone therapy refers to LHRHa, anti-androgens or orchidectomy.

If a participant allocated to receive transdermal oestradiol switches to receiving SOC Hormone Therapy i.e. LHRHa, in the **absence of progression**, then this information should be recorded on the SOC Hormone Therapy Log. However, any changes in hormone therapy initiated to treat disease progression should be recorded on the Additional Treatment Log e.g. switching from transdermal oestradiol to LHRH due to progressive disease.

7.2.2 Data Collection For SOC Systemic therapy: docetaxel, abiraterone, enzalutamide, or apalutamide

The decision to use docetaxel, abiraterone, enzalutamide or apalutamide as part of the standard-of-care (SOC) must be made before randomisation and should be recorded on the Randomisation CRF

to ensure the use of SOC agents is balanced between the control and research arms. The date of the starting systemic treatment should be recorded at the time of randomisation; this can be a planned date when randomisation occurs prior to systemic treatment commencing but should be within 12 weeks of starting hormone therapy (see **Section 6.1.3**). For participants allocated to arms A, K or L all further details should be recorded on the SOC Systemic Treatment CRF and this form sent to the CTU by the 24 week follow-up appointment. If a participant does not receive the planned systemic therapy, this must also be recorded on the SOC Systemic Treatment CRF, together with the reason why. For all participants who have started or planned to start SOC abiraterone, enzalutamide, or apalutamide, details relating to starting date of treatment, dose, and permanent stopping of SOC abiraterone, enzalutamide or apalutamide must be recorded on the SOC Systemic Treatment Log. If a participant does not receive the planned SOC treatment, this must also be recorded on the SOC Systemic Treatment CRF together with the reason why.

7.2.3 Data Collection And Non-Administration Of Standard Radiotherapy

The Radiotherapy Detail CRF should be completed for **all STAMPEDE participants** regardless of being planned for, or subsequently receiving, primary radiotherapy. Where radiotherapy is not reported as planned at randomisation, this form should still be received for confirmation it was not given. For participants where radiotherapy was reported as planned at randomisation but not given, a reason should be provided on the Radiotherapy Detail CRF for example, due to early metastatic progression or participant refusal, whether this is standard-of-care radiotherapy for participants (on any research arm) or research RT to the prostate for Arm H participants.

All radiotherapy and details should be recorded on the Radiotherapy Detail CRF upon completion of the RT schedule. We will now collect acute and late RT side-effects alongside other adverse events on the toxicity form.

7.2.4 Data Collection For Palliative Radiotherapy

Details of any radiotherapy given for progressive disease should be recorded on the Additional Treatment Log and if necessary (e.g. RT for bone pain) as a Skeletal related event (SRE) on the follow-up form.

7.2.5 Data Collection for Research (M1) Radiotherapy

Arm H only: all radiotherapy and acute side-effects details will be recorded on the Radiotherapy Detail and Radiotherapy Acute Toxicity CRFs upon completion of the RT schedule; any RT late side-effects should be recorded on the Follow-up CRF under the section for RTOG Toxicities.

In those cases where RT is not given (for example, due to early metastatic progression or participant refusal), this should be stated on the Radiotherapy Detail CRF together with the reason for non-administration of the treatment.

7.2.6 Data Collection for Additional Treatments Given for Disease Progression

All treatments given for disease progression are recorded on the Additional Treatment Log. Additional treatment should not be given in the absence of disease progression. This log should be updated with all subsequent changes to treatment. Only treatments for progressive disease need to be recorded; details of supportive treatments such as pain killers or bone-strengthening agents e.g. zoledronic acid, given to relieve symptoms, do not need to be provided.

In some scenarios, SOC hormone therapies such as LHRHa or anti-androgens may be given as a treatment for progressive disease. For example, LHRHa may be re-started on relapse for participants with M0 disease who discontinued hormone therapy and commenced surveillance. In addition,

participants allocated to trandermal oestradiol may switch to LHRHa on progression. Historically, some participants progressing on LHRHa will have commenced anti-androgens (dual androgen blockade) as a treatment for progression. In all cases, if treatment is being started for disease progression, treatment data are collected on the Additional Treatment Log and the details of the progression event recorded on the Progression Log.

Please note that any change in ADT which are solely a change in the participant's long-term hormone therapy, and not for disease progression, should be reported on the SOC Hormone Therapy Log only and **not** on the Additional Treatment Log.

7.3 FOLLOW-UP PROCEDURE

Every effort should be made to follow-up all participants who have been randomised up until formal closure of a comparison. Participants should, if possible, remain under the care of an oncologist or urologist for the duration of the trial. If care of a participant is returned to the GP, it is the responsibility of the responsible clinician who obtained the participant's consent to participate in the trial to ensure that all relevant data collection forms are completed. Nurse-led follow-up is permitted and should be conducted in line with local practice and procedures. This can also be performed by a suitably qualified individual who is delegated by the Principal Investigator.

If the participant moves away from the local area, arrangements should be made for trial follow-up to be undertaken by their new local site. Details of other participating site can be obtained from the STAMPEDE Trial Team. Information on participant transfer procedures is detailed in **Section 8.2.** If the responsible clinician moves, appropriate arrangements should be made to arrange for trial follow-up to continue at the site.

All efforts should be made to preserve the initial participant's consent for long-term survival information to be flagged through national registries, for example NHS Digital (previously the Health and Social Care Information Centre); Office of National Statistics (ONS) in England/Wales; General Register Office in Scotland; Hospital Episode Statistics (HES) or Public Health England.

Please see **Section 8** for more information on early stopping of follow-up.

7.3.1 Follow-up Telephone Consultations

In certain circumstances it may be appropriate to replace hospital visits with telephone consultations providing that it is still possible to collect all the necessary follow-up information. In these instances, it is acceptable to replace appointments with telephone consultations providing the required blood results and safety tests are available to the research team. All necessary information required to complete the Follow-up CRF is still required. All details on the telephone consultation must be recorded in the participants' notes as per in person assessments.

7.3.2 Follow-up Using Electronic Healthcare Records

All participants are asked to provide consent to enable the CTU to improve the reliability of long-term follow-up data through linking to other sources of electronic healthcare data. This may include hospital based record systems, NHS digital and national registers, such as the office of national statistics or data held by public health England or other sources which hold relevant information about treatment or outcomes. To ensure study data is updated with accurate data held by others the CTU will collect direct identifiers (participant name and NHS number) and securely store this data for this purpose only, and separately from the trial database. This information will be securely transferred and used to verify the data received by the CTU.

7.4 TRIAL & COMPARISON CLOSURE

For the purpose of complying with UK the clinical regulations (UK Medicines for Human Use Act [Clinical Trials]), each comparison will only be considered 'closed' when active follow-up has ceased. Active follow-up is defined as hospital-based or telephone assessments required to be able to complete the scheduled follow-up assessments. This will be reviewed separately for each comparison after the point of the primary analysis and, if appropriate, later, updated analyses. Longer-term outcome data beyond this time point may be sought through linkage with national registers where possible (and where adequate consent has been obtained) and/or via site research teams. The end of the STAMPEDE Trial is defined as 12 months after the most recent participant, randomised to the last remaining comparison, has completed follow up. Follow-up may include the use of registry data.

7.4.1 Comparisons for which Follow-up has Ceased

In Q3-2018, active follow-up stopped for all trial participants allocated to the research arms within the "original comparisons"; this is defined as all participants allocated to arms B, C, D, E, and F together with all participants allocated to arm A recruited before 15-Nov-2011.

Active follow-up will be stopped to participants in the "M1|RT comparison" (i.e. those recruited to Arm H and the contemporaneously randomised metastatic participants allocated to Arm A), except for those participants who are in the control arm of the "enzalutamide + abiraterone comparison". This follow-up will stop between Q4-2020 and Q1-2021 after sufficient data cleaning has been completed to allow reliable publication of long-term follow-up results. Targeted lists of all participants covered by this change will be disseminated to sites when the date is confirmed.

Active follow-up is defined as hospital-based or telephone assessments required to be able to complete the scheduled follow-up assessments. It should be noted that there may still be some data collection requested from sites to support ongoing sub-studies on closed comparisons. These requests may be for confirmation of health status or data already collected at sites e.g. baseline imaging data and FFPE tumour blocks.

For M1 participants on arm C and contemporaneous arm A, the PSA at baseline will be collected retrospectively where available. Further details regarding this data collection will be disseminated to the respective sites.

Given the above, any longer term analyses of data beyond comparison closure will be performed using observational data collected through national registers and NHS Digital or other datasets, providing such data are accessible.

CRFs, clinical notes and administrative documentation should be kept in a secure location (for example, locked filing cabinets in a room with restricted access). It is permissible to archive this information providing that it can be made accessible and available to the competent or equivalent authorities, the Sponsor, and other delegated authorities with suitable notice as the data may be subject to audit or inspection from any of the above. Information must be held for 25 years after the end of the trial as per **Section 13.1.**

Table 28: Summary of timing of case report forms (CRFs)

CASE REPORT FORMS	TIMING OF ASSESSMENT AND CRF
Baseline	
Eligibility Checklist	At Randomisation
Randomisation	At Randomisation
Saliva Pathology	At randomisation or any point on trial. When saliva sample has been taken and sent to Sponsor's designated laboratory.
Treatment	
SOC Hormone Therapy Log	To be completed for participants A-K, when treatment is first started and subsequently every time there is a change in SOC hormone therapy to report (including when Arm L participants switch to SOC HT pre-progression). To be sent in with the corresponding Follow-up CRF.
SOC Systemic Therapy CRF (replaces SOC docetaxel CRF which is no longer in use)	To be completed for all participants randomised to STAMPEDE:<br>To be sent at 20 weeks after randomisation. A form is required for all participants to confirm which treatment received, including if no SOC systemic therapy received<br>Re-send upon completion of SOC abiraterone, enzalutamide or apalutamide treatment
Abiraterone and Enzalutamide Treatment Log (research treatment)	To be completed for participants on arms G or J, when treatment is first started and subsequently every time there is a dose change, treatment pause and re-start.<br>To be sent in with the corresponding Follow-up CRF.
Metformin Treatment	To be completed for participants on arm K, when treatment is first started and subsequently every time there is a dose change, treatment pause and re-start.<br>To be sent in with the corresponding Follow-up CRF.
Transdermal Oestradiol Treatment Log	To be completed for participants on arm L, when treatment is first started and subsequently when reporting change in dose or type of patch.<br>For the transdermal oestradiol arm, the 6-week follow-up form can be completed at the same time as the 4-week visit for the hormone tests (see Section 6.2.5.B)
RT Detail	To be completed for all participants randomised to STAMPEDE:<br>• Upon completion of SOC RT<br>• If planned RT is no longer planned (at 10 months after randomisation)<br>• Arm H participants when research RT completed<br>• Arm A participants with newly-diagnosed M1 disease at 3 months to confirm RT was not given
Blood Form	For arm J and contemporaneous A participants only. Taken at progression and end of first line treatment and pre-progression if participant has metastatic disease. Refer to the Sample Collection and Handling Manual for time points.
Metabolic sub study sample CRF	For arm K participants at selected sites recruited on protocol v21.0 that are participating in the metabolic sub study.

Case Report Forms	Timing Of Assessment and CRF
Assessments	
Follow-up	To be completed at every comparison specific follow-up until comparison closure (See **Table 1** and **Table 2** for comparison specific assessment schedules)
Toxicity	Required at each follow-up until 30 days after permanent stopping of protocol treatment (IMP).
Transdermal Oestradiol Treatment Hormone Results Log	To be completed whenever there are testosterone and oestradiol test results while arm L participants are on transdermal oestradiol.<br><br>For the first cohort of participants starting treatment with a combination of abiraterone, enzalutamide, or apalutamide alongside transdermal oestradiol, please immediately send the log to the CTU following any blood test in the first 3 months of treatment (see **Section 6.2.5.C**).
End of Research Treatment	To be completed when (each) allocated research treatment is permanently stopped or in the event that allocated research treatment is never started (in each case a reason for stopping/never starting should be provided).
Progression Log	To be completed at the first occurrence of each progression event (PSA, local, nodal, distant metastases) and for each method of detection (clinical/symptomatic and objective/radiological).<br>Skeletal-related events confirmed as progression should also be reported here.
Additional Treatment Log	To be completed each time a participant who has progressed starts or completes any additional treatment for progression.
Serious Adverse Event	To be completed following any Serious Adverse Event having confirmed none of the trial specific expedited reporting exemptions are met
Death	At Death
Administration	
Consent form	At Randomisation and when re-consenting following transfer procedure
Participant Transfer Confirmation Form	To be completed when a participant is transferred to a different hospital for the administration of trial treatment and follow-up
Tissue Sample Form	To be completed when sending tumour blocks to Sponsor's designated laboratory.
Co-enrolment	To be completed when a participant is co-enrolled in a post-progression interventional prostate cancer trial. Please see **Section 5.2** for more information.

Table 29: Schedule For Completion Of Treatment Forms For All Comparisons By Arm.

Timing From Randomisation			Treatment Log[1]
Years	Months	Weeks	
**6-Weekly**			
0	-	6[2]	G, J, K, L
-	-	12	G, J, K, L
-	-	18	G, J, K, L
-	6	24	G, J, K, L
**12-Weekly**			
-	9	36	G, J, K, L
1	12	48	G, J, K, L
-	15	60	G, J, K, L
-	18	72	G, J, K, L
-	21	84	G, J, K, L
-	-	96	G, J, K, L
**6-Monthly**			
2	24	104	G, J, K, L
	30	130	G, J, K, L
3	36	156	G, J, K, L
	42	182	G, J, K, L
4	48	208	G, J, K, L
	54	234	G, J, K, L
5	60	260	G, J, K, L
**Annual**			
6	72	-	G, J, K, L
7	84	-	G, J, K, L
Etc.	-	-	G, J, K, L

Key:

G = SOC + abiraterone
J = SOC + enzalutamide + abiraterone
K = SOC + metformin ± RT ± SOC docetaxel/abiraterone/enzalutamide/apalutamide
L = Transdermal oestradiol ± RT ± SOC docetaxel/abiraterone/enzalutamide/apalutamide

[1] For participants in Arm L on transdermal oestradiol, the hormone tests results are to be reported on the Transdermal Oestradiol Treatment Hormone Results Log
[2] For the transdermal oestradiol arm, the 6-week follow-up form can be completed at the same time as the 4-week visit for the hormone tests (see **Section 6.2.5.B**)

8 STOPPING OF TREATMENT OR FOLLOW-UP

Participants should be given every encouragement to adhere to their allocated protocol treatment and follow-up schedule, in order to reduce bias. However, a participant has the right to withdraw consent for participation in any aspect of this trial at any time.

8.1 STOPPING RESEARCH INTERVENTIONS

A participant may stop **any STAMPEDE research treatment** for the following reasons:

- Unacceptable toxicity
- Intercurrent illness which prevents further treatment
- Participant refusal
- Any alteration in the participant’s condition which justifies the discontinuation of treatment in the clinician’s opinion

In all cases, the reason for permanent stopping of research treatment should be recorded on the End of Research Treatment CRF.

In the event of stopping research treatment, unless a participant states otherwise, consent is assumed for continued recording of trial data.

8.1.1 Stopping Research Treatment: Abiraterone, Enzalutamide + Abiraterone

For **participants randomised to Arm G or J**, research treatment should also be discontinued for the following reasons:

- Disease progression whilst on therapy. As detailed in Section 7.1.3, the disease event for stopping treatment may be after the first reportable Failure-Free Survival event. Treatment must be stopped once all three types (biochemical, radiological and clinical) of progression have occurred.
- Intention to commence a new systemic anti-cancer treatment due to evidence of relapse

Trial abiraterone must stop if other systemic treatments are initiated at any time for disease progression control (including chemotherapy, radium-223 etc). Anti-androgens (e.g. bicalutamide) should not be given in combination with abiraterone or enzalutamide due to the risk of toxicity. However, participants may continue on abiraterone or abiraterone and enzalutamide if they receive radiotherapy on a single occasion for a skeletal-related event. Sites must contact the STAMPEDE trial team for further guidance as appropriate.

8.1.2 Stopping Research Treatment: Metformin

For **participants** randomised to Arm K, treatment duration is detailed in Table 9.
Please note that in contrast to other treatments tested in STAMPEDE metformin does **not** need to be stopped following progression. Metformin treatment should aim to **continue post-progression** whilst participants continue to receive ADT.

Reasons for early stopping of metformin include:

- Decline in renal function (metformin must be stopped if GFR ≤30ml/min/1.73m^2, see Section 6.2.4.C)
- Decline in performance status (WHO PS >2)
- Unacceptable toxicity
- Participant refusal

- Intercurrent illness preventing continued metformin treatment
- Investigator decision e.g. administration of IMP within a CTIMP in CRPC setting

If treatment is paused for >3 months or >50% of doses are missed for any reason, it is at the discretion of site investigators as to whether it is appropriate to restart trial treatment with metformin. Contact the CTU for any advice if required.

8.1.3 Stopping Research Treatment: Transdermal Oestradiol

For **participants randomised to Arm L**, treatment with transdermal oestradiol may be discontinued for the following main reasons:

- Unacceptable toxicity
- Participant refusal
- Intercurrent illness
- Investigator decision
- Cardiovascular event (see **Section 7.1.4.A**)

For participants who stop transdermal oestradiol patches due to unacceptable toxicity or intercurrent illness, site investigators can consider changing treatment to LHRHa or allowing a break from hormone therapy. On re-initiation of hormone therapy, the investigator can choose whether it is in the participant's best interests to recommence transdermal oestradiol patches or LHRHa.

If transdermal oestradiol patches are chosen to restart, it is important to recommence with the loading regimen (See **Section 6.2.5.B**) and monitor oestradiol and testosterone levels closely. The process outlined for monitoring hormone levels and titrating doses at randomisation should be used when restarting transdermal oestradiol patches in order to ensure the correct dose is achieved.

In addition, if there is evidence of disease progression, subsequent therapy is at the discretion of the treating clinician with references to any relevant guidelines (see **Table 10**).

8.2 BREAKS IN SOC ADT

The SWOG trial (48) comparing intermittent versus continuous ADT in hormone sensitive metastatic prostate cancer did not find evidence to support that intermittent therapy was non-inferior for overall survival (hazard ratio for death with intermittent therapy 1.10; 90% confidence interval 0.99 to 1.23). Thus, STAMPEDE does not support intermittent androgen therapy as an appropriate upfront treatment approach.

Some participants will experience toxicity or report their QL is adversely impacted by ADT. In these instances we would recommend trying to ameliorate any symptoms with appropriate lifestyle or medical interventions, as per local or national guidelines. Please check that any treatment for symptoms will not interact with the trial treatment.

However, if the participant continues to struggle with ADT, treatment breaks can be considered, although this may impact the trial treatment as well – see below.

Participants who require breaks in SOC ADT due to unacceptable toxicity or an intercurrent illness can restart ADT as long as the investigators deems it is safe to do so, and as long as it remains in the participant's best interests.

All treatment stop and start dates must be recorded, with the reason for the break, on the hormone therapy and trial treatment logs so they can be considered during any data analyses.

8.2.1.A Impact on trial treatment if participant has a break in SOC ADT

Abiraterone:
Participants allocated to receive abiraterone as part of Arm G cannot continue their trial treatment whilst SOC ADT is stopped.

Abiraterone in combination with Enzalutamide, or either treatment alone:
Participants allocated to receive abiraterone with enzalutamide, or remain on either agent as monotherapy, as part of Arm J cannot continue their trial treatment whilst SOC ADT is stopped.

Metformin:
Participants allocated to receive metformin as part of Arm K should continue metformin as per the proposed treatment length at randomisation after stopping SOC ADT. This is as long as it remains in their best interests as assessed by the local clinician. See **Table 9** for specific instructions about the proposed length of metformin treatment for participants who receive radical treatment versus those who do not.

Transdermal Oestradiol:
Transdermal oestradiol patches replace SOC ADT treatment. Please see transdermal oestradiol section **8.1.3** for further details about breaks or stopping trial transdermal oestradiol patch treatment.

8.3 PARTICIPANT TRANSFERS

For participants moving away from the area and planning to transfer care, every effort should be made for the participant to be followed-up at another trial site. The participant will need to sign a new consent form at the new trial site. Once this has been done, the new trial site will take over responsibility for their ongoing participation in the trial.

To document the transfer process the main contact person at both the current and receiving hospitals should complete and sign the Patient Transfer Confirmation form. A fully completed form must be returned to the CTU prior to the participant transfer and any outstanding data queries for the participant should be completed prior to transfer.

On receipt of the completed transfer form, a member of the STAMPEDE team will confirm the database has been updated and request confirmation of the name of the participant's new clinician. Photocopies of the following documents may then be sent to the new hospital to complete the transfer and originals must also be retained at the original site for monitoring purposes:

- Consent form
- Completed CRFs
- Any documentation relating to the participant's participation in STAMPEDE (participant names must be removed from any documentation).

8.4 EARLY CESSATION OF TRIAL PARTICIPATION

If a participant explicitly withdraws consent to have any further trial data recorded, their decision must be respected and the CTU must be informed in writing in the form of a letter, a template is available upon request. All communication surrounding the early cessation of trial participation should be noted in the participant's records. Please note, data for the participant prior to this decision will still be required.

In the majority of cases, participants continue to give permission for their data and information on their health to continue to be collected via clinical notes and national registries. Any information on the follow-up status, however minimal, would be helpful. Investigators are encouraged to facilitate ongoing collection of follow-up data for example, through considering telephone consultations (see **Section 7.3.1**).

Early cessation of trial participation should not be undertaken lightly and the site must consider the implications for the trial and the participant in reaching such a decision. Without long-term data, the efficacy of trial treatments would be less reliable and could lead to inconclusive results. The early stopping of trial treatment should not lead to the early cessation of trial participation and in such cases follow-up assessments should be continued as per trial protocol.

Participants can change their minds about withdrawal at any time and reaffirm their consent to participate in the trial. Follow-up data should be collected only from the point of when consent was re-instated.

9 STATISTICAL CONSIDERATIONS

9.1 METHOD OF RANDOMISATION

Participants will be randomised centrally using a computerised algorithm developed and maintained by CTU. Randomisation will be performed using the method of minimisation over a number of clinically important stratification factors with an additional random element. To decrease determinability, the factors are not listed here but can be found in the Statistical Analysis Plan.

Participants will be randomised between arms as follows:

- All participants who fulfil both comparison-specific eligibility criteria for metformin and transdermal oestradiol will be allocated between A:K:L.

- All participants ineligible for metformin, but eligible for transdermal oestradiol, will be allocated between A:L

- All participants ineligible for transdermal oestradiol, but eligible for metformin, will be allocated between A:K.

See **Appendix H** for the allocation weighting of each arm by previous protocol version; this also shows allocation weighting for research arms previously closed to recruitment.

9.2 OUTCOME MEASURES

The definitive primary outcome measure for each comparison in the trial is overall survival (all-cause mortality), unless otherwise stated. The design of the trial is such that it is important to have additional intermediate primary outcome measures to assess activity in each research arm as the trial progresses.

For comparisons involving research arms B to J the intermediate primary outcome measure is failure-free survival (FFS); this and other outcome measures are listed in **Table 30.** Note that this reflects the original analysis plan for research arm J.

Table 30: Trial Outcome Measures by Comparison Stage (Arms B-J)

COMPARISON STAGE	PRIMARY OUTCOME MEASURE	SECONDARY OUTCOME MEASURES
Pilot phase	Safety*	Feasibility
Activity Stages (AS)	Failure-free survival (FFS)†	Overall survival (OS)<br>Toxicity<br>Symptomatic skeletal events (SSE)
Efficacy Stage (ES)	Overall survival<br>Metastatic progression- free-survival (mPFS) – Arm J M0	Quality-of-life<br>Cost effectiveness<br>Failure-free survival†<br>Toxicity<br>Symptomatic skeletal events (SSE)

*Based on toxicity
†Including biochemical failure (see Section 7.1.3)

For the “enzalutamide + abiraterone comparison” the original plans for the final Efficacy Stage analysis were updated in late 2019, after the earlier analysis stages had been completed. The updated efficacy stage analysis will use metastatic progression-free-survival (mPFS) as the definitive primary outcome measure for participants with baseline M0 disease, and overall survival as the primary outcome measure for participants with baseline M1 disease.

For the “metformin comparison” the intermediate and definitive primary outcome measure are the same, being overall survival; see **Table 33** for full details of all outcome measures for that comparison.

For the “transdermal oestradiol comparison”, overall survival and progression-free survival are the definitive co-primary outcome measures, and the intermediate primary outcome measure is progression-free survival (PFS); see **Table 35**. The rationale for choosing progression-free survival rather than failure-free survival as the outcome measure for this comparison is outlined in **Section 9.7.3**.

The reasons for different emphases in each recruitment stage are explained in **Section 9.3**.

9.3 SAMPLE SIZE: PRINCIPLES

The design is a multi-arm multi-stage, multi-centre, platform, randomised controlled trial. There are a number of stages for each research arm: a Pilot/Feasibility/Safety Phase, Activity Stages and a final Efficacy Stage. Full details of the methodology underlying the trial design are given by Royston et al. (49, 50) The original sample size calculations were performed using the stage2 (version 1.2.0, Mar-2002) and stagen (version 1.1.1, May-2004) programs, both implemented in Stata (Stata Corp, TX) and updated using the later nstage program (version 1.0.3, Jun-2007; version 2.1.0, Jun-2009; version 3.0.1, Sep-2014). (51)

Other than transdermal oestradiol, we have adequately powered each comparison to detect an appropriate improvement in overall survival at the final Efficacy Stage, with high power at each of the planned interim Activity Stages to detect a pre-defined target difference in the intermediate primary outcome. For example, in a cohort with 2 years median FFS and 4 years median overall survival (OS) a target HR of 0.75 for research arm relative to control would translate into an absolute improvement in FFS of 10%, from approximately 50% to 60% at two years, and in OS of 10%, from approximately 50% to 60%, at four years.

The “transdermal oestradiol comparison” is powered only for contributing to a meta-analysis of participants from the STAMPEDE “transdermal oestradiol comparison” and the PATCH trial. It will assess non-inferiority of transdermal oestradiol in terms of overall and progression-free survival which are co-primary outcome measures. For details of the sample size calculations, planned analyses and corresponding operating characteristics, see version 10 of the PATCH protocol.

As each comparison is powered to detect a relative difference in survival, the analyses will be performed when the pre-planned number of events has been reported in the control arm, rather than after a certain number of participants have been recruited to the comparison or a certain amount of time has elapsed. Further details of the sample size calculations and varying assumptions for each research comparison are summarised in the relevant **Sections 9.4-9.8** and detailed in a separate Statistical Design Document which is available on request.

As with all trials, changes in both the standard-of-care and second-line therapies over time are possible which improve outcomes and thus will affect the observed control arm event rates and

associated reporting timelines. In particular, from protocol v8.0, standard-of-care RT was mandated for all participants with N0 M0 disease and no RT contraindication (this is likely to improve outcomes for this subgroup) and standard-of-care docetaxel permitted from protocol v14.0. Further agents are starting to be licensed for participants with castrate-refractory disease which may also improve survival rates. Improved FFS rates would delay the intermediate analyses, for comparisons where FFS is the intermediate primary outcome measure; while improved OS would delay the definitive analyses. Similarly, improved PFS rates could delay both the time of intermediate and definitive analysis for the "transdermal oestradiol comparison". For each comparison event rates are estimated based on data which are publicly available at the time of design. The Statistical Design Document for arms A-K includes models where median survival is varied around such estimated rates.

Figure 4: Schema of progress of STAMPEDE through the trial*

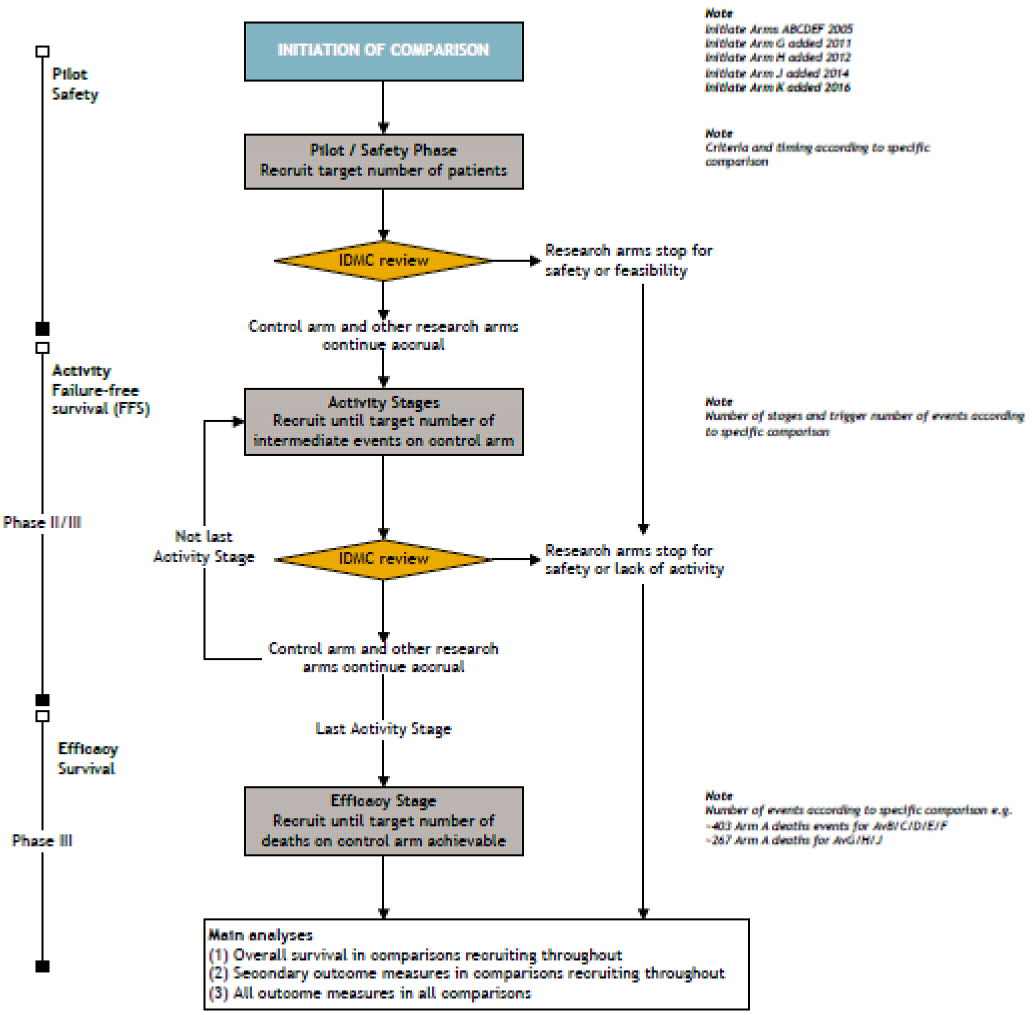

Key
FFS: Failure-free survival
HR: Hazard ratio
IDMC: Independent Data Monitoring Committee
Pts: Patients

Notes
Exact accrual depends on many factors including accrual rate, event rate and arms recruiting in each stage

* Except for the "transdermal oestradiol comparison"

9.4 SAMPLE SIZE ISSUES & TRIAL STAGES: ADDITIONAL RESEARCH ARM H

This is the "M1|RT comparison" and includes participants allocated to research Arm H (SOC+RT) and newly-diagnosed M1 participants with no contraindication to RT allocated to the control Arm A whilst Arm H was open to recruitment. Suitability for allocation to the comparison was assessed before randomisation to ensure comparability with contemporaneous control arm participants.

Table 31: Guidelines for stopping accrual to additional research Arm H

Activity Stage	Sig Level	Power	Targeted HR	Number of control arm events	Consider discontinuation if (HRobserved) is...
I	0.50	95%	0.75	~75	>1.00
II	0.25	95%	0.75	~142	>0.92
III	0.10	95%	0.75	~221	>0.89

9.4.1 Pilot Phase: Additional Research Arm H

The IDMC reviewed safety data, in the context of data from the control arm, when the first 30 participants allocated to Arm H had been on trial for around six months.

9.4.2 Activity Stages I-III: Additional Research Arm H

The same principles were applied to this new comparison as to previous comparisons and an equal allocation ratio of control arm participants to participants allocated to Arm H was employed, as for Arm G. The number of control arm events required to trigger the intermediate analyses are the same as for the "abiraterone comparison" (see Table 31).

9.4.3 Efficacy Stage IV: Additional Research Arm H

The analysis of Efficacy Stage IV for this comparison was planned for when ~267 deaths had been observed in the relevant control arm participants. This was to give 90% power to detect the targeted hazard ratio of 0.75 at one-sided significance level of 0.025.

9.4.4 Sample Size For Additional Research Arm H

Consideration was given to ceasing further randomisations to Arm H if it was not showing sufficient evidence of activity on the intermediate primary outcome measure (FFS), as for the other research arms. This research comparison is relevant to around 60% of participants joining STAMPEDE. At the point of the scientific approval, accrual was averaging around 80 participants per month to the trial; therefore, up to approximately 48 participants a month would be eligible for the comparison. If accrual to the trial was slower at 70 participants per month, then accrual to this comparison could be between 18 and 42 participants per month, depending on which other trial arms are open to recruitment at the time.

We were targeting a 25% relative improvement in overall survival following local radiotherapy to the prostate in this participant group. This is the same size of effect targeted with the other research arms in STAMPEDE. This relative improvement can be further justified in the light of MRC PR07 which demonstrated an improvement of this magnitude for adding radiotherapy to ADT in locally-advanced disease, with a hazard ratio for overall survival of 0.77 (95% CI 0.61 to 0.98). In that trial, fewer than half of the deaths were from prostate cancer, whereas in newly-diagnosed metastatic participants nearly all people will die of their disease. Therefore, it is relevant to note the relative benefit of radiotherapy in PR07 in terms of prostate cancer-specific survival, where the hazard ratio was 0.46 (95% CI 0.34 to 0.61) after a median follow-up time of 8 years (52).

We anticipated that around 1250 participants were required over 4 years to observe 267 control arm deaths after 5.25 years. This assumed that (i) recruitment was constantly 70 pts/m to the trial overall; (ii) the original research arms stopped accrual within 6 months after activation of the RT arm; (iii) the abiraterone arm stopped accrual around 24 months after activation of the RT arm; and (iv) a further new research arm with an equal allocation ratio was introduced 18 months after activation of the RT arm. In Protocol version 13.0, we reflected on these four points: (i) recruitment to the trial has been faster; (ii) the original research arms completed accrual 2 months after activation of the RT arm; (iii) the abiraterone arm stopped accrual 12 months after activation of the RT arm; and (iv) Arm J was activated 18 months after activation of the RT arm, Arm H.

Of participants joining STAMPEDE during this time, 60% have been eligible for the "M1|RT comparison". Prior to randomisation, a RT schedule had to be nominated: Weekly or Daily. We have observed that around half of participants in the comparison are nominated for RT with the Daily schedule and half for the Weekly schedule, primarily chosen by trial site with participant groups nominated for each schedule observed to be comparable at baseline. There will likely be interest to know the effect of each RT schedule when the main results are reported. This will be explored by "within schedule" comparisons of participants randomised to research vs control (arms H vs A) within each nominated RT schedule.

To ensure adequate power for these "within schedule" analyses, in Protocol version 13.0, the target sample size was increased from 1,250 participants up to around 1,800 participants, resulting in an approximate increase in the split by planned RT schedule from 625 to 900 in each "within schedule" analysis. A FFS analysis "within schedule" was carried out at the time of the "main analysis"; this was predicted to have ~300 control arm FFS events by schedule (FFS "within schedule" analysis parameters: target HR=0.75, power 90%, 1-sided α=0.015). For either of the RT schedules showing evidence of an effect on FFS, a comparative "within schedule" analysis was planned to be carried out on survival when ~199 control arm deaths are observed in that schedule comparison. This is a closed test with OS only formally compared within schedule if there is an advantage in FFS for that RT schedule at the main analysis. Thus, extending recruitment enables a secondary analysis of the impact of RT on survival by planned "RT schedule" to happen within around 18 months from the first main analysis.

All sample scenarios are documented in the Trial Master File.

All participants joining the trial will be starting long-term ADT for the first time. The focus of this comparison is on the newly-diagnosed, metastatic participants (with no contraindications to RT), which is the largest subgroup of participants in the trial and the group of participants at highest risk of death from prostate cancer. Participants with non-metastatic disease will be excluded from this particular comparison as there are already randomised data demonstrating the survival benefit from radiotherapy in participants with locally-advanced disease. Radiotherapy is now mandatory in node negative participants; it is also recommended in the node-positive, non-metastatic (N+ M0) group. Relapsing participants are also excluded from this comparison.

For the control arm of the whole trial, we constructed sample size scenarios based on median failure-free survival being 18, 24 or 30 months; the event rate would depend on the participant mix. We now know that around 60% of participants have M1 disease at trial entry and we have reported that FFS at 24 months is 51% across the whole of the control arm participant sample.(53)

For the updated sample size calculation for the "M1|RT comparison", we based our estimates on the subgroup of participants with newly-diagnosed M1 disease in the control arm. Therefore, we

estimated median FFS for control arm participants in this comparison to be 1 year and estimated that median overall survival would be around 3.5 years.

9.5 SAMPLE SIZE ISSUES & TRIAL STAGES: ADDITIONAL RESEARCH ARM J

This is the "enzalutamide + abiraterone comparison" and includes participants allocated to research Arm J (SOC + enzalutamide + abiraterone) and participants contemporaneously allocated to the control Arm A.

Note that as of Protocol version 20.0 the details of the Efficacy Stage analysis for the "enzalutamide + abiraterone comparison" have changed to reflect separate analyses for non-metastatic (M0) and metastatic (M1) participants and a broadening of the therapeutic intervention being tested. See below for further information.

9.5.1 Pilot Phase: Additional Research Arm J

The IDMC first reviewed safety data for this combination when the first 50 participants allocated to Arm J had been on trial around 6 weeks (i.e. to the first follow-up visit).

Furthermore, an additional review of safety was performed when these 50 Arm J participants had been on trial for around 6 months. Safety is routinely reviewed at regular intervals and additional safety reviews will be performed if the IDMC raises any concerns.

Direct comparison will be available with contemporaneously randomised participants on Arm A (SOC alone). Contextual data will be provided from Arm G (SOC plus abiraterone). Indicative safety data may also be available on the combination from other studies in CRPC.

9.5.2 Activity Stages I-II: Additional Research Arm J

The principles of intermediate analyses were applied to this new comparison as to previous comparisons, but some of the details were different, and an equal allocation ratio of control arm participants to participants allocated to Arm J was employed; as for Arms G and H. Owing to the expected accrual rate to the trial (>100 pts/m), the expected slower event rate in all participants given improvements to SOC and specifically in participants randomised to this comparison. Given the simultaneous recruitment of M1 (but not M0) participants to the "M1|RT comparison", only two activity stages were planned before accrual completed. These are set out in Table 32.

The IDMC intermediate activity stage reviews were completed in Nov-2015 and Mar-2016 for Arm J and recommended continuation of the comparison.

Table 32: Guidelines for stopping accrual to the additional research Arm J

ACTIVITY STAGE	SIG LEVEL	POWER	TARGETED HR	NUMBER OF CONTROL ARM EVENTS	CONSIDER DISCONTINUATION IF (HROBSERVED) IS...
I	0.40	95%	0.70	~66	>0.957
II	0.12	95%	0.70	~139	>0.869

9.5.3 Efficacy Stage III: Additional Research Arm J

The analysis of the final Efficacy Stage for this comparison was originally planned to be performed when around 267 deaths had been observed in the control arm. This would give 90% power to detect the targeted hazard ratio of 0.75 at a one-sided significance level of 0.025.

In late 2019 the plans for the Efficacy Stage III analysis of the “enzalutamide + abiraterone comparison” were updated. See section 9.5.5 for further details.

9.5.4 Sample Size For Additional Research Arm J

Consideration was given to ceasing further randomisations to Arm J if it was not showing sufficient evidence of activity on the intermediate primary outcome measure (FFS), just as for the other research arms.

The participant mix for this comparison is likely to represent a more favourable prognosis on average than in the original comparisons, due to concurrent recruitment of M1 but not M0 participants, to Arm H.

We anticipated that around 1,800 participants were required within 3.5 years to observe ~267 control arm deaths within 6 years. The default scenario assumes that (i) recruitment is constantly 70pts/m to the trial overall, (ii) Arm H (M1|RT) accrues throughout and (iii) a further new research arm with an equal allocation ratio is introduced 18 months after activation of Arm J. The stopping date for Arm G is no longer an assumption.

Variations on these factors are documented in a Statistical Design Document. If accrual rates to the trial were at 150pts/m (as observed during summer 2013), accrual of around 1,800 participants to the comparison could be achieved within 2 years. These sample scenarios are documented in the Trial Master File.

Updating the standard-of-care to include docetaxel has minimal impact on the projected time to maturity of the “enzalutamide + abiraterone comparison”.

9.5.5 Update to Efficacy Stage Analysis For Additional Research Arm J

In Protocol version 20.0 the planned Efficacy Stage analysis of the “enzalutamide + abiraterone comparison” was updated. This followed the larger-than-expected improvement in overall survival seen in the primary analysis of the “abiraterone comparison”; evidence from other trials combining enzalutamide with abiraterone did not result in further efficacy gains; and continued divergence in the aims of first-line therapy for patients with baseline metastatic and non-metastatic disease since the comparison was conceived.

In late 2019, the STAMPEDE TMG and TSC approved an updated analysis plan designed to test the impact of the addition of the more broadly-defined therapeutic intervention of androgen receptor (AR)-targeted therapy on patient outcomes, compared to SOC treatment alone. Patients from the “abiraterone comparison” (A vs G) and the “enzalutamide + abiraterone comparison” (A vs J) will be included in a combined analysis comparing the addition of AR-targeted therapy (abiraterone or the combination of enzalutamide and abiraterone) to SOC with SOC alone. Those with baseline M0 disease at entry to the study will be analysed separately to those with baseline M1 disease.

<u>Non-metastatic (M0) patients</u>
The Efficacy Stage analysis for these patients will test whether adjuvant AR-targeted therapy improves survival compared to SOC alone, using the primary outcome of metastatic progression-free-survival (mPFS).

A total of 1,982 patients with M0 disease were randomised to arms G or J or contemporaneously to the control arm. We plan to perform the Efficacy Stage analysis when a total of 315 mPFS events have been observed in the control arm patients for this sub-group. This will provide 90% power to

confirm a treatment effect equivalent to a HR of 0.75 for AR-targeted therapy, at the 1.25% one-sided significance level, based on an assumption of 70% survival in the control arm at 66 months.

Metastatic (M1) patients
The Efficacy Stage analysis for these patients will validate whether a new biomarker that includes lobular vs basal transcriptomic sub-classification (PAM50) is predictive of differential response to adjuvant AR-targeted therapy. Prior to the data freeze for the analysis, as many M1 patients as possible will be classified into one of two subgroups, biomarker positive (luminal B) and biomarker negative (basal), based on the results of the biomarker assay. The primary outcome measure will be overall survival.

A total of 1,916 patients with M1 disease were randomised to arms G or J or contemporaneously to the control arm. The timing and power for the planned analysis will depend on the proportion of these patients who are successfully classified using the biomarker assay. For example, if 50% of patients are classified, we expect to have 80% power to confirm an interaction between allocation to adjuvant AR-targeted therapy and biomarker classification at the two-sided 5% significance level if the analysis is performed in the spring of 2022, based on a minimum follow-up duration of 70 months. This assumes that 40% of patients will be classified as 'positive' and the remaining 60% as 'negative', with an anticipated treatment effect equivalent to a HR of 0.85 for AR-targeted therapy in the 'positive' group and a treatment effect HR of 0.45 for patients in the 'negative' biomarker group, equivalent to a hazard ratio ratio (HRR) of 1.9.

Further details of the calculations and assumptions underpinning the Efficacy Stage analysis planned for both subgroups can be found in the SAP for the "enzaluatmide + abiraterone comparison".

9.6 SAMPLE SIZE ISSUES & TRIAL STAGES: ADDITIONAL RESEARCH ARM K

This is the "metformin comparison" and includes participants allocated to research Arm K (SOC + metformin) and the equivalent non-diabetic participants with no contraindication to metformin contemporaneously allocated to the control Arm A whilst Arm K is open to recruitment. Suitability for allocation to the comparison is assessed before randomisation to ensure comparability with contemporaneous control arm participants

9.6.1 Implementation: Additional Research Arm K

The implementation of the MAMS principles are different in this comparison for the following reasons:

- Although all non-diabetic participants will be eligible for allocation to the "metformin comparison", the timing of the analyses will be driven only by the M1 participants. (See Section 9.6.4 for discussion of the implications for power overall and in M0/M1 subgroup analyses.)

- Failure-free survival will not be used as the intermediate primary outcome measure; overall survival will be used as both the intermediate and definitive primary outcome measure. This is because we are not convinced that any comment on metformin's usefulness should be determined from an ability to act on a PSA-driven outcome measure. Furthermore, treatment with metformin is intended to continue throughout long-term hormone therapy which may include going well beyond an FFS event, particularly in M1 participants.

- The target HR is 0.80 for overall survival (a 20% relative improvement). This is a smaller relative improvement in survival than targeted for previous comparisons because of metformin's known low toxicity profile, the low cost of the drug and the potential positive

effects on metabolic parameters and morbidity; a smaller impact on overall survival may still have clinical benefit.

9.6.2 Outcome Measures: Additional Research Arm K

Table 33 lists the outcome measures for this comparison and can be compared with the outcome measures for the other comparisons in Table 30.

Table 33: Trial outcome measures by stage for the "metformin comparison"

COMPARISON STAGE	PRIMARY OUTCOME MEASURES	SECONDARY OUTCOME MEASURES
Pilot phase	Safety*	Feasibility<br>Metabolic effects§<br>Cardiovascular event: major adverse cardiac events‡
Activity Stage (AS) I	Overall survival	Failure-free survival† (FFS)<br>Symptomatic skeletal events (SSE)<br>Toxicity<br>Metabolic effects §<br>Cardiovascular event: major adverse cardiac events‡
Efficacy Stage (ES) II	Overall survival	Metastatic progression-free survival (M0 participants)<br>Progression free survival (M1 participants)<br>Toxicity<br>Symptomatic skeletal events (SSE)<br>Failure-free survival† (FFS)<br>Metabolic effects §<br>Quality-of-life<br>Cost effectiveness<br>Correlative outcomes ₳<br>Cardiovascular event: major adverse cardiac events‡

*Based on toxicity
§Including changes in: BMI; Haemoglobin A1c (HbA1c); waist circumference and a new diagnosis of diabetes mellitus
‡MACE; nonfatal MI, nonfatal stroke, & death from CVS causes
†Including biochemical failure (see Section 6.1.2 and Appendix J)
₳Plasma lipid and fasting triglyceride levels, fasting plasma glucose
Sarcopenia and/or radiological progression free survival (rPFS)
Plasma insulin
AMP Kinase

Note: All arms are unblinded so primary outcome measures for this comparison are objectively measured with caution to be taken around interpretation of more subjective secondary outcome measures such as symptomatic skeletal events

9.6.3 Pilot Phase: Additional Research Arm K

The IDMC reviewed safety data for this comparison when the first 50 participants allocated to Arm K had been on trial around 12 months. Furthermore, analyses were conducted on metabolic parameters (see Table 33). If there was harm observed in metabolic effects, or any serious concerns regarding the toxicity profile, recruitment would be stopped; there were no formal criteria to guide this.

Safety is routinely reviewed at regular intervals and additional safety reviews will be performed if the IDMC raises any concerns.

9.6.4 Activity Stage I: Additional Research Arm K

The principles of intermediate analyses will be applied to this new comparison as to previous comparisons, but some of the details will be different, and an equal allocation ratio of control arm participants to participants allocated to Arm K is employed; as for Arms G, H and J. Owing to the expected accrual rate to the trial overall (>100 pts/m) and the interim primary outcome being overall survival, only one intermediate activity stage is planned before accrual is completed; this is set out in Table 34.

Although analyses are triggered by events in M1 participants, they will include all participants in the "metformin comparison"; this will have high power. A separate subgroup analysis in M1 participants (conventionally-powered) and M0 participants (limited power) will then look at consistency of effect; few deaths in M0 participants are expected at this time. The IDMC recommendation will be based on the totality of the available data, including safety, metabolic and compliance data.

The IDMC reviewed the intermediate activity stage data for Arm K in May 2020 and recommended continuation of the comparison.

Table 34: Guidelines for stopping accrual to the additional research Arm K

Activity Stage	Sig Level	Power	Targeted HR	Number of control arm events	Consider discontinuation if HR_K (observed) is...
I	0.40	92%	0.80	~121 M1 deaths	>0.965

9.6.5 Efficacy Stage II: Additional Research Arm K

The analysis of the final Efficacy Stage for this comparison will be performed when around 473 deaths have been observed for M1 participants randomised contemporaneously to the control arm. This would give 92% power to detect the targeted hazard ratio of 0.80 at a one-sided significance level of 0.025 at the final Efficacy Stage, and 86% pairwise power overall.

As with the intermediate activity, this analysis will include all participants in the comparison, with a separate subgroup analysis in M1 and M0 participants looking at consistency of effect. At this time point we predict approximately 100 control arm M0 deaths will be observed. Further subgroup analyses, defined by the stratification factors, are planned to check for consistency of effect at intermediate and final analyses. Due to this comparison being powered for overall survival in M1 patients, the relatively high OS for M0 patients means that anaysis of OS in this subgroup will not have high power. As such, an additional outcome measure of metastasis-free survival will be analysed as part of the subgroup analysis.

9.6.6 Sample Size For Additional Research Arm K

Consideration would be given to ceasing further randomisations to Arm K if it did not show sufficient evidence of improvement on overall survival at the intermediate analysis.

We anticipate that around 2,800 participants, including around 1,700 M1 participants, are required over 3 years to observe ~473 control arm M1 deaths over around 7 years. (This is a revision from the initial target – see **Section 9.6.7**). This number and time will be dependent on the observed overall survival. The default scenario assumes (i) recruitment is constantly 100pts/m to the trial overall, (ii) co-recruitment throughout of the equivalent of one other research arm, and (iii) the majority of metastatic participants will also have docetaxel but non-metastatic participants will not. Variations on these factors are documented in a Statistical Design Document. Sample scenarios are documented in the Trial Master File.

Updating the standard-of-care to permit first-line use of docetaxel was assumed within the sample size scenarios and is reflected in the projected time to maturity of the “metformin comparison”.

9.6.7 Further Sample Size Issues For Additional Research Arm K

Analyses for the “metformin comparison” will be timed from randomisation. The point of randomisation compared to the start of hormone therapy may differ, depending on the planned use of docetaxel. This practical information will be reviewed by the TMG and IDMC.

For the development of Protocol v19, the sample size calculations for the “metformin comparison” were discussed by the TMG and revised to the estimates as presented in Section 9.6.4 – Section 9.6.6.

The original sample size estimates for this comparison were based on a lower target for power than the previously-added comparisons, with 90% power for the interim analysis, 85% power for the final analysis and 80% pairwise power overall. The observed accrual to the “metformin comparison” is higher than forecast. Therefore the TMG took the opportunity to revisit the sample size target for the “metformin comparison”.

The revised sample size estimates aim for a higher target power of 92% at both interim and final analysis with 86% pairwise power overall, increasing the analysis power for this comparison in line with that of previous STAMPEDE comparisons. These revisions have resulted in the overall sample size for the comparison increasing from 1800 patients in Protocol v18 to 2800 patients in Protocol v19. This was determined to be achievable within the forecast timelines for recruitment i.e. by the end of 2019, and has the benefit of bringing forward the reporting timelines by approximately one year.

9.7 SAMPLE SIZE ISSUES & TRIAL STAGES: ADDITIONAL RESEARCH ARM L

This is the “transdermal oestradiol comparison” and includes participants allocated to research Arm L (transdermal oestradiol ± RT ± docetaxel) and the equivalent, eligible participants contemporaneously allocated to the control Arm A (SOC).

The phase III evaluation of the clinical efficacy of transdermal oestradiol will ultimately be based on the relevant data from this comparison within STAMPEDE and the PATCH trial, combined using an individual participant data meta-analysis. The overall evaluation is based on a non-inferiority design.

9.7.1 Implementation And Outcome Measures: Additional Research Arm L

The transdermal oestradiol evaluation is based on the following approach.

9.7.1.A Earlier Stages In The PATCH Trial

- The early stages of the PATCH trial already demonstrated the safety and early activity of transdermal oestradiol in comparison to LHRH therapy (see **Appendix I**) (31). The pilot phase (completed in 2010, n=254) showed the rates of cardiovascular events in the transdermal oestradiol and LHRH arms were similar, and the castration rates were equivalent. These results were confirmed by longer-term data including nearly 900 patients enrolled up to Oct-2015.

- A pre-planned, confidential interim analysis undertaken in Jun-2013, based on progression-free survival, at the end of the Phase II component of the PATCH trial, led the PATCH IDMC to recommend further recruitment for an extension to Phase III. That analysis included 638 patients with 206 PFS events, and reviewed data against a pre-specified non-inferiority margin hazard ratio of 1.25 with a 1-sided alpha 0.25.

9.7.1.B STAMPEDE And PATCH Meta-analysis

- To assess the clinical efficacy of transdermal oestradiol, the relevant data from the STAMPEDE “transdermal oestradiol comparison” will be combined with that data from all patients recruited into PATCH; the data from STAMPEDE will not be analysed alone.

- As the eligibility criteria with respect to the timing of start of ADT differs between the STAMPEDE “transdermal oestradiol comparison” and the PATCH trial (see **Section 4.3.1**), the “transdermal oestradiol comparison” will undergo an initial Pilot Phase to assess castration rates and safety among those participants on Arm L. This will also include a safety review of participants receiving transdermal oestradiol in combination with docetaxel. The data will be reviewed by the PATCH IDMC when there are 30 participants in Arm L who have been followed up for at least 18 weeks. A feasibility review will also be performed at the same time.

- The pre-planned Activity Stage II, on intermediate primary outcome measure progression-free survival, will take place based on combined data from the STAMPEDE “transdermal oestradiol comparison” participants and PATCH patients.

- The same approach will be used at the final Efficacy Stage, with progression-free and overall survival as definitive co-primary outcome measures (see PATCH Protocol v13.0for further details). The rationale for choosing progression-free survival as both the intermediate primary outcome measure and as part of the definitive co-primary outcome measure for the “transdermal oestradiol comparison” is outlined in **Section 9.7.3**

Table 35 summarises the outcome measures for each stage of this research comparison. The target sample size for the meta-analysis of the "transdermal oestradiol comparison" is approximately 2,500 participants, with around 700 to be recruited through the STAMPEDE "transdermal oestradiol comparison". By Feb-2017, around 1,200 patients had been recruited directly to the PATCH trial.

Table 35: Trial outcome measures by stage for the "transdermal oestradiol comparison"

COMPARISON STAGE	DATA SOURCE(S)	PRIMARY OUTCOME MEASURES	SECONDARY OUTCOME MEASURES
Pilot phase (completed 2010)	PATCH trial	Cardiovascular morbidity and mortality	Castration rates<br>Other toxicities<br>Metabolic effects
Activity Stage I (completed 2013)	PATCH trial	Progression-Free Survival*	Cardiovascular and other toxicities<br>Castration rates<br>Metabolic effects
Activity Stage II+$	PATCH and STAMPEDE trials	Progression-Free Survival*	Cardiovascular & other toxicities
Efficacy Stage III$	PATCH and STAMPEDE trials	Progression-Free Survival*<br>Overall survival	Cardiovascular & other toxicities<br>Prostate cancer specific survival<br>Quality-of-life

* Defined as the earliest among biochemical failure, clinical progression (local progression, lymph node progression, distant metastases), or death from any cause (see Section 9.7.3).

+ In addition, there is Pilot Phase to assess castration rates and safety among Arm L participants within STAMPEDE, since the eligibility criteria with respect to timing of start of ADT differs between the transdermal oestradiol comparison within STAMPEDE and the PATCH trial (see Section 4.3.1).

$ The timing of these analyses is determined by when a pre-specified number of events for the primary outcome measure have been observed in the control arms for the PATCH and STAMPEDE trials combined. Please see the PATCH Protocol v10.0 for further details.

9.7.2 Additional Use of Outcome Data from the "transdermal oestradiol comparison"

Participants allocated to the "transdermal oestradiol comparison" may provide additional consent to participate in translational sub-studies, see Section 4.7 for details. Subsequent correlative analysis using outcome data from these participants will be undertaken by the STAMPEDE team and collaborators, overseen by the STAMPEDE BRG and other STAMPEDE oversight committees.

9.7.3 Definition of PFS and Use As Co-primary Outcome Measure: Additional Research Arm L

Note that the definition of progression-free survival (PFS) used within the "transdermal oestradiol comparison" analyses differs slightly to that of failure-free survival used for other research comparisons within STAMPEDE. This is because it includes death from <u>any</u> cause as an event- i.e. both PCa deaths and non-PCa deaths (see Appendix C for further details of the definition of progression). Progression-free survival is hence defined as time from randomisation to the first of: biochemical failure, clinical progression or death from any cause.

The use of PFS rather than FFS for the "transdermal oestradiol comparison" has no practical impact on STAMPEDE. The rationale for choosing PFS as part of the co-primary outcome measure for the "transdermal oestradiol comparison" is to capture any potential effects on survival due to the different toxicity profiles between transdermal oestradiol and LHRH.

Although PFS and survival are co-primary endpoints, their respective primary analyses will be triggered at different timepoints particularly because PFS is likely to contain a relatively low proportion of deaths as the contributing first PFS event.

9.7.4 Abiraterone/Enzalutamide/Apalutamide enhanced safety monitoring

Most patients with metastatic disease entering the trial prior to the COVID-19 pandemic received docetaxel as part of their standard treatment. However, following the COVID-19 pandemic, clinicians have the option to offer alternative therapies including enzalutamide, abiraterone, or apalutamide. There is no experience of combining these agents with transdermal oestradiol patches (though no significant interaction is anticipated) an enhanced safety monitoring study will be conducted. This will involve close monitoring of all patients who receive these agents in combination with transdermal oestrogen including testosterone, oestradiol, PSA levels and any events reported through SAE forms in real time.

In addition, the IDMC will formally review the enhanced safety monitoring data utilising a Simon Two Stage design, based on the castration rates at twelve weeks among patients still undergoing treatment with patches, with a significance level of 5% and power of 80%. The enhanced safety monitoring study will test a null response of 78% (P0) against an alternative response of 93% (P1), the level observed in patients receiving patches alone. The optimal design requires at least 8/10 patients to be castrate at stage I, and 37/43 patients to be castrate at stage II. Alongside efficacy data, toxicity data will undergo clinical review, and both aspects will be considered before deciding whether further patients should be treated with the combination. Any concerns raised by any aspect of the data will be discussed between the IDMC and appropriate TMG members, with any recommendations discussed with the TSC.

Clinicians have the option to treat patients with any of enzalutamide, abiraterone, or apalutamide. Initial analyses will consider patients who receive any of these treatments. If sufficient patients receive any particular one of these treatments, secondary analyses will look within each treatment.

9.8 FURTHER NOTES ON TRIAL DESIGN

9.8.1 Overall Sample Size

Given the adaptive nature of the study, there is no formal overall sample size target, but the numbers of participants required for each comparison are detailed in **Sections 9.4-9.7**. To date, more than 11,000 participants have been recruited overall.

9.8.2 Factorial Design

We note here that we did not employ a factorial design in the original design of this trial because we anticipated the possibility of synergy between SOC, zoledronic acid and docetaxel and between SOC, zoledronic acid and celecoxib.

It would not be possible to assess any such interactions reliably in a factorial trial (see the Statistical Design Document for further details).

9.9 INTERIM MONITORING AND ANALYSES

The accumulating data will be reviewed at regular intervals (approximately annually) by an Independent Data Monitoring Committee (IDMC), including pre-specified formal intermediate analyses of activity data (see also **Section 16**). These analyses will be performed by the trial team at CTU. Only participants randomised contemporaneously, and eligible for that comparison, will be included in the comparison of each research arm against control e.g. participants allocated to the control arm prior to Protocol version 15.0 will not contribute to the "metformin comparison" (Arm A vs Arm K). For the "transdermal oestradiol comparison", the relevant STAMPEDE data will only be analysed as a meta-analysis in combination with the PATCH trial. Therefore, interim data from this comparison will be reviewed by the PATCH IDMC.

The IDMC will be asked to give advice on whether the accumulating data from the trial justifies continuing recruitment of further participants or further follow-up; guidelines for discontinuation of accrual for the relevant Activity Stages, together with results from any other relevant trials will aid them in this. A decision to discontinue recruitment, either in all participants or in selected subgroups, will be made only if the result is likely to convince a broad range of clinicians including those entering participants into the trial and the general clinical community. The intermediate stopping guidelines apply to the intermediate primary outcome measure.

To stop accrual early for benefit in any comparison would require convincing data in terms of the definitive primary outcome measure, overall survival. For example, this could be one-sided $p<0.0005$ as proposed by Haybittle-Peto.(54, 55) The use of such a guideline for stopping for benefit has a minimal impact on the operating characteristics.

If a decision is made to continue without change, the IDMC will advise on the frequency of future reviews of the data on the basis of accrual and event rates. The IDMC will make recommendations to the Trial Steering Committee (TSC, see **Section 16**) as to whether the trial should continue in its present form. While the trial is ongoing the accumulating data will generally remain confidential, unless the TSC and IDMC agree that the data should be made public.

9.10 OUTLINE ANALYSIS PLAN

Analyses will be performed on an intention-to-treat basis.

For comparisons involving arms A-K, the standard unadjusted log-rank approach will be applied to analyses of intermediate and definitive primary outcome measures. The impact of potential confounders including the stratification factors used at randomisation will be considered in a Cox proportional hazards model.

Flexible parametric models will be used to calculate the absolute differences between the arms to show treatment differences over time and to estimate restricted mean "survival" times (RMST). The estimated difference in RMST will be used preferentially to compare treatment arms if the proportional hazards assumptions required for hazard ratios cannot be supported.

In the "transdermal oestradiol comparison," a meta-analysis approach will be used to combine data from the STAMPEDE and PATCH trials. The analysis will also take into account the change in randomisation ratio partway through the PATCH trial (from 2:1 for transdermal oestradiol versus LHRH before Feb-2011, to 1:1 thereafter). In addition, as the comparison uses a non-inferiority design, sensitivity analyses will be conducted based on a number of pre-defined descriptions for the per-protocol population.

9.10.1 Pilot / Safety Phases

Feasibility of the trial originally, and now of individual research comparisons, was and still is considered in terms of acceptability of the trial randomisation, reported toxicities and adherence to trial medication. Sites participating in the Pilot Phase for the original research arms were required to keep an anonymised log of all participants assessed for trial eligibility (see protocol v2.0) so that the number of participants who did not participate in the study and the number of eligible participants who chose to not participate in the study could be summarised (reasons for non-participation were collected where the participant was willing). The anonymised logs are no longer needed for new research arms (since protocol v8.0).

For each research comparison we shall describe the incidence of expected and unexpected severe toxicities and adverse events/reactions (see **Section 11.1.1**) amongst the participants who are randomised to the comparison to decide whether to continue beyond this Pilot/safety Phase.

9.10.2 Activity And Efficacy Stages

The approach to analysis of these stages is summarised within the sample size calculations (see earlier subsections of **Section 9**). Each research arm will be compared in a pairwise fashion against the contemporaneously recruited control arm, except for the planned Efficacy Stage analyses for the "enzalutamide + abiraterone comparison", in which patients allocated to AR-targeted therapy (arms G and J) will be compared against contemporaneously recruited control arm patients.

Full details are available in the relevant Statistical Analysis Plan. See **Figure 4** for an overview of the schema of progress.

10 MONITORING AND QUALITY ASSURANCE

10.1 DATA MONITORING

To ensure patient safety and data integrity is maintained to a high standard, remote and on-site monitoring will be conducted throughout the lifetime of the study.

10.1.1 Central Monitoring Of Consent

Anonymised copies of the participant's initial consent form (including the additional research consent) should be sent to the STAMPEDE team at the CTU, as soon as randomisation has been completed. Once the consent has been received and reviewed by the CTU the participants "treatment and follow up schedule" can be released to sites.

Any subsequent re-consent forms should be sent as soon as possible to enable central monitoring and recording of consent. The dates and signatures should be visible on the copies sent to the CTU; however the name of the participant must be omitted. Any queries resulting after central monitoring will be redirected to sites for clarification. The original non-anonymised consent forms should be kept at site in the Investigator Site File.

10.1.2 Central Monitoring Of Data

Data provided to the CTU will be checked for data errors, inconsistent and missing data. The STAMPEDE team will issue data clarification requests, query reports or address issues identified via email with site staff. Data Quality and site performance sites will be reviewed, issues identified when appropriate will be fed back to sites. Sites may be identified for training or onsite monitoring through central monitoring checks.

10.1.3 Direct Access to Patient Data

Participating investigators should agree to allow trial-related monitoring, including audits, ethics committee review and regulatory inspections by providing direct access to source data and documents as required. Patients' consent for this must be obtained. A list of source data use for the trial and their locations should be maintained by the site.

10.1.4 Monitoring Visits to Investigator Sites

A selection of institutions will be visited during the course of the STAMPEDE trial. The CTU will give the responsible investigator prior notice of the monitoring visit to allow adequate time, space and staff for these visits. The standard operating procedures (SOPs) for monitoring are available from the CTU.

After the monitoring visit the monitor will complete a site visit report. Once the TMT have reviewed the report and agreed on any recommendations the monitor will finalise the report and send a copy to the Principal Investigator (PI) at the site. A copy will be kept in the CTU STAMPEDE Trial Master File.

Remote or self- monitoring could be utilised through the course of the trial. Site staff may be asked to scan and send anonymised sections of a participant's medical record to the CTU for remote verification or asked to complete a form to confirm compliance with protocol procedures.

10.2 CONFIDENTIALITY

All information collected during the course of the research will be kept strictly confidential. In addition, all procedures for handling, processing, storage and destruction of data are compliant with the Data Protection Act 1998. No individual participants will be identified when results from the trial are published.

Participants are asked to give their permission for information about their health status to be obtained from the Office of National Statistics (ONS), via NHS Digital (formerly HSCIC), Public Health England, National Cancer Research Advisory Service, or any similar or national equivalent. This will facilitate data collection and verification and reduce the burden on sites. In addition, participants will be asked for permission to inform their GP of their involvement in the STAMPEDE trial.

11 SAFETY REPORTING

The principles of GCP require that both investigators and sponsors follow specific procedures when reporting adverse events/reactions in clinical trials. These procedures are described in this section of the protocol and in **Section 7.1.5**.

Further information on the expected toxicities for the protocol treatments (investigational medicinal products (IMPs)) being tested in arms on active follow-up can be found in the reference safety information (RSI) accessible via the STAMPEDE website: http://www.stampedetrial.org/centres/essential-documents/reference-safety-information-rsi/.

11.1 SAFETY REPORTING DEFINITIONS

The definitions of the EU Directive 2001/20/EC Article 2 based on the principles of GCP apply to this trial protocol. These definitions are given in **Table 36**.

Table 36: Event Terms and Definitions

TERM	DEFINITION
Adverse Event (**AE**)	Any untoward medical occurrence in a clinical trial participant to whom a medicinal product has been administered. These include occurrences which are not necessarily caused by the product.
Adverse Reaction (**AR**)	Any untoward and unintended reaction to an investigational medicinal product related to any dose administered.
Unexpected Adverse Reaction (**UAR**)	An adverse reaction, the nature or severity of which is not consistent with the information about the medicinal product in question set out in reference safety information (summary of product characteristics or Investigator brochure) for that product.
Serious Adverse Event (**SAE**) or Serious Adverse Reaction (**SAR**) or Suspected Unexpected Serious Adverse Reaction (**SUSAR**)	Respectively any adverse event, adverse reaction or unexpected adverse reaction that fulfils the definition of **serious:**<br>• results in death<br>• is life-threatening*<br>• requires hospitalisation or prolongation of existing hospitalisation**<br>• results in persistent or significant disability or incapacity<br>• consists of a congenital anomaly or birth defect<br>• Other important medical condition***

Clarifications and Exceptions

*The term 'life-threatening' in the definition of 'serious' refers to an event in which the participant was at risk of death at the time of the event; it does not refer to an event which hypothetically might have caused death if it were more severe.

**Hospitalisation is defined as an inpatient admission, regardless of length of stay, even if the hospitalisation is a precautionary measure for continued observation. A&E attendances are not defined as a hospitalisation unless participants are admitted. Hospitalisations for a pre-existing condition, not thought to have been exacerbated by STAMPEDE protocol treatment or IMPs (including elective procedures that have not worsened) do not constitute an SAE.

*** Medical judgement should be exercised in deciding whether an AE/AR is serious in other situations. Important AE/ARs that are not immediately life-threatening or do not result in death or hospitalisation but may jeopardise the

participant or may require intervention to prevent one of the other outcomes listed in the definition above, should also be considered serious.

11.1.1 Adverse event definitions

Adverse events (AE) include:

- An exacerbation of a pre-existing illness
- An increase in frequency or intensity of a pre-existing episodic event or condition
- A condition (even though it may have been present prior to the start of the trial) detected after trial drug administration
- Continuous persistent disease or a symptom present at baseline that worsens following administration of the study treatment

NB: Within STAMPEDE non-melanoma skin cancers (e.g.: basal cell carcinoma and squamous cell carcinoma) are not considered important medical conditions and therefore are considered adverse events, unless they fulfil any of the other "serious" criteria – detailed in Table 36.

Serious Adverse Events (SAE) are AEs that fulfil the definition of serious as detailed in Table 36**.** SAEs are reported using the SAE CRF. If the event is assessed as possibly, probably or definitely related to a protocol treatment (IMP), it is categorised as a Serious Adverse Reaction (SARs). If the reaction is unexpected based on the approved reference safety information, it is categorised as a Suspected Unexpected Serious Adverse Reaction (SUSAR), see Table 37**.**

Notable Adverse Events (NAE) these include pregnancy occurring in a partner of a STAMPEDE participant. Pregnancies must be followed up until outcome, whether this is a live birth, stillbirth, or planned or spontaneous abortion. NAEs should be reported on the SAE CRF, in the same manner as SAEs.

11.1.2 Defining "treatment" for the purposes of safety reporting

STAMPEDE is an adaptive platform protocol in which research treatments are given in addition to standard-of-care (SOC) therapies, or as alternatives in the case of transdermal oestradiol. As per MHRA recommendations **all protocol treatments** (i.e. both protocol SOC and protocol research treatments) and are regarded as **investigational medicinal products** (IMPs) within the STAMPEDE platform for the purposes of safety reporting.

Protocol treatments (IMPs):

- **Protocol SOC treatments** are IMPs that are standard forms of treatment permitted as part of the STAMPEDE protocol.
    - Licensed ADT (e.g. LHRH analogues) given in the setting of hormone-sensitive prostate cancer
    - Docetaxel given in hormone-sensitive prostate cancer
    - Abiraterone given in hormone-sensitive prostate cancer
    - Enzalutamide given in hormone-sensitive prostate cancer
    - Apalutamide given in hormone-sensitive prostate cancer

Please note, if a participant allocated to transdermal oestradiol switches to standard ADT in the absence of progression, this would still be considered as being on protocol treatment (IMP).

- **Protocol research treatments** are the IMPs that are additional or alternative treatments participants allocated to research arms on active follow-up (G-L) receive as part of the STAMPEDE protocol:

- Arm G: abiraterone
- Arm J: abiraterone & enzalutamide
- Arm K: metformin
- Arm L: transdermal oestradiol

Note, the research treatment in arm H (prostate RT) is not an IMP, but safety reporting requirements to the CTU are the same.

Non-protocol treatments:

- All prostate cancer treatments commenced post disease progression (as defined in the protocol – Section 7.1.3).
- ADT given after progression, (e.g.: commenced in HSPC setting and now continues for the management of CRPC, or ADT given after progression after completing M0 course of treatment - See Figure 5).

11.2 SITE INVESTIGATOR RESPONSIBILITIES

The Site Investigator may be any medically qualified individual delegated to undertake safety reporting for the STAMPEDE trial. It is recommended that the Principal Investigator delegate safety reporting to at least one other individual in order to ensure reporting cover during their absence.

11.2.1 Notification period

All events that fall within the notification period must be reported, events outside the notification period do not need reporting.

Adverse Events (AEs): All AEs are reportable from the time of randomisation until 30 days after discontinuation of protocol treatment (IMPs)* (refer to Section 11.1.2). All AEs should be recorded in the participant's medical notes and on the Toxicity (AE) CRF linked to the Follow-up CRF. The Toxicity (AE) CRF should be sent to the CTU within one month of the corresponding Follow-up CRF being due.

Serious Adverse Events (SAEs): All unrelated events i.e. SAEs are reportable from the time of randomisation until the participant has progressed AND is 30 days after discontinuation of all protocol treatment (IMPs)* (refer to Section 11.1.2) or comparison closure (see Section 7.4 for definition of comparison closure).

*N.B. ADT before progression is a protocol SOC treatment (IMP). However, even though ADT following progression is not protocol SOC treatment (IMP) the reporting period continues until depot expiry of the last dose before progression + 30 days is completed. Therefore when the participant is on ADT in the form of LHRHa, this is assumed to be 30 days after the depot expiration date (e.g. up to 8 weeks after administration of a 4-week depot or 16 weeks after administration of a 12-week depot) following the final dose given before progression was diagnosed (See Figure 5).

Serious Adverse Reactions (SARs and SUSARs): All related SAEs i.e. all SARs and SUSARs are reportable from the time of randomisation until comparison closure (see Section 7.4 for definition of comparison closure).

Notable Adverse Events: All notable adverse events are reportable from randomisation until comparison closure, using the SAE CRF. Notable Adverse Events must be reported within 24 hours of the investigator being made aware of the event using the SAE CRF.

<u>All SAEs must be reported within 24 hours of the investigator being made aware of the event using the SAE CRF and the Investigator is responsible for providing follow up information for SAEs until resolution.</u>

Figure 5: Diagram to show notification/reporting period for AEs and SAEs occuring on LHRHa

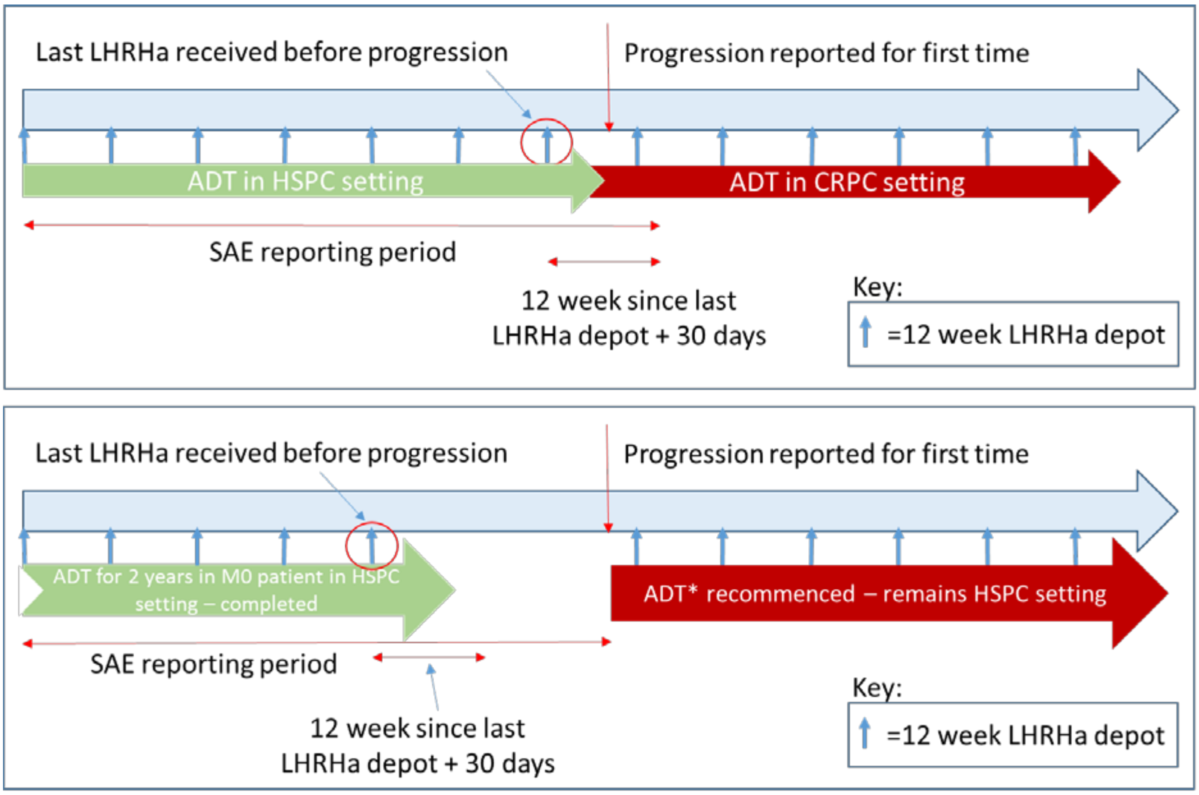

*Any additional treatment started at this stage eg: abiraterone would not be considered IMP

11.2.2 Trial-Specific "Expedited Reporting" Exemptions

The following events which may fulfil the definition of "serious" are exempt from expedited reporting. They are still require to be reported as an AE on the Toxicity (AE) CRF, or on an alternative CRF e.g. progression log, which will be used to report these events to the MHRA, but an SAE CRF is not required.

- **Death as a result of disease progression or disease-related deaths:** Do not complete an SAE CRF, unless death is considered to be caused by trial treatment (i.e. a SAR). The details should be reported on the Death Form.

- **Non-fatal progression events:** events that fulfil the definition of serious e.g. result in hospital admission, but are due to disease progression are exempt from reporting as an SAE, instead details should be provided on the Progression Log.

- **Elective hospitalisation** and surgery for treatment of locally-advanced or metastatic prostate cancer or its complications. These should be recorded as a non-trial inpatient admission on the follow-up form under Non-Trial visits.

- **Elective hospitalisation** to simplify treatment or procedures. If related to prostate cancer, record as non-trial inpatient admission on the follow-up form.

11.2.3 Investigator Assessment

11.2.3.A Seriousness

When an AE occurs the investigator or delegate **must** assess whether the event is serious. Refer to **Table 31** for what fulfils the criteria of serious and **Section 11.2** for a list of exemptions from expedited reporting.

11.2.3.B Grading severity of adverse event

The severity (i.e. intensity) of all AEs **must** be graded using Common Terminology Criteria for Adverse Events (CTCAE) v4.03. The complete CTCAE v4.03 can be found at:
http://www.stampedetrial.org/centres/tools-training/training-materials-resouces/
Any questions concerning this process should be directed to the CTU team in the first instance.

11.2.3.C Causality

The Investigator **must** assess the causal relationship of all serious events or reactions in relation to protocol treatment using the definitions in **Table 32**.

11.2.3.D Notification responsibilities for non-protocol treatments

It should be noted that ADT, docetaxel, abiraterone, enzalutamide and apalutamide may be given as non-trial treatments in the management of CRPC. It is not necessary to report AEs or SAEs relating to this non-trial use where the treatment commenced post progression. Instead the yellow card system should be used to notify the regulatory authorities of adverse drug reactions in this setting: (https://yellowcard.mhra.gov.uk/)

Figure 6: SAE reporting flowchart

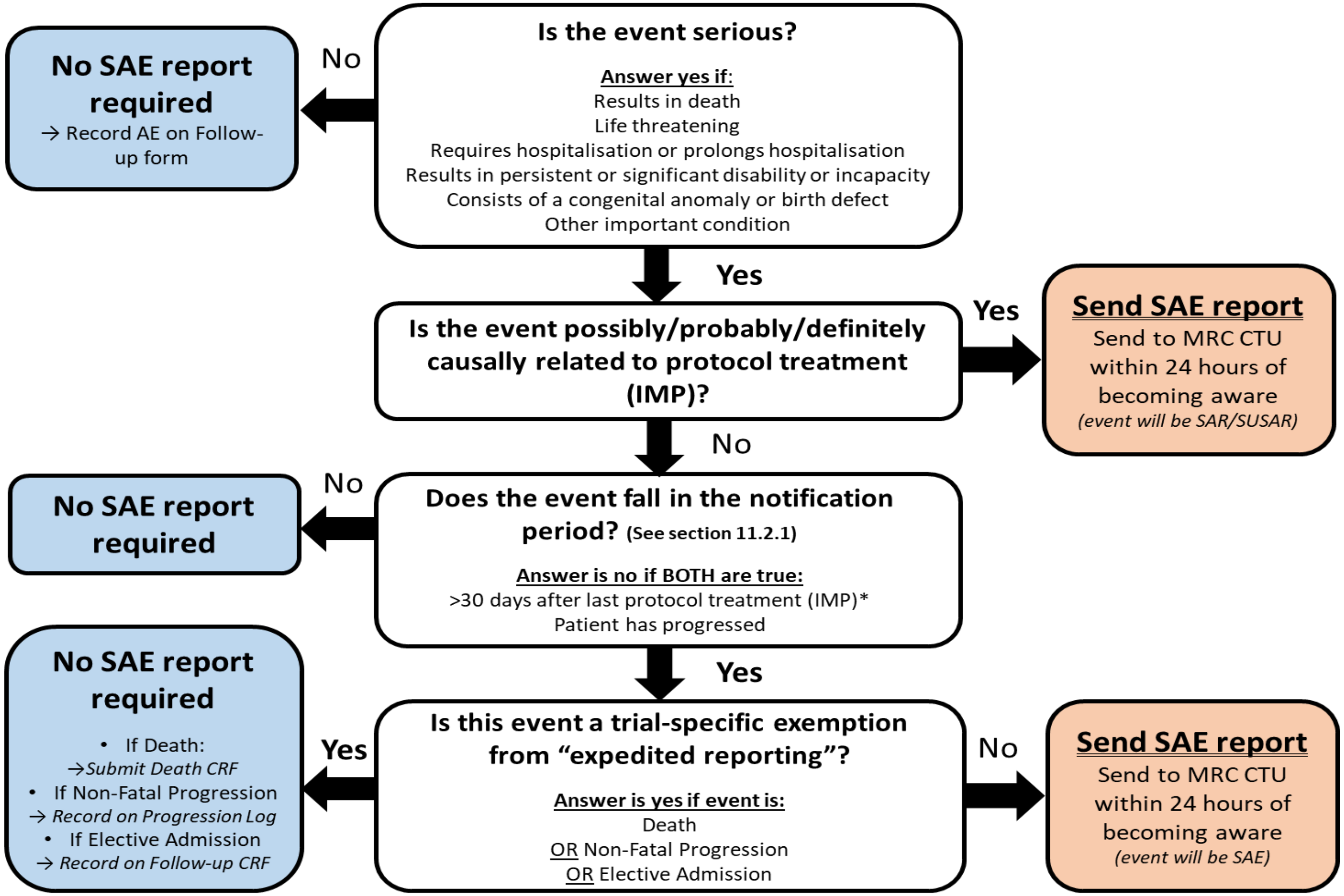

**Exposure to LHRHa is assumed to be until the depot expiration date, therefore unrelated SAEs are reportable up until 8 weeks after the administration of a 4-week depot or 16 weeks after the administration of a 12-week depot.*

Box 1: SAE report notification checklist

Before sending the SAE CRF please check that the event falls within the notification period and does not meet any of the "exemption from expedited reporting criteria", see Section 11.2. The SAE CRF must be submitted within 24 hours of an Investigator becoming aware of the event. The following are the minimum criteria required for initial processing and review:

1. At least **two** patient identifiers
2. **One event term** that can be coded to CTCAE version 4.03
3. Indication of why the event was **serious**
4. **Grade** severity of event/reaction according to CTCAE version 4.03
5. Date of **onset** when the event met the criteria of serious. Please refer to Table 31
6. Provide details for **all protocol treatments (IMPs) allocated** (i.e. both protocol SOC and protocol research treatments, including any allocated protocol research treatment that has not started) whether ongoing or completed at time of event onset.
7. Assessment of **causality** in relation to **each** protocol treatment (i.e.: both protocol SOC and protocol research treatments, including any allocated protocol research treatment that has not started) – *please note this can be provided later if clinician is not available within 24 hours of becoming aware of the event. This can be completed by the trial team based on correspondence with site clinician, and signed by the clinician at a later date.*
8. **Signature** (This can be a site trial team member in the first instance to meet the reporting timelines, but the CRF must be re-sent once a clinician has reviewed and signed the form)

SAE REPORTING
Fax to 020 7670 4818 within 24 hours of becoming aware of the event
Or send via **encrypted** email to mrcctu.stampede@ucl.ac.uk

11.2.4 Event Follow-up

Participants must be followed up until clinical recovery is complete or stabilised (resolution of the event – this can include an outcome that the event is "resolved with ongoing sequela"). Follow-up should continue after completion of protocol treatment if necessary. The Investigator is responsible for providing follow up information for SAEs until resolution. Follow-up information should be updated on the original SAE CRF by ticking the box marked "follow-up" and faxing to the CTU as information becomes available. Extra information and/or copies of test results may be provided separately but must be anonymised. The participant must be identified by trial ID and initials only. The participant's name should not be used on any correspondence.

11.3 CTU RESPONSIBILITIES

The STAMPEDE trial team will acknowledge receipt of all SAEs via email. Please contact the STAMPEDE trial team if an acknowledgement email is not received within 3 working days.

At least one medically qualified person at the CTU, or comparison chief-investigator or another appropriate TMG member will review all SAE reports received. The rationale for answers provided can be discussed between the site and CTU, however, ultimately the causality assessment given by the local Investigator at the hospital cannot be overruled.

The CTU is undertaking the duties of trial sponsor and is responsible for the reporting of SUSARs and other SARs to the regulatory authorities (through the MHRA to competent authorities in other European member states) and the UK research ethics committees. Additionally, the CTU has sponsor oversight for reporting in other countries in which the trial is taking place. The CTU is responsible for reporting fatal and life-threatening SUSARs to the UK competent authorities within 7 days of the CTU becoming aware of the event; other SUSARs must be reported within 15 days.

SAKK (Schweizerische Arbeitsgemeinschaft für Klinische Krebsforschung), the Swiss Group for Clinical Cancer Research coordinate site participation for STAMPEDE, and are responsible for reporting SUSARs to the relevant Swiss competent authority and lead ethics in accordance to their local regulations, on behalf of the CTU.

The CTU will also keep all investigators informed of any safety issues that arise during the course of the trial.

The CTU will submit Annual Safety Reports in the form of a Developmental Safety Update Report (DSUR) to Competent Authorities (Regulatory Authority and Ethics Committee).

Any drug companies involved will also be notified of reportable (serious and unexpected and drug-related/unknown relationship) events as per their agreement with the sponsor. CTU will also provide companies with a copy of the Annual Safety Report in the required format.

11.3.1 Sponsor (CTU) Assessment

11.3.1.A Expectedness

If there is at least a reasonable possibility of causal relationship to the protocol treatment (all IMPs i.e. SOC and research), an assessment of the expectedness of the event will be made by the Sponsor (the STAMPEDE team at the CTU). This determines whether a reaction is a SAR or SUSAR, see **Table 37**.

Expectedness is determined using the current reference safety information (RSI) (i.e. summary of product characteristics section 4.8 or current investigator brochure) approved for the trial. An event is considered unexpected if it is:

- Not listed in the RSI
- If severity exceeds that listed in the RSI
- If frequency exceeds that listed in the RSI
- If event outcome exceeds that listed in the RSI

Table 37: How causality and expectedness determine event outcome (SAE/SAR/SUSAR)

CAUSAL RELATIONSHIP (RELATEDNESS)	DESCRIPTION	EXPECTEDNESS ASSESSED BY CTU	
		EXPECTED REACTION	UNEXPECTED REACTION
Definitely	There is clear evidence to suggest a causal relationship and other possible contributing factors can be ruled out.	SAR	SUSAR
Probably	There is evidence to suggest a causal relationship and the influence of other factors is unlikely.		SUSAR
Possibly	There is some evidence to suggest a causal relationship (e.g. because the event occurs within a reasonable time after administration of the trial medication). However, the influence of other factors may have contributed to the event (e.g. the participant's clinical condition, other concomitant treatments)		SUSAR
Unlikely	There is little evidence to suggest there is a causal relationship (e.g. the event did not occur within a reasonable time after administration of the trial medication). There is another reasonable explanation for the event (e.g. the participant's clinical condition, other concomitant treatment).	Unrelated SAE<br>No assessment required as unrelated to treatment	
Unrelated	There is no evidence of any causal relationship	Unrelated SAE<br>No assessment required as unrelated to treatment	

12 ETHICAL CONSIDERATIONS AND APPROVAL

12.1 ETHICAL CONSIDERATIONS

12.1.1.A Randomisation

This is a randomised trial therefore neither the participants nor their physicians will be able to choose the participants' treatment. Treatment will be allocated randomly using a computer-based algorithm. This is to ensure that the groups of participants receiving each of the different treatments are as similar as possible.

All participants, with the exception of those allocated to transdermal oestradiol (Arm L), will receive standard hormone treatment. All participants, including those allocated to Arm L, may also receive other standard-of-care (SOC) treatments such as prostate radiotherapy and/or docetaxel, abiraterone, enzalutamide or apalutamide. Use of these SOC treatments will be unaffected by trial participation and is left to the discretion of the treating clinician and participant.

Participants may be randomised to receive additional treatment (metformin) given with standard-of-care treatments, or an alternative form of hormone treatment (transdermal oestradiol). An even allocation ratio is being currently being used which means all eligible participants have an equal chance of being randomised to the control or research arms.

Through the introduction of a "transdermal oestradiol comparison" into the STAMPEDE trial platform, sufficient data will be collected to evaluate this treatment approach more rapidly. By undertaking a meta-analysis using data collected in both PATCH and STAMPEDE trials, fewer participants overall are allocated the control arm i.e. more participants gain access to novel treatments and results will be available sooner.

12.1.1.B Evaluation of Novel Therapeutic Strategies

There is some evidence to suggest that the newer treatment options may have advantages over standard treatment alone with regards to clinical outcome, but this is not confirmed and toxicity may be increased. This trial will follow a large group of people who have been randomly allocated to either the standard treatment(s) or the novel treatment strategies in order to measure the benefits of these approaches. All participants will be followed-up for toxicity and safety issues, so that any benefits can be weighed against any negative aspects, including the impact treatments have on other aspects of medical health e.g. cardiovascular disease, as well as quality-of-life and value for money (health economic analysis).

12.1.1.C Additional Tests and Hospital Visits

Trial participants will have some additional hospital visits and some extra blood samples compared with standard practice, the exact requirements depend on the allocated treatment and stage of disease. Efforts are made to reduce the burden of extra visits and tests, for example extra blood tests can be performed at a time when a blood draw would be performed as part of standard care, or participants can have the blood samples taken at their GP's surgery instead.

12.1.1.D Facilitating Participant Feedback From Investigations and Additional Analyses

For participants who choose to take part in additional sub-studies, biological samples including blood, saliva and remaining stored FFPE tumour samples will be used in research projects. These projects will enable the study of genetic factors and other biomarkers that can help identify individuals who serve to benefit most from the treatments tested in STAMPEDE, and to further understand why and how treatment resistance develops. All samples will remain anonymised and only made accessible to approved collaborators granted access by the STAMPEDE oversight

committees. We will make every effort to protect the confidentiality of this information and make sure personal identities are protected.

From protocol v16.0 onwards, participants may opt to receive feedback regarding genetic results that may arise from the research analyses of genetic material extracted from any of the biological samples collected as part of the trial e.g. saliva, FFPE tumour blocks or circulating tumour DNA extracted from blood. Only results which are of established clinical relevance and for which testing would be available under standard NHS genetic testing guidelines will be fed back. Any genetic analysis undertaken as part of additional research associated with STAMPEDE does not replace clinically indicated investigations as only a proportion of STAMPEDE participants will undergo prospective testing and therefore it cannot be guaranteed that results will be fed back in a timely fashion.

This change has been made in response to emerging data that demonstrates a small proportion of people may have genetic faults in genes such as Breast Cancer Gene 2 (BRCA2). This has implications for both participants and potentially their biological relatives. For participants and their treating clinician, knowledge of this information may facilitate access into further clinical trials and may potentially impact on the choice of treatment following progression.

Any participant who consents to receive feedback and in whom a known pathogenic mutation of clinical significance is detected on testing of research samples collected as part of STAMPEDE will be told of this. Participants will be recommended to undergo genetic counselling accessed via clinical genetics services and consider confirmatory testing. This is necessary to determine if the defect is germline (inherited) and ensures access to appropriate ongoing support. If confirmed as a germline (inherited) abnormality, this will enable biological relatives to also access appropriate genetic counselling and testing if they wish.

The introduction of the “metformin comparison” means that all participants, not known to be diabetic, will be screened for diabetes prior to trial entry. This is to enable the effect of metformin to be studied in non-diabetic participants. All participants in whom screening bloods are abnormal will be referred for confirmatory tests and further management according to local guidelines e.g. via their GP. Screening is expected to lead to a small proportion of potential trial participants receiving a new diagnosis of diabetes but will ensure appropriate management of both conditions.

12.1.1.E Considering the Impact of Emerging Data

If new information emerges during the course of the trial which may affect the treatment or follow-up of participants all Principal Investigators (PIs) will be informed of this and required to inform trial participants.

12.1.1.F Electronic health records

Participants are requested to provide consent to permit linkage of trial data to other sources of electronic health data to improve the reliability of long-term follow-up data. Explicit consent is requested for the CTU to store direct identifiers (name and NHS number) securely and seperately from anonymised trial data. This is to permit verification of the information held by others and received by the CTU, ensuring that the trial database is only updated with accurate information.

12.2 ETHICAL APPROVAL

The protocol has a Favourable Opinion from an appropriate Research Ethics Committee, according to national guidelines. Additionally, each site must also obtain management permission for research (Local R&D approval or equivalent) from the relevant host organisations before participants can be entered into the trial. The participant’s informed consent to participate in the trial should be

obtained after a full explanation has been given of the treatment options, including the conventional and generally accepted methods of treatment. Participant information sheets and participant consent forms are available on the STAMPEDE website (www.stampedetrial.org).

The right of the participant to refuse to take part in the trial without giving reasons must be respected. After the participant has entered the trial, the clinician must remain free to give alternative treatment to that specified in the protocol, at any stage, if he feels it to be in the best interest of the participant. However, the reason for doing so should be recorded and the participant will remain within the trial for the purpose of follow-up and data analysis according to the treatment option to which he has been allocated. Similarly, the participant must remain free to withdraw at any time from the protocol treatment without giving reasons and without prejudicing his further treatment.

A statement of MRC policy on ethical considerations in clinical trials of cancer therapy, including the question of informed consent, is available from the MRC Head Office web site (http://www.mrc.ac.uk). In addition, the MRC and the Wellcome Trust framework on the feedback of health-related findings in research is readily available (https://www.mrc.ac.uk/documents/pdf/mrc-wellcome-trust-framework-on-the-feedback-of-health-related-findings-in-researchpdf/) and has been used when developing the trial specific processes.

13 REGULATORY APPROVAL

This trial has been approved in the UK by the MHRA and will be conducted under a Clinical Trials Authorisation CTA 20363/0404/001 in the UK.

The trial has been approved in Switzerland by Swissmedic (Ref: 2009 DR 3235).

13.1 DATA COLLECTION & RETENTION

CRFs, clinical notes and administrative documentation should be kept in a secure location (for example, locked filing cabinets in a room with restricted access) and held for 25 years after the end of the trial. During this period, all data should be accessible to the competent or equivalent authorities, the Sponsor, and other delegated authorities with suitable notice as it may be subject to audit or inspection from any of the above.

14 INDEMNITY

University College London holds insurance against claims from participants for injury caused by their participation in this clinical trial. Participants may be able to claim compensation if they can prove that UCL has been negligent. However, as this clinical trial is being carried out in a hospital, the hospital continues to have a duty of care to the participant of the clinical trial. University College London does not accept liability for any breach in the hospital's duty of care, or any negligence on the part of hospital employees. This applies whether the hospital is an NHS Trust or otherwise.

Participants may also be able to claim compensation for injury caused by participation in this clinical trial without the need to prove negligence on the part of University College London or another party. Participants who sustain injury and wish to make a claim for compensation should do so in writing in the first instance to the Chief Investigator, who will pass the claim to the managing organisation's Insurers, via the managing organisation's office.

Hospitals selected to participate in this clinical trial must provide clinical negligence insurance cover for harm caused by their employees and a copy of the relevant insurance policy or summary should be provided on request.

15 FINANCE

STAMPEDE is funded by Cancer Research UK's Clinical Research Committee (formerly the Clinical Trials Advisory Awards Committee; CTAAC). It is also funded by the MRC through the MRC Clinical Trials Unit at UCL. The trial has National Institute for Health Research Clinical Research Network (NIHR CRN) approval and, therefore, local NCRN funds may be available at each site to support entry of participants into this trial.

Funding arrangements for research arms and sub studies now closed to recruitment can be found in earlier protocols.

Standard therapies including **ADT**, **prostate radiotherapy** and **docetaxel** will be administered as per routine clinical care using local NHS supplies.

Abiraterone is manufactured by Janssen Pharma PV (pharmaceutical companies of Johnson & Johnson). They have agreed to provide free drug, funds to distribute drug to participating sites and to help support the conduct and management of the trial.

If abiraterone is required to be given to participants as standard of care, funding will not be provided and the drug should be administered as per routine clinical care using local NHS supplies.

Enzalutamide is manufactured by Astellas Pharma. They have agreed to provide free drug and funds to distribute drug to participating sites and to help support the conduct and management of the trial.

If enzalutamide is required to be given to participants as standard of care, funding will not be provided and the drug should be administered as per routine clinical care using local NHS supplies.

Metformin will be administered using local NHS supplies.

Transdermal oestradiol will be administered as either Progynova TS 100 patches, manufactured by Bayer, or Femseven 100 patches, manufactured by Theramex who have agreed to supply these patches at a trial-specific discounted price. All accredited STAMPEDE sites will be able to order Progynova for use in the STAMPEDE trial through Alliance Healthcare Ltd wholesalers and Femseven patches through AAH Pharmaceuticals Ltd wholesalers.

Apalutamide given to participants as standard of care will be administered using local NHS supplies.

16 TRIAL COMMITTEES

16.1 TRIAL MANAGEMENT GROUP (TMG)

A Trial Management Group (TMG) has been formed comprising: the Chief Investigator; each comparison lead investigator; other co-investigators and members of MRC CTU at UCL internal Trial Management Team. The membership of the TMG may be expanded if other groups of trialists wish to participate. It will also be amended during the trial if other circumstances require e.g. retirement.

The TMG will be responsible for the day-to-day running and management of the trial. The TMG will meet by teleconference at least on a monthly basis where possible and in person as needed.

Further details of TMG functioning are provided in the TMG charter (available on request).

16.2 TRIAL STEERING COMMITTEE (TSC)

A Trial Steering Committee (TSC) has been formed to provide overall supervision for the trial and provide advice through its independent chair. The ultimate decision for the continuation of the trial lies with the TSC. The TSC will meet regularly, as required by the trial, and at least annually.

The relationship of the TSC with the other STAMPEDE working groups is detailed in **Figure 7**. Further details of TSC functioning are provided in the TSC charter (available on request).

16.3 INDEPENDENT DATA MONITORING COMMITTEE (IDMC)

An Independent Data Monitoring Committee (IDMC) has been formed. The IDMC will be the only group who sees the confidential, accumulating data to the trial. Reports to the IDMC will be produced by the CTU. The IDMC will meet within 6 months of the trial opening with the frequency of meetings dictated by the IDMC. The IDMC will consider data in accordance with the analysis plan (see **Section 9**) and will be advisory to the TSC. The IDMC can recommend premature closure or reporting of the trial, or that recruitment to any research arm is discontinued.

From protocol v8.0 onwards, any recommendation from the IDMC to stop recruitment to one or more trial arms will be acted upon immediately, pending ratification from the TSC. As this period between meetings should be very short, sites would not be notified until after the TSC have made a decision. IDMC recommendations based on emerging safety issues will be discussed with sites promptly.

The relationship of the IDMC with the other STAMPEDE working groups is detailed in **Figure 7**. Further details of IDMC functioning and the procedures for interim analysis and monitoring are provided in the IDMC charter (available on request).

Data from the "transdermal oestradiol comparison" are viewed by the PATCH IDMC, in meta-analysis with PATCH, rather than by the STAMPEDE IDMC. Recommendations of any actions relating to STAMPEDE would be made to the STAMPEDE TSC.

16.4 TMG SUB-GROUPS AND EXPERT PANELS

The trial has a number of TMG sub-groups and expert panels, each comprising of specific members of the TMG, MRC CTU at UCL, field experts and other STAMPEDE clinicians and site staff. The groups are all chaired by TMG members and report directly into the TMG.

- The **Biological Research Group** (BRG), the **Bone and Imaging Group** (BIG) and the **Metabolic Translational Group** (MTG) all input and provide expert oversight of relevant translational aspects of the trial and associated sub-studies.
- The **STRATOSPHere Consortium Management Group** (STRATOSPHere: STratification for RAtional Treatment-Oncomarker pairings of STAMPEDE Participants starting long-term Hormone treatment) coordinates the parallel translational programme funded by Prostate Cancer UK.
- The **Comparison Management Groups** (CMGs) were developed to input into the running of each comparison and to propose, plan and develop new comparisons as required. The CMGs are comprised of:
    - Arm G – Abiraterone CMG
    - Arm H – M1|RT CMG
    - Arm J - Abiraterone and Enzalutamide CMG
    - Arm K – Metformin CMG
    - Arm L – tE2 CMG
    - Future proposals CMG
    - Original comparisons CMG
- The **Site Advisory Team** (SAT) includes STAMPEDE site research staff to provide advice to the TMG concerning the running of the trial, including how proposed amendments to the protocol and CRFs directly affect staff practices.
- The **Outcome Review Group** (ORG) conducts cause of death reviews as required for secondary end point analysis.
- The **Clinical Safety Committee** (CSC) review all SAEs of STAMPEDE participants and provide guidance to site clinicians and research staff in regards to clinical safety aspects of the trial.
- The **Genetic Sub-Group** (GSG) provides oversight of all results arising from genetic testing.
- The **Quality of Life** group (QOL group) will advise on how to optimise use of QOL data within the STAMPEDE trial

The relationship of each of these groups with the other STAMPEDE working groups is detailed in **Figure 7.**

16.5 MRC CTU AT UCL INTERNAL GROUPS

CTU requires a number of internal working groups to run a platform protocol. These internal groups assist the TMG in the operation of STAMPEDE, providing guidance on scientific strategies of research and publication, research governance in regulatory information and protocol review and the management of research quality within the STAMPEDE trial.

The relationship of each of these groups with the other STAMPEDE working groups is detailed in **Figure 7**.

Figure 7: Organigram of the relationships between STAMPEDE working groups

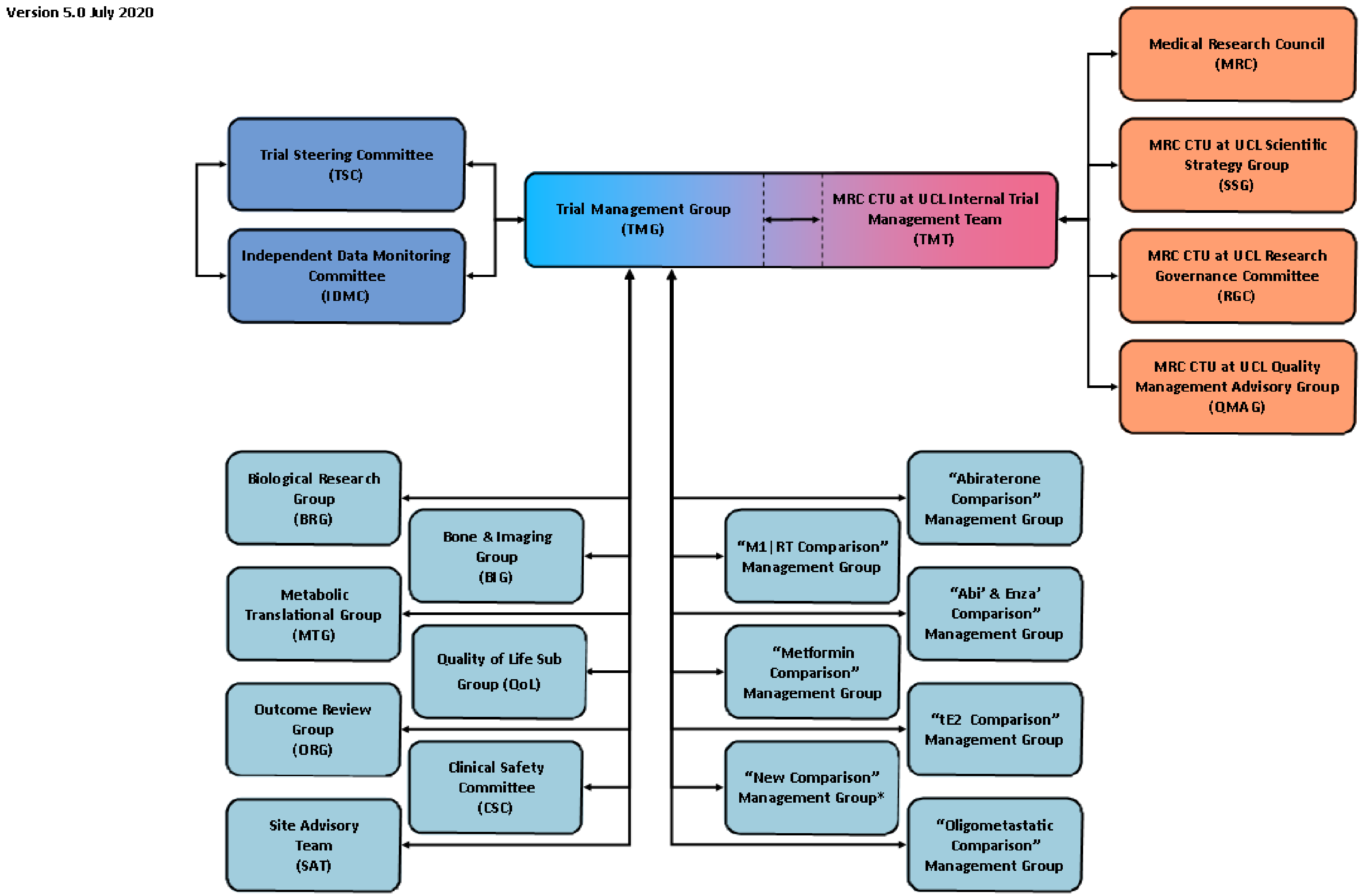

*The number and timeline of current and planned comparisons will dictate the need for the number of CMGs in operation. At any point there may be one or more.

17 ANCILLARY STUDIES

17.1 PATIENT REPORTED OUTCOMES

STAMPEDE collects patient reported outcomes in the form of the EORTC QLQ-30 Quality of Life form and the EQ-5D Health Economics Form.

The research nurse should approach participants at appropriate clinical visits to complete a questionnaire. If no clinical visit is scheduled for the participant (with a window of 4 weeks around the expected date) the nurse should organise the completion of the questionnaire e.g. by post or secure e-mail.

Questionnaires should be self-administered; participants should be encouraged to complete the questionnaires without conferring with friends or relatives and all questions should be answered even if the participant feels them to be irrelevant. The research teams should encourage the participants to answer all questions but should not review the responses as these should remain confidential. Copies of questionnaires should not be retained at site.

17.1.1 Quality of life (QL)

The EORTC QLQ-C30 with the prostate-specific module QLQ PR25 will be used. Key items for assessment are pain reduction for participants with metastatic disease and urinary symptoms for participants with locally-advanced disease. In addition, specific hypotheses will be generated for each of the research arms.

17.1.1.A Changes in QL data collection from protocol v19.0 onwards

Initial participation in the QL sub-study was limited to the first 700 participants recruited (this was reached in Sep-2008). After a pause, the QL sub-study re-opened from the implementation of protocol version 8.0 (Nov 2011 onwards).

From protocol v19.0, QL and HE data collection changed, as laid out in **Table 33.** QL and HE collection stopped for most participants but continued as planned in participants in the “abiraterone comparison” and “abiraterone and enzalutamide comparison” and became lifelong in participants in the “metformin comparison”. HE collection (without QL) also continued in participants in the “M1|RT comparison” randomised after Apr 2016.

Table 33 summarises the participant reported outcome data collection (QL and HE) by comparison. Going forward, for each new comparison within STAMPEDE, a pre-defined sample size for the participant reported outcomes will be described and a sampling approach considered where appropriate.

17.1.2 Health Economics

The EuroQol (EQ-5D) will be used in the study as a generic measure of health-related quality-of-life which can be linked to public preferences. This data will be used to calculate quality-adjusted life-years as part of the economic evaluation. Healthcare resource use will be collected at each follow-up. This includes non-trial inpatient days, non-trial outpatient, GP visits and data on concomitant medications. Information on participants’ use of primary care and community-based services will also be collected as additional questions in the questionnaire. Costs will be calculated on the basis of representative UK unit costs at the point of analysis. A cost-effectiveness analysis will compare all regimens that continue to recruit into their final Efficacy Stage. For further details please refer to **Appendix G**.

Table 38: Patient reported outcome data collection by comparison

COMPARISON	PARTICIPANT DETAILS	COLLECTION OF PATIENT REPORTED OUTCOMES<br>E.G. EORTC QLQ-C30, EQ-3D
"Original"	Arms B, C, D, E, F and Arm A recruited between trial start (2005) and 15-Nov-2011	No further collection of participant reported outcomes as comparisons have closed to follow-up
"Abiraterone"	Arms A and G randomised between 15-Nov-2011 and 17-Jan-2014	Data collection to continue until disease progression or 5 years post randomisation (i.e. all data collection stops 17-Jan-2019).
"Abiraterone and enzalutamide"	Arms A and J randomised between 29-Jul-2014 and 31-March 2016	Data collection to continue until disease progression or 5 years post randomisation whichever occurs first.
"M1|RT"	Arms A and H randomised between 22-Jan-2013 and 02-Sep-2016	From protocol v19.0 QL and HE data collection will stop for all participants recruited to Arm H prior to April-2016.<br>From protocol v21.0 HE (EQ-5D) data collection will stop along with active follow up for all A and H participants randomised between Apr-2016 to Sep-2016.
"Metformin"	Arms A and K randomised since 05-Sep-2016	From protocol v19.0 the QL and HE sub-studies are closed to newly randomised participants.<br>For all existing arm A and K participants (i.e. randomised prior to activation of protocol v19.0) data collection continues at each follow-up lifelong.
"Transdermal oestradiol"	Arms A and L randomised since 20-Jun-2017	From protocol v19.0 the QL and HE sub-studies are closed to newly randomised participants within this comparison. QL data will be collected through the PATCH trial.

17.2 TRANSLATIONAL SUB-STUDIES

Samples obtained from consenting STAMPEDE participants are analysed as part of separate translational sub-studies. These are conducted through collaborations with other academic and industry partners. All applications for collaboration and sample access are reviewed by the STAMPEDE oversight committees and overseen by the STAMPEDE BRG. For details on eligibility criteria for each translational sub-study refer to **Section 4.7.** For details regarding sample collection refer to the **Sample collection and handling manual** available via the website.

17.2.1 Germline DNA Analysis

DNA is being extracted from saliva samples provided by consenting participants enrolled in STAMPEDE. The purpose of this sub-study is to examine the germline (inherited) genetic changes present in people with high-risk localised or metastatic prostate cancer. The aim is to determine the prevalance of germline genetic aberrations present pre-diagnosis and to correlate prostate cancer risk single-nucleotide-polymorphisms (SNP) genetic profiles, identified in Genome-wide Association Studies (GWAS) and other sequence variants from next generation sequencing (NGS), with duration of response to ADT and the experimental treatments tested in STAMPEDE.

All newly randomised trial participants who join arms A, K or L are eligible to join this sub-study. For details relating to Saliva sample collection and shipping refer to the **Sample collection and handling manual.**

17.2.2 Circulating Tumour-DNA Analysis (Sequential Blood Samples)

The aims of this analysis include identification of molecular subgroups with differential treatment effects and, through sequential sampling, identification of molecular changes associated with disease progression to explore resistance mechanisms and early detection of treatment failure.

Sequential samples are required in order to detect genetic changes within tumours over time. The most important sampling timepoint is at progression, as it is hoped this can inform the potential mechanisms of treatment resistance. The sampling schedule is different for M0 and M1 participants and is detailed in the **Sample collection and handling manual**.

For details relating to blood sample collection including eligibility criteria and shipping refer to the **Sample collection and handling manual.**

17.2.3 Tissue Sample Analysis (FFPE Blocks)

As the clinical outcome data matures for several of the treatments comparisons evaluated within STAMPEDE, correlative analysis of the archival formalin-fixed paraffin-embedded (FFPE) tumour tissue will be undertaken, aiming to identify if genetic mutations present in prostate cancer cells pre-treatment predict how well each treatment works. In addition, projects providing preliminary prevalence and feasibility data to inform future biomarker-directed randomisations will be conducted.

From 2016 onwards, the CTU has been coordinating the retrieval of archival tumour blocks from selected consenting STAMPEDE participants. These samples are usually stored as FFPE tissue blocks at the hospital where the procedure was performed. Randomising sites will be asked to assist in the retrieval of FFPE samples when these are requested. Research teams will be required to confirm sufficient consent has been provided and to provide an anonymised copy of the relevant consent form. If not done so already, an anonymised copy of the consent form should also be sent to the CTU, as per **Section 10.1.1.**

For further details on where to check sufficient informed consent, sample processing and shipping and reimbursement, see the **Sample collection and handling manual.**

17.2.4 Biomarker-Screening Pilot

A biomarker-screening pilot was conducted in a subset of STAMPEDE sites. This ran from Dec 2017 to Jun 2018 and has now been completed.

17.2.5 Informed consent to receive results arising from genetic sub-studies

The consent process was updated for participants joining the trial from protocol v16.0 onwards (activated June-2017). Trial participants are asked to provide explicit informed consent if they wish to receive feedback of any results that arise from research analyses of genetic material extracted from any of the biological samples collected as part of the trial e.g. saliva, FFPE tumour blocks or circulating tumour DNA extracted from blood.

Only results which are of established clinical relevance and for which testing would be available under standard NHS genetic testing guidelines will be fed back e.g. pathogenic BRCA2 mutations. Any genetic analysis undertaken as part of additional research associated with STAMPEDE does not replace clinically indicated investigations as only a proportion of STAMPEDE participants will undergo prospective testing. Analyses are conducted on a purely research basis and it cannot be guaranteed that results will be fed back immediately.

STAMPEDE investigators are strongly recommended to refer all participants in whom a clinically relevant genetic result is detected during research analyses to a clinical geneticist. This is to facilitate access to genetic counselling and the required confirmatory testing. This is also necessary in order to offer appropriate advice to biological relatives in the event of confirmatory testing detecting a germline (inherited) abnormality. The list of clinically relevant gene mutations to be fed back will be based on current clinical guidelines. The STAMPEDE Biological Research Group will review this periodically to ensure it remains current and to oversee this process.

Information provided to STAMPEDE participants who joined the trial prior to Protocol version 16.0, stated that any subsequent genetic results would not be linked to them or their families and therefore results will not be provided in this instance. It is possible for trial participants to update their consent by re-consenting to the current Additional Research Consent Form. This should be anonymised and sent to the CTU as per standard procedures, see **Section 10.1.1.**

17.3 DISEASE VOLUMETRIC ANALYSIS SUB-STUDY

Baseline imaging obtained from STAMPEDE participants are accessed and analysed as part of the trial data collection. Collection and analysis will be undertaken in collaboration with partners on the TMG, initially, in ordered to determine disease volume. For details partaking to retrospective imaging centralisation and image handling, please refer to the individual sub study Working Practices available from the CTU. All subsequent applications for collaboration and imaging access are reviewed by the STAMPEDE oversight committees following the usual processes.

17.4 USING ROUTINE DATA TO IDENTIFY CLINICAL TRIAL OUTCOMES

This sub-study is developing methods to explore whether routine data can be used to quickly and accurately capture trial-related events in centrally-held datasets. These methods need to be developed and validated using different sources of routine data and to identify different types of events. These data sources include, but are not limited to, data from the Public Health England (PHE) National Cancer Registration and Analysis Service (NCRAS), the National Radiotherapy dataset (RTDS)

and the Systemic Anti-Cancer Therapy dataset (SACT) and NHS Digital, Hospital Episode Statistics (HES), and Office of National Statistics (ONS) data.

The overall aim of this sub-study is to develop a clinically useable tool, to accurately identify disease driven events and trial outcomes, to help reduce the burden of collecting trial data from traditional participant-investigator contact. By using data that has already been accurately collected in patients that have given appropriate consent, or for whom the appropriate permissions are in place (e.g. via the Confidentiality Advisory Group), it may be possible to improve timeliness, reduce costs and save resources. Development of enhanced ways to obtain trial data is being undertaken, to recalculate analyses already carried out but also to perform secondary analyses not possible with conventionally collected trial data. The projected aim is to utilise validated methods for routine follow-up and/or analysis in the future, as outlined in the protocol, longer term outcome data may be sought via routine data sources.

17.5 METFORMIN METABOLIC SUBSTUDY

The aim of this project is to explore the heterogeneity of metabolic changes associated with ADT and the effect that metformin has on these changes. Multiple blood markers of metabolic and disease status and sarcopenia assessed by cross-sectional imaging will be examined and linked with baseline characteristics and clinical outcomes.

ADT is standard of care for patients with advanced prostate cancer. It is effective but has side effects, one of them being metabolic dysfunction including obesity, sarcopenia, hyperinsulinemia, and insulin resistance. We will assess whether metformin will alter the percentage of patients with a poor prognostic lipid signature. We will explore whether adding metformin improves oncological outcomes through metabolic reprogramming of the host. In addition we want to determine whether the side effects of ADT can be mitigated by metformin, thus potentially decreasing cardiovascular morbidity and mortality. If we find a decrease in sarcopenia by adding metformin this will also be of importance since sarcopenia does not only affect the self-esteem of men, but also correlates with increased morbidity and mortality.

If metformin is associated with an improvement in metabolic parameters or sarcopenia, it could change clinical standard of care very rapidly, even independently of a benefit in cancer-specific or overall survival.

From protocol v21.0 we will collect sequential blood samples at baseline, regular time points throughout the trial and at progression. In addition we will request CT scans and FFPE tissue blocks from sites. The metabolic sub-study will be for participants allocated to the “metformin comparison”.

For eligibility criteria for the metabolic sub-study refer to **Section 4.7.4**.

For details relating to blood sample collection and shipping, please request the **Metabolic Sub-study Sample Collection and Handling Manual** from the CTU.

18 PATIENT AND PUBLIC INVOLVEMENT

Patient and Public Involvement (PPI) in research is defined by INVOLVE (an advisory group established by the NIHR) as research being carried out ‘with’ or ‘by’ members of the public rather than ‘to’, ‘about’ or ‘for’ them. INVOLVE intends ‘public’ to include patients, potential patients, carers and other users of health and social care services, as well as people from organisations that represent people who use services. In some cases, this may include involvement of a trial’s participants in guidance or oversight of a trial.

18.1 POTENTIAL IMPACT OF PPI

PPI is in place to have constant patient overview and investment to guide research. Ultimately STAMPEDE has been created to test whether alterations in treatment help to improve outcomes and quality of life of patients. It is essential to have patients’ input as they understand what other patients are going through

The nature of STAMPEDE is such that, even after a main analysis of a comparison has been performed, other participants are still being recruited to other arms. We have a duty to participants and the public to disseminate findings and results, both negative and positive. With this in mind, participants are periodically provided with study findings and updates. Study findings are also presented at conferences.

18.2 PATIENT REPRESENTATIVES

Patient representatives are actively involved in the management of STAMPEDE including updates and alterations. Part of their role is to review all material that will enter the hands of a patient or family member. This is to ensure all documentation used is clear, concise and has wording that is appropriate for everyone, as well as conveying the intended information. Patient representatives sit on the Trial Management Group (TMG).

19 PUBLICATIONS

The results from different sites will be analysed together and published as soon as possible. Individual clinicians must not publish data concerning their participants that are directly relevant to questions posed by the study until the TMG has published its report. The TMG together with the STAMPEDE collaborators will form the basis of the writing committee and decide on the nature of publications.

For the “transdermal oestradiol comparison”, as the efficacy analyses will based on relevant data from the STAMPEDE and PATCH trials, TMGs for the two studies will form the writing committee. Any release, of efficacy or safety data, presentation or publication will be agreed with the TSC according to the terms of their charter.

All publications will acknowledge the participating sites and clinicians, and these will be detailed in an appendix to the main report. Papers will have named authors determined by the TMG according to the following principles:

- To be as inclusive as possible where this is practicable
- To ensure that there is justification for anyone to be named as an author
- Reasons for nomination for authorship may include: trial design; grant holding; day-to-day trial oversight (TMG membership); analysis; discussion and interpretation of data; representation for key groups; active participation at large recruiting sites.
- It should be accepted that the people qualifying for authorship will vary over time. In addition, key positions will vary depending on the nature of the publication: clinical lead for clinical papers, statistician lead for methodology papers, translational papers may be led by authors not on the main TMG if appropriate (e.g. the bone sub-study). In the event of any dispute related to authorship or data release, the TSC will be responsible for making the executive decision.

In the presentations, this list of sites will also be shown. The term “the STAMPEDE investigators” will clearly be stated and relevant names included in the presentation credits.

A detailed **Publication Plan** is documented separately.

20 DATA AND/OR SAMPLE SHARING

Data will be shared according to the CTU's controlled access approach and Standard Operating Procedure, based on the following principles:

- No data should be released in response to a data release request that would compromise an ongoing trial, unless specifically for safety reasons.
- There must be a strong scientific or other legitimate rationale for the data to be used for the requested purpose.
- Investigators who have invested time and effort into developing a trial or study should have a period of exclusivity in which to pursue their aims with the data, before key trial data are made available to other researchers.
- The resources required to process requests should not be under-estimated, particularly successful requests which lead to preparing data for release. Therefore adequate resources must be available in order to comply in a timely manner or at all, and the scientific aims of the study must justify the use of such resources.
- Data exchange complies with Information Governance and Data Security Policies in all of the relevant countries.

Data will be available for sharing on successful request and after the main publication for each comparison. Researchers wishing to access STAMPEDE data should contact the TMG via the CTU team in the first instance. All requests must be reviewed and approved by the TMG and TSC prior to release of data. Investigators should in term ensure the CTU team are regularly updated on the progress of their project and any presentation and publication must be in accordance to the agreements in place.

21 PROTOCOL AMENDMENTS

21.1 PROTOCOL

21.1.1 Amendments Made To Protocol Version 1.0 (May-2004)

Administrative changes such as typos, word change etc.

Name additions/changes to:

TMG members

TSC members

IDMC members

'General Information' Section – additional information re. Abridged version of protocol

Section 1.2 – Figure 1, Celecoxib duration amended

Section 1.3 – Figure 2, addition of cardiovascular assessment form, name and timings amended

Section 2.3 – Docetaxel information updated

Section 2.4 – Additional text re dose and duration justification for Celecoxib use.

Section 3 – Title change and content updated

Section 4.2 – New exclusion criteria added

Section 4.3.1 – New investigations added and additional text re testosterone measurements and additional text re. prior celecoxib treatment

Section 6.1.4 – Celecoxib duration amended

Section 6.1.5 – Additional text re. Co-administration of docetaxel and bisphosphonates

Section 6.1.6 – Celecoxib duration amended

Section 6.2.2 – additional docetaxel information

Section 6.2.3 – addition of CVS event history

Section 11 – Safety reporting updated

Section 12.1 – Additional text re. the collection of blood for genetic and serum marker studies

Section 15 – Additional information re. Central Subvention for docetaxel arms

21.1.2 Amendments Made To Protocol Version 1.1 (May-2005)

Section 6.2 Administration and Dose Modifications, subsection 6.2.1 Zoledronic Acid

21.1.3 Amendments Made To Protocol Version 2.0 (Jun-2005)

General Information section – SAE reporting fax number and timeframe added.

Section 1.2 – Addition of anti-androgen use for M0 patients as a method of HT

Section 1.2 – Increase in amount of blood needed & addition tissue sample request.

Section 1.3 Trial Documentation updated to include new table detailing trial documentation ahead of accreditation, the inclusion of the radiotherapy forms and correct case report form timings

Section 2.1 – Addition of anti-androgen use for M0 patients as a method of HT

Section 4.1.3 – Inclusion criteria Vii "Normal testosterone prior to hormone treatment" removed.

Section 4.1.3 - φnote has been omitted and moved to section 4.2 (see number 8)

Section 4.2 – Exclusion criteria added to exclude patients with active peptic ulceration, gastrointestinal bleeding and inflammatory bowel disease.

Section 4.2 – Exclusion Criteria added to exclude patients with planned major dental work

Section 4.3.1 - All blood test timelines changed from 14 days to 28 days.

Section 4.3.1 – Hormone Therapy pre-randomisation deadline extended from 4 weeks to 12 weeks.

Section 4.3.1 – Additional information regarding the use of NSAIDs and cox-2-inhibitors before coming on to the STAMPEDE study and once commenced on study treatment

Section 4.3.2 – Updated to ask for all vitamins and minerals the patient is taking to be recorded.

Section 4.3.3 – Updated to include the extra blood required and the request for consent of patients' tissue samples.

Section 6.1.1 – Addition of anti-androgen use for M0 patients as a method of HT

Section 6.1.6 – Addition of the calcium & vitamin name "calcichew".

Section 6.6.2 – asking also to collect vitamins and minerals under concomitant medication.

Section 6.6.3 – New section to inform investigators that patient's, who they wish to give radiotherapy to, are also eligible for STAMPEDE

Section 6.6.4 – New section to detail what data is being collected on the radiotherapy given to patients.

Section 7.1; figure 4 – Addition of radiotherapy form and in note, addition of AA alone

Section 7.1.2 – omission of repeated scans and x-rays at 24 weeks, also omitted in note under figure 4.

Chapter 11 – Safety reporting section updated

Section 17.3 – Increase in amount of blood needed & additional tissue sample request.

21.1.4 Amendments Made To Protocol Version 3.0 (Jul-2006)

Front Cover - NCRN logo added for accuracy

Front Cover - Clarification that protocol developed with NCRI rather than on behalf of

Front Cover - Clarification that it is a 6 arm trial

General Information section - MRC CTU staff section updatedyyyy

Section 1.2 – Statistics section updated.

Section 1.2 - Additional research paragraph updated to reflect additional studies and for clarification of terms

Section 1.2 - Blood collection volume changed to reflect new technique used

Section 1.3 (figure 3) - Table showing case report form schedule updated to reflect clarification of follow-up schedule and addition of new CRF (End of Treatment)

Section 2.2 - AS changed to HT (clarification of terms)

Section 2.3 - Updated in information in regard to use of docetaxel added to reflect up to date practice

Section 2.5 - Sub-headings numbered for consistency

Section 3.0 - Information in regard to the Pilot Phase now written in past tense as Pilot Phase has now been completed

Section 4.1.1 - Inclusion criteria extended so that patients who fulfil 2 out of the three of the first inclusion criteria can be eligible.

Section 4.3.1 - Change in time scales by which baseline investigations need to be completed.

Section 4.3.1 - Clarification that chest X-ray is only required if chest is not included in the CT

Section 4.3.1 - Removal of 12 week timeline for baseline PSA test to be performed. (Stipulation that it must be performed before start of HT)

Section 4.3.2 – Information added in regard to time allowed from randomisation to start of treatment

Section 4.3.3 - Additional research paragraph updated to reflect additional studies and for clarification of terms

Section 4.3.3 - Blood collection volume changed to reflect new technique used

Sections 6.1.2-6.1.6 - Androgen Suppression replaced with hormone therapy for consistency of terms

Section 6.2.2 - '(Taxotere)' Removed for consistency

Section 6.2.2 _ information added in regard to the need to closely monitor liver function prior to docetaxel administration

Section 7.1 - Page number reference updated

Section 7.1.1 - PSA measurement timings updated to accurately reflect follow-up schedule

Section 7.3 (Table 4) - Table and key updated to accurately reflect follow-up schedule and to include information about new CRFs and removal of withdrawal CRF

Section 8 - Rewording for clarification of definition of trial withdrawal

Section 8.1 - Instruction that withdrawal from trial treatment should be recorded on End of Treatment Form rather than withdrawal form

Section 8.1 - Information updated to emphasise that trial treatment must be discontinued following a progression

Section 8.2- Information added in regard to patient transfers

Section 8.3 - Instruction that withdrawal from trial completely must be notified in writing to the MRC CTU rather than included on withdrawal form

Section 9 and Summary – Target event numbers updated to reflect the slightly revised numbers obtained by using –nstage- which is the new, recommended program for MAMS trials

Sections 11.1 and 11.2 - Form numbers removed to allow for future changes in numbering

Section 11.2 – Reference to toxicity grading website added

Section 11.2.1 - Reference to table in appendix G added

Section 12.2 - 'Suggested' removed from 'Suggested patient information sheets'

Section 13 - CTA reference added

Section 17.3 - Information added to reflect new blood collection method for DNA analysis and in regard to additional translational studies for which funding has recently been approved

21.1.5 Amendments Made To Protocol Version 4.0 (Dec-2007)

General Information Section - Randomisation and SAE reporting details sections clarified

Section 1.2 and throughout protocol - Efficacy Stages 1-111 renamed to Activity Stages 1-111 for accuracy and clarity

Section 1.2 - Follow schedule corrected

Section 4.1.2 - Inclusion criteria widened to include high risk relapsing patients, that would not have met the previous PSA based criteria

Section 4.1.3 - Note added to reference location of WHO performance status definitions

Section 4.2 - Notes added to reference locations of toxicity gradings and NYHA classifications

Section 4.3.1 - Timings of baseline scan information changed to accurately reflect most common current practice

Section 6.1.1 - Information about use of LHRH antagonists to ensure that the protocol accurately reflects current and future practice

Section 6.1.1 - Information about suggested duration of hormone therapy added to ensure that the protocol accurately reflects current practice

Section 6.2.2 - Additional information added about the timing of liver function tests prior to docetaxel administration added for clarity

Section 6.6.4 - Information on radiotherapy data collection added

Section 7.1.1 - Erroneous information about the timing of PSA measurements removed

Figure 3 - Moved to new section in protocol for clarity and extended to include current information on data collection

Figure 3b - Added to describe how extent of data collection during follow-up should change, post treatment and post progression

Figure 4 - Notes added to explain the changes in data collected at follow-up and to information that the quality-of-life study will be applicable to the first 700 patients randomised only

Figure 4 - Note added to include palliative radiotherapy CRF

Section 11.3 - SAE reporting information updated

Section 19 - Protocol amendments list updated

21.1.6 Amendments Made To Protocol Version 5.0 (Aug-2008)

1. General Information Section – Randomisation phone line number updated – non UK extension added
2. Section 3 – Information about QL study removed to reflect closure of QL study after first 700 patients
3. Section 4.2 – Exclusion criteria clarified to explain that only patients with severe poor cardiovascular history should be excluded
4. Section 4.3.1 – Information on co-administration of NSAIDS with celecoxib changed based on clinical advice.
5. Section 5 - Randomisation phone line number updated – non UK extension added
6. Section 6.2.1. – Information added to clarify that patients who develop an osteonecrosis of the jaw should stop zoledronic acid treatment
7. Section 6.2.3 – 'severe' text added to accurately reflect which patients should be excluded based on their cardiovascular history
8. Section 7.1.2 – Definition of disease progression extended for clarity
9. Figure 3 – Updated to include reference to newly created skeletal related event form

10. Figure 4 – Previous error in table amended to show that the 4th Zoledronic Acid form that is submitted contains information about 3 cycles rather than 2 as previously indicated

11. Table 4 – 'Other important medical condition' added to definition of serious in the SAE section, to accurately reflect SAE form and current practice

12. Section 11.1 – Information added on reporting or pregnancies

13. Section 17 - Information about QL study removed to reflect closure of QL study after first 700 patients

21.1.7 Amendments Made To Protocol Version 6.0 (Jul-2009)

1. General Information Section – Trial Pharmacist removed and changes of:

Co-Investigator

Patient Representatives

Trial Manager

Data Manager

General Information Section - Coordinating Centre – address change

General Information Section – change of Sponsor address

Section 1.1 – ratio of patients randomised to the investigational arms updated

Section 1.2 – figure 1b added to clarify trial design from Apr-2011 onwards

Section 1.2 – paragraph added to explain trial changes after the second activity analysis

Section 1.2 – wording added to clarify that QL data only collected for first 700 patients randomised

Section 1.3 – SSA Favourable Opinion removed from list of trial documentation required ahead of site accreditation

Section 2.1 – Amount of people diagnosed with prostate cancer annually updated

Section 2.4 –note added to explain completion of recruitment to celecoxib- containing arms

Section 2.5.2 - note added to explain completion of recruitment to celecoxib- containing arms

Section 3 – SSA Favourable Opinion removed

Section 4.2 – Exclusion criterion xiii greyed out

Section 4.3.1 – paragraph removed regarding potential randomisation to celecoxib-containing arms

Section 5 – Randomisation instructions expanded to exclude public holidays or dates when notice has been given by the CTU

Section 6.1.4 – formatting changed to grey font to reflect recruitment completion for arm D

Section 6.1.6 - formatting changed to grey font to reflect recruitment completion for arm F

Section 6.2.3 – recruitment note added

Section 6.6.3 – radiotherapy statement changed to reflect data from recent trials

Section 7.1.2 – removal of reference to SRE- specific CRF

Section 7.3 – Figure 3 - Addition of Bone Density Risk Factor Form and BMD sub-study assessment forms to summary of timing table

Section 7.3 – Figure 4 – Weeks added to timings of assessments post 2 years

Section 7.3- Figure 4 – note added to explain recruitment completion for arms D and F

Section 12.1 – Wording changed to reflect change to randomisation allocation ratio

Section 12.1 – Addition of statement regarding new information emerging during the trial

Section 12.2 – Reference to SSA removed

Section 16.3 – Statement added regarding actioning IDMC recommendation ahead of TSC ratification

21.1.8 Amendments Made To Protocol Version 7.0 (Jul--2011)

1. General Information Section- SAE reporting fax number corrected

2. Section 11- SAE reporting fax number corrected

21.1.9 Amendments Made To Protocol Version 7.1 (Jul-2011)

Throughout protocol – numbering has been updated in some sections new accommodate new information that has been added.

General Information Section – contact details updated

General Information Section – Funding information updated to include involvement from additional company

General Information Section – Wording on compliance and regulations updated to reflect current MRC CTU standard wording

General Information Section – Abbreviations list updated

Section 1.1 – The number of investigational agents being studied updated from three to four

Section 1.1 – Information regarding celecoxib updated to reflect that recruitment to these arms was discontinued in Apr-2011

Section 1.1 – Information about new IMP, Abiraterone inserted

Section 1.1 – Sample size and trial duration information updated to reflect changes brought about by additional trial arm

Section 1.2 – Summary information updated to reflect the discontinuation of recruitment to celecoxib arms and the addition of abiraterone

Figures 1a, b and c - Updated to reflect the discontinuation of recruitment to celecoxib arms and the addition of abiraterone

Section 1.2 – Information on trial stages updated to reflect changes brought about by additional trial arm

Section 1.2 – Information updated regarding the re-opening of the quality-of-life sub-study from implementation of protocol version 8.0

Section 2.1 – Wording related to hormone therapy updated for clarity

Section 2.1 - Updated to reflect the discontinuation of recruitment to celecoxib arms and the addition of abiraterone

Section 2.2 – Updated references added

Section 2.3 – Updated references added

Section 2.5 – Section added to give background information on new IMP, abiraterone

Section 2.6.1 – Updated references added

Section 2.7 – Section added to give information regarding radiotherapy which is to be given as part of standard care following recently published trial data.

Section 3 – Wording updated regarding selection of investigators to reflect current MRC CTU practice

Section 4.1 – Inclusion criteria updated with new criterion regarding radiotherapy use

Section 4.1 - Inclusion criteria updated with new criterion regarding contraceptive use

Section 4.1 – Wording of inclusion and exclusion criteria updated for clarity

Section 4.1 – Exclusion criteria updated with new criterion regarding acceptable liver function for trial entry

Section 4.1 – Exclusion criteria updated with specifics related to blood pressure levels

Section 4.1 - Exclusion criteria updated with new criterion regarding concomitant medications

Section 4.1 - Exclusion criteria updated with new criterion regarding prior treatment with abiraterone

Section 4.1 - Exclusion criteria updated with new criterion regarding prior treatment with chemotherapy

Section 4.1 - Exclusion criteria updated with new criterion regarding prior treatment with zoledronic acid

Section 4.3 – Wording updated to reflect that patients who initially fail screening can be re-screened at a later date

Section 4.3.2 – Wording updated regarding prior anti-androgen and LHRH use updated for clarity

Section 5.1 – Co-enrolment guidelines information updated to describe newly created co-enrolment CRF

Section 6.1 – Trial treatment information updated to reflect the fact that anti-androgens alone will be no longer permitted as hormone therapy

Section 6.1.1 – Updated to describe patients for whom radiotherapy should be given as standard practice

Section 6.1.1 a and b - Sections added to give information regarding radiotherapy treatment

Section 6.1.1-6.1.6 – References to further sections updated

Section 6.1.7 – Section added to describe abiraterone treatment

Section 6.2.4 - Section added to describe abiraterone treatment

Section 6.6 - Section added to give information regarding radiotherapy treatment

Section 7.1.1 – Reference to blood being taken at patient's home removed as this does not occur in practice

Section 7.1.2 – Wording updated regarding the reporting of biochemical failures for clarity

Section 7.1.2 – Wording updated regarding skeletal-related events for clarity

Section 7.1.3 – Section added to describe additional assessments required related to abiraterone treatment

Section 7.1.4 – Section added to provide information on when treatment should commence

Figure 4 – Updated for clarity regarding return of BMD sub-study forms, the addition the co-enrolment CRF and the description of the re-opening of the QL Sub-study.

Figure 5 – Updated with reference to abiraterone and co-enrolment form

Section 7.3 - Wording on trial closure updated to reflect current MRC CTU standard wording

Section 8.1 – Additional criteria for definition of progression added for clarity

Section 8.1 – Definition of progression for abiraterone patients added.

Section 9 – Statistical information updated to describe the addition of the new trial arm

Section 11 – Safety reporting wording updated for clarity

Section 11 – SAE reporting fax number updated

Section 12 – Ethical information updated to describe the unequal randomisation allocation ratio

Section 12 – Ethical information updated to describe that the visit schedule will vary according to trial arm

Section 12.2 – Wording updated to reflect international participation in the trial

Section 13 – Wording updated to reflect international participation in the trial

Section 14 – Wording updated to reflect international participation in the trial

Section 15 - Updated to reflect the discontinuation of recruitment to celecoxib arms and the addition of abiraterone

Section 16 – Reference to trial committee charters added for information

Section 17.1 – Information added to reflect re-opening of quality-of-life sub-study

Section 17.2 – Timing of health economics analysis updated to previous error

Section 18 – Information on publication policy expanded for clarity

Section 19 – Information regarding amendments to protocol appendices moved to the separate appendices document

Section 20 – References extensively updated

21.1.10 Amendments Made To Protocol Version 8.0 (Sep-2011)

Throughout protocol – numbering, sections headings, tables, figures and bibliographical references have been updated in some sections to accommodate new information that has been added

Throughout protocol – Androgen Deprivation Therapy has replaced Hormone Therapy as deemed more representative of the type of hormone therapy used in the study

General Information Section – New staff members of the MRC CTU and Co-Investigators added and contact details updated

General Information Section – Abbreviations list updated

Section 1.1 – Information regarding the new research radiotherapy treatment inserted

Section 1.1 – Information regarding docetaxel updated

Section 1.2 – Wording updated to reflect the addition of the new research comparison arm

Section 1.3 – Additional criteria for the re-accreditation of participating centres (for protocol version 9.0 only)

Section 2.1.1 – Wording updated to clarify the use of anti-androgen in trial patients

Section 2.1.2 – Information added to describe the rationale for the RT comparison arm

Section 2.8 – Information added to describe research RT treatment to prostate for patients with newly diagnosed metastatic disease

Section 3.1 – Information added to describe RT Quality Assurance procedures and centre accreditation

Section 4.1.1 to 4.1.3 – Wording updated to clarify inclusion criteria for all patients groups (newly diagnosed non-metastatic, metastatic and relapsing patients)

Section 4.2 – Clarification added on cardiovascular exclusion criteria

Section 4.2 – New exclusion criterion added concerning patients with prior exposure to hormone therapy

Section 4.2 – New exclusion criterion added to reflect the addition of the new RT comparison arm

Section 4.4.1 – Clarification added regarding pre-randomisation checks

Section 4.4.2 – Clarification added regarding permissible hormone therapy duration prior to randomisation

Section 4.4.5 – Information added regarding starting research radiotherapy treatment

Section 4.4.6 – Information updated on concomitant medications

Section 5 – Clarification regarding randomisation allocation added to reflect the addition of the new RT research arm

Section 6.1.8 – Information added to describe the administration of research radiotherapy

Section 6.2.1 – Clarification added regarding the measurement of serum creatinine levels prior to the administration of zoledronic acid

Section 6.2.3 – Clarification regarding the completion of recruitment to the celecoxib containing arms

Section 6.25 – Information added regarding the administration of research radiotherapy treatment

Section 6.6 – Clarification incorporated to describe the administration of standard-of-care radiotherapy

Section 7.1.4 – Information added regarding data collection and non-administration of standard radiotherapy

Section 7.2 – Section updated to include new treatment specific CRFs and timing of CRFs

Section 8.1 – Clarification added for the criteria to stop treatment for patients randomised to arm G

Section 8.2 – Section expanded to include additional details on study patient transfer to different centres

Section 8.3 – Additional sentence inserted to reinforce the importance of compliance with follow-up assessments

Section 9.1 – Additional paragraph inserted to clarify the method of randomisation and allocation distribution in the light of the introduction of the new RT arm

Section 9.4 – Wording updated to clarify the assessment of safety data

Section 9.5.4 – Wording updated concerning the end of randomisations to arm G

Section 9.6 to 9.6.4 – Section added describing sample size issues and trial stages for arm H

Section 9.8 – Clarification on intermediate stopping guidelines

Section 9.9 – Clarification on the outline analysis plan

Section 11 – Information on safety reporting updated to reflect the addition of the research RT comparison arm

Section 11 – Clarification added regarding arm A safety reporting timelines

Section 12.1 – Clarification added regarding the Principal Investigator’s responsibilities

Section 14 – Indemnity section updated to reflect current MRC policy

Section 16 – Clarification regarding TMG membership

Section 17.3 – Section on Bone Mineral Density sub-study removed

Section 19 – Information regarding amendments to protocol appendices moved to the separate appendices document

Section 20 – References updated

21.1.11 Amendments Made To Protocol Version 9.0 (Oct-2012)

Throughout protocol – numbering, sections headings, tables, figures and bibliographical references have been updated in some sections to accommodate the completion of recruitment to original research arms B, C and E.

Throughout protocol – Tenses have been changed to reflect activities that were in the future and which have now been passed.

Section 1 – Figure added and clarifications added to each figure

Section 2 – Previous reference 8 removed

Section 4 – Clarification of acceptable alternatives to bone scans

Section 6.2.5 – Correction of an error defining the PTV: the wording has been reordered

Table 4 – Dose-volume objectives corrected: order swapped

Table 5- Correction CRFs names

Section 17.3.2 – Clarification that DNA may be extracted

21.1.12 Amendments Made To Protocol Version 10.0 (Apr-2013)

Throughout protocol – numbering, sections headings, tables, figures and bibliographical references have been updated in some sections

Throughout protocol – typos have been corrected

Section 4 –Clarification of exclusion criteria V (now V and VI)

Section 6 – Timing of orchidectomy prior to randomisation extended to 12 weeks

Section 6 – Clarification of hypokalaemia, blood pressure and fluid retention management

Section 9 – Statistical considerations amended in light of the recruitment extension for the abiraterone comparison

Section 14 - Section updated to reflect the changes in the structure of the MRC CTU (now MRC CTU at UCL) and indemnity arrangements

21.1.13 Amendments Made To Protocol Version 11.0 (Sep-2013)

Throughout protocol – numbering, sections headings, tables, figures and bibliographical references have been updated in some sections

Throughout protocol – typos have been corrected

Co-investigators list updated to reflect the addition of the “enzalutamide + abiraterone comparison” lead

Section 1.2 – Enzalutamide added as trial treatment

Section 1.2 – Protocol version 12.0 added to the list of amendments

Section 2.10 – Rationale for the combination of enzalutamide and abiraterone

Section 4.2 – Eligibility criteria amended to reflect the addition of enzalutamide + abiraterone arm

Section 4.4.2 – Wording clarified

Section 6.8 – Clarification regarding end of trial treatment after starting trial therapy

Section 6.10 – Section added to describe enzalutamide and abiraterone treatment for the new research arm (Arm J)

Section 6.11.4.A – Section added to describe the management of toxicities from trial abiraterone

Section 6.11.4.B - Section added to describe the management of toxicities from trial enzalutamide

Section 9.1.4 – Section added to describe the statistical considerations concerning the introduction of Arm J

Section 9.3 – Principles and assumption for the introduction of Arm J added

Section 9.7 and sub-sections – Sample size issues and trial stages for Arm J

Section 9.9 – Details on interim monitoring and analyses for Arm J added

Section 11.2.1.D – Wording clarified regarding safety reporting requirements for control arm

Section 12.1 – Wording clarified

Section 15 – Details on funding for the “enzalutamide + abiraterone comparison” added

Section 19 - Amendments made to protocol updated

Reference list updated

21.1.14 Amendments Made To Protocol Version 12.0 (Jan-2014)

Throughout protocol – typos have been corrected

Section 4.4.2. Wording clarified

Section 4.3. Wording clarified for eligibility to M1|RT comparison

Section 6.10. Addition of use of dexamethasone post-biochemical progression for Arm J patients

Section 6.11.4.A. Correction of CTCAE version

Section 6.11.4.C. Clarification on enzalutamide dose modification to be in line with current SmPC

Section 9.6. Sample size increase for M1|RT comparison

Section 11. Correction of safety reporting timelines for Arm A patients

Section 17. Addition of saliva samples collection for DNA analysis

Table 4, 5 and 6. Clarification on Case Report Forms and Follow-up schedule

21.1.15 Amendments Made To Protocol Version 13.0 (Feb-2015)

Throughout protocol – typos have been corrected

Throughout protocol – clarification on the new definition of standard-of-care

Table of contents updated to reflect any changes to the protocol

Section 1.1. Wording added throughout section to include reference to survival results from “original comparisons”

Section 2.1.1. Section improved to include reference to survival results from “original research comparisons”

Section 2.1.2. Section improved to include reference to survival results from “original research comparisons”.

Section 2.1.3. Additional section added to describe the role of docetaxel for people with M0 or M1 disease

Section 2.9. Clarification on treatment completion and primary results for “original research comparisons”

Section 4.2. Clarification of Exclusion criteria XIII and XVI

Section 4.4.2. Clarification on HT prior to randomisation

Section4.4.3. New section to clarify standard-of-care docetaxel treatment prior to randomisation

Section 4.4.7. Clarification on concomitant medication and contra-indicated concomitant medications

Section 4.5. Clarification provided on tissue block collection

Section 6. Inclusion of docetaxel into the standard-of-care

Section 6.2.3 New section to describe standard-of-care docetaxel administration

Section 6.11. Improvement throughout sections and sub-sections for abiraterone and enzalutamide-related toxicity management

Section 6.12. Section improved throughout to incorporate clearer details on concomitant medications and drug-to-drug interactions

Section 7.1.4. New section to describe data collection for standard-of-care docetaxel

Section 9.7.4. Clarification provided about implications for “enzalutamide+ abiraterone comparison” following change of standard-of-care treatment

Section 11.2.1.D Clarification on SAE notification timelines to reflect change in standard-of-care treatments (addition of docetaxel)

Figure 1. Figure updated to reflect change in standard-of-care

Figure 2. Figure updated to reflect trial history and recruitment over time

Figure 3. Figure updated to reflect changes in standard-of-care and recruiting arms

Table 1. Table updated to remove repetition

Table 13. Table updated to include new CRF to report standard-of-care docetaxel treatment

Table 15. Table updated to include only active trial treatments

21.1.16 Amendments Made To Protocol Version 14.0 (Oct-2015)

Throughout protocol – typos have been corrected

Throughout protocol – clarification on the new definition of standard-of-care

Table of contents updated to reflect any changes to the protocol

Section 1. Wording added throughout section to include reference “metformin comparison”

Section 2. Section updated to include reference “metformin comparison”

Section 4.2. Exclusion criteria review to reflect Arm J closure and instruction of “metformin comparison”

Section 4.3. Clarification of comparison specific eligibility (M1|RT and metformin)

Section 4.5.7. Clarification on concomitant medication and contra-indicated concomitant medications

Section 6. Treatment sections improved throughout

Section 6.11. Section updated to include details on metformin treatment

Section 6.12. Amendment throughout sections and sub-sections for metformin treatment

Section 6.13. Amendment throughout sections and sub-sections for metformin treatment

Section 6.13. Improvement throughout sections and sub-sections for abiraterone and enzalutamide treatment

Section 7.0. Amendment throughout sections and sub-sections to include assessment and procedures specific to “metformin comparison”

Section 9.0. Section updated and streamlined to capture statistical considerations on each comparison

Section 9.0. Details on “metformin comparison” added

Section 11. Safety processes updated and clarified

Section 16.0 Membership to oversight groups updated

Section 11.2.1.D Clarification on SAE notification timelines to reflect change in standard-of-care treatments (addition of docetaxel)

21.1.17 Amendments Made To Protocol Version 15.0 (Mar-2017)

Throughout protocol – re-structure of the treatment-related information for ease of use

Throughout protocol – clarification on the definition of standard-of-care

Throughout protocol – typos have been corrected

Addition of TMG members

Table of contents updated to reflect any changes to the protocol

New section for summary of trial added in table format

Section 1. Revised format for the summary of treatment groups, with the new transdermal oestradiol arm also added

Section 2. Clarification regarding research treatments that have previously reported or completed recruitment, section updated to include the "transdermal oestradiol comparison"

Section 3. New sections added for the "transdermal oestradiol comparison" and future planned biomarker-selected comparisons

Section 4.1.4. Change in definition of adequate renal function

Section 4.3. New section added for the biomarker screening pilot, selection criteria removed for "research RT comparison"

Section 4.4.1. Change in definition of adequate renal function

Section 4.4.2. New section added for the patient selection criteria specific to the "transdermal oestradiol comparison"

Section 4.5. Screening procedure tables and figure added for clarification.

Section 4.5.1. New section added for biomarker screening pilot investigations prior to randomisation.

Section 5.1.1. New section added for the biomarker screening pilot registration.

Section 6. New sections added for the "transdermal oestradiol comparison"

Section 7. Amendment throughout sections and sub-sections to include assessment and procedures specific to "transdermal oestradiol comparison"

Section 7.1.4.B. Section added on cardiovascular outcomes for the "transdermal oestradiol comparison"

Table 18. Table added to clarify follow-up assessments

Section 8. Section updated for "transdermal oestradiol comparison"

Section 9. Section updated for "transdermal oestradiol comparison"

Section 12.1.1.D. Section added on participant feedback from investigations and additional analyses

Section 15. Section updated for "transdermal oestradiol comparison" and biomarker screening pilot

21.1.18 Amendments Made To Protocol Version 16.0 (Oct-2017)

Summary of trial- Table 1: Schedule of Assessments has been added

Abbreviations & Glossary- new terms have been added

Section 1- Table 4: Abiraterone information updated as results of primary analysis published

Section 4.3 - Biomarker timelines redefined, the length of prior hormone therapy has increased to reflect change in turnaround time for testing

Section 4.6 - Biomarker screening information updated

Section 6.2 – Clarification on safety monitoring required for patients receiving trial abiraterone added . Abiraterone overdose information altered for clarity.

Section 6.3.4 - Drug interactions updated to specify that tamoxifen is contraindicated in combination with abiraterone, enzalutamide and transdermal oestradiol.

Section 6.5 – Detail on requirements at site to demonstrate compliance with per-protocol required safety monitoring added

Section 7 – Schedule for assessments updated, removal of table 19

Section 7.1 - Clarification on additional safety monitoring required for patients receiving trial abiraterone added

Section 7.4 - Table 20 QL information removed and added to Table 1: Schedule for Assessments

Section 10.1.1- Central monitoring of consent information added

Section 11 – Re-structured and re-worded for clarity on reporting requirements for safety data captured on the SAE CRF. Explanation provided for exempted events and definitions added. Table 28 and Box 1 updated and Figure 1 added.

Section 11.2- Updated SAE exceptions, SAE flow chart added for clarity

Section 11.3 - Update of investigator assessments and notification checklist for expedited safety reporting

Section 11.4 - Update of wording of CTU responsibilities

Section 17.4 - Sub-study information added to include Disease Volumetric sub-study

21.1.19 Amendments Made To Protocol Version 17.0 (Feb-2017)

Throughout protocol - Typos have been corrected, abbreviations & glossary & table of contents updated

Throughout protocol - Addition of abiraterone as SOC & original comparisons closed to active follow-up

Throughout protocol – Update of and removal of biomarker pilot information now randomisation to the rucaparib comparison is to be activated

Trial administration – Information updated, full contact list linked to website, all comparison chief-investigators added as co-signatories

Summary of trial – Updated, “rucaparib comparison” added and “original comparisons” closed to active follow-up; figure 1 updated with new randomisation schema and S-STAMPEDE Cohort study

Schedule of Assessments updated– Table 1a removed, Tables 1, 2 and 3 added

Lay Summary – Re-drafted, “rucaparib comparison” added

Section 2 – Role of SOC abiraterone added, reported comparisons updated and rationale for comparisons that have completed recruitment removed. Rational for the “rucaparib comparison” added

Section 3.1 – Addition of site and investigator criteria

Section 4 – Complete restructuring of section, addition of biomarker screening and registration information.

Section 4.2 – Additional information about proposed approach to staged informed consent

Section 4.4 – Clarification as to required pre-randomisation screening by comparison

Section 4.5.4 – Detail regarding SOC abiraterone (permitted in metformin comparison only)

Section 4.9.3 – Eligibility to be randomised to the “rucaparib comparison” added

Section 4.10 - Sub-study eligibility criteria clarified; new germline blood sub-study added (PAXgene for S1A and S1M) and stratified – STAMPEDE cohort study added

Section 5 – additional information relating to registration and randomisation to the “rucaparib comparison” added

Section 6.1.4- SOC abiraterone detail added

Section 6.2.7.C – Table 19: Additional assessments required following change of transdermal oestradiol patch or dose added

Section 6.2.9 - Rucaparib treatment specific information added

6.3 – Concomitant medications updated: clarification that spironalactone is contraindicated with abiraterone and rucaparib drug interactions added

Section 7.1 – Table 27: summary of follow-up schedules by participant group added

Section 7.1.5.D – Additional Safety assessment required for participants receiving rucaparib added

Section 7.2.3 – Data collection for SOC abiraterone clarified

Section 7.2.8 & 7.3.2 - Data collection & Follow-up procedures for S-STAMPEDE Cohort participants described

Section 7.3.3 – Clarification added regarding procedures to use linked follow-up information obtained from sources of electronic health data

Table 29 and 28 updated with new CRFs

Section 8.1.4 – Reasons to stop rucaparib

Section 9.8 – addition of statisitcal considerations relating to the “rucaparib comparison”

Section 11 – Stopping of SARs and SUSARs reporting for “original comparisons” closed to active follow up and addition of rucaparib-specific notable events

Section 12- ethical considerations updated with detail relating to data to permit linkage with sources of electronic health data

Section 13 – Data archiving and retention guidance added

Section 16 – Updates of STAMPEDE oversight committees including expanded TMG sub-groups

Section 17.1.1 A- Closure of HE & QL sub-studies to new participants and stopping of data collection for several comparisons; summarised in Table 40.

Section 17.2 – S-STAMPEDE cohort study & additional germline data collection added

Section 17.4 – New sub-study: Using routine data to identify clinical trial outcomes added

Section 18 – New section regarding patient and public involvement in STAMPEDE added

Section 20 - New section regarding data and sample sharing added

21.1.20 Amendments made to Protocol Version 18.0 (Jun-2018)

Throughout protocol: Removal of "rucaparib comparison" information

Throughout protocol: Redraft of biomarker screening pilot study into ancillary studies section

Summary of Trial – Figure 1 updated; removal of registration information for S-STAMPEDE Cohort study

Schedule of Assessments – Removal of registration from figure 1; Table 2 updated to include PSA within 8 weeks of randomisation; Table 3 S-STAMPEDE Schedule of Assessments removed

Abbreviations & Glossary - Terms relating to the "rucaparib comparison" have been deleted

Section 1 – Lay summary "rucaparib comparison" information removed

Section 2 – Rationale for incorporating molecular stratificaion and "rucaparib comparison" removed

Section 3.2 – "Rucaparib comparison" comparison-specific site accreditation removed

Section 4 – Removal of biomarker screening and registration information

Section 4.2 – Removal of staged informed consent process

Section 4.3 – Removal of biomarker screening eligibility information

Section 4.4.3 – Removal of "rucaparib comparison" screening investigations prior to randomisation

Section 4.5 – Removal of prior permitted SOC treatments for "rucaparib comparison"

Section 4.7 – Removal of and clarification to the general inclusion & exclusion criteria of Serum Pottasium & Cardiovascular disease respectively.

Section 4.9.3 – Removal of "rucaparib comparison" specific eligibility criteria

Section 4.10 – Addition of information regarding biomarker pilot screening

Section 4.10.1 – Removal of S-STAMPEDE Cohort sub-study

Section 4.10.2 – Removal of PAXgene sample collection

Section 5 – Removal of information relating to registration and randomisation to the "rucaparib comparison"

Section 6.2.6.B – Addition of additional metformin dose reduction stages

Section 6.2.9 – Removal of "rucaparib comparison" research treatment information

Section 6.3.1 – Removal of "rucaparib comparison" therapeutic interactions information

Section 7 - Table 30 removal of "rucaparib comparison" specific CRFs; table 31 – removal of arm S1M schedule for completion of treatment forms

Section 7.1.5.D – Removal of rucaparib additional safety assessments

Section 7.2.8 – Removal of data collection for S-STAMPEDE Cohort participants

Section 7.3.2 – Removal of follow-up for S-STAMPEDE cohort participants

Section 8.1.1 – Clarification of metformin, abiraterone and enzalutamide use post progression

Section 8.1.4 – Removal of "rucaparib comparison" stopping trial treatment information

Section 9.6.7 – Revised sample size for "metformin comparison"

Section 9.8 – Removal of statistical consideration relating to the "rucaparib comparisons"

Section 10.1.1 – Updated central monitoring of consent process

Section 11 – Removal of rucaparib-sepcific notable events

Section 11.1.1 – Clarification of trial-specific exemptions and notable adverse events

Section 16 – Addition of Genetic Sub-Group

Section 17.1.1.A – Clarification of Qulaity of Life and Health Economics data collection

Section 17.2 – Details of biomarker screening pilot study information moved here

Section 17.2.1 – removal of S-STAMPEDE Cohort study information

21.1.21 Amendments made to Protocol Version 19.0 (Aug-2020)

Throughout protocol: Section headings and table numbers renumbered, references updated, Centre changed to Site for consistency

General information - Sponsor updated to UCL, Trial contacts updated, Glossary updated

Summary of Trial – M1RT status updated, transdermal oestradiol number of participants updated

Figure 1 updated to include new SOC options and clarify options based on HbA1c level.

Figure 2 updated to reflect extended recruitment in metformin and te2 comparisons

Schedule of Assessments –remove height and upadte footnotes

Abbreviations – minor updates

Section 1 – updated comparisons closed to recruitment – addition of Arm H. Arms E and F added to Table 6.

Section 2.1.3 – Addition of new SOC options abiraterone, enzalutamide and apalutamide

Section 2.3 – Addition of M1RT results

Section 2.5.2.B – Updated target recruitment for “transdermal oestradiol” comparison

Section 3.1.2 – Addition to recommend additional investigators be delegated for safety reporting to cover absences

Section 4.2 – Minor rewording clarification

Section 4.1 – Update to screening investigations, addition of timeframes in days, M1 imaging clarified, several baseline investigations moved to pre-randomisation. ECG removed, option for no fasting glucose added.

Section 4.3 – Table 8 - Addition of new SOC options abiraterone, enzalutamide and apalutamide

Section 4.3.2 – Added oligometastatic disease

Section 4.3.3 –Clarification SOC docetaxel cannot be given if SOC abiraterone, enzalutamide and apalutamide is planned

Section 4.3.4 – New section for SOC abiraterone, enzalutamide and apalutamide. Addition of enhanced monitoring for “transdermal oestradiol” comparison

Section 4.4.4 – General inclusion criteria III removed, haematological value thresholds clarified

Section 4.5 – General exclusion criteria II added (consolidates previous VII-IX), clarification where both AST and ALT results required, VI clarified exclusion is for unhealed surgical wounds rather than surgerical intervention

Section 4.6.1 – Added metabolic substudy to “metformin“ comparison requirements

Section 4.6.2 – Added eligibility criteria for participants not yet started on SOC abiraterone, enzalutamide and apalutamide

Section 4.7.2 – Clarification circulating tumour DNA sub study not recruiting

Section 4.7.4 – New section for Eligibility for metformin sub study

Section 5.1 – Addition of new instruction to provide randomisation documents to CTU after randomisation.

Section 5.2 – Clarification of wording for co-enrolment in other trials (interventional and non-interventional)

Section 6 – Reformatted throughout, new subheadings and layout, multiple new tables inserted

Section 6.1 –Addition of SOC combination table to replace text list

Section 6.1.1 – Section renamed Androgen Deprivation Therapy (previously Hormone Therapy)

Section 6.1.2 – Updated wording on Radiotherapy for M0 participants

Section 6.1.2.C – New section for oligometastatic participants

Section 6.1.3 – Administration of SOC RT moved up from 6.7

Section 6.1.5 – New title SOC upfront systemic therapy – addition of SOC abiraterone, enzalutamide and apalutamide

Section 6.2 –Research treatment broken down per IMP instead of per comparison.

Section 6.2.1 – Addition of Table 9 treatment duration for all research treatments and Table 10 management of trial treatment post progression

Section 6.2.2 – Some text now in tables. Updated wording about associated toxicities and contraindications

Section 6.2.3 – Some text now in tables. Updated wording about associated toxicities and contraindications

Section 6.2.3.C – New table for hypertension monitoring.

Section 6.2.4 - Some text now in tables. Updated wording about associated toxicities and contraindications

Section 6.2.5 - Reformatted, some text now in tables. Updated wording about associated toxicities and contraindications. Additional detail on moving from induction to maintenance dose on oestradiol level, and changing brands of patches

Section 6.3 – Clarification of wording. Details of drug interactions and additional safety monitoring moved up to sections 6.2.2, 6.2.3, 6.2.4 and 6.2.5 respectively

Section 7 – Added Figure 3 - PSA progression example scenarios

Section 7.1.4 – Clarification added to explain rationale for continuing to collect metabolic test results post progression

Section 7.1.3.A –Addition of Figure 3 example progression schenarios

Section 7.1.4 – Addition of rationale for continuing metabolic tests beyond progression

Section 7.1.5.A Clarification of required tests for abiraterone and enzalutamide, specifically when one or the other is discontinued.

Section 7.1.5.C - Addition of real time monitoring of hormone results for te2 participants to check safety of combination with new SOC abiraterone, enzalutamide and apalutamide

Section 7.2.3 – Addition of SOC systemic therapy log

Section 7.2.4 – Addition of requirement to submit radiotherapy CRF even if radiotherapy not given

Section 7.3 – Nurse led follow up expanded to allow for other appropriately qualified individuals

Section 7.3.1 – Minor changes

Section 7.4.1 – Table 28 updated with new CRFs for SOC systemic therapy, and metabolic sub study CRF. Removal of baseline form, bone density risk factor. Blood form moved. Updated key for Table 29.

Section 8.1 – Clarification of consent for data collection

Section 8.1.3 – Additional wording on stopping te2 and options for pausing, switching and restarting.

Section 8.2 – New section about permitted breaks in SOC ADT treatment

Section 9.2 – Additional wording regarding analysis of “enzalutamide + abiraterone comparison”

Section 9.5 – Additional wording regarding analysis of “enzalutamide + abiraterone comparison”

Section 9.7.4 – New section detailing enhanced safety monitoring of combination new SOC and transdermal oestradiol

Section 9.10 – removal of Mann-Whitney test as relevant for original comparisons now closed.

Section 9.10.2 - Additional wording regarding analysis of “enzalutamide + abiraterone comparison”

Section 10.1 – Minor clarification to wording

Section 10.1.2 – Clarification of central monitoring processes

Section 10.1.3 – Wording updated in line with current protocol template

Section 11.1.1 – Clarification of notable events (NEs) to be collected, new cancers no longer included.

Section 11.1.2 – Addition of new SOC enzalutamide and apalutamide

Section 11.2 – Update to add requirement for investigator absence cover, updated AE, SAE and NE notification period. Figure 5 added.

Section 11.2.2 – Updated expedited reporting exemptions

Section 11.2.3.C – Expectedness removed from site investigator responsibilities. Table 37 updated

Section 11.2.3.D – Figure 6 updated. Box 1 updated

Section 11.3 – Updated process for causality queries. Clarification of Swiss reporting responsibilities

Section 11.3.1 – Expectedness added to sponsor responsibilities

Section 12.1.1.A – Updated with new SOC enzalutamide and apalutamide

Section 13 – CTA number updated

Section 15 - Clarification for SOC enza and addition of SOC apalutamide

Section 16.2 - Clarification on frequency of TSC meetings

Section 16.4 – subgroups updated

Section 16.5 – Figure 7 updated

Section 17.1 – Addition that secure email now permitted for sending CRFs

Section 17.2.2 – Minor changes, sub study is now closed

Section 17.2.4 – Removed details of biomarker sub study as now closed

Section 17.5 – New section added for metformin metabolic sub study

Section 20 – Clarification that any data released must be approved by TMG, TSC and subject to agreements

Section 22 – References updated, removal of several references linked to protocol v18 which was not released to sites

21.1.22 Amendments made to Protocol Version 20.0 (Oct-2020)

Minor amendment to correct typograhical, spelling, formatting and cross-reference error, or clarify wording throughout.

Minor updates to Summary of Trial table

Table 2 – ECG removed from Cardiac asseessment row, weight added to waist measurement row

4.2.2 – Timeframe for pre - SOC docetaxel bloods updated from 4 months to 16 weeks in line with other timeframes.

4.4 Header title changed

4.6 Footnote 1 restored

5.2 Wording revised to allow participants to continue on STAMPEDE research treatment while co-enrolled in IMP trial, providing no interactions.

6.1.2 Header title changed

6.1.3.A Header title changed

6.2 Footnote 2 removed from Table 10

6.2.3.C – Restoration of Table 11, deleted in error when accepting tracked changes. Table 11 outlines 3 monthly blood pressure monitoring as specified in section 7.1.5.B

6.3 Header title changed

11.2.2 – Correction to bullet list exemption, text removed

22 REFERENCES

1. James ND, Sydes MR, Clarke NW, Mason MD, Dearnaley DP, Spears MR, et al. Addition of docetaxel, zoledronic acid, or both to first-line long-term hormone therapy in prostate cancer (STAMPEDE): survival results from an adaptive, multiarm, multistage, platform randomised controlled trial. Lancet. 2016;387(10024):1163-77.
2. Mason MD, Clarke NW, James ND, Dearnaley DP, Spears MR, W.S.R. A, et al. Adding celecoxib with or without zoledronic acid for hormone-naive prostate cancer: long-term survival results from an adaptive, multi-arm, multi-stage, platform, randomised controlled trial. 2017. In press. DOI: 10.1200/JCO.2016.69.0677.
3. James ND, de Bono JS, Spears MR, Clarke NW, Mason MD, Dearnaley DP, et al. Abiraterone for Prostate Cancer Not Previously Treated with Hormone Therapy. N Engl J Med. 2017;377(4):338-51.
4. Parker CC, James ND, Brawley CD, Clarke NW, Hoyle AP, Ali A, et al. Radiotherapy to the primary tumour for newly diagnosed, metastatic prostate cancer (STAMPEDE): a randomised controlled phase 3 trial. Lancet. 2018;392(10162):2353-66.
5. cancerresearchuk. Prostate cancer statistics
2015 [Available from: http://www.cancerresearchuk.org/health-professional/cancer-statistics/statistics-by-cancer-type/prostate-cancer.
6. Sharifi N, Gulley JL, Dahut WL, Sharifi N, Gulley JL, Dahut WL. An update on androgen deprivation therapy for prostate cancer. Endocrine-Related Cancer. 2010;17(4):R305-15.
7. Widmark A, Klepp O, Solberg A, Damber JE, Angelsen A, Fransson P, et al. Endocrine treatment, with or without radiotherapy, in locally advanced prostate cancer (SPCG-7/SFUO-3): an open randomised phase III trial. Lancet. 2009;373(9660):301-8.
8. Warde PR, Mason MD, Sydes MR, Gospodarowicz MK, Swanson GP, Kirkbride P, et al. Intergroup randomized phase III study of androgen deprivation therapy (ADT) plus radiation therapy (RT) in locally advanced prostate cancer (CaP) (NCIC-CTG, SWOG, MRC-UK, INT: T94-0110; NCT00002633). J Clin Oncol. 2010;28(18s Supplement: Proceedings of ASCO 2010):Abstr CRA4504.
9. Warde P, Mason M, Ding K, Kirkbride P, Brundage M, Cowan R, et al. Survival Benefit with Combined Androgen Deprivation and Radiation Therapy in Locally Advanced Prostate Cancer – Results of a Phase III Trial. The Lancet. 2011 - in press.
10. Mason M, Sydes M, Parulekar W, Parmar M, Anderson J, Barber J, et al. Final analysis of intergroup randomized phase III study of androgen deprivation therapy (ADT) + radiation therapy (RT) in locally advanced prostate cancer (CaP) (NCIC-CTG, SWOG, MRC-UK, INT: T94-0110). National Cancer Research Institute (NCRI) Cancer Conference 2012. 2012;2012.
11. Warde P, Mason M, Ding K, Kirkbride P, Brundage M, Cowan R, et al. Combined androgen deprivation therapy and radiation therapy for locally advanced prostate cancer: a randomised, phase 3 trial. The Lancet. 2011;378:2104-11.
12. Nicholas D James MRS, Noel W Clarke, David P Dearnaley, Malcolm D Mason, Christopher C Parker, Alastair W S Ritchie, J. Martin Russell, Francesca Schiavone, Gerhardt Attard, Johann S de Bono, Alison Birtle,Daniel S Engeler,Tony Elliott, David Matheson, Joe O'Sullivan, Delia Pudney, Narayanan Srihari, Jan Wallace, Jim Barber, Isabel Syndikus, Mahesh K B Parmar, Matthew R Sydes. Failure-free survival and the impact of radiotherapy in patients with newly diagnosed non metastatic prostate cancer: Data from patients in the control arm of the STAMPEDE trial (MRC PR08, CRUK/06/019). JAMA Oncology. 2015.
13. Sweeney CJ, Chen YH, Carducci M, Liu G, Jarrard DF, Eisenberger M, et al. Chemohormonal Therapy in Metastatic Hormone-Sensitive Prostate Cancer. N Engl J Med. 2015;373(8):737-46.
14. Vale CL, Burdett S, Rydzewska LH, Albiges L, Clarke NW, Fisher D, et al. Addition of docetaxel or bisphosphonates to standard of care in men with localised or metastatic, hormone-sensitive

prostate cancer: a systematic review and meta-analyses of aggregate data. Lancet Oncol. 2016;17(2):243-56.
15. James ND, Ingleby FC, Clarke NW, Amos C, Attard G, Cross W, et al. Docetaxel for hormone-naive prostate cancer (PCa): Results from long-term follow-up of non-metastatic (M0) patients in the STAMPEDE randomised trial. Annals of Oncology [Internet]. 2019 Oct; 30. Available from: https://doi.org/10.1093/annonc/mdz248.008a.
16. Fizazi K, Tran N, Fein L, Matsubara N, Rodriguez-Antolin A, Alekseev BY, et al. Abiraterone plus Prednisone in Metastatic, Castration-Sensitive Prostate Cancer. N Engl J Med. 2017;377(4):352-60.
17. Hoyle AP, Ali A, James ND, Cook A, Parker CC, de Bono JS, et al. Abiraterone in "High-" and "Low-risk" Metastatic Hormone-sensitive Prostate Cancer. Eur Urol. 2019;76(6):719-28.
18. Davis ID, Martin AJ, Stockler MR, Begbie S, Chi KN, Chowdhury S, et al. Enzalutamide with Standard First-Line Therapy in Metastatic Prostate Cancer. N Engl J Med. 2019;381(2):121-31.
19. Chi KN, Agarwal N, Bjartell A, Chung BH, Pereira de Santana Gomes AJ, Given R, et al. Apalutamide for Metastatic, Castration-Sensitive Prostate Cancer. N Engl J Med. 2019;381(1):13-24.
20. James ND, Sydes MR, Clarke NW, Mason MD, Dearnaley DP, Spears MR, et al. Addition of docetaxel, zoledronic acid, or both to first-line long-term hormone therapy in prostate cancer (STAMPEDE): survival results from an adaptive, multiarm, multistage, platform randomised controlled trial. Lancet. 2015.
21. Smith MR, Finkelstein JS, McGovern FJ, Zietman AL, Fallon MA, Schoenfeld DA, et al. Changes in body composition during androgen deprivation therapy for prostate cancer. J Clin Endocrinol Metab. 2002;87(2):599-603.
22. Eriksson A, Attvall S, Bonnier M, Eriksson JW, Rosander B, Karlsson FA. Short-term effects of metformin in type 2 diabetes. Diabetes Obes Metab. 2007;9(4):483-9.
23. Bailey CJ. Treating insulin resistance in type 2 diabetes with metformin and thiazolidinediones. Diabetes Obes Metab. 2005;7(6):675-91.
24. Ohira M, Miyashita Y, Ebisuno M, Saiki A, Endo K, Koide N, et al. Effect of metformin on serum lipoprotein lipase mass levels and LDL particle size in type 2 diabetes mellitus patients. Diabetes Res Clin Pract. 2007;78(1):34-41.
25. Wulffele MG, Kooy A, de Zeeuw D, Stehouwer CD, Gansevoort RT. The effect of metformin on blood pressure, plasma cholesterol and triglycerides in type 2 diabetes mellitus: a systematic review. J Intern Med. 2004;256(1):1-14.
26. Lubik AA, Gunter JH, Hendy SC, Locke JA, Adomat HH, Thompson V, et al. Insulin increases de novo steroidogenesis in prostate cancer cells. Cancer Res. 2011;71(17):5754-64.
27. Venkateswaran V, Haddad AQ, Fleshner NE, Fan R, Sugar LM, Nam R, et al. Association of diet-induced hyperinsulinemia with accelerated growth of prostate cancer (LNCaP) xenografts. J Natl Cancer Inst. 2007;99(23):1793-800.
28. Noto H, Goto A, Tsujimoto T, Noda M. Cancer risk in diabetic patients treated with metformin: a systematic review and meta-analysis. PLoS One. 2012;7(3):e33411.
29. Rothermundt C, Hayoz S, Templeton AJ, Winterhalder R, Strebel RT, Bartschi D, et al. Metformin in chemotherapy-naive castration-resistant prostate cancer: a multicenter phase 2 trial (SAKK 08/09). Eur Urol. 2014;66(3):468-74.
30. Krupski TL, Smith MR, Lee WC, Pashos CL, Brandman J, Wang Q, et al. Natural history of bone complications in men with prostate carcinoma initiating androgen deprivation therapy. Cancer. 2004;101(3):541-9.
31. Langley RE, Cafferty FH, Alhasso AA, Rosen SD, Sundaram SK, Freeman SC, et al. Cardiovascular outcomes in patients with locally advanced and metastatic prostate cancer treated with luteinising-hormone-releasing-hormone agonists or transdermal oestrogen: the randomised, phase 2 MRC PATCH trial (PR09). Lancet Oncol. 2013;14(4):306-16.
32. Byar DP. Proceedings: The Veterans Administration Cooperative Urological Research Group's studies of cancer of the prostate. Cancer. 1973;32(5):1126-30.

33. Hedlund PO, Henriksson P. Parenteral estrogen versus total androgen ablation in the treatment of advanced prostate carcinoma: effects on overall survival and cardiovascular mortality. The Scandinavian Prostatic Cancer Group (SPCG)-5 Trial Study. Urology. 2000;55(3):328-33.
34. Gilbert DC, Duong T, Kynaston HG, Alhasso AA, Cafferty FH, Rosen SD, et al. Quality-of-life outcomes from the Prostate Adenocarcinoma: TransCutaneous Hormones (PATCH) trial evaluating luteinising hormone-releasing hormone agonists versus transdermal oestradiol for androgen suppression in advanced prostate cancer. BJU Int. 2016.
35. Smith DC, Redman BG, Flaherty LE, Li L, Strawderman M, Pienta KJ. A phase II trial of oral diethylstilbesterol as a second-line hormonal agent in advanced prostate cancer. Urology. 1998;52(2):257-60.
36. Hsueh AJ, Peck EJ, Jr., clark JH. Progesterone antagonism of the oestrogen receptor and oestrogen-induced uterine growth. Nature. 1975;254(5498):337-9.
37. Hsueh AJW, Peck Jr EJ, Clark JH. Progesterone antagonism of the oestrogen receptor and oestrogen-induced uterine growth. Nature. 1975;254:337.
38. Janssen. L. ZYTIGA® (abiraterone acetate) Investigator Brochure. 13 ed2017.
39. BNF. Abiraterone interactions [Available from: https://www.medicinescomplete.com/#/content/bnf/_293081460_interactions.
40. Woo HH, Begbie S, Gogna K, Mainwaring PN, Murphy DG, Parnis F, et al. Multidisciplinary consensus: a practical guide for the integration of abiraterone into clinical practice. Asia Pac J Clin Oncol. 2014;10(3):228-36.
41. Zentiva. Tamsulosin 400 microgram prolonged-release hard capsules, Summary of Product Characteristics [Available from: https://www.medicines.org.uk/emc/product/9245/smpc.
42. Tucci M, Roca E, Ferrari L, Pia A, Dalla Volta A, Bedussi F, et al. Abiraterone and prednisone therapy may cause severe hypoglycemia when administered to prostate cancer patients with type 2 diabetes receiving glucose-lowering agents. Endocrine. 2019;64(3):724-6.
43. Astellas. Xtandi®(enzalutamide, MDV3100) Investigator Brochure. 11 ed2019.
44. Lipska KJ, Bailey CJ, Inzucchi SE. Use of metformin in the setting of mild-to-moderate renal insufficiency. Diabetes Care. 2011;34(6):1431-7.
45. Merck. Glucophage 500 mg film coated tablets Summary of Product Characteristics [Available from: https://www.medicines.org.uk/emc/product/987/smpc.
46. Salpeter SR, Greyber E, Pasternak GA, Salpeter EE. Risk of fatal and nonfatal lactic acidosis with metformin use in type 2 diabetes mellitus: systematic review and meta-analysis. Arch Intern Med. 2003;163(21):2594-602.
47. Viani GA, Bernardes da Silva LG, Stefano EJ. Prevention of gynecomastia and breast pain caused by androgen deprivation therapy in prostate cancer: tamoxifen or radiotherapy? International journal of radiation oncology, biology, physics. 2012;83(4):e519-24.
48. Hussain M, Tangen CM, Berry DL, Higano CS, Crawford ED, Liu G, et al. Intermittent versus continuous androgen deprivation in prostate cancer. N Engl J Med. 2013;368(14):1314-25.
49. Royston P, Parmar MKB, Qian W. Novel designs for multi-arm clinical trials with survival outcomes with an application in ovarian cancer. Statistics in Medicine. 2003;22(14):2239-56.
50. Royston P, Barthel FMS, Parmar MKB, Choodari-Oskooei B, Isham V. Designs for clinical trials with time-to-event outcomes based on stopping guidelines for lack of benefit. Trials. 2011;12(1):81.
51. Royston P. nstage: MAMS trial sample size calculator. MRC Clinical Trials Unit, London2009.
52. Mason MD, Parulekar WR, Sydes MR, Brundage M, Kirkbride P, Gospodarowicz M, et al. Final Report of the Intergroup Randomized Study of Combined Androgen-Deprivation Therapy Plus Radiotherapy Versus Androgen-Deprivation Therapy Alone in Locally Advanced Prostate Cancer. J Clin Oncol. 2015;33(19):2143-50.
53. James ND, Sydes MR, Mason MD, Clarke NW, Anderson J, Dearnaley DP, et al. Celecoxib plus hormone therapy versus hormone therapy alone for hormone-sensitive prostate cancer: first results from the STAMPEDE multiarm, multistage, randomised controlled trial. The Lancet Oncology. 2012;13(5):549-58.

54. Haybittle JL. Repeated assessment of results in clinical trials of cancer treatment. Br J Radiol. 1971;44(526):793-7.
55. Peto R, Pike MC, Armitage P, Breslow NE, Cox DR, Howard SV, et al. Design and analysis of randomized clinical trials requiring prolonged observation of each patient. I. Introduction and design. Br J Cancer. 1976;34(6):585-612.

DocuSign Envelope ID: D18485C5-2BA5-4361-929F-A527801C0052

STAMPEDE

Systemic Therapy in Advancing or Metastatic Prostate Cancer: Evaluation of Drug Efficacy
A multi-arm multi-stage randomised controlled trial

MRC PR08

ISRCTN number:	ISRCTN78818544
EUDRACT number:	2004-000193-31
CTA number:	00316/0026/001-0001
NCT number:	NCT00268476

STATISTICAL ANALYSIS PLAN for analysis of the "Enzalutamide + abiraterone comparison" including appendix A describing meta-analyses of M0 and M1 patients with the "abiraterone comparison"

MRC Clinical Trials Unit at UCL

Tel: +44 (0)20 7670 4798
Fax: +44 (0)20 7670 4818
Email: mrcctu.stampede@ucl.ac.uk

Version date: 4.0, 07-June-2022
Will replace version 3.0 (30-July-2021)

Contacts
Laura Murphy
Chris Brawley
Louise Brown

This template and all preceding versions will be stored in the Statistical Analysis Master File for this trial held in S:\MRCCTU_Stampede_Stats\SAP

DocuSign Envelope ID: D18485C5-2BA5-4361-929F-A527801C0052

CONTENTS

1 ABBREVIATIONS 4

2 BACKGROUND AND DESIGN 6
2.1 TRIAL SUMMARY 6
2.2 COMPARISONS 7
2.3 ELIGIBILITY CRITERIA 9
2.4 METHOD OF RANDOMISATION 10
2.5 ALLOCATION RATIO 10

3 DATA COLLECTION AND PROCESSING 11
3.1 CASE REPORT FORMS AND VARIABLES 11
3.2 DATA COMPLETION SCHEDULE FOR CONSENT, BASELINE, TREATMENT & FOLLOW-UP ..11
3.3 DATA COMPLETION SCHEDULE FOR OTHER 'NON-SCHEDULED' CRFS 12
3.4 MANAGEMENT OF DATASETS 12
3.4.1 Data Cleaning 12
3.4.2 Data verification 13
3.4.3 Database Lock 14
3.5 DATA CODING 14
3.5.1 Free-text variables 14
3.6 PRE-PLANNED DATA CHECKS 14
3.7 DATA RETURNS 15
3.8 DATA MATURITY 15
3.9 MISSING DATA 15

4 OUTCOME MEASURES 16

5 SAMPLE SIZE CALCULATIONS 20
5.1 SAMPLE SIZE 20
5.2 SAMPLE SIZE FOR INTERMEDIATE ANALYSES 20
5.3 FURTHER DETAILS IN SAMPLE SIZE CALCULATIONS AND DESIGN 21

6 STATISTICAL ANALYSIS 22
6.1 TIME PERIODS 22
6.2 POPULATIONS FOR ANALYSIS 23
6.3 RECRUITMENT AND PATIENT FLOW 24
6.3.1 Recruitment 24
6.3.2 patient flow through the trial 24
6.4 BASELINE CHARACTERISTICS 24
6.5 STANDARD OF CARE TREATMENT 25
6.6 TRIAL TREATMENT 26
6.7 SURVIVAL OUTCOME MEASURES 27
6.7.1 primary outcome measure 27
6.7.2 secondary outcome measures 27
6.7.3 statistical analysis methods 27
6.7.4 Proportional and non-proportional hazards 28
6.7.5 Competing Risks 29
6.7.7 patients with no data post randomisation 29
6.7.8 withdrawn patients with outcome data 29
6.7.9 sensitivity analysis to assess impact of covid-19 pandemic 29
6.8 PRE-SPECIFIED SUBGROUP ANALYSES 29

DocuSign Envelope ID: D18485C5-2BA5-4361-929F-A527801C0052

6.8.1 Stratification Factors used for minimisation 30
6.8.2 OTHER PRE-SPECIFIED SUBGROUP ANALYSIS 30
6.8.2.1 Metastatic volume in M1 patients...30
6.9 PSA RESPONSE 30
6.10 TOXICITY AND SAFETY ANALYSES 31
6.11 QUALITY-OF-LIFE ANALYSIS... 33
6.12 HEALTH ECONOMICS 33

7 SIGNATURES OF APPROVAL 41

DocuSign Envelope ID: D18485C5-2BA5-4361-929F-A527801C0052

1 ABBREVIATIONS

Abbreviation	Expansion
**Abi**	Abiraterone
**ADT**	Androgen-deprivation therapy
**AMP**	Adenosine monophosphate
**AS**	Activity Stage
**BMD**	Bone mineral density
**CCI**	Comparison Chief Investigator
**Cel**	Celecoxib
**CHF**	Congestive heart failure
**CI**	Chief Investigator
**CI**	Confidence interval
**CRF**	Case Report Form
**CTCAE**	Common Toxicity Criteria for Adverse Events
**CTU**	Clinical Trials Unit
**CV**	Cerebrovascular
**DAB**	Dual Androgen Blockade
**DMP**	Data Management Plan
**Doc**	Docetaxel
**DAB**	Dual Androgen Blockade (previously Maximum Androgen Blockade [MAB])
**Enza**	Enzalutamide
**ES**	Efficacy Stage
**FFS**	Failure-free survival
**FPM**	Flexible parametric models
**HE**	Health Economics
**HEAP**	Health Economics Analysis Plan
**HR**	Hazard ratio
**HT**	Hormone therapy
**IDMC**	Independent Data Monitoring Committee
**ITT**	Intention-to-treat
**KM**	Kaplan-Meier
**LHRH**	Luteinising hormone-releasing hormone
**LOB**	Lack-of-benefit
**M0**	Non-metastatic
**M1**	Metastatic
**MACE**	Major adverse cardiac event
**MAMS**	Multi-arm multi-stage
**MCAR**	Missing completely at random
**MI**	Myocardial infarction
**MPFS**	Metastatic progression-free survival
**MRC**	Medical Research Council
**N+**	Lymph node-positive
**N0**	Lymph node-negative
**NX**	Lymph node stage unknown
**NSAID**	Non-steroidal anti-inflammatory drug
**ONS**	Office of National Statistics
**OS**	Overall survival
**PCa**	Prostate cancer
**PH**	Proportional hazards
**PHE**	Public Health England
**PSA**	Prostate specific antigen
**q6wk**	Every 6 weeks
**q12wk**	Every 12 weeks
**q6m**	Every 6 months

DocuSign Envelope ID: D18485C5-2BA5-4361-929F-A527801C0052

Abbreviation	Expansion
**q12m**	Every 12 months
**QL**	Quality of Life
**RMST**	Restricted mean survival time
**rPFS**	Radiological progression-free survival
**RT**	Radiotherapy
**RTOG**	Radiation Therapy Oncology Group
**SAE**	Serious adverse event
**SAP**	Statistical Analysis Plan
**SOC**	Standard-of-care
**SOP**	Standard Operating Procedures
**TBD**	To be determined
**TMG**	Trial Management Group
**TSC**	Trial Steering Committee
**WHO PS**	WHO Performance Status
**ZA**	Zoledronic acid

DocuSign Envelope ID: D18485C5-2BA5-4361-929F-A527801C0052

2 BACKGROUND AND DESIGN

This section gives a brief summary of the trial, including the trial aims/objectives; treatment/randomisation arms; main outcomes; and the patient eligibility criteria.

Full details of the background to the trial and its design are presented within the current, activated version of the protocol (Version 23.0; Oct-2021).

2.1 TRIAL SUMMARY

STAMPEDE is a multi-centre, platform protocol, including a number of randomised controlled trials. It recruits patients with locally advanced (M0) or metastatic (M1) prostate cancer who are starting long-term androgen-deprivation therapy (ADT) for the first time. Patients can have either newly-diagnosed disease or have been previously treated with radical radiotherapy or surgery but now have a rising prostate specific antigen (PSA). Further details on eligibility can be found in section 2.3.

The trial uses multi-arm, multi-stage (MAMS) methods to simultaneously assess a number of different research treatments. The trial aims to assess the effects of adding one or two approaches to the standard-of-care (SOC).

The investigational agents to date are:

(i) A bisphosphonate, zoledronic acid

(ii) A cytotoxic chemotherapeutic agent, docetaxel

(iii) A cyclooxygenase (Cox-2) inhibitor, celecoxib

(iv) A CYP-17 inhibitor, abiraterone

(v) Radiotherapy to the prostate amongst newly-diagnosed M1 patients only

(vi) An androgen receptor signalling inhibitor, enzalutamide

(vii) Metformin

(viii) Transdermal oestradiol (tE2)

Patients on the control arm receive the standard-of-care; the research arms have this standard supplemented with other potential treatments, except for patients allocated to the tE2 arm who receive transdermal oestradiol in place of standard hormone treatment.

The standard-of-care is based on ADT, achieved through the use of luteinising hormone-releasing hormone (LHRH) analogues or antagonists, Dual Androgen Blockade (DAB: long-term anti-androgens in combination with LHRH agonist) or bilateral orchidectomy according to local practice (bicalutamide for non-metastatic (M0) patients was allowed in some early versions of the Protocol). This standard-of-care is also the backbone of therapy for the research arms.

DocuSign Envelope ID: D18485C5-2BA5-4361-929F-A527801C0052

Standard-of-care radiotherapy (RT) was mandated for all N0M0 patients (unless contraindicated) and encouraged for N+M0 patients. RT to the prostate was permitted for M1 patients, too, although whilst the M1|RT research Arm H was recruiting, radiotherapy was not permitted as SOC for newly-diagnosed M1 patients and should only be received through allocation to Arm H for patients corresponding to this sub-set.

Standard-of-care docetaxel was permitted from Protocol version 14.0 (Dec-2015) onwards, following the results of STAMPEDE's "original comparisons"[1], along with other external trial results combined within a meta-analysis[2].

Planned use of docetaxel, as reported at randomisation, is included as a stratification factor from this time.

2.2 COMPARISONS

A research comparison is defined by those patients allocated to the research arm, along with the corresponding contemporaneously randomised, eligible control arm patients. See Table 1 for the definition of each research comparison within STAMPEDE to date.

Table 1: STAMPEDE Research Comparisons

Comparison name	Included Arms	Eligible patients	Accrual		Time Period(s)	NB
			Start Date	End Date		
"Zoledronic acid comparison"	A, B	All patients	05-Oct-2005	31-Mar-2013	1-4	Note
"Docetaxel comparison"	A, C	All patients	05-Oct-2005	31-Mar-2013	1-4	Note
"Celecoxib comparison"	A, D	All patients	05-Oct-2005	06-Apr-2011	1	Note
"Zoledronic acid + docetaxel comparison"	A, E	All patients	05-Oct-2005	31-Mar-2013	1-4	Note
"Zoledronic acid + celecoxib comparison"	A, F	All patients	05-Oct-2005	06-Apr-2011	1	Note
"Abiraterone comparison"	A, G	All patients	15-Nov-2011	17-Jan-2014*	3-5	Note
"M1|RT comparison"	A, H	Newly-diagnosed M1 pts<br>No contraindication to RT	22-Jan-2013	02-Sep-2016	4-9	Note
"Enzalutamide + abiraterone comparison"	A, J	All patients	29-Jul-2014	31-Mar-2016	7-9	Note
"Metformin comparison"	A, K	Non-diabetic pts<br>No contraindication to metformin	05-Sep-2016	TBD	10-TBD	---
"tE2 comparison"	A, L	<8wk anti-androgen use<br>Maximum 4wk LHRH t'py<br>No bilateral orchidectomy	20-Jun-2017	TBD	11-TBD	---

***Note:** One patient was manually randomised to Arm G after the cut-off of 17-Jan-2014

D, F: The celecoxib-containing arms closed accrual early due to lack of sufficient activity following their Activity Stage 2 analysis.

B, C, E: The remaining original research arms closed to recruitment having reached an acceptable sample size (based on time to analysis projections).

G: The abiraterone arm closed to recruitment having reached its revised sample size target (1800 pts) ahead of schedule.

H: The M1|RT arm closed to recruitment having reached its revised target sample size ahead of schedule.

J: The enzalutamide + abiraterone arm closed to recruitment having reached its target sample size ahead of schedule.

[1] http://www.thelancet.com/pdfs/journals/lancet/PIIS0140-6736(15)01037-5.pdf
[2] http://www.thelancet.com/pdfs/journals/lanonc/PIIS1470-2045(15)00489-1.pdf

DocuSign Envelope ID: D18485C5-2BA5-4361-929F-A527801C0052

The trial is summarised at relevant times for this comparison in Figures 1 and 2. The trial initially started with Arms A to F. Additional research arms have been included in the trial over time.

At the time a new research arm is activated, the strata totals in the randomisation system are reset; the team checks for imbalances before this occurs. Consideration is given to applying a small weight to the reset total to help correct this only if there is a major imbalance, defined as a difference of 12 patients or more in any given strata (NB to date this has not been required).

Figure 1: Recruiting arms from Jul-2014 to Mar-2016 (Protocol version 12.0)

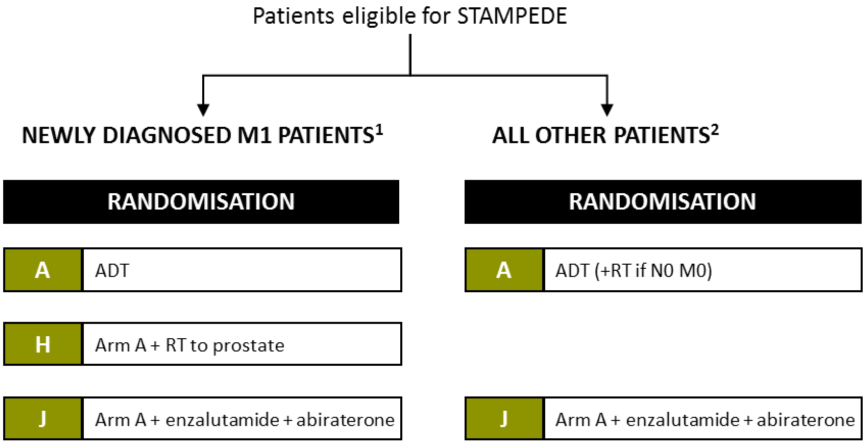

[1] except pts with a contra-indication to RT
[2] all suitable pts with newly diagnosed locally advanced disease should also have RT[1]

DocuSign Envelope ID: D18485C5-2BA5-4361-929F-A527801C0052

Figure 2: Activity-by-time graph showing patients contributing to this comparison

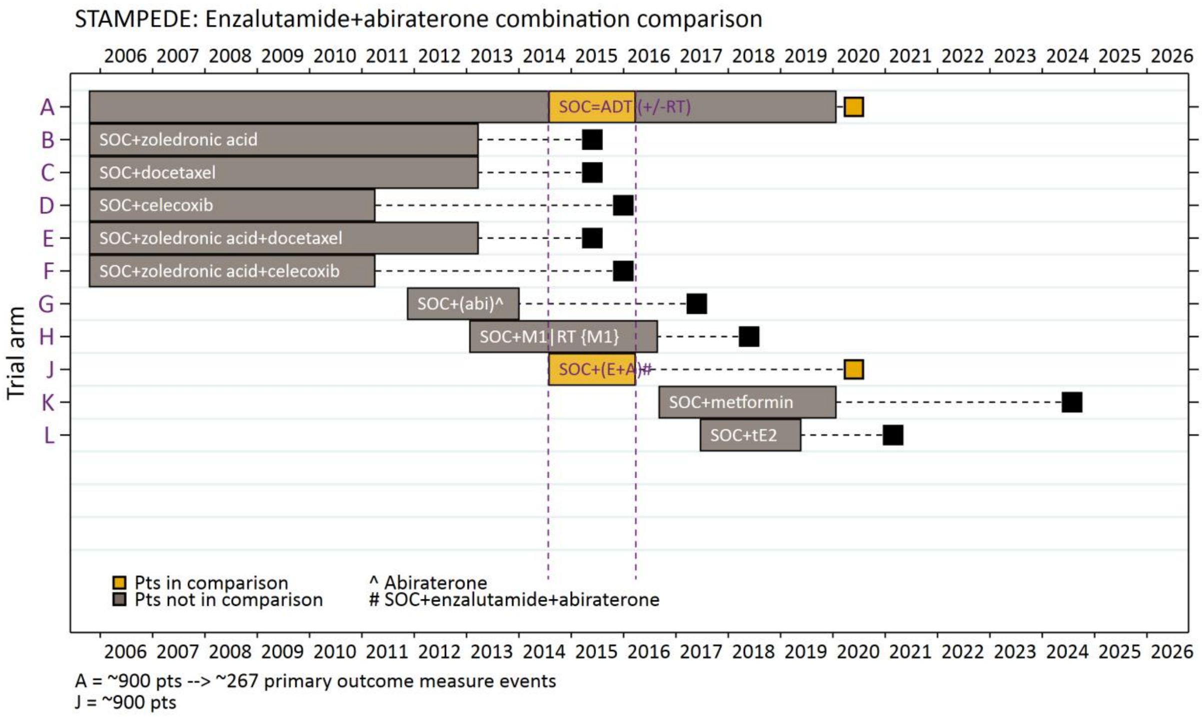

2.3 ELIGIBILITY CRITERIA

The eligibility criteria describe a broad population of patients unified by the need to start long-term ADT for the first time.

In the broadest terms, this includes patients who have had previous local therapy and now have high-risk relapse, along with patients with newly-diagnosed disease. For this comparison, this included patients with:

- High-risk locally advanced disease (at least two of: Stage T3/4 N0 M0 histologically confirmed prostate adenocarcinoma, PSA≥40ng/ml or Gleason sum score 8-10)
- Nodal involvement
- Metastatic disease

All patients need to give written consent, be sufficiently fit for any protocol treatment and follow-up, and meet other detailed eligibility criteria.

The details of the eligibility criteria have changed a little over time; this is shown by protocol version in a specific document [3] which will continue to be updated throughout the trial.

[3] S:\MRCCTU_Stampede\10 Audit & Quality Control\10.1_Monitoring & QC\10.1.1_Monitoring Tools

DocuSign Envelope ID: D18485C5-2BA5-4361-929F-A527801C0052

2.4 METHOD OF RANDOMISATION

Patients are randomised centrally using a computerised algorithm developed and maintained by the CTU. Randomisation is performed using the method of minimisation over a number of clinically important stratification factors with an additional random element. These factors are:

• Randomising centre	each centre
• Metastases	M0 vs M1
• Nodal involvement	N0 vs NX vs N+
• Age at randomisation	up to 69yrs vs 70yrs and over
• WHO performance status	PS=0 vs PS=1-2
• Method of ADT[4]	Orchidectomy vs LHRH agonist vs LHRH antagonist vs Dual Androgen Blockade (DAB)
• Regular aspirin or NSAID use at baseline	yes vs no
• Radiotherapy planned[5]	yes vs no
• Docetaxel planned[6]	yes vs no

When implementing the additional random element of the randomisation, an 80% probability of allocation will be split between the (one or more) arms with the lowest strata totals (i.e. 80% probability of being allocated to one of the minimising arms); and the remaining 20% probability of allocation will be split between the remaining (one or more) arms. This method should provide simplicity of reporting and implementation.

2.5 ALLOCATION RATIO

As of Oct-2021 (Protocol version 23.0), the trial had one control arm and ten research arms. Overall, the allocation ratio has been A:B:C:D:E:F:G:H:J:K:L = 2:1:1:1:1:1:2:2:2:2:2.

Therefore, this comparison had equal weighting for the research and control patients in comparison-eligible patients i.e. allocation ratio 1:1.

[4] Method of ADT options have changed over time, from LHRH vs orchidectomy, to then include bicalutamide, then specify LHRH agonist or antagonist and more recently exclude bicalutamide but include DAB; see S:\\MRCCTU_Stampede_Stats\\SAP\\Stratification_Factors_OverTime

[5] "Radiotherapy planned" was added as a stratification factor at the start of recruitment to Efficacy Stage I for the "original comparisons" (Mar-2008)

[6] Docetaxel planned was added as a stratification factor from 17-Dec-2015 following publication of the "original comparisons" results indicating docetaxel improved overall survival

3 DATA COLLECTION AND PROCESSING

3.1 CASE REPORT FORMS AND VARIABLES

Full details of data collection and timings are described in the protocol[7].

A copy of the Case Report Forms (CRFs) and Quality of Life (QL) questionnaires are presented in the Statistical Master File and the Trial Master File. Details of the variables, and any corresponding validations, are presented in the metadata which forms part of the Trial Master File.

3.2 DATA COMPLETION SCHEDULE FOR CONSENT, BASELINE, TREATMENT & FOLLOW-UP

A record of consent to trial participation in the J comparison is held by the MRC CTU for 1773/1976 patients randomised; for the remaining 203 patients, consent was collected by the local site prior to randomisation but a copy of the completed consent form was not available to the MRC CTU at the time the data were frozen. All patients are included in analyses to maintain the ITT integrity of the trial results.

Table 2 gives detail on the expected timing of scheduled CRFs. Please note that this only applies to arm J; please see earlier versions of the SAP for detail of timings for other comparisons.

Table 2: Timings for completion of scheduled CRFs on arm J

Timing of Assessment		Baseline		Treatment		Outcomes		Freq
		Rand[N]	Pre-Trt	RT	SOC Doc	Follow-up	QL HE	
Yr 0	Wk 0	✓	✓				✓	
	Wk 6					✓	✓	6 weekly
	Wk 12					✓	✓	
	Wk 18					✓	✓	
	Wk 24				J only	✓	✓	
	Wk 36					✓	✓	12 weekly
	Wk 48			M0 only		✓	✓	
	Wk 60					✓	✓	
	Wk 72					✓	✓	
	Wk 84					✓	✓	
	Wk 96					✓	✓	
Yr 2	Month 24					✓	✓	6 monthly
	Month 30					✓	✓	
Yr 3	Month 36					✓	✓	
	Month 42					✓	✓	
Yr 4	Month 48					✓	✓	
	Month 54					✓	✓	
Yr 5	Month 60					✓	✓	
Yr 6+						✓	✓	annual

Note:

[7] S:\MRCCTU_Stampede\01 Study Protocol_Key Documents\1.1 Signed Protocol

DocuSign Envelope ID: D18485C5-2BA5-4361-929F-A527801C0052

Pre-Trt assessment includes the following CRFs: baseline, cardiovascular, bone density risk factor questionnaire.

QL & HE data were collected for the first 700 patients randomised and then for all patients randomised from Protocol v8.0 onwards (Nov-2011) who opt-in.

QL & HE data is collected until disease progression.

SOC Doc forms are required for all patients randomised from 17-Dec-2015, when the change in SOC to permit upfront docetaxel use was implemented, and for any patients randomised before this date and receiving upfront docetaxel.

3.3 DATA COMPLETION SCHEDULE FOR OTHER 'NON-SCHEDULED' CRFS

The following CRFs should be completed as required:

- Hormone therapy log (All Arms)
- Abiraterone & enzalutamide treatment log (Arms G & J)
- Toxicity form (All Arms)
- RT acute toxicity form (All Arms, pts who received RT only)
- Progression log (All Arms)
- Additional treatment log (All Arms)
- Saliva pathology form (All Arms)
- Blood form (All Arms)
- SAE form (All Arms)
- End of research treatment form (Research Arms only)
- Co-enrolment form (All Arms)
- Death form (All Arms)

For all CRFs, details of associated timings and requirements are given within their guidance notes.

3.4 MANAGEMENT OF DATASETS

The datasets will be managed in accordance with CTU Standard Operating Procedures (SOPs) and in accordance with the Data Management Plan[8]. Full details of data cleaning, verification and lock procedures are contained in this document.

3.4.1 DATA CLEANING

Routine data extracts are run on an approximate fortnightly basis. In preparation for an IDMC review and interim or final efficacy analysis, several of these extracts will be used, at least one to check the data and a final one for analysis. The statistician will work with the data managers to resolves as many queries and discrepancies as possible (entered onto MACRO prior to the final freeze) and this process will be formally documented with the exact details agreed prior to the analysis in the in the Database Lock Quality Checklist.

If any outstanding data queries are resolved during the analysis that relate to data in the final copied dataset (eg. problems that are found during analysis or amended CRFs that are returned to CTU), and it is desired to

[8] S:\MRCCTU_Stampede\08 Data Management\8.6 Data Management Plan

DocuSign Envelope ID: D18485C5-2BA5-4361-929F-A527801C0052

include these changes in the report or analysis being run, corrections should be made in MACRO as usual but the data should not be extracted again. Rather, the corrections should be made in the Statistician's analysis files and appropriately highlighted and annotated (notably any confirmed changes should only be made within cr_fieldupdates.do). For the primary analysis the Senior Statistician will decide on a case-by-case basis whether a correction warrants exporting the data again.

Datasets for all analyses will be constructed and analyses performed using Stata programs (*.do).

3.4.2 DATA VERIFICATION

Data verification, consistency and range checks are performed at the data entry stage by the CTU, as well as checks for missing data, as documented in the trial working practices (copies of these checks can be found in the Trial Master File). Additional range, consistency and missing data checks will be performed automatically within the MACRO databases, as programmed in the database. Copies of the data chase and query report forms are stored electronically, with completed and returned forms stored in patient files. Some of the checks may be duplicated and others will be performed when the datasets for analysis are constructed by the statistician. All outcome variables (primary and secondary) including treatment details will be examined for unusual, outlying, unlabelled or inconsistent values by the Statistician.

In general, given the thorough nature of our follow-up procedure we expect the issue of missing data to be relatively minimal. We anticipate high compliance with initial data collection as this is close to the time of patient registration. If any data is missing, imputation will not routinely be done for the main analyses, but this may be reconsidered if recommended or requested. Sensitivity analyses may be undertaken which include additional variables in statistical models, e.g. alternative adjusted Cox models including other variables considered to be predictive of the outcome. For these analyses, missing values of adjustment variables may be multiply imputed under the Missing At Random (MAR) assumption to avoid reliance on complete case analysis.

Data from sites on overall survival, the definitive primary outcome for patients with baseline M1 disease and a component of the metastasis-free survival primary outcome for patients with baseline M0 disease, and other outcome measures may be supplemented by linked data from the Office of National Statistics (ONS) or a similar system. This would be used to ascertain the final survival status of those not reported as having died on the most recent follow-up, and additionally to help in the ascertainment of causes of death.

Any problems with trial data will be queried with the Trial Managers, Data Managers, or Statistician(s), as appropriate. Where possible, data queries will be resolved, although it is accepted that due to administrative reasons and data availability, the dataset cannot be perfect. Evidence of preferential data collection on one arm would be unacceptable and further data cleaning would be required.

Prior to formal final primary analyses an additional set of checks will be completed. These include a Trial Manager checking primary outcome data in MACRO against the original CRFs (including date of death) for a sample of patients to ensure concordance. These checks are detailed in the Trial Management File, kept by the STAMPEDE Trial Managers.

DocuSign Envelope ID: D18485C5-2BA5-4361-929F-A527801C0052

3.4.3 DATABASE LOCK

A copy of the trial databases will be locked before the primary activity analysis for each research comparison once the Database Lock Quality Checklist has been agreed and the conditions met. However, the live databases will remain open, as long-term follow-up data for patients may be collected in the database after this final activity stage analysis. Additionally, some patients contribute to multiple comparisons that may mature at differing times.

3.5 DATA CODING

The coding of the raw variables can be found in the metadata[9]. The latest version has the largest version number "X" at the end of the document name.

Coding of variables for analysis can be found in the Analysis File Specification documentation[10].

3.5.1 FREE-TEXT VARIABLES

If required, review and (re-)coding of free-text variables is carried out by a clinical member of the TMG.

Such free-text variables to be reviewed and (re-)coded include:

- Those items reported as "other, specify", including reason for treatment action; toxicity; additional treatment; and place of death.
- Concomitant medications (collected as free-text at baseline; up to Protocol v15.0, Sep-2016)

3.6 PRE-PLANNED DATA CHECKS

- Current ADT on randomisation form *vs* baseline form (patients randomised before 05-Sep-2016; check for concordance)
- Broad disease category *vs* [pre-HT PSA value, metastatic status, nodal status, gleason score, previous treatment reported]; check for concordance
- Disease progression on FU form *vs* progression form; check for concordance
- Death on progression form *vs* death form [deaths reported before 05-Sep-2016]; check for concordance
- Death reported as an SAE on Death CRF *vs* SAE form with death reported
- Metastatic status (M0 versus M1) concordance between randomisation server and subsequent randomisation form. In a small number of patients, the assessment of metastatic status at randomisation can change on the basis of subsequent testing and imaging such that the randomisation form becomes a more correct representation of the true metastatic status of the patient. For the purposes of separating M0 and M1 analyses, the data from the randomisation form (rather than the rando server) will be used to select patients. The number of patients in whom this status changed will be described in any data report. It is acknowledged that the minimisation factors will differ slightly from the true status.

[9] S:\MRCCTU_Stampede\08 Data Management\8.4 Metadata and Data Handling\Metadata\STAMPEDE Metadata vX

[10] S:\MRCCTU_Stampede_Stats\Data\Auto_Macro\Analysis File Spec

DocuSign Envelope ID: D18485C5-2BA5-4361-929F-A527801C0052

3.7 DATA RETURNS

- Data Forms returned compared to expectations (routine forms only; randomisation, baseline (incl. cardiovascular & BD Risk), follow-up, radiotherapy detail/acute toxicity)
- Death, progression and end of trial participation forms received for the control arm
- FU forms expected vs number of FU forms received, for each patient
- Scatter plot of timing of most recent received FU for each patient *vs* time from randomisation, by treatment arm (including detail on number of patients randomised, number of deaths, number of withdrawals, number okay and number late for follow-up)
- Number of FU forms dated within each week since patient's randomisation
- Time since last FU form (KM graph; subgroup: randomised <24wks vs 24+wks)
- Duration of follow-up after randomisation (time from randomisation to last contact), by arm, with median using reverse KM plot
- For surviving patients, the time since last follow-up form received, by treatment arm
- Details of patients Lost-to-FU or for whom early stopping of follow-up and data collection has been reported

3.8 DATA MATURITY

Median follow-up time will be calculated using a "reversed" Kaplan-Meier approach, taking censor date (if alive) to be an event and death as the time of censorship. The median follow-up time will be detailed by arm and within any pre-defined sub-groups of interest. In patients who were last known to be alive, time to last follow-up will be presented using standard summary statistics. Date last seen (if alive) is as defined within the censor date in Table 4.

3.9 MISSING DATA

Missing data will be assumed to be missing completely at random (MCAR) in the main intention-to-treat analyses. Information on covariates included in the main pre-specified analysis models is collected at randomisation so these key variables should be complete in most cases.

Where appropriate, for sensitivity analyses and additional exploratory analyses, missing data on explanatory variables to be included in analysis models may be multiply imputed using the Missing At Random (MAR) assumption to avoid reliance on complete-case analysis. Evidence in favour of the MAR assumption will be presented at the same time and sensitivity analyses conducted to assess the impact of data being Missing Not At Random (MNAR) on estimates of treatment effect.

DocuSign Envelope ID: D18485C5-2BA5-4361-929F-A527801C0052

4 OUTCOME MEASURES

For all outcome measures the comparison is between patients on the relevant research arm and those contemporaneously randomised control arm patients eligible for the research arm of interest. Outcomes are listed in Table 3 and definitions are provided in Table 4.

Note: All arms are un-blinded so primary outcome measures for all comparisons are objectively measured, with caution to be taken around interpretation of more subjective secondary outcome measures such as symptomatic skeletal events.

As for other comparisons, the "enzalutamide+abiraterone comparison" (A-J) was originally designed to use Failure-Free-Survival (FFS) as the intermediate outcome and Overall Survival (OS) as the final primary outcome. However, the ICECaP meta-analysis of patients with M0 disease has demonstrated that Metastasis-Free-Survival (MFS), defined as time from randomisation to distant metastases confirmed by imaging or histological evidence or death from any cause, is a robust surrogate outcome measure for OS in this M0 patient group (9). Therefore, MFS was added to the list of outcome measures in 2021 and will become the primary outcome measure for M0 patients in the combined A-G and A-J analysis. Details for this analysis are provided in Appendix A. Primary outcome measure remains OS for M1 patients and details for meta-analysis methods combining the A-G and A-J comparisons are also provided in Appendix A.

Table 3: Trial outcome measures by comparison stage for the A-J comparison

COMPARISON STAGE	PRIMARY OUTCOME MEASURE	SECONDARY OUTCOME MEASURES[T]
**Pilot phase(s)**	Safety[*]	Feasibility
**Activity Stages**	Failure-free survival (FFS)[†]	Overall survival (OS)[x]<br>Biochemical Failure<br>Progression-free-survival (PFS)<br>Disease-specific survival<br>Non-PCa death<br>Lymph node progression<br>Distant metastases (Metastatic Progression-Free Survival)<br>Toxicity<br>Skeletal related events (SREs)<br>Therapy for progression
**Efficacy Stage**	Overall survival [x]	All OMs as for Activity Stages plus:<br>Metastasis-Free-Survival^<br>Cost effectiveness

[T] Presented at activity stages where data is mature

[*] Based on toxicity

[†] Including biochemical failure (see Table 4 for definition of FFS)

[x] Cause of death with a view to cause-specific survival from PCa (with death from other causes as a competing risk) is considered under this.

^ As defined in ICECaP 2017 *JCO* paper: DOI: 10.1200/JCO.2017.73.9987

DocuSign Envelope ID: D18485C5-2BA5-4361-929F-A527801C0052

Table 4: Definition of outcome measures and censoring dates

TERM	DEFINITION
Overall survival (OS)	Time from randomisation until death from any cause. For surviving patients, censor date 1 is used; if ONS data is available use censor date 2.
Failure-free Survival (FFS)	Time from randomisation until the first of the following events:<br>• Biochemical failure<br>• Local progression<br>• Lymph node progression<br>• Distant metastases<br>• Skeletal Related Event (where confirmed disease progression)<br>• Death from prostate cancer<br>For patients who have not had an event, censor date 3 is used (see below).<br>If a suspected event is reported for any of: local progression, lymph node progression, distant metastases progression this will be counted as a FFS event.
Progression-free Survival (PFS)	Time from randomisation until first of:<br>• Local progression<br>• Lymph node progression<br>• Distant metastases<br>• Skeletal Related Event (where confirmed disease progression)<br>• Death from prostate cancer<br>For patients who have not had an event, censor date 3 is used (see below).<br>If a suspected event is reported for any of: local progression, lymph node progression, distant metastases progression this will be counted as a PFS event.
Metastatic Progression-Free Survival (mPFS)	Time from randomisation until first of:<br>• Distant metastases<br>• Skeletal Related Event (where confirmed disease progression)<br>• Death from prostate cancer<br>For patients who have not had an event, censor date 3 is used (see below).<br>If a suspected event is reported for distant metastases progression this will be counted as an MPFS event
Metastasis-Free Survival (MFS)	Time from randomisation until first of:<br>• Radiologically-confirmed distant metastases<br>• Death from any cause<br>For patients who have not had an event, censor date 3 is used (see below).
Skeletal Related Event (SRE)	• Bone pain requiring radiotherapy and/or surgery<br>• Pathological fracture with or without disease progression at that cancer site<br>• Metastatic spinal cord compression
Disease-specific survival	Time from randomisation until death from prostate cancer (see below).<br>For patients who have not had an event, censor date 1 is used; if ONS data is available use censor date 2 (see below).

DocuSign Envelope ID: D18485C5-2BA5-4361-929F-A527801C0052

<table>
<tr><th>TERM</th><th>DEFINITION</th></tr>
<tr><td>Death from prostate cancer</td><td>All deaths are reviewed following the death review process[11]. A Statistician runs and maintains the Stata program to automatically assign either PCa or non-PCa as a cause of death with the following rules:
<table>
<tr><th colspan="2">Rule</th><th>Cause death</th></tr>
<tr><td>1</td><td>Primary cause of death is PCa and no secondary causes are reported; progression event prior to death; no evidence of another cancer as an SAE</td><td>PCa</td></tr>
<tr><td>2</td><td>Primary cause of death is pneumonia and secondary cause of death is PCa; progression event prior to death</td><td>PCa</td></tr>
<tr><td>3</td><td>Primary cause of death is neutropenic sepsis and secondary cause of death is PCa; progression event prior to death</td><td>PCa</td></tr>
<tr><td>4</td><td>Primary cause of death is carcinomatosis and secondary cause of death is PCa; progression event prior to death</td><td>PCa</td></tr>
<tr><td>5</td><td>Death is reported as caused by PCa treatment; progression event prior to death</td><td>PCa</td></tr>
<tr><td>8</td><td>Site reports on Death CRF (version 13.0+) that responsible consultant considers death predominantly caused by PCa or protocol research / standard-of-care treatment</td><td>PCa</td></tr>
<tr><td>6</td><td>Primary cause of death is other primary cancer, and is confirmed by SAE report</td><td>Non-PCa</td></tr>
<tr><td>7</td><td>Primary cause of death is cardiovascular disease; PCa not listed as secondary cause of death</td><td>Non-PCa</td></tr>
<tr><td>9</td><td>Site reports on Death CRF (version 13.0+) that responsible consultant considers death predominantly caused by cardiovascular disease or other condition</td><td>Non-PCa</td></tr>
</table>

Any patients that cannot by classified by these rules will be reviewed by a clinician. Further information will be sought where there is insufficient information for the reviewer(s) to make a judgement. Where the review is not completed for any reason, the local investigator’s opinion will be taken.</td></tr>
<tr><td>Censor date 1 for OS</td><td>Date taken from the latest of the relevant variables defined below:

Date of randomisation (Form 1)
BMD assessment date (scan, blood sample, urine sample)
Date of treatment cycle (as taken from the bisphosphonate, docetaxel; Forms 4, 5, 6)
Date bloods taken (as taken from the bisphosphonate Forms 4, 5)
Date of last SOC docetaxel cycle (Form 21)
Date of any treatment action (Forms 7, 7B, 7C, 7D)
Date of any tE2 treatment action for Arm L patients (Form 25)
Date of tests recorded on hormone results log for Arm L patients (Form 24)
Dates reported on the Follow-up CRF (including date of PSA tests, date of any surgical interventions, date of any SRE, date of any metabolic or cardiovascular event; Forms 7, 7A)
Date of any reported progression event (Form 8)
Date additional treatment started or stopped (Forms 8, 8A)
Date of first/last RT fraction (Form 9A)
Date of late RT toxicity assessment (Form 10)
Date HT/research treatment ended (Form 11)
SAE date (onset, resolved, recent HT or trial treatment administration, start/end date of other treatment, test date) (Form 14)
Date of palliative RT fraction (Form 19)
Date blood or saliva sample obtained as reported on the pathology form (Form 18)
Date of co-enrolment to another trial (Form 15)
Date trial participation ended (Form 20)
Date last known alive (Form 7 from Version 13.0)
Date of death (Censoring date only for outcomes other than overall survival and disease-specific survival; Form 12)

Notes:</td></tr>
</table>

[11] : S:\MRCCTU_Stampede_Stats\Data\Death Review\

DocuSign Envelope ID: D18485C5-2BA5-4361-929F-A527801C0052

TERM	DEFINITION
	• Dates from the QoL forms are no longer used as a censor date as these are completed by the patient and cannot be queried for errors.<br>• Dates of form completion are no longer used as the CRF may have been completed retrospectively.<br>• Any date pre-randomisation is ignored within the calculation.<br>Unusual dates which have not yet been resolved or dates after the date of the corresponding data freeze will be ignored for the purposes of calculating this censor date.
Censor date 2 for OS	For patients with successful flagging with ONS (or equivalent) a censoring date will be set as 4 or 8 weeks before the ONS data transfer
Censor date 3 for non-OS outcome measures	Date taken from the latest of the relevant variables defined below:<br>• Date of randomisation (Form 1)<br>• Date of assessment on the Follow-up CRF (unless recorded as a missed visit; Form 7)<br>• Date of any reported progression event (If type of progression not included as event; Form 8)

5 SAMPLE SIZE CALCULATIONS

5.1 SAMPLE SIZE

This comparison includes patients allocated to research Arm J and all patients contemporaneously allocated to the control Arm A who meet the eligibility criteria for the research treatment. The sample size calculations were performed using the latest update of –nstage- available at the time, with an allocation ratio of 1:1.

This comparison was added in Jul-2014 and was designed under similar assumptions as the original research comparisons; however a smaller HR for the intermediate outcome measure of 0.70 was targeted with only 2 pre-planned activity analyses.

Consideration was given to ceasing further randomisations to Arm J if it was not showing sufficient evidence of activity on the intermediate primary outcome measure (FFS), just as for the other research arms.

The patient mix for this comparison is likely to represent a more favourable prognosis on average than in earlier comparisons, due to concurrent recruitment of M1 but not M0 patients to Arm H (resulting in a lower proportion of M1 patients in the "enzalutamide + abiraterone comparison").

We anticipated that around 1,800 patients needed to be recruited within 3.5 years to observe ~269 control arm deaths within 6 years. For our target HR of 0.75 for SOC+enzalutamide+abiraterone vs SOC alone on OS this would give 90% power to detect a treatment difference of this magnitude with a one-sided significance level of 2.5%. The timing of the definitive analysis will be partly dependent on the observed overall survival. The default scenario assumed that (i) recruitment is constantly 70pts/m to the trial overall, (ii) the M1|RT Arm H accrues throughout and (iii) a further new research arm with an equal allocation ratio is introduced 18 months after activation of Arm J.

Note that these sample size calculations have been superseded by the new analysis plan set out for M0 patients in Appendix A but are presented for completeness and transparency.

5.2 SAMPLE SIZE FOR INTERMEDIATE ANALYSES

Intermediate analyses will be collated and reported to the IDMC. Data will be released to the TSC if required and on to the TMG if deemed appropriate. Not all comparisons will be featured in every intermediate reporting, depending on the stage of the trial, the need for the analysis and time pressures.

Table 5 gives details of the intermediate analyses for all research comparisons.

Table 5: Intermediate and Final Activity Stage Analyses – Targeted vs Observed events

Research Comparison	AS#	Control Arm Events		IDMC / Main Report	IDMC Recommendation
		Target	Observed		
Original (Arms B-F)	1	113 FFS	129 FFS	30-Mar-2010	Continue as planned
(Arms B-F)	2	216 FFS	209 FFS	31-Mar-2011	Stop accrual to arms D&F
(Arms B,C,E)	3	334 FFS	341 FFS	30-May-2012	Continue as planned
(Arms B,C,E)	4	404 OS	415 OS	13-May-2015	Main report
Abiraterone (Arm G)	1	75 FFS	88 FFS	30-Aug-2013	Continue as planned
	2	142 FFS	162 FFS	07-Feb-2014	Continue as planned
	3	221 FFS	241 FFS	06-Jun-2014	Continue as planned
	4	267 OS	262 OS	10-Feb-2017	Main report
M1|RT (Arm H)	1	75 FFS	69 FFS	06-Jun-2014	Continue as planned
	2	142 FFS	144 FFS	21-Nov-2014	Continue as planned
	3	221 FFS	255 FFS	13-May-2015	Continue as planned
	4	267 OS	TBD OS	Q2/3-2018	Main report
Enzalutamide+abiraterone (Arm J)	1	66 FFS	85 FFS	05-Nov-2015	Continue as planned
	2	139 FFS	136 FFS	22-Mar-2016	Continue as planned
	3	269 OS	345 OS	21-Jun-2021	Main report
Metformin (Arm K)	1	121 OS*	TBD OS*	TBD	TBD
	2	473 OS*	TBD OS*	TBD	Main report

* Events in M1 patients only

5.3 FURTHER DETAILS IN SAMPLE SIZE CALCULATIONS AND DESIGN

For further details relating to the sample size calculations and design for each research comparison, see the Statistical Design Document. All do-files, Stata logs, and related presentations can be found within the relevant section of the Statistical Master File.

DocuSign Envelope ID: D18485C5-2BA5-4361-929F-A527801C0052

6 STATISTICAL ANALYSIS

The results of the analyses will be reported following the principle of the ICH E3 guidelines on the Structure and Content of Clinical Study Reports.[12]

6.1 TIME PERIODS

The main analysis for all comparisons using Cox PH models will be stratified by each time period when the choice of treatment allocations at randomisation within STAMPEDE was changed, or another fundamental aspect which may affect the patient population being randomised. As of Jun-2017, there are eleven time-defined strata (see Table 6 and Figure 3 below). The main analysis of the enzalutamide + abiraterone comparison includes periods 7 and 8.

Table 6: Time periods within STAMPEDE

Time Period	Definition	Accrual		Co-recruiting research arms
		Start date	End date	
1	From the start of the trial up to the stopping of the celecoxib-containing research Arms D & F	05-Oct-2005	06-Apr-2011	B C D E F
2	Post-closure of Arms D & F up to the opening of the abiraterone research Arm G	06-Apr-2011	14-Nov-2011	B C E
3	Post-opening of Arm G up to the opening of the M1 radiotherapy research Arm H	15-Nov-2011	21-Jan-2013	B C E G
4	Post-opening of Arm H up to the closure of the remaining original research Arms B, C & E	22-Jan-2013	31-Mar-2013	B C E G H
5	Post-closure of Arms B, C & E up to the closure of abiraterone research Arm G	01-Apr-2013	17-Jan-2014*	G H
6	Post-closure of Arm G up to the opening of the enzalutamide+abiraterone research Arm J	18-Jan-2014	28-Jul-2014	H
7	Post-opening of Arm J up to the update in SOC to permit planned use of docetaxel as first line treatment	29-Jul-2014	16-Dec-2015	H J
8	Post-update of SOC up to the closure of enzalutamide+abiraterone research Arm J	17-Dec-2015	31-Mar-2016	H J
9	Post closure of Arm J up to the close of M1|RT research Arm H and opening of metformin research Arm K	01-Apr-2016	02-Sep-2016	H
10	Post-opening of Arm K to the opening of transdermal oestradiol research arm L	03-Sep-2016	19-Jun-2017	K
11	Post-opening of Arm L onwards	20-Jun-2017	TBD	K L

***Note:** One patient was manually randomised to Arm G after the cut-off of 17-Jan-2014

[12] http://www.ich.org/fileadmin/Public_Web_Site/ICH_Products/Guidelines/Efficacy/E3/E3_Guideline.pdf

DocuSign Envelope ID: D18485C5-2BA5-4361-929F-A527801C0052

Figure 3: Arms active over time (time strata)

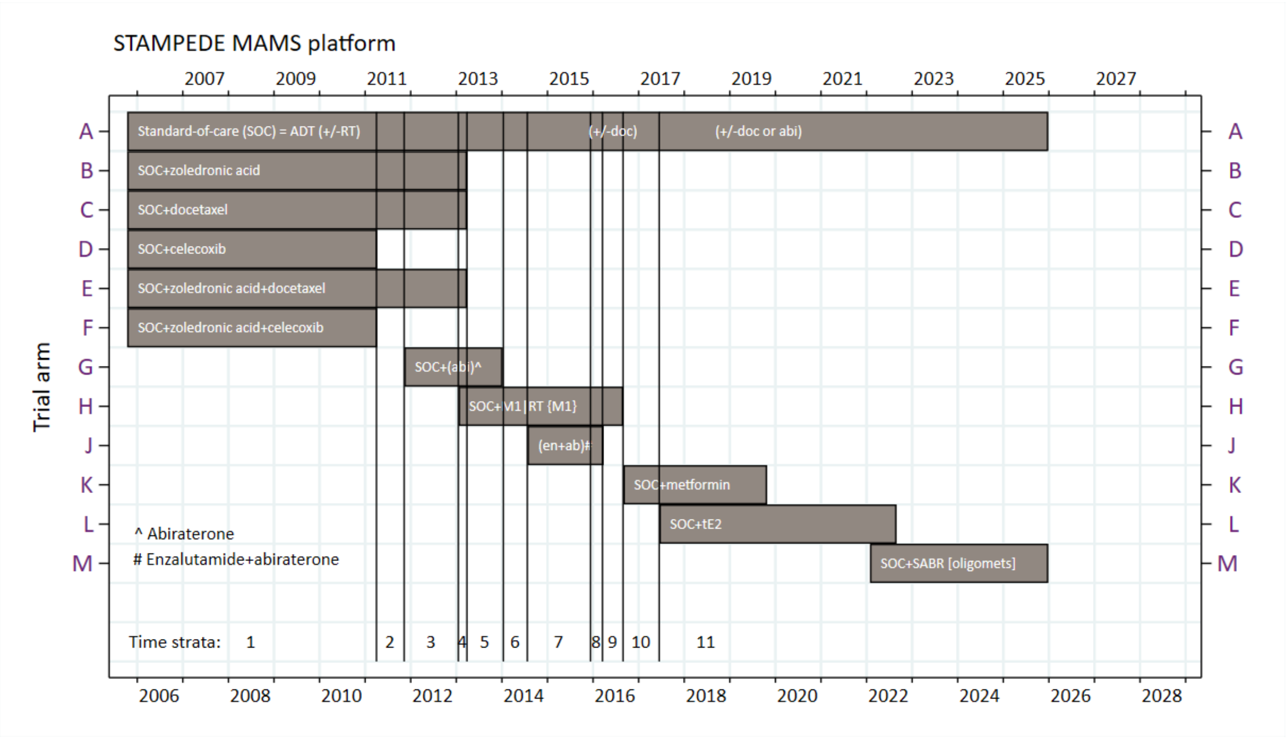

6.2 POPULATIONS FOR ANALYSIS

We define two populations for analysis; the intention-to-treat population and the safety population. The ITT population will be used for all analyses unless specified. The safety population will be included in analyses of adverse events, toxicity and other safety data (safety analyses).

Intention-to-treat (ITT) population

- Comprised of all randomised patients, whether or not they actually received the allocated trial treatment.
- In ITT analyses by treatment arm, patients will be included in the treatment arm to which they were randomised.

Safety population

- Comprised of patients who have been administered at least one dose of their allocated trial treatment.
- Patients will be classed as having started trial treatment if they report a treatment start date, report an SAE which is treatment related, or report death as being within 4 weeks of trial treatment (& related to trial treatment)
- In safety population analyses, patients will be included in the treatment arm corresponding to the treatment they actually started; for example, an Arm G or J patient not starting abiraterone or enzalutamide will be excluded from the analysis.

DocuSign Envelope ID: D18485C5-2BA5-4361-929F-A527801C0052

For visual illustration, a CONSORT flow diagram will clearly identify any patients found to be ineligible post-randomisation or stopping trial follow-up early; these patients will be included in relevant analyses where possible. For reference, a template flow diagram can be found in S:\\MRCCTU_Stampede_Stats\\SAP.

6.3 RECRUITMENT AND PATIENT FLOW

6.3.1 RECRUITMENT

- Graph of observed recruitment to the relevant comparison
- Table of recruitment by year & centre
- Table of recruitment across relevant time strata, by treatment arm

6.3.2 PATIENT FLOW THROUGH THE TRIAL

The flow of patients through the trial during the time the relevant comparison was recruited will be presented in a CONSORT diagram that also presents the ITT and safety populations (see section 6.2).

6.4 BASELINE CHARACTERISTICS

The following baseline characteristics will be presented, broken down by treatment arm unless otherwise stated, either as n (%) or median (IQR; min-max) as applicable. Any imbalances between the arms will be assessed using χ^2 or Fishers Exact tests for categorical data and t-tests or Mann-Whitney U tests for continuous data.

- All stratification factors (see section 2.4)
- Randomisation CRF data:
  - Age at randomisation (years)
  - PSA at randomisation (ng/ml; defined as PSA pre-HT)
  - Ln (PSA) at randomisation (defined as Ln (PSA) pre-HT)
  - Time from diagnosis to randomisation (days)
  - Pain from prostate cancer at randomisation: Absent; Present
  - Broad disease category: N0M0 new; N+M0 new; M1 new; Local treatment now relapsing
  - T-stage at randomisation
  - N-stage at randomisation
  - Any metastases at randomisation
  - Bone metastases at randomisation
  - Liver metastases at randomisation
  - Lung metastases at randomisation
  - Nodal metastases at randomisation
  - Other metastases at randomisation
  - Metastatic volume (when available)
  - Use of aspirin
  - Use of NSAIDsUse of short-term bisphosphonates (subgroup: broad disease category)

- Planned type of HT (randomisation CRF; subgroup: broad disease category)
- Planned use of long-term anti-androgens
- Planned use of radiotherapy (subgroup: nodal & metastatic status)
- Cumulative randomisations (subgroup: nodal, metastatic & planned RT status)
- Participation in QL study
- Time from randomisation to starting current HT (times negative if pt starts HT pre-randomisation)
- Previous HT type: None; LHRH (agonist or antagonist if known); AAs alone; DAB
- Previous local therapy type (if known): None; radical prostatectomy; radical radiotherapy; radical prostatectomy with post-operative radical radiotherapy; other
- Duration of previous HT (days)
- Months between end of previous HT and randomisation (subgroup: broad disease category)

- Baseline CRF data:
  - Gleason sum score at presentation
  - T-stage at presentation
  - N-stage at presentation
  - M-stage at presentation
  - PSA at first presentation (ng/ml)
  - Ln(PSA) at first presentation
  - Concomitant medications (to be clinically recoded)
- Cardiovascular assessment data
  - Smoking status (subgroup: broad disease category)
  - Diabetes and type (subgroup: broad disease category)
  - History of MI, CV disease, CHF, angina or hypertension (subgroup: broad disease category)

6.5 STANDARD OF CARE TREATMENT

For all standard-of-care treatments the following data will be presented, broken down by treatment arm:

- Hormone therapy details (from the FU CRF, HT CRF or the HT Log)
  - Numbers reporting treatment action of changing or stopping (N, %)
  - Time to treatment action
  - Reason for treatment action
- Docetaxel treatment details (from the SOC Docetaxel Treatment CRF; planned details from Randomisation CRF)
  - Reported vs planned docetaxel
  - Time from randomisation to first cycle of docetaxel (days)
  - Time from starting ADT to first cycle of docetaxel (days)
  - Number of cycles administered
  - Reason for less than 6 cycles
  - Daily steroid formulation

    - Daily steroid dose
- Radiotherapy treatment details (from the RT detail CRF; planned details from the Randomisation CRF)
    - Reported vs planned use of RT
    - Timing from randomisation to first fraction of RT in:
        - All patients
        - Patients planned for RT
        - N0M0 patients
        - N+M0 patients

6.6 TRIAL TREATMENT

For **abiraterone** and **enzalutamide** research treatment data the following analyses are planned:

- Number of patients starting allocated trial treatment (N, %)
- Details of patients confirmed as not starting allocated trial treatment (as reported on the End of Research Treatment CRF)
- Time from randomisation to start of treatment (in all pts; censor those who don't report starting)
- Starting dose
- Number of treatments administered (where multiple allocated; i.e. Arm J enzalutamide + abiraterone)
- Treatment compliance/tolerance
    - Numbers reporting treatment action of changing or pausing or restarting (N, %)
    - Time to treatment action (only in pts who report starting)
    - Reason for treatment action (including associated toxicity details if relevant)
    - Length of each treatment break
    - Proportion of missed days
- Frequency and reason for dose modification and delays, treatment termination
    - Include detail of any protocol deviations in relation to overdoses.
- Permanent cessation of trial treatment (as reported on the End of Research Treatment CRF)
    - Numbers (N, %)
    - Time from randomisation to stopping (Overall & subgroup: metastatic status)
        - Censor at randomisation if explicitly reported as not starting
        - Censor at last contact if reported as starting but no report of permanent stopping
    - Time from starting treatment to stopping in patients who report starting (Overall & subgroup: metastatic status)
        - Censor at last contact if reported as starting but no report of permanent stopping
    - Reason (including associated toxicity details if relevant)

Plus

- Additional treatments given (as reported on the Additional Treatment CRF)
    - Include detail of any patients receiving non-protocol disease-directed interventions prior to study outcomes

6.7 SURVIVAL OUTCOME MEASURES

6.7.1 PRIMARY OUTCOME MEASURE

The primary outcome measure up to March 2021 was overall survival (see Table 4 for definition). From March 2021, for analyses where the M0 patients are analysed separately, the primary outcome measure for M0 patients will be metastasis-free-survival (MFS), see Table 4 for definition. This is based upon the ICECAP consortium recommendation that MFS is a suitable surrogate for overall survival in these patients. Overall survival remains the primary outcome measure for M1 patients.

The following data will be presented overall and split by metastatic status, if required:

- Incidence of death, by treatment arm
- Estimate of survival by year from randomisation, by treatment arm (%, 95% CI)
- Cause of death, by treatment arm (death form and death review process)
- Reported cause of death *vs* review cause of death
- Place of death, by treatment arm
- Death within 4 weeks of trial drug, by treatment arm
- Death related to trial treatment, by treatment arm
- Time from randomisation to death from any cause, by treatment arm
    - KM survival plot and median from FPM
    - Censor individuals at last contact if not died
- Comparisons of research vs control from Cox model
    - Adjusted for all stratification factors except centre and method of hormones; stratified by time period

6.7.2 SECONDARY OUTCOME MEASURES

For all secondary outcome measures the following analyses will be performed for the research comparison, overall and split by metastatic status, unless specified:

- Incidence of the outcome, by treatment arm
- Estimate of (*freedom from*) outcome by year from randomisation, by treatment arm (%, 95% CI)
- First reported progression event, by treatment arm [FFS only]
- Time from randomisation to outcome, by treatment arm
    - KM survival plot and median from FPM
    - Censor individuals at last contact if outcome not reported
- Comparisons of research vs control from Cox model adjusted for stratification factors (excluding centre and method of hormones); stratified by time period.

6.7.3 STATISTICAL ANALYSIS METHODS

Time-to-event data will be presented using Kaplan-Meier curves. Censoring dates will be used in all time-to-event analyses for patients who have not experienced the event in question (e.g. progression, death), as defined in Table 4. For KM plots, all patients randomised to the comparison being analysed will be included. Those patients

DocuSign Envelope ID: D18485C5-2BA5-4361-929F-A527801C0052

who have no reported event and contribute no information, such that they are censored at the date of randomisation, will be censored with a time of 0.1 days.

The HR will be analysed using a Cox proportional hazards model, adjusted for those stratification factors used at randomisation (see section 2.4) except for centre and method of hormones, and stratified by the relevant time period/strata for when the comparison was recruiting (see Table 6). This adjusted estimate will be the primary analysis estimate of treatment effect.

Differences in time-to-event outcomes will be assessed using the log-rank χ^2 test and expressed using a hazard ratio (HR) with both a one-sided 97.5% confidence interval and also a 2-sided 95% confidence interval; where the design alpha differs from 0.025 the upper limit of the corresponding one-sided confidence interval will be presented (for example, for an alpha of 0.25 this would be the upper limit of a one-sided 75% confidence interval). The number of events observed and the log-rank expected number of events will be presented.

Alternative estimation of the treatment effect will use exploratory methods e.g. log-rank hazard ratio. Flexible parametric models, modelling the difference between treatment groups over time, will be fitted to the time-to-event data with and without including time-dependent treatment effects.

6.7.4 PROPORTIONAL AND NON-PROPORTIONAL HAZARDS

The assumption of proportional-hazards (PH) will be tested on the basis of Schoenfeld residuals after fitting the Cox model. Application in Stata will be using the command `-estat phtest-` with evidence of non-PH between the two treatment groups being the main focus. Appropriate methods will be applied to the data in the case of any violation and presented alongside the primary analysis to aid interpretation; see details below.

If there is no evidence of non-PH in the treatment effect, the HR for the adjusted Cox model takes primacy.

If there is evidence of non-PH in the treatment effect, the HRs can be difficult to interpret and the restricted mean survival time (RMST) (or "restricted mean time-to-event time") difference constructed from a flexible parametric model with time-varying treatment effect will take primacy. This analysis will use a flexible parametric model, adjusted for the stratification factors used at randomisation (except for centre and method of hormones) and relevant time strata, to determine the time-dependent treatment effect and then predict values needed for subsequent RMST analysis. The application of this methodology will be particularly useful in the case where there is adequate evidence that the proportional hazard assumptions are violated at the 5% level (i.e. P<0.05). Application in Stata will be using the command `-strmst-`.

RMST will be calculated and reported for the main treatment effects regardless of whether the PH assumption is violated, as this is recognised as a useful measure. The time within which RMST will be determined by the timing of events in the control arm (using the command `-maturity_rmst-` in Stata). For this purpose, t* (the time up to when we want to compute the outcome measure of RMST) will be defined as the maximum available follow-up time (if clinically meaningful) where there is most power for the analysis, or a suitable salient time point otherwise, for the research comparison of interest at the time of the analysis.

DocuSign Envelope ID: D18485C5-2BA5-4361-929F-A527801C0052

6.7.5 Competing Risks

Analysis of those outcomes where there are considered to be competing risks will be performed by fitting an adjusted Cox model as well as a competing risks regression model, with a competing risk defined as any event the patient would likely have experienced had they not experienced the outcome of interest first. To date, outcomes with competing risks are FFS, PFS, mPFS, SRE and disease-specific survival where the competing risk is death from non-PCa cause.

6.7.7 Patients with no data post randomisation

All models used to estimate treatment effect will include those individuals with no reported outcome event and contributing no censoring information such that they are censored at their date of randomisation (t=0.1 days after randomisation).

6.7.8 Withdrawn patients with outcome data

In May-2019 it was highlighted that a small number of patients who had withdrawn consent for further data collection had outcome data (e.g. death date) recorded in the database; as a result, they were included as events rather than censorings in analyses. To avoid this issue in future analyses, the code used to produce time-to-event and event indicator variables was updated. Any patients who withdraw consent for future data collection and for whom an outcome event has not been reported up to the date of withdrawal are now included as censored on the date of withdrawal in the relevant analysis (or earlier for non-OS outcomes, see Table 4).

6.7.9 Sensitivity analysis to assess impact of COVID-19 pandemic

From March 2020, there was significant disruption to UK cancer services as a result of the COVID-19 pandemic. It is unclear whether this disruption had an impact on the ways in which patients were followed up or assessed for progressive disease. Therefore, for all analyses where it is considered possible that results could be affected by the pandemic, a sensitivity analysis will be undertaken where the administrative censoring date of 1st March 2020 is used as a truncation date. A visual (not statistical) comparison will be made between the point estimates from primary analyses that include and exclude data after this date and the numbers of events and censoring occurrences excluded will be summarised by randomised group.

6.8 PRE-SPECIFIED SUBGROUP ANALYSES

STAMPEDE recruits patients at high risk of dying from prostate cancer and who are starting long-term hormone therapy for the first time (hormone-naïve disease). This is a broad spectrum of patients including both men with metastases at randomisation and men without metastases, and men with de novo disease or disease relapsing after previous local therapy.

DocuSign Envelope ID: D18485C5-2BA5-4361-929F-A527801C0052

6.8.1 STRATIFICATION FACTORS USED FOR MINIMISATION

The stratification factors described in section 2.4 (apart from recruiting centre or hormone use) used for minimisation at the point of randomisation will form subgroups in which treatment effect will be assessed, with an interaction p-value of less than 0.1 used to suggest evidence of a potential difference in treatment effect across the relevant subgroups. As with all subgroups, we accept that there is limited power to detect an interaction and for analyses restricted to patients in a particular subgroup. The raised probability of a type 1 error from multiple testing will be acknowledged. As a result, these subgroup analyses will be regarded as exploratory.

6.8.2 OTHER PRE-SPECIFIED SUBGROUP ANALYSIS

6.8.2.1 METASTATIC VOLUME AND RISK IN M1 PATIENTS

During the trial, interest has grown in estimating the "volume" of metastases, following analyses of docetaxel in CHAARTED which divided its metastatic patients into "low volume" and "high volume" metastatic disease, and of abiraterone in LATITUDE which divided metastatic patients into "low risk" and "high risk", and only recruited the latter. Volume has been measured in A-G and is being measured in A-J using prospectively collected information on the presence of visceral metastases from the screening CRF and post-hoc review of the number of bone metastases using retrospectively collected baseline whole-body bone scintigraphy, CT and MRI scans. When available, subgroup analysis of treatment effect by volume and risk will be conducted. The following definitions will be used to classify patients into low and high volume for the purpose of this analysis:

High-volume disease: four or more bone metastases on bone scan, including one or more outside the vertebral bodies or pelvis, and/or visceral metastases

Low volume disease: Bone metastases on imaging as defined above not meeting high volume definition and no visceral metastases.

The following definition will be used to classify patients into high risk for the purpose of this analysis:

any two of the following: (1) three or more bone metastases on bone scan, (2) Gleason sum ≥8, and (3) any visceral metastases.

Given that there may be an incomplete series of bone scans collected for volume assessment, and it is possible that scans will not be missing at random, subgroup analyses that combine the G and J comparisons will only be conducted by pooling the interaction HRs using deft meta-analysis methods (7).

6.9 PSA RESPONSE

PSA response data within the first 24 weeks on trial are used to determine a patient's biochemical progression category (see Protocol for details). Of interest is whether this differs by allocated treatment arm, therefore the following data should be presented by treatment arm on an intention-to-treat basis:

DocuSign Envelope ID: D18485C5-2BA5-4361-929F-A527801C0052

- Boxplot of Log of PSA nadir (to 24 weeks after randomisation)
    - Give data underpinning this including N, min, Q, Max, mean, SD
- Biochemical progression category, N (%)

6.10 TOXICITY AND SAFETY ANALYSES

Toxicity data will be reported using the NCI Common Toxicity Criteria for Adverse Events and presented in the **ITT populations for interim analysis reports and annual review reports** to the IDMC; however breakdown of safety/toxicity data should be presented in the **safety population for final analysis publication and reports**, with only headline figures shown for the ITT population to demonstrate comparability of the populations.

Data presented by treatment arm should be:

- KM plot of time to first G3-5 toxicity reported on the FU, Toxicity or SAE CRF; include maximum SAE grade
- Worst toxicity grade in any category (Overall & subgroup: metastatic status); in each category; proportion with grade 3-5, for the following time points/periods:
    - Within 6m from randomisation (up to and including week 30)
    - At 1 year (+/- 12 weeks)
    - At 2 years (+/- 12 weeks)
    - Ever on trial
- Time to first grade 3-5 SAE
- Time to any grade SAE
- Time to first grade 3-5 SAR
- Time to any grade SAR
- Time to first grade 3-5 SUSAR
- Time to any grade SUSAR
- Table of all reported SAEs reviewed as being related to treatment (classified as a SAR or SUSAR); table to include the following details ordered by trial arm and Patient ID:
    - Patient ID
    - Date of randomisation
    - Date of onset
    - Main diagnosis and associated symptom(s)
    - Grade (as determined by clinical reviewer; site-attributed grade used if not yet reviewed)
    - Why the event is serious
    - SAE status
    - Causal relationship to HT/trial drug; related? expected?
    - Reviewed relatedness; SAR or SUSAR

DocuSign Envelope ID: D18485C5-2BA5-4361-929F-A527801C0052

Safety analyses will be performed and presented on the safety population. All safety analyses will focus on adverse events experienced during treatment and up to 30 days after the end of research treatment (see Protocol).

Safety will be evaluated by tabulation of adverse events at or up to pre-defined follow-up time points. Adverse events will be classified using the NCI Common Toxicity Criteria for Adverse Events (CTCAE v4.03 for all assessments dated from Protocol v19.0 (26-Nov-2018) onwards; assessments made before this using CTCAE v3.0 and CTCAE v4.0 will be re-coded to fit with CTCAE v4.03) and summarised for each treatment arm. Reported grading is "0 = toxicity not experienced" up to "5 = fatal".

Adverse events (AEs) may be detected through several sources reported by sites on CRFs:

1. **Follow-up CRF–** routinely reported symptoms and "toxicities" (severity not seriousness reported)
   - AEs reported here up to Sep-2016.
2. **Toxicity CRF –** prompted reporting of symptoms and "toxicities" (severity not seriousness reported)
   - AEs reported here from Sep-2016 onwards.
   - Linked to routine follow-up visits, where sites are asked to report any toxicities experienced in the period covered by the follow-up assessment; and treatment actions and permanent stopping of treatment where toxicity is given as the reason for the action.
3. **SAE CRF –** spontaneously reported serious adverse events (severity and seriousness reported)

Not all serious events are severe nor are all severe events serious.

"Seriousness" is a term specific to the reporting of events to regulatory bodies. We have prioritised the consideration of "severity" for balancing evidence of treatment side-effects against activity data. SAE forms and follow-up forms both request the severity of events. Therefore, these sources can be merged to form one dataset for reporting the **severity** of toxicities experienced across different body systems specific disease categories. The focus of severity-reporting will be on toxicities with grade 3, 4 or 5 (fatal), however all toxicity grades will be reported for completeness.

For toxicity data reported **at** pre-specified set time points from randomisation (e.g. at one year since randomisation), this will be reported only for patients who have not progressed before the set time point; this is because for patients on either the control arm or research arms where trial treatment is to be stopped at progression, toxicity data is only expected up to the time of first reported progression. For patients on research arms where trial treatment can (and should) continue beyond first progression (i.e. beyond a first FFS event), namely current Arms G, J and K, an additional report of toxicity data at specific time points from randomisation will be presented only for research arm patients still on treatment at that time; this is because toxicity data for their contemporaneous control patients would not have toxicity collected after the FFS events. Reporting windows will be defined around these set time points which will be as close to the time of interest while accepting that clinical practice means that most patients will not be reviewed on a specific day. These windows are as follows:

DocuSign Envelope ID: D18485C5-2BA5-4361-929F-A527801C0052

- Toxicity at one year on trial: based on information provided for follow-up assessment or SAE report closest to a patient's 1-year anniversary of randomisation to the trial, within 12 weeks of this anniversary. Patients are included in the relevant cross-sectional analysis if progression / permanent treatment stopping has not been reported by 48 weeks since randomisation.
- Toxicity at two years on trial: based on information provided for follow-up assessment or SAE report closest to a patient's 2-year anniversary of randomisation, within 12 weeks of this anniversary. Patients are included in the relevant cross-sectional analysis if progression / permanent treatment stopping has not been reported by 96 weeks since randomisation.

For toxicity data reported **up to** pre-specified time points from randomisation this will include all patients with follow-up/toxicity/SAE data available within that time frame.

All patients receive ADT as standard-of-care and so interest will be in the additional toxicity reported for patients on research arm relative to control arm, compared informally. Interest will also be in any proportion of known treatment toxicity above that which is expected in this population.

"Relatedness" is only collected for SAEs and cannot be reported for all adverse events.

6.11 QUALITY-OF-LIFE ANALYSIS

Quality of life data will not be analysed as part of the primary analysis, but may be analysed separately at a later date. A separate SAP will be developed for such analyses.

6.12 HEALTH ECONOMICS

Health economic data will not be analysed as part of the primary analysis, but may be analysed separately at a later date. A separate SAP will be developed for such analyses.

APPENDIX A – EXTENSION OF STATISTICAL ANALYSIS PLAN DESCRIBING THE COMBINED ANALYSES OF M0 AND M1 PATIENTS IN THE A-G AND A-J COMPARISONS

This appendix relates to a combined analysis of the patients recruited into either the Abiraterone (A-G) or Abiraterone + Enzalutamide (A-J) comparisons, separated by metastatic status. The appendix should be used in combination with the main J comparison statistical analysis plan that describes in more detail the methods for analysis of the whole comparison. The analysis for M0 patients was perfomed in August 2021 based on the requirement for the pre-defined number of events in the M0 control groups. Sections A1.2-A1.5 and the addendum are unchanged since July 2021. This appendix is being updated in March 2022 to describe the similar meta-analysis methods that will be employed to combine the M1 patients from the A-G and A-J comparisons. The timing of the M1 analysis was not dependent on pre-defined criteria but planned for after closure of the trial to collected of any further trial-related data.

Please note, in a small number of patients, the assessment of metastatic status at randomisation has changed based on subsequent testing and imaging such that the randomisation form becomes a more correct representation of the true metastatic status of the patient. For the purposes of separating M0 and M1 analyses, the data from the randomisation form (extracted from MACRO on 03-Aug-2021, rather than the randomisation server) will be used to select patients. The number of patients in whom this status changed will be described in any data report. It is acknowledged that the minimisation factors will differ slightly from the true status.

A1.1 Recruitment and progress for A-G and A-J comparisons

Recruitment into the A-G (abiraterone) comparison commenced in Nov 2011 and closed in Jan 2014 with a total of 1917 patients randomised using a 1:1 ratio. The primary results were published in 2017 with results reported by M0 (N=915) and M1 (N=1002) subgroups.

Recruitment into the A-J (abiraterone + enzalutamide) comparison commenced in July 2014 and closed in March 2016 with a total of 1976 patients randomised using a 1:1 ratio. The primary analysis was planned to occur when 267 deaths had occurred in the control arm for all patients. We have already reached this event total but rather than undertake the final analysis in all patients, we aim to modify the final statistical analysis and the rationale for this is described below.

Follow-up continued in both A-G and A-J comparisons until November 2021. Patients still receiving abiraterone and/or enzalutamide will be given access to continued drug supply, but no further outcome data is being collected on them apart from SAE reporting.

A1.2 New knowledge with implications for the final analysis of the A-J comparison

1. Abiraterone is more effective than was expected when Arm A-J was designed. The target HR for the Arm A-G comparison was 0.75 and the observed HR was 0.63, 95% confidence interval [CI], 0.52 to 0.76; P<0.001 (1). Failure free survival benefit was even greater: 0.29; 95% CI, 0.25 to 0.34; P<0.001. These results were confirmed in the independent, industry-sponsored LATITUDE trial (2). Similar benefit has been observed with enzalutamide in the EnzaMet trial, although the comparison in the latter trial was ADT with a non-steroidal anti-androgen (3), and with the anti-androgen apalutamide in the industry-sponsored Titan trial (4). Given the high efficacy of abiraterone or enzalutamide as single agents, it is considered unlikely that we will detect superiority for the combination over the single agents in an unselected population. This is especially notable in the M0 cohort where only 34/460 patients in the active arm died at primary analysis of Arm A-G (1).

2. Two of three trials in mCRPC evaluating combination CYP17A1 and AR inhibition have been reported. Both found no increase in efficacy for the combination but a slight increase in toxicity (5). Most notably, the Alliance study randomized 1311 1st line mCRPC patients 1:1 to abiraterone and enzalutamide versus

DocuSign Envelope ID: D18485C5-2BA5-4361-929F-A527801C0052

enzalutamide. This was an open label trial, and no OS benefit was found (NCT01949337, presented at ASCO 2019 by Morris et al.). The third trial is the industry-sponsored ACIS trial that randomised 983 1st line mCRPC patients to abiraterone with apalutamide versus abiraterone with placebo (NCT02257736) and is due to be reported in 2021.

Given the major differences in event rates for M0 and M1 patients we propose to split these two populations for the A-J primary analysis and combine them with the patients from the A-G comparison testing abiraterone alone. Thus, we will be testing the efficacy of androgen receptor (AR) therapy (using abiraterone alone or in combination with enzalutamide) in M1 and M0 patients separately. Further imaging and biomarker-stratified analyses are also planned to identify both prognostic and predictive biomarkers to inform de-escalation or escalation of AR therapy. Separate SAPs will be developed for these analyses.

A1.3 Proposed outcome measures for M0 patients

As for other STAMPEDE comparisons, A-G and A-J have been designed to use Failure-Free-Survival (FFS) as the intermediate outcome and Overall Survival (OS) as the final primary outcome. However, the ICECAP meta-analysis of patients with M0 disease has demonstrated that Metastasis-Free-Survival (MFS), defined as time from randomisation to distant metastases confirmed by imaging or histological evidence or death from any cause, is a robust surrogate outcome measure for OS (6). A similar meta-analysis of patients with M1 disease is currently under way (STOPCAP) but has not yet reported. Therefore, we propose to use MFS as our primary outcome measure for the M0 patients but will keep OS as our primary outcome measure for the M1 patients. A full description of these outcome measures is provided in the main A-J comparison SAP.

See Addendum 1 for a summary of the number of events in the control arm for different outcome measures in the A-G and A-J comparisons based upon data extraction in July 2019 when this SAP was first updated.

A1.4 Proposed combined analysis of A-G and A-J comparisons for M0 patients

By combining M0 patients from A-G and A-J into one meta-analysis we will have 920 M0 patients randomised between Abi and control in A-G and 1062 M0 patients randomised between Abi/Enza vs control in A-J => total meta-analysis size of 1982 patients equally randomised between AR therapy vs control over a 4.5 year recruitment period.

There was ~6 month gap in recruitment between Jan-June 2014 between close of A-G and activation of A-J. Thus, there are no shared control group issues for the M0 patients as each trial has their own set of unshared concurrent controls.

Addendum 1 summarises the number of MFS events in the control arms of the M0 patients in A-G and A-J, with a total of 135 (A-G) and 87 (A-J) = 222 MFS events having occurred by July 2019.

Addendum 2 provides the nstage software output from Stata indicating the number of events we need in the control arms of A-G and A-J to power a robust comparison in M0 patients. The following assumptions were used:

- Using 70% survival at 66 months (taken from the M0 patients in the A-G control arm curves)
- Assume a recruitment rate of 37 patients per month for 4.5 years (54 months) => 1998 patients
- Target power for primary analysis: 90%, significance level of 1.25% (one-sided) to account for one previous primary analysis of M0 patients in the A-G comparison.
- Target HR for treatment: 0.75

For a simple 2-arm trial as there are no shared M0 controls, we require 315 MFS events in the control arm. Further consideration of the number of shared events with the 2017 G analysis indicates that only one quarter of the events in the control arm have been used in that previous 2017 analysis so our one-sided significance level of 1.25% is conservative. Accounting for the actual number of shared events indicates that this can be relaxed to 2.18% and this would lead to the need for at least 277 MFS events to have occurred in M0 patients in the combined control arms of A-G and A-J. There will also be a small loss in power

associated with combining the datasets using a meta-analysis approach so boosting the power to 92% will require 299 events in the M0 control arms. Thus, we will aim for at least 300 MFS events to have occurred in the control arm before we undertake the analysis.

Timelines for reporting M0 results

We have used ARTPEP and other forecasting approaches to predict when ~300 control arm events will be reached, and this suggests that it will occur in Q2 2021 (see Addendum 3). Following data cleaning and analysis, we anticipate that this analysis of M0 patients will be able to report by Q4 2021 at the latest. It is possible we may be able to analyse earlier to coincide with presentation of results at ESMO 2021 but this will depend on the extent of data cleaning and quality by early August 2021.

A1.5 Meta-analysis methods for the combined analysis of M0 patients in A-G and A-J

An IPD meta-analysis will be undertaken to combine the A-G and A-J comparisons for M0 patients. Definition of outcomes, handling of data and analysis methods for survival outcomes will be as described in the main SAP for the primary analysis of the Abiraterone + Enzalutamide comparison (version 4.0). As for the main analysis the adjusted treatment effect estimate is regarded as the primary analysis and this pooled meta-analysis will combine the estimates adjusted for the minimisation strata.

After consultation with the MRC CTU meta-analysis group, it was agreed that a fixed effects meta-analysis would be most appropriate for the pooled estimation of treatment effects in this meta-analysis. Given the longer follow-up in the A-G comparison we anticipate a more precise estimate than that for the A-J comparison and it is possible that the A-J comparison treatment effect estimate will be larger than for A-G as it is testing a combination of two AR therapies. Both of these aspects are likely to generate some trial heterogeneity but a fixed rather than random effects meta-analysis was preferred as a) we are exploring the pooled effect of any AR therapy in these patients and b) all aspects of trial delivery have been almost identical for the two comparisons in terms of sites, protocols and eras of SoC. Forest plots will be used to present the adjusted estimates for each comparison separately alongside the pooled estimate. Any subgroup analyses or tests of interaction undertaken will be pooled using appropriate deft methods as described here (7)

A1.6 Statistical analysis for the combined analysis of M1 patients in A-G and A-J

Using the same methods as with the M0 patients, M1 patients from A-G and A-J comparisons will be combined for an IPD meta-analysis with OS as primary outcome. The A-G cohort represents the longest follow-up with trial-related data collected for ADT + 2nd generation hormone agent. A-J is the only cohort to have combined CYP3A4 inhibition with AR antagonism in mHSPC. The meta-analysis will include 1003 patients randomised between the control and "Abiraterone" arm, and 916 patients randomised between the control and "Enzalutamide and Abiraterone" arm. Any subgroup analyses or tests of interaction undertaken will be pooled using appropriate deft methods as described here (7). The primary result of interest for the community is evaluation of a difference in treatment effect (interaction HR) and between-trial heterogeneity for the A-G and A-J cohorts. These will be performed and reported as for the M0 cohorts. As this analysis indirectly compares the efficacy of adding enzalutamide to abiraterone, this is justified given a large benefit for the primary endpoint is likely to be required for a change in the current clinical practice of abiraterone with ADT to combination abiraterone and enzalutamide with ADT. Indirect comparisons with enzalutamide and ADT may also be required to change clinical practice.

REFERENCES

1. James ND, de Bono JS, Spears MR, Clarke NW, Mason MD, Dearnaley DP, et al. Abiraterone for Prostate Cancer Not Previously Treated with Hormone Therapy. N Engl J Med. 2017;377(4):338-51.

2. Fizazi K, Tran N, Fein L, Matsubara N, Rodriguez-Antolin A, Alekseev BY, et al. Abiraterone plus Prednisone in Metastatic, Castration-Sensitive Prostate Cancer. N Engl J Med. 2017;377(4):352-60.

DocuSign Envelope ID: D18485C5-2BA5-4361-929F-A527801C0052

3. Davis ID, Martin AJ, Stockler MR, Begbie S, Chi KN, Chowdhury S, et al. Enzalutamide with Standard First-Line Therapy in Metastatic Prostate Cancer. N Engl J Med. 2019;381(2):121-31.

4. Chi KN, Agarwal N, Bjartell A, Chung BH, Pereira de Santana Gomes AJ, Given R, et al. Apalutamide for Metastatic, Castration-Sensitive Prostate Cancer. N Engl J Med. 2019;381(1):13-24.

5. Attard G, Borre M, Gurney H, Loriot Y, Andresen-Daniil C, Kalleda R, et al. Abiraterone Alone or in Combination With Enzalutamide in Metastatic Castration-Resistant Prostate Cancer With Rising Prostate-Specific Antigen During Enzalutamide Treatment. J Clin Oncol. 2018;36(25):2639-46.

6. Xie W, Regan MM, Buyse M, Halabi S, Kantoff PW, Sartor O et al. Metastasis-Free Survival Is a Strong Surrogate of Overall Survival in Localized Prostate Cancer. J Clin Oncol. 2017 Sep 20;35(27):3097-3104. doi: 10.1200/JCO.2017.73.9987.

7. Fisher DJ, Carpenter JR, Morris TP, Freeman SC, Tierney JF. Meta-analytical methods to identify who benefits most from treatments: daft, deluded, or deft approach?. bmj. 2017 Mar 3;356.

DocuSign Envelope ID: D18485C5-2BA5-4361-929F-A527801C0052

ADDENDUM 1 - Numbers of events in control arms by July 2019

Abiraterone comparison (A vs G) - control event numbers

Has FFS event occured?	Mets status M0	M1	Total
No event	263	69	332
Yes event	192	433	625
Total	455	502	957

Has MFS event (ICECAP definition) occured?	Mets status M0	M1	Total
No event	320	143	463
Yes event	135	359	494
Total	455	502	957

Has patient died?	Mets status M0	M1	Total
Not died	362	195	557
Yes died	93	307	400
Total	455	502	957

Enz+Abi comparison (A vs J) - control event numbers

Has FFS event occured?	Mets status M0	M1	Total
No event	413	103	516
Yes event	120	351	471
Total	533	454	987

Has MFS event (ICECAP definition) occured?	Mets status M0	M1	Total
No event	446	188	634
Yes event	87	266	353
Total	533	454	987

Has patient died?	Mets status M0	M1	Total
Not died	479	258	737
Yes died	54	196	250
Total	533	454	987

DocuSign Envelope ID: D18485C5-2BA5-4361-929F-A527801C0052

ADDENDUM 2 - Nstage calculation for target control arm event number, based on observed MFS in combined analysis of A-G and A-J for M0 patients

```
nstage, s(0.7) t(66) tunit(4) accrue(37) alpha(0.0125) omega(0.9) hr0(1) hr1(0.75)
tstop(54) nstage(1) aratio(1) arms(2)

n-stage trial design                  version 3.0.1, 10 Sept 2014
----------------------------------------------------------------
Sample size for a 2-arm 1-stage trial with time-to-event outcome
based on Royston et al. (2011) Trials 12:81
----------------------------------------------------------------

Median survival time: 128.3 time units

Operating characteristics
------------------------------------------------------
Alpha(1S)    Power    HR|H0    HR|H1 Crit. HR Duration
------------------------------------------------------
    0.013    0.901    1.000    0.750    0.836   97.750
------------------------------------------------------
Patient accrual stopped at time 54.000

Duration is expressed in one month periods and assumes
survival times are exponentially distributed

Sample size and number of events
------------------------------------
           Overall  Control   Exper.
------------------------------------
Arms             2        1        1
Acc. rate       37       19       19
Patients      1998      999      999
Events         563      315      248
------------------------------------
```

DocuSign Envelope ID: D18485C5-2BA5-4361-929F-A527801C0052

ADDENDUM 3 - Estimation of control arm event accrual for M0 patients, assuming linear increase based on observed quarterly accrual in 2018

As of the July 2019 data extraction, there were 135+87=222 MFS events in the combined G+J control arms. To account for the lag in reporting that is typically seen between a data extraction and return of the progression CRFs from sites, we add 16 additional events to the total of 222 observed leading to a total of 238 by Q3 2019.

Event forecast assuming rate of MFS event accrual continues as per 2018 for both comparisons (5 per quarter in A-G comparison; 6 per quarter in A-J comparison; total = 11 events per quarter). We anticipate reaching 315 events in the control arm of the combined comparisons at the end of Q2 2021. Therefore, we propose to close follow-up of both A-G and A-J comparisons at the end of June 2021. We would require 3 months for data cleaning and analysis with reporting in Q4 2021. Further close out funding would be required to complete the FSR and EuRACT upload by June 2022.

	quarter	quarte~y	tot_ev~s	
1.	1	Q3 2019	238	
2.	2	Q4 2019	249	
3.	3	Q1 2020	260	
4.	4	Q2 2020	271	
5.	5	Q3 2020	282	
6.	6	Q4 2020	293	
7.	7	Q1 2021	304	
8.	8	Q2 2021	315	A-G and A-J comparisons closes follow-up
9.	9	Q3 2021	321	
10	10	Q4 2021	327	
11	11	Q1 2022	333	
12	12	Q2 2022	339	

7 SIGNATURES OF APPROVAL

Date: 25-May-2022

Version: 4.0

Signatures

Name	Trial Role	Signature	Date
Nick James	Chief Investigator*	DocuSigned by: Nick James C376081600384F4...	22-Jun-2022
Gert Attard	CCI: "Enzalutamide + abiraterone comparison"	DocuSigned by: Gert Attard 9E5B668D6847454...	21-Jun-2022
Louise Brown	Project Lead	DocuSigned by: Louise Brown B826E526896F494...	16-Jun-2022
Chris Brawley	Trial statistician	DocuSigned by: Chris Brawley 5CD2873052DC4D3...	22-Jun-2022
Laura Murphy	Trial statistician	DocuSigned by: Laura Murphy 1F26EDC94B1A4A1...	17-Jun-2022
Max Parmar	CTU Director and Programme Lead	DocuSigned by: Max Parmar 47F3FE2126EF4EE...	23-Jun-2022

*On behalf of the STAMPEDE Trial Management Group

Credits: Other statisticians involved in development of this SAP have been Rachael Jinks, Gordana Jovic and Melissa Spears

Reviewers: Ian White (Apr-2018), Babak Oskooei-Choodari (Apr-2018)

STAMPEDE OVERSIGHT COMMITTEES, STAFF AND COLLABORATORS

Version: 29-Mar-2023

CONTENTS

NOTES.. 2
TRIAL MANAGEMENT GROUP.. 3
INDEPENDENT DATA MONITORING COMMITTEE 4
TRIAL STEERING COMMITTEE 5
MRC CLINICAL TRIALS UNIT AT UCL STAFF... 6
SWISS GROUP FOR CANCER CLINICAL RESARCH (SAKK) STAFF 11
BIOLOGY AND IMAGING SUBGROUPS 12
INVESTIGATORS AND COLLABORATORS: SITE STAFF............................... **Error! Bookmark not defined.**
INDUSTRY COLLABORATORS.. 152
PARTICIPANTS 153

NOTES

Investigators and site staff are those who have formally appeared at any time on a site's Delegation Logs.

CTU staff are those who have worked on or contributed to the trial any time from the outset until the date of this report.

The independent members of the Independent Data Monitoring Committee and Trial Oversight Committee play an important role in the conduct of the trial.

Industry collaborators are a subset of the people who have worked, on relevant sections, with the trial staff to ensure the trial runs efficiently.

TRIAL MANAGEMENT GROUP

(Listing only member external to the trials unit or senior staff at the trials unit)

Area	Status	Member	Geography	Role
Clinical/Surgical	Current	Gerhardt Attard	London (UCL), UK	CCI
~	~	Simon Chowdhury	London (Guys), UK	
~	~	Noel Clarke	Manchester, UK	Deputy CI, Co-CCI
~	~	William Cross	Leeds, UK	
~	~	David Dearnaley	Sutton, UK	
~	~	Silke Gillessen	Lugano, Switzerland[1]	CCI
~	~	Nicholas James	London (ICR), UK[2]	CI
~	~	Rob Jones	Glasgow, UK	
~	~	Zafar Malik	Wirral, UK	
~	~	Chris Parker	Sutton, UK	CCI
~	~	J Martin Russell	Glasgow, BOC	
~	Previous	Daniel Aebersold	Berne, Switzerland	
~	~	John Anderson	Sheffield, UK	
~	~	Johann de Bono	Sutton, UK	
~	~	Malcolm Mason	Cardiff, UK	
~	~	John Masters	London, UK	
~	~	Rick Popert	London (Guys), UK	
~	~	Alastair Ritchie	Gloucester, UK	
~	~	George Thalmann	Berne, Switzerland	
PPI	Current	David Matheson	Other	
~	~	Robin Millman	~	
~	Previous	John Dwyer	~	
~	~	David Hoe-Richardson	~	
~	~	Jim Stansfeld	~	
Senior CTU	Current	Claire Amos	MRC CTU at UCL	
~	~	Louise Brown	~	
~	~	Adrian Cook	~	
~	~	Duncan Gilbert	~	
~	~	Ruth Langley	~	CCI
~	~	Mahesh Parmar	~	Programme Lead[3]
~	~	Matthew Sydes	~	
~	Previous	Nafisah Atako	~	
~	~	Cheryl Pugh	~	
Clinical Fellow	Current	Hoda Abdely-Aty	MRC CTU at UCL	
~	~	Hannah Rush	~	
~	Previous	Clare Gilson	~	
~	~	Archie MacNair	~	

Key: CI = Chief Investigator
CCI = Comparison CI
CoCCI = Comparison Co-CI

Note: The full list of MRC CTU at UCL staff is detailed below in a subsequent section.

[1] Previously Manchester, UK & St Gallen, Switzerland
[2] Previously Birmingham, UK & Warwick, UK
[3] Also CTU Director

INDEPENDENT DATA MONITORING COMMITTEE
(All members independent)

Member	Status	Role
Richard Emsley	Current	
Bertrand Tombal	~	Chair 3
Ronald de Wit	~	
Chris Williams	Previous	Chair 1
John Yarnold	~	Chair 2
Doug Altman	~	
Reg Hall	~	

TRIAL STEERING COMMITTEE
(Listing only independent members)

Member	Status	Role
Paula Ghaneh	Current	Chair 3
Tim Clayton	~	
Jan-Erik Dember	~	
Paul Nash	~	PPI rep
James Larkin	~	
Jonathan Ledermann	Previous	Chair 1
Richard Emsley	~	
John Fitzpatrick	~	
Alan Horwich	~	
David Kirk	~	
Jim Paul	~	

MRC CLINICAL TRIALS UNIT AT UCL STAFF

Area	Status	Name
Statisticians	Current	Louise Brown
~	~	Adrian Cook
~	~	Peter Dutey-Magni
~	~	Laura Murphy
~	~	Matthew Nankivell
~	~	Mahesh Parmar
~	~	Matthew Sydes
~	~	Yathushan Yogeswaran
~	Previous	Sophie Barthel
~	~	Daniel Bratton
~	~	Christopher Brawley
~	~	Babak Choodari-Oskooei
~	~	Trinh Duong
~	~	Andrew Embleton
~	~	Melissa Gannon (nee Spears)
~	~	Fiona Ingleby
~	~	Elizabeth James
~	~	Rachel Jinks (nee Morgan)
~	~	Gordana Jovic
~	~	Patrick Royston
~	~	Melissa Spears
Project and Trial Management	Current	
~	~	Claire Amos
~	~	Charlene Carvalho
~	~	Mazna Anjun
~	~	Anna Griffiths
~	~	Fleur Hudson
~	~	Panos Maniatis
	~	Connor McAlpine
~	~	Claire Murphy
~	~	Horeja Njai
	Previous	Shabinah Ali
	~	Sofeya Ishqa
~	~	Dipa Noor
~	~	Malissa Richmond
~	~	Karen Sanders
~	~	Lily Clarke
~	~	Michelle Buckner
~	~	Nafisah Atako
~	~	Alanna Brown
~	~	Joanna Calvert
~	~	Rahela Choudhury
~	~	Zoe Cotton
~	~	Adam Cursley
~	~	Tom Fairfield
~	~	Silvia Forcat
~	~	Michelle Gabriel
~	~	Charlene Green
~	~	Adam Gregory
~	~	Anna Herasimtschuk

MRC CLINICAL TRIALS UNIT AT UCL STAFF

Area	Status	Name
~	~	Caroline Hogan
~	~	Brooke Jackson
~	~	Sarah Jackson
~	~	Neil Kelk
~	~	James Latham
~	~	Dymphna Lee
~	~	Sarah Miller
~	~	Sharon Naylor
~	~	Dipa Noor
~	~	Jacqui Nuttall
~	~	Jenny Petrie
	~	Cheryl Pugh
~	~	Orla Prendiville
~	~	Cheryl Pugh
~	~	Karen Sanders
~	~	Francesca Schiavone
~	~	Clare Shakeshaft
~	~	Aminata Sy
~	~	Charlotte Tyson
~	~	Hannah Vaughan
~	~	Christopher Wanstall
~	~	Katie Ward
~	~	Melanie Weiss
~	~	Arlen Wilcox
Clinicians	Current	Hoda Abdel-Aty
~	~	Duncan Gilbert
~	~	Ruth Langley
~	~	Hannah Rush
~	Previous	Clare Gilson
~	~	Archie Macnair
~	~	Sarah Meredith
~	~	Alastair Ritchie
Data Scientists and Programmers	Current	Christina Chung
~	~	Carlos Diaz-Montana
~	~	Georgia Marley
~	~	Lindsey Masters
~	~	Mary Rauchenberger
~	~	Stephen Townsend
~	~	Nadine Van-Looy
~	Previous	Carly Au
~	~	Will Cragg
~	~	Dominic Hague
~	~	Zaheer Islam
~	~	Sajad Khan
~	~	Dominic Mounsey
~	~	Nancy Tappenden
~	~	Nadine Van-Looy
Data Management	Current	Shaan Akbar
~	~	Yumna Ali

MRC CLINICAL TRIALS UNIT AT UCL STAFF

Area	Status	Name
~	~	Ify Ejizu-Allen
~	~	Hanna Barghouty
~	~	Sophia Bradshaw
~	~	Daneil Clarke
~	~	Haresh Dadlani
~	~	Donna Dobson
~	~	Lawrence Evans
~	~	Isabella Jaques
~	~	Alexander Lawton
~	~	Simona Wade
~	Previous	Margaret Hook
		Nazia Parkar
		Eva Ades
~	~	Carly Au
~	~	Katherine Beaney
~	~	Nargis Begum
~	~	Katharine Bellenger
~	~	Lina Bergstrom
~	~	Veronica Birzu
~	~	Robin Carpenter
~	~	Elizabeth Clark
~	~	Emma Donoghue
~	~	Amy Fiddament
~	~	Shree Gajjar
~	~	Hannah Gardner
~	~	Jenna Grabey
~	~	Richard Gracie
~	~	Charlene Green
~	~	Adam Gregory
~	~	Dominic Hague
~	~	Shama Hassan
~	~	Jordan Hedges
~	~	Robyn Henry-Cockles
~	~	William Hudson
~	~	Sofeya Ishaq
~	~	Danielle Johnson
~	~	Saba Khan
~	~	Zohrah Khan
~	~	Linda Ly
~	~	Adele Mabley
~	~	Georgia Mannion-Krase
~	~	Jacque Millett
~	~	Myfanwy Nicholas
~	~	Rachel Ogunleye
~	~	Meghna Pandya
~	~	Reena Patel
~	~	Sara Peres
~	~	Tasmin Philips
~	~	Philip Pollock
~	~	Chathurika Rajapakse

MRC CLINICAL TRIALS UNIT AT UCL STAFF

Area	Status	Name
~	~	Tim Smith
~	~	Hannah Sweeney
~	~	Arpita Upadhyaya
~	~	Laura Van Dyck
~	~	Hannah Vaughan
~	~	Peter Vaughan
~	~	Katie Ward
~	~	Steph Wetton
~	~	Andrew Whitney
~	~	Selin Yurdakul
Other Operations	Current	Fleur Hudson
~	~	Nicola Joffe
~	~	Macey Murray
Trial Assistants	Current	Tracey Fisher
~	~	Olivia Mahoro
~	~	Lynda Micklewright
~	Previous	Elizabeth Adesanya
~	~	Yumna Ali
~	~	Atma Amin
~	~	Hannah Babiker
~	~	Bryony Bathie
~	~	Helen Chapman
~	~	Georgia Cowley
~	~	Leigh Dobson
~	~	James Dunn
~	~	Robbie Dunn
~	~	Amy Fiddament
~	~	Ben Forson
~	~	Adam Gregory
~	~	Nasir Jamil
~	~	Tasheeka Jeyapalan
~	~	Sherwen Kang
~	~	Harry Kitson
~	~	Rebecca Lo
~	~	Joseph Martin
~	~	Nour Merzouki
~	~	Lynda Micklewright
~	~	Meghna Pandya
~	~	Ray Phillips
~	~	Jamie Simmons
~	~	Shanaz Sohail
~	~	Jeevan Sohal
~	~	Crystallynn The
~	~	Nat Thorogood
~	~	Stephanie Tsenti
~	~	Alexandra Wadia
~	~	Stephanie Wetton
STOPCAP Meta-Analysis team	Current	Sarah Burdett
~	~	David Fisher

MRC CLINICAL TRIALS UNIT AT UCL STAFF

Area	Status	Name
~	~	Peter Godolphin
~	~	Larysa Rydzewska
~	~	Jayne Tierney
~	~	Claire Vale
Administration support	Current	Sarah Banbury
~	~	Beth Jarman
~	Previous	Lesley Brempong
~	~	Emmanuel Harding
~	~	Gillian Hurst
~	~	Nishat Tasnim
~	~	Jemima Thompson

SWISS GROUP FOR CANCER CLINICAL RESARCH (SAKK) STAFF

Area	Status	Name
SAKK operations	~	Estelle Cassolly
~	~	Pierre Fustier
~	~	Eloïse Kremer
~	~	Corinne Schar

BIOLOGY AND IMAGING SUBGROUPS
(Members of translational subgroups or work packages; TMG members not repeated here)

Person	Status	Geography
Adnan Ali	Previous	Manchester, UK
Radhi Anand	Previous	London (UCL), UK
Hassan Douis	Previous	Birmingham, UK
Dan Berney	Current	London (Barts), UK
Ros Eeles	~	London (ICR), UK
Stephenie Friedrich	~	London (UCL), UK
Emily Grist	~	London (UCL), UK
Anis A Hamid	~	Melbourne, Aus & Boston (DFCI), USA
Aine Haran	~	Manchester, UK
A M Mahedi Hassan	~	London (UCL), UK
Alex Hoyle	~	Manchester, UK
Sakunthala Kudahetti	~	London (Barts), UK
Sharanpreet Lall	~	London (UCL), UK
Gianmarco Leone	~	London (UCL), UK
Hing Leung	~	Glasgow, BOC
Stefano Lise	~	London (UCL), UK
Larissa Mendes	~	London (UCL), UK
Karolina Nowakowska-Pawelkowicz	~	London (UCL), UK
Charles Parker	~	London (UCL), UK
Marina Parry	~	London (UCL), UK
Alison Parry-Jones	~	Cardiff, UK
Chris Sweeney	~	Boston (DFCI), USA, Adelaide Australia
Suparna Thakali	~	London (UCL), UK
Nina Tinariu	~	London (ICR), UK
Maria Vico	~	London (UCL), UK
Sara Santos Vidal	~	London (Barts), UK
Daniel Wetterskog	~	London (UCL), UK
Anna Wingate	~	London (UCL), UK
Carla Bautista	Previous	London (UCL), UK
Paolo Cremaschi	~	London (UCL), UK
Thomas Hambrock	~	Manchester, UK
Alex Landless	~	London (UCL), UK
Nik Matthews	~	London (ICR), UK
Mariana Buongermino Pereira	~	London (UCL), UK
Kamila Sychowska	~	London (UCL), UK
David Waugh	Previous	Belfast, UK
Leila Zakka	~	London (UCL), UK

INVESTIGATORS AND COLLABORATORS: SITE STAFF
Staff on site delegation logs

City	Care site	Name	Site PI
Abergavenny	Nevill Hall Hospital	Christian Smith	
Aberystwyth	Bronglais General Hospital	Christine Kotonya	
Aberystwyth	Bronglais General Hospital	Elin Jones	PI
Aberystwyth	Bronglais General Hospital	Helen Tench	
Aberystwyth	Bronglais General Hospital	Mark Narain	
Aberystwyth	Bronglais General Hospital	Philip Jones	
Aberystwyth	Bronglais General Hospital	Russel Canavan	
Aberystwyth	Bronglais General Hospital	Sajid Durrani	
Aberystwyth	Bronglais General Hospital	Emma Nurse	Pharmacist
Aberystwyth	Bronglais General Hospital	Gwenan Parry Jones	
Aberystwyth	Bronglais General Hospital	Kirsty Marie Dennett	
Aberystwyth	Bronglais General Hospital	Rhian Elin Jones	
Aberystwyth	Bronglais General Hospital	Sandra Griffiths nee Evens	
Aberystwyth	Bronglais General Hospital	Cerith Morgan	
Aberystwyth	Bronglais General Hospital	Kenneth Richard Williams	
Aberystwyth	Bronglais General Hospital	Llinos Strange	Pharmacist
Aberystwyth	Bronglais General Hospital	Sean Thomas	
Aberystwyth	Bronglais General Hospital	Toby Frederick Trugeion-Smith	
Aberystwyth	Bronglais General Hospital	Basharat Jameel	
Aberystwyth	Bronglais General Hospital	Bleddyn Edwards	
Aberystwyth	Bronglais General Hospital	Geraint Morgan	Pharmacist
Aberystwyth	Bronglais General Hospital	John Edwards	
Aberystwyth	Bronglais General Hospital	Donna Robson	
Aberystwyth	Bronglais General Hospital	Heather McGuinness	
Aberystwyth	Bronglais General Hospital	Ronda Loosley	
Aberystwyth	Bronglais General Hospital	Claire Duggan	
Aberystwyth	Bronglais General Hospital	Sarah Jones	

Aberystwyth	Bronglais General Hospital	Abigail Hynes	
Aberystwyth	Bronglais General Hospital	Rebecca Wolf-Roberts	
Ashford	William Harvey Hospital	Albert Edwards	Co-I
Ashford	William Harvey Hospital	Charlotte Mott	
Ashford	William Harvey Hospital	Ifigenia Vasiliadou	
Ashford	William Harvey Hospital	Lavarniya Rajakumar	
Ashford	William Harvey Hospital	Mathini Sridharan	
Ashford	William Harvey Hospital	Patryk Brulinski	
Ashford	William Harvey Hospital	Rakesh Raman	Co-I
Ashford	William Harvey Hospital	Rohit Malde	
Ashford	William Harvey Hospital	Stephane Tankoua	
Ashford	William Harvey Hospital	Arafat Mizra	
Ashford	William Harvey Hospital	Carys Thomas	PI
Ashford	William Harvey Hospital	Clary Evans	
Ashford	William Harvey Hospital	Kannon Nathan	
Ashford	William Harvey Hospital	Kathryn Lees	
Ashford	William Harvey Hospital	Mathilda Cominos	
Ashford	William Harvey Hospital	Matthew Fenton	
Ashford	William Harvey Hospital	Mohammed Osman	
Ashford	William Harvey Hospital	Natasha Mithal	Co-I
Ashford	William Harvey Hospital	Sharon Beesley	
Ashford	William Harvey Hospital	Sugeeta Sukumar	
Ashford	William Harvey Hospital	Udaiveer Panwar	
Ashford	William Harvey Hospital	Coral Greenstreet	
Ashford	William Harvey Hospital	Hayley Blackgrove	
Ashford	William Harvey Hospital	Katy Taylor	
Ashford	William Harvey Hospital	Victoria Williamson	
Ashford	William Harvey Hospital	Natalie Catt	
Ashford	William Harvey Hospital	Arafat Mirza	
Ashford	William Harvey Hospital	Sam Gibson	
Ashford	William Harvey Hospital	Steve Dann	
Ashford	William Harvey Hospital	Andrew Gillian	Pharmacist
Ashford	William Harvey Hospital	Miguel Capo-Mir	Pharmacist
Ashford	William Harvey Hospital	Cindy Slater	

Ashford	William Harvey Hospital	Hasmath Marjolin	
Ashford	William Harvey Hospital	Nikki Crisp	
Ashford	William Harvey Hospital	Rachel Larkins	
Ashford	William Harvey Hospital	Sandra Holness	
Ashford	William Harvey Hospital	Sarah Lines	
Ashford	William Harvey Hospital	Susan Rogers	
Ashford	William Harvey Hospital	Tessa Hammond	Pharmacist
Ashford	William Harvey Hospital	Claire White	
Ashford	William Harvey Hospital	Julie Buckley	
Ashford	William Harvey Hospital	Laura Kehoe	
Ashford	William Harvey Hospital	Lesley Rose	
Ashford	William Harvey Hospital	Louise Gladwell	
Ashford	William Harvey Hospital	Sarah Lightfoot	Pharmacist
Ashford	William Harvey Hospital	Tracy Boakes	
Ashford	William Harvey Hospital	Alba Tubau	
Ashford	William Harvey Hospital	Bonny Appleby	
Ashford	William Harvey Hospital	Linda Wray	Pharmacist
Ashford	William Harvey Hospital	Louise Allen	
Ashford	William Harvey Hospital	Marian Wood	
Ashford	William Harvey Hospital	Adedolapo Sanni	
Ashford	William Harvey Hospital	Claire Pelham	
Ashford	William Harvey Hospital	Elizabeth Williamson	
Ashford	William Harvey Hospital	Hilary Zurakovsky	
Ashford	William Harvey Hospital	Jill Baker	
Ashford	William Harvey Hospital	Joanne Williams	
Ashford	William Harvey Hospital	Julie-Ann Davies	
Ashford	William Harvey Hospital	Karen Robinson	
Ashford	William Harvey Hospital	Kathleen (Kathy) Walsh	
Ashford	William Harvey Hospital	Kim Mears	
Ashford	William Harvey Hospital	Kim Travis	
Ashford	William Harvey Hospital	Margaret Lipsham	
Ashford	William Harvey Hospital	Paula Whichelo	
Ashford	William Harvey Hospital	Sharon Middleton	
Ashford	William Harvey Hospital	Sue Kelly	

Ashford	William Harvey Hospital	Susan Drakeley	
Ashford	William Harvey Hospital	Sydnie Loveland	
Ashford	William Harvey Hospital	Molua Young	
Ashford	William Harvey Hospital	Denise Crawford	
Aylesbury	Stoke Mandeville Hospital	Ami Sabharwal	
Aylesbury	Stoke Mandeville Hospital	Andy Theobold	
Aylesbury	Stoke Mandeville Hospital	Janice Carpenter	
Aylesbury	Stoke Mandeville Hospital	Katherine Hyde	PI
Aylesbury	Stoke Mandeville Hospital	Thinn Pwint	Co-I
Aylesbury	Stoke Mandeville Hospital	Christopher Alcock	
Aylesbury	Stoke Mandeville Hospital	Gerard Andrade	
Aylesbury	Stoke Mandeville Hospital	Joanne Brady	
Aylesbury	Stoke Mandeville Hospital	Niki Panakis	
Aylesbury	Stoke Mandeville Hospital	Philip Camilleri	Co-I
Aylesbury	Stoke Mandeville Hospital	Prabir Chakraborti	
Aylesbury	Stoke Mandeville Hospital	Sean O'Cathail	
Aylesbury	Stoke Mandeville Hospital	Jonathan Greenland	
Aylesbury	Stoke Mandeville Hospital	Moncy Mathew	
Aylesbury	Stoke Mandeville Hospital	Rahul Kurup	
Aylesbury	Stoke Mandeville Hospital	Neil Trew-Smith	
Aylesbury	Stoke Mandeville Hospital	Alice Ngumo	
Aylesbury	Stoke Mandeville Hospital	Gail Varley	
Aylesbury	Stoke Mandeville Hospital	Janet Weir	
Aylesbury	Stoke Mandeville Hospital	Manisha Joshi	
Aylesbury	Stoke Mandeville Hospital	Siobhan Gettings	
Aylesbury	Stoke Mandeville Hospital	Cheryl Padilla-Harris	
Aylesbury	Stoke Mandeville Hospital	Maggie Aldersley	
Aylesbury	Stoke Mandeville Hospital	Margaret Bowerbank	
Aylesbury	Stoke Mandeville Hospital	Michelle Taylor-Siddons	Pharmacist
Aylesbury	Stoke Mandeville Hospital	Tracey Stammers	
Aylesbury	Stoke Mandeville Hospital	Anna Osadcow	
Aylesbury	Stoke Mandeville Hospital	Kathryn Herbert	
Aylesbury	Stoke Mandeville Hospital	Rossana Mancinelli	
Aylesbury	Stoke Mandeville Hospital	Sarah Manyangadze	

Aylesbury	Stoke Mandeville Hospital	Christine Collins	
Aylesbury	Stoke Mandeville Hospital	Hazel Wynn	
Aylesbury	Stoke Mandeville Hospital	Iram Husain	
Aylesbury	Stoke Mandeville Hospital	Jasvinder Bains	
Aylesbury	Stoke Mandeville Hospital	Roisin Kavanagh	
Aylesbury	Stoke Mandeville Hospital	Andrew Protheroe	
Ayr	Ayr Hospital	Aisha Tufail	
Ayr	Ayr Hospital	David McIntosh	
Ayr	Ayr Hospital	Helena Belikova	
Ayr	Ayr Hospital	Jawaher Ansari	
Ayr	Ayr Hospital	Rebecca Muirhead	
Ayr	Ayr Hospital	Xia Ren	Co-I
Ayr	Ayr Hospital	Christina Lai	
Ayr	Ayr Hospital	Esfandiyar Khan	
Ayr	Ayr Hospital	Hilary Glen	PI
Ayr	Ayr Hospital	Lye Mun Tho	
Ayr	Ayr Hospital	Nicholas Macleod	
Ayr	Ayr Hospital	Rana Mahmood	
Ayr	Ayr Hospital	Stefan Nowich	
Ayr	Ayr Hospital	Kirsty O'Hara	
Ayr	Ayr Hospital	Kristy Ross	
Ayr	Ayr Hospital	Brian McGlynn	
Ayr	Ayr Hospital	Mark Wilson	
Ayr	Ayr Hospital	Philip Cannon	
Ayr	Ayr Hospital	Kathleen Smith	
Ayr	Ayr Hospital	Deborah Dunn	Pharmacist
Ayr	Ayr Hospital	Jane McClements	
Ayr	Ayr Hospital	Susan Walton	Pharmacist
Ayr	Ayr Hospital	Alison Murphy	Pharmacist
Ayr	Ayr Hospital	Danielle Gilmour	
Ayr	Ayr Hospital	Elaine Watson	
Ayr	Ayr Hospital	Lillian White	
Ayr	Ayr Hospital	Lynne McNeil	Pharmacist
Ayr	Ayr Hospital	Margaret McKernan	

Ayr	Ayr Hospital	Sharon Meehan	
Ayr	Ayr Hospital	Clare Love	
Ayr	Ayr Hospital	Claudia Coubrough (Turley)	
Ayr	Ayr Hospital	Danna Yorston	Pharmacist
Ayr	Ayr Hospital	Diane Woodburn	Pharmacist
Ayr	Ayr Hospital	Dianne Hunter	
Ayr	Ayr Hospital	Jenna Mitchell	
Ayr	Ayr Hospital	Kirsten Laws (nee Borthwick)	
Barnet	Barnet General Hospital	Ami Mehta	
Barnet	Barnet General Hospital	Andrew Eichholz	
Barnet	Barnet General Hospital	Anita Mitra	
Barnet	Barnet General Hospital	Annette Hawkins	
Barnet	Barnet General Hospital	Emily Scott	
Barnet	Barnet General Hospital	Gehan Soosaipillai	
Barnet	Barnet General Hospital	Gillian Marks	
Barnet	Barnet General Hospital	Kate Smith	Co-I
Barnet	Barnet General Hospital	Kimberley Durno	Co-I
Barnet	Barnet General Hospital	Magdalena Kubiak	Co-I
Barnet	Barnet General Hospital	Sarah Needleman	PI
Barnet	Barnet General Hospital	Ursula McGovern	
Barnet	Barnet General Hospital	Danielle Collier	
Barnet	Barnet General Hospital	Andie David	
Barnet	Barnet General Hospital	Panayiotis Panayiotou	
Barnet	Barnet General Hospital	Heather Hughes	
Barnet	Barnet General Hospital	Prital Patel	
Barnet	Barnet General Hospital	Sandra Faustino	
Barnet	Barnet General Hospital	Anita Amadi	
Barnet	Barnet General Hospital	Alice Coady	
Barnet	Barnet General Hospital	Christine Ellis	Pharmacist
Barnet	Barnet General Hospital	Veronica Conteh	
Barnstable	North Devon District Hospital	Becky Holbrook	
Barnstaple	North Devon District Hospital	Ajaz Lone	
Barnstaple	North Devon District Hospital	Chantal Oelofse	
Barnstaple	North Devon District Hospital	Elizabeth Kershaw	

Barnstaple	North Devon District Hospital	Faisal Hussain	
Barnstaple	North Devon District Hospital	Mohini Varughese	PI
Barnstaple	North Devon District Hospital	Peter Stephens	Co-I
Barnstaple	North Devon District Hospital	Philippa Smith	
Barnstaple	North Devon District Hospital	Sarah Park	
Barnstaple	North Devon District Hospital	Victoria Ford	Co-I
Barnstaple	North Devon District Hospital	Andy Bull	
Barnstaple	North Devon District Hospital	Denise Sheehan	
Barnstaple	North Devon District Hospital	Elizabeth Toy	
Barnstaple	North Devon District Hospital	Maria Martinez	
Barnstaple	North Devon District Hospital	Amy Thomas	
Barnstaple	North Devon District Hospital	Chloe Peters	
Barnstaple	North Devon District Hospital	Hannah Ong	
Barnstaple	North Devon District Hospital	Jenna Furse	
Barnstaple	North Devon District Hospital	Nyasha Manomano	Pharmacist
Barnstaple	North Devon District Hospital	Jeffrey man sum Chan	
Barnstaple	North Devon District Hospital	Joshua Gregory	Pharmacist
Barnstaple	North Devon District Hospital	Michal Ian Lamparski	Pharmacist
Barnstaple	North Devon District Hospital	Thomas Baddick	Pharmacist
Barnstaple	North Devon District Hospital	Eng Ong	
Barnstaple	North Devon District Hospital	Henry Goss	Pharmacist
Barnstaple	North Devon District Hospital	Martin Moody	
Barnstaple	North Devon District Hospital	Rufus Smith	
Barnstaple	North Devon District Hospital	Ashley Hanson	
Barnstaple	North Devon District Hospital	Katherine Horder	
Barnstaple	North Devon District Hospital	Maria Beaumont	Pharmacist
Barnstaple	North Devon District Hospital	Rebecca Davey	Pharmacist
Barnstaple	North Devon District Hospital	Fiona Thomas	
Barnstaple	North Devon District Hospital	Helen Black	
Barnstaple	North Devon District Hospital	Laura Hanson	
Barnstaple	North Devon District Hospital	Lynne Van-Koutrik	
Barnstaple	North Devon District Hospital	Lynsey Balmbra-Jenks	
Barnstaple	North Devon District Hospital	Natalie Kemp	Pharmacist
Barnstaple	North Devon District Hospital	Samantha Ley	

Barnstaple	North Devon District Hospital	Susan Collard	
Barnstaple	North Devon District Hospital	Alicia Leal-Santos	Pharmacist
Barnstaple	North Devon District Hospital	Faye Windsor	
Barnstaple	North Devon District Hospital	Judyta Lomza	Pharmacist
Barnstaple	North Devon District Hospital	Lynne Van Koutrik	
Basel	Universitätsspital Basel	M Timmermann	
Basel	Universitätsspital Basel	Cyrill Rentsch	PI
Basel	Universitätsspital Basel	Frank Stenner-Liewen	Co-I
Basel	Universitätsspital Basel	Nicole Ebinger	
Basel	Universitätsspital Basel	Stephen Wyler	
Basel	Universitätsspital Basel	N Ott	
Basel	Universitätsspital Basel	Cyrill Rentsch	
Basel	Universitätsspital Basel	Eloise Kremer	
Basel	Universitätsspital Basel	Heike Puschel	
Basel	Universitätsspital Basel	Heike Puschel	
Basel	Universitätsspital Basel	Mana Farsad	
Basel	Universitätsspital Basel	Nicole Neumann	
Basel	Universitätsspital Basel	Simone Marini	
Basel	Universitätsspital Basel	Bettina Seifest	
Basel	Universitätsspital Basel	Kristina Muller	
Basel	Universitätsspital Basel	Alexander Bachmann	
Basel	Universitätsspital Basel	Christoph Rochlitz	
Basingstoke	Basingstoke and North Hampshire Hospital	Dileep Soory	
Basingstoke	Basingstoke and North Hampshire Hospital	Hilawati Yusof	
Basingstoke	Basingstoke and North Hampshire Hospital	Ingrid White	
Basingstoke	Basingstoke and North Hampshire Hospital	Rosalyne Westley	
Basingstoke	Basingstoke and North Hampshire Hospital	Sangeeta Paisey	PI
Basingstoke	Basingstoke and North Hampshire Hospital	David Barlow	
Basingstoke	Basingstoke and North Hampshire Hospital	Eva Letalova	
Basingstoke	Basingstoke and North Hampshire Hospital	Jenny Nobes	
Basingstoke	Basingstoke and North Hampshire Hospital	Joanna Stokoe	
Basingstoke	Basingstoke and North Hampshire Hospital	Katharine Webb	
Basingstoke	Basingstoke and North Hampshire Hospital	Katherine Aitken	
Basingstoke	Basingstoke and North Hampshire Hospital	Katie Wood	

Basingstoke	Basingstoke and North Hampshire Hospital	Katie Wood	
Basingstoke	Basingstoke and North Hampshire Hospital	Rao Vuyyuru	
Basingstoke	Basingstoke and North Hampshire Hospital	Richard Shaffer	PI
Basingstoke	Basingstoke and North Hampshire Hospital	Sree Susaria	
Basingstoke	Basingstoke and North Hampshire Hospital	Teresa Guerrero-Urbano	
Basingstoke	Basingstoke and North Hampshire Hospital	Jo-Anna Conyngham	
Basingstoke	Basingstoke and North Hampshire Hospital	Jo-Anna Conyngham	
Basingstoke	Basingstoke and North Hampshire Hospital	Lauriane Kerwood	Pharmacist
Basingstoke	Basingstoke and North Hampshire Hospital	Lorraine Poole	
Basingstoke	Basingstoke and North Hampshire Hospital	Louise Beattie	
Basingstoke	Basingstoke and North Hampshire Hospital	Rebecca Wills	Pharmacist
Basingstoke	Basingstoke and North Hampshire Hospital	Zoe Finlay	
Basingstoke	Basingstoke and North Hampshire Hospital	Rachel Bryan	
Basingstoke	Basingstoke and North Hampshire Hospital	Duncan Cooke	
Basingstoke	Basingstoke and North Hampshire Hospital	Godfrey Bownie-Mukumbu	
Basingstoke	Basingstoke and North Hampshire Hospital	Roger Hudson	Pharmacist
Basingstoke	Basingstoke and North Hampshire Hospital	Bintha Paruthickal	
Basingstoke	Basingstoke and North Hampshire Hospital	Catherine McLean	
Basingstoke	Basingstoke and North Hampshire Hospital	Jackie Smith	
Basingstoke	Basingstoke and North Hampshire Hospital	Nanda Basker	
Basingstoke	Basingstoke and North Hampshire Hospital	Pennie Porter	
Basingstoke	Basingstoke and North Hampshire Hospital	Abigail Edwards	
Basingstoke	Basingstoke and North Hampshire Hospital	Adrienn Fazekasne Fulep	
Basingstoke	Basingstoke and North Hampshire Hospital	Angela Frith	Pharmacist
Basingstoke	Basingstoke and North Hampshire Hospital	Christine Podesta	
Basingstoke	Basingstoke and North Hampshire Hospital	Fasar Sarwar	
Basingstoke	Basingstoke and North Hampshire Hospital	Helen Richards	
Basingstoke	Basingstoke and North Hampshire Hospital	Victoria Corner	
Basingstoke	Basingstoke and North Hampshire Hospital	Carmen Wu	Pharmacist
Basingstoke	Basingstoke and North Hampshire Hospital	Catherine Rimington	
Basingstoke	Basingstoke and North Hampshire Hospital	Christina Narh	
Basingstoke	Basingstoke and North Hampshire Hospital	Claire Williams	
Basingstoke	Basingstoke and North Hampshire Hospital	Julie Gwilt	
Basingstoke	Basingstoke and North Hampshire Hospital	Kathryn Leach (nee Noake)	

Basingstoke	Basingstoke and North Hampshire Hospital	Liz Happle	
Basingstoke	Basingstoke and North Hampshire Hospital	Sara Fawcitt	
Bath	Royal United Hospital	Abigail Gee	Co-I
Bath	Royal United Hospital	Catherine McDonald	Co-I
Bath	Royal United Hospital	Claire Dyke	
Bath	Royal United Hospital	Frances Du Feu	
Bath	Royal United Hospital	Georgina Gullick	Co-I
Bath	Royal United Hospital	Ioana Fodor	
Bath	Royal United Hospital	Kathryn Falconer	
Bath	Royal United Hospital	Nathalie Webber	Co-I
Bath	Royal United Hospital	Tom Wilson	Co-I
Bath	Royal United Hospital	Abigail Jenner	
Bath	Royal United Hospital	Chris Williams	
Bath	Royal United Hospital	Christine Elwell	
Bath	Royal United Hospital	Gareth Ayre	
Bath	Royal United Hospital	Hugh Newman	
Bath	Royal United Hospital	Lorna Hawley	
Bath	Royal United Hospital	Mark Beresford	PI
Bath	Royal United Hospital	Matthew Sephton	
Bath	Royal United Hospital	Olivera Frim	Co-I
Bath	Royal United Hospital	Penny Kehagioglou	
Bath	Royal United Hospital	Susan Masson	
Bath	Royal United Hospital	Abigail Pocock	
Bath	Royal United Hospital	Claire Barron	
Bath	Royal United Hospital	Jill MacDonald-Burn	Pharmacist
Bath	Royal United Hospital	Joanne Avis	
Bath	Royal United Hospital	Leonie Harrison	
Bath	Royal United Hospital	Rachael Bolitho	
Bath	Royal United Hospital	Rachael Exley	
Bath	Royal United Hospital	Samantha Williams	Pharmacist
Bath	Royal United Hospital	Shaolin Chidavaenzi	
Bath	Royal United Hospital	Guillaume Livera	
Bath	Royal United Hospital	Joseph Needham	
Bath	Royal United Hospital	Roland Wynn-Williams	

Bath	Royal United Hospital	Michael Daly	
Bath	Royal United Hospital	Tom Tylee	
Bath	Royal United Hospital	Amy Singh	
Bath	Royal United Hospital	Joanna Wilson	
Bath	Royal United Hospital	Kristelle Vassallo	
Bath	Royal United Hospital	Rowan Appleby	
Bath	Royal United Hospital	Vicki Portingale	
Bath	Royal United Hospital	Beatrice Hamilton	
Bath	Royal United Hospital	Carey Milsom (nee Logan)	
Bath	Royal United Hospital	Christine Cox	
Bath	Royal United Hospital	Kate Moloney	
Bath	Royal United Hospital	Ruth Brydon-Hill	
Bath	Royal United Hospital	Samantha Curtis	
Bath	Royal United Hospital	Sarah Murdoch	
Bath	Royal United Hospital	Yuko Francis	
Bath	Royal United Hospital	Agata Leonarska	
Bath	Royal United Hospital	Bryony Robertson	
Bath	Royal United Hospital	Carolina Juan Chofre	
Bath	Royal United Hospital	Claire Davis	
Bath	Royal United Hospital	Eve Tomlinson	
Bath	Royal United Hospital	Jane Crozier	
Bath	Royal United Hospital	Jess White	
Bath	Royal United Hospital	Katarzyna Machura	
Bath	Royal United Hospital	Laura Cini	
Bath	Royal United Hospital	Rebecca Wassall	
Bath	Royal United Hospital	Carly Laxon-Takooree	
Bath	Royal United Hospital	Claire Craige	
Bath	Royal United Hospital	Hannah Blades	
Bath	Royal United Hospital	Jackie Davies	
Bath	Royal United Hospital	Margaret Macmillan	Pharmacist
Bath	Royal United Hospital	Vicki Clarke	
Bath	Royal United Hospital	Tania Williams (Née Allen)	
Bebington	Clatterbridge Centre for Oncology	Amir Montazeri	
Bebington	Clatterbridge Centre for Oncology	Azman Ibrahim	Co-I

Bebington	Clatterbridge Centre for Oncology	Helen Innes	
Bebington	Clatterbridge Centre for Oncology	Isabel Syndikus	
Bebington	Clatterbridge Centre for Oncology	Peter Robson	
Bebington	Clatterbridge Centre for Oncology	Shaun Tolan	
Bebington	Clatterbridge Centre for Oncology	Zafar Malik	PI
Bebington	Clatterbridge Centre for Oncology	Elizabeth Gallimore	
Bebington	Clatterbridge Centre for Oncology	Jodie Henderson	
Bebington	Clatterbridge Centre for Oncology	Rachel Pritchard	
Bebington	Clatterbridge Centre for Oncology	Sarah Dalby	
Bebington	Clatterbridge Centre for Oncology	Emma Whitby	
Bebington	Royal Liverpool University Hospital	Emma Whitby	
Bebington	Clatterbridge Centre for Oncology	Laura McAllister	
Bebington	Clatterbridge Centre for Oncology	Ian Allen	
bebington	University Hospital Aintree	Ian Allen	
Bebington	Clatterbridge Centre for Oncology	Matthew Stott	
Bebington	Clatterbridge Centre for Oncology	Paul Griffiths	
Bebington	Clatterbridge Centre for Oncology	Priyank Patel	
Bebington	Clatterbridge Centre for Oncology	Burhan Zavery	Pharmacist
Bebington	Clatterbridge Centre for Oncology	Wesley Artist	
Bebington	Clatterbridge Centre for Oncology	Annemieke Earnshaw	
Bebington	Clatterbridge Centre for Oncology	Elizabeth Harrison	
Bebington	Clatterbridge Centre for Oncology	Sharon Dunn (nee Johnson)	
Bebington	Clatterbridge Centre for Oncology	Sue Green	
Bebington	Clatterbridge Centre for Oncology	Diane Fildes	
Bebington	Clatterbridge Centre for Oncology	Helen Flint	Pharmacist
Bebington	Clatterbridge Centre for Oncology	Sandra Robinson	Pharmacist
Bebington	Clatterbridge Centre for Oncology	Caroline Dunn	
Bebington	Clatterbridge Centre for Oncology	Claire Harwood	
Bebington	Clatterbridge Centre for Oncology	Jess Hulse	
Bebington	Clatterbridge Centre for Oncology	Katie Sloan	
Bebington	Clatterbridge Centre for Oncology	Suzanne Maloney	
Bebington	Clatterbridge Centre for Oncology	Dawn Porter	
Bebington	Clatterbridge Centre for Oncology	Lisa Dobson (nee Child)	Pharmacist
Beckett Street	St James University Hospital (Leeds)	Catherine Gray	

Beckett Street	St James University Hospital (Leeds)	Claire Daisey	
Belfast	Belfast City Hospital	Claire Rooney	
Belfast	Belfast City Hospital	Laura Feeney	
Belfast	Belfast City Hospital	Laura Mooney	
Belfast	Belfast City Hospital	Michael McMahon	
Belfast	Belfast City Hospital	Nicola Hill	
Belfast	Belfast City Hospital	Orla Houlihan	
Belfast	Belfast City Hospital	Patricia Calisaya	
Belfast	Belfast City Hospital	Peter Bryson	
Belfast	Belfast City Hospital	Phil Turner	
Belfast	Belfast City Hospital	Rachel Ellis	
Belfast	Belfast City Hospital	Sai Jonnada	
Belfast	Belfast City Hospital	Seosamh McCauley	
Belfast	Belfast City Hospital	Swati Ray	Co-I
Belfast	Belfast City Hospital	Aiden Cole	
Belfast	Belfast City Hospital	Ciaran Fairmichael	
Belfast	Belfast City Hospital	Darren Mitchell	
Belfast	Belfast City Hospital	David Stewart	
Belfast	Belfast City Hospital	Jackie Harney	
Belfast	Belfast City Hospital	Jonathan McAleese	
Belfast	Belfast City Hospital	Lois Mulholland	
Belfast	Belfast City Hospital	Lucy Jellett	
Belfast	Belfast City Hospital	Melvyn Ang	
Belfast	Belfast City Hospital	Paula McCloskey	
Belfast	Belfast City Hospital	Poh Lin Shum	Co-I
Belfast	Belfast City Hospital	Prantik Das	
Belfast	Belfast City Hospital	Rebecca Goody	
Belfast	Belfast City Hospital	Rhun Evans	
Belfast	Belfast City Hospital	Ruth Eakin	
Belfast	Belfast City Hospital	Ruth Johnston	
Belfast	Belfast City Hospital	Salil Vengalil	
Belfast	Belfast City Hospital	Sarah McGahey	
Belfast	Belfast City Hospital	Stephen Stranex	
Belfast	Belfast City Hospital	Suneil Jain	Co-I

Belfast	Belfast City Hospital	Aine McKeown	
Belfast	Belfast City Hospital	Alison Logie	
Belfast	Belfast City Hospital	Jemma Robinson	
Belfast	Belfast City Hospital	Karen McKenna	
Belfast	Belfast City Hospital	Benedict Dadebo	
Belfast	Belfast City Hospital	Keith Rooney	
Belfast	Belfast City Hospital	Michael Hanna	
Belfast	Belfast City Hospital	William Snelling	
Belfast	Belfast City Hospital	Chris Hagan	
Belfast	Belfast City Hospital	Jonathan Thompson	
Belfast	Belfast City Hospital	Patrick Keane	
Belfast	Belfast City Hospital	Peter Clarke	
Belfast	Belfast City Hospital	Ciara McIlmunn	
Belfast	Belfast City Hospital	Diane Law	
Belfast	Belfast City Hospital	Ellen Brown	
Belfast	Belfast City Hospital	Fiona Tarpey	
Belfast	Belfast City Hospital	Karen Campfield	
Belfast	Belfast City Hospital	Kerry Nicholls	
Belfast	Belfast City Hospital	Margot Creighton	
Belfast	Belfast City Hospital	Eileen Dillon	
Belfast	Belfast City Hospital	Joanne McAllister	
Belfast	Belfast City Hospital	Kairen McCloy	
Belfast	Belfast City Hospital	Linda McNeice	Pharmacist
Belfast	Belfast City Hospital	Lynsey Morrow	
Belfast	Belfast City Hospital	Stacey Hetherington	
Belfast	Belfast City Hospital	Wendy Cunningham	
Belfast	Belfast City Hospital	Adrina O'Donnell	
Belfast	Belfast City Hospital	Catherine Davidson	
Belfast	Belfast City Hospital	Chryelle McAlister	
Belfast	Belfast City Hospital	Grace Lavery	
Belfast	Belfast City Hospital	Karen Allen (nee Parsons)	
Belfast	Belfast City Hospital	Sara McCusker(nee Stokes)	
Belfast	Belfast City Hospital	Sharon McClean	
Belfast	Belfast City Hospital	Sophie Lynch	

Belfast	Belfast City Hospital	Aishleen Brunton	
Belfast	Belfast City Hospital	Angela Morrison	
Belfast	Belfast City Hospital	Angela Rosbotham	
Belfast	Belfast City Hospital	Barbara Harvey	
Belfast	Belfast City Hospital	Eimear Henry	
Belfast	Belfast City Hospital	Emma Hanna	
Belfast	Belfast City Hospital	Geraldine Douris	
Belfast	Belfast City Hospital	Joanne Todd	
Belfast	Belfast City Hospital	Mairead Devine	
Belfast	Belfast City Hospital	Naomi Hill	
Belfast	Belfast City Hospital	Ruth Boyd	
Belfast	Belfast City Hospital	Sharon Hynds	
Belfast	Belfast City Hospital	Shirley McKenna	
Belfast	Belfast City Hospital	Joe O'Sullivan	PI
Bellinzona	Istituto Oncologico della Svizzera Italiana	Gianfranco Pesce	
Bellinzona	Istituto Oncologico della Svizzera Italiana	Maria Delgrande	
Bellinzona	Istituto Oncologico della Svizzera Italiana	Ngwa Che Azinwi	
Bellinzona	Istituto Oncologico della Svizzera Italiana	Ricardo Pereira Mestre	PI
Bellinzona	Istituto Oncologico della Svizzera Italiana	Vittoria Espeli	
Bellinzona	Istituto Oncologico della Svizzera Italiana	Carolina De Almeida	
Bellinzona	Istituto Oncologico della Svizzera Italiana	Eloise Kremer	
Berne	Inselspital (University Hospital Berne)	Timo Nannen	
Berne	Inselspital (University Hospital Berne)	Beat Roth	
Berne	Inselspital (University Hospital Berne)	Simone Rimoldi	
Berne	Inselspital (University Hospital Berne)	Susan Meierhans	
Berne	Inselspital (University Hospital Berne)	Anselm Lafita	
Berne	Inselspital (University Hospital Berne)	Anna-Katharina Herrmann	
Berne	Inselspital (University Hospital Berne)	Antje Ulrich	
Berne	Inselspital (University Hospital Berne)	Barbara Uhlmann	
Berne	Inselspital (University Hospital Berne)	Eloise Kremer	
Berne	Inselspital (University Hospital Berne)	Kathi Ochsner	
Berne	Inselspital (University Hospital Berne)	Daniel Aebersold	
Berne	Inselspital (University Hospital Berne)	Jörg Beyer	PI
Berne	Inselspital (University Hospital Berne)	George Thalmann	

Biel	Spitalzentrum Biel	Annette Winkler Vatter	
Biel	Spitalzentrum Biel	Béatrice Zimmerli Schwab	
Biel	Spitalzentrum Biel	Eloise Kremer	
Biel	Spitalzentrum Biel	Silvia Hanselmann	
Biel	Spitalzentrum Biel	Markus Borner	PI
Birmingham	Queen Elizabeth Hospital (Birmingham)	Amarpal Bains	
Birmingham	Queen Elizabeth Hospital (Birmingham)	Arvind Tripathy	
Birmingham	Queen Elizabeth Hospital (Birmingham)	Daniel Henderson	
Birmingham	Birmingham Heartlands Hospital	Frances Shaw	
Birmingham	Queen Elizabeth Hospital (Birmingham)	Jay Ansari	
Birmingham	City Hospital (Birmingham)	Lalit Pallan	
Birmingham	City Hospital (Birmingham)	Robert Stevenson	Co-I
Birmingham	City Hospital (Birmingham)	Sachin Trivedi	Co-I
Birmingham	Queen Elizabeth Hospital (Birmingham)	Sameed Hussain	
Birmingham	Queen Elizabeth Hospital (Birmingham)	Sudha Karanam	
Birmingham	Queen Elizabeth Hospital (Birmingham)	Syed Tirmazy	
Birmingham	Queen Elizabeth Hospital (Birmingham)	Abel Zachariah	
Birmingham	Birmingham Heartlands Hospital	Anjali Zarkar	PI
Birmingham	Queen Elizabeth Hospital (Birmingham)	Anjali Zarkar	
Birmingham	City Hospital (Birmingham)	Daniel Ford	
Birmingham	Queen Elizabeth Hospital (Birmingham)	Daniel Ford	Co-I
Birmingham	Queen Elizabeth Hospital (Birmingham)	David Fackrell	PI
Birmingham	City Hospital (Birmingham)	Emilio Porfiri	PI
Birmingham	Queen Elizabeth Hospital (Birmingham)	Emilio Porfiri	Co-I
Birmingham	Queen Elizabeth Hospital (Birmingham)	Erica Beaumont	
Birmingham	Queen Elizabeth Hospital (Birmingham)	Hannah Tween	
Birmingham	Queen Elizabeth Hospital (Birmingham)	Jenny Pascoe	
Birmingham	Queen Elizabeth Hospital (Birmingham)	Atiqa Ahmed	
Birmingham	Queen Elizabeth Hospital (Birmingham)	Charlotte Sabine	
Birmingham	Queen Elizabeth Hospital (Birmingham)	Charlotte Trinham	
Birmingham	Queen Elizabeth Hospital (Birmingham)	Daniella Lynch	
Birmingham	Queen Elizabeth Hospital (Birmingham)	Keia Spooner	
Birmingham	City Hospital (Birmingham)	Laura Butler	
Birmingham	Queen Elizabeth Hospital (Birmingham)	Laura Butler	

Birmingham	Queen Elizabeth Hospital (Birmingham)	Laura Caley	
Birmingham	Queen Elizabeth Hospital (Birmingham)	Rosie Henvey	
Birmingham	Birmingham Heartlands Hospital	Samarah Haq	
Birmingham	Birmingham Heartlands Hospital	Sanya Shafiq	
Birmingham	Queen Elizabeth Hospital (Birmingham)	Sibil Fernandez	
Birmingham	Queen Elizabeth Hospital (Birmingham)	Stephanie Palmer	
Birmingham	Queen Elizabeth Hospital (Birmingham)	Zhane Peterkin	
Birmingham	Queen Elizabeth Hospital (Birmingham)	Andrew Palmer	
Birmingham	Queen Elizabeth Hospital (Birmingham)	Biruk Asfaw	
Birmingham	Queen Elizabeth Hospital (Birmingham)	Christopher McGhee	
Birmingham	Queen Elizabeth Hospital (Birmingham)	Fahd Niaz	
Birmingham	Birmingham Heartlands Hospital	Michael Tarn	
Birmingham	Queen Elizabeth Hospital (Birmingham)	Richard Winter	
Birmingham	Queen Elizabeth Hospital (Birmingham)	Simon Horley	
Birmingham	City Hospital (Birmingham)	Steven Shanu	Pharmacist
Birmingham	Birmingham Heartlands Hospital	Adrian Kelly	
Birmingham	City Hospital (Birmingham)	Brian Gammon	
Birmingham	Birmingham Heartlands Hospital	Chen Bartlett	Pharmacist
Birmingham	Birmingham Heartlands Hospital	James Whitehouse	
Birmingham	Queen Elizabeth Hospital (Birmingham)	Vijay Patel	
Birmingham	Queen Elizabeth Hospital (Birmingham)	Vishy Veeranna	
Birmingham	Queen Elizabeth Hospital (Birmingham)	Aliyah Mannan	
Birmingham	City Hospital (Birmingham)	Amy Orme	
Birmingham	City Hospital (Birmingham)	Debbie Devonport	
Birmingham	Queen Elizabeth Hospital (Birmingham)	Fiona Evans	
Birmingham	Queen Elizabeth Hospital (Birmingham)	Golaleh McGinnell	
Birmingham	Queen Elizabeth Hospital (Birmingham)	Gullinder Jokhi	
Birmingham	Queen Elizabeth Hospital (Birmingham)	Karen Tester	
Birmingham	Queen Elizabeth Hospital (Birmingham)	Kathryn Adams	
Birmingham	Queen Elizabeth Hospital (Birmingham)	Lea Booth	
Birmingham	Birmingham Heartlands Hospital	Madhura Chandrashekara	
Birmingham	Queen Elizabeth Hospital (Birmingham)	Michelle Bates	
Birmingham	Queen Elizabeth Hospital (Birmingham)	Nasreen Akhtar	
Birmingham	Queen Elizabeth Hospital (Birmingham)	Nicola Betteridge	

Birmingham	Queen Elizabeth Hospital (Birmingham)	Parminder Sohal
Birmingham	Birmingham Heartlands Hospital	Ellen Drew
Birmingham	City Hospital (Birmingham)	Joanne Dasgin
Birmingham	Queen Elizabeth Hospital (Birmingham)	Pamela Jones
Birmingham	City Hospital (Birmingham)	Alice Longe
Birmingham	Queen Elizabeth Hospital (Birmingham)	Alice Longe
Birmingham	Queen Elizabeth Hospital (Birmingham)	Alison Grant
Birmingham	Queen Elizabeth Hospital (Birmingham)	Amanda Davies
Birmingham	Queen Elizabeth Hospital (Birmingham)	Amisha Desai
Birmingham	Queen Elizabeth Hospital (Birmingham)	Amna Shah
Birmingham	Birmingham Heartlands Hospital	Ann Schumacher
Birmingham	Birmingham Heartlands Hospital	Arlene Oldan
Birmingham	Queen Elizabeth Hospital (Birmingham)	Emma Bruce
Birmingham	Queen Elizabeth Hospital (Birmingham)	Hannah Tolson
Birmingham	Queen Elizabeth Hospital (Birmingham)	Heather Jones
Birmingham	Queen Elizabeth Hospital (Birmingham)	Helen Jones
Birmingham	Queen Elizabeth Hospital (Birmingham)	Helen Preston
Birmingham	Birmingham Heartlands Hospital	Janet Prentice
Birmingham	City Hospital (Birmingham)	Jasbinder Kaur
Birmingham	Queen Elizabeth Hospital (Birmingham)	Jenny Hiley
Birmingham	Birmingham Heartlands Hospital	Jill Lyons
Birmingham	Birmingham Heartlands Hospital	Julia Sampson
Birmingham	Birmingham Heartlands Hospital	Kamaldeep Ajimal
Birmingham	Queen Elizabeth Hospital (Birmingham)	Lisa Thomas
Birmingham	Birmingham Heartlands Hospital	Lisa - Marie Brueton
Birmingham	Queen Elizabeth Hospital (Birmingham)	Mahmoda Begum
Birmingham	Queen Elizabeth Hospital (Birmingham)	Maria Bandeira
Birmingham	Queen Elizabeth Hospital (Birmingham)	Nicola Anderson
Birmingham	Queen Elizabeth Hospital (Birmingham)	Salma Afzal
Birmingham	Queen Elizabeth Hospital (Birmingham)	Sam Hopkins (nee Poole)
Birmingham	Queen Elizabeth Hospital (Birmingham)	Sara Diffley
Birmingham	Queen Elizabeth Hospital (Birmingham)	Shaleen Bishop
Birmingham	Queen Elizabeth Hospital (Birmingham)	Sharon Holmes
Birmingham	Queen Elizabeth Hospital (Birmingham)	Tracy Soulsby

Birmingham	Queen Elizabeth Hospital (Birmingham)	Trish Brady	
Birmingham	Birmingham Heartlands Hospital	Alison Maidment	Pharmacist
Birmingham	City Hospital (Birmingham)	Angela Williams	
Birmingham	Queen Elizabeth Hospital (Birmingham)	Claire Brown	
Birmingham	Queen Elizabeth Hospital (Birmingham)	Claire Draycott	
Birmingham	Queen Elizabeth Hospital (Birmingham)	Fiona Catherine Stead	
Birmingham	Queen Elizabeth Hospital (Birmingham)	Fiona Catherine Stead	
Birmingham	Queen Elizabeth Hospital (Birmingham)	Gemma Cole	
Birmingham	City Hospital (Birmingham)	Harriet Goddard	
Birmingham	Queen Elizabeth Hospital (Birmingham)	Helen Clarke	
Birmingham	Queen Elizabeth Hospital (Birmingham)	Jane Cook	
Birmingham	Queen Elizabeth Hospital (Birmingham)	Joanna Gray (nee Finney)	
Birmingham	City Hospital (Birmingham)	Julie Simpson	Pharmacist
Birmingham	City Hospital (Birmingham)	Marion Tatman	
Birmingham	Birmingham Heartlands Hospital	Mary (Ellen) Drew	
Birmingham	Birmingham Heartlands Hospital	Penny Goodby (nee Harbach	
Birmingham	Queen Elizabeth Hospital (Birmingham)	Rosemarie Seadon	Pharmacist
Birmingham	City Hospital (Birmingham)	Yin May Chin	
Birmingham	Queen Elizabeth Hospital (Birmingham)	Nicholas James	PI
Blackburn	Royal Blackburn Hospital	Ahmed Salah	
Blackburn	Royal Blackburn Hospital	Alexandra Ferrera	
Blackburn	Royal Blackburn Hospital	Andrew Brocklehurst	
Blackburn	Royal Blackburn Hospital	Anthea Cree	
Blackburn	Royal Blackburn Hospital	Danya Abdulwahid	
Blackburn	Royal Blackburn Hospital	Ilyas Ahmed	
Blackburn	Royal Blackburn Hospital	Jasima Latif	
Blackburn	Royal Blackburn Hospital	Jennifer King	
Blackburn	Royal Blackburn Hospital	Karan Patel	
Blackburn	Royal Blackburn Hospital	Omi Parikh	PI
Blackburn	Royal Blackburn Hospital	Parth Desai	
Blackburn	Royal Blackburn Hospital	Prasad Kellati	
Blackburn	Royal Blackburn Hospital	Richard Walshaw	
Blackburn	Royal Blackburn Hospital	Sophia Callaghan	
Blackburn	Royal Blackburn Hospital	Sophie Raby	Co-I

Blackburn	Royal Blackburn Hospital	Twesige Mugisa	
Blackburn	Royal Blackburn Hospital	William Croxford	
Blackburn	Royal Blackburn Hospital	Zhu Oong	Co-I
Blackburn	Royal Blackburn Hospital	Ajay Mehta	
Blackburn	Royal Blackburn Hospital	Deborah Williamson	
Blackburn	Royal Blackburn Hospital	Falalu Danwata	
Blackburn	Royal Blackburn Hospital	Graham Read	
Blackburn	Royal Blackburn Hospital	Imran Haidar	
Blackburn	Royal Blackburn Hospital	Marcus Wise	
Blackburn	Royal Blackburn Hospital	Ruth Conroy	
Blackburn	Royal Blackburn Hospital	Tanmay Mukhopadhyay	
Blackburn	Royal Blackburn Hospital	Win Soe	
Blackburn	Royal Blackburn Hospital	Zia Rehman	
Blackburn	Royal Blackburn Hospital	Rizwana Hussain	Pharmacist
Blackburn	Royal Blackburn Hospital	Ana Batista	Pharmacist
Blackburn	Royal Blackburn Hospital	Farrah Burrows	
Blackburn	Royal Blackburn Hospital	Fatima Butt	
Blackburn	Royal Blackburn Hospital	Humairaa Timol	
Blackburn	Royal Blackburn Hospital	Naomi Charlton	
Blackburn	Royal Blackburn Hospital	Bethany Fielding	
Blackburn	Royal Blackburn Hospital	Andrew Hunnisett	
Blackburn	Royal Blackburn Hospital	Andrew Lancaster	
Blackburn	Royal Blackburn Hospital	Darren Rusk	Pharmacist
Blackburn	Royal Blackburn Hospital	James Grunshaw	
Blackburn	Royal Blackburn Hospital	Joseph Dykes	
Blackburn	Royal Blackburn Hospital	Matthew Lovell	
Blackburn	Royal Blackburn Hospital	Nicholas Pounder	
Blackburn	Royal Blackburn Hospital	Stephen Kilroy	
Blackburn	Royal Blackburn Hospital	Dayle Squires	
Blackburn	Royal Blackburn Hospital	Hani Hanna	Pharmacist
Blackburn	Royal Blackburn Hospital	Alison Blackburn	
Blackburn	Royal Blackburn Hospital	Deborah Smith	
Blackburn	Royal Blackburn Hospital	Farzana Patel	
Blackburn	Burnley General Hospital	Jacqueline Thomas	

Blackburn	Royal Blackburn Hospital	Jacqueline Thomas	
Blackburn	Royal Blackburn Hospital	Kathryn Hayes	
Blackburn	Burnley General Hospital	Lynsey Waring	
Blackburn	Royal Blackburn Hospital	Lynsey Waring	
Blackburn	Royal Blackburn Hospital	Angela Hugill	
Blackburn	Royal Blackburn Hospital	Helen Frankland	
Blackburn	Royal Blackburn Hospital	Janet Ryan-Smith	
Blackburn	Royal Blackburn Hospital	Sue Ashworth	
Blackburn	Royal Blackburn Hospital	Alexandra McCarrick	
Blackburn	Royal Blackburn Hospital	Christina Robinson	
Blackburn	Royal Blackburn Hospital	Debbie Sutton	Pharmacist
Blackburn	Royal Blackburn Hospital	Gaynor Bowen	
Blackburn	Royal Blackburn Hospital	Jackie Carey	
Blackburn	Royal Blackburn Hospital	Jackie Nuttall	
Blackburn	Royal Blackburn Hospital	Jan Flaherty	
Blackburn	Royal Blackburn Hospital	Jennifer McCallum	
Blackburn	Royal Blackburn Hospital	Jenny Cockerill-Taylor	
Blackburn	Royal Blackburn Hospital	Jessica Whiston	
Blackburn	Royal Blackburn Hospital	Marianna Theodoulou	
Blackburn	Royal Blackburn Hospital	Maricica Zabrautanu	
Blackburn	Royal Blackburn Hospital	Philippa Springle	
Blackburn	Royal Blackburn Hospital	Rachel Bolton	
Blackburn	Royal Blackburn Hospital	Samatha Guy	
Blackburn	Royal Blackburn Hospital	Sarah Ainsworth	
Blackburn	Burnley General Hospital	Sarah Keith	
Blackburn	Royal Blackburn Hospital	Sarah Keith	
Blackburn	Royal Blackburn Hospital	Tracey Kilduff	
Blackburn	Royal Blackburn Hospital	Victoria Taylor	Pharmacist
Blackburn	Royal Blackburn Hospital	Diane Forrest	
Blackburn	Royal Blackburn Hospital	Hazel Aston	
Blackburn	Royal Blackburn Hospital	Helene Chorley	
Blackburn	Royal Blackburn Hospital	Jeanette Hargreaves	
Blackburn	Royal Blackburn Hospital	Karen Beard	
Blackburn	Royal Blackburn Hospital	Karen Jewers	

Blackburn	Royal Blackburn Hospital	Vivienne Tickle	
Bolton	Royal Bolton Hospital	Ajay Mehta	
Bolton	Royal Bolton Hospital	Tony Elliott	
Bolton	Royal Bolton Hospital	Karen Lee	Pharmacist
Bolton	Royal Bolton Hospital	Gillian Mobb	
Bolton	Royal Bolton Hospital	Ling Lee	PI
Bolton	Royal Bolton Hospital	Hemant Patel	
Bolton	Royal Bolton Hospital	Michael Pantelides	
Bolton	Royal Bolton Hospital	Richard Jones	
Bolton	Royal Bolton Hospital	Robert Hull	
Bolton	Royal Bolton Hospital	Janet Keegan	
Bolton	Royal Bolton Hospital	Shirley Cocks	
Bolton	Royal Bolton Hospital	Charlotte Lever	
Bolton	Royal Bolton Hospital	Debbie Forkin	
Bolton	Royal Bolton Hospital	Janine Hurst	
Bolton	Royal Bolton Hospital	Julie Chadwick	
Bolton	Royal Bolton Hospital	Louise Dawson	
Bolton	Royal Bolton Hospital	Raksha Mistry	
Bolton	Royal Bolton Hospital	Sally Shaw	Pharmacist
Bolton	Royal Bolton Hospital	Zoe Gall	
Bolton	Royal Bolton Hospital	Collette Hunt	Pharmacist
Bolton	Royal Bolton Hospital	Karen Jewers	
Bolton	Royal Bolton Hospital	Lindsay Rawlinson	Pharmacist
Boston	Pilgrim Hospital	Ana Fernandez-Ots	Co-I
Boston	Pilgrim Hospital	Andrew Sloan	
Boston	Pilgrim Hospital	Christian Arias	Co-I
Boston	Pilgrim Hospital	David Ballesteros-Quintail	Co-I
Boston	Pilgrim Hospital	Sekar (DV) Kittappa	Co-I
Boston	Pilgrim Hospital	Sindhu Ramamurthy	
Boston	Pilgrim Hospital	Miguel Panades	PI
Boston	Pilgrim Hospital	Prantik Das	
Boston	Pilgrim Hospital	Eileen Busby	
Boston	Pilgrim Hospital	Gunjan Phalod	
Boston	Pilgrim Hospital	Karen Metcalf	

Boston	Pilgrim Hospital	Sally Ann Molsher	
Boston	Pilgrim Hospital	Andrew Judd	
Boston	Pilgrim Hospital	Simon Archer	
Boston	Pilgrim Hospital	Beverley Mashegede	
Boston	Pilgrim Hospital	Isobel Thomas	
Boston	Pilgrim Hospital	Kimberley Netherton	
Boston	Pilgrim Hospital	Kinga Szymiczek	
Boston	Pilgrim Hospital	Laura Walsh	
Boston	Pilgrim Hospital	Kerry Pettitt	
Boston	Pilgrim Hospital	Victoria Knight (n. Sherburn)	
Boston	Pilgrim Hospital	Amanda Roper	
Boston	Pilgrim Hospital	Amy Kirkby	
Boston	Pilgrim Hospital	Giuseppe Banna	
Boston	Pilgrim Hospital	Helen Carolan	
Boston	Pilgrim Hospital	Helen Palmer	Pharmacist
Boston	Pilgrim Hospital	Jayne Borley	Pharmacist
Boston	Pilgrim Hospital	Jenny Salmon	
Boston	Pilgrim Hospital	Jo Fletcher	
Boston	Pilgrim Hospital	Rebecca Spencer	Pharmacist
Boston	Pilgrim Hospital	Tara Lawrence nee Palmer	
Boston	Pilgrim Hospital	Alice Latty	
Boston	Pilgrim Hospital	Anita Young	
Boston	Pilgrim Hospital	Bryony Saint	
Boston	Pilgrim Hospital	Carol Lockwood	
Boston	Pilgrim Hospital	Trish Tsuro	
Bournemouth	Royal Bournemouth Hospital	Kenneth Oguejiofor	
Bournemouth	Royal Bournemouth Hospital	Matthew Roberts	
Bournemouth	Royal Bournemouth Hospital	Deborah Hands	
Bournemouth	Royal Bournemouth Hospital	George Astras	
Bournemouth	Royal Bournemouth Hospital	Joe Davies	
Bournemouth	Royal Bournemouth Hospital	Rao Vuyyuru	
Bournemouth	Royal Bournemouth Hospital	Sue Brock	PI
Bournemouth	Royal Bournemouth Hospital	Tom Geldart	Co-I
Bournemouth	Royal Bournemouth Hospital	Julie Thomson	

Bournemouth	Royal Bournemouth Hospital	Laura Purandare	
Bournemouth	Royal Bournemouth Hospital	Mirela Mukaj	
Bournemouth	Royal Bournemouth Hospital	Natasha Ottley	
Bournemouth	Royal Bournemouth Hospital	Taslima Rabbi	
Bournemouth	Royal Bournemouth Hospital	Alison Hogan	Pharmacist
Bournemouth	Royal Bournemouth Hospital	Rachel Bower	
Bournemouth	Royal Bournemouth Hospital	Ben Loat	
Bournemouth	Royal Bournemouth Hospital	Cameron Huck	
Bournemouth	Royal Bournemouth Hospital	David Chrastek	
Bournemouth	Royal Bournemouth Hospital	Joseph Cavill	
Bournemouth	Royal Bournemouth Hospital	Luke Vamplew	
Bournemouth	Royal Bournemouth Hospital	Carlton Rowlands	
Bournemouth	Royal Bournemouth Hospital	Roger Hudson	
Bournemouth	Royal Bournemouth Hospital	Emma Sharland	
Bournemouth	Royal Bournemouth Hospital	Joanne Sheppard	
Bournemouth	Royal Bournemouth Hospital	Cathie Purnell	
Bournemouth	Royal Bournemouth Hospital	Linda Purandare	
Bournemouth	Royal Bournemouth Hospital	Min Wu	
Bournemouth	Royal Bournemouth Hospital	Katherine Major	
Bournemouth	Royal Bournemouth Hospital	Lynsey Houlton	
Bournemouth	Royal Bournemouth Hospital	Rebecca Miln	
Bournemouth	Royal Bournemouth Hospital	Sarah Savage	
Bournemouth	Royal Bournemouth Hospital	Sophie Jackson	
Bournemouth	Royal Bournemouth Hospital	Stephanie Jones	
Bournemouth	Royal Bournemouth Hospital	Tiffany Joyce	
Bournemouth	Royal Bournemouth Hospital	Eve Broadley	
Bournemouth	Royal Bournemouth Hospital	Kate Preece	
Bournemouth	Royal Bournemouth Hospital	Natalya Boyd	
Bradford	Bradford Royal Infirmary	Adel Jebar	Co-I
Bradford	Bradford Royal Infirmary	Andrew Viggars	Co-I
Bradford	Bradford Royal Infirmary	Ann Henry	
Bradford	Bradford Royal Infirmary	Anthi Zeniou	
Bradford	Bradford Royal Infirmary	Catherine Handforth	
Bradford	Bradford Royal Infirmary	Charlotte Richardson	

Bradford	Bradford Royal Infirmary	Christopher Williams	
Bradford	Bradford Royal Infirmary	Eldho Joseph	Co-I
Bradford	Bradford Royal Infirmary	Emily Montague	
Bradford	Bradford Royal Infirmary	Finbar Slevin	
Bradford	Bradford Royal Infirmary	Ian Boon	
Bradford	Bradford Royal Infirmary	Jessica Pearce	Co-I
Bradford	Bradford Royal Infirmary	Leila Koudsi	
Bradford	Bradford Royal Infirmary	Louise Karsera	Co-I
Bradford	Bradford Royal Infirmary	Love Goyal	
Bradford	Bradford Royal Infirmary	Lucy Jones	Co-I
Bradford	Bradford Royal Infirmary	Lucy Ward	
Bradford	Bradford Royal Infirmary	Manjusha Soni	Co-I
Bradford	Bradford Royal Infirmary	Reem Mahmood	
Bradford	Bradford Royal Infirmary	Sally Martin	Co-I
Bradford	Bradford Royal Infirmary	Samuel Briggs	
Bradford	Bradford Royal Infirmary	Simon Brown	PI
Bradford	Bradford Royal Infirmary	Sohail Mughal	Co-I
Bradford	Bradford Royal Infirmary	Sree Rodda	
Bradford	Bradford Royal Infirmary	Carmel Loughrey	
Bradford	Bradford Royal Infirmary	Dan Lee	
Bradford	Bradford Royal Infirmary	Ganesan Jeyasangar	
Bradford	Bradford Royal Infirmary	Jamal Zekri	
Bradford	Bradford Royal Infirmary	Sue Cheeseman	
Bradford	St Luke's (Bradford)	Susan Cheeseman	
Bradford	Bradford Royal Infirmary	You Yone	
Bradford	Bradford Royal Infirmary	Eleanor Waldron	
Bradford	Bradford Royal Infirmary	Jannika Lazarte	
Bradford	Bradford Royal Infirmary	Lucille Kenyon	
Bradford	Bradford Royal Infirmary	Qamar Akbar	
Bradford	Bradford Royal Infirmary	Declan Ryan-Wakeling	
Bradford	Bradford Royal Infirmary	Kelvin Stewart	
Bradford	Bradford Royal Infirmary	Leslie Masters	
Bradford	Bradford Royal Infirmary	Umair Hamid	Pharmacist
Bradford	Bradford Royal Infirmary	Mohammed Patel	

Bradford	Bradford Royal Infirmary	Osman Chohan	Pharmacist
Bradford	Bradford Royal Infirmary	Richard Benton	
Bradford	Bradford Royal Infirmary	Carol Firth	
Bradford	Bradford Royal Infirmary	Dawn McNulty	
Bradford	Bradford Royal Infirmary	Helen Wilson	
Bradford	Bradford Royal Infirmary	Kay Cockroft	
Bradford	Bradford Royal Infirmary	Manitha Vinod	
Bradford	Bradford Royal Infirmary	Wendy Cardozo	
Bradford	Bradford Royal Infirmary	Hayley Inman	
Bradford	Bradford Royal Infirmary	Jane Sewell	
Bradford	Bradford Royal Infirmary	Sarah Tinker	
Bradford	Bradford Royal Infirmary	Chandran Nallathambi	
Bradford	Bradford Royal Infirmary	Charlotte Johnson-Smith	
Bradford	Bradford Royal Infirmary	Elizabeth McIntosh	
Bradford	Bradford Royal Infirmary	Gail Opio-Te	
Bradford	Bradford Royal Infirmary	Jacqueline Quantrill	
Bradford	Bradford Royal Infirmary	Kim Storton	
Bradford	Bradford Royal Infirmary	Robina Ghulam	
Bradford	Bradford Royal Infirmary	Shefali Parikh	
Bradford	Bradford Royal Infirmary	Susan Shorter	
Bradford	Bradford Royal Infirmary	Victoria Drew	
Bradford	Bradford Royal Infirmary	Anne Marie Kay	
Bradford	Bradford Royal Infirmary	Laura Jaques	
Bradford	Bradford Royal Infirmary	Linda Bamford	
Bradford	Bradford Royal Infirmary	Sophia Khan	
Bradford	Bradford Royal Infirmary	Sophie Stephenson	
Brighton	Royal Sussex County Hospital	Ashok Nikapota	
Brighton	Royal Sussex County Hospital	Aun Mohammad	
Brighton	Royal Sussex County Hospital	George Plantaniotis	
Brighton	Royal Sussex County Hospital	Angus Robinson	PI
Brighton	Royal Sussex County Hospital	Daniel Henderson	
Brighton	Royal Sussex County Hospital	David Bloomfield	
Brighton	Royal Sussex County Hospital	George Devtsch	
Brighton	Royal Sussex County Hospital	George Plataniotis	Co-I

Brighton	Royal Sussex County Hospital	Jackie Sham	
Brighton	Royal Sussex County Hospital	Marie Wilkins	
Brighton	Royal Sussex County Hospital	Tarun Durga	
Brighton	Royal Sussex County Hospital	Vivien Tse	
Brighton	Royal Sussex County Hospital	Angela Man	
Brighton	Royal Sussex County Hospital	Catherine Hunter	
Brighton	Royal Sussex County Hospital	Chritianne Whitfield	
Brighton	Royal Sussex County Hospital	Lisa Furnival	
Brighton	Royal Sussex County Hospital	Rachel Rose Edmunds	
Brighton	Royal Sussex County Hospital	Samantha Hodges	
Brighton	Royal Sussex County Hospital	Summer Ibrahim	
Brighton	Royal Sussex County Hospital	Jean Tremlett	
Brighton	Royal Sussex County Hospital	Andrew Hart	
Brighton	Royal Sussex County Hospital	Matthew Seal	
Brighton	Royal Sussex County Hospital	Paul Frattaroli	
Brighton	Royal Sussex County Hospital	Sebastien Martin	
Brighton	Royal Sussex County Hospital	Tiago Rodrigues	
Brighton	Royal Sussex County Hospital	Simon Matthews	
Brighton	Royal Sussex County Hospital	Stephen Brown	
Brighton	Royal Sussex County Hospital	Caroline Walker	
Brighton	Royal Sussex County Hospital	Dorota Bak-Blaz	
Brighton	Royal Sussex County Hospital	Karen Walker	
Brighton	Royal Sussex County Hospital	Emma Foreman	Pharmacist
Brighton	Royal Sussex County Hospital	Alison Porges	
Brighton	Royal Sussex County Hospital	Amy Murray	
Brighton	Royal Sussex County Hospital	Annie Oliver	
Brighton	Royal Sussex County Hospital	Elizabeth Corbett	
Brighton	Royal Sussex County Hospital	Jane Peterson	
Brighton	Royal Sussex County Hospital	Joanne Magennis	
Brighton	Royal Sussex County Hospital	Jodie Smith	
Brighton	Royal Sussex County Hospital	Katie Langford	
Brighton	Royal Sussex County Hospital	Lucy Curtis	
Brighton	Royal Sussex County Hospital	Poppy Lavender	
Brighton	Royal Sussex County Hospital	Ranee Lactao	

Brighton	Royal Sussex County Hospital	Bobbie Yoong	
Brighton	Royal Sussex County Hospital	Elaine Noon	
Brighton	Royal Sussex County Hospital	Helen Mitchell	
Brighton	Royal Sussex County Hospital	Jane Dexter	
Brighton	Royal Sussex County Hospital	Jane Hanson	
Brighton	Royal Sussex County Hospital	Julie Smith	
Brighton	Royal Sussex County Hospital	Kirsty Bracewell	
Brighton	Royal Sussex County Hospital	Lisa Barrott	
Brighton	Royal Sussex County Hospital	Maggie Cole	
Brighton	Royal Sussex County Hospital	Monika Musiol	
Brighton	Royal Sussex County Hospital	Pauline Martin	
Brighton	Royal Sussex County Hospital	Sue Trotter	
Brighton	Royal Sussex County Hospital	Tamsin Kent	
Brighton	Royal Sussex County Hospital	Tenesa Sargent	
Brighton	Royal Sussex County Hospital	Victoria Sellick	
Bristol	Bristol Haematology & Oncology Centre	Amit Bahl	PI
Bristol	Bristol Haematology & Oncology Centre	Chris Herbert	
Bristol	Bristol Haematology & Oncology Centre	Emily Foulstone	
Bristol	Bristol Haematology & Oncology Centre	Hugh Newman	
Bristol	Bristol Haematology & Oncology Centre	Jyothsna Chennupati	
Bristol	Bristol Haematology & Oncology Centre	Mark Beresford	
Bristol	Bristol Haematology & Oncology Centre	Paula Wilson	
Bristol	Bristol Haematology & Oncology Centre	Serena Hilman	
Bristol	Bristol Haematology & Oncology Centre	Susan Masson	
Bristol	Bristol Haematology & Oncology Centre	Eve Watson	
Bristol	Bristol Haematology & Oncology Centre	Kay Drury	
Bristol	Bristol Haematology & Oncology Centre	Azeem Arshad	
Bristol	Bristol Haematology & Oncology Centre	Nick Robins	
Bristol	Bristol Haematology & Oncology Centre	Sibusiso Dhladhla	
Bristol	Bristol Haematology & Oncology Centre	Harvey Dymond	
Bristol	Bristol Haematology & Oncology Centre	Ian Penwarden	Pharmacist
Bristol	Bristol Haematology & Oncology Centre	Lloyd Abood	
Bristol	Bristol Haematology & Oncology Centre	Marc Coe	
Bristol	Bristol Haematology & Oncology Centre	Robert Hollister	

Bristol	Bristol Haematology & Oncology Centre	Stephen Lang	
Bristol	Bristol Haematology & Oncology Centre	Tristan Grey	
Bristol	Bristol Haematology & Oncology Centre	Shalini Mohan	
Bristol	Bristol Haematology & Oncology Centre	Beth Thorne	
Bristol	Bristol Haematology & Oncology Centre	Amy Holloway	Pharmacist
Bristol	Bristol Haematology & Oncology Centre	Bryony Parrish	
Bristol	Bristol Haematology & Oncology Centre	Helen Saldanha	
Bristol	Bristol Haematology & Oncology Centre	Hayley Jones	
Bristol	Bristol Haematology & Oncology Centre	Jayne Leonard	
Bristol	Bristol Haematology & Oncology Centre	Kimberly Rockley	
Bristol	Bristol Haematology & Oncology Centre	Lindsay Ball	Pharmacist
Bristol	Bristol Royal Infirmary	Lindsay Ball	
Bristol	Bristol Haematology & Oncology Centre	Polly Gingell	
Bristol	Bristol Haematology & Oncology Centre	Sally-Ann Hall	Pharmacist
Bristol	Bristol Haematology & Oncology Centre	Sandra Williams (nee Price)	Pharmacist
Bristol	Bristol Haematology & Oncology Centre	Sarah Bishop	
Bristol	Bristol Haematology & Oncology Centre	Seonaid Wright	
Bristol	Bristol Haematology & Oncology Centre	Sharon Short	
Burnley	Burnley General Hospital	Ahmed Salah	
Burnley	Burnley General Hospital	Andrew Brocklehurst	
Burnley	Burnley General Hospital	Anthea Cree	
Burnley	Burnley General Hospital	Danya Abdulwahid	
Burnley	Burnley General Hospital	Ilyas Ahmed	
Burnley	Burnley General Hospital	Karan Patel	
Burnley	Burnley General Hospital	Omi Parikh	PI
Burnley	Burnley General Hospital	Prasad Kellati	
Burnley	Burnley General Hospital	Richard Walshaw	
Burnley	Burnley General Hospital	Twesige Mugisa	
Burnley	Burnley General Hospital	Deborah Williamson	
Burnley	Burnley General Hospital	Imran Haidar	
Burnley	Burnley General Hospital	Marcus Wise	
Burnley	Burnley General Hospital	Ruth Conroy	
Burnley	Burnley General Hospital	Tanmay Mukhopadhyay	
Burnley	Burnley General Hospital	Win Soe	

Burnley	Burnley General Hospital	Zia Rehman	
Burnley	Burnley General Hospital	Rizwana Hussain	Pharmacist
Burnley	Burnley General Hospital	Ana Batista	Pharmacist
Burnley	Burnley General Hospital	Fatima Butt	
Burnley	Burnley General Hospital	Humairaa Timol	
Burnley	Burnley General Hospital	Bethany Fielding	
Burnley	Burnley General Hospital	Darren Rusk	
Burnley	Burnley General Hospital	Matthew Lovell	
Burnley	Burnley General Hospital	Stephen Kilroy	
Burnley	Burnley General Hospital	Dayle Squires	
Burnley	Burnley General Hospital	Hani Hanna	Pharmacist
Burnley	Burnley General Hospital	Farzana Patel	
Burnley	Burnley General Hospital	Angela Hugill	
Burnley	Burnley General Hospital	Helen Frankland	
Burnley	Burnley General Hospital	Janet Ryan-Smith	
Burnley	Burnley General Hospital	Sue Ashworth	
Burnley	Burnley General Hospital	Alexandra McCarrick	
Burnley	Burnley General Hospital	Christina Robinson	
Burnley	Burnley General Hospital	Debbie Sutton	
Burnley	Burnley General Hospital	Gaynor Bowen	
Burnley	Burnley General Hospital	Jackie Carey	
Burnley	Burnley General Hospital	Jan Flaherty	
Burnley	Burnley General Hospital	Karen Riley	
Burnley	Royal Blackburn Hospital	Karen Riley	
Burnley	Burnley General Hospital	Philippa Springle	
Burnley	Burnley General Hospital	Samatha Guy	
Burnley	Burnley General Hospital	Sarah Ainsworth	
Burnley	Burnley General Hospital	Tracey Kilduff	
Burnley	Burnley General Hospital	Victoria Taylor	Pharmacist
Burnley	Burnley General Hospital	Diane Forrest	
Burnley	Burnley General Hospital	Helene Chorley	
Burnley	Burnley General Hospital	Jeanette Hargreaves	
Burnley	Burnley General Hospital	Karen Beard	
Burnley	Burnley General Hospital	Karen Jewers	

Burnley	Burnley General Hospital	Louise Dawson	
Burnley	Burnley General Hospital	Vivienne Tickle	
Burton upon Trent	Queen's Hospital Burton	Prabir Chakraborti	
Burton upon Trent	Queen's Hospital Burton	Rosemary Corfield	
Burton-on-Trent	Queen's Hospital Burton	Anita Szita	
Burton-on-Trent	Queen's Hospital Burton	Chandrani Mallik	
Burton-on-Trent	Queen's Hospital Burton	Rohit Malde	
Burton-on-Trent	Queen's Hospital Burton	Seheli Bandyopahdyay	
Burton-on-Trent	Queen's Hospital Burton	Sudipta Datta	
Burton-on-Trent	Queen's Hospital Burton	Chris Curtis	
Burton-on-Trent	Queen's Hospital Burton	Dakshinamoorthy Muthukumar	
Burton-on-Trent	Queen's Hospital Burton	Divya Ramadasan	
Burton-on-Trent	Queen's Hospital Burton	Hanine Medani	
Burton-on-Trent	Queen's Hospital Burton	Karzan Hama	
Burton-on-Trent	Queen's Hospital Burton	Mike Smith-Howell	PI
Burton-on-Trent	Queen's Hospital Burton	Rajeev Kaushal	
Burton-on-Trent	Queen's Hospital Burton	Shahzad Ahmed	
Burton-on-Trent	Queen's Hospital Burton	Shan Chetiyawardana	
Burton-on-Trent	Queen's Hospital Burton	V Gajek	
Burton-on-Trent	Queen's Hospital Burton	Pugazhenthi Pattu	
Burton-on-Trent	Queen's Hospital Burton	Ali Mahmmod	
Burton-on-Trent	Queen's Hospital Burton	Lorraine Carter	
Burton-on-Trent	Queen's Hospital Burton	Annette Fleet	
Burton-on-Trent	Queen's Hospital Burton	Elizabeth Kemp	
Burton-on-Trent	Queen's Hospital Burton	Jennifer Moyes	Pharmacist
Burton-on-Trent	Queen's Hospital Burton	Gill Bell	
Burton-on-Trent	Queen's Hospital Burton	Jo Burns	
Burton-on-Trent	Queen's Hospital Burton	Katy English (nee Parkes)	
Burton-on-Trent	Queen's Hospital Burton	Sarah Hathaway-Lees	
Burton-on-Trent	Queen's Hospital Burton	Ann Adams	Pharmacist
Burton-on-Trent	Queen's Hospital Burton	Clare Mewies	
Burton-on-Trent	Queen's Hospital Burton	Helen Cox	
Burton-on-Trent	Queen's Hospital Burton	Helena Cox	
Burton-on-Trent	Queen's Hospital Burton	Jacqueline Elliott	

Bury St Edmunds	West Suffolk Hospital	Alex Martin	Co-I
Bury St Edmunds	West Suffolk Hospital	Yvonne Rimmer	
Bury St Edmunds	West Suffolk Hospital	David Matter	
Bury St Edmunds	West Suffolk Hospital	Fred Tuck	
Bury St Edmunds	West Suffolk Hospital	John Raja Ravendar	
Bury St Edmunds	West Suffolk Hospital	Mark Heath	
Bury St Edmunds	West Suffolk Hospital	Amanda Neal	
Bury St Edmunds	West Suffolk Hospital	Jill Thain	
Bury St Edmunds	West Suffolk Hospital	Lisa Patterson	
Bury St Edmunds	West Suffolk Hospital	Rachel Stocking	
Bury St Edmunds	West Suffolk Hospital	Susan Hale	
Bury St Edmunds	West Suffolk Hospital	Yvonne Field	
Bury St Edmunds	West Suffolk Hospital	Deborah Clements-Dimmock	
Bury St Edmunds	West Suffolk Hospital	Frances Flynn	
Bury St Edmunds	West Suffolk Hospital	Joanne Kellett	
Bury St Edmunds	West Suffolk Hospital	Elizabeth Devoy	
Bury St Edmunds	West Suffolk Hospital	Gill Brett	
Bury St Edmunds	West Suffolk Hospital	Helen Small	
Bury St Edmunds	West Suffolk Hospital	Tracey Murray	
Bury St. Edmonds	West Suffolk Hospital	Cathryn Woodward	PI
Bury St. Edmunds	West Suffolk Hospital	James Curtis	Pharmacist
Bury St. Edmunds	West Suffolk Hospital	Cherri Blades	
Camarthen	Glangwili General (formerly West Wales General)	Mau-Don Phan	PI
Camarthen	Glangwili General (formerly West Wales General)	Sonya Goriah	Co-I
Camarthen	Glangwili General (formerly West Wales General)	Sandra Griffiths nee Evens	
Camarthen	Glangwili General (formerly West Wales General)	Bryan Phillips	
Camarthen	Glangwili General (formerly West Wales General)	Bleddyn Edwards	
Camarthen	Glangwili General (formerly West Wales General)	Meena Raj	
Camarthen	Glangwili General (formerly West Wales General)	Ann Hewins	
Camarthen	Glangwili General (formerly West Wales General)	Samantha Coetzee	
Camarthen	Glangwili General (formerly West Wales General)	Rocio Riba	Pharmacist
Camarthen	Glangwili General (formerly West Wales General)	Zohra Omar	
Cambridge	Addenbrooke's Hospital	Gin Lee	
Cambridge	Addenbrooke's Hospital	Andrew Styling	

Cambridge	Addenbrooke's Hospital	Hossameldin Attia	Co-I
Cambridge	Addenbrooke's Hospital	James Jones	
Cambridge	Addenbrooke's Hospital	James Tanner	
Cambridge	Addenbrooke's Hospital	Kamarul Zaki	
Cambridge	Addenbrooke's Hospital	Mirela Hategan	
Cambridge	Addenbrooke's Hospital	Nicola Thompson	
Cambridge	Addenbrooke's Hospital	Sian Pugh	
Cambridge	Addenbrooke's Hospital	Simon Pacey	
Cambridge	Addenbrooke's Hospital	Danish Mazhar	PI
Cambridge	Addenbrooke's Hospital	Han Wong	
Cambridge	Addenbrooke's Hospital	Luke Hughes-Davies	
Cambridge	Addenbrooke's Hospital	Richard Benson	
Cambridge	Addenbrooke's Hospital	Kate Beesley	
Cambridge	Addenbrooke's Hospital	Safaa Therese	
Cambridge	Addenbrooke's Hospital	James Watson	
Cambridge	Addenbrooke's Hospital	Matthew Stone	
Cambridge	Addenbrooke's Hospital	Isaac Opara	
Cambridge	Addenbrooke's Hospital	Amy Strong n.Chandradass	
Cambridge	Addenbrooke's Hospital	Jane Bushen	
Cambridge	Addenbrooke's Hospital	Sandra Cunningham	
Cambridge	Addenbrooke's Hospital	Svitlana Iyevkova	
Cambridge	Addenbrooke's Hospital	Tatiana Hernandez	Co-I
Cambridge	Addenbrooke's Hospital	Anita Chhabra	Pharmacist
Cambridge	Addenbrooke's Hospital	Abigail Frost	
Cambridge	Addenbrooke's Hospital	Carole Hewitt	
Cambridge	Addenbrooke's Hospital	Ellie Couch	
Cambridge	Addenbrooke's Hospital	Gemma Cullen (née Godsall)	
Cambridge	Addenbrooke's Hospital	Rachel Lister	
Cambridge	Addenbrooke's Hospital	Rebecca Bradley	
Cambridge	Addenbrooke's Hospital	Amanda Walker	
Cambridge	Addenbrooke's Hospital	Debra Mansergh	Pharmacist
Cambridge	Addenbrooke's Hospital	Glynn Rolland	
Cambridge	Addenbrooke's Hospital	Vanessa Moreira	
Cantebruy	Queen Elizabeth The Queen Mother Hospital	Jessica Little	Co-I

Cantebury	Queen Elizabeth The Queen Mother Hospital	Jennifer Turner	Co-I
Cantebury	William Harvey Hospital	Jennifer Turner	Co-I
Cantebury	William Harvey Hospital	Jessica Little	Co-I
Canterbury	Kent and Canterbury Hospital	Albert Edwards	Co-I
Canterbury	Kent and Canterbury Hospital	Charlotte Mott	
Canterbury	Kent and Canterbury Hospital	Fiora Elwes	Co-I
Canterbury	Kent and Canterbury Hospital	Ifigenia Vasiliadou	
Canterbury	Kent and Canterbury Hospital	Ilyas Ahmed	
Canterbury	Kent and Canterbury Hospital	Ioannis Trigonis	Co-I
Canterbury	Kent and Canterbury Hospital	Jennifer Turner	Co-I
Canterbury	Kent and Canterbury Hospital	Jessica Gough	Co-I
Canterbury	Kent and Canterbury Hospital	Lavarniya Rajakumar	
Canterbury	Kent and Canterbury Hospital	Mathini Sridharan	
Canterbury	Kent and Canterbury Hospital	Patryk Brulinski	Co-I
Canterbury	Kent and Canterbury Hospital	Rakesh Raman	Co-I
Canterbury	Kent and Canterbury Hospital	Rohit Malde	
Canterbury	Kent and Canterbury Hospital	Sarah Beasley	
Canterbury	Kent and Canterbury Hospital	Stephane Tankoua	
Canterbury	Kent and Canterbury Hospital	Alice Rendall	Co-I
Canterbury	Kent and Canterbury Hospital	Arafat Mizra	
Canterbury	Kent and Canterbury Hospital	Carys Thomas	PI
Canterbury	Kent and Canterbury Hospital	Christos Mikropoulos	
Canterbury	Kent and Canterbury Hospital	Clary Evans	
Canterbury	Kent and Canterbury Hospital	Gemma Hegarty	
Canterbury	Kent and Canterbury Hospital	Jessica Little	Co-I
Canterbury	Kent and Canterbury Hospital	Joao Galante	Co-I
Canterbury	Kent and Canterbury Hospital	Kannon Nathan	
Canterbury	Kent and Canterbury Hospital	Kathryn Lees	
Canterbury	Kent and Canterbury Hospital	Mathilda Cominos	
Canterbury	Kent and Canterbury Hospital	Matthew Fenton	
Canterbury	Kent and Canterbury Hospital	Mohammed Osman	Co-I
Canterbury	Kent and Canterbury Hospital	Natasha Mithal	Co-I
Canterbury	Kent and Canterbury Hospital	Sharon Beesley	
Canterbury	Kent and Canterbury Hospital	Sugeeta Sukumar	

Canterbury	Kent and Canterbury Hospital	Udaiveer Panwar	
Canterbury	Kent and Canterbury Hospital	Van Sim	Co-I
Canterbury	Kent and Canterbury Hospital	Coral Greenstreet	
Canterbury	Kent and Canterbury Hospital	Hayley Blackgrove	
Canterbury	Kent and Canterbury Hospital	Katy Taylor	
Canterbury	Kent and Canterbury Hospital	Victoria Williamson	
Canterbury	Kent and Canterbury Hospital	Natalie Catt	
Canterbury	Kent and Canterbury Hospital	Arafat Mirza	
Canterbury	Kent and Canterbury Hospital	Sam Gibson	
Canterbury	Kent and Canterbury Hospital	Steve Dann	
Canterbury	Kent and Canterbury Hospital	Andrew Gillian	Pharmacist
Canterbury	Kent and Canterbury Hospital	Miguel Capo-Mir	Pharmacist
Canterbury	Kent and Canterbury Hospital	Cindy Slater	
Canterbury	Kent and Canterbury Hospital	Diane Long	
Canterbury	Kent and Canterbury Hospital	Hasmath Marjolin	
Canterbury	Kent and Canterbury Hospital	Laura Mould	
Canterbury	Kent and Canterbury Hospital	Nikki Crisp	
Canterbury	Kent and Canterbury Hospital	Rachel Larkins	
Canterbury	Kent and Canterbury Hospital	Sandra Holness	
Canterbury	Kent and Canterbury Hospital	Sarah Lines	
Canterbury	Kent and Canterbury Hospital	Susan Rogers	
Canterbury	Kent and Canterbury Hospital	Claire White	
Canterbury	Kent and Canterbury Hospital	Julie Buckley	
Canterbury	Kent and Canterbury Hospital	Laura Kehoe	
Canterbury	Kent and Canterbury Hospital	Lesley Rose	
Canterbury	Kent and Canterbury Hospital	Louise Gladwell	
Canterbury	Kent and Canterbury Hospital	Sarah Lightfoot	Pharmacist
Canterbury	Kent and Canterbury Hospital	Tracy Boakes	
Canterbury	Kent and Canterbury Hospital	Alba Tubau	
Canterbury	Kent and Canterbury Hospital	Bonny Appleby	
Canterbury	Kent and Canterbury Hospital	Caroline Sunderland	
Canterbury	Kent and Canterbury Hospital	Carolyn Hargreaves	
Canterbury	Kent and Canterbury Hospital	Linda Wray	
Canterbury	Kent and Canterbury Hospital	Louise Allen	

Canterbury	Kent and Canterbury Hospital	Marian Wood	
Canterbury	Kent and Canterbury Hospital	Adedolapo Sanni	
Canterbury	Kent and Canterbury Hospital	Claire Pelham	
Canterbury	Kent and Canterbury Hospital	Elizabeth Williamson	
Canterbury	Kent and Canterbury Hospital	Hilary Zurakovsky	
Canterbury	Kent and Canterbury Hospital	Jill Baker	
Canterbury	Kent and Canterbury Hospital	Joanne Williams	
Canterbury	Kent and Canterbury Hospital	Julie-Ann Davies	
Canterbury	Kent and Canterbury Hospital	Karen Robinson	
Canterbury	Kent and Canterbury Hospital	Kathleen (Kathy) Walsh	
Canterbury	Kent and Canterbury Hospital	Kim Mears	
Canterbury	Kent and Canterbury Hospital	Kim Travis	
Canterbury	Kent and Canterbury Hospital	Margaret Lipsham	
Canterbury	Kent and Canterbury Hospital	Paula Whichelo	
Canterbury	Kent and Canterbury Hospital	Sharon Middleton	
Canterbury	Kent and Canterbury Hospital	Sue Kelly	
Canterbury	Kent and Canterbury Hospital	Susan Drakeley	
Canterbury	Kent and Canterbury Hospital	Sydnie Loveland	
Canterbury	Kent and Canterbury Hospital	Molua Young	
Canterbury	Kent and Canterbury Hospital	Denise Crawford	
Cardiff	Velindre Hospital	Aida Hanim Kamarudin	Co-I
Cardiff	Velindre Hospital	Alok Chand	
Cardiff	Velindre Hospital	Andrew Kidd	Co-I
Cardiff	Velindre Hospital	Clair Brunner	
Cardiff	Velindre Hospital	Lisa Victoria Jane Clayton	
Cardiff	Velindre Hospital	Michael Button	Co-I
Cardiff	Velindre Hospital	Nida Hassan	
Cardiff	Velindre Hospital	Satish Kumar	Co-I
Cardiff	Velindre Hospital	Srijith Sashidharan	
Cardiff	Velindre Hospital	Diana Mort	
Cardiff	Velindre Hospital	Jacob Tanguay	PI
Cardiff	Velindre Hospital	Jason Lester	
Cardiff	Velindre Hospital	Jim Barber	Co-I
Cardiff	Velindre Hospital	John Staffurth	Co-I

Cardiff	Velindre Hospital	Louise Harris	
Cardiff	Velindre Hospital	Nachiappan Palaniappan	Co-I
Cardiff	University Hospital of Wales	Elizabeth Bois (nee Harris)	
Cardiff	Velindre Hospital	Emily Rumney	
Cardiff	Velindre Hospital	Emma Cook	
Cardiff	Velindre Hospital	Naomi Woods	
Cardiff	Velindre Hospital	Rebecca Mitchell	
Cardiff	Velindre Hospital	Sandra Greenslade	
Cardiff	Velindre Hospital	Christian Smith	
Cardiff	Velindre Hospital	Gareth Hunt	Pharmacist
Cardiff	Velindre Hospital	James Morgan	
Cardiff	University Hospital of Wales	Krishna Narahari	PI
Cardiff	Velindre Hospital	Phillip Morgan	
Cardiff	Velindre Hospital	Rashmi Jadon	
Cardiff	Velindre Hospital	Robert Henley	
Cardiff	Velindre Hospital	Ross McLeish	
Cardiff	Velindre Hospital	Toby Hiscott	
Cardiff	University Hospital of Wales	Kevin Pearse	
Cardiff	Velindre Hospital	Michael Brown	Pharmacist
Cardiff	University Hospital of Wales	Richard Coulthard	
Cardiff	Velindre Hospital	Amanda Jcekia	
Cardiff	Velindre Hospital	Gillian Willetts	
Cardiff	University Hospital of Wales	Helen Clark	
Cardiff	Velindre Hospital	Helen Clark	
Cardiff	Velindre Hospital	Jayne Richards	
Cardiff	Velindre Hospital	Lynda Holman	
Cardiff	University Hospital of Wales	Samantha Holliday	
Cardiff	Velindre Hospital	Tracy Rees	
Cardiff	Velindre Hospital	Caroline Vitolo	
Cardiff	University Hospital of Wales	Clare Geere (nee Jones)	
Cardiff	Velindre Hospital	Hana Thomas	
Cardiff	Velindre Hospital	Kay Wilson	
Cardiff	University Hospital of Wales	Lynne Harry	
Cardiff	Velindre Hospital	Alison Johnson	

Cardiff	Velindre Hospital	Catherine Sullivan	
Cardiff	Velindre Hospital	Clare Donnithorne	Pharmacist
Cardiff	University Hospital of Wales	Colette Clements	
Cardiff	Velindre Hospital	Colette Kemp	
Cardiff	Velindre Hospital	Debbie O'Connor	
Cardiff	Velindre Hospital	Gladys Makuta	
Cardiff	Velindre Hospital	Jessica Dermott (nee Platt)	
Cardiff	Velindre Hospital	Joanne Preece	
Cardiff	Velindre Hospital	Lisa Stafford	
Cardiff	Velindre Hospital	Loretta Sweeney	
Cardiff	Velindre Hospital	Louise Morgan	
Cardiff	Velindre Hospital	Lucy Chestney	
Cardiff	Velindre Hospital	Necia Jones	
Cardiff	Velindre Hospital	Renata Poole	Pharmacist
Cardiff	Velindre Hospital	Sarah Fry	
Cardiff	Velindre Hospital	Sonya Osborne	
Cardiff	Velindre Hospital	Amanda Jackson	
Cardiff	Velindre Hospital	Bethan Tranter	
Cardiff	Velindre Hospital	Catherine John	
Cardiff	Velindre Hospital	Cathy Richards	Pharmacist
Cardiff	Velindre Hospital	Charlotte Young	
Cardiff	Velindre Hospital	Clare Boobier	
Cardiff	Velindre Hospital	Donna Lear	
Cardiff	Velindre Hospital	Karen Pow	Pharmacist
Cardiff	Velindre Hospital	Kathy Bishop	
Cardiff	Velindre Hospital	Leanne Quinn	
Cardiff	Velindre Hospital	Lucy Wilbraham	
Cardiff	Velindre Hospital	Vicki Reynolds	
Cardiff	University Hospital of Wales	Howard Kynaston	
Cardiff	Velindre Hospital	Malcolm Mason	Co-I
Cardiff	Velindre Hospital	Lynette Lane	
Carlisle	Cumberland Infirmary	Fiona Douglas	
Carlisle	Cumberland Infirmary	Anil Kumar	PI
Carlisle	Cumberland Infirmary	Fergus Young	Pathologist

Carlisle	Cumberland Infirmary	Jonathan Nicoll	
Carlisle	Cumberland Infirmary	Muhammad Rahman	
Carlisle	Cumberland Infirmary	Norma Sidek	
Carlisle	Cumberland Infirmary	Jenna Wildey	
Carlisle	Cumberland Infirmary	Christopher Brewer	Pharmacist
Carlisle	Cumberland Infirmary	Ivor Hughes	Pharmacist
Carlisle	Cumberland Infirmary	Beverley Wilkinson	
Carlisle	Cumberland Infirmary	Patricia Nicholls	
Carlisle	Cumberland Infirmary	Charlotte Eyles	
Carlisle	Cumberland Infirmary	Grace Fryer	
Carlisle	Cumberland Infirmary	Angela Birt	
Carlisle	Cumberland Infirmary	Diane Donnelly	Pharmacist
Chelmsford	Broomfield Hospital	Isabella Maund	
Chelmsford	Broomfield Hospital	Abdel Hamid	PI
Chelmsford	Broomfield Hospital	Gopalakrishnan Srinivasan	Co-I
Chelmsford	Broomfield Hospital	Kiran Kancherla	
Chelmsford	Broomfield Hospital	Priscilla Leone	
Chelmsford	Broomfield Hospital	Udaiveer Panwar	
Chelmsford	Broomfield Hospital	You Yone	
Chelmsford	Broomfield Hospital	Jennifer Child	
Chelmsford	Broomfield Hospital	Emma Mitchell	
Chelmsford	Broomfield Hospital	Amon Wijunamai	
Chelmsford	Broomfield Hospital	Dane Goodere-Bennett	
Chelmsford	Broomfield Hospital	Bryan Singizi	
Chelmsford	Broomfield Hospital	Christian Barnett	
Chelmsford	Broomfield Hospital	Melanie Boxall	
Chelmsford	Broomfield Hospital	Melanie Ruben	
Chelmsford	Broomfield Hospital	Victoria Apps	
Chelmsford	Broomfield Hospital	Donna Briggs	Pharmacist
Chelmsford	Broomfield Hospital	Emma Cannon	Pharmacist
Chelmsford	Broomfield Hospital	Jane Giles	Pharmacist
Chelmsford	Broomfield Hospital	Elizabeth Dawson	
Chelmsford	Broomfield Hospital	Emma Cannon	
Chelmsford	Broomfield Hospital	Lucy Willsher	

Chelmsford	Broomfield Hospital	Nicola Cutmore	
Chelmsford	Broomfield Hospital	Victoria Scott	
Chelmsford	Broomfield Hospital	Edel Spruce	
Chelmsford	Broomfield Hospital	Frances Cairns	Pharmacist
Chelmsford	Broomfield Hospital	Lucy Cooper	Pharmacist
Chelmsford	Broomfield Hospital	Sarah Ferguson	
Chelmsford	Broomfield Hospital	Sian Gibson	
Chelmsford	Broomfield Hospital	Yvonne Lester	
Chelmsford	Broomfield Hospital	Tracey Camburn	
Chelsmford	Broomfield Hospital	Enca Parsons	
Cheltenham	Cheltenham General Hospital	Caitlin Bowden	
Cheltenham	Cheltenham General Hospital	Laura Malins	
Cheltenham	Cheltenham General Hospital	Sai Jonnada	
Cheltenham	Cheltenham General Hospital	Victoria Bell	
Cheltenham	Cheltenham General Hospital	Vishal Bhalla	
Cheltenham	Cheltenham General Hospital	Alex Williams	
Cheltenham	Cheltenham General Hospital	Audrey Cook	
Cheltenham	Cheltenham General Hospital	Duncan Stow	
Cheltenham	Cheltenham General Hospital	Jo Bowen	PI
Cheltenham	Cheltenham General Hospital	Jyothsna Chennupati	
Cheltenham	Cheltenham General Hospital	Peter Jenkins	Co-I
Cheltenham	Cheltenham General Hospital	Roger Owen	
Cheltenham	Cheltenham General Hospital	Amy Skelton	
Cheltenham	Cheltenham General Hospital	Bethan Cartwright	
Cheltenham	Cheltenham General Hospital	Louise Kidner	
Cheltenham	Cheltenham General Hospital	Lucy Blake	
Cheltenham	Cheltenham General Hospital	Madelaine Smith	
Cheltenham	Cheltenham General Hospital	Sarah Stanley	
Cheltenham	Cheltenham General Hospital	Rachel Carter	
Cheltenham	Cheltenham General Hospital	Sarah Beazer	
Cheltenham	Cheltenham General Hospital	Matthew Tan	
Cheltenham	Cheltenham General Hospital	Samuel Croly	
Cheltenham	Cheltenham General Hospital	Ian Ingledew	
Cheltenham	Cheltenham General Hospital	Richard Wallis	Pharmacist

Cheltenham	Cheltenham General Hospital	Helen Babbage	
Cheltenham	Cheltenham General Hospital	Jennifer Dewett	
Cheltenham	Cheltenham General Hospital	Jennifer Smith	
Cheltenham	Cheltenham General Hospital	Jill Chittock	
Cheltenham	Cheltenham General Hospital	Julia Hall	
Cheltenham	Cheltenham General Hospital	Julie Allen	
Cheltenham	Cheltenham General Hospital	Lin Crossley	
Cheltenham	Cheltenham General Hospital	Nicola Robinson	
Cheltenham	Cheltenham General Hospital	Rachel Durrant	Pharmacist
Cheltenham	Cheltenham General Hospital	Rachel Sayers	
Cheltenham	Cheltenham General Hospital	Chris Ford	
Cheltenham	Cheltenham General Hospital	Kate Trigg-Hogarth	
Cheltenham	Cheltenham General Hospital	Sue Wronski	
Cheltenham	Cheltenham General Hospital	Susan Anderson	
Cheltenham	Cheltenham General Hospital	Abi Stuart	
Cheltenham	Cheltenham General Hospital	Charlotte Ayrton	
Cheltenham	Cheltenham General Hospital	Elaine Sizer	
Cheltenham	Cheltenham General Hospital	Elisabeth Read	Pharmacist
Cheltenham	Cheltenham General Hospital	Rebecca Mesher	
Cheltenham	Cheltenham General Hospital	Catherine Stuart-Grumbar	
Cheltenham	Cheltenham General Hospital	Janet Forkes	
Cheltenham	Cheltenham General Hospital	Jennifer Healey-Mariano	
Cheltenham	Cheltenham General Hospital	Rehana Bakawala	
Chester	Countess of Chester Hospital	Azman Ibrahim	PI
Chester	Countess of Chester Hospital	Elizabeth Gallimore	
Chester	Countess of Chester Hospital	Emma Barry	
Chester	Countess of Chester Hospital	Grace McGrath	
Chester	Countess of Chester Hospital	Jenny Miller	
Chester	Countess of Chester Hospital	Lucy Beresford	
Chester	Countess of Chester Hospital	Shannon Spicer	
Chester	Countess of Chester Hospital	Joshua Williams	
Chester	Countess of Chester Hospital	Judith Prince	
Chester	Countess of Chester Hospital	Wesley Artist	Pharmacist
Chester	Countess of Chester Hospital	Chelcie Faulkner	

Chester	Countess of Chester Hospital	Helen Jeffrey	
Chester	Countess of Chester Hospital	Jenny Grounds	
Chester	Countess of Chester Hospital	Kathryn Cawley	
Chester	Countess of Chester Hospital	Rebecca Grogan	
Chester	Countess of Chester Hospital	Rebecca Hopcroft	
Chester	Countess of Chester Hospital	Sarah Illingworth	
Chester	Countess of Chester Hospital	Sue Green	
Chester	Countess of Chester Hospital	Helen Eccleson	
Chester	Countess of Chester Hospital	Denise Archer	
Chester	Countess of Chester Hospital	Janet Spriggs	
Chester	Countess of Chester Hospital	Lisa Dobson (nee Child)	Pharmacist
Chester	Countess of Chester Hospital	Mary Aldous	
Chur	Kantonsspital Graubünden	Dirk Kienle	
Chur	Kantonsspital Graubünden	M Mark	
Chur	Kantonsspital Graubünden	Michael Schwitter	
Chur	Kantonsspital Graubünden	Raeto Strebel	PI
Chur	Kantonsspital Graubünden	Richard Cathomas	
Chur	Kantonsspital Graubünden	Roger von Moos	
Chur	Kantonsspital Graubünden	Carin Aebli	
Chur	Kantonsspital Graubünden	Eloise Kremer	
Chur	Kantonsspital Graubünden	Gabriela Manetsch	
Chur	Kantonsspital Graubünden	Radmila Moudry	Pharmacist
Colchester	Colchester General Hospital	Anita Szita	Co-I
Colchester	Colchester General Hospital	Muthar Kumar	
Colchester	Essex County Hospital	Muthar Kumar	
Colchester	Colchester General Hospital	Sunil Skaria	
Colchester	Colchester General Hospital	Bruce Sizer	Co-I
Colchester	Essex County Hospital	Bruce Sizer	
Colchester	Colchester General Hospital	Dakshinamoorthy Muthukumar	PI
Colchester	Colchester General Hospital	Devy Basu	Co-I
Colchester	Essex County Hospital	Devy Basu	
Colchester	Colchester General Hospital	Rana Mahmood	Co-I
Colchester	Colchester General Hospital	Nicola Taylor	
Colchester	Essex County Hospital	Pugazhenthi Pattu	

Colchester	Colchester General Hospital	Richard Gant	Pharmacist
Colchester	Colchester General Hospital	Louies Mabelin	
Colchester	Colchester General Hospital	Liz Hunting	
Colchester	Essex County Hospital	Liz Hunting	
Colchester	Colchester General Hospital	Lucy Thorogood	
Colchester	Essex County Hospital	Lucy Thorogood	
Colchester	Colchester General Hospital	Katrina Cooke	
Colchester	Colchester General Hospital	Michelle Fisher	
Colchester	Colchester General Hospital	Nicola Cutmore	
Colchester	Colchester General Hospital	Celine Driscoll	
Colchester	Essex County Hospital	Celine Driscoll	
Colchester	Colchester General Hospital	Daisuke Takeuchi	
Colchester	Colchester General Hospital	Hayley Hewer	
Colchester	Essex County Hospital	Hayley Hewer	
Colchester	Colchester General Hospital	Jane Ketley-O'Donel	Pharmacist
Colchester	Essex County Hospital	Jane Ketley-O'Donel	
Colchester	Essex County Hospital	Lorna Dewar	
Colchester	Colchester General Hospital	Michelle Marshall	
Colchester	Essex County Hospital	Michelle Marshall	
Cookridge	Cookridge Hospital	Carmel Loughrey	
Cosham	Queen Alexandra Hospital	Robert Keating	
Cottingham	Castle Hill Hospital	Dulani Ranatunge	
Cottingham	Castle Hill Hospital	George Bozat	
Cottingham	Castle Hill Hospital	Iqtedar Muazzam	
Cottingham	Castle Hill Hospital	Jenny Marsden	
Cottingham	Castle Hill Hospital	Khawaje Zahid	
Cottingham	Castle Hill Hospital	Louise Karsera	
Cottingham	Castle Hill Hospital	Mohan Hingorani	Co-I
Cottingham	Castle Hill Hospital	Faheem Bashir	Co-I
Cottingham	Castle Hill Hospital	Mateen Akhtar	
Cottingham	Castle Hill Hospital	Mohammad Butt	Co-I
Cottingham	Castle Hill Hospital	Bob Bush	Pharmacist
Cottingham	Castle Hill Hospital	A Yousuff	
Cottingham	Castle Hill Hospital	Adam Wolstencroft	Pharmacist

Cottingham	Castle Hill Hospital	Ian Beckley	
Cottingham	Castle Hill Hospital	John Hetherington	
Cottingham	Castle Hill Hospital	Jonathan Gill	
Cottingham	Castle Hill Hospital	Kristian Plowman	
Cottingham	Castle Hill Hospital	Matthew Simms	PI
Cottingham	Castle Hill Hospital	Karen Stubbs	
Cottingham	Castle Hill Hospital	Linzi Bone	
Cottingham	Castle Hill Hospital	Paula O'Reilly	
Cottingham	Castle Hill Hospital	Julie Rawlings	
Cottingham	Castle Hill Hospital	Lucy Richardson	
Cottingham	Castle Hill Hospital	Mary Garthwaite	
Cottingham	Castle Hill Hospital	Sarah Moffat	
Cottingham	Castle Hill Hospital	Suzy Bunton	
Cottingham	Castle Hill Hospital	Carol Hodson	
Cottingham	Castle Hill Hospital	Linda Hoggarth	
Cottingham	Castle Hill Hospital	Sarah Brown	
Cottingham	Castle Hill Hospital	Sarah Palmer	
Cottingham	Castle Hill Hospital	Vicki Lowthorpe	
Coventry	University Hospital Coventry and Warwickshire	Michael Tilby	PI
Coventry	University Hospital Coventry and Warwickshire	Shah Rafique	Co-I
Coventry	University Hospital Coventry and Warwickshire	Andrew Chan	
Coventry	University Hospital Coventry and Warwickshire	Andrew Stockdale	
Coventry	University Hospital Coventry and Warwickshire	Jane Worlding	
Coventry	University Hospital Coventry and Warwickshire	Joanna Hamilton	Co-I
Coventry	University Hospital Coventry and Warwickshire	Senthil Kumar Athmanathan	
Coventry	University Hospital Coventry and Warwickshire	Yakhub Khan	PI
Coventry	University Hospital Coventry and Warwickshire	Fiona McGurk	Pharmacist
Coventry	University Hospital Coventry and Warwickshire	Lucy Miller	
Coventry	University Hospital Coventry and Warwickshire	Mariam Bharuchi	
Coventry	University Hospital Coventry and Warwickshire	Rajbinder Deol	
Coventry	University Hospital Coventry and Warwickshire	Vicky Sturgess	
Coventry	University Hospital Coventry and Warwickshire	Donald Macdonald	
Coventry	University Hospital Coventry and Warwickshire	Mohamed Mooradun	
Coventry	University Hospital Coventry and Warwickshire	Mohammed Khan	Pharmacist

Coventry	University Hospital Coventry and Warwickshire	Albert Mislang	
Coventry	University Hospital Coventry and Warwickshire	Jason Allen	
Coventry	University Hospital Coventry and Warwickshire	Karandeepu Pachoo	Pharmacist
Coventry	University Hospital Coventry and Warwickshire	Kieran Jefferson	
Coventry	University Hospital Coventry and Warwickshire	Mark Whitmore	
Coventry	University Hospital Coventry and Warwickshire	Sukhbinder Salh	Pharmacist
Coventry	Coventry and Warwickshire Hospital	Kathleen Rose	
Coventry	University Hospital Coventry and Warwickshire	Kathleen Rose	
Coventry	University Hospital Coventry and Warwickshire	Laura Stanley	
Coventry	Coventry and Warwickshire Hospital	Leila Fortunato	
Coventry	University Hospital Coventry and Warwickshire	Noor Ayesha Shah	
Coventry	University Hospital Coventry and Warwickshire	Zoe O'Neill	
Coventry	University Hospital Coventry and Warwickshire	Elaine Simmons	
Coventry	University Hospital Coventry and Warwickshire	Rachel Bazeley	
Coventry	University Hospital Coventry and Warwickshire	Sonia Powell	
Coventry	University Hospital Coventry and Warwickshire	Theresa Griffiths	
Coventry	University Hospital Coventry and Warwickshire	Vikki Browne	
Coventry	University Hospital Coventry and Warwickshire	Charlie-marie Suddens	
Coventry	University Hospital Coventry and Warwickshire	Dannielle Burgess	
Coventry	University Hospital Coventry and Warwickshire	Fiona Tranter	
Coventry	University Hospital Coventry and Warwickshire	Jenny Warmington	
Coventry	University Hospital Coventry and Warwickshire	Luanne Carey	
Coventry	University Hospital Coventry and Warwickshire	Padama Singh	Pharmacist
Coventry	University Hospital Coventry and Warwickshire	Sarah O'Toole	
Coventry	University Hospital Coventry and Warwickshire	Stacey Clarke	Pharmacist
Coventry	University Hospital Coventry and Warwickshire	Su Ngwenya	
Coventry	University Hospital Coventry and Warwickshire	Sue Robinson	
Coventry	University Hospital Coventry and Warwickshire	Tammi-Lea Beeby	
Coventry	University Hospital Coventry and Warwickshire	Linda Wimbush	
Coventry	University Hospital Coventry and Warwickshire	Maggie Brown	
Coventry	University Hospital Coventry and Warwickshire	Rachel Thompson	Pharmacist
Coventry	University Hospital Coventry and Warwickshire	Rebecca Aaron	Pharmacist
Crewe	Leighton Hospital	William Croxford	
Crewe	Leighton Hospital	Anna Tran	PI

Crewe	Leighton Hospital	Catherine Thompson	
Crewe	Leighton Hospital	David Butterworth	
Crewe	Leighton Hospital	James Wylie	
Crewe	Leighton Hospital	Michael Braun	
Crewe	Leighton Hospital	Adele Hough	
Crewe	Leighton Hospital	Annabel Tomlinson	
Crewe	Leighton Hospital	Gemma Nash	
Crewe	Leighton Hospital	Karen Hillyer	
Crewe	Leighton Hospital	Katherine Hampton	
Crewe	Leighton Hospital	Sarah Tinsley	Pharmacist
Crewe	Leighton Hospital	Andrew Ritchings	Pharmacist
Crewe	Leighton Hospital	Osman Chohan	
Crewe	Leighton Hospital	P Irwin	
Crewe	Leighton Hospital	P Javle	
Crewe	Leighton Hospital	Carole Bennion	
Crewe	Leighton Hospital	Caroline Walker	
Crewe	Leighton Hospital	Joanne Hughes	
Crewe	Leighton Hospital	Julie Meir	
Crewe	Leighton Hospital	Karen Wilson	
Crewe	Leighton Hospital	Kim Best	
Crewe	Leighton Hospital	Leanne Everall	
Crewe	Leighton Hospital	Lydia Buxton	
Crewe	Leighton Hospital	Nicola Ritchings	
Crewe	Leighton Hospital	Chris Hough	
Crewe	Leighton Hospital	Bethan Roberts	
Crewe	Leighton Hospital	Emma Margerum	
Crewe	Leighton Hospital	Jane Sellman	
Crewe	Leighton Hospital	Karen Gilbert	
Crewe	Leighton Hospital	Rachel Smith	
Crewe	Leighton Hospital	Taya Jones	Pharmacist
Crewe	Leighton Hospital	Thiraviyam Elumalai	
Crewe	Leighton Hospital	Tracy Larcombe	
Crewe	Leighton Hospital	Carolyn Mansfield	
Crewe	Leighton Hospital	Julia Gemmell	

Crewe	Leighton Hospital	Sarah Hoswell	
Crewe	Leighton Hospital	Vanessa Adamson	
Croydon	Croydon University Hospital	Yvonne Campbell	
Croydon	Croydon University Hospital	Ann Payne	
Croydon	Croydon University Hospital	Anne Haldeos	
Croydon	Croydon University Hospital	Cheryl Batish	
Darlington	Darlington Memorial Hospital	Mohammed Kagzi	PI
Darlington	Darlington Memorial Hospital	Clive Peedell	
Darlington	Darlington Memorial Hospital	John Hardman	
Darlington	Darlington Memorial Hospital	Julia McBride	
Darlington	Darlington Memorial Hospital	Steven Pratt	
Darlington	Darlington Memorial Hospital	Tanmay Mukhopadhyay	
Darlington	Darlington Memorial Hospital	Rachel Chatt	
Darlington	Darlington Memorial Hospital	Jonathan Stoddard	
Darlington	Darlington Memorial Hospital	Richard Nendick	Pharmacist
Darlington	Darlington Memorial Hospital	Calum Polwart	Pharmacist
Darlington	Darlington Memorial Hospital	Hyder Latif	
Darlington	Darlington Memorial Hospital	John Vickers	
Darlington	Darlington Memorial Hospital	Asia Sarwar	
Darlington	Darlington Memorial Hospital	Helen Haley	
Darlington	Darlington Memorial Hospital	Kimberly Stamp	
Darlington	Darlington Memorial Hospital	Lynsey Stephenson	Pharmacist
Darlington	Darlington Memorial Hospital	Alison Chilvers	
Darlington	Darlington Memorial Hospital	Claire Henderson	
Darlington	Darlington Memorial Hospital	Susan Wadd	
Darlington	Darlington Memorial Hospital	Lorna Morgan	
Darlington	Darlington Memorial Hospital	Fiona Strong	
Darlington	Darlington Memorial Hospital	Jane Shaw	
Darlington	Darlington Memorial Hospital	Penny Gamble	
Dartford	Darent Valley Hospital	Louise Lacey	
Derb	Queen's Hospital Burton	Christopher Kent	
Derby	Royal Derby Hospital	Ajith Gopinathan Nair	
Derby	Royal Derby Hospital	Alastair McCabe	
Derby	Royal Derby Hospital	Ayman Ramadan	

Derby	Royal Derby Hospital	Chin-Hiong Chong	
Derby	Royal Derby Hospital	Jun Hao Lim	
Derby	Royal Derby Hospital	Kiran Das	
Derby	Royal Derby Hospital	Lauren Jones	
Derby	Royal Derby Hospital	Maeve Pomeroy	
Derby	Royal Derby Hospital	Peter Mason	
Derby	Royal Derby Hospital	Sadia Abdullah	
Derby	Royal Derby Hospital	Seheli Bandyopahdyay	
Derby	Royal Derby Hospital	Thangarajah Mugunthan	
Derby	Royal Derby Hospital	Timothy Podd	
Derby	Royal Derby Hospital	Virgil Sivoglo	
Derby	Royal Derby Hospital	Christopher Kent	
Derby	London Road Community Hospital	Dakshinamoorthy Muthukumar	
Derby	Royal Derby Hospital	Dakshinamoorthy Muthukumar	
Derby	Royal Derby Hospital	Jessica Davies	
Derby	Royal Derby Hospital	Lokesh Puttarachaiah	
Derby	Royal Derby Hospital	Louise Brookes	
Derby	Royal Derby Hospital	Mike Smith-Howell	
Derby	London Road Community Hospital	Prabir Chakraborti	
Derby	Royal Derby Hospital	Prabir Chakraborti	
Derby	Royal Derby Hospital	Prantik Das	PI
Derby	Royal Derby Hospital	Rania Mohammed	
Derby	Royal Derby Hospital	Sarah Taylor	
Derby	Royal Derby Hospital	Sathan Boonyaprapa	
Derby	Royal Derby Hospital	Shahzad Ahmed	
Derby	Royal Derby Hospital	Caroline Coulson	
Derby	Royal Derby Hospital	Georgia Wright	
Derby	Royal Derby Hospital	Helen Beveridge	
Derby	Royal Derby Hospital	Marie Ann Goldsworthy	Pharmacist
Derby	London Road Community Hospital	Heini Jussila	
Derby	London Road Community Hospital	Keeley Smith	
Derby	Royal Derby Hospital	Chris Worth	
Derby	Royal Derby Hospital	James Aldous	
Derby	Royal Derby Hospital	Pugazhenthi Pattu	

Derby	Royal Derby Hospital	Aaron Gallagher	
Derby	London Road Community Hospital	Colin Ward	
Derby	Royal Derby Hospital	Colin Ward	Pharmacist
Derby	Royal Derby Hospital	Fanuel Magaya	
Derby	Royal Derby Hospital	Alison Carrick	
Derby	Royal Derby Hospital	Charlotte Downes	
Derby	Royal Derby Hospital	Donna Beal	
Derby	Royal Derby Hospital	Elizabeth Nadin	
Derby	Royal Derby Hospital	Joely Morgan	
Derby	Royal Derby Hospital	Kashmira Subramanian	
Derby	Royal Derby Hospital	Margaret Harper	
Derby	London Road Community Hospital	Wendy Morrisroe	
Derby	Royal Derby Hospital	Wendy Morrisroe	
Derby	Royal Derby Hospital	Elizabeth Bedford	
Derby	Royal Derby Hospital	Ellie Piggott	
Derby	Royal Derby Hospital	Josephine Chmiel	
Derby	Royal Derby Hospital	Liz Bedford	
Derby	Royal Derby Hospital	Lucy McCandless	
Derby	Royal Derby Hospital	Manni Sandhu	
Derby	Royal Derby Hospital	Nicole McKee	
Derby	Royal Derby Hospital	Nicole McKee (nee Isitt)	
Derby	Royal Derby Hospital	Wendy Abbott	Pharmacist
Derby	Royal Derby Hospital	Claire Wintle	
Derby	Royal Derby Hospital	Emily Mignott	
Derby	Royal Derby Hospital	Emma Brooks	
Derby	Royal Derby Hospital	Hege Strand	
Derby	Royal Derby Hospital	Janet Tomlinson	
Derby	Royal Derby Hospital	Jennifer Mitchell	
Derby	Royal Derby Hospital	Jodie Fitzgerald	
Derby	Royal Derby Hospital	Julie Edmonds	
Derby	London Road Community Hospital	Sarah Hathaway-Lees	
Derby	Royal Derby Hospital	Sarah Longhurst	
Derby	London Road Community Hospital	Debbie Davis	
Derby	Royal Derby Hospital	Gemma Irvine	

Derby	Royal Derby Hospital	Gemma Redfern	
Derby	London Road Community Hospital	Jane Lawrie	
Derby	Royal Derby Hospital	Julie Dockree	
Derby	London Road Community Hospital	Karen Simmonds	
Derby	Royal Derby Hospital	Karen Simmonds	
Derby	London Road Community Hospital	Kay Bowdler	
Derby	London Road Community Hospital	Kristina Duggleby	
Derby	Royal Derby Hospital	Lorraine McDonald	
Derby	Royal Derby Hospital	Mishelle Fanuncio	
Derby	London Road Community Hospital	Sarah Hare	
Derby	Royal Derby Hospital	Sarah Hare	
Derby	Royal Derby Hospital	Shobha Saravanasuthan	
Derby	Royal Derby Hospital	Sonya Bradshaw	
Derby	Royal Derby Hospital	Susan Smith	
Devon	Royal Devon and Exeter Hospital	Anna Lydon	
Doncaster	Doncaster Royal Infirmary	Jessica Tay	Co-I
Doncaster	Doncaster Royal Infirmary	Muneeb Qureshi	
Doncaster	Doncaster Royal Infirmary	Pooja Iyer	
Doncaster	Doncaster Royal Infirmary	Virgil Sivoglo	PI
Doncaster	Doncaster Royal Infirmary	Carmel Pezaro	PI
Doncaster	Doncaster Royal Infirmary	Catherine Ferguson	
Doncaster	Doncaster Royal Infirmary	Georgia Hooton	
Doncaster	Doncaster Royal Infirmary	Janet Field	
Doncaster	Doncaster Royal Infirmary	Jennifer Taylor	
Doncaster	Doncaster Royal Infirmary	Alexandra Firth	
Doncaster	Doncaster Royal Infirmary	Robert Chadwick	
Doncaster	Doncaster Royal Infirmary	Ben East	
Doncaster	Doncaster Royal Infirmary	Amy Neal	
Doncaster	Doncaster Royal Infirmary	Deborah Walstow	
Doncaster	Doncaster Royal Infirmary	Lisa Warren	
Doncaster	Doncaster Royal Infirmary	Meredyth Harris	
Doncaster	Doncaster Royal Infirmary	Nicola Wilkinson	
Doncaster	Doncaster Royal Infirmary	Nicole Jeffcutt	
Doncaster	Doncaster Royal Infirmary	Sharon Ann Allen	

Doncaster	Doncaster Royal Infirmary	Janine Smedley (nee McCabe)	
Doncaster	Doncaster Royal Infirmary	Joanne McNally	Pharmacist
Doncaster	Doncaster Royal Infirmary	Rachel Codling	
Doncaster	Doncaster Royal Infirmary	Barbara Burlace	
Doncaster	Doncaster Royal Infirmary	Joanne Derx	
Doncaster	Doncaster Royal Infirmary	Laura Ellis	
Doncaster	Doncaster Royal Infirmary	Lucy Smith	Co-I
Doncaster	Doncaster Royal Infirmary	Sarah Brown	
Doncaster	Doncaster Royal Infirmary	Kim Wood	
Dorchester	Dorset County Hospital	Benjamin Masters	PI
Dorchester	Dorset County Hospital	Perric Crellin	
Dorchester	Dorset County Hospital	Kate Taylor	
Dorchester	Dorset County Hospital	Ananda Chakrabarti	
Dorchester	Dorset County Hospital	Andrew Rees	
Dorchester	Dorset County Hospital	Naveed Afzal	
Dorchester	Dorset County Hospital	Robert Blegay	
Dorchester	Dorset County Hospital	Stephen Andrews	
Dorchester	Dorset County Hospital	Andrew Cornaby	
Dorchester	Dorset County Hospital	Andrew Gibbins	
Dorchester	Dorset County Hospital	Piet Bakker	
Dorchester	Dorset County Hospital	Simon Sharpe	
Dorchester	Dorset County Hospital	Louise O'Shea	
Dorchester	Dorset County Hospital	Sally Love	
Dorchester	Dorset County Hospital	Sarah Williams	
Dorchester	Dorset County Hospital	Susan Carr	
Dorchester	Dorset County Hospital	Jackie Gibbins	
Dorchester	Dorset County Hospital	Josie Goodsell	
Dorchester	Dorset County Hospital	Beverley Anderson	
Dorchester	Dorset County Hospital	Delia Whiteman	
Dorchester	Dorset County Hospital	Laura Bough	
Dorchester	Dorset County Hospital	Stephanie Jones	
Dorchester	Dorset County Hospital	Tracy Glen	
Dorchester	Dorset County Hospital	Lynn Billett	
Dorchester	Dorset County Hospital	Pauline Ashcroft	Pharmacist

Dorchester	Dorset County Hospital	Sally Breakspear	
Dorchester	Dorset County Hospital	Sarah Horton	
Dorchester	Dorset County Hospital	Suzy Wignall	
Duckworth Lane, Bradford	Bradford Royal Infirmary	Helen Robertshaw	
Dudley	Russells Hall Hospital	Georgi Georgiev	
Dudley	Russells Hall Hospital	Joseph Mano	Co-I
Dudley	Russells Hall Hospital	Pek Keng-Koh	PI
Dudley	Russells Hall Hospital	Syed Tirmazy	
Dudley	Russells Hall Hospital	Abel Zachariah	
Dudley	Russells Hall Hospital	Mano Joseph	Co-I
Dudley	Russells Hall Hospital	Prakash Ramachandra	
Dudley	Russells Hall Hospital	Emily McDonald	
Dudley	Russells Hall Hospital	Joann Atkinson	
Dudley	Russells Hall Hospital	Julie Matthews	
Dudley	Russells Hall Hospital	Andrew Moores	
Dudley	Russells Hall Hospital	David Edwards	
Dudley	Russells Hall Hospital	Lawrence Emtage	
Dudley	Russells Hall Hospital	Manesh Patel	
Dudley	Russells Hall Hospital	Paul Anderson	
Dudley	Russells Hall Hospital	Irene Gardner	
Dudley	Russells Hall Hospital	Vanessa Moore	Pharmacist
Dudley	Russells Hall Hospital	Ruckie Kahlon	
Dudley	Russells Hall Hospital	Anna Summerfield	
Dudley	Russells Hall Hospital	Ellen Shirley	
Dudley	Russells Hall Hospital	Heather McClure	
Dudley	Russells Hall Hospital	Karen Pearson	
Dudley	Russells Hall Hospital	Nadira Jilani	
Dudley	Russells Hall Hospital	Angela Watts	
Dudley	Russells Hall Hospital	Dee Harris	
Dudley	Russells Hall Hospital	Ellie Traverse	
Dudley	Russells Hall Hospital	Hayley Pearson	
Dudley	Russells Hall Hospital	Jayne Kanwar	
Dudley	Russells Hall Hospital	Jenny O'Grady	

Dudley	Russells Hall Hospital	Karen Kanyi	
Dudley	Russells Hall Hospital	Karen McGarry	
Dudley	Russells Hall Hospital	Lesley Edwards	
Dudley	Russells Hall Hospital	Lucie Smith (nee Williams)	Pharmacist
Dudley	Russells Hall Hospital	Sally Keates-Porter	
Dudley	Russells Hall Hospital	Sara Smith	
Dudley	Russells Hall Hospital	Kath Harrow	
Dundee	Ninewells Hospital	Sangeetha Ponnusamy	
Durham	University Hospital of North Durham	Sarah Welsh	
Durham	University Hospital of North Durham	Rhona McMenemin	
Durham	University Hospital of North Durham	Andrew Parker	
Durham	University Hospital of North Durham	Lorna Morgan	
Durham	University Hospital of North Durham	Dorothy Turnbull	
Durham	University Hospital of North Durham	Jean Dent	
Durham	University Hospital of North Durham	Jeanette Maughan	
Durham	University Hospital of North Durham	Julie Elliot	Pharmacist
Durham	University Hospital of North Durham	Julie Elliott	Pharmacist
East Bournemouth	Royal Bournemouth Hospital	Nicky Naraine	
East Sussex	Eastbourne District General Hospital	Graham Watson	
Eastbourne	Eastbourne District General Hospital	Aspasia Soultati	
Eastbourne	Conquest Hospital	Caroline Manetta	PI
Eastbourne	Eastbourne District General Hospital	Caroline Manetta	PI
Eastbourne	Conquest Hospital	Duncan Gilbert	
Eastbourne	Eastbourne District General Hospital	Duncan Gilbert	
Eastbourne	Eastbourne District General Hospital	Fiona McKinna	
Eastbourne	Eastbourne District General Hospital	Peter Rimington	
Eastbourne	Eastbourne District General Hospital	David Sharp	
Eastbourne	Eastbourne District General Hospital	Mark Whitfield	
Eastbourne	Eastbourne District General Hospital	Neville Sharma	Pharmacist
Eastbourne	Eastbourne District General Hospital	William Lawrence	
Eastbourne	Eastbourne District General Hospital	Jo-Anne Taylor	
Eastbourne	Eastbourne District General Hospital	Kay Jones-Skipper	
Eastbourne	Eastbourne District General Hospital	Angie Bowey	
Eastbourne	Eastbourne District General Hospital	Lauren McCrisken	

Eastbourne	Eastbourne District General Hospital	Prudence Hobbs	Pharmacist
Eastbourne	Eastbourne District General Hospital	Shelley Baumber	
Eastbourne	Eastbourne District General Hospital	Theresa Baumber	
Eastbourne	Eastbourne District General Hospital	Joanna Howard	
Edinburgh	Western General Hospital	Alistair Law	Co-I
Edinburgh	Western General Hospital	Amy Cooper	
Edinburgh	Western General Hospital	Archie Macnar	
Edinburgh	Western General Hospital	Caroline Bruce	
Edinburgh	Western General Hospital	Mark Stares	
Edinburgh	Western General Hospital	Martin Doak	
Edinburgh	Western General Hospital	Olvsola Faluyi	
Edinburgh	Western General Hospital	Claire Arthur	
Edinburgh	Western General Hospital	Duncan McLaren	PI
Edinburgh	Western General Hospital	Ewan Brown	
Edinburgh	Western General Hospital	Grahame Howard	
Edinburgh	Western General Hospital	Hannah Lord	
Edinburgh	Western General Hospital	John McGrane	
Edinburgh	Western General Hospital	Katie Wood	
Edinburgh	Western General Hospital	Sanjana Masinghe	
Edinburgh	Western General Hospital	Emma Lewis	
Edinburgh	Western General Hospital	Heather Howie	
Edinburgh	Western General Hospital	Nikki Gilluley	Pharmacist
Edinburgh	Western General Hospital	Ben Elliott	Pharmacist
Edinburgh	Western General Hospital	Jahangeer Malik	Co-I
Edinburgh	Western General Hospital	Richard Allan	
Edinburgh	Western General Hospital	Roland Donat	
Edinburgh	Western General Hospital	Alan McNeill	
Edinburgh	Western General Hospital	Brian Rogers	
Edinburgh	Western General Hospital	David Jeffrey	
Edinburgh	Western General Hospital	David Tulloch	
Edinburgh	Western General Hospital	Prasad Bollina	
Edinburgh	Western General Hospital	Lynn Ho	
Edinburgh	Western General Hospital	Vivienne Wilson	
Edinburgh	Western General Hospital	Barbara Mayne	

Edinburgh	Western General Hospital	Alison Clark	
Edinburgh	Western General Hospital	Alison McKinlay	
Edinburgh	Western General Hospital	Beverley Mitchell	
Edinburgh	Western General Hospital	Catherine Woods	
Edinburgh	Western General Hospital	Jennifer Baxter	
Edinburgh	Western General Hospital	Kirsty Peebles	
Edinburgh	Western General Hospital	Sarah Thompson	
Edinburgh	Western General Hospital	Tracy Brear	
Edinburgh	Western General Hospital	Ailsa Liddle	
Edinburgh	Western General Hospital	Andrea Stanton	
Edinburgh	Western General Hospital	Ann Cochrane	
Edinburgh	Western General Hospital	Fiona Gardiner	
Edinburgh	Western General Hospital	Fionagh Ross	Pharmacist
Edinburgh	Western General Hospital	Hazel Milligan	
Edinburgh	Western General Hospital	Heather Dalrymple	
Edinburgh	Western General Hospital	Heather McVicars	
Edinburgh	Western General Hospital	Lisa Egan	
Edinburgh	Western General Hospital	Lois Pollock	
Edinburgh	Western General Hospital	Susan Forman	
Edinburgh	Western General Hospital	Theresa Savage	
Edmonton	North Middlesex Hospital	Anna Thompson	
Edmonton	North Middlesex Hospital	Lucinda Melcher	
Edmonton	North Middlesex Hospital	Mausam Singhera	
Edmonton	North Middlesex Hospital	Nishi Gupta	PI
Edmonton	North Middlesex Hospital	Stephen Karp	
Edmonton	North Middlesex Hospital	Ursula McGovern	
Edmonton	North Middlesex Hospital	Ayesha Ahmed Surti	
Edmonton	North Middlesex Hospital	Chloe Van Someren	
Edmonton	North Middlesex Hospital	Sagal Kullane	
Edmonton	North Middlesex Hospital	Girish Bhome	Pharmacist
Edmonton	North Middlesex Hospital	Tom Caumont	
Edmonton	North Middlesex Hospital	Tessa Light	
Edmonton	North Middlesex Hospital	Kerri Rees	
Edmonton	North Middlesex Hospital	Beatrice Balachandran	

Edmonton	North Middlesex Hospital	Bernadette Collins	
Edmonton	North Middlesex Hospital	Ferrial Syed	
Edmonton	North Middlesex Hospital	Kathy O'Farrell	
Edmonton	North Middlesex Hospital	Pauline Lee	
Exeter	Royal Devon and Exeter Hospital	Ayman Nassar	
Exeter	Royal Devon and Exeter Hospital	David Jonathan Chambers	
Exeter	Royal Devon and Exeter Hospital	Lyndon Ridges-Jones	Co-I
Exeter	Royal Devon and Exeter Hospital	Mohini Varughese	PI
Exeter	Royal Devon and Exeter Hospital	Natalie Nityey	
Exeter	Royal Devon and Exeter Hospital	Peter Stephens	Co-I
Exeter	Royal Devon and Exeter Hospital	Shiv Uppal	
Exeter	Royal Devon and Exeter Hospital	Victoria Ford	Co-I
Exeter	Royal Devon and Exeter Hospital	Anne Hong	
Exeter	Royal Devon and Exeter Hospital	Denise Sheehan	
Exeter	Royal Devon and Exeter Hospital	Elizabeth Toy	
Exeter	Royal Devon and Exeter Hospital	Rajaguru Srinivasan	Co-I
Exeter	Royal Devon and Exeter Hospital	San Aung	Co-I
Exeter	Royal Devon and Exeter Hospital	Tim Norris	
Exeter	Royal Devon and Exeter Hospital	Ceri Davies	
Exeter	Royal Devon and Exeter Hospital	Eleonor (Alethea) Brown	
Exeter	Royal Devon and Exeter Hospital	Grace Justice	
Exeter	Royal Devon and Exeter Hospital	Kerri-ellen Oakley	
Exeter	Royal Devon and Exeter Hospital	Sophie James	
Exeter	Royal Devon and Exeter Hospital	Stephanie Ann Ellis	
Exeter	Royal Devon and Exeter Hospital	Theresa Lawless	
Exeter	Royal Devon and Exeter Hospital	Susan Downer	
Exeter	Royal Devon and Exeter Hospital	Alan Betts	
Exeter	Royal Devon and Exeter Hospital	James Leavy	
Exeter	Royal Devon and Exeter Hospital	Matt Trivett	
Exeter	Royal Devon and Exeter Hospital	Petar Hitev	
Exeter	Royal Devon and Exeter Hospital	Christoph Lohan	
Exeter	Royal Devon and Exeter Hospital	John Anderson	
Exeter	Royal Devon and Exeter Hospital	Ross Curwen	
Exeter	Royal Devon and Exeter Hospital	Elaine Vandcandelaere	

Exeter	Royal Devon and Exeter Hospital	Emma Guerin	
Exeter	Royal Devon and Exeter Hospital	Frances Hood	
Exeter	Royal Devon and Exeter Hospital	Melissa Davey	
Exeter	Royal Devon and Exeter Hospital	Alison Augstburger	
Exeter	Royal Devon and Exeter Hospital	Claire Webb	
Exeter	Royal Devon and Exeter Hospital	Emma Robjohns	
Exeter	Royal Devon and Exeter Hospital	Ingrid Seath	
Exeter	Royal Devon and Exeter Hospital	Kate O'Connor	
Exeter	Royal Devon and Exeter Hospital	Alison Roantree	
Exeter	Royal Devon and Exeter Hospital	Jane Piper	
Exeter	Royal Devon and Exeter Hospital	Rosie Mew	Co-I
Exeter	Royal Devon and Exeter Hospital	Sophie Warren	
Exeter	Royal Devon and Exeter Hospital	Susan Davenport	Pharmacist
Exeter	Royal Devon and Exeter Hospital	Tracey Foss	
Exeter	Royal Devon and Exeter Hospital	Beverley Kemp	
Exeter	Royal Devon and Exeter Hospital	Claire Ridler	
Exeter	Royal Devon and Exeter Hospital	Dawn Edwards	
Exeter	Royal Devon and Exeter Hospital	Elizabeth Davey	
Exeter	Royal Devon and Exeter Hospital	Fiona Walters (nee Hall)	Pharmacist
Exeter	Royal Devon and Exeter Hospital	Kizzy Baines	
Exeter	Royal Devon and Exeter Hospital	Lyndel Moore	
Exeter	Royal Devon and Exeter Hospital	Shirley Todd	Pharmacist
Exeter	Royal Devon and Exeter Hospital	Suzy Tasker	
Exeter	Royal Devon and Exeter Hospital	Tamika Chapter	Pharmacist
Fulwood	Blackburn Royal Infirmary	Natalie Charnley	
Fulwood	Burnley General Hospital	Natalie Charnley	
Fulwood	Royal Preston Hospital	Hazel Aston	
Gillingham	Medway Maritime Hospital	Afroditi Karathanasi	
Gillingham	Medway Maritime Hospital	Diletta Bianchini	
Gillingham	Medway Maritime Hospital	Stergios Boussios	PI
Gillingham	Medway Maritime Hospital	Swapna Thomas	
Gillingham	Medway Maritime Hospital	Charlotte Abson	Co-I
Gillingham	Medway Maritime Hospital	Christos Mikropoulos	Co-I
Gillingham	Medway Maritime Hospital	Henry Taylor	PI

Gillingham	Medway Maritime Hospital	Tessa Lawrence	
Gillingham	Medway Maritime Hospital	Agne Sadauskaite	
Gillingham	Medway Maritime Hospital	Corinne Borley	
Gillingham	Medway Maritime Hospital	Durga Maya Gurung	
Gillingham	Medway Maritime Hospital	Jodie Seymour	
Gillingham	Medway Maritime Hospital	Mary Everett	
Gillingham	Medway Maritime Hospital	Charles Davis	
Gillingham	Medway Maritime Hospital	James Sawyer	Pharmacist
Gillingham	Medway Maritime Hospital	Kevin Naicker	
Gillingham	Medway Maritime Hospital	Khalid Abdalla	Pharmacist
Gillingham	Medway Maritime Hospital	Peter Milverton	
Gillingham	Medway Maritime Hospital	Philip Adeniran	
Gillingham	Medway Maritime Hospital	Simon Wan	
Gillingham	Medway Maritime Hospital	Parool Darbar	Pharmacist
Gillingham	Medway Maritime Hospital	Richard Thornton	
Gillingham	Medway Maritime Hospital	Alba Tuban	
Gillingham	Medway Maritime Hospital	Alison Richards	
Gillingham	Medway Maritime Hospital	Clarissa Madla	
Gillingham	Medway Maritime Hospital	Deirdre Cooke	
Gillingham	Medway Maritime Hospital	Elizabeth Newman-Horne	
Gillingham	Medway Maritime Hospital	Gayzel Vallejera	
Gillingham	Medway Maritime Hospital	Katarzyna Urbanczyk	
Gillingham	Medway Maritime Hospital	Kay Jones	
Gillingham	Medway Maritime Hospital	Lisa Parker	
Gillingham	Medway Maritime Hospital	Louise Black	
Gillingham	Medway Maritime Hospital	Louise Brassington	
Gillingham	Medway Maritime Hospital	Marie Louise Hollands	
Gillingham	Medway Maritime Hospital	Nicola Southwell	
Gillingham	Medway Maritime Hospital	Emma Sutton	
Gillingham	Medway Maritime Hospital	Judy Filmer	
Gillingham	Medway Maritime Hospital	Suzie Reyner	
Gillingham	Medway Maritime Hospital	Adedolapo Sanni	
Gillingham	Medway Maritime Hospital	Carol Mayger	Pharmacist
Gillingham	Medway Maritime Hospital	Tamara Diamond	

Glasgow	Beatson West of Scotland Cancer Centre	Aisha Tufail	
Glasgow	Beatson West of Scotland Cancer Centre	Almudena Cascales	
Glasgow	Beatson West of Scotland Cancer Centre	Ashleigh Kerr	
Glasgow	Beatson West of Scotland Cancer Centre	Brendan McCann	
Glasgow	Beatson West of Scotland Cancer Centre	Cicely Cunningham	
Glasgow	Beatson West of Scotland Cancer Centre	Derek Grose	Co-I
Glasgow	Beatson West of Scotland Cancer Centre	Ian Sanders	
Glasgow	Beatson West of Scotland Cancer Centre	Jawaher Ansari	
Glasgow	Beatson West of Scotland Cancer Centre	Kathryn Banfill	Co-I
Glasgow	Beatson West of Scotland Cancer Centre	Miranda Ashton	
Glasgow	Beatson West of Scotland Cancer Centre	Rebecca Muirhead	
Glasgow	Beatson West of Scotland Cancer Centre	Stephen McKay	
Glasgow	Beatson West of Scotland Cancer Centre	Abdulla Al-hasso	
Glasgow	Beatson West of Scotland Cancer Centre	Aqilah Othman	
Glasgow	Beatson West of Scotland Cancer Centre	Awris Jalil	
Glasgow	Beatson West of Scotland Cancer Centre	Azmat Sadozye	
Glasgow	Beatson West of Scotland Cancer Centre	Balaji Venugopal	
Glasgow	Beatson West of Scotland Cancer Centre	Ben Fulton	
Glasgow	Beatson West of Scotland Cancer Centre	Carolynn Lamb	Co-I
Glasgow	Beatson West of Scotland Cancer Centre	Christina Wilson	
Glasgow	Beatson West of Scotland Cancer Centre	David Dodds	
Glasgow	Beatson West of Scotland Cancer Centre	Esfandiyar Khan	
Glasgow	Beatson West of Scotland Cancer Centre	Hilary Glen	
Glasgow	Beatson West of Scotland Cancer Centre	Husam Marashi	
Glasgow	Beatson West of Scotland Cancer Centre	Jan Wallace	
Glasgow	Beatson West of Scotland Cancer Centre	Janet Graham	
Glasgow	Beatson West of Scotland Cancer Centre	John Graham	PI
Glasgow	Beatson West of Scotland Cancer Centre	Kathryn Graham	
Glasgow	Beatson West of Scotland Cancer Centre	Lye Mun Tho	
Glasgow	Beatson West of Scotland Cancer Centre	Martin Russell	
Glasgow	Beatson West of Scotland Cancer Centre	Maryon Hardie	
Glasgow	Beatson West of Scotland Cancer Centre	Mohammed Alfayez	
Glasgow	Beatson West of Scotland Cancer Centre	Nicholas Macleod	
Glasgow	Beatson West of Scotland Cancer Centre	Norma Sidek	

Glasgow	Beatson West of Scotland Cancer Centre	Patricia Roxburgh	
Glasgow	Beatson West of Scotland Cancer Centre	Paula Henry-Stephenson	
Glasgow	Beatson West of Scotland Cancer Centre	Rana Mahmood	
Glasgow	Beatson West of Scotland Cancer Centre	Rob Jones	PI
Glasgow	Beatson West of Scotland Cancer Centre	Sally Hall	
Glasgow	Beatson West of Scotland Cancer Centre	Saranya Kakumanu	
Glasgow	Beatson West of Scotland Cancer Centre	Sophie Barrett	
Glasgow	Beatson West of Scotland Cancer Centre	Stefan Nowicki	
Glasgow	Beatson West of Scotland Cancer Centre	Tareq Abdullah	
Glasgow	Beatson West of Scotland Cancer Centre	Diann Taggart	
Glasgow	Beatson West of Scotland Cancer Centre	Katie Galbraith	
Glasgow	Beatson West of Scotland Cancer Centre	Nicola Cairns	
Glasgow	Beatson West of Scotland Cancer Centre	Gerard Forrest	
Glasgow	Beatson West of Scotland Cancer Centre	Calum Innes	
Glasgow	Beatson West of Scotland Cancer Centre	Graeme Lumsden	
Glasgow	Beatson West of Scotland Cancer Centre	Martin Ball	Pharmacist
Glasgow	Beatson West of Scotland Cancer Centre	Nathan Richardson	
Glasgow	Beatson West of Scotland Cancer Centre	Ross Carruthers	
Glasgow	Beatson West of Scotland Cancer Centre	Hannah Weir	
Glasgow	Beatson West of Scotland Cancer Centre	Linzi Rae	
Glasgow	Beatson West of Scotland Cancer Centre	Maureen Connolly	
Glasgow	Beatson West of Scotland Cancer Centre	Ailsa Griffen	
Glasgow	Beatson West of Scotland Cancer Centre	Jacqueline Gourlay	Pharmacist
Glasgow	Beatson West of Scotland Cancer Centre	Jan Graham	
Glasgow	Beatson West of Scotland Cancer Centre	Karen Bell	
Glasgow	Beatson West of Scotland Cancer Centre	Alice Coy	
Glasgow	Beatson West of Scotland Cancer Centre	Gemma Johnson	
Glasgow	Beatson West of Scotland Cancer Centre	Gillian Barmack	
Glasgow	Beatson West of Scotland Cancer Centre	Jennifer Petrie	
Glasgow	Beatson West of Scotland Cancer Centre	Annette Charlick	
Glasgow	Beatson West of Scotland Cancer Centre	Antonia MacMillan	
Glasgow	Beatson West of Scotland Cancer Centre	Catriona Cowan	
Glasgow	Beatson West of Scotland Cancer Centre	Claire Steele	
Glasgow	Beatson West of Scotland Cancer Centre	Fiona McQueen	Pharmacist

Glasgow	Beatson West of Scotland Cancer Centre	Jenny Brown	Pharmacist
Glasgow	Beatson West of Scotland Cancer Centre	Judith Dixon	
Glasgow	Beatson West of Scotland Cancer Centre	Kirsteen Stuart	
Glasgow	Beatson West of Scotland Cancer Centre	Kirsten Laws (nee Borthwick)	
Glasgow	Beatson West of Scotland Cancer Centre	Lorraine Barwell	
Glasgow	Beatson West of Scotland Cancer Centre	Louise Bruce	
Glasgow	Beatson West of Scotland Cancer Centre	Lynne Grieve	
Glasgow	Beatson West of Scotland Cancer Centre	Maria Nicol	
Glasgow	Beatson West of Scotland Cancer Centre	Patricia Baird	
Glasgow	Beatson West of Scotland Cancer Centre	Ruth Orr	
Glasgow	Beatson West of Scotland Cancer Centre	Sai Juan Jia	
Gloucester	Gloucestershire Royal Hospital	Laura Malins	
Gloucester	Gloucestershire Royal Hospital	Sai Jonnada	
Gloucester	Gloucestershire Royal Hospital	Victoria Bell	
Gloucester	Gloucestershire Royal Hospital	Audrey Cook	
Gloucester	Gloucestershire Royal Hospital	Jo Bowen	PI
Gloucester	Gloucestershire Royal Hospital	Peter Jenkins	Co-I
Gloucester	Gloucestershire Royal Hospital	Roger Owen	
Gloucester	Gloucestershire Royal Hospital	Amy Skelton	
Gloucester	Gloucestershire Royal Hospital	Bethan Cartwright	
Gloucester	Gloucestershire Royal Hospital	Sarah Stanley	
Gloucester	Gloucestershire Royal Hospital	Sarah Beazer	
Gloucester	Gloucestershire Royal Hospital	Samuel Croly	
Gloucester	Gloucestershire Royal Hospital	Richard Wallis	
Gloucester	Gloucestershire Royal Hospital	Julia Hall	
Gloucester	Gloucestershire Royal Hospital	Julie Allen	
Gloucester	Gloucestershire Royal Hospital	Lin Crossley	
Gloucester	Gloucestershire Royal Hospital	Rachel Sayers	
Gloucester	Gloucestershire Royal Hospital	Chris Ford	
Gloucester	Gloucestershire Royal Hospital	Kate Trigg-Hogarth	
Gloucester	Gloucestershire Royal Hospital	Sue Wronski	
Gloucester	Gloucestershire Royal Hospital	Abi Stuart	
Gloucester	Gloucestershire Royal Hospital	Charlotte Ayrton	
Gloucester	Gloucestershire Royal Hospital	Claire Salter	

Gloucester	Gloucestershire Royal Hospital	Elaine Sizer	
Gloucester	Gloucestershire Royal Hospital	Elisabeth Read	
Gloucester	Gloucestershire Royal Hospital	Louise Moore	
Gloucester	Gloucestershire Royal Hospital	Sarah Matthews	
Glouster	Gloucestershire Royal Hospital	Janet Forkes	
Guildford	Royal Surrey County Hospital	Carla Perna	PI
Guildford	Royal Surrey County Hospital	Emmanuel Larbi	
Guildford	Royal Surrey County Hospital	James Lowe	
Guildford	Royal Surrey County Hospital	Leslie Cheng	Co-I
Guildford	Royal Surrey County Hospital	Mahwish Karim	Co-I
Guildford	Royal Surrey County Hospital	Melanie Boafo-Yirenkyi	
Guildford	Royal Surrey County Hospital	Sara Khaksar	
Guildford	Royal Surrey County Hospital	Charlotte Shelley	
Guildford	Royal Surrey County Hospital	Jenny Nobes	
Guildford	Royal Surrey County Hospital	Joanna Stokoe	
Guildford	Royal Surrey County Hospital	Julian Money-Kyrle	
Guildford	Royal Surrey County Hospital	Katie Wood	
Guildford	Royal Surrey County Hospital	Katie Wood	
Guildford	Royal Surrey County Hospital	Mahomed Moosa	
Guildford	Royal Surrey County Hospital	Maria Drzymala	
Guildford	Royal Surrey County Hospital	Richard Shaffer	
Guildford	Royal Surrey County Hospital	Robert Laing	
Guildford	Royal Surrey County Hospital	Sree Susaria	
Guildford	Royal Surrey County Hospital	Teresa Guerrero-Urbano	
Guildford	Royal Surrey County Hospital	Imogen Heenan	
Guildford	Royal Surrey County Hospital	Kavita Bhat	
Guildford	Royal Surrey County Hospital	Lesley Harden	
Guildford	Royal Surrey County Hospital	Nick Pilkington	
Guildford	Royal Surrey County Hospital	Richmond Abeseabe	
Guildford	Royal Surrey County Hospital	Angela Morgan	
Guildford	Royal Surrey County Hospital	Miriam White	
Guildford	Royal Surrey County Hospital	Adele Hugg	
Guildford	Royal Surrey County Hospital	Caterina Bissa	
Guildford	Royal Surrey County Hospital	Jane Woods	

Guildford	Royal Surrey County Hospital	Julie Wilkinson	
Guildford	Royal Surrey County Hospital	Veronica Davis	
Guildford	Royal Surrey County Hospital	Frances Sidi	
Guildford	Royal Surrey County Hospital	Jen Julius	
Guildford	Royal Surrey County Hospital	Julia Whittle	
Guildford	Royal Surrey County Hospital	Kate Penhaligon	
Guildford	Royal Surrey County Hospital	Kathrin Narvaez-Vega	Pharmacist
Guildford	Royal Surrey County Hospital	Lucinda Scott	
Guildford	Royal Surrey County Hospital	Marianne Dabbs	
Guildford	Royal Surrey County Hospital	Stephy Joseph	
Guildford	Royal Surrey County Hospital	Catherine Medcalf	Pharmacist
Guildford	Royal Surrey County Hospital	Celia Harris	
Guildford	Royal Surrey County Hospital	Daisy Floyd	
Guildford	Royal Surrey County Hospital	Fiona Butler	Pharmacist
Guildford	Royal Surrey County Hospital	Linda Nardone	
Guildford	Royal Surrey County Hospital	Sarah De Swert	
Guildford	Royal Surrey County Hospital	Sue Sargent	
Guildford	Royal Surrey County Hospital	Teresa Keating	
Guildford	Royal Surrey County Hospital	Zephyrine King	
Halifax	Calderdale Royal Hospital	Miranda Usher	
Halifax	Calderdale Royal Hospital	Lisa Gledhill	
Halton	Halton Hospital	Carrie Lowthian	
Hampstead	Royal Free Hospital	R Bradford	
Harlow	Princess Alexandra Hospital (Harlow)	Albert Edwards	
Harlow	Princess Alexandra Hospital (Harlow)	Hamoun Rozati	Co-I
Harlow	Princess Alexandra Hospital (Harlow)	Paul Kabuubi	
Harlow	Princess Alexandra Hospital (Harlow)	Shroma De Silva	
Harlow	Princess Alexandra Hospital (Harlow)	Tasia Aghadiuno	Co-I
Harlow	Princess Alexandra Hospital (Harlow)	Zainab Wasim	Co-I
Harlow	Princess Alexandra Hospital (Harlow)	Anna Lerner	Co-I
Harlow	Princess Alexandra Hospital (Harlow)	Lucinda Melcher	PI
Harlow	Princess Alexandra Hospital (Harlow)	Nishi Gupta	Co-I
Harlow	Princess Alexandra Hospital (Harlow)	Reena Davda	
Harlow	Princess Alexandra Hospital (Harlow)	Reena Davda	Co-I

Harlow	Princess Alexandra Hospital (Harlow)	Nikki White (nee Staines)	
Harlow	Princess Alexandra Hospital (Harlow)	Sylwia Goliaszewska	
Harlow	Princess Alexandra Hospital (Harlow)	Ahmed Hnoosh	
Harlow	Princess Alexandra Hospital (Harlow)	Ervin Shpuza	
Harlow	Princess Alexandra Hospital (Harlow)	Sunjalee Fernando	Pharmacist
Harlow	Princess Alexandra Hospital (Harlow)	Cait Rees	
Harlow	Princess Alexandra Hospital (Harlow)	Amanda Lewis	
Harlow	Princess Alexandra Hospital (Harlow)	Amelia Daniel	
Harlow	Princess Alexandra Hospital (Harlow)	Amy Lewis	
Harlow	Princess Alexandra Hospital (Harlow)	Gemma Cook	
Harlow	Princess Alexandra Hospital (Harlow)	Hana Malinkovicova	
Harlow	Princess Alexandra Hospital (Harlow)	Joanne Kellaway	
Harlow	Princess Alexandra Hospital (Harlow)	Tracey White	
Harlow	Princess Alexandra Hospital (Harlow)	Evelyn Holmes	Pharmacist
Harlow	Princess Alexandra Hospital (Harlow)	Jodie Johnson	
Harlow	Princess Alexandra Hospital (Harlow)	Teresa Light	
Haverford West	Withybush General Hospital	Sandra Griffiths nee Evens	
Headington	Churchill Hospital	James Wakelin	
Headington	Churchill Hospital	Jane Gibbard	
Headington	Churchill Hospital	Leigh Burns	
Headington	Churchill Hospital	Sandie Wellman	
Hereford	Hereford County Hospital	Caitlin Bowden	
Hereford	Hereford County Hospital	Cara Watson	
Hereford	Hereford County Hospital	David Stow	
Hereford	Hereford County Hospital	Timothy Spencer	
Hereford	Hereford County Hospital	Vishal Bhalla	
Hereford	Hereford County Hospital	Warren Grant	PI
Hereford	Hereford County Hospital	Audrey Cook	
Hereford	Hereford County Hospital	Duncan Stow	
Hereford	Hereford County Hospital	Maxine Flubacher	
Hereford	Hereford County Hospital	Nina Reeve	
Hereford	Hereford County Hospital	Bethan Richards	
Hereford	Hereford County Hospital	Jolanta Pueskacz	
Hereford	Hereford County Hospital	Linda Moseley	Pharmacist

Hereford	Hereford County Hospital	Lisa King	
Hereford	Hereford County Hospital	Nicola Williamson	
Hereford	Hereford County Hospital	Sarah Chapman	
Hereford	Hereford County Hospital	Serrafina Carini	
Hereford	Hereford County Hospital	Sophie Cooper	
Hereford	Hereford County Hospital	Stacey Turner	
Hereford	Hereford County Hospital	Harriet Taylor	
Hereford	Hereford County Hospital	Jagdish Chana	Pharmacist
Hereford	Hereford County Hospital	Terry Watson	
Hereford	Hereford County Hospital	Andy Hedges	Pharmacist
Hereford	Hereford County Hospital	Amanda Davies	
Hereford	Hereford County Hospital	Isabel Martin (Whitehouse)	
Hereford	Hereford County Hospital	June Thomas	
Hereford	Hereford County Hospital	Laura Lees	
Hereford	Hereford County Hospital	Melanie Evans	
Hereford	Hereford County Hospital	Rebecca Bengree	
Hereford	Hereford County Hospital	Susan Anderson	
Hereford	Hereford County Hospital	Zara Roberts	
Hereford	Hereford County Hospital	Bethany Wellington	
Hereford	Hereford County Hospital	Janine Jones (Birch)	
Hereford	Hereford County Hospital	Naeem Musani	
Hereford	Hereford County Hospital	Susan Anderson	
Hereford	Hereford County Hospital	Caroline Thomas	Pharmacist
Hereford	Hereford County Hospital	Catherine Reed	
Hereford	Hereford County Hospital	Claire Hughes	
Hereford	Hereford County Hospital	Gill Horsfield	
Hereford	Hereford County Hospital	Jenny Howls	Pharmacist
Hereford	Hereford County Hospital	Kate Hammerton	
Hereford	Hereford County Hospital	Rachel Lowe	
Hereford	Hereford County Hospital	Sophie Boyd (nee Evans)	
Hereford	Hereford County Hospital	Anita Ashton	
Hereford	Hereford County Hospital	Janet Forkes	
Hereford	Hereford County Hospital	Sophie Boyd	
Herts	Lister Hospital	Rachel Low	

High Wycombe	Wycombe Hospital	Ami Sabharwal	PI
High Wycombe	Wycombe Hospital	Avinash Gupta	
High Wycombe	Wycombe Hospital	Janice Carpenter	
High Wycombe	Wycombe Hospital	Katherine Hyde	PI
High Wycombe	Wycombe Hospital	Thinn Pwint	Co-I
High Wycombe	Wycombe Hospital	Benjamin Fairfax	
High Wycombe	Wycombe Hospital	Gerard Andrade	Co-I
High Wycombe	Wycombe Hospital	Niki Panakis	
High Wycombe	Wycombe Hospital	Philip Camilleri	Co-I
High Wycombe	Wycombe Hospital	Prabir Chakraborti	Co-I
High Wycombe	Wycombe Hospital	Sally Trent	
High Wycombe	Wycombe Hospital	Sean O'Cathail	Co-I
High Wycombe	Wycombe Hospital	Vivek Mohan	
High Wycombe	Wycombe Hospital	Wasiru Saka	
High Wycombe	Stoke Mandeville Hospital	Chrissie Butcher	
High Wycombe	Wycombe Hospital	Chrissie Butcher	
High Wycombe	Wycombe Hospital	Claire Fernandez	
High Wycombe	Wycombe Hospital	Amarjit Bdesha	
High Wycombe	Wycombe Hospital	Moncy Mathew	
High Wycombe	Wycombe Hospital	Neil Haldar	
High Wycombe	Wycombe Hospital	Rahul Kurup	
High Wycombe	Wycombe Hospital	John Patrick Kelleher	
High Wycombe	Wycombe Hospital	Neil Trew-Smith	
High Wycombe	Wycombe Hospital	Alice Ngumo	
High Wycombe	Wycombe Hospital	Anita Cserbane	
High Wycombe	Wycombe Hospital	Gail Varley	
High Wycombe	Wycombe Hospital	Janet Weir	
High Wycombe	Wycombe Hospital	Manisha Joshi	
High Wycombe	Wycombe Hospital	Penny Carter	
High Wycombe	Wycombe Hospital	Siobhan Gettings	
High Wycombe	Wycombe Hospital	Maggie Aldersley	
High Wycombe	Wycombe Hospital	Michelle Taylor-Siddons	Pharmacist
High Wycombe	Wycombe Hospital	Tracey Stammers	
High Wycombe	Wycombe Hospital	Ans-Mari Bester	

High Wycombe	Wycombe Hospital	Aruna Nair	
High Wycombe	Wycombe Hospital	Catherine Northey	
High Wycombe	Wycombe Hospital	Emma Hogbin	
High Wycombe	Wycombe Hospital	Erica Lieberman	Pharmacist
High Wycombe	Wycombe Hospital	Helena Stone	
High Wycombe	Wycombe Hospital	Kathryn Herbert	
High Wycombe	Wycombe Hospital	Rossana Mancinelli	
High Wycombe	Wycombe Hospital	Samantha Thomas	
High Wycombe	Wycombe Hospital	Sarah Manyangadze	
High Wycombe	Wycombe Hospital	Susan McLain-Smith	
High Wycombe	Wycombe Hospital	Tiffany Chan	Pharmacist
High Wycombe	Stoke Mandeville Hospital	Bhavna Badiani	
High Wycombe	Wycombe Hospital	Bhavna Badiani	
High Wycombe	Wycombe Hospital	Christine Collins	
High Wycombe	Wycombe Hospital	Evelyn Chan	
High Wycombe	Wycombe Hospital	Hazel Wynn	
High Wycombe	Wycombe Hospital	Ileana Nguyen	
High Wycombe	Wycombe Hospital	Jasvinder Bains	
High Wycombe	Wycombe Hospital	Nicola Bowers	
High Wycombe	Wycombe Hospital	Roisin Kavanagh	
High Wycombe	Wycombe Hospital	Andrew Protheroe	
Huddersfield	Huddersfield Royal Infirmary	Omer Babiker	Co-I
Huddersfield	Huddersfield Royal Infirmary	Rob Turner	
Huddersfield	Huddersfield Royal Infirmary	Samantha Turnbull	Co-I
Huddersfield	Huddersfield Royal Infirmary	Barbara Crosse	
Huddersfield	Huddersfield Royal Infirmary	Emma Woodward	
Huddersfield	Huddersfield Royal Infirmary	Jane Hook	
Huddersfield	Huddersfield Royal Infirmary	Uschi Hofmann	PI
Huddersfield	Huddersfield Royal Infirmary	Hannah Riley	
Huddersfield	Huddersfield Royal Infirmary	Sanya Anjum	
Huddersfield	Huddersfield Royal Infirmary	Sarah Hanley	
Huddersfield	Huddersfield Royal Infirmary	Adam Mawer	Pharmacist
Huddersfield	Huddersfield Royal Infirmary	Deivasikamani Ramanujam	Co-I
Huddersfield	Huddersfield Royal Infirmary	Mohammad Irfan Alam	

Huddersfield	Huddersfield Royal Infirmary	Nicolas Bryan	Co-I
Huddersfield	Huddersfield Royal Infirmary	Hayley Webster	Pharmacist
Huddersfield	Huddersfield Royal Infirmary	Julie Millward	
Huddersfield	Huddersfield Royal Infirmary	Kathryn Smith	
Huddersfield	Huddersfield Royal Infirmary	Kully Sandhu	
Huddersfield	Huddersfield Royal Infirmary	Lear Matapure	
Huddersfield	Huddersfield Royal Infirmary	Lee-Ann Bayo	
Huddersfield	Huddersfield Royal Infirmary	Melanie Quesne	
Huddersfield	Huddersfield Royal Infirmary	Nicky Daker	
Huddersfield	Huddersfield Royal Infirmary	Hayley Inman	
Huddersfield	Huddersfield Royal Infirmary	Lisa Shaw	
Huddersfield	Huddersfield Royal Infirmary	Miranda Usher	
Huddersfield	Huddersfield Royal Infirmary	Belinda McLean	
Huddersfield	Huddersfield Royal Infirmary	Diane Kelly	
Huddersfield	Huddersfield Royal Infirmary	Karen Bicknell	
Huddersfield	Huddersfield Royal Infirmary	Katherine Tighe	
Huddersfield	Huddersfield Royal Infirmary	Lindsay Greenhalgh	Pharmacist
Huddersfield	Huddersfield Royal Infirmary	Lucy Jones	Co-I
Huddersfield	Huddersfield Royal Infirmary	Monica Narasimham	
Huddersfield	Huddersfield Royal Infirmary	Naledi Mzwimbi	
Huddersfield	Huddersfield Royal Infirmary	Paula Gomes	Pharmacist
Huddersfield	Huddersfield Royal Infirmary	Christine Turner	
Huddersfield	Huddersfield Royal Infirmary	Denise Hancock	
Huddersfield	Huddersfield Royal Infirmary	Lisa Gledhill	
Huddersfield	Huddersfield Royal Infirmary	Mandy Madigan	
Huddersfield	Huddersfield Royal Infirmary	Rachel Parker	
Huddersfield	Huddersfield Royal Infirmary	Sharon Woolley	
Huddersfield	Huddersfield Royal Infirmary	Stacey Freeth	
Huddersfield	Huddersfield Royal Infirmary	Tracy Wood	
Huddersfield	Huddersfield Royal Infirmary	Ibrar Hussain	
Hull	Princess Royal Hospital (Hull)	Christopher Hamilton	
Hull	Princess Royal Hospital (Hull)	Robert Dealey	PI
Hull	Princess Royal Hospital (Hull)	Emma Bertram	Pharmacist
Hull	Princess Royal Hospital (Hull)	Sarah Moffat	

Hull	Princess Royal Hospital (Hull)	Suzy Bunton	
Hull	Princess Royal Hospital (Hull)	Claire Levesley	
Hull	Princess Royal Hospital (Hull)	Linda Hoggarth	
Ilford	King George Hospital	Ramachandran Subramaniam	
Ilford	King George Hospital	Neil Fisher	
Inverness	Raigmore Hospital	Alison Nicholls	
Inverness	Raigmore Hospital	Anne Marie Pollock	
Inverness	Raigmore Hospital	Charles Kodikara	
Inverness	Raigmore Hospital	Feng Yi Soh	
Inverness	Raigmore Hospital	Marion Paterson	
Inverness	Raigmore Hospital	Neil McPhail	PI
Inverness	Raigmore Hospital	Sin Ting (Amy) Chan	Co-I
Inverness	Raigmore Hospital	Aristoula Papakostidi	
Inverness	Raigmore Hospital	Azmat Sadozye	
Inverness	Raigmore Hospital	Carol Macgregor	
Inverness	Raigmore Hospital	David Whillis	
Inverness	Raigmore Hospital	Kay Kelly	
Inverness	Raigmore Hospital	Martin Russell	
Inverness	Raigmore Hospital	Steve Nicholson	
Inverness	Raigmore Hospital	Denise Campbell	
Inverness	Raigmore Hospital	Graeme Jervis	Pharmacist
Inverness	Raigmore Hospital	Ian Shread	
Inverness	Raigmore Hospital	Michael Loynd	
Inverness	Raigmore Hospital	Jude Madeleine	Pharmacist
Inverness	Raigmore Hospital	Sean Neville	
Inverness	Raigmore Hospital	Sudhir Borgaonkar	
Inverness	Raigmore Hospital	Anglise Addison	
Inverness	Raigmore Hospital	Karina McQuiston	
Inverness	Raigmore Hospital	Mary McKenzie	Pharmacist
Inverness	Raigmore Hospital	Melanie McIlroy	
Inverness	Raigmore Hospital	Rachel Mackay	
Inverness	Raigmore Hospital	Una Taylor	Pharmacist
Inverness	Raigmore Hospital	Alison Macdonald	
Inverness	Raigmore Hospital	Angela Macgregor	

Inverness	Raigmore Hospital	Florence Anderson	
Inverness	Raigmore Hospital	Georgina Simpson	
Inverness	Raigmore Hospital	Jane Campbell	
Inverness	Raigmore Hospital	Sandra Brown	
Inverness	Raigmore Hospital	Seonaid Arnott	
Inverness	Raigmore Hospital	Anna Robertson	
Inverness	Raigmore Hospital	Anna Skene	
Inverness	Raigmore Hospital	Jane Sinclair	
Inverness	Raigmore Hospital	Joan Stewart	
Inverness	Raigmore Hospital	Karen Callum	
Inverness	Raigmore Hospital	Laura Maclennan	
Inverness	Raigmore Hospital	Margaret Chisholm	
Inverness	Raigmore Hospital	Melissa Lynch	
Inverness	Raigmore Hospital	Zoe Urquhart	Pharmacist
Inverness	Raigmore Hospital	Audrey Campbell	
Inverness	Raigmore Hospital	Catriona Morrison	
Inverness	Raigmore Hospital	Fiona Campbell	
Inverness	Raigmore Hospital	Glenda Sinclair	
Inverness	Raigmore Hospital	Susan Bain	
Ipswich	Ipswich Hospital	Deborah Abrams	
Ipswich	Ipswich Hospital	Kevin Redshaw	
Ipswich	Ipswich Hospital	Sam Obay	
Ipswich	Ipswich Hospital	William Ine	Co-I
Ipswich	Ipswich Hospital	Adiba Hoodbhoy	
Ipswich	Ipswich Hospital	Christopher Scrase	
Ipswich	Ipswich Hospital	Jennifer Collins	
Ipswich	Ipswich Hospital	Ramachandran Venkitaraman	PI
Ipswich	Ipswich Hospital	Sarah Treece	
Ipswich	Ipswich Hospital	Sheen Cherian	
Ipswich	Ipswich Hospital	TJ Podd	Co-I
Ipswich	Ipswich Hospital	Debbie Austin	
Ipswich	Ipswich Hospital	Andy Dann	
Ipswich	Ipswich Hospital	Chris Rose	
Ipswich	Ipswich Hospital	Joe Wells	

Ipswich	Ipswich Hospital	Matt Mendoza	
Ipswich	Ipswich Hospital	Gautam Banerjee	
Ipswich	Ipswich Hospital	Ian Floodgate	Pharmacist
Ipswich	Ipswich Hospital	John Parry	
Ipswich	Ipswich Hospital	Mohsen Habib	
Ipswich	Ipswich Hospital	Paul Ridley	
Ipswich	Ipswich Hospital	Peter Donaldson	
Ipswich	Ipswich Hospital	Robert Brierly	PI
Ipswich	Ipswich Hospital	Charlotte Etheridge	
Ipswich	Ipswich Hospital	Karen Bass	
Ipswich	Ipswich Hospital	Kerry Howlett (nee Brown)	Pharmacist
Ipswich	Ipswich Hospital	Mandy Riley (nee Evans)	
Ipswich	Ipswich Hospital	Susan Teh (Seok San)	
Ipswich	Ipswich Hospital	Sonia Kerridge	
Ipswich	Ipswich Hospital	Susan Upson	
Ipswich	Ipswich Hospital	Yvonne Tricker	
Ipswich	Ipswich Hospital	Harriet Williami	
Ipswich	Ipswich Hospital	Jo Woor	
Ipswich	Ipswich Hospital	Julie Simpson	
Ipswich	Ipswich Hospital	Angharad Williams	
Ipswich	Ipswich Hospital	Julie Spurgeon	
Ipswich	Ipswich Hospital	Natalie Lloyd	
Isle of Wight	St Mary's Hospital (Newport)	Elizabeth Harrison	
Keighley	Airedale General Hospital	Ann Henry	
Keighley	Airedale General Hospital	Charlotte Richardson	
Keighley	Airedale General Hospital	Clara Sentamans	
Keighley	Airedale General Hospital	Nathalie Casanova	
Keighley	Airedale General Hospital	Simon Brown	PI
Keighley	Airedale General Hospital	Sohail Mughal	Co-I
Keighley	Airedale General Hospital	Dan Lee	
Keighley	Airedale General Hospital	Ganesan Jeyasangar	
Keighley	Airedale General Hospital	Michael Crawford	
Keighley	Airedale General Hospital	Sue Cheeseman	
Keighley	Airedale General Hospital	Amy Pendrill	

Keighley	Airedale General Hospital	Andrew Gash	
Keighley	Airedale General Hospital	Joseph Quinn	
Keighley	Airedale General Hospital	Satti Saggu	
Keighley	Airedale General Hospital	Carl Booth	Pharmacist
Keighley	Airedale General Hospital	Anita Ratcliffe	
Keighley	Airedale General Hospital	Josie Snell	
Keighley	Airedale General Hospital	Lisa Bullough	
Keighley	Airedale General Hospital	Liz Shenton	
Keighley	Airedale General Hospital	Pip Hill	
Keighley	Airedale General Hospital	Rachel Kennedy	
Keighley	Airedale General Hospital	Sharron Parkinson	
Keighley	Airedale General Hospital	Alison Shaw	
Keighley	Airedale General Hospital	Gillian Darnbrook	Pharmacist
Keighley	Airedale General Hospital	Jasmine Hartley	
Keighley	Airedale General Hospital	Alison Swindells	Pharmacist
Keighley	Airedale General Hospital	Hayley Bates	
Keighley	Airedale General Hospital	Louise Binns	
Keighley	Airedale General Hospital	Ruth Johnson	
Keighley	Airedale General Hospital	Fiona Farquhar	
Keighley	Airedale General Hospital	Helen Henson	
Keighley	Airedale General Hospital	Judy McAlister	
Keighley	Airedale General Hospital	Maxine Briggs	
Keighley	Airedale General Hospital	Mandy Swanepoel	
Kidderminster	Kidderminster General Hospital	Ayyaz Munawar	Co-I
Kidderminster	Kidderminster General Hospital	Kirsty Clarke	Co-I
Kidderminster	Kidderminster General Hospital	M Habib Khan	
Kidderminster	Kidderminster General Hospital	Shaikh Rana	Co-I
Kidderminster	Kidderminster General Hospital	Lisa Capaldi	PI
Kidderminster	Kidderminster General Hospital	Mark Churn	
Kidderminster	Kidderminster General Hospital	Paul Flinders	Co-I
Kidderminster	Kidderminster General Hospital	Hugh Morrow	
Kidderminster	Kidderminster General Hospital	Monica Gauntlett	Pharmacist
Kidderminster	Kidderminster General Hospital	Veronica Rowlands	
Kidderminster	Kidderminster General Hospital	Emma Marshall	

Kidderminster	Kidderminster General Hospital	Hayley Hodson	
Kidderminster	Kidderminster General Hospital	Helen Tranter	
Kidderminster	Kidderminster General Hospital	Julie Wollaston	
Kidderminster	Kidderminster General Hospital	Kate Field	
Kidderminster	Kidderminster General Hospital	Patricia Rimell	
Kidderminster	Kidderminster General Hospital	Sarah Moss	
Kidderminster	Kidderminster General Hospital	Sally Stringer (pr. Davis)	
Kidderminster	Kidderminster General Hospital	Alison Rosoman	
Kidderminster	Kidderminster General Hospital	Jayne Tyler	
Kidderminster	Kidderminster General Hospital	Linda Higgins	
Kilmarnock	Ayr Hospital	Ricky Hunter	Pharmacist
Kilmarnock	Ayr Hospital	Jennifer Keith	
Kilmarnock	Crosshouse Hospital	Margaret McKernan	
Kilmarnock	Ayr Hospital	Maureen Templeton	
Lancaster	Royal Lancaster Infirmary	Sophie Raby	
Larbert	Forth Valley Royal Hospital	Adam Peters	
Larbert	Forth Valley Royal Hospital	Amy Martin	
Larbert	Forth Valley Royal Hospital	Caroline Lowrie	
Larbert	Forth Valley Royal Hospital	John Martin Russell	
Larbert	Forth Valley Royal Hospital	Saurabh Borgaonkar	
Larbert	Forth Valley Royal Hospital	Stephen McKay	
Larbert	Forth Valley Royal Hospital	Carolynn Lamb	
Larbert	Forth Valley Royal Hospital	Nadja Melquiot	
Larbert	Forth Valley Royal Hospital	Norma Sidek	PI
Larbert	Forth Valley Royal Hospital	Eilidh Henderson	
Larbert	Forth Valley Royal Hospital	Kirsty Young	
Larbert	Forth Valley Royal Hospital	Mhari TTaylor	
Larbert	Forth Valley Royal Hospital	Stephanie Brogan (nee Roddie)	
Larbert	Forth Valley Royal Hospital	Seamus Teahan	
Larbert	Forth Valley Royal Hospital	Anna Hamilton	
Larbert	Forth Valley Royal Hospital	Anne Todd	
Larbert	Forth Valley Royal Hospital	Joanne Robinson	Pharmacist
Larbert	Forth Valley Royal Hospital	Lesley Symon	
Larbert	Forth Valley Royal Hospital	Patricia Turner	

Larbert	Forth Valley Royal Hospital	Lynn Prentice	
Larbert	Forth Valley Royal Hospital	Maureen Hamill	
Larbert	Forth Valley Royal Hospital	Sally Young	
Larbert	Forth Valley Royal Hospital	Alison Yule	
Larbert	Beatson West of Scotland Cancer Centre	Maureen Hamill	
Lausanne	Centre Hospitalier Universitaire Vaudois (CHUV)	Geert Van Driessche	
Lausanne	Centre Hospitalier Universitaire Vaudois (CHUV)	Benangene Midez	
Lausanne	Centre Hospitalier Universitaire Vaudois (CHUV)	Angela Orcurto	
Lausanne	Centre Hospitalier Universitaire Vaudois (CHUV)	Antonella Diciolla	
Lausanne	Centre Hospitalier Universitaire Vaudois (CHUV)	Claire Perrinjaquet	
Lausanne	Centre Hospitalier Universitaire Vaudois (CHUV)	Dominik Berthold	PI
Lausanne	Centre Hospitalier Universitaire Vaudois (CHUV)	Louis Parisod	
Lausanne	Centre Hospitalier Universitaire Vaudois (CHUV)	Sofiya Latifyan	
Lausanne	Centre Hospitalier Universitaire Vaudois (CHUV)	Fernanda Herrera	
Lausanne	Centre Hospitalier Universitaire Vaudois (CHUV)	Akram Farhat	
Lausanne	Centre Hospitalier Universitaire Vaudois (CHUV)	Yohan Boillat	
Lausanne	Centre Hospitalier Universitaire Vaudois (CHUV)	Alexandra Rideau	
Lausanne	Centre Hospitalier Universitaire Vaudois (CHUV)	Fabrice Lalubin	
Lausanne	Centre Hospitalier Universitaire Vaudois (CHUV)	Hans-peter Roth	
Lausanne	Centre Hospitalier Universitaire Vaudois (CHUV)	Jean-Philippe Zurcher	
Lausanne	Centre Hospitalier Universitaire Vaudois (CHUV)	Tewfik Abedlaziz	
Lausanne	Centre Hospitalier Universitaire Vaudois (CHUV)	Kaniana Ntanga Muambayi	
Lausanne	Centre Hospitalier Universitaire Vaudois (CHUV)	Sandra Toffanin	
Lausanne	Centre Hospitalier Universitaire Vaudois (CHUV)	Agnes Hiou Feige	
Lausanne	Centre Hospitalier Universitaire Vaudois (CHUV)	Alice Abdallah	
Lausanne	Centre Hospitalier Universitaire Vaudois (CHUV)	Aline Voidey	
Lausanne	Centre Hospitalier Universitaire Vaudois (CHUV)	Anabela Costa	
Lausanne	Centre Hospitalier Universitaire Vaudois (CHUV)	Anna-Sophia Briod	
Lausanne	Centre Hospitalier Universitaire Vaudois (CHUV)	Carmen Castagna	
Lausanne	Centre Hospitalier Universitaire Vaudois (CHUV)	Catherine Bender	
Lausanne	Centre Hospitalier Universitaire Vaudois (CHUV)	Celine Yerly	
Lausanne	Centre Hospitalier Universitaire Vaudois (CHUV)	Cosette Schuler	
Lausanne	Centre Hospitalier Universitaire Vaudois (CHUV)	Cynthia Leclerc	
Lausanne	Centre Hospitalier Universitaire Vaudois (CHUV)	Eloise Kremer	

Lausanne	Centre Hospitalier Universitaire Vaudois (CHUV)	Floriane Bouilly	
Lausanne	Centre Hospitalier Universitaire Vaudois (CHUV)	Margaret McLauchlan	
Lausanne	Centre Hospitalier Universitaire Vaudois (CHUV)	May-Lucie Meyer	Co-I
Lausanne	Centre Hospitalier Universitaire Vaudois (CHUV)	Nathalie Divorne	
Lausanne	Centre Hospitalier Universitaire Vaudois (CHUV)	Nicole James Faresse	
Lausanne	Centre Hospitalier Universitaire Vaudois (CHUV)	Norlene Silva	
Lausanne	Centre Hospitalier Universitaire Vaudois (CHUV)	Rebecca Oppenheim	
Lausanne	Centre Hospitalier Universitaire Vaudois (CHUV)	Sophie Voegtlin	
Lausanne	Centre Hospitalier Universitaire Vaudois (CHUV)	Sylvie Haudidier	
Lausanne	Centre Hospitalier Universitaire Vaudois (CHUV)	Veronica Aedo	
Lausanne	Centre Hospitalier Universitaire Vaudois (CHUV)	Patrice Jichlinski	
Lausanne	Centre Hospitalier Universitaire Vaudois (CHUV)	Galaad Bernard	
Lausanne	Centre Hospitalier Universitaire Vaudois (CHUV)	Marc Schnety	
Lausanne	Centre Hospitalier Universitaire Vaudois (CHUV)	Sabine Galland	
Lausanne	Centre Hospitalier Universitaire Vaudois (CHUV)	Sophia Murel	
Leeds	St James University Hospital (Leeds)	Ann Henry	
Leeds	St James University Hospital (Leeds)	Ann Henry	
Leeds	St James University Hospital (Leeds)	Christopher Williams	
Leeds	St James University Hospital (Leeds)	Christy Ralph	
Leeds	Bradford Royal Infirmary	Ee Siang Choong	
Leeds	Bradford Royal Infirmary	Emma Dugdale	
Leeds	St James University Hospital (Leeds)	Ian Boon	
Leeds	St James University Hospital (Leeds)	Joseph Joji	
Leeds	Airedale General Hospital	Katy Clarke	
Leeds	Bradford Royal Infirmary	Katy Clarke	
Leeds	Bradford Royal Infirmary	Michael Flatley	Co-I
Leeds	St James University Hospital (Leeds)	Naveen Vasudev	Co-I
Leeds	Cookridge Hospital	Anne Kiltie	
Leeds	St James University Hospital (Leeds)	Anne Kiltie	
Leeds	St James University Hospital (Leeds)	Carmel Loughrey	
Leeds	St James University Hospital (Leeds)	David Bottomley	
Leeds	St James University Hospital (Leeds)	Hima Bindu Musunuru	
Leeds	St James University Hospital (Leeds)	Janet Brown	
Leeds	St James University Hospital (Leeds)	John Chester	

Leeds	St James University Hospital (Leeds)	Kevin Franks	
Leeds	St James University Hospital (Leeds)	Krishna Shastry	
Leeds	Bradford Royal Infirmary	Lisa Owen	Co-I
Leeds	St James University Hospital (Leeds)	Luis Daverede	
Leeds	St James University Hospital (Leeds)	Richard Khafagy	
Leeds	St James University Hospital (Leeds)	Satinder Jagdev	
Leeds	St James University Hospital (Leeds)	Claire Posnett	
Leeds	St James University Hospital (Leeds)	Hannah Roberts	
Leeds	St James University Hospital (Leeds)	Javeria Akhtar	
Leeds	St James University Hospital (Leeds)	Jodene Hill	
Leeds	St James University Hospital (Leeds)	Judith Evans	
Leeds	St James University Hospital (Leeds)	Svetoslava Doshmanonska	
Leeds	St James University Hospital (Leeds)	Liz Hudson	
Leeds	St James University Hospital (Leeds)	Christopher Main	
Leeds	St James University Hospital (Leeds)	Ruiyang Yan	
Leeds	St James University Hospital (Leeds)	Sanjeev Kotwal	
Leeds	St James University Hospital (Leeds)	William Cross	PI
Leeds	Leeds General Infirmary	Adrian Joyce	
Leeds	St James University Hospital (Leeds)	Adrian Joyce	
Leeds	St James University Hospital (Leeds)	Alan Paul	
Leeds	St James University Hospital (Leeds)	James Cavanagh	
Leeds	St James University Hospital (Leeds)	Peter Whelan	PI
Leeds	St James University Hospital (Leeds)	Rafal Turo	
Leeds	St James University Hospital (Leeds)	Sam Lotfi	
Leeds	St James University Hospital (Leeds)	Stephen Prescott	
Leeds	St James University Hospital (Leeds)	Sunjay Jain	
Leeds	St James University Hospital (Leeds)	Catherine Parbutt	
Leeds	St James University Hospital (Leeds)	Jude Clarke	
Leeds	St James University Hospital (Leeds)	Lorraine Wiseman	
Leeds	St James University Hospital (Leeds)	Polapo Ajayi	
Leeds	St James University Hospital (Leeds)	Angela Morgan	
Leeds	St James University Hospital (Leeds)	Anne Crossley	
Leeds	St James University Hospital (Leeds)	Dolapo Ajayi	
Leeds	St James University Hospital (Leeds)	Hannah Wigginton	

Leeds	St James University Hospital (Leeds)	Lorraine Wiseman	
Leeds	Leeds General Infirmary	Caroline Bedford	
Leeds	St James University Hospital (Leeds)	Caroline Bedford	Pharmacist
Leeds	St James University Hospital (Leeds)	Charlotte Pool	
Leeds	St James University Hospital (Leeds)	Emily Davies	
Leeds	St James University Hospital (Leeds)	Emma Lundy	Pharmacist
Leeds	St James University Hospital (Leeds)	Gemma Austin (nee Glover)	
Leeds	St James University Hospital (Leeds)	Helen Payne	
Leeds	St James University Hospital (Leeds)	Jade McCann	
Leeds	St James University Hospital (Leeds)	Judith Chapman	
Leeds	St James University Hospital (Leeds)	Maria Hall	
Leeds	St James University Hospital (Leeds)	Mercy Kaiga	
Leeds	St James University Hospital (Leeds)	Sue Rodwell	
Leeds	St James University Hospital (Leeds)	Sue Sibson	
Leeds	Cookridge Hospital	Richard Kaplan	
Leeds	St James University Hospital (Leeds)	Richard Kaplan	
Liestal	Kantonsspital Liestal	Eloise Kremer	
Liestal	Kantonsspital Liestal	Simone Marini	
Liestal	Kantonsspital Liestal	Vanessa Fuhrer	
Liestal	Kantonsspital Liestal	Andreas Lohri	
Lincoln	Lincoln County Hospital	Ana Fernandez-Ots	Co-I
Lincoln	Lincoln County Hospital	Andrew Sloan	
Lincoln	Lincoln County Hospital	Christian Arias	
Lincoln	Lincoln County Hospital	David Ballesteros-Quintail	
Lincoln	Lincoln County Hospital	Elena Macleod	
Lincoln	Lincoln County Hospital	Sindhu Ramarwothy	Co-I
Lincoln	Lincoln County Hospital	Yogesh Nishchal	
Lincoln	Lincoln County Hospital	Alfredo Addeo	
Lincoln	Lincoln County Hospital	Karin Baria	
Lincoln	Lincoln County Hospital	Miguel Panades	PI
Lincoln	Lincoln County Hospital	Prantik Das	Co-I
Lincoln	Lincoln County Hospital	Thiagarajan Sreenivasan	PI
Lincoln	Pilgrim Hospital	Thiagarajan Sreenivasan	PI
Lincoln	Lincoln County Hospital	Gunjan Phalod	

Lincoln	Lincoln County Hospital	Andrew Judd	
Lincoln	Lincoln County Hospital	Ray McDermott	
Lincoln	Lincoln County Hospital	Stephen Audu	Pharmacist
Lincoln	Lincoln County Hospital	Simon Archer	
Lincoln	Lincoln County Hospital	Alyson Wilson	
Lincoln	Lincoln County Hospital	Diane Carey	
Lincoln	Lincoln County Hospital	Kathryn Pearson	
Lincoln	Lincoln County Hospital	Kerri Johnson	
Lincoln	Lincoln County Hospital	Laura Walsh	
Lincoln	Lincoln County Hospital	Maryanne Okubanjo	
Lincoln	Lincoln County Hospital	Olesya Francis	
Lincoln	Lincoln County Hospital	Caroline Taylor	Pharmacist
Lincoln	Lincoln County Hospital	Suzanne Archer	
Lincoln	Lincoln County Hospital	Giuseppe Banna	
Lincoln	Lincoln County Hospital	Helen Carolan	
Lincoln	Lincoln County Hospital	Janet Tomlinson	
Lincoln	Lincoln County Hospital	Jayne Borley	Pharmacist
Lincoln	Lincoln County Hospital	Jenny Salmon	
Lincoln	Lincoln County Hospital	Kathryn Hoare	
Lincoln	Lincoln County Hospital	Rebecca Spencer	
Lincoln	Lincoln County Hospital	Rhiannan Pegg	
Lincoln	Lincoln County Hospital	Sarah Bell	Pharmacist
Lincoln	Lincoln County Hospital	Sarah Coombs	
Lincoln	Lincoln County Hospital	Stephanie Barker	
Lincoln	Lincoln County Hospital	Annette Hilldrith	
Lincoln	Lincoln County Hospital	Carol Lockwood	
Lincoln	Lincoln County Hospital	Rachel Newton	
Lincoln	Lincoln County Hospital	Susie Butler	
Liverpool	Royal Liverpool University Hospital	Jasima Latif	
Liverpool	Royal Liverpool University Hospital	Chinnamani Eswar	Co-I
Liverpool	University Hospital Aintree	Paul Hill	
Liverpool	Royal Liverpool University Hospital	Peter Robson	
Liverpool	University Hospital Aintree	Peter Robson	PI
Liverpool	Royal Liverpool University Hospital	Zafar Malik	PI

Liverpool	University Hospital Aintree	Lucy Berresford	
Liverpool	University Hospital Aintree	Rachael Fergusson	
Liverpool	Royal Liverpool University Hospital	Katy Treherne	
Liverpool	Royal Liverpool University Hospital	Kevin McDonald	
Liverpool	Royal Liverpool University Hospital	Paul Griffiths	
Liverpool	Royal Liverpool University Hospital	Philip Reynolds	
Liverpool	University Hospital Aintree	Wesley Artist	
Liverpool	University Hospital Aintree	Lorraine Lancaster	
Liverpool	Royal Liverpool University Hospital	Nidhi Sibal	
Liverpool	Royal Liverpool University Hospital	Sharon Dunn (nee Johnson)	
Liverpool	Royal Liverpool University Hospital	Sue Green	
Liverpool	Royal Liverpool University Hospital	Sandra Robinson	
Liverpool	University Hospital Aintree	Sandra Robinson	Pharmacist
Liverpool	Royal Liverpool University Hospital	Nicola Bermingham	
Liverpool	Royal Liverpool University Hospital	Pembe Yesildag	
Liverpool	Royal Liverpool University Hospital	Dawn Porter	
Liverpool	University Hospital Aintree	Haley McCulloch	
Liverpool	Royal Liverpool University Hospital	Julie Griffiths	
Liverpool	University Hospital Aintree	Julie Griffiths	
Liverpool	University Hospital Aintree	Leigh Pauls	
Liverpool	Royal Liverpool University Hospital	Lisa Dobson (nee Child)	
Liverpool	University Hospital Aintree	Lisa Dobson (nee Child)	Pharmacist
Liverpool	Royal Liverpool University Hospital	Lizzie Dale	
Liverpool	Royal Liverpool University Hospital	Pauline Pilkington	
London	Royal Marsden Hospital (London)	Adnan Akhtar	
London	Guy's Hospital (London)	Ajay Aggarwal	
London	University College Hospital	Anita Mitra	
London	Royal Marsden Hospital (London)	Anna Wilkins	
London	University College Hospital	Anuradha Jayaram	
London	University College Hospital	Bianca Tryillo	
London	University College Hospital	Bihani Kularatne	
London	Guy's Hospital (London)	Caterina Aversa	
London	St Bartholomews Hospital (London)	Cavitha Vivekananthan	
London	Guy's Hospital (London)	Chara Stavraka	Co-I

London	Guy's Hospital (London)	Charalampos Gousis	
London	Guy's Hospital (London)	Charleen Chan Wah Hak	
London	Guy's Hospital (London)	Clare Gilson	
London	Guy's Hospital (London)	Deborah Enting	
London	Guy's Hospital (London)	Delali Adjogatse	
London	University College Hospital	Dieo Ottaviani	
London	Guy's Hospital (London)	Eirini Tsotra	
London	Guy's Hospital (London)	Eleni Josephides	
London	Guy's Hospital (London)	Elias Pintus	
London	Queen Elizabeth Hospital (Woolwich)	Elias Pintus	Co-I
London	Royal Marsden Hospital (London)	Emily Durie	
London	Royal Free Hospital	Emily Scott	Co-I
London	Royal Marsden Hospital (London)	Ewan Chapman	Co-I
London	St Georges Hospital (London)	Gelareh Eslamian	
London	University College Hospital	Gianmarco Leone	
London	Royal Free Hospital	Grant Stewart	Co-I
London	Guy's Hospital (London)	Hannah Rush	
London	Royal Marsden Hospital (London)	James Lowe	
London	Royal Marsden Hospital (London)	Jana McHugh	
London	Guy's Hospital (London)	Jennifer Turner	
London	Royal Marsden Hospital (London)	Kallol Bhadra	
London	Guy's Hospital (London)	Kamarul Zaki	
London	St Bartholomews Hospital (London)	Karen Tipples	PI
London	Royal Free Hospital	Kate Smith	Co-I
London	Guy's Hospital (London)	Kiruthikah Thillai	
London	St Georges Hospital (London)	Laura Camburn	Co-I
London	Royal Marsden Hospital (London)	Leron Okonta	
London	Royal Free Hospital	Magdalena Kubiak	Co-I
London	University College Hospital	Maise Albakir	
London	Guy's Hospital (London)	Mark Voskoboynik	
London	Guy's Hospital (London)	Matthaius Kapiris	
London	St Georges Hospital (London)	Mohammed Mahgoub	
London	Guy's Hospital (London)	Muhammad Khan	
London	Royal Marsden Hospital (London)	Nicholas Vanas	

London	Royal Free Hospital	Nicola Rosenfelder	Co-I
London	Guy's Hospital (London)	Nikolaos Tsoukalas	
London	Charing Cross Hospital	Paul Kabuubi	
London	Guy's Hospital (London)	Rosalind Kieran	
London	St Georges Hospital (London)	Roxane Mather	
London	Royal Free Hospital	Ruochen Li	
London	Guy's Hospital (London)	Sabeeh Butt	
London	Guy's Hospital (London)	Sarah Howiett	Co-I
London	Royal Free Hospital	Sarah Needleman	PI
London	Charing Cross Hospital	Stephen Mangar	Co-I
London	Hammersmith Hospital	Stephen Mangar	Co-I
London	St Marys Hospital (London)	Stephen Mangar	
London	Guy's Hospital (London)	Stephen Morris	
London	Guy's Hospital (London)	Thomas Bird	
London	Guy's Hospital (London)	Thubeena Manickavasagar	
London	Royal Marsden Hospital (London)	Tzveta porrovska	
London	Royal Marsden Hospital (London)	Vedang Murthy	
London	Guy's Hospital (London)	Vinod Mullassery	
London	Queen Elizabeth Hospital (Woolwich)	Vinod Mullassery	Co-I
London	Guy's Hospital (London)	Vishal Manik	Co-I
London	Guy's Hospital (London)	Yin Wu	
London	Charing Cross Hospital	Alison Falconer	PI
London	Hammersmith Hospital	Alison Falconer	PI
London	St Marys Hospital (London)	Alison Falconer	PI
London	Royal Marsden Hospital (London)	Alison Reid	
London	Guy's Hospital (London)	Angel Garcia-Imhof	
London	Guy's Hospital (London)	Anna Karpathakis	
London	Guy's Hospital (London)	Archie Macnair	
London	Queen Elizabeth Hospital (Woolwich)	Arunansu Kar	
London	North Middlesex Hospital	Asim Ray	
London	Royal Free Hospital	Daniel Smith	
London	Guy's Hospital (London)	Daniel Tong	
London	Guy's Hospital (London)	Danielle Crawley	
London	Guy's Hospital (London)	Debra Josephs	

London	Charing Cross Hospital	Ethna Mannion	
London	North Middlesex Hospital	Farhad Neave	
London	Guy's Hospital (London)	Hartmut Kristeleit	
London	Queen Elizabeth Hospital (Woolwich)	Hartmut Kristeleit	
London	University College Hospital	Heather Payne	Co-I
London	North Middlesex Hospital	Jackie Newby	
London	St Georges Hospital (London)	Jason Chow	Co-I
London	St Bartholomews Hospital (London)	Jonathon Shamash	
London	University College Hospital	Judith Cave	
London	Royal Marsden Hospital (London)	Karen Chan	
London	Royal Free Hospital	Katherine Pigott	
London	Guy's Hospital (London)	Lawrence Krieger	
London	St Georges Hospital (London)	Lisa Pickering	
London	Guy's Hospital (London)	Lucy Juggins	
London	Royal Free Hospital	Maria Vilarino-Varela	PI
London	University College Hospital	Mark Linch	Co-I
London	St Georges Hospital (London)	Mehran Afshar	PI
London	Charing Cross Hospital	Naveed Sarwar	
London	Royal Marsden Hospital (London)	Nicholas Van As	
London	Queen Elizabeth Hospital (Woolwich)	Nick Maisey	
London	St Bartholomews Hospital (London)	Paula Wells	Co-I
London	Guy's Hospital (London)	Ramin Ajami	
London	University College Hospital	Reena Davda	
London	Guy's Hospital (London)	Ronald Beaney	
London	Royal Marsden Hospital (London)	Rosalind Eeles	
London	Guy's Hospital (London)	Rushan Sylva	
London	Guy's Hospital (London)	Sarah Hargreaves	
London	Guy's Hospital (London)	Sarah Rudman	PI
London	Guy's Hospital (London)	Sharmistha Ghosh	
London	Guy's Hospital (London)	Sheeba Irshad	
London	Guy's Hospital (London)	Simon Chowdhury	
London	Guy's Hospital (London)	Simon Hughes	
London	Queen Elizabeth Hospital (Woolwich)	Simon Hughes	
London	St Marys Hospital (London)	Simon Stewart	

London	Queen Elizabeth Hospital (Woolwich)	Sindu Vivekanandan	PI
London	St Georges Hospital (London)	Sophie McGrath	
London	St Bartholomews Hospital (London)	Stephanie Gibbs	
London	University College Hospital	Stephen Harland	
London	Guy's Hospital (London)	Susanne Allan	
London	Guy's Hospital (London)	Teresa Guerrero-Urbano	
London	University College Hospital	Ursula McGovern	PI
London	University College Hospital	Uzma Asghar	
London	Guy's Hospital (London)	Vasiliki Michalarea	
London	Royal Marsden Hospital (London)	Vincent Khoo	PI
London	St Bartholomews Hospital (London)	Wing-Kin Liu	
London	University College Hospital	Thomas Amoaten	
London	St Georges Hospital (London)	Alice Dainty	
London	Guy's Hospital (London)	Anita Soma	Pharmacist
London	St Georges Hospital (London)	Asha Mistry	
London	St Georges Hospital (London)	Claire Gilmartin	
London	Royal Marsden Hospital (London)	Eleanor Quinn	
London	Royal Free Hospital	Emma Douch	
London	St Bartholomews Hospital (London)	Hannah Payne	
London	Royal Marsden Hospital (London)	Holly Hogan	
London	Royal Marsden Hospital (London)	Jennyfa Ali	
London	Royal Free Hospital	Jessica Hunt	
London	Charing Cross Hospital	Kerry Richards	
London	Royal Free Hospital	Kharishma Makani	
London	Guy's Hospital (London)	Louisa McDonald	
London	University College Hospital	Martha Wilson	
London	Royal Marsden Hospital (London)	Nicola Lucas	
London	University College Hospital	Nicole Bonsu	
London	St Bartholomews Hospital (London)	Olivia Bolton	
London	University College Hospital	Samirah Rokib	
London	Guy's Hospital (London)	Sarah King	
London	St Georges Hospital (London)	Serena Dover	
London	St Bartholomews Hospital (London)	Sultana Begum	
London	Guy's Hospital (London)	Tahereh Ghadimi	

London	St Georges Hospital (London)	Uforma Ogrigri	Pharmacist
London	Royal Marsden Hospital (London)	Vijitha Vijayakumar	
London	Guy's Hospital (London)	Vivien Quan	
London	Queen Elizabeth Hospital (Woolwich)	Melody Ncube	
London	Queen Elizabeth Hospital (Woolwich)	Abel Jalloh	
London	Queen Elizabeth Hospital (Woolwich)	Abhijit Jadhav	
London	Guy's Hospital (London)	Antonio Querol-Rubiera	
London	Guy's Hospital (London)	Declan Cahill	
London	Guy's Hospital (London)	Fahim Ahmed	
London	St Bartholomews Hospital (London)	Fatjon Dekaj	
London	Charing Cross Hospital	Gareth Barker	Pharmacist
London	St Marys Hospital (London)	Gareth Barker	
London	St Georges Hospital (London)	Geoffrey Howell	
London	St Georges Hospital (London)	Hakim Guessous	
London	University College Hospital	Ignacio Blanch	
London	Queen Elizabeth Hospital (Woolwich)	Jagdev Bains	Pharmacist
London	University College Hospital	Joel Watson	
London	St Bartholomews Hospital (London)	Jude Nixon	
London	St Georges Hospital (London)	Juel Tuazon	
London	University College Hospital	Kristian Warnes	Pharmacist
London	Royal Marsden Hospital (London)	Li Wancheung	
London	Queen Elizabeth Hospital (Woolwich)	Luke Maidment	
London	St Georges Hospital (London)	Michael Brown	
London	Royal Marsden Hospital (London)	Michael Moneypenny	
London	St Bartholomews Hospital (London)	Oscar Riches	
London	St Bartholomews Hospital (London)	P Cathcart	
London	Guy's Hospital (London)	Rayhan Ahmed	
London	Queen Elizabeth Hospital (Woolwich)	Rayhan Ahmed	
London	Guy's Hospital (London)	Rick Popert	
London	St Georges Hospital (London)	Robert Varro	
London	Charing Cross Hospital	Ross Dalton-Short	
London	St Georges Hospital (London)	Sam Hollingworth	
London	St Bartholomews Hospital (London)	Sebastien Martin	
London	Whittington Hospital (London)	Simon Wan	

London	Guy's Hospital (London)	Sumeet Sisodia	
London	Queen Elizabeth Hospital (Woolwich)	Vinod Mullessey	PI
London	St Bartholomews Hospital (London)	Alastair Nicholson	
London	Royal Marsden Hospital (London)	Bernard Sill	
London	Royal Marsden Hospital (London)	Bernard Siu	
London	North Middlesex Hospital	Chris Abbott	Pharmacist
London	University College Hospital	Danny Garrett	
London	Guy's Hospital (London)	Gerry Trillana	
London	Hammersmith Hospital	Ilyas Ali	
London	Guy's Hospital (London)	Jozer Calara	
London	Charing Cross Hospital	Kwame Ansu	
London	Queen Elizabeth Hospital (Woolwich)	Lee Porin	
London	St Georges Hospital (London)	Mark Quarrell	
London	Queen Elizabeth Hospital (Woolwich)	Nigel Holmes	
London	Guy's Hospital (London)	Philip Reynolds	
London	Queen Elizabeth Hospital (Woolwich)	Philip Reynolds	
London	Charing Cross Hospital	Steve Edwards	
London	Hammersmith Hospital	Steve Edwards	
London	St Marys Hospital (London)	Steve Edwards	
London	Queen Elizabeth Hospital (Woolwich)	Thomas Sarkodie	
London	Guy's Hospital (London)	Thomas Spencer	
London	Guy's Hospital (London)	Trevor Bott	
London	Royal Marsden Hospital (London)	Trevor Bott	
London	University College Hospital	Adrienne Abioye	
London	Queen Elizabeth Hospital (Woolwich)	Joyce Maravi	
London	Royal Free Hospital	Kaliyanee Ramtohul	
London	Guy's Hospital (London)	Ngozi Muoneke	
London	St Georges Hospital (London)	Nia Alsamarrai	
London	St Bartholomews Hospital (London)	Resmi Jayachandran	
London	University College Hospital	Roshni Goel	
London	Royal Marsden Hospital (London)	Ruth Stafferton	
London	St Bartholomews Hospital (London)	Samantha Chetiyawardana	
London	Queen Elizabeth Hospital (Woolwich)	Samia Pilgrim	
London	Royal Marsden Hospital (London)	Sarah Storrs	

London	Charing Cross Hospital	Zohanon Sabine Loko	
London	St Marys Hospital (London)	Zohanon Sabine Loko	
London	Charing Cross Hospital	Andrea Davis-Cook	Pharmacist
London	St Marys Hospital (London)	Andrea Davis-Cook	
London	Guy's Hospital (London)	Chi Yee Chung	Pharmacist
London	Royal Marsden Hospital (London)	Debra Townsend-Thorn	
London	University College Hospital	Holly Baker (nee. Wing)	Pharmacist
London	University College London	Holly Baker (nee. Wing)	
London	Hammersmith Hospital	Regina Storch	Pharmacist
London	Guy's Hospital (London)	Sharon McPherson	
London	Royal Marsden Hospital (London)	Sijy Pillai	
London	Royal Free Hospital	Aarti Nandani	Pharmacist
London	Queen Elizabeth Hospital (Woolwich)	Aarti Shah	
London	University Hospital Lewisham	Aarti Shah	
London	University College Hospital	Agnieska Zielonka	
London	University College Hospital	Aileen Austria	
London	Royal Free Hospital	Anna Osadcow	
London	St Georges Hospital (London)	Anne Haldeos	
London	Queen Elizabeth Hospital (Woolwich)	Anne-Marie Vindidu	Pharmacist
London	Royal Marsden Hospital (London)	Annette Musallam	
London	Royal Marsden Hospital (London)	Asma Varachia	
London	Guy's Hospital (London)	Awo Abdi	
London	Charing Cross Hospital	Bindu Chikkamuniyappa	
London	St Marys Hospital (London)	Bindu Chikkamuniyappa	
London	St Georges Hospital (London)	Chandni Patel	
London	Guy's Hospital (London)	Claire Glendon	
London	Royal Free Hospital	Claire Jarvis	
London	St Georges Hospital (London)	Deirdre Daly	
London	St Bartholomews Hospital (London)	Denise Humfress	
London	Guy's Hospital (London)	Emma O'Connor	
London	Guy's Hospital (London)	Eva Batovska	
London	Royal Marsden Hospital (Sutton)	Eva Batovska	Pharmacist
London	Royal Marsden Hospital (London)	Giulia Carlino	
London	Royal Free Hospital	Hannah Powell	

London	Royal Marsden Hospital (London)	Helen Stidwell	
London	University College Hospital	Helene Zilkha	
London	St Georges Hospital (London)	Jane Gregg	
London	University College Hospital	Jane Leach	Pharmacist
London	University College Hospital	Javeria Akhtar	
London	Queen Elizabeth Hospital (Woolwich)	Jennifer Martin	Pharmacist
London	Royal Marsden Hospital (London)	Juliet Owusu	
London	Guy's Hospital (London)	Kafui Dossa	
London	Royal Marsden Hospital (London)	Karen Brooks	
London	Royal Marsden Hospital (London)	Lexi Vick	
London	Guy's Hospital (London)	Linda Shephard	
London	Royal Free Hospital	Lorna O'Shea	
London	Guy's Hospital (London)	Louisa Fleure	
London	St Bartholomews Hospital (London)	Marina Baccarini	
London	Royal Marsden Hospital (London)	Marisa Pinto Peixoto	
London	Queen Elizabeth Hospital (Woolwich)	Martha Handousa	
London	Queen Elizabeth Hospital (Woolwich)	Miriam Cottle	
London	Queen Elizabeth Hospital (Woolwich)	Nadia El-Sayed	
London	Royal Free Hospital	Naomi Anderson	
London	University College Hospital	Natasha Aslam	
London	Royal Marsden Hospital (London)	Nicola Harman	
London	Hammersmith Hospital	Nikki Kettley	
London	Guy's Hospital (London)	Rebecca Way	
London	Royal Free Hospital	Sabina Melander	
London	Queen Elizabeth Hospital (Woolwich)	Sagira Khatun	Pharmacist
London	Guy's Hospital (London)	Sally Walker	
London	St Marys Hospital (London)	Severine Rey	
London	St Bartholomews Hospital (London)	Shahanara Ferdous	
London	Queen Elizabeth Hospital (Woolwich)	Shahreen Ahmed	
London	Queen Elizabeth Hospital (Woolwich)	Shanna Wilson	
London	Guy's Hospital (London)	Susie Slater	
London	Queen Elizabeth Hospital (Woolwich)	Suzanne Chukundah	
London	University College Hospital	Suzy Lowi	
London	Royal Free Hospital	Tesha Suddason	

London	Queen Elizabeth Hospital (Woolwich)	Theodorah Nago	
London	University College Hospital	Yemi Ilumoka	
London	Charing Cross Hospital	Akeema Paul	
London	St Marys Hospital (London)	Akeema Paul	
London	St Marys Hospital (London)	Angela Chamberlain	
London	Royal Free Hospital	Angela McCadden	
London	Guy's Hospital (London)	Anna Parker	
London	University College Hospital	Annelies Gillesen	
London	Guy's Hospital (London)	Belinda Chitando	
London	Queen Elizabeth Hospital (Woolwich)	Belinda Chitando	
London	Queen Elizabeth Hospital (Woolwich)	Bridget Kabagambe	
London	Guy's Hospital (London)	Catherine Rogers	
London	Guy's Hospital (London)	Cheryl Lawrence	
London	St Bartholomews Hospital (London)	Cheryl Lawrence	Pharmacist
London	Royal Marsden Hospital (London)	Chloe McCormack	
London	Charing Cross Hospital	Daisy Floyd	
London	St Marys Hospital (London)	Daisy Floyd	
London	North Middlesex Hospital	Debbie Blois	
London	Royal Marsden Hospital (London)	Debbie Rolfe	
London	St Georges Hospital (London)	Debbie Rolfe	
London	University College Hospital	Didem Agdiran	
London	Guy's Hospital (London)	Donna Cassidy	
London	Royal Free Hospital	Elizabeth Woodford	Pharmacist
London	Guy's Hospital (London)	Emilia Caverly	
London	Hammersmith Hospital	Emily Pickford	
London	Queen Elizabeth Hospital (Woolwich)	Eti Omoregie	
London	Guy's Hospital (London)	Francesca Curran	
London	St Marys Hospital (London)	Gillian Hornzee	
London	University College Hospital	Hannah Ansell	Pharmacist
London	Queen Elizabeth Hospital (Woolwich)	Hazel Harrop	
London	Guy's Hospital (London)	Helen Snow	
London	St Georges Hospital (London)	Helen Tighe	
London	Charing Cross Hospital	Ibiyemi Sadare (Olaleye)	
London	St Bartholomews Hospital (London)	Janet Kiff	

London	St Bartholomews Hospital (London)	Janet Oladimeji	
London	Guy's Hospital (London)	Janette Nichol	
London	Royal Marsden Hospital (London)	Jennifer Morrison	
London	Charing Cross Hospital	Jill Gallagher	
London	St Marys Hospital (London)	Joy Liao	
London	St Marys Hospital (London)	Joy Liau	
London	North Middlesex Hospital	Judy Hill	
London	Royal Free Hospital	Juniebel Cooke	Pharmacist
London	Guy's Hospital (London)	Kate Williams	
London	Royal Marsden Hospital (London)	Laillah-Crystal Banda	
London	Queen Elizabeth Hospital (Woolwich)	Laura Beschizza	
London	St Marys Hospital (London)	Laura Custins	
London	North Middlesex Hospital	Lorraine Hurl	
London	Guy's Hospital (London)	Lucy Reed	
London	Queen Elizabeth Hospital (Woolwich)	Maria Liskova	
London	Royal Free Hospital	Marisa Lanzman	
London	St Marys Hospital (London)	Melloney Allnutt	
London	Guy's Hospital (London)	Michelle Dutton	
London	Charing Cross Hospital	Najma Ahmed	
London	University College Hospital	Noan-Minh Chau	
London	University College Hospital	Patricia Danaswamy	
London	Queen Elizabeth Hospital (Woolwich)	Rachel Harper	
London	Guy's Hospital (London)	Rebecca Todd	
London	Guy's Hospital (London)	Ruth Johnson	
London	Charing Cross Hospital	Samantha Weller	
London	Royal Free Hospital	Sara Fawcitt	
London	Queen Elizabeth Hospital (Woolwich)	Sharai Chitando	
London	Guy's Hospital (London)	Sharon Clovis	
London	St Georges Hospital (London)	Sophie Golden	
London	Guy's Hospital (London)	Srivani Kandasamy	
London	Guy's Hospital (London)	Stephanie Argue	
London	Charing Cross Hospital	Stephanie Steadman	
London	Royal Free Hospital	Su Fung Lo	Pharmacist
London	St Georges Hospital (London)	Sue Cromarty	

London	Royal Marsden Hospital (London)	Suraya Quadir	
London	Guy's Hospital (London)	Suzanne Vizor	
London	Guy's Hospital (London)	Temi Olusi	Pharmacist
London	Guy's Hospital (London)	Ursula Kirwan	
London	University College Hospital	John Masters	
London	Royal Marsden Hospital (London)	Nicholas James	
London	Guy's Hospital (London)	Peter Harper	
London	University College Hospital	Richard Kaplan	Co-I
Maidstone	Maidstone Hospital	Amanda Clarke	
Maidstone	Maidstone Hospital	Claire Baldry	
Maidstone	Maidstone Hospital	Delali Adjogatse	
Maidstone	Maidstone Hospital	Patryk Brulinski	PI
Maidstone	Maidstone Hospital	Alicia Synowiec	
Maidstone	Maidstone Hospital	Clary Evans	
Maidstone	Maidstone Hospital	Emma Kipps	
Maidstone	Maidstone Hospital	Henry Taylor	
Maidstone	Maidstone Hospital	Jess Brady	
Maidstone	Maidstone Hospital	Kathryn Lees	
Maidstone	Maidstone Hospital	Matthew Fittall	
Maidstone	Maidstone Hospital	Romaana Mir	
Maidstone	Maidstone Hospital	Sharon Beesley	
Maidstone	Maidstone Hospital	Amie Thomas	
Maidstone	Maidstone Hospital	Anna English	
Maidstone	Maidstone Hospital	Katy Taylor	
Maidstone	Maidstone Hospital	Gavin Fossey	
Maidstone	Maidstone Hospital	Ian Pamphlett	
Maidstone	Maidstone Hospital	Alison Davison	
Maidstone	Maidstone Hospital	Ann Phillips	
Maidstone	Maidstone Hospital	Jane Murray	
Maidstone	Maidstone Hospital	Louise Hooper-Gilham	
Maidstone	Maidstone Hospital	Alison Richards	
Maidstone	Maidstone Hospital	Carmel Jope	
Maidstone	Maidstone Hospital	Clare Calvert	
Maidstone	Maidstone Hospital	Jane Brown	

Maidstone	Maidstone Hospital	Julia Sunnucks	
Maidstone	Maidstone Hospital	Laura Clayton	
Maidstone	Maidstone Hospital	Verity Roberts	
Maidstone	Maidstone Hospital	Claudia Woodger	
Maidstone	Maidstone Hospital	Emma Craske	
Maidstone	Maidstone Hospital	Joanne Patterson	Pharmacist
Maidstone	Maidstone Hospital	Joanne Williams	
Maidstone	Maidstone Hospital	Lisa Tribe	
Maidstone	Maidstone Hospital	Sarah Martins	
Maidstone	Maidstone Hospital	Su Burrage	
Maidstone	Maidstone Hospital	Vivienne Breen	
Manchester	Christie Hospital	Andrew Hudson	
Manchester	Christie Hospital	Ather Kazmi	
Manchester	Christie Hospital	Christoph Oing	Co-I
Manchester	Christie Hospital	Hebalalla Abdelaal	
Manchester	Christie Hospital	Martin Swinton	
Manchester	Christie Hospital	Robin Portner	
Manchester	Christie Hospital	Sreeja Aruketty	
Manchester	Christie Hospital	Stefanie Fisder	
Manchester	Christie Hospital	Stephen Chin	
Manchester	Christie Hospital	Yee Pei Song	
Manchester	Christie Hospital	A Jegannathen	
Manchester	Christie Hospital	Ananya Choudhury	
Manchester	Christie Hospital	Anna Bruzzan	
Manchester	Christie Hospital	Anna Tran	
Manchester	Withington Hospital	Beatriz Duran Jimenez	Pharmacist
Manchester	Wythenshawe Hospital	Beatriz Duran Jimenez	
Manchester	Christie Hospital	Catherine Coyle	
Manchester	Christie Hospital	Clara Chan	
Manchester	Christie Hospital	David Thompson	
Manchester	Christie Hospital	Jacqueline Livsey	
Manchester	Christie Hospital	James Wylie	
Manchester	Withington Hospital	James Wylie	
Manchester	Christie Hospital	John Logue	

Manchester	Christie Hospital	Michael Braun	
Manchester	Christie Hospital	Richard Cowan	
Manchester	Christie Hospital	Ruth Conroy	Co-I
Manchester	Christie Hospital	Shaun Tolan	
Manchester	Christie Hospital	Silke Gillessen	
Manchester	Christie Hospital	Tony Elliott	
Manchester	Christie Hospital	You Yone	
Manchester	Christie Hospital	Amber Hart	
Manchester	Withington Hospital	Anna Gipson	
Manchester	Christie Hospital	Charlotte Heywood	
Manchester	Christie Hospital	Kim Fair	
Manchester	Christie Hospital	Laura Flanagan	
Manchester	Christie Hospital	Maria Petsa	
Manchester	Christie Hospital	Samah Mughal	
Manchester	Christie Hospital	Sarah Green	
Manchester	Christie Hospital	Sarah-Ellen Ellen (née McCarthy)	
Manchester	Withington Hospital	Tania Cutts	
Manchester	Christie Hospital	Willemijn Spoor	
Manchester	Christie Hospital	Alkesh Patel	
Manchester	Christie Hospital	Kamlesh Patel	
Manchester	Christie Hospital	Tony Elliott	
Manchester	Withington Hospital	A. Emara	
Manchester	Wythenshawe Hospital	A. Emara	
Manchester	Christie Hospital	Ali Al-Hashimi	Pharmacist
Manchester	Christie Hospital	Damian McCall	
Manchester	Withington Hospital	Damian McCall	
Manchester	Christie Hospital	Damian McCaul	
Manchester	Christie Hospital	Ekugbe Onogbe	
Manchester	Christie Hospital	Ekugbe Onoge	
Manchester	Christie Hospital	Ian Bottomley	
Manchester	Christie Hospital	Vijay Ramani	
Manchester	Withington Hospital	Vijay Ramani	Co-I
Manchester	Christie Hospital	Vijay Sangar	
Manchester	Withington Hospital	Vijay Sangar	PI

Manchester	Wythenshawe Hospital	Vijay Sangar	PI
Manchester	Withington Hospital	Helen Haydock	
Manchester	Withington Hospital	Vivienne Benson	
Manchester	Wythenshawe Hospital	Vivienne Benson	
Manchester	Christie Hospital	Kate O'Connor	
Manchester	Christie Hospital	Sue Davison	
Manchester	Withington Hospital	Annie Duffy	
Manchester	Wythenshawe Hospital	Annie Duffy	
Manchester	Christie Hospital	Catherine Pettersen	
Manchester	Wythenshawe Hospital	Claire McGuire	
Manchester	Withington Hospital	Humera Ahmed	
Manchester	Wythenshawe Hospital	Julie Fielding	
Manchester	Withington Hospital	Kathryn Fellows	
Manchester	Wythenshawe Hospital	Kathryn Slevin	
Manchester	Wythenshawe Hospital	Kirsty Melia	
Manchester	Withington Hospital	Linda Bailey	
Manchester	Wythenshawe Hospital	Linda Bailey	
Manchester	Christie Hospital	Lucy Worsley	
Manchester	Christie Hospital	Megan Bunce	
Manchester	Wythenshawe Hospital	Molly Bennett	
Manchester	Withington Hospital	Rebecca Corless	
Manchester	Christie Hospital	Roonak Nazari	
Manchester	Christie Hospital	salina tsui	
Manchester	Wythenshawe Hospital	Sarah Liptrott	
Manchester	Christie Hospital	Sharon Capper	
Manchester	Withington Hospital	Stephanie Hargreaves	
Manchester	Christie Hospital	Thiraviyam Elumalai	
Manchester	Christie Hospital	Trishna Uttamlal	
Manchester	Wythenshawe Hospital	Wendy Guest	
Manchester	Christie Hospital	Zhara Mahmood	
Manchester	Wythenshawe Hospital	Angela Chrisopoulou	
Manchester	Wythenshawe Hospital	Angela Gowrie	
Manchester	Christie Hospital	Anna Bowron	
Manchester	Christie Hospital	Catherine Redshaw	

Manchester	Withington Hospital	Catherine Redshaw	
Manchester	Christie Hospital	Cathryn James	
Manchester	Christie Hospital	Cathryn Jones	
Manchester	Christie Hospital	Emma Burke	
Manchester	Christie Hospital	Emma Lowther	
Manchester	Withington Hospital	Fiona Murtagh	
Manchester	Withington Hospital	Janet Smith	
Manchester	Christie Hospital	Jeanette Lyons	
Manchester	Christie Hospital	Joanne Oliver	
Manchester	Withington Hospital	Julie Bramley	
Manchester	Withington Hospital	Karen Robb	
Manchester	Withington Hospital	Lillian Partington	Pharmacist
Manchester	Withington Hospital	Lindsay Piper	
Manchester	Wythenshawe Hospital	Lindsay Piper	
Manchester	Withington Hospital	Lorraine Turner	
Manchester	Christie Hospital	Lydia Sutherland	Pharmacist
Manchester	Christie Hospital	Lynne Gilmore	
Manchester	Christie Hospital	Marie Woolley	
Manchester	Christie Hospital	Sarah-Ellen Smith	
Manchester	Christie Hospital	Sue Seifi	
Manchester	Christie Hospital	Susan Arrand	
Manchester	Withington Hospital	Tarnya Hulme	
Manchester	Withington Hospital	Thobekile Mthethwa	
Manchester	Wythenshawe Hospital	Thobekile Mthethwa	
Manchester	Withington Hospital	Tracey Platt	
Manchester	Wythenshawe Hospital	Tracey Platt	
Manchester	Christie Hospital	Noel Clarke	PI
Manchester	Christie Hospital	Jackie O'Dwyer	
Manchester	Christie Hospital	Viv Thomas	
Margate	Queen Elizabeth The Queen Mother Hospital	Albert Edwards	Co-I
Margate	Queen Elizabeth The Queen Mother Hospital	Charlotte Mott	
Margate	Queen Elizabeth The Queen Mother Hospital	Ifigenia Vasiliadou	Co-I
Margate	Queen Elizabeth The Queen Mother Hospital	Lavarniya Rajakumar	
Margate	Queen Elizabeth The Queen Mother Hospital	Mathini Sridharan	

Margate	Queen Elizabeth The Queen Mother Hospital	Patryk Brulinski	
Margate	Queen Elizabeth The Queen Mother Hospital	Rakesh Raman	Co-I
Margate	Queen Elizabeth The Queen Mother Hospital	Rohit Malde	
Margate	Queen Elizabeth The Queen Mother Hospital	Stephane Tankoua	
Margate	Queen Elizabeth The Queen Mother Hospital	Arafat Mizra	
Margate	Queen Elizabeth The Queen Mother Hospital	Carys Thomas	PI
Margate	Queen Elizabeth The Queen Mother Hospital	Clary Evans	
Margate	Queen Elizabeth The Queen Mother Hospital	Kannon Nathan	
Margate	Queen Elizabeth The Queen Mother Hospital	Kathryn Lees	
Margate	Queen Elizabeth The Queen Mother Hospital	Mathilda Cominos	
Margate	Queen Elizabeth The Queen Mother Hospital	Matthew Fenton	
Margate	Queen Elizabeth The Queen Mother Hospital	Mohammed Osman	
Margate	Queen Elizabeth The Queen Mother Hospital	Natasha Mithal	Co-I
Margate	Queen Elizabeth The Queen Mother Hospital	Sharon Beesley	
Margate	Queen Elizabeth The Queen Mother Hospital	Sugeeta Sukumar	
Margate	Queen Elizabeth The Queen Mother Hospital	Udaiveer Panwar	
Margate	Queen Elizabeth The Queen Mother Hospital	Coral Greenstreet	
Margate	Queen Elizabeth The Queen Mother Hospital	Hayley Blackgrove	
Margate	Queen Elizabeth The Queen Mother Hospital	Katy Taylor	
Margate	Queen Elizabeth The Queen Mother Hospital	Victoria Williamson	
Margate	Queen Elizabeth The Queen Mother Hospital	Natalie Catt	
Margate	Queen Elizabeth The Queen Mother Hospital	Arafat Mirza	
Margate	Queen Elizabeth The Queen Mother Hospital	Sam Gibson	
Margate	Queen Elizabeth The Queen Mother Hospital	Steve Dann	
Margate	Queen Elizabeth The Queen Mother Hospital	Andrew Gillian	Pharmacist
Margate	Queen Elizabeth The Queen Mother Hospital	Miguel Capo-Mir	Pharmacist
Margate	Queen Elizabeth The Queen Mother Hospital	Cindy Slater	
Margate	Queen Elizabeth The Queen Mother Hospital	Hasmath Marjolin	
Margate	Queen Elizabeth The Queen Mother Hospital	Nikki Crisp	
Margate	Queen Elizabeth The Queen Mother Hospital	Rachel Larkins	
Margate	Queen Elizabeth The Queen Mother Hospital	Sandra Holness	
Margate	Queen Elizabeth The Queen Mother Hospital	Sarah Lines	
Margate	Queen Elizabeth The Queen Mother Hospital	Susan Rogers	
Margate	Queen Elizabeth The Queen Mother Hospital	Claire White	

Margate	Queen Elizabeth The Queen Mother Hospital	Julie Buckley	
Margate	Queen Elizabeth The Queen Mother Hospital	Laura Kehoe	
Margate	Queen Elizabeth The Queen Mother Hospital	Lesley Rose	
Margate	Queen Elizabeth The Queen Mother Hospital	Louise Gladwell	
Margate	Queen Elizabeth The Queen Mother Hospital	Sarah Lightfoot	Pharmacist
Margate	Queen Elizabeth The Queen Mother Hospital	Tracy Boakes	
Margate	Queen Elizabeth The Queen Mother Hospital	Alba Tubau	
Margate	Queen Elizabeth The Queen Mother Hospital	Bonny Appleby	
Margate	Queen Elizabeth The Queen Mother Hospital	Jo Williams	
Margate	Queen Elizabeth The Queen Mother Hospital	Linda Wray	Pharmacist
Margate	Queen Elizabeth The Queen Mother Hospital	Louise Allen	
Margate	Queen Elizabeth The Queen Mother Hospital	Marian Wood	
Margate	Queen Elizabeth The Queen Mother Hospital	Adedolapo Sanni	
Margate	Queen Elizabeth The Queen Mother Hospital	Claire Pelham	
Margate	Queen Elizabeth The Queen Mother Hospital	Elizabeth Williamson	
Margate	Queen Elizabeth The Queen Mother Hospital	Hilary Zurakovsky	
Margate	Queen Elizabeth The Queen Mother Hospital	Jill Baker	
Margate	Queen Elizabeth The Queen Mother Hospital	Joanne Williams	
Margate	Queen Elizabeth The Queen Mother Hospital	Julie-Ann Davies	
Margate	Queen Elizabeth The Queen Mother Hospital	Karen Robinson	
Margate	Queen Elizabeth The Queen Mother Hospital	Kathleen (Kathy) Walsh	
Margate	Queen Elizabeth The Queen Mother Hospital	Kim Mears	
Margate	Queen Elizabeth The Queen Mother Hospital	Kim Travis	
Margate	Queen Elizabeth The Queen Mother Hospital	Margaret Lipsham	
Margate	Queen Elizabeth The Queen Mother Hospital	Paula Whichelo	
Margate	Queen Elizabeth The Queen Mother Hospital	Sharon Middleton	
Margate	Queen Elizabeth The Queen Mother Hospital	Sue Kelly	
Margate	Queen Elizabeth The Queen Mother Hospital	Susan Drakeley	
Margate	Queen Elizabeth The Queen Mother Hospital	Sydnie Loveland	
Margate	Queen Elizabeth The Queen Mother Hospital	Molua Young	
Margate	Queen Elizabeth The Queen Mother Hospital	Denise Crawford	
Merseyside	Southport and Formby District General Hospital	Julie Griffiths	
Middlesborough	James Cook University Hospital	Julia McBride	
Middlesborough	James Cook University Hospital	Fiona Rowling	Pharmacist

Middlesbrough	James Cook University Hospital	Cheng Lee Chaw	
Middlesbrough	James Cook University Hospital	Maha Zarroug	
Middlesbrough	James Cook University Hospital	Mohammed Kagzi	
Middlesbrough	James Cook University Hospital	Clive Peedell	PI
Middlesbrough	James Cook University Hospital	David Wilson	
Middlesbrough	James Cook University Hospital	Devadasan Shakespeare	
Middlesbrough	James Cook University Hospital	Hans Van der Voet	
Middlesbrough	James Cook University Hospital	Jason Wong	
Middlesbrough	James Cook University Hospital	John Hardman	
Middlesbrough	James Cook University Hospital	Steven Pratt	
Middlesbrough	James Cook University Hospital	Anne Hardwick	Pharmacist
Middlesbrough	James Cook University Hospital	Emma Pringleton	
Middlesbrough	James Cook University Hospital	Jo Atkinson	
Middlesbrough	James Cook University Hospital	Joanne Atkinson	
Middlesbrough	James Cook University Hospital	Kate Rees	
Middlesbrough	James Cook University Hospital	Michaela Davenport	
Middlesbrough	James Cook University Hospital	Michaela Devenport	
Middlesbrough	James Cook University Hospital	Rebecca Richards	
Middlesbrough	James Cook University Hospital	Andrew Vaux	
Middlesbrough	James Cook University Hospital	Craig Mower	
Middlesbrough	James Cook University Hospital	Luca Settimo	
Middlesbrough	James Cook University Hospital	Piers Loxley Winder	
Middlesbrough	James Cook University Hospital	Piers Winders	
Middlesbrough	James Cook University Hospital	David Chadwick	
Middlesbrough	James Cook University Hospital	Keith Harland	
Middlesbrough	James Cook University Hospital	Paul Jones-King	
Middlesbrough	James Cook University Hospital	Alison Barnes	
Middlesbrough	James Cook University Hospital	Claire Elliott	
Middlesbrough	James Cook University Hospital	Emanuela Mahmoud	
Middlesbrough	James Cook University Hospital	Alison Chilvers	
Middlesbrough	James Cook University Hospital	Carol Long	
Middlesbrough	James Cook University Hospital	Helen Carver	
Middlesbrough	James Cook University Hospital	Jane Thompson	
Middlesbrough	James Cook University Hospital	Julie Potts	

Middlesbrough	James Cook University Hospital	Lisa Peacock (nee Wayman)	
Middlesbrough	James Cook University Hospital	Paula Milne	
Middlesbrough	James Cook University Hospital	Emma Thompson	
Middlesbrough	James Cook University Hospital	Helen Dunn	Pharmacist
Middlesbrough	James Cook University Hospital	Katherine Tyler	
Middlesbrough	James Cook University Hospital	Lynne Naylor	
Middlesbrough	James Cook University Hospital	Sarah Kiddell	
Middlesbrough	James Cook University Hospital	Sarah McAuliffe	
Middlesbrough	James Cook University Hospital	Agnieszka Skotnicka	Pharmacist
Middlesbrough	James Cook University Hospital	Andrea Watson	
Middlesbrough	James Cook University Hospital	Charlotte Jacobs(née Kitching)	
Middlesbrough	James Cook University Hospital	Rita Mohan	
Middlesbrough	James Cook University Hospital	Vicky Hanlon	Pharmacist
Newcastle upon Tyne	Newcastle General Hospital	Judith Moore	
Newcastle-upon-Tyne	Freeman Hospital	Alex Mitchell	
Newcastle-upon-Tyne	Freeman Hospital	Elle Cameron	
Newcastle-upon-Tyne	Freeman Hospital	John Frew	Co-I
Newcastle-upon-Tyne	Freeman Hospital	Nicola Hannaway	
Newcastle-upon-Tyne	Freeman Hospital	Noor Harris	
Newcastle-upon-Tyne	Freeman Hospital	Robert Chandler	
Newcastle-upon-Tyne	Freeman Hospital	Shahid Iqbal	Co-I
Newcastle-upon-Tyne	Freeman Hospital	Ashraf Azzabi	PI
Newcastle-upon-Tyne	Freeman Hospital	Ian Pedley	Co-I
Newcastle-upon-Tyne	Freeman Hospital	Rhona McMenemin	Co-I
Newcastle-upon-Tyne	Freeman Hospital	Amanda Henderson	
Newcastle-upon-Tyne	Freeman Hospital	Catherine Marsh	
Newcastle-upon-Tyne	Freeman Hospital	Emma King	
Newcastle-upon-Tyne	Freeman Hospital	Gemma O'Neill	
Newcastle-upon-Tyne	Freeman Hospital	Georgia Ross	
Newcastle-upon-Tyne	Freeman Hospital	Hannah Downs	
Newcastle-upon-Tyne	Freeman Hospital	Janine Tate	
Newcastle-upon-Tyne	Freeman Hospital	Katie Bain	
Newcastle-upon-Tyne	Freeman Hospital	Lucy Blackwell	
Newcastle-upon-Tyne	Freeman Hospital	Marianne Smith	

Newcastle-upon-Tyne	Freeman Hospital	Victoria Thomas	
Newcastle-upon-Tyne	Freeman Hospital	Andrew Herridge	
Newcastle-upon-Tyne	Freeman Hospital	Craig Alderson	Pharmacist
Newcastle-upon-Tyne	Freeman Hospital	Edgar Paez	
Newcastle-upon-Tyne	Freeman Hospital	Gerard Oakes	
Newcastle-upon-Tyne	Freeman Hospital	Ian Campbell	Pharmacist
Newcastle-upon-Tyne	Freeman Hospital	Roger Carr	
Newcastle-upon-Tyne	Freeman Hospital	Thomas Jarvis	
Newcastle-upon-Tyne	Freeman Hospital	Ben Hood	
Newcastle-upon-Tyne	Freeman Hospital	Mark Johnson	
Newcastle-upon-Tyne	Freeman Hospital	Naeem Soomro	
Newcastle-upon-Tyne	Freeman Hospital	Peter Murphy	
Newcastle-upon-Tyne	Freeman Hospital	Dianne Turner	
Newcastle-upon-Tyne	Freeman Hospital	Elizabeth Reay	
Newcastle-upon-Tyne	Freeman Hospital	Kristine Hawkins	
Newcastle-upon-Tyne	Freeman Hospital	Ruth Latter	
Newcastle-upon-Tyne	Freeman Hospital	Sarah Osborne	
Newcastle-upon-Tyne	Freeman Hospital	Sarah Rowling	
Newcastle-upon-Tyne	Freeman Hospital	Sarah Wright	
Newcastle-upon-Tyne	Freeman Hospital	Nichola Waugh	
Newcastle-upon-Tyne	Freeman Hospital	Ann Hudson	
Newcastle-upon-Tyne	Freeman Hospital	Caroline Dobeson	
Newcastle-upon-Tyne	Freeman Hospital	Diane Conner	
Newcastle-upon-Tyne	Freeman Hospital	Diane Connor	
Newcastle-upon-Tyne	Freeman Hospital	Dianne Wake	
Newcastle-upon-Tyne	Freeman Hospital	Elaine Greaves	
Newcastle-upon-Tyne	Freeman Hospital	Hazel Forsyth	
Newcastle-upon-Tyne	Freeman Hospital	Hazel Masson	
Newcastle-upon-Tyne	Freeman Hospital	Irene Jobson	
Newcastle-upon-Tyne	Freeman Hospital	Jenny Smith	
Newcastle-upon-Tyne	Freeman Hospital	Julie Thohig	
Newcastle-upon-Tyne	Freeman Hospital	Laura Jameson	
Newcastle-upon-Tyne	Freeman Hospital	Lauren Boal	
Newcastle-upon-Tyne	Freeman Hospital	Lavanya Mariappan	

Newcastle-upon-Tyne	Freeman Hospital	Penny Bradley	
Newcastle-upon-Tyne	Freeman Hospital	Ruth Sinnott	
Newcastle-upon-Tyne	Freeman Hospital	Sue Farrell	
Newcastle-upon-Tyne	Freeman Hospital	Sunita Kholi	
Newcastle-upon-Tyne	Freeman Hospital	Sunita Kollu	
Newcastle-upon-Tyne	Freeman Hospital	Xue Jiang	
Newcastle-upon-Tyne	Freeman Hospital	Carole Stobbart	
Newcastle-upon-Tyne	Freeman Hospital	Kay Carson	Pharmacist
Newcastle-upon-Tyne	Freeman Hospital	Lesley Naik	
Newport	St Mary's Hospital (Newport)	Kudingila Madhava	
Newport	St Mary's Hospital (Newport)	Alison Brown	
Newport	St Mary's Hospital (Newport)	Cindy Whitbread	
Newport	St Mary's Hospital (Newport)	Tracey Tidbury	
North Shields	North Tyneside General Hospital	Mark Johnson	
Northampton	Northampton General Hospital	Rachel Gabitass	
Northwood	Mount Vernon Hospital	Neel Bhuva	
Northwood	Mount Vernon Hospital	Adam Mitchell	Co-I
Northwood	Mount Vernon Hospital	Charlotte Westbury	
Northwood	Mount Vernon Hospital	David Woolf	
Northwood	Mount Vernon Hospital	Hamoun Rozati	Co-I
Northwood	Mount Vernon Hospital	Hannah Tharmalingam	
Northwood	Mount Vernon Hospital	Janaka Cooray	
Northwood	Mount Vernon Hospital	Joanne Kosmin	
Northwood	Mount Vernon Hospital	Lai Cheng Yew	
Northwood	Mount Vernon Hospital	Mohammed Abdul-Latif	
Northwood	Mount Vernon Hospital	Russell Moule	
Northwood	Mount Vernon Hospital	Sara Kashani	Co-I
Northwood	Mount Vernon Hospital	Claire Zane	
Northwood	Mount Vernon Hospital	Dolan Basak	
Northwood	Mount Vernon Hospital	Henry Mandeville	
Northwood	Mount Vernon Hospital	Huiqi Yang	
Northwood	Mount Vernon Hospital	Jeanette Dickson	
Northwood	Mount Vernon Hospital	Jennifer Chard	
Northwood	Mount Vernon Hospital	Kasia Owczarczyk	

Northwood	Mount Vernon Hospital	Katie Wood	
Northwood	Mount Vernon Hospital	Kent Yip	
Northwood	Mount Vernon Hospital	Lai-Cheng Yew	
Northwood	Mount Vernon Hospital	Linda Swaney	
Northwood	Mount Vernon Hospital	M Williams	
Northwood	Mount Vernon Hospital	Mausam Singhera	
Northwood	Mount Vernon Hospital	Nicola Anyamene	
Northwood	Mount Vernon Hospital	Olivia Hatcher	
Northwood	Mount Vernon Hospital	Paolo De Jesu	
Northwood	Mount Vernon Hospital	Peter Ostler	
Northwood	Mount Vernon Hospital	Rachael Khong	
Northwood	Mount Vernon Hospital	Robert Hughes	
Northwood	Mount Vernon Hospital	Roberto Alonzi	
Northwood	Mount Vernon Hospital	Shaista Harpeer	
Northwood	Mount Vernon Hospital	Shiv Gayadeen	
Northwood	Mount Vernon Hospital	Sonia Li	
Northwood	Mount Vernon Hospital	Viwod Mullassery	PI
Northwood	Mount Vernon Hospital	Aamna Rashid	
Northwood	Mount Vernon Hospital	Freya Ball	
Northwood	Mount Vernon Hospital	Harmeet Sangha	
Northwood	Mount Vernon Hospital	Juhee Kong	
Northwood	Mount Vernon Hospital	Lucy Collins	
Northwood	Mount Vernon Hospital	Rose Bell	
Northwood	Mount Vernon Hospital	Tahmina Shakil	
Northwood	Mount Vernon Hospital	Alice Ramsden	
Northwood	Mount Vernon Hospital	David Tan	
Northwood	Mount Vernon Hospital	Farhan Ahmed	Pharmacist
Northwood	Mount Vernon Hospital	Sam Bosompem	Pharmacist
Northwood	Mount Vernon Hospital	Heidi Rana	
Northwood	Mount Vernon Hospital	Jessica Finch	
Northwood	Mount Vernon Hospital	Kari Evans	
Northwood	Mount Vernon Hospital	Nalini Shah	
Northwood	Mount Vernon Hospital	Rakhi Jain	Pharmacist
Northwood	Mount Vernon Hospital	Shakeda Lakha	

Northwood	Mount Vernon Hospital	Bhanthi Kanagaratnam	
Northwood	Mount Vernon Hospital	Hannah Phillips	
Northwood	Mount Vernon Hospital	Harsha Vara	Pharmacist
Northwood	Mount Vernon Hospital	Helen Cladd	
Northwood	Mount Vernon Hospital	Justina Kailey	
Northwood	Mount Vernon Hospital	Lesley Mitchell	
Northwood	Mount Vernon Hospital	Nazma Damani	
Northwood	Mount Vernon Hospital	Nicola Cutmore	
Northwood	Mount Vernon Hospital	Paulina Kowalewska	
Northwood	Mount Vernon Hospital	Sapna Kaur	
Northwood	Mount Vernon Hospital	Elaine Lousley	
Northwood	Mount Vernon Hospital	Hameeda Sultany	Pharmacist
Northwood	Mount Vernon Hospital	Jessica Milner	
Northwood	Mount Vernon Hospital	Sara Abbassi	
Northwood	Mount Vernon Hospital	Suzanne Jenkins	
Northwood	Mount Vernon Hospital	Peter Hoskin	PI
Nottingham	Nottingham University Hospitals, City Campus	Adam Fullagar	
Nottingham	Nottingham University Hospitals, City Campus	Charlotte Kamlow	
Nottingham	Nottingham University Hospitals, City Campus	Chin Chong	
Nottingham	Nottingham University Hospitals, City Campus	Ewan Shawcroft	
Nottingham	Nottingham University Hospitals, City Campus	Junhao Lim	Co-I
Nottingham	Nottingham University Hospitals, City Campus	Lauren Jones	
Nottingham	Nottingham University Hospitals, City Campus	Maeve Pomeroy	
Nottingham	Nottingham University Hospitals, City Campus	Melanie Boafo-Yirenkyi	
Nottingham	Nottingham University Hospitals, City Campus	Rebekah Webb	
Nottingham	Nottingham University Hospitals, City Campus	Rohan Tharaka	Co-I
Nottingham	Nottingham University Hospitals, City Campus	Rohan Tharakan	
Nottingham	Nottingham University Hospitals, City Campus	Sadia Abdullah	Co-I
Nottingham	Nottingham University Hospitals, City Campus	Sarah Taylor	
Nottingham	Nottingham University Hospitals, City Campus	Shiv Uppal	
Nottingham	Nottingham University Hospitals, City Campus	Thomas Moore	
Nottingham	Nottingham University Hospitals, City Campus	Wai Hou Sam	
Nottingham	Nottingham University Hospitals, City Campus	Ananth Sivanandan	
Nottingham	Nottingham University Hospitals, City Campus	Charlotte Ellis	

Nottingham	Nottingham University Hospitals, City Campus	Daniel Saunders	
Nottingham	Nottingham University Hospitals, City Campus	Eliot Chadwick	Co-I
Nottingham	Nottingham University Hospitals, City Campus	Georgina Walker	Co-I
Nottingham	Nottingham University Hospitals, City Campus	Ian Sayers	
Nottingham	Nottingham University Hospitals, City Campus	Jamie Mills	
Nottingham	Nottingham University Hospitals, City Campus	Louise Brookes	
Nottingham	Nottingham University Hospitals, City Campus	Santhanam Sundar	PI
Nottingham	Nottingham University Hospitals, City Campus	Adele Malson	
Nottingham	Nottingham University Hospitals, City Campus	Asmaa Sa Omer	
Nottingham	Nottingham University Hospitals, City Campus	Camille Hutchinson	
Nottingham	Nottingham University Hospitals, City Campus	Hannah Thurlow	
Nottingham	Nottingham University Hospitals, City Campus	Jade Eggleton	
Nottingham	Nottingham University Hospitals, City Campus	Kayleigh Mills	
Nottingham	Nottingham University Hospitals, City Campus	Lucy Howard	
Nottingham	Nottingham University Hospitals, City Campus	Leanne Alder	
Nottingham	Nottingham University Hospitals, City Campus	Alex Blades	
Nottingham	Nottingham University Hospitals, City Campus	Jacob Szolin-Jones	
Nottingham	Nottingham University Hospitals, City Campus	Daniel Kumar	
Nottingham	Nottingham University Hospitals, City Campus	Matthew Brazkiewicz	
Nottingham	Nottingham University Hospitals, City Campus	Owen Cole	
Nottingham	Nottingham University Hospitals, City Campus	Tin Sang-Tsang	Pharmacist
Nottingham	Nottingham University Hospitals, City Campus	Anita Stevenson	Pharmacist
Nottingham	Nottingham University Hospitals, City Campus	Katie Carter	
Nottingham	Nottingham University Hospitals, City Campus	Phillipa Sum	
Nottingham	Nottingham University Hospitals, City Campus	Samantha Chetiyawardana	
Nottingham	Nottingham University Hospitals, City Campus	Tania Slater	
Nottingham	Nottingham University Hospitals, City Campus	Carol Gooch	
Nottingham	Nottingham University Hospitals, City Campus	Kathryn Moore	
Nottingham	Nottingham University Hospitals, City Campus	Susan Elliott	
Nottingham	Nottingham University Hospitals, City Campus	Cody Jevons	
Nottingham	Nottingham University Hospitals, City Campus	Rachael Chivers	
Nottingham	Nottingham University Hospitals, City Campus	Sarah Widdowson	
Nottingham	Nottingham University Hospitals, City Campus	Stephanie McGonagle	
Nottingham	Nottingham University Hospitals, City Campus	Caitlin Todd	

Nottingham	Nottingham University Hospitals, City Campus	Rena Chauhan	Pharmacist
Nottingham	Nottingham University Hospitals, City Campus	Stacey Green	
Nuneaton	George Eliot Hospital	Andrew White	
Nuneaton	George Eliot Hospital	Ellanna Guithi	
Nuneaton	George Eliot Hospital	Michael Tilby	Co-I
Nuneaton	George Eliot Hospital	Andrew Chan	
Nuneaton	George Eliot Hospital	Yakhub Khan	PI
Nuneaton	George Eliot Hospital	Damilola Jayeoba	Pharmacist
Nuneaton	George Eliot Hospital	Holly Lawrence	
Nuneaton	George Eliot Hospital	Kerry Flahive	
Nuneaton	George Eliot Hospital	sara Taylor	
Nuneaton	George Eliot Hospital	Arandeep Hayer	
Nuneaton	George Eliot Hospital	Albert Mislang	
Nuneaton	George Eliot Hospital	Jacob Bourne	
Nuneaton	George Eliot Hospital	Andrea Mills	
Nuneaton	George Eliot Hospital	Inderjit Atwal	
Nuneaton	George Eliot Hospital	Jenna Williams	
Nuneaton	George Eliot Hospital	Alison McCallum	
Nuneaton	George Eliot Hospital	Jessica Gunn	
Nuneaton	George Eliot Hospital	Karen Shorthose	
Nuneaton	George Eliot Hospital	Michaela Hill	
Nuneaton	George Eliot Hospital	Pritpal Klear	
Nuneaton	George Eliot Hospital	Rachael Oates	
Nuneaton	George Eliot Hospital	Rachel Fergusson	
Nuneaton	George Eliot Hospital	Sarah Fergusson	
Nuneaton	George Eliot Hospital	Winni Singh	
Nuneaton	George Eliot Hospital	Jeanette Knapp	
Nuneaton	George Eliot Hospital	Melanie Taylor	Pharmacist
Nuneaton	George Eliot Hospital	Sabiya Nasima	
Nuneaton	George Eliot Hospital	Judith Lake	
Oldham	Royal Oldham Hospital	Agata Rembielak	
Oldham	Royal Oldham Hospital	Anthea Cree	
Oldham	Royal Oldham Hospital	Ehab Ibrahim	Co-I
Oldham	Royal Oldham Hospital	Helen Joyce	

Oldham	Royal Oldham Hospital	Hwoeifen Soohoo	
Oldham	Royal Oldham Hospital	Kanal Gupta	
Oldham	Royal Oldham Hospital	Mohammad Abutarb	
Oldham	Royal Oldham Hospital	Parth Desai	Co-I
Oldham	Royal Oldham Hospital	Richard Walshaw	
Oldham	Royal Oldham Hospital	Shabaz Hussain	
Oldham	Royal Oldham Hospital	Shazril Imran Shaukat	
Oldham	Royal Oldham Hospital	Stephen Kennedy	
Oldham	Royal Oldham Hospital	Victoria Lavin	
Oldham	Royal Oldham Hospital	Ananya Choudhury	Co-I
Oldham	Royal Oldham Hospital	Anna Tran	Co-I
Oldham	Royal Oldham Hospital	Jacqueline Livsey	
Oldham	Royal Oldham Hospital	Peter Mbanu	
Oldham	Royal Oldham Hospital	Ruth Conroy	PI
Oldham	Royal Oldham Hospital	Shaveta Mehta	Co-I
Oldham	Royal Oldham Hospital	Dellesa Robinson	
Oldham	Royal Oldham Hospital	Sophie Hovenden	
Oldham	Royal Oldham Hospital	Mark Livingstone	Pharmacist
Oldham	Royal Oldham Hospital	Farhan Karim	
Oldham	Royal Oldham Hospital	Richard Jones	
Oldham	Royal Oldham Hospital	Terence Hinton	
Oldham	Royal Oldham Hospital	Sarah Warran	
Oldham	Royal Oldham Hospital	Udeme Ohia	Pharmacist
Oldham	Royal Oldham Hospital	Leena Mistry	
Oldham	Royal Oldham Hospital	Amy Slack	
Oldham	Royal Oldham Hospital	Anna Pracz	Pharmacist
Oldham	Royal Oldham Hospital	Dawn Johnstone	
Oldham	Royal Oldham Hospital	Joanne Allsop	
Oldham	Royal Oldham Hospital	Joanne Johnson	
Oldham	Royal Oldham Hospital	Joanne Reed	
Oldham	Royal Oldham Hospital	Kamala Ramatar	
Oldham	Royal Oldham Hospital	Kirstie Smith	
Oldham	Royal Oldham Hospital	Lyndsay Scarratt	
Oldham	Royal Oldham Hospital	Ruth Halford	

Oldham	Royal Oldham Hospital	Suzanne Bland	
Oldham	Royal Oldham Hospital	Wendy Cook	
Oldham	Royal Oldham Hospital	Hadia Ashraf	
Oldham	Royal Oldham Hospital	Jemma McLaughlin	Pharmacist
Oxford	Churchill Hospital	Ami Sabharwal	Co-I
Oxford	Churchill Hospital	Avinash Gupta	Co-I
Oxford	Churchill Hospital	Katherine Hyde	Co-I
Oxford	Churchill Hospital	Laura Robledo	
Oxford	Churchill Hospital	Silke Hahnewald	
Oxford	Churchill Hospital	Simon Wyatt	
Oxford	Churchill Hospital	Tessa Greenhalgh	
Oxford	Churchill Hospital	Thinn Pwint	
Oxford	Churchill Hospital	Benjamin Fairfax	Co-I
Oxford	Churchill Hospital	David J Cole	Co-I
Oxford	Churchill Hospital	Elaine Sugden	
Oxford	Churchill Hospital	Gerard Andrade	Co-I
Oxford	Churchill Hospital	Mark Prentice	
Oxford	Churchill Hospital	Meenali Chitnis	Co-I
Oxford	Churchill Hospital	Paul Colin Miller	
Oxford	Churchill Hospital	Philip Camilleri	Co-I
Oxford	Churchill Hospital	Robert Stuart	Co-I
Oxford	Churchill Hospital	Robert Watson	
Oxford	Churchill Hospital	Elizabeth Hadley	
Oxford	Churchill Hospital	Kelly Wigglesworth	
Oxford	Churchill Hospital	Lauren Booker	
Oxford	Churchill Hospital	Patrycja Jastrzebska	
Oxford	Churchill Hospital	Sophia Shahzad	
Oxford	Churchill Hospital	Swapna Thummala	
Oxford	Churchill Hospital	Sywlia Bekulart	
Oxford	Churchill Hospital	Daniel Ajzensztejn	Co-I
Oxford	Churchill Hospital	Henry Chesson	
Oxford	Churchill Hospital	Hugo De La Pena	
Oxford	Churchill Hospital	Mark Tuthill	
Oxford	Churchill Hospital	Naveen Sankighatta	

Oxford	Churchill Hospital	Richard Cousins	
Oxford	Churchill Hospital	Will Goodman	
Oxford	Churchill Hospital	Matthew Mooney	
Oxford	Churchill Hospital	Martha Woodward	
Oxford	Churchill Hospital	Rachel Hart	
Oxford	Churchill Hospital	Rosita Broderick	
Oxford	Churchill Hospital	Ann Murphy	
Oxford	Churchill Hospital	Charlotte Davies	
Oxford	Churchill Hospital	Jo Wilson	
Oxford	Churchill Hospital	Kerrie Marston	
Oxford	Churchill Hospital	Julie Pinder	
Oxford	Churchill Hospital	Katherine Jacob	Pharmacist
Oxford	Churchill Hospital	Magdalena Benysek	
Oxford	Churchill Hospital	Sandra Mukkath	
Oxford	Churchill Hospital	Sarah Markus	
Oxford	Churchill Hospital	Ana De Veciana	Pharmacist
Oxford	Churchill Hospital	Anju Chalin	
Oxford	Churchill Hospital	Evanthia Komninidou	
Oxford	Churchill Hospital	Gabriela Kuzmycha	
Oxford	Churchill Hospital	Gemma Austin (nee Glover)	
Oxford	Churchill Hospital	Hazel Wynn	
Oxford	Churchill Hospital	Jane Boutflower	
Oxford	Churchill Hospital	Sarah Lawrey	
Oxford	Churchill Hospital	Trish Green	
Oxford	Churchill Hospital	Usharani Devi Wahengbam	
Oxford	Churchill Hospital	Andrew Protheroe	PI
Paisley	Royal Alexandra Hospital	Tiago Rodrigues	
Poole	Poole Hospital	Fiona Mellor	
Poole	Poole Hospital	Joseph Davies	Co-I
Poole	Poole Hospital	Yogesh Nishchal	Co-I
Poole	Poole Hospital	Joe Davies	
Poole	Poole Hospital	Maxine Flubacher	
Poole	Poole Hospital	May Lwin	
Poole	Poole Hospital	Perric Crellin	Co-I

Poole	Poole Hospital	Sue Brock	PI
Poole	Poole Hospital	Becky Troke	
Poole	Poole Hospital	Felicity Clapp	
Poole	Poole Hospital	Sarah Patch	
Poole	Poole Hospital	Elizabeth Woodward	
Poole	Poole Hospital	Nichola Downs	
Poole	Poole Hospital	Craig Vincent	
Poole	Poole Hospital	Neal Beamish	
Poole	Poole Hospital	Roger Wheelwright	
Poole	Poole Hospital	Deryck Burton	
Poole	Poole Hospital	Lee Tbaily	
Poole	Poole Hospital	Emma Wesley	
Poole	Poole Hospital	Emma Williams	
Poole	Poole Hospital	Helen Morling	
Poole	Poole Hospital	Kate Mutendera	
Poole	Poole Hospital	Kate Urquhart	
Poole	Poole Hospital	Louise Heckford	
Poole	Poole Hospital	Savina Elitova	Pharmacist
Poole	Poole Hospital	Delia Whiteman	
Poole	Poole Hospital	Elizabeth Clarke	
Poole	Poole Hospital	Lyn Jackson	
Poole	Poole Hospital	Sally Munden	
Poole	Poole Hospital	Sharon Power	Pharmacist
Poole	Poole Hospital	Sophie Rix	
Poole	Poole Hospital	Stephanie Jones	
Poole	Poole Hospital	Amanda Iskender	
Poole	Poole Hospital	Hilary Blaney	
Poole	Poole Hospital	Sally Gillespie	
Poole	Poole Hospital	Sandy Pressdee	
Poole	Poole Hospital	Sara Orford	
Poole	Poole Hospital	Seonaid Wright	
Poole	Poole Hospital	Susan Saxby	
Poole	Poole Hospital	Teresa Coffin	
Portadown	Craigavon Area Hospital	Fionnuala Houghton	Co-I

Portadown	Craigavon Area Hospital	Judith Carser	PI
Portadown	Craigavon Area Hospital	Leanne McCourt	
Portsmouth	Queen Alexandra Hospital	Akash Maniam	
Portsmouth	Queen Alexandra Hospital	Akash Maniam	
Portsmouth	Queen Alexandra Hospital	Alice White	
Portsmouth	Queen Alexandra Hospital	Alisha Damani	
Portsmouth	Queen Alexandra Hospital	Azarel Virgo	
Portsmouth	Queen Alexandra Hospital	Caroline Chau	
Portsmouth	Queen Alexandra Hospital	Charlotte Davies	
Portsmouth	Queen Alexandra Hospital	Chloe Holden	
Portsmouth	Queen Alexandra Hospital	Eleanor Jones	
Portsmouth	Queen Alexandra Hospital	Harliana Mohd Yusof	Co-I
Portsmouth	Queen Alexandra Hospital	Jack Broadfoot	
Portsmouth	Queen Alexandra Hospital	Jeng Heng Ching	
Portsmouth	Queen Alexandra Hospital	Joanna Hack	Co-I
Portsmouth	Queen Alexandra Hospital	Joni Howells	
Portsmouth	Queen Alexandra Hospital	Kudingila Madhava	
Portsmouth	Queen Alexandra Hospital	Mark Noble	
Portsmouth	Queen Alexandra Hospital	Matthew Moe	
Portsmouth	Queen Alexandra Hospital	Megan Rowley	
Portsmouth	Queen Alexandra Hospital	Mona Hassan	
Portsmouth	Queen Alexandra Hospital	Nataliya Martynyuk	
Portsmouth	Queen Alexandra Hospital	Oluwatobi Adeagbo	Co-I
Portsmouth	Queen Alexandra Hospital	Shyamkia Acharige	Co-I
Portsmouth	Queen Alexandra Hospital	Syed Shah	Co-I
Portsmouth	Queen Alexandra Hospital	Umapathy Hombaiah	Co-I
Portsmouth	Queen Alexandra Hospital	Vara Prasad Devara	
Portsmouth	Queen Alexandra Hospital	Victoria True	
Portsmouth	Queen Alexandra Hospital	Yvonne Mangan	
Portsmouth	Queen Alexandra Hospital	Joanna Gale	PI
Portsmouth	Queen Alexandra Hospital	Khalid Hameed	
Portsmouth	Queen Alexandra Hospital	Mario Uccello	
Portsmouth	Queen Alexandra Hospital	May Lwin	
Portsmouth	Queen Alexandra Hospital	Mya Gyi	

Portsmouth	Queen Alexandra Hospital	Sarah Ellis	
Portsmouth	Queen Alexandra Hospital	Yoodhvir Nagar	
Portsmouth	Queen Alexandra Hospital	Daniel Bloomfield	
Portsmouth	Queen Alexandra Hospital	Dominic Hodgson	
Portsmouth	Queen Alexandra Hospital	Robert Williams	
Portsmouth	Queen Alexandra Hospital	Jennifer Hale	
Portsmouth	Queen Alexandra Hospital	Jillian Andrews	
Portsmouth	Queen Alexandra Hospital	Tracey Dobson	
Portsmouth	Queen Alexandra Hospital	Tracy Callen	
Portsmouth	Queen Alexandra Hospital	Wendy Golding	Pharmacist
Portsmouth	Queen Alexandra Hospital	Wendy Stacey	
Portsmouth	Queen Alexandra Hospital	Catherine Tolentino	
Portsmouth	Queen Alexandra Hospital	Giuseppe Banna	Co-I
Portsmouth	Queen Alexandra Hospital	Heather Cuell	
Portsmouth	Queen Alexandra Hospital	Kathy Blight	Pharmacist
Portsmouth	Queen Alexandra Hospital	Lisa Murray	
Portsmouth	Queen Alexandra Hospital	Mila Roca	
Portsmouth	Queen Alexandra Hospital	Anna Stephenson	
Portsmouth	Queen Alexandra Hospital	Badrriyya Mohamedali	
Portsmouth	Queen Alexandra Hospital	Catrin Watkinson	
Portsmouth	Queen Alexandra Hospital	Lorna Meadows	
Portsmouth	Queen Alexandra Hospital	Mary Wands	Pharmacist
Postfach 834	Kantonsspital Winterthur	Claudia Langer	
Postfach 834	Kantonsspital Winterthur	Miklos Pless	
Postfach 834	Kantonsspital Winterthur	Natalie Fisher	
Postfach 834	Kantonsspital Winterthur	Sabina Schacher	
Postfach 834	Kantonsspital Winterthur	SusyAnn Shaw	
Postfach 834	Kantonsspital Winterthur	Veronika Nagy	
Postfach 834	Kantonsspital Winterthur		
Postfach 834	Kantonsspital Winterthur	Beatrice Brinkers	
Postfach 834	Kantonsspital Winterthur	Cindy Wanger	
Postfach 834	Kantonsspital Winterthur	Martina Pfitzner	
Postfach 834	Kantonsspital Winterthur	Nicole Kradolfer	
Postfach 834	Kantonsspital Winterthur	Hubert John	PI

Prescot Street	Royal Liverpool University Hospital	Lynsey Dean	
Preston	Royal Preston Hospital	Christina Hague	Co-I
Preston	Royal Preston Hospital	Jose Rico	Co-I
Preston	Royal Preston Hospital	Martin Swinton	
Preston	Royal Preston Hospital	Natalie Charnley	Co-I
Preston	Royal Preston Hospital	Nicola Flaum	Co-I
Preston	Royal Preston Hospital	Omi Parikh	Co-I
Preston	Royal Preston Hospital	Sophie Raby	Co-I
Preston	Royal Preston Hospital	William Croxford	
Preston	Royal Preston Hospital	Yee Pei Song	Co-I
Preston	Royal Preston Hospital	Alison Birtle	PI
Preston	Royal Preston Hospital	Anna Macpherson	
Preston	Royal Preston Hospital	Catherine Thompson	
Preston	Royal Preston Hospital	Deborah Williamson	
Preston	Royal Preston Hospital	Duleer Majeed	Co-I
Preston	Royal Preston Hospital	Falalu Danwata	
Preston	Royal Preston Hospital	Marcus Wise	Co-I
Preston	Royal Preston Hospital	Norma Sidek	
Preston	Royal Preston Hospital	Shahzad Gul	
Preston	Royal Preston Hospital	Tanmay Mukhopadhyay	
Preston	Royal Preston Hospital	Win Soe	
Preston	Royal Preston Hospital	Claire Corless	
Preston	Royal Preston Hospital	Rebecca Wilby (nee Hall)	
Preston	Royal Preston Hospital	Rose Ellard	
Preston	Royal Preston Hospital	Sarah Preston	
Preston	Royal Preston Hospital	Margaret Brunton	
Preston	Royal Preston Hospital	Andrew Martyniak	
Preston	Royal Preston Hospital	Davide Garau	
Preston	Royal Preston Hospital	Dominic Mounsey	
Preston	Royal Preston Hospital	Billy Hefferon	
Preston	Royal Preston Hospital	David Barber	Pharmacist
Preston	Royal Preston Hospital	Hemant Patel	
Preston	Royal Preston Hospital	Roy Shentall	
Preston	Royal Preston Hospital	Cassandra Gleeson	

Preston	Royal Preston Hospital	Deborah Weavers	
Preston	Royal Preston Hospital	Haiyan Huang	
Preston	Royal Preston Hospital	Mandy Armstrong	
Preston	Royal Preston Hospital	Nafisa Arden	
Preston	Royal Preston Hospital	Sharon Curran	
Preston	Royal Preston Hospital	Shelia Calvert	
Preston	Royal Preston Hospital	Andrea Ashton	
Preston	Royal Preston Hospital	Louise Hough	Pharmacist
Preston	Royal Preston Hospital	Catherine Walmsley	
Preston	Royal Preston Hospital	Christina Robinson	
Preston	Royal Preston Hospital	Deepsi Khatiwada	
Preston	Royal Preston Hospital	Hazel Preston	
Preston	Royal Preston Hospital	Amanda Alty	
Preston	Royal Preston Hospital	Caroline Hatch	
Preston	Royal Preston Hospital	Claire Hennigan	
Preston	Burnley General Hospital	Helen Spickett	
Preston	Royal Blackburn Hospital	Helen Spickett	
Preston	Royal Preston Hospital	Katherine Ashton	
Preston	Royal Preston Hospital	Nina Vekaria	
Preston	Royal Preston Hospital	Patricia Knight	
Preston	Royal Preston Hospital	Stephanie Cornthwaite	
Reading	Royal Berkshire Hospital	Elizabeth Haydon	
Reading	Royal Berkshire Hospital	Emilia Bruton	Co-I
Reading	Royal Berkshire Hospital	Gagan Bhatnagar	
Reading	Royal Berkshire Hospital	Nicola Dallas	
Reading	Royal Berkshire Hospital	Osamah Alasadi	
Reading	Royal Berkshire Hospital	Osamah Al-Asadi	Co-I
Reading	Royal Berkshire Hospital	Phillip Webb	
Reading	Royal Berkshire Hospital	Phillipa Johnstone	
Reading	Royal Berkshire Hospital	Rowena Cazalet	Co-I
Reading	Royal Berkshire Hospital	Silke Hahnewald	
Reading	Royal Berkshire Hospital	Abdolnasser Aminiraouf	
Reading	Royal Berkshire Hospital	Ali Abbas	
Reading	Royal Berkshire Hospital	Georges Sinclair	

Reading	Royal Berkshire Hospital	Helen O'Donnell	
Reading	Royal Berkshire Hospital	Paul Rogers	PI
Reading	Royal Berkshire Hospital	Rebecca Johnson	Co-I
Reading	Royal Berkshire Hospital	Rebecca Varatharajah	Co-I
Reading	Royal Berkshire Hospital	Richard B Brown	
Reading	Royal Berkshire Hospital	Robert Jones	
Reading	Royal Berkshire Hospital	Sean O'Cathail	
Reading	Royal Berkshire Hospital	Shawn Ellis	
Reading	Royal Berkshire Hospital	Simon Wyatt	
Reading	Royal Berkshire Hospital	Stephen Parr	
Reading	Royal Berkshire Hospital	James Church	
Reading	Royal Berkshire Hospital	Steven Gulliver	
Reading	Royal Berkshire Hospital	Thomas Kindley	
Reading	Royal Berkshire Hospital	Geraldine Mason	Pharmacist
Reading	Royal Berkshire Hospital	Kate Preston	
Reading	Royal Berkshire Hospital	Nicole Gould	
Reading	Royal Berkshire Hospital	Wioletta Kowalczyk-Williams	
Reading	Royal Berkshire Hospital	Christina Lewis	
Reading	Royal Berkshire Hospital	Pooja Pabari	
Reading	Royal Berkshire Hospital	Royda Hadi	
Reading	Royal Berkshire Hospital	Andreia da Cruz	
Reading	Royal Berkshire Hospital	Anna Gillham	
Reading	Royal Berkshire Hospital	Claire Connolly	
Reading	Royal Berkshire Hospital	Emma Vowell	
Reading	Royal Berkshire Hospital	Jo Hand	
Reading	Royal Berkshire Hospital	Maxine Gauntlett	
Reading	Royal Berkshire Hospital	Omotola Ogunnigbo	Pharmacist
Reading	Royal Berkshire Hospital	Sanita Gurm	
Reading	Royal Berkshire Hospital	Allison Hunt	
Reading	Royal Berkshire Hospital	Catherine Deytrikh-Smith	
Reading	Royal Berkshire Hospital	Debbie Cartwright	
Reading	Royal Berkshire Hospital	Fiona Everson	
Reading	Royal Berkshire Hospital	Gabrielle Ball	
Reading	Royal Berkshire Hospital	Helen Purdon	

Reading	Royal Berkshire Hospital	Jane Atkinson	
Reading	Royal Berkshire Hospital	Juliette Dye	
Reading	Royal Berkshire Hospital	Kirsty Horwood	Pharmacist
Reading	Royal Berkshire Hospital	Kristy Coomber	
Reading	Royal Berkshire Hospital	Maryam Amole	
Reading	Royal Berkshire Hospital	Norma Shields	Pharmacist
Reading	Royal Berkshire Hospital	Sian James	
Reading	Royal Berkshire Hospital	Suzanne Foxwell	
Reading	Royal Berkshire Hospital	Tolu Okeke	
Redditch	Alexandra Hospital	Ayyaz Munawar	Co-I
Redditch	Alexandra Hospital	Bartlomeij Kurec	
Redditch	Alexandra Hospital	Menna Fonda	Co-I
Redditch	Alexandra Hospital	Mujtaba Syed-Khaja	Co-I
Redditch	Alexandra Hospital	Nge Nge Thida	Co-I
Redditch	Alexandra Hospital	Asha Sivapalasuntharam	
Redditch	Alexandra Hospital	James Best	Co-I
Redditch	Alexandra Hospital	Joanna Hamilton	
Redditch	Alexandra Hospital	Lisa Capaldi	PI
Redditch	Alexandra Hospital	Paul Flinders	
Redditch	Alexandra Hospital	Sabihya Wontumi	Co-I
Redditch	Alexandra Hospital	Thakshayini Shanthakumar	Co-I
Redditch	Alexandra Hospital	Jacob Taylor	
Redditch	Alexandra Hospital	Jonathan Davies	
Redditch	Alexandra Hospital	Alison Harrison	
Redditch	Alexandra Hospital	Amanda Holdsworth	
Redditch	Alexandra Hospital	Ann White	
Redditch	Alexandra Hospital	Jayna Thakrar	
Redditch	Alexandra Hospital	Veronica Rowlands	
Redditch	Alexandra Hospital	Hayley Hodson	
Redditch	Alexandra Hospital	Helen Tranter	
Redditch	Alexandra Hospital	Maggie Brown	
Redditch	Alexandra Hospital	Stephanie Cook	Pharmacist
Redditch	Alexandra Hospital	Jennifer Young	Pharmacist
Redditch	Alexandra Hospital	Jeanette Knapp	

Redhill	East Surrey Hospital	Eva Letalova	
Romford	Queen's Hospital (Romford)	Alexander Pawsey	Co-I
Romford	Queen's Hospital (Romford)	Danny Koroma Koroma	
Romford	Queen's Hospital (Romford)	Kathryn Tarver	PI
Romford	Queen's Hospital (Romford)	Maria Martinou	PI
Romford	Queen's Hospital (Romford)	Jonathon Shamash	
Romford	Queen's Hospital (Romford)	Ramachandran Subramaniam	
Romford	Queen's Hospital (Romford)	Stephanie Gibbs	PI
Romford	Queen's Hospital (Romford)	Thi Vu	
Romford	Queen's Hospital (Romford)	Mohammed Rashid Khan	
Romford	Queen's Hospital (Romford)	Revanth Jannapureddy	
Romford	Queen's Hospital (Romford)	Samuel Mugari	
Romford	Queen's Hospital (Romford)	Simerjyot Mudhar	
Romford	Queen's Hospital (Romford)	Neale O'Brien	
Romford	Oldchurch Hospital	Neil Fisher	
Romford	Queen's Hospital (Romford)	Yousaf Razzak	
Romford	Queen's Hospital (Romford)	Parveen Dugh	Pharmacist
Romford	Queen's Hospital (Romford)	Amani Chowdhury	
Romford	Queen's Hospital (Romford)	Mariha Khalid	
Romford	Queen's Hospital (Romford)	Ana-Marie Pena-Remorin	
Romford	Queen's Hospital (Romford)	Dalisay Domingo	
Romford	Queen's Hospital (Romford)	Helen Mackenzie	
Romford	Queen's Hospital (Romford)	Tina Mills-Baldock	
Runcorn	Warrington Hospital	Isabel Syndikus	PI
Runcorn	Warrington Hospital	Shaun Tolan	Co-I
Runcorn	Halton Hospital	Duncan Knowles	
Runcorn	Halton Hospital	Ian Allen	
Runcorn	Halton Hospital	Andrea Young	
Runcorn	Halton Hospital	Rebecca Madew (nee Tinker)	
Runcorn	Halton Hospital	Nemonie Marriott	
Salford	Salford Royal Hospital	Nicholas Boxall	
Salford	Salford Royal Hospital	Anna Tran	Co-I
Salford	Salford Royal Hospital	Richard Cowan	
Salford	Salford Royal Hospital	Danielle Platt	

Salford	Salford Royal Hospital	Elina Jose	
Salford	Salford Royal Hospital	Joanne Henry	
Salford	Salford Royal Hospital	Kay Goulden	
Salford	Salford Royal Hospital	Euan Green	Co-I
Salford	Salford Royal Hospital	Maurice Lau	Co-I
Salford	Salford Royal Hospital	Oliver Wadsworth	
Salford	Salford Royal Hospital	Tony Elliott	Co-I
Salford	Salford Royal Hospital	Chris Betts	
Salford	Salford Royal Hospital	David Shackley	
Salford	Salford Royal Hospital	Jason Howard	Pharmacist
Salford	Salford Royal Hospital	Kieran O'Flynn	
Salford	Salford Royal Hospital	Mark Stapleton	
Salford	Salford Royal Hospital	Richard Jones	
Salford	Salford Royal Hospital	Christine Farnworth	
Salford	Salford Royal Hospital	Claire Duncan (nee Keatley)	Pharmacist
Salford	Salford Royal Hospital	Claire Keatley	
Salford	Salford Royal Hospital	Kathryn Cawley	
Salford	Salford Royal Hospital	Siny George	
Salford	Salford Royal Hospital	Cellins Vinod	
Salford	Salford Royal Hospital	Claire Dickson	
Salford	Salford Royal Hospital	Helen Farrell	
Salford	Salford Royal Hospital	Samia Hanif	
Salford	Salford Royal Hospital	Soney Dharmaprasad	
Salford	Salford Royal Hospital	Angela Ashton	
Salford	Salford Royal Hospital	Anne-Marie Peers	Pharmacist
Salford	Salford Royal Hospital	Ashley Harris	Pharmacist
Salford	Salford Royal Hospital	Catherine Redshaw	
Salford	Salford Royal Hospital	Jean Jellicoe	
Salford	Salford Royal Hospital	Kathryn Fry	
Salford	Salford Royal Hospital	Leah Harter	
Salford	Salford Royal Hospital	Rachael Allen	
Salford	Salford Royal Hospital	Vicky Thomas	
Salford	Salford Royal Hospital	Noel Clarke	PI
Salford	Salford Royal Hospital	Jill Youd	

Salford	Salford Royal Hospital	Melanie Taylor	
Salford	Salford Royal Hospital	Sarah Kirk	
Scarborough	Scarborough General Hospital	Ian Renwick	
Scarborough	Scarborough General Hospital	Khaliq Rehman	Co-I
Scarborough	Scarborough General Hospital	Laith Alsaket	
Scarborough	Scarborough General Hospital	Mohan Hingorani	PI
Scarborough	Scarborough General Hospital	Nabil El-Mahdawi	
Scarborough	Scarborough General Hospital	Mohammad Muneeb Khan	PI
Scarborough	Scarborough General Hospital	Richard Khafagy	
Scarborough	Scarborough General Hospital	Russell Morgan	
Scarborough	Scarborough General Hospital	Abigail Rowbotham	
Scarborough	Scarborough General Hospital	Caroline Savage	
Scarborough	Scarborough General Hospital	Diana Ionita	
Scarborough	Scarborough General Hospital	Fizzah Asif	
Scarborough	Scarborough General Hospital	Lydia Kerr	
Scarborough	Scarborough General Hospital	Poppy Cottrell-Howe	Pharmacist
Scarborough	Scarborough General Hospital	Courtney Cole	
Scarborough	Scarborough General Hospital	Donna Anderson	
Scarborough	Scarborough General Hospital	Rachel Spooner	
Scarborough	Scarborough General Hospital	Callum Childs	
Scarborough	Scarborough General Hospital	Dominic Burns	
Scarborough	Scarborough General Hospital	Andrew Robertson	
Scarborough	Scarborough General Hospital	Arran Fletcher	
Scarborough	Scarborough General Hospital	Jordan Toohie	
Scarborough	Scarborough General Hospital	Kevin Brame	
Scarborough	Scarborough General Hospital	Paul Wood	Pharmacist
Scarborough	Scarborough General Hospital	Simon Hawkyard	Co-I
Scarborough	Scarborough General Hospital	Jacqui Smith	
Scarborough	Scarborough General Hospital	Lisa Armitage	
Scarborough	Scarborough General Hospital	Samantha Stead	
Scarborough	Scarborough General Hospital	Tania Neale	
Scarborough	Scarborough General Hospital	Tanya Hartley	
Scarborough	Scarborough General Hospital	Alison Ames	
Scarborough	Scarborough General Hospital	Alison Turnbull	

Scarborough	Scarborough General Hospital	Anne Nunn	
Scarborough	Scarborough General Hospital	Joanne Fletcher	
Scarborough	Scarborough General Hospital	Rachel Harrison	
Scarborough	Scarborough General Hospital	Sacha Honour	Pharmacist
Scarborough	Scarborough General Hospital	Adnan Kabir	
Scarborough	Scarborough General Hospital	Alicia Rodgers	
Scarborough	Scarborough General Hospital	Janine Mallinson	
Scarborough	Scarborough General Hospital	Laura Barman	
Scarborough	Scarborough General Hospital	Pippa Carlton-Rylance	
Scarborough	Scarborough General Hospital	Polly Needs	
Scarborough	Scarborough General Hospital	Amie Stewart	
Scarborough	Scarborough General Hospital	Carol Popplestone	
Scarborough	Scarborough General Hospital	Cheryl Donne	Pharmacist
Scarborough	Scarborough General Hospital	Chloe Box	Pharmacist
Scarborough	Scarborough General Hospital	Jane Taylor	Pharmacist
Scarborough	Scarborough General Hospital	Sarah Kent	
Scarborough	Scarborough General Hospital	Tracey Hawkes	
Scarborough	Scarborough General Hospital	Vic Gacek	
Sheffield	Weston Park Hospital	Lucy Walkington	
Sheffield	Doncaster Royal Infirmary	Mymoona Alzouebi	
Sheffield	Weston Park Hospital	Mymoona Alzouebi	
Sheffield	Weston Park Hospital	Prashanth Sanganalmath	
Sheffield	Weston Park Hospital	Roseleen Sheehan	
Sheffield	Weston Park Hospital	Shabbir Rawther	Co-I
Sheffield	Weston Park Hospital	Tathagata Das	
Sheffield	Weston Park Hospital	Virgil Sivoglo	Co-I
Sheffield	Weston Park Hospital	Carmel Pezaro	PI
Sheffield	Weston Park Hospital	Catherine Ferguson	
Sheffield	Weston Park Hospital	James Lester	
Sheffield	Weston Park Hospital	Janet Brown	
Sheffield	Weston Park Hospital	Katie Bowen	
Sheffield	Weston Park Hospital	Linda Evans	
Sheffield	Weston Park Hospital	Louise Murray	
Sheffield	Weston Park Hospital	Omar Din	PI

Sheffield	Weston Park Hospital	Peter Kirkbride	
Sheffield	Weston Park Hospital	Chloe Clegg	
Sheffield	Weston Park Hospital	Georgia Douglas	
Sheffield	Weston Park Hospital	Jessica Medcalf	
Sheffield	Weston Park Hospital	Rebecca Lomax-Allen	
Sheffield	Weston Park Hospital	Steffy George	
Sheffield	Weston Park Hospital	Alexandra Firth	
Sheffield	Weston Park Hospital	Cyper Allan	
Sheffield	Weston Park Hospital	Ryan Asher	
Sheffield	Weston Park Hospital	Ryan Davies	
Sheffield	Weston Park Hospital	John Martindale	
Sheffield	Doncaster Royal Infirmary	Mark Holliday	
Sheffield	Weston Park Hospital	Mark Holliday	
Sheffield	Weston Park Hospital	Richard Brown	
Sheffield	Weston Park Hospital	Roger Burkinshaw	
Sheffield	Weston Park Hospital	Anne Smythe	
Sheffield	Weston Park Hospital	Eileen Marsh	
Sheffield	Weston Park Hospital	Gemma Dale	
Sheffield	Weston Park Hospital	Janine Smedley (nee McCabe)	
Sheffield	Weston Park Hospital	Julia Disney	Pharmacist
Sheffield	Weston Park Hospital	Kate Gibbins	
Sheffield	Weston Park Hospital	Jess Aldred	
Sheffield	Weston Park Hospital	Leigh Fiorentino	
Sheffield	Weston Park Hospital	Lucy Birch	
Sheffield	Weston Park Hospital	Ruta Segamogaite	
Sheffield	Weston Park Hospital	Catherine Spalton	
Sheffield	Weston Park Hospital	Elizabeth Hodgkinson	Pharmacist
Sheffield	Weston Park Hospital	Joanne Bird	
Sheffield	Weston Park Hospital	Katherine Williams	
Sheffield	Weston Park Hospital	Lucy Smith	
Sheffield	Weston Park Hospital	Lynne Ashmore	
Sheffield	Weston Park Hospital	Marion Hutchinson	Pharmacist
Sheffield	Weston Park Hospital	Rachel Toes	
Sheffield	Weston Park Hospital	Sarah Brown	

Sheffield	Weston Park Hospital	Susan Bishop	
Sheffield	Weston Park Hospital	Kim Wood	
Shrewsbury	Royal Shrewsbury Hospital	Gemma Searle	
Shrewsbury	Royal Shrewsbury Hospital	Shazad Aslam	
Shrewsbury	Royal Shrewsbury Hospital	Abel Zachariah	
Shrewsbury	Royal Shrewsbury Hospital	Aitzaz Qaisar	
Shrewsbury	Royal Shrewsbury Hospital	Beshar Allos	
Shrewsbury	Royal Shrewsbury Hospital	Erica Beaumont	
Shrewsbury	Royal Shrewsbury Hospital	Huzeifa Abdel	
Shrewsbury	Royal Shrewsbury Hospital	Huzeifa Gadir	
Shrewsbury	Royal Shrewsbury Hospital	James Best	
Shrewsbury	Royal Shrewsbury Hospital	Lisa Capaldi	
Shrewsbury	Royal Shrewsbury Hospital	Lucy Pennant	
Shrewsbury	Royal Shrewsbury Hospital	Mathai Varghese	
Shrewsbury	Royal Shrewsbury Hospital	Narayanan Srihari	PI
Shrewsbury	Royal Shrewsbury Hospital	Qamar Ghafoor	
Shrewsbury	Royal Shrewsbury Hospital	Rajanee Bhana	
Shrewsbury	Royal Shrewsbury Hospital	Sundus Yahya	
Shrewsbury	Royal Shrewsbury Hospital	Alison Tilley	
Shrewsbury	Royal Shrewsbury Hospital	Anna Law	
Shrewsbury	Royal Shrewsbury Hospital	Danielle Childs	
Shrewsbury	Royal Shrewsbury Hospital	Danielle Childs	
Shrewsbury	Royal Shrewsbury Hospital	Gemma Lee	
Shrewsbury	Royal Shrewsbury Hospital	Jenny Simm	
Shrewsbury	Royal Shrewsbury Hospital	Lisa Evans	
Shrewsbury	Royal Shrewsbury Hospital	Nicola Henderson	
Shrewsbury	Royal Shrewsbury Hospital	Riquella Abbott	
Shrewsbury	Royal Shrewsbury Hospital	Suzanne Pope	
Shrewsbury	Royal Shrewsbury Hospital	Andy Taylor	
Shrewsbury	Royal Shrewsbury Hospital	Michael Leigh	
Shrewsbury	Royal Shrewsbury Hospital	Ravi Prashant	Co-I
Shrewsbury	Royal Shrewsbury Hospital	Craig Pickering	
Shrewsbury	Royal Shrewsbury Hospital	Sanal Jose	
Shrewsbury	Royal Shrewsbury Hospital	Emma Weaver	

Shrewsbury	Royal Shrewsbury Hospital	Mandy Beekes	
Shrewsbury	Royal Shrewsbury Hospital	Natasha Wallbank	
Shrewsbury	Royal Shrewsbury Hospital	Rachel McGregor	
Shrewsbury	Royal Shrewsbury Hospital	Gill Ferguson	Pharmacist
Shrewsbury	Royal Shrewsbury Hospital	Harpreet Singh	
Shrewsbury	Royal Shrewsbury Hospital	Hayley Hughes	
Shrewsbury	Royal Shrewsbury Hospital	Sandra Smith	
Shrewsbury	Royal Shrewsbury Hospital	Sunita Kurian-Downer	
Shrewsbury	Royal Shrewsbury Hospital	Indukala Chennattukungu	
Shrewsbury	Royal Shrewsbury Hospital	Jenny Lakin	
Shrewsbury	Royal Shrewsbury Hospital	Angela Yeomans	Pharmacist
Shrewsbury	Royal Shrewsbury Hospital	Catherine Santiago	
Shrewsbury	Royal Shrewsbury Hospital	Elena Michael	
Shrewsbury	Royal Shrewsbury Hospital	Karen Nicholas	
Shrewsbury	Royal Shrewsbury Hospital	Mandy Bates	
Shrewsbury	Royal Shrewsbury Hospital	Nicola Jones	
Shrewsbury	Royal Shrewsbury Hospital	Rebecca Wilcox	
Shrewsbury	Royal Shrewsbury Hospital	Renee Poulsom	Pharmacist
Shrewsbury	Royal Shrewsbury Hospital	Sally Potts	
Shrewsbury	Royal Shrewsbury Hospital	Siobhan Kilbane	
Shrewsbury	Royal Shrewsbury Hospital	Verity King	
Shrewsbury	Royal Shrewsbury Hospital	Catherine Orrell	
Shrewsbury	Royal Shrewsbury Hospital	Emma Neeves	
Shrewsbury	Royal Shrewsbury Hospital	Helen Moore	
Shrewsbury	Royal Shrewsbury Hospital	Marion Adams	
South Shields	South Tyneside District Hospital	Ashraf Azzabi	PI
South Shields	South Tyneside District Hospital	Sally Hall	
South Shields	South Tyneside District Hospital	Judith Mckenna	
South Shields	South Tyneside District Hospital	Amy Burns	
South Shields	South Tyneside District Hospital	Jessica De Sousa	
South Shields	South Tyneside District Hospital	Judith Moore	
South Shields	South Tyneside District Hospital	Sue Morrison	
South Shields	South Tyneside District Hospital	Maxine Turner	Pharmacist
Southampton	Southampton General Hospital	Caroline Chan	

Southampton	Southampton General Hospital	Caroline Chau	
Southampton	Southampton General Hospital	Chloe Holden	Co-I
Southampton	Southampton General Hospital	Chris Coyle	
Southampton	Southampton General Hospital	Emma Brown	Co-I
Southampton	Southampton General Hospital	Kim Teasdale	Co-I
Southampton	Southampton General Hospital	Matthew Wheater	
Southampton	Southampton General Hospital	Robert Kemp	
Southampton	Southampton General Hospital	Simon Crabb	PI
Southampton	Southampton General Hospital	Tessa Greenhalgh	Co-I
Southampton	Southampton General Hospital	Catherine Heath	
Southampton	Southampton General Hospital	Graham Mead	
Southampton	Southampton General Hospital	Harish Reddy	Co-I
Southampton	Southampton General Hospital	Victoria McFarlane	
Southampton	Southampton General Hospital	Ivanila Atauasova	Pharmacist
Southampton	Southampton General Hospital	Anna Sieradzka	
Southampton	Southampton General Hospital	Anna Stephenson	
Southampton	Southampton General Hospital	Nithya Raj	
Southampton	Southampton General Hospital	Rajitha Kamalakshan	
Southampton	Southampton General Hospital	Rebecca Rice	
Southampton	Southampton General Hospital	Adele Ruiz	
Southampton	Southampton General Hospital	Julie Kennedy	
Southampton	Southampton General Hospital	Susan Morton	
Southampton	Southampton General Hospital	Aneta Zahorska	
Southampton	Southampton General Hospital	Annelise Haskell	
Southampton	Southampton General Hospital	Archana Gadve	
Southampton	Southampton General Hospital	Caroline Andrews	
Southampton	Southampton General Hospital	Carolyn Mitchell	Pharmacist
Southampton	Southampton General Hospital	Deborah Scott	
Southampton	Southampton General Hospital	Holly Burton	Pharmacist
Southampton	Southampton General Hospital	Leanne Reader	
Southampton	Southampton General Hospital	Liane Armstrong	
Southampton	Southampton General Hospital	Lucy Galloway	
Southampton	Southampton General Hospital	Maureen McAuley	Pharmacist
Southampton	Southampton General Hospital	Nikki Carney	

Southampton	Southampton General Hospital	Nikki Prewitt	
Southampton	Southampton General Hospital	Oyeleye Oyebola	
Southampton	Southampton General Hospital	Sarah Oliver	
Southampton	Southampton General Hospital	Sau-Mon Tsang	Pharmacist
Southampton	Southampton General Hospital	Yanli Li	
Southampton	Southampton General Hospital	Carina Mundy	
Southampton	Southampton General Hospital	Donna Kimber	Pharmacist
Southampton	Southampton General Hospital	Fabiola Morales-Azofra	
Southampton	Southampton General Hospital	Julie Gwilt	
Southampton	Southampton General Hospital	Kirsty Cumming	
Southampton	Southampton General Hospital	Lorraine Street	
Southampton	Southampton General Hospital	Lucy Elswood	
Southampton	Southampton General Hospital	Shauna Wakefield	
Southport	Southport and Formby District General Hospital	Manal Alameddine	PI
Southport	Southport and Formby District General Hospital	Neeraj Bhalla	PI
Southport	Southport and Formby District General Hospital	Asha Sivapalasuntharam	
Southport	Southport and Formby District General Hospital	Chinnamani Eswar	
Southport	Southport and Formby District General Hospital	Margaret Brunton	
Southport	Southport and Formby District General Hospital	Ken Gardner	
Southport	Southport and Formby District General Hospital	Laurie Lomax	
Southport	Southport and Formby District General Hospital	Ann Wearing	
Southport	Southport and Formby District General Hospital	Dawn Barker	
Southport	Southport and Formby District General Hospital	Marie McBride	
Southport	Southport and Formby District General Hospital	Sandra Robinson	
Southport	Southport and Formby District General Hospital	Anna Morris	
Southport	Southport and Formby District General Hospital	Heidi Moran	
Southport	Southport and Formby District General Hospital	Teresa Monahan	
Southport	Southport and Formby District General Hospital	Angela Scullion	
Southport	Southport and Formby District General Hospital	Lisa Dobson (nee Child)	
Southport	Southport and Formby District General Hospital	Linda Schinkel	
St Gallen	Kantonsspital St Gallen	Aurelius Omlin	Co-I
St Gallen	Kantonsspital St Gallen	Christoph Schwab	Co-I
St Gallen	Kantonsspital St Gallen	Dominik Abt	
St Gallen	Kantonsspital St Gallen	Christian Rothermundt	Co-I

St Gallen	Kantonsspital St Gallen	Daniel Engeler	PI
St Gallen	Kantonsspital St Gallen	Mannel Jungi	
St Gallen	Kantonsspital St Gallen	Silke Gillessen	
St Gallen	Kantonsspital St Gallen	Stefan Prensser	
St Gallen	Kantonsspital St Gallen	Sigrid Patel	
St Gallen	Kantonsspital St Gallen	Eloise Kremer	
St Gallen	Kantonsspital St Gallen	Karin Zuern	
St Gallen	Kantonsspital St Gallen	Karin Zurn	
St Gallen	Kantonsspital St Gallen	Claudia Hormann	
St Gallen	Kantonsspital St Gallen	Sibylle Schapper	
St Leonards-on-Sea	Conquest Hospital	Aspasia Soultati	
St Leonards-on-Sea	Conquest Hospital	Fiona McKinna	
St Leonards-on-Sea	Conquest Hospital	Kathryn Lees	
St Leonards-on-Sea	Conquest Hospital	Sharon Beesley	
St Leonards-on-Sea	Conquest Hospital	Claire Rutherford	
St Leonards-on-Sea	Conquest Hospital	Sarah Draper	
St Leonards-on-Sea	Conquest Hospital	Mark Whitfield	Pharmacist
St Leonards-on-Sea	Conquest Hospital	Steve Garnett	
St Leonards-on-Sea	Conquest Hospital	Gail Pottinger	
St Leonards-on-Sea	Conquest Hospital	Jo-Anne Taylor	
St Leonards-on-Sea	Conquest Hospital	Kay Jones-Skipper	
St Leonards-on-Sea	Conquest Hospital	Atikah Ayaz	
St Leonards-on-Sea	Conquest Hospital	Lauren McCrisken	
St Leonards-on-Sea	Conquest Hospital	Sarah Goodwin	
St Leonards-on-Sea	Conquest Hospital	Theresa Baumber	
St Leonards-on-Sea	Conquest Hospital	Joanna Howard	
St. Gallen	Klinik fur Urologie	Claudia Hormann	
St. Gallen	Klinik fur Urologie	Sibylle Schapper	
St.Leonards-on-Sea	Conquest Hospital	Roger Plail	
Stevenage	Lister Hospital	Anna Anosova	
Stevenage	Lister Hospital	David Woolf	
Stevenage	Lister Hospital	Mawuelikem Assoku	
Stevenage	Lister Hospital	Nikhil Oommen	
Stevenage	Lister Hospital	Alkhaldi Ashraf	

Stevenage	Lister Hospital	David Ward	
Stevenage	Lister Hospital	Jonathan Towler	
Stevenage	Lister Hospital	Leena Mukherjee	
Stevenage	Lister Hospital	Robert Hughes	PI
Stevenage	Lister Hospital	Steven Watkins	
Stevenage	Lister Hospital	Natalie Rahim	
Stevenage	Lister Hospital	Sayyida Nembhard	
Stevenage	Lister Hospital	Martin Ebon	
Stevenage	Lister Hospital	Stephen Almond	
Stevenage	Lister Hospital	Clare Collins	
Stevenage	Lister Hospital	Jemma Gilmore	
Stevenage	Lister Hospital	Sunita Gohil	
Stevenage	Lister Hospital	Sura Dabbagh	Pharmacist
Stevenage	Lister Hospital	Alice Valle	
Stevenage	Lister Hospital	Corinne Bradshaw	
Stevenage	Lister Hospital	Elen Witness	
Stevenage	Lister Hospital	Rhos Gabriel	
Stevenage	Lister Hospital	Anita Rana	Pharmacist
Stevenage	Lister Hospital	Katie Poole	
Stevenage	Lister Hospital	Roisin Schimmel	
Stevenage	Lister Hospital	Vicky Hills	
Stockport	Stepping Hill Hospital	Carmel Anandadas	
Stockport	Stepping Hill Hospital	Satish Venkateshan	
Stockport	Stepping Hill Hospital	Umi Hatimy	
Stockport	Stepping Hill Hospital	Apurna Jegannathen	
Stockport	Stepping Hill Hospital	Catherine Coyle	
Stockport	Stepping Hill Hospital	John Logue	PI
Stockport	Stepping Hill Hospital	Abigail Mackley	
Stockport	Stepping Hill Hospital	Anna Kellingray	
Stockport	Stepping Hill Hospital	Oluwademilade Odewumi	
Stockport	Stepping Hill Hospital	Sarah Smallwood	
Stockport	Stepping Hill Hospital	Aelens Brauckman	
Stockport	Stepping Hill Hospital	Benjamin Ralphs	
Stockport	Stepping Hill Hospital	Donald van Welsenes	

Stockport	Stepping Hill Hospital	John Kilmartin	
Stockport	Stepping Hill Hospital	Jonathan Wong	Pharmacist
Stockport	Stepping Hill Hospital	Paul Berry	
Stockport	Stepping Hill Hospital	Wasim Akhtar	
Stockport	Stepping Hill Hospital	Adebanji Adeyoju	
Stockport	Stepping Hill Hospital	Andrew Sinclair	
Stockport	Stepping Hill Hospital	David Ross	
Stockport	Stepping Hill Hospital	Gerald Collins	
Stockport	Stepping Hill Hospital	Patrick O'Reilly	
Stockport	Stepping Hill Hospital	Richard Brough	
Stockport	Stepping Hill Hospital	Stephen Bromage	
Stockport	Stepping Hill Hospital	Stephen CW Brown	
Stockport	Stepping Hill Hospital	Helen Haydock	
Stockport	Stepping Hill Hospital	Louise Brown	
Stockport	Stepping Hill Hospital	Susan Hopkins	
Stockport	Stepping Hill Hospital	Abigail Pemberton	
Stockport	Stepping Hill Hospital	Alissa Kent	
Stockport	Stepping Hill Hospital	Emma Goodwin	
Stockport	Stepping Hill Hospital	Emma Taylor	
Stockport	Stepping Hill Hospital	Julie Melville	
Stockport	Stepping Hill Hospital	Katrina Wade	
Stockport	Stepping Hill Hospital	Lucy Orrell	Pharmacist
Stockport	Stepping Hill Hospital	Magda Kujawa	
Stockport	Stepping Hill Hospital	Miriam Avery	
Stockport	Stepping Hill Hospital	Mkyla Reilly	
Stockport	Stepping Hill Hospital	Susan Graham	Pharmacist
Stockport	Stepping Hill Hospital	Zoe Jordan	
Stockport	Stepping Hill Hospital	Catherine Fox	Pharmacist
Stockport	Stepping Hill Hospital	Christina Gilmour	
Stockport	Stepping Hill Hospital	Eleanor Anscombe	
Stockport	Stepping Hill Hospital	Emma Hewitt	Pharmacist
Stockport	Stepping Hill Hospital	Jean Cheetham	
Stockport	Stepping Hill Hospital	Jill Taylor	
Stockport	Stepping Hill Hospital	Nicola Hermitage	

Stockport	Stepping Hill Hospital	Pat Clitheroe	
Stockport	Stepping Hill Hospital	Sam Corcoran	
Stockport	Stepping Hill Hospital	Sarah Connolly nee McKenna	
Stockport	Stepping Hill Hospital	Sheila Hodgkinson	
Stockport	Stepping Hill Hospital	Tracie Cocks	
Stockton on Tees	North Tees General Hospital	Devadasan Shakespeare	
Stockton-on-Tees	University Hospital of North Tees	Abdul Mian	
Stockton-on-Tees	University Hospital of North Tees	Darren Leaning	PI
Stockton-on-Tees	University Hospital of North Tees	Jenny Smith	
Stockton-on-Tees	University Hospital of North Tees	Devadasan Shakespeare	
Stockton-on-Tees	University Hospital of North Tees	Gaurav Kumar	
Stockton-on-Tees	University Hospital of North Tees	Gala Stancev Stevanovic	
Stockton-on-Tees	University Hospital of North Tees	Helen Wardle (nee Wilson)	
Stockton-on-Tees	University Hospital of North Tees	Moira Percival	
Stockton-on-Tees	University Hospital of North Tees	Andrew Sigsworth	
Stockton-on-Tees	University Hospital of North Tees	Bill Wetherill	Pharmacist
Stockton-on-Tees	University Hospital of North Tees	Victor Palit	
Stockton-on-Tees	University Hospital of North Tees	Hyder Latif	
Stockton-on-Tees	University Hospital of North Tees	Helen Dunn (nee Carey)	Pharmacist
Stockton-on-Tees	University Hospital of North Tees	Jeanette Naisbitt	
Stockton-on-Tees	University Hospital of North Tees	Pam Race	
Stockton-on-Tees	University Hospital of North Tees	Sarah Pitcairn	
Stockton-on-Tees	University Hospital of North Tees	Alison Chilvers	
Stockton-on-Tees	University Hospital of North Tees	Leigh Pollard	
Stockton-on-Tees	University Hospital of North Tees	Emma Jameson	
Stockton-on-Tees	University Hospital of North Tees	Lynda Poole	
Stoke-on-Trent	Royal Stoke University Hospital	Sumera Butt	
Stoke-on-Trent	Royal Stoke University Hospital	Fawzi Adab	
Stoke-on-Trent	Royal Stoke University Hospital	Rajanee Bhana	
Stoke-on-Trent	Royal Stoke University Hospital	Salil Vengalil	PI
Stoke-on-Trent	Royal Stoke University Hospital	Elizabeth Sellars	
Stoke-on-Trent	Royal Stoke University Hospital	Emma Jackson	
Stoke-on-Trent	Royal Stoke University Hospital	Christopher Luscombe	
Stoke-on-Trent	Royal Stoke University Hospital	Robert Green	

Stoke-on-Trent	Royal Stoke University Hospital	Liberty Verueco	
Stoke-on-Trent	Royal Stoke University Hospital	Sharon Rollison	
Stoke-on-Trent	Royal Stoke University Hospital	Alison Tute	Pharmacist
Stoke-on-Trent	Royal Stoke University Hospital	Angela Peake	
Stoke-on-Trent	Royal Stoke University Hospital	Angela Ward	
Stoke-on-Trent	Royal Stoke University Hospital	Marion Evans	
Stoke-on-Trent	Royal Stoke University Hospital	Eden Ball	
Stoke-on-Trent	Royal Stoke University Hospital	Alison Myatt	
Stoke-on-Trent	Royal Stoke University Hospital	Elizabeth Williamson	
Stoke-on-Trent	Royal Stoke University Hospital	Grace Gough	
Stoke-on-Trent	Royal Stoke University Hospital	Isabel Breeze	Pharmacist
Stoke-on-Trent	Royal Stoke University Hospital	Julie Storer	
Stoke-on-Trent	Royal Stoke University Hospital	Katrina Parkinson	
Stoke-on-Trent	Royal Stoke University Hospital	Rowena Smith	
Sunderland	Sunderland Royal Hospital	Rachel Pearson	Co-I
Sunderland	Sunderland Royal Hospital	Shahid Iqbal	
Sunderland	Sunderland Royal Hospital	Ashraf Azzabi	PI
Sunderland	Sunderland Royal Hospital	Ian Pedley	Co-I
Sunderland	Sunderland Royal Hospital	Kathryn Wright	Co-I
Sunderland	Sunderland Royal Hospital	Stephen Laybourne	
Sunderland	Sunderland Royal Hospital	Rod Beard	Pharmacist
Sunderland	Sunderland Royal Hospital	Stephen Butler	
Sunderland	Sunderland Royal Hospital	Amanda Howey	
Sunderland	Sunderland Royal Hospital	Fiona Wakinshaw	
Sunderland	Sunderland Royal Hospital	Jane Cole	
Sunderland	Sunderland Royal Hospital	Paula Newton	
Sunderland	Sunderland Royal Hospital	Terri Haldane	
Sunderland	Sunderland Royal Hospital	Christine Harle	
Sunderland	Sunderland Royal Hospital	Fiona Wakinshaw	
Sunderland	Sunderland Royal Hospital	Julia Scott	
Sunderland	Sunderland Royal Hospital	Karen Shield	Pharmacist
Sunderland	Sunderland Royal Hospital	Michelle Edwards	Pharmacist
Sunderland	Sunderland Royal Hospital	Vivienne Hullock	
Sutton	Royal Marsden Hospital (Sutton)	Adham Hijab	

Sutton	Royal Marsden Hospital (Sutton)	Alex Tan	
Sutton	Royal Marsden Hospital (Sutton)	Angela Pathmanathan	Co-I
Sutton	Royal Marsden Hospital (Sutton)	Gerard McVey	
Sutton	Royal Marsden Hospital (Sutton)	Nora Sundahl	Co-I
Sutton	Royal Marsden Hospital (Sutton)	Susan Lalondrelle	
Sutton	Royal Marsden Hospital (Sutton)	Alison Tree	
Sutton	Royal Marsden Hospital (Sutton)	Chris Parker	PI
Sutton	Royal Marsden Hospital (Sutton)	Ray Shepherd	
Sutton	Royal Marsden Hospital (Sutton)	Robert Huddart	
Sutton	Royal Marsden Hospital (Sutton)	Rosalind Eeles	
Sutton	Royal Marsden Hospital (Sutton)	Vincent Khoo	
Sutton	Royal Marsden Hospital (Sutton)	Lucy Featherstone	Pharmacist
Sutton	Royal Marsden Hospital (Sutton)	Victoria Sjolin	Pharmacist
Sutton	Royal Marsden Hospital (Sutton)	Martha Bullimore	
Sutton	Royal Marsden Hospital (Sutton)	Alexander Macnab	
Sutton	Royal Marsden Hospital (Sutton)	Amir El Ghazal	
Sutton	Royal Marsden Hospital (Sutton)	Douglas Brand	Co-I
Sutton	Royal Marsden Hospital (Sutton)	Nick Hunnings	Pharmacist
Sutton	Royal Marsden Hospital (Sutton)	Tiaan Jacobs	
Sutton	Royal Marsden Hospital (Sutton)	Ruth Woode-Amissah	
Sutton	Royal Marsden Hospital (Sutton)	Zaynah Gurreebun	
Sutton	Royal Marsden Hospital (Sutton)	Sally Moore	
Sutton	Royal Marsden Hospital (Sutton)	Annette Musallam	
Sutton	Royal Marsden Hospital (Sutton)	Claire Crowley	
Sutton	Royal Marsden Hospital (Sutton)	Helen Stidwell	
Sutton	Royal Marsden Hospital (Sutton)	Janine Flohr	
Sutton	Royal Marsden Hospital (Sutton)	Jenni Parmar	
Sutton	Royal Marsden Hospital (Sutton)	Kelly Jones	
Sutton	Royal Marsden Hospital (Sutton)	Kirsty Cuthbertson	
Sutton	Royal Marsden Hospital (Sutton)	Laura Hennelly	
Sutton	Royal Marsden Hospital (Sutton)	Rookmeen Alighan	
Sutton	Royal Marsden Hospital (Sutton)	Bernadette Johnson	
Sutton	Royal Marsden Hospital (Sutton)	Chloe McCormack	
Sutton	Royal Marsden Hospital (Sutton)	Fatima Ahmed	

Sutton	Royal Marsden Hospital (Sutton)	Sue Cromarty	Pharmacist
Sutton	Royal Marsden Hospital (Sutton)	Alan Horwich	
Sutton	Royal Marsden Hospital (Sutton)	David Dearnaley	
Sutton Coldfield	Good Hope Hospital	Mark O'Beirn	Co-I
Sutton Coldfield	Good Hope Hospital	Daniel Ford	PI
Sutton Coldfield	Good Hope Hospital	Lorna Swaddle	
Sutton Coldfield	Good Hope Hospital	Steve Hay	
Sutton Coldfield	Good Hope Hospital	Chen Bartlett	
Sutton Coldfield	Good Hope Hospital	James Whitehouse	
Sutton Coldfield	Good Hope Hospital	Helen Thomas	
Sutton Coldfield	Good Hope Hospital	Ellen Drew	
Sutton Coldfield	Good Hope Hospital	Ann Schumacher	
Sutton Coldfield	Good Hope Hospital	Arlene Oldan	
Sutton Coldfield	Good Hope Hospital	Beena Mistry	
Sutton Coldfield	Good Hope Hospital	Helen Taylor	
Sutton Coldfield	Good Hope Hospital	Janet Prentice	
Sutton Coldfield	Good Hope Hospital	Kamaldeep Ajimal	
Sutton Coldfield	Good Hope Hospital	Rachael O'Beney	
Sutton Coldfield	Good Hope Hospital	Sarah Rogers	
Sutton Coldfield	Good Hope Hospital	Shobit Baijal	
Sutton Coldfield	Good Hope Hospital	Sundip Sohanpal	
Sutton Coldfield	Good Hope Hospital	Alison Maidment	Pharmacist
Sutton Coldfield	Good Hope Hospital	Katy Moore	Pharmacist
Sutton Coldfield	Good Hope Hospital	Lubna Khan	
Sutton-in-Ashfield	King's Mill Hospital	Andrew Brocklehurst	
Sutton-in-Ashfield	King's Mill Hospital	Elena Macleod	
Sutton-in-Ashfield	King's Mill Hospital	Fiona Smith	
Sutton-in-Ashfield	King's Mill Hospital	James Price	
Sutton-in-Ashfield	King's Mill Hospital	Jun Lim	
Sutton-in-Ashfield	King's Mill Hospital	Lauren Jones	
Sutton-in-Ashfield	King's Mill Hospital	Michael Ocathail	
Sutton-in-Ashfield	King's Mill Hospital	Muhammad Gill	
Sutton-in-Ashfield	King's Mill Hospital	Robert Goldspring	
Sutton-in-Ashfield	King's Mill Hospital	Sadia Abdullah	

Sutton-in-Ashfield	King's Mill Hospital	Sarah Taylor	
Sutton-in-Ashfield	King's Mill Hospital	Wai Hou Sam	
Sutton-in-Ashfield	King's Mill Hospital	Benjamin Masters	Co-I
Sutton-in-Ashfield	King's Mill Hospital	Daniel Saunders	PI
Sutton-in-Ashfield	King's Mill Hospital	Eliot Chadwick	
Sutton-in-Ashfield	King's Mill Hospital	Georgina Walker	PI
Sutton-in-Ashfield	King's Mill Hospital	Louise Brookes	Co-I
Sutton-in-Ashfield	King's Mill Hospital	Andrea Palfreman	
Sutton-in-Ashfield	King's Mill Hospital	Jamie-Rae Burgoyne	
Sutton-in-Ashfield	King's Mill Hospital	Lisa Rahn	
Sutton-in-Ashfield	King's Mill Hospital	Victoria Moore	
Sutton-in-Ashfield	King's Mill Hospital	Shila Hamzepur	
Sutton-in-Ashfield	King's Mill Hospital	Terri-Ann Sewell	
Sutton-in-Ashfield	King's Mill Hospital	Wayne Lovegrove	
Sutton-in-Ashfield	King's Mill Hospital	Dominic Nash	
Sutton-in-Ashfield	King's Mill Hospital	Steve Haigh	Pharmacist
Sutton-in-Ashfield	King's Mill Hospital	Inez Wynter	
Sutton-in-Ashfield	King's Mill Hospital	Katie Slack	
Sutton-in-Ashfield	King's Mill Hospital	Sarah Shelton	
Sutton-in-Ashfield	King's Mill Hospital	Margaret Wheatley	
Sutton-in-Ashfield	King's Mill Hospital	Samantha Boam	
Sutton-in-Ashfield	King's Mill Hospital	Samantha March	Pharmacist
Sutton-in-Ashfield	King's Mill Hospital	Linda Otter	Pharmacist
Sutton-in-Ashfield	King's Mill Hospital	Lynne Wade	
Sutton-in-Ashfield	King's Mill Hospital	Rebecca Holmes	
Sutton-in-Ashfield	King's Mill Hospital	Susan Smith	
Swansea	Singleton Hospital	Ahmed Shaheen	PI
Swansea	Singleton Hospital	Aijaz Lone	Co-I
Swansea	Singleton Hospital	David Brown	
Swansea	Singleton Hospital	Delia Pudney	
Swansea	Singleton Hospital	Fiona Williams	Co-I
Swansea	Singleton Hospital	Helen Fitzgerald	Co-I
Swansea	Singleton Hospital	N Sindgi	
Swansea	Singleton Hospital	Nia Jackson	Co-I

Swansea	Singleton Hospital	Rhian Davies	Co-I
Swansea	Singleton Hospital	Ricky Fraser	
Swansea	Singleton Hospital	Russell Banner	
Swansea	Singleton Hospital	Sarah Gwynne	
Swansea	Singleton Hospital	Satish Kumar	
Swansea	Singleton Hospital	Sharath Gangadhara	
Swansea	Singleton Hospital	Sheena Lam	Co-I
Swansea	Singleton Hospital	Wael Mohamed	Co-I
Swansea	Singleton Hospital	Gianfilippo Bertelli	
Swansea	Singleton Hospital	Jason Lester	
Swansea	Singleton Hospital	Mau-Don Phan	Co-I
Swansea	Singleton Hospital	Nicola Davies	
Swansea	Singleton Hospital	Angharad Phillips	
Swansea	Singleton Hospital	Anne Thomas	Pharmacist
Swansea	Singleton Hospital	Bethan Williams	
Swansea	Singleton Hospital	Ellen Tait	
Swansea	Singleton Hospital	Karen Chesters	
Swansea	Singleton Hospital	Katie Tanner	
Swansea	Singleton Hospital	Naomi Woods	
Swansea	Singleton Hospital	Nicola Lemon	
Swansea	Singleton Hospital	Alex Richards	
Swansea	Singleton Hospital	Carl Ackland	
Swansea	Singleton Hospital	James Morgan	
Swansea	Singleton Hospital	Lewis Jones	
Swansea	Singleton Hospital	Mark Rogers	
Swansea	Singleton Hospital	Stuart Evans	Pharmacist
Swansea	Singleton Hospital	Ashok Kumar	
Swansea	Singleton Hospital	Brian Phillips	
Swansea	Singleton Hospital	Euan Pratt	
Swansea	Singleton Hospital	Amanda Cook	
Swansea	Singleton Hospital	Charlotte Young	
Swansea	Singleton Hospital	Dawn Withers	
Swansea	Singleton Hospital	Gillian Palmer	
Swansea	Singleton Hospital	Gillian Willetts	

Swansea	Singleton Hospital	Helen Cheley	
Swansea	Singleton Hospital	Lesley Richards	
Swansea	Singleton Hospital	Lynne Breeze-Jones	
Swansea	Singleton Hospital	Alex Franklin	
Swansea	Singleton Hospital	Gillian Jones	Pharmacist
Swansea	Singleton Hospital	Karen Phillips	
Swansea	Singleton Hospital	Mair Roberts	Pharmacist
Swansea	Singleton Hospital	Amy Quinton	
Swansea	Singleton Hospital	Chelsea Jenkins	
Swansea	Singleton Hospital	Elizabeth Evans	
Swansea	Singleton Hospital	Emily Harris (n. Marchant)	
Swansea	Singleton Hospital	Emma Dangerfield	
Swansea	Singleton Hospital	Judith Gooding	Pharmacist
Swansea	Singleton Hospital	Maria Johnstone	
Swansea	Singleton Hospital	Michelle Romano	
Swansea	Singleton Hospital	Renata Poole	Pharmacist
Swansea	Singleton Hospital	Alison Stretch	
Swansea	Singleton Hospital	Amanda Jackson	
Swansea	Singleton Hospital	Donna Lear	
Swansea	Singleton Hospital	Gail Povey	
Swansea	Singleton Hospital	Jayne Caparros	
Swansea	Singleton Hospital	Leanne Quinn	
Swansea	Singleton Hospital	Lisa Ellis	
Swansea	Singleton Hospital	Lorraine Gammon	
Swansea	Singleton Hospital	Nia Viney	
Swansea	Singleton Hospital	Susie Pitcher	
Swansea	Singleton Hospital	Tracey Ford	
Swansea	Singleton Hospital	John Wagstaff	
Swindon	Great Western Hospital	Dorota Marciniak	
Swindon	Great Western Hospital	Raj Jampana	
Swindon	Great Western Hospital	Aruna Medisetti	
Swindon	Great Western Hospital	David J Cole	
Swindon	Great Western Hospital	Dorothe Maramak	
Swindon	Great Western Hospital	Esme Hill	

Swindon	Great Western Hospital	Gerard Andrade	
Swindon	Great Western Hospital	Omar Khan	PI
Swindon	Great Western Hospital	Shiroma De Silva-Minor	
Swindon	Great Western Hospital	Victoria Gibson	
Swindon	Great Western Hospital	Emma Wakefield	Pharmacist
Swindon	Great Western Hospital	Graham Brown	
Swindon	Great Western Hospital	Joseph Stevens	
Swindon	Great Western Hospital	Ronak Patel	
Swindon	Great Western Hospital	Tim Owen	
Swindon	Great Western Hospital	Christopher Clarke	
Swindon	Great Western Hospital	David Newell	
Swindon	Great Western Hospital	Jonathan Lewis	
Swindon	Great Western Hospital	Mike Lewis	
Swindon	Great Western Hospital	Vivian Zinyemba	
Swindon	Great Western Hospital	Chanelle Meyer	
Swindon	Great Western Hospital	Karen Smith	
Swindon	Great Western Hospital	Rebecca Belcher	
Swindon	Great Western Hospital	Sarah Cotton	
Swindon	Great Western Hospital	Sue Meakin	
Swindon	Great Western Hospital	Amanda Colston	
Swindon	Great Western Hospital	Caroline Pensotti	
Swindon	Great Western Hospital	Debbie Palmer	
Swindon	Great Western Hospital	Jean Kordula	
Swindon	Great Western Hospital	Laura McCafferty	
Swindon	Great Western Hospital	Lesley Haxton	
Swindon	Great Western Hospital	Sarah Grayland	
Swindon	Great Western Hospital	Sarah Long	
Swindon	Great Western Hospital	Catherine Lewis Clarke	
Swindon	Great Western Hospital	Cerila Parajes	
Swindon	Great Western Hospital	Deborah Scott	
Swindon	Great Western Hospital	Donna Lake	
Swindon	Great Western Hospital	Fahad Fazal	
Swindon	Great Western Hospital	Rachel Messenger	
Swindon	Great Western Hospital	Suzannah Pegler	

Swindon	Great Western Hospital	Aiste Baltramaityte	
Swindon	Great Western Hospital	Ania Jones	
Swindon	Great Western Hospital	Ellen Starling	
Swindon	Great Western Hospital	Ellie Hewitt	
Swindon	Great Western Hospital	Nicola Cowling	
Swindon	Great Western Hospital	Sally-Ann Parkin (nee) Lee	
Swindon	Great Western Hospital	Tracey Sargent	
Swindon	Great Western Hospital	Helen Winter	
Swindon	Great Western Hospital	Jan Dodge	
Swindon	Great Western Hospital	Tracey Sargent	
Taunton	Musgrove Park Hospital	Clair Brunner	
Taunton	Musgrove Park Hospital	Elena Macleod	
Taunton	Musgrove Park Hospital	Hannah Berry	
Taunton	Musgrove Park Hospital	Joanne Botten	
Taunton	Musgrove Park Hospital	Manivannan Periasamy	
Taunton	Musgrove Park Hospital	Mohini Varughese	Co-I
Taunton	Musgrove Park Hospital	Nicola Cox	Co-I
Taunton	Musgrove Park Hospital	Olivia Fraser	
Taunton	Musgrove Park Hospital	Darren Brady	
Taunton	Musgrove Park Hospital	Emma Gray	PI
Taunton	Musgrove Park Hospital	George Plataniotis	
Taunton	Musgrove Park Hospital	Gihan Ratnayake	
Taunton	Musgrove Park Hospital	John Graham	PI
Taunton	Musgrove Park Hospital	Joseph Jelski	
Taunton	Musgrove Park Hospital	Manjusha Keni	
Taunton	Musgrove Park Hospital	Mary Tighe	
Taunton	Musgrove Park Hospital	Rebecca Denslow	Co-I
Taunton	Musgrove Park Hospital	Robert Zorica	
Taunton	Musgrove Park Hospital	Abby Farzaneh	
Taunton	Musgrove Park Hospital	Amy Sawyer	
Taunton	Musgrove Park Hospital	Christina Branfield	
Taunton	Musgrove Park Hospital	Corinne Pawley	
Taunton	Musgrove Park Hospital	Hayley Cornall	
Taunton	Musgrove Park Hospital	Jasmine Youens	

Taunton	Musgrove Park Hospital	Odunayo Kalejaiye	
Taunton	Musgrove Park Hospital	Rachel Coe	
Taunton	Musgrove Park Hospital	Rebecca Brown	
Taunton	Musgrove Park Hospital	Rebecca Twemlow	
Taunton	Musgrove Park Hospital	Guillermo Reina-Ruiz	
Taunton	Musgrove Park Hospital	John Allinson-Smith	
Taunton	Musgrove Park Hospital	Joshua Woollven	
Taunton	Musgrove Park Hospital	Lee Talbot	
Taunton	Musgrove Park Hospital	Luke Stephens	Pharmacist
Taunton	Musgrove Park Hospital	Tamlyn Russell	
Taunton	Musgrove Park Hospital	Ian Bodger	
Taunton	Musgrove Park Hospital	Jarrod Dunn	Pharmacist
Taunton	Taunton and Somerset Hospital	Jarrod Dunn	Pharmacist
Taunton	Musgrove Park Hospital	Richard Burgess	
Taunton	Musgrove Park Hospital	Ruaraidh MacDonagh	
Taunton	Taunton and Somerset Hospital	Ruaraidh MacDonagh	
Taunton	Musgrove Park Hospital	Simon Goldsworthy	
Taunton	Musgrove Park Hospital	Alison Snell	
Taunton	Musgrove Park Hospital	Amanda Groves	
Taunton	Musgrove Park Hospital	Ceri Poyntz-wright	
Taunton	Musgrove Park Hospital	Clair Hinton	
Taunton	Musgrove Park Hospital	Claire Sowerby	
Taunton	Musgrove Park Hospital	Fiona Goodchild	
Taunton	Musgrove Park Hospital	Francesca Allen	
Taunton	Musgrove Park Hospital	Joanne Rogers	
Taunton	Musgrove Park Hospital	Joanne Taylor	
Taunton	Musgrove Park Hospital	Lynn Leat	
Taunton	Musgrove Park Hospital	Sue Mahoney	
Taunton	Musgrove Park Hospital	Susan Crouch	
Taunton	Musgrove Park Hospital	Michelle Farrar	
Taunton	Musgrove Park Hospital	Alison Whitcher	
Taunton	Musgrove Park Hospital	Anna Masamba	
Taunton	Musgrove Park Hospital	Catherine Lane	
Taunton	Musgrove Park Hospital	Christine Webster	

Taunton	Musgrove Park Hospital	Fen Lewen	
Taunton	Musgrove Park Hospital	Joan Kemp	Pharmacist
Taunton	Musgrove Park Hospital	Karen Tanner	
Taunton	Musgrove Park Hospital	Martha Wrigley	
Taunton	Musgrove Park Hospital	Moira Tait	
Taunton	Musgrove Park Hospital	Nicola Cutmore	
Taunton	Musgrove Park Hospital	Rebecca Purnell	
Taunton	Musgrove Park Hospital	Samantha Northover	
Taunton	Musgrove Park Hospital	Sara Green	
Taunton	Musgrove Park Hospital	Sara Myers	
Taunton	Musgrove Park Hospital	Alison Chedham	
Taunton	Musgrove Park Hospital	Angela Locke	
Taunton	Musgrove Park Hospital	Jan Ashcroft	
Taunton	Taunton and Somerset Hospital	Jan Ashcroft	
Taunton	Musgrove Park Hospital	Joy Rowe	Pharmacist
Taunton	Musgrove Park Hospital	Judith Mathie	
Taunton	Taunton and Somerset Hospital	Judith Mathie	
Taunton	Musgrove Park Hospital	Lisa Bowern	
Taunton	Musgrove Park Hospital	Lucy Howell-Drewett	
Taunton	Musgrove Park Hospital	Nita Beacham	
Taunton	Musgrove Park Hospital	Rebecca Tucker	
Taunton	Taunton and Somerset Hospital	Rebecca Tucker	
Taunton	Musgrove Park Hospital	Rebecca Wallbutton	
Tessin	Istituto Oncologico della Svizzera Italiana	Anna Llado	
Tessin	Istituto Oncologico della Svizzera Italiana	Enrico Roggero	PI
Tessin	Istituto Oncologico della Svizzera Italiana	Barbara Marongiu	
Tessin	Istituto Oncologico della Svizzera Italiana	Michele Moro	
Tessin	Istituto Oncologico della Svizzera Italiana	Sabine Van Den Bosch	
Thornton Heath	Croydon University Hospital	Jane Thomson	
Torquay	Torbay District General Hospital	Helen Saxby	
Torquay	Torbay District General Hospital	Victoria Bell	
Torquay	Torbay District General Hospital	Victoria Cope	
Torquay	Torbay District General Hospital	Anna Lydon	PI
Torquay	Torbay District General Hospital	Beverley Watkins	

Torquay	Torbay District General Hospital	Erica Watts	
Torquay	Torbay District General Hospital	Fiona Roberts	Co-I
Torquay	Torbay District General Hospital	Jorg Michels	
Torquay	Torbay District General Hospital	Rajaguru Srinivasan	
Torquay	Torbay District General Hospital	Emily Flavell	
Torquay	Torbay District General Hospital	Helen Kimber	
Torquay	Torbay District General Hospital	Kirsty Jones	
Torquay	Torbay District General Hospital	Kirsty Pearce	
Torquay	Torbay District General Hospital	Sally Wells	Pharmacist
Torquay	Torbay District General Hospital	Sophie Gittus	
Torquay	Torbay District General Hospital	Stacey Davies	
Torquay	Torbay District General Hospital	Linda Welsh	
Torquay	Torbay District General Hospital	Kenneth Almedilla	
Torquay	Torbay District General Hospital	Lee Merry	
Torquay	Torbay District General Hospital	Peter Fletcher	
Torquay	Torbay District General Hospital	Peter Pugh	
Torquay	Torbay District General Hospital	Seamus McDermott	
Torquay	Torbay District General Hospital	Andrew Harford-Brown	
Torquay	Torbay District General Hospital	Magdi Kirollos	
Torquay	Torbay District General Hospital	Mark Brennan	
Torquay	Torbay District General Hospital	Martyn Blundell	Pharmacist
Torquay	Torbay District General Hospital	Robert Mason	
Torquay	Torbay District General Hospital	Angela Foulds	
Torquay	Torbay District General Hospital	Kirsty Lester	
Torquay	Torbay District General Hospital	Lauren Blunt	
Torquay	Torbay District General Hospital	Michele Allison	
Torquay	Torbay District General Hospital	Shelley Chamberlain	
Torquay	Torbay District General Hospital	Tyler Lowe	
Torquay	Torbay District General Hospital	Christine Rawlings	
Torquay	Torbay District General Hospital	Donna Cuffe	
Torquay	Torbay District General Hospital	Elaine Vandecandalaere	
Torquay	Torbay District General Hospital	Amy Millington	
Torquay	Torbay District General Hospital	Catherine Brookman	
Torquay	Torbay District General Hospital	Catherine Marshall	

Torquay	Torbay District General Hospital	Classy Lam	
Torquay	Torbay District General Hospital	Emmie Arbury	
Torquay	Torbay District General Hospital	Fleur Rogers	
Torquay	Torbay District General Hospital	Janet Palmer	
Torquay	Torbay District General Hospital	Petra Gee	
Torquay	Torbay District General Hospital	Sally Maddison	
Torquay	Torbay District General Hospital	Sarah Rees	
Torquay	Torbay District General Hospital	Sophie Norman	
Torquay	Torbay District General Hospital	Sue Forbes	
Torquay	Torbay District General Hospital	Amanda Vian	
Torquay	Torbay District General Hospital	Helen Greedus	
Torquay	Torbay District General Hospital	Ingrid Koehler	
Torquay	Torbay District General Hospital	Jo Blurton	
TORQUAY	Torbay District General Hospital	Lorraine Thornton	
Torquay	Torbay District General Hospital	Louise Paatz	Pharmacist
Torquay	Torbay District General Hospital	Lyn Micklewright	
Torquay	Torbay District General Hospital	Melody Cross	
Torquay	Torbay District General Hospital	Sarah Wright	
Torquay	Torbay District General Hospital	Victoria Bell	

INDUSTRY COLLABORATORS

Clovis Oncology
Support for the STAMPEDE trial has been provided by Clovis Oncology.

Janssen
Laurent Antoni
Joaquin Casariego garcia luben
Ilde Herrygars
Florence Lefresne
Rod Murphy
Mohamed Samir
Hind Stitou
Support for the STAMPEDE trial has been provided by Janssen.

Novartis
Support for the STAMPEDE trial has been provided by Novartis Pharmaceuticals UK Limited.

Sanofi-Aventis
Paul Cadle
Christine Geffriaud-Ricouard
Zsuzsanna Devecseri
Support for the STAMPEDE study has been provided by Sanofi-Aventis.

Pfizer
Pam Brambles
Support for the STAMPEDE study has been provided Pfizer

Astellas
John Galfin
Karla Martins
Gunther Boysen
Padraig Moran
Support for the STAMPEDE study has been provided by astellas

PARTICIPANTS

Nearly 12,000 people have chosen to participate in STAMPEDE. In addition to their clinical teams, they have been supported by family, friends and other key people. Every person who has participated in the trial is appreciated by the trial team and should be appreciated by the wider public. The findings from clinical trials can change practice for the future, but clinical trials only happen because people find the time and make the effort to support them. Thank you.
